# Efficacy and Safety of the RTS,S/AS01 Malaria Vaccine during 18 Months after Vaccination: A Phase 3 Randomized, Controlled Trial in Children and Young Infants at 11 African Sites

**DOI:** 10.1371/journal.pmed.1001685

**Published:** 2014-07-29

**Authors:** The RTS,S Clinical Trials Partnership (2014)

**Affiliations:** St. George's, University of London, United Kingdom

## Abstract

**Background:**

A malaria vaccine could be an important addition to current control strategies. We report the safety and vaccine efficacy (VE) of the RTS,S/AS01 vaccine during 18 mo following vaccination at 11 African sites with varying malaria transmission.

**Methods and Findings:**

6,537 infants aged 6–12 wk and 8,923 children aged 5–17 mo were randomized to receive three doses of RTS,S/AS01 or comparator vaccine.

VE against clinical malaria in children during the 18 mo after vaccine dose 3 (per protocol) was 46% (95% CI 42% to 50%) (range 40% to 77%; VE, *p<*0.01 across all sites). VE during the 20 mo after vaccine dose 1 (intention to treat [ITT]) was 45% (95% CI 41% to 49%). VE against severe malaria, malaria hospitalization, and all-cause hospitalization was 34% (95% CI 15% to 48%), 41% (95% CI 30% to 50%), and 19% (95% CI 11% to 27%), respectively (ITT).

VE against clinical malaria in infants was 27% (95% CI 20% to 32%, per protocol; 27% [95% CI 21% to 33%], ITT), with no significant protection against severe malaria, malaria hospitalization, or all-cause hospitalization.

Post-vaccination anti-circumsporozoite antibody geometric mean titer varied from 348 to 787 EU/ml across sites in children and from 117 to 335 EU/ml in infants (per protocol).

VE waned over time in both age categories (Schoenfeld residuals *p*<0.001). The number of clinical and severe malaria cases averted per 1,000 children vaccinated ranged across sites from 37 to 2,365 and from −1 to 49, respectively; corresponding ranges among infants were −10 to 1,402 and −13 to 37, respectively (ITT). Meningitis was reported as a serious adverse event in 16/5,949 and 1/2,974 children and in 9/4,358 and 3/2,179 infants in the RTS,S/AS01 and control groups, respectively.

**Conclusions:**

RTS,S/AS01 prevented many cases of clinical and severe malaria over the 18 mo after vaccine dose 3, with the highest impact in areas with the greatest malaria incidence. VE was higher in children than in infants, but even at modest levels of VE, the number of malaria cases averted was substantial. RTS,S/AS01 could be an important addition to current malaria control in Africa.

**Trial registration:**

www.ClinicalTrials.gov NCT00866619

*Please see later in the article for the Editors' Summary*

## Abstract 2

Mary Hamel and colleagues in the RTS,S Clinical Trials Partnership report updated safety and efficacy results from an ongoing Phase 3 trial, including calculations of vaccine impact (malaria cases prevented).

*Please see later in the article for the Editors' Summary*

## Editors' Summary

**Background:**

Every year, more than 200 million cases of malaria occur worldwide, and more than 600,000 people, mainly children living in sub-Saharan Africa, die from this parasitic disease. Malaria parasites are transmitted to people through the bites of infected night-flying mosquitoes and cause fever that needs to be treated promptly with anti-malarial drugs to prevent anemia (a reduction in red blood cell numbers) and life-threatening organ damage. Malaria transmission can be prevented by using long-lasting insecticides sprayed on the indoor walls of homes to kill the mosquitoes that spread the malaria parasite or by sleeping under insecticide-treated nets to avoid mosquito bites and further reduce mosquito numbers. Widespread use of these preventative measures, together with the introduction of artemisinin combination therapy (an effective anti-malarial treatment), has reduced the global burden of malaria by 45% in all age groups, and by 51% among young children, since 2000.

**Why Was This Study Done?:**

Unfortunately, the emergence of insecticide and drug resistance is threatening this advance in malaria control. Moreover, additional interventions—specifically, effective malaria vaccines—will be needed to eliminate malaria in the large areas of Africa where malaria transmission remains high. Currently, there is no licensed malaria vaccine, but RTS,S/AS01, the most advanced malaria vaccine candidate, is undergoing phase 3 clinical trials (the last stage of testing before licensing) in infants and children in seven African countries. The RTS,S Clinical Trials Partnership reported encouraging results on the efficacy and safety of RTS,S/AS01 during 12 months of follow-up in 2011 and 2012. Here, researchers report on the 18-month efficacy and safety of RTS,S/AS01. Vaccine efficacy (VE) is the reduction in the incidence of a disease (the number of new cases that occur in a population in a given period) among trial participants who receive the vaccine compared to the incidence among participants who do not receive the vaccine.

**What Did the Researchers Do and Find?:**

The researchers randomly assigned 6,537 infants aged 6–12 weeks and 8,923 children aged 5–17 months to receive three doses of RTS,S/AS01 or a control vaccine. During 18 months of follow-up, there were 0.69 episodes of clinical malaria (a high temperature and parasites in the blood) per person-year among the children who received all the planned doses of RTS,S/AS01 (the “per protocol” population) and 1.17 episodes per person-year among the control children—a VE against clinical malaria in the per-protocol population of 46%. A similar VE was seen in an intention-to-treat analysis that included all the enrolled children, regardless of whether they received all of the planned vaccine doses; intention-to-treat analyses reflect the real-life situation—in which children sometimes miss vaccine doses—better than per-protocol analyses. In intention-to-treat analyses, the VE among children against severe malaria (fever, parasites in the blood, and symptoms such as anemia) and hospitalization for malaria was 34% and 41%, respectively. Among infants, the VE against clinical malaria was 27% in both per-protocol and intention-to-treat analyses; the vaccine showed no protection against severe malaria or hospitalization. In both infants and children, VE waned with time since vaccination. Across all the study sites, RTS,S/AS01 averted an average of 829 and 449 cases of clinical malaria per 1,000 children and infants vaccinated, respectively. Finally, the serious adverse event meningitis (inflammation of the tissues lining the brain and spinal cord) occurred more frequently in trial participants given RTS,S/AS01 than in those given the control vaccine, but the incidence of other serious adverse events was similar in both groups of participants.

**What Do These Findings Mean?:**

These and other findings show that, during 18 months of follow-up, vaccination of children and young infants with RTS,S/AS01 prevented many cases of clinical and severe malaria and that the impact of vaccination was highest in regions with the highest incidence of malaria. They indicate, as in the earlier analysis, that the VE against clinical and severe malaria is higher in children than in young infants and suggest that protection wanes over time. Whether or not the vaccine played a causal role in the observed cases of meningitis cannot be determined from these results, and the occurrence of meningitis will be followed closely during the remainder of the trial. Other study limitations (for example, variations in the clinical characteristics of participants from one center to another) may also affect the accuracy of these findings and their interpretation. However, by showing that even a modest VE can avert a substantial number of malaria cases, these findings suggest that vaccination with RTS,S/AS01 could have a major public health impact in sub-Saharan Africa.

**Additional Information:**

Please access these websites via the online version of this summary at http://dx.doi.org/10.1371/journal.pmed.1001685.

- Information is available from the World Health Organization on all aspects of malaria (in several languages), including malaria immunization; the World Malaria Report 2013 provides details of the current global malaria situation; the World Health Organization also provides information on its Global Immunization Vision and Strategy (in English and French)
- The US Centers for Disease Control and Prevention provides information on malaria, including a selection of personal stories about malaria
- Information is available from the Roll Back Malaria Partnership on the global control of malaria and on the Global Malaria Action Plan (in English and French); its website includes a fact sheet about malaria in Africa
- The latest results from the phase 3 trial of RTS,S are available on the website of the PATH Malaria Vaccine Initiative, a global program of the international nonprofit organization PATH that aims to accelerate the development of malaria vaccines and ensure their availability and accessibility in the developing world
- MedlinePlus provides links to additional information on malaria and on immunization (in English and Spanish)

## Introduction

Considerable gains have been made in the control of malaria during the past decade as a result of improved diagnosis, introduction of effective treatment with artemisinin combination therapy (ACT), and widespread deployment of insecticide-treated nets (ITNs) [1],[2]. It is estimated that malaria mortality fell by 45% in all age groups and by 51% in children under 5 y of age between 2000 and 2012. However, in 2012 *Plasmodium falciparum* still caused an estimated 207 million cases of malaria and 627,000 deaths, mostly in young children in sub-Saharan Africa [3], and the gains that have been made recently in malaria control are threatened by emerging insecticide and drug resistance [4],[5]. New interventions are required if malaria is to be finally contained in the large areas of Africa where malaria transmission remains high [6]. A malaria vaccine is needed to complement current interventions.

RTS,S/AS01 is currently the most advanced malaria vaccine candidate and the first to undergo large-scale phase 3 evaluation in Africa. The RTS,S/AS01 malaria vaccine, which targets the pre-erythrocytic stage of *P. falciparum*, induces humoral and cellular immune responses to the circumsporozoite protein present on the surface of sporozoites and liver stage schizonts. RTS,S was identified as a potential candidate for further development following encouraging results in an experimental challenge study [7]. Subsequent phase 2 studies in adults and children showed that the vaccine was safe, was immunogenic, and provided protection against clinical episodes of malaria in the range of 30%–60% [8]–[10]. The AS01 adjuvant was shown to be more immunogenic than the AS02 adjuvant used in initial studies [11], and RTS,S/AS01 was well tolerated and efficacious [12]–[14]. These encouraging phase 2 trial results led to the decision to conduct a large-scale phase 3 clinical trial involving 15,460 children recruited at 11 sites in seven countries across Africa. We have reported previously overall vaccine efficacy (VE) and safety during 12 mo of follow-up [15],[16]. The first analysis, conducted in 6,000 children aged 5–17 mo at the time of first vaccination, showed that, per protocol, RTS,S/AS01 gave 56% (97.5% CI 51% to 60%) protection against the first or only episode of clinical malaria and 47% (95% CI 22% to 64%) efficacy against severe malaria during 12 mo of follow-up. VE was lower in young infants vaccinated at the age of 6–12 wk when RTS,S/AS01 was given at the same time as routine Expanded Program on Immunization (EPI) vaccines; VE against first or only clinical malaria episode in the per-protocol population was 31% (97.5% CI 24% to 38%) against clinical malaria and 37% (95% CI 5% to 58%) against severe malaria. The vaccine was immunogenic and generally safe, although an imbalance in cases of meningitis was seen in both age categories between participants who received RTS,S/AS01 and the control vaccine (rabies vaccine for children 5–17 mo at enrollment and meningococcal C conjugate vaccine for infants 6–12 wk at enrollment) [15],[16]. The objectives of the analyses reported in this paper were determination of VE in both age categories of children during 18 mo of follow-up, just prior to administration of a booster dose of RTS,S/AS01 and, for the first time, an analysis of variations in the immunogenicity and VE of RTS,S/AS01 by study site. Site-specific measurements of VE provide insight into the determinants of the overall estimates of VE and are used to calculate the number of clinical and severe malaria cases averted, important measures of public health impact that will be useful to policy makers.

## Methods

The trial protocol was approved by the ethical review board at each study center and partner institution and by the national regulatory authority in each country (Table S1A). The study was conducted in accordance with Good Clinical Practice guidelines [17].

### Study Design

**Figure 1:**
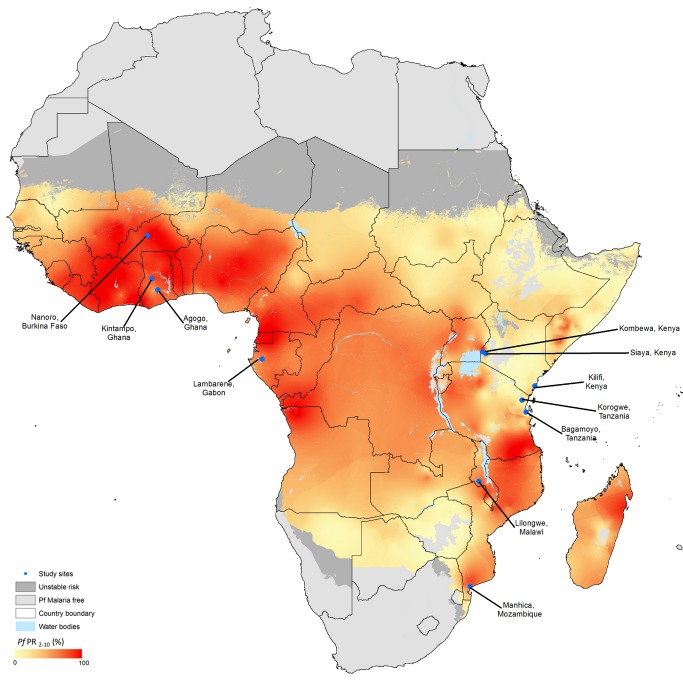
Study sites and malaria endemicity. Adapted from Hay et al. [34]. The location of each participating study site is shown on this previously published map showing the spatial distribution of *P. falciparum* (Pf) malaria endemicity. The data are the model-based geostatistical point estimates of the annual mean *P. falciparum* parasite rate (PR) age-standardized for 2–10 y for 2007 within the stable spatial limits of *P. falciparum* malaria transmission, displayed as a continuum of yellow to red from 0%–100% (see map legend). The rest of the land area was defined as unstable risk (medium grey) or no risk (light grey). Nanoro, Burkina Faso, has highly seasonal malaria transmission.

Trial methods have been reported previously [15],[16],[18] and are available in the Text S1. This randomized, controlled, double-blind trial of the candidate malaria vaccine RTS,S/AS01 is being conducted at 11 sites in seven African countries (Figure 1). The trial is designed to evaluate VE, safety, and immunogenicity during an average period of 49 mo (range: 41–55 mo) after the first dose of study vaccine in children and an average period of 41 mo (range: 32–48 mo) after the first dose of study vaccine in young infants. Informed consent was obtained from the participants' parents or guardians. Children with a moderate or severe illness; a major congenital defect; malnutrition requiring hospitalization; a hemoglobin concentration <5.0 g/dl, or <8 g/dl with clinical signs of decompensation; a history of atypical febrile seizures; a neurological disorder; or WHO stage III or stage IV HIV disease at the time of presentation were ineligible for enrollment. ITNs were provided or made available to any child who presented for screening. Reported ITN use or protection by indoor residual spraying was documented during a home visit 12 mo after the third dose of study vaccine. Anthropometric measurements were taken at enrollment and 1 and 18 mo after the third dose of study vaccine.

### Randomization and Vaccination

From March 27, 2009, through January 31, 2011, 15,460 young infants and children were assigned randomly to one of three groups in a 1∶1∶1 ratio. One group received RTS,S/AS01 followed by a booster dose 18 mo after completing the primary vaccination series, a second group received the RTS,S/AS01 primary vaccination series without a booster, and the third group received comparator vaccines, rabies vaccine (VeroRab, Sanofi-Pasteur) for children or meningococcal C conjugate vaccine (Menjugate, Novartis) for young infants. This analysis combines the first and second groups (referred to as the RTS,S/AS01 group) and compares this with the third (control) group, prior to the administration of the booster dose in the first group (1). Young infants received the study vaccine at the same time as EPI vaccines.

### Surveillance for Clinical and Severe Malaria

Malaria was detected by passive surveillance. Clinical malaria was defined as an illness accompanied by a temperature ≥37.5°C and *P. falciparum* asexual parasitemia (>5,000 parasites/mm^3^) or as an algorithm-defined case of severe malaria. Other case definitions are presented in Tables 1, 2, S2, and S5.

**Table 1:** Vaccine efficacy against all episodes of clinical and severe malaria in children aged 5–17 mo at enrollment.

Type of Event (Up to Month 20)	RTS,S/AS01 Vaccine				Control Vaccine				Protective Efficacy	
	Number of Children	Number of Events	Person-Years	Event Rate or Affected Rate (Percent)^1^	Number of Children	Number of Events	Person-Years	Event Rate or Affected Rate (Percent)^1^	Percent (95% CI)	*p*-Value^2^
**Clinical malaria—primary case definition** 3										
Per-protocol population (18 mo after third dose of vaccine)	4,557	4,257	6,186.0	0.69	2,328	3,639	3,100.4	1.17	45.7 (41.7 to 49.5)	<0.001
ITT population (20 mo after first dose of vaccine)	5,949	5,106	9,059.1	0.56	2,974	4,305	4,484.4	0.96	45.1 (41.4 to 48.7)	<0.001
**Clinical malaria—secondary case definition** 4										
Per-protocol population (18 mo after third dose of vaccine)	4,557	6,616	6,094.5	1.09	2,328	5,409	3,031.8	1.78	45.4 (41.6 to 48.9)	<0.001
ITT population (20 mo after first dose of vaccine)	5,949	8,207	8,939.1	0.92	2,974	6,482	4,400.6	1.47	43.1 (39.5 to 46.5)	<0.001
**Severe malaria without comorbidity** 5										
Per-protocol population (18 mo after third dose of vaccine)	4,557	120		2.6	2,328	95		4.1	35.5 (14.6 to 51.1)	0.001
ITT population (20 mo after first dose of vaccine)	5,949	156		2.6	2,974	118		4.0	33.9 (15.3 to 48.3)	<0.001
**Malaria hospitalization** 6										
Per-protocol population (18 mo after third dose of vaccine)	4,557	236		5.2	2,328	206		8.8	41.5 (29.1 to 51.7)	<0.001
ITT population (20 mo after first dose of vaccine)	5,949	310		5.2	2,974	261		8.8	40.6 (29.7 to 49.8)	<0.001
**All-cause hospitalization** 7										
Per-protocol population (18 mo after third dose of vaccine)	4,557	706		15.5	2,328	445		19.1	19.0 (8.5 to 28.1)	<0.001
ITT population (20 mo after first dose of vaccine)	5,949	1,003		16.9	2,974	622		20.9	19.4 (10.8 to 27.1)	<0.001

1 Event rate for clinical malaria; affected rate (percent) for severe malaria and hospitalization.

2 For clinical malaria: *p*-value from negative binomial regression. For severe malaria, malaria hospitalization, and all-cause hospitalization: *p*-value from two-sided Fisher's exact test.

3 Clinical malaria primary case definition: illness in a child brought to a study facility with a measured temperature of ≥37.5°C and *P. falciparum* asexual parasitemia at a density of >5,000 parasites/mm^3^ or a case of malaria meeting the primary case definition of severe malaria.

4 Clinical malaria secondary case definition: illness in a child brought to a study facility with a measured temperature of ≥37.5°C or reported fever within the last 24 h and *P. falciparum* asexual parasitemia at a density of >0 parasites/mm^3^.

5 Severe malaria primary case definition: *P. falciparum* asexual parasitemia at a density of >5,000 parasites/mm^3^ with one or more markers of disease severity and without diagnosis of a coexisting illness. Markers of severe disease were prostration, respiratory distress, a Blantyre coma score of ≤2 (on a scale of 0 to 5, with higher scores indicating a higher level of consciousness), two or more observed or reported seizures, hypoglycemia, acidosis, elevated lactate level, or hemoglobin level of <5 g/dl. Coexisting illnesses were defined as radiographically proven pneumonia, meningitis established by analysis of cerebrospinal fluid, bacteremia, or gastroenteritis with severe dehydration.

6 Malaria hospitalization case definition: a medical hospitalization with confirmed *P. falciparum* asexual parasitemia at a density of >5,000 parasites/mm^3^.

7 All-cause hospitalization primary case definition: a medical hospitalization of any cause, excluding planned admissions for medical investigation/care or elective surgery and admissions for trauma.

**Table 2:** Vaccine efficacy against all episodes of clinical and severe malaria in infants aged 6–12 wk at enrollment.

Type of Event (Up to Month 20)	RTS,S/AS01 Vaccine				Control Vaccine				Protective Efficacy	
	Number of Children	Number of Events	Person-Years	Event Rate or Affected Rate (Percent)^1^	Number of Children	Number of Events	Person-Years	Event Rate or Affected Rate (Percent)^1^	Percent (95% CI)	*p*-Value^2^
**Clinical malaria—primary case definition** 3										
Per-protocol population (18 mo after third dose of vaccine)	3,996	3,848	5,396.8	0.71	2,007	2,464	2,674.0	0.92	26.6 (20.3 to 32.4)	<0.001
ITT population (20 mo after first dose of vaccine)	4,358	4,252	6,583.6	0.65	2,179	2,751	3,273.6	0.84	27.0 (21.1 to 32.5)	<0.001
**Clinical malaria—secondary case definition** 4										
Per-protocol population (18 mo after third dose of vaccine)	3,996	5,781	5,321.4	1.09	2,007	3,718	2,624.6	1.42	27.8 (22.0 to 33.1)	<0.001
ITT population (20 mo after first dose of vaccine)	4,358	6,564	6,494.6	1.01	2,179	4,221	3,216.4	1.31	27.7 (22.3 to 32.7)	<0.001
**Severe malaria without comorbidity** 5										
Per-protocol population (18 mo after third dose of vaccine)	3,996	100		2.5	2,007	59		2.9	14.9 (−19.5 to 38.9)	0.348
ITT population (20 mo after first dose of vaccine)	4,358	121		2.8	2,179	66		3.0	8.3 (−25.7 to 32.6)	0.581
**Malaria hospitalization** 6										
Per-protocol population (18 mo after third dose of vaccine)	3,996	165		4.1	2,007	100		5.0	17.1 (−7.3 to 35.7)	0.142
ITT population (20 mo after first dose of vaccine)	4,358	200		4.6	2,179	115		5.3	13.0 (−10.4 to 31.2)	0.221
**All-cause hospitalization** 7										
Per-protocol population (18 mo after third dose of vaccine)	3,996	716		17.9	2,007	383		19.1	6.1 (−6.6 to 17.2)	0.273
ITT population (20 mo after first dose of vaccine)	4,358	871		20.0	2,179	458		21.0	4.9 (−6.7 to 15.2)	0.328

1 Event rate for clinical malaria; affected rate (percent) for severe malaria and hospitalization.

2 For clinical malaria: *p*-value from negative binomial regression. For severe malaria, malaria hospitalization, and all-cause hospitalization: *p*-value from two-sided Fisher's exact test.

3 Clinical malaria primary case definition: illness in a child brought to a study facility with a measured temperature of ≥37.5°C and *P. falciparum* asexual parasitemia at a density of >5,000 parasites/mm^3^ or a case of malaria meeting the primary case definition of severe malaria.

4 Clinical malaria secondary case definition: illness in a child brought to a study facility with a measured temperature of ≥37.5°C or reported fever within the last 24 h and *P. falciparum* asexual parasitemia at a density of >0 parasites/mm^3^.

5 Severe malaria primary case definition: *P. falciparum* asexual parasitemia at a density of >5,000 parasites/mm^3^ with one or more markers of disease severity and without diagnosis of a coexisting illness. Markers of severe disease were prostration, respiratory distress, a Blantyre coma score of ≤2 (on a scale of 0 to 5, with higher scores indicating a higher level of consciousness), two or more observed or reported seizures, hypoglycemia, acidosis, elevated lactate level, or hemoglobin level of <5 g/dl. Coexisting illnesses were defined as radiographically proven pneumonia, meningitis established by analysis of cerebrospinal fluid, bacteremia, or gastroenteritis with severe dehydration.

6 Malaria hospitalization case definition: a medical hospitalization with confirmed *P. falciparum* asexual parasitemia at a density of >5,000 parasites/mm^3^.

7 All-cause hospitalization primary case definition: a medical hospitalization of any cause, excluding planned admissions for medical investigation/care or elective surgery and admissions for trauma.

### Safety Surveillance

Data on serious adverse events (SAEs) were collected by passive surveillance. SAE classification was made using all available clinical evidence, and was not bound by stringent laboratory or diagnostic criteria. Verbal autopsies were conducted on deaths that occurred outside a hospital. SAEs were coded from clinician-assigned diagnoses according to the preferred terms of the Medical Dictionary for Regulatory Activities (MedDRA) [19].

### Immunogenicity

Anti-circumsporozoite (anti-CS) antibodies were measured by ELISA in the first 200 participants in each age category at each study site at enrollment and 1 mo after the third dose of vaccine. The threshold for a positive titer was 0.5 EU/ml [20].

### Laboratory and Radiologic Procedures

Laboratory and radiologic procedures are described fully in Text S1. All blood smears were read by two independent microscopists, and parasite densities were determined using standardized procedures. Discrepant findings were resolved according to a formal algorithm. Standardized, automated biochemical and hematological methods were used. Standard microbiology methods for blood and CSF culture were followed using automated Bactec incubators and pediatric bottles (Bactec BD Diagnostic Systems). A rigorous external quality assurance process was implemented at all sites for all laboratory procedures that produced study end points.

### Statistical Analysis

Efficacy against all episodes of malaria was analyzed by negative binomial regression with follow-up time as offset, allowing for interdependence between episodes within the same individual. Overall estimates were adjusted for study site as a fixed effect, whereas site estimates were unadjusted for covariates. Inter-site variation was evaluated by site interaction terms, and prespecified univariate analyses and multivariate models were used to explore the impact of covariates on post-vaccination anti-CS responses or on VE. Transmission intensity was defined in the models as the malaria incidence in control children or control young infants, depending on the age category included in the model. VE against all other clinical end points was estimated as a relative risk (RR) reduction with Fisher's exact *p-*values. VE over time was evaluated by Schoenfeld residuals *p-*values, Andersen-Gill Cox models with time-varying covariates, and incidence reduction by 6-mo periods. Vaccine impact was calculated as the difference in incidence between the RTS,S/AS01 and control groups, expressed per 1,000 children vaccinated for each 6-mo period of follow-up. The cumulative number of cases averted over 18 mo was calculated by summing the number of cases averted for each 6-mo follow-up period. The per-protocol population included all participants who received three doses of vaccine and contributed to efficacy surveillance, starting 14 d after the third dose. The intention-to-treat (ITT) population included all participants who received at least one dose of vaccine. The more specific primary case definition of clinical malaria was used for determination of VE. VE calculated using secondary case definitions is also reported. The co-primary end points of VE in infants and children, presented in prior publications, were described with 97.5% CIs; all other VE end points are presented with 95% CIs. A sensitive secondary case definition (measured or reported fever in the prior 24 h and *P. falciparum* parasite density >0 parasites/mm^3^) was used for the evaluation of the impact of RTS,S/AS01 on clinical malaria because, in clinical practice, sick children who present to a health facility with any level of malaria parasitemia receive treatment for malaria. Data were censored at the time of administration of a booster dose, 20 mo after dose 1, or at the date of emigration, withdrawal of consent, or death.

## Results

### Study Population

**Figure 2:**
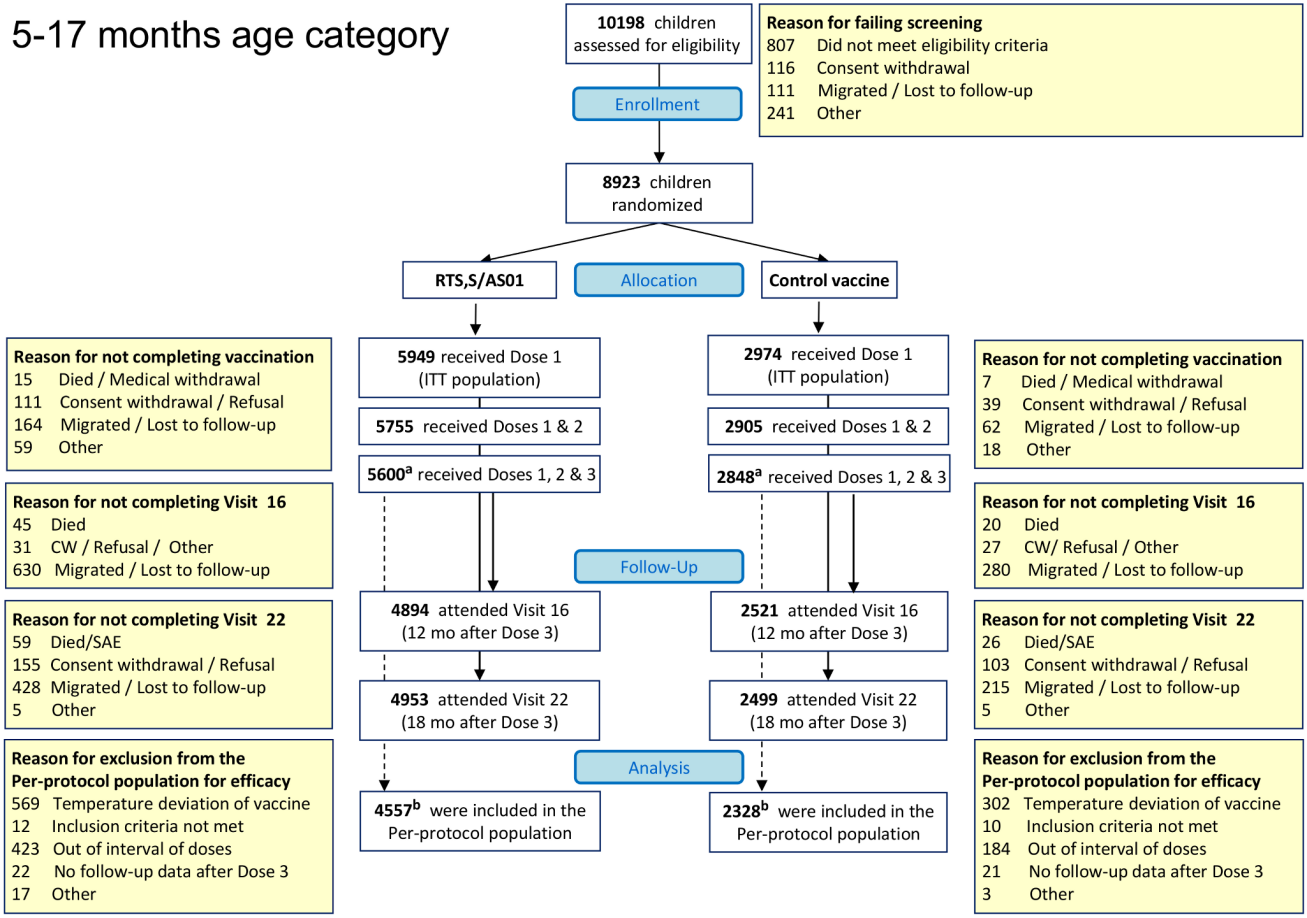
CONSORT diagram of children aged 5–17 mo at enrollment and followed until 18 mo post-vaccination. ^a^One child enrolled in the 5–17-mo age category who was reported previously to have received three doses of study vaccine, and was included in the per-protocol analyses reported previously, had received only the first and second doses of study vaccine. ^b^The date of birth of three children who were included in the per-protocol analysis reported previously was corrected, and these children were identified as “out of age range” when they received the first dose of study vaccine. These three children were excluded from the per-protocol analyses reported here. CW, consent withdrawal.

**Figure 3:**
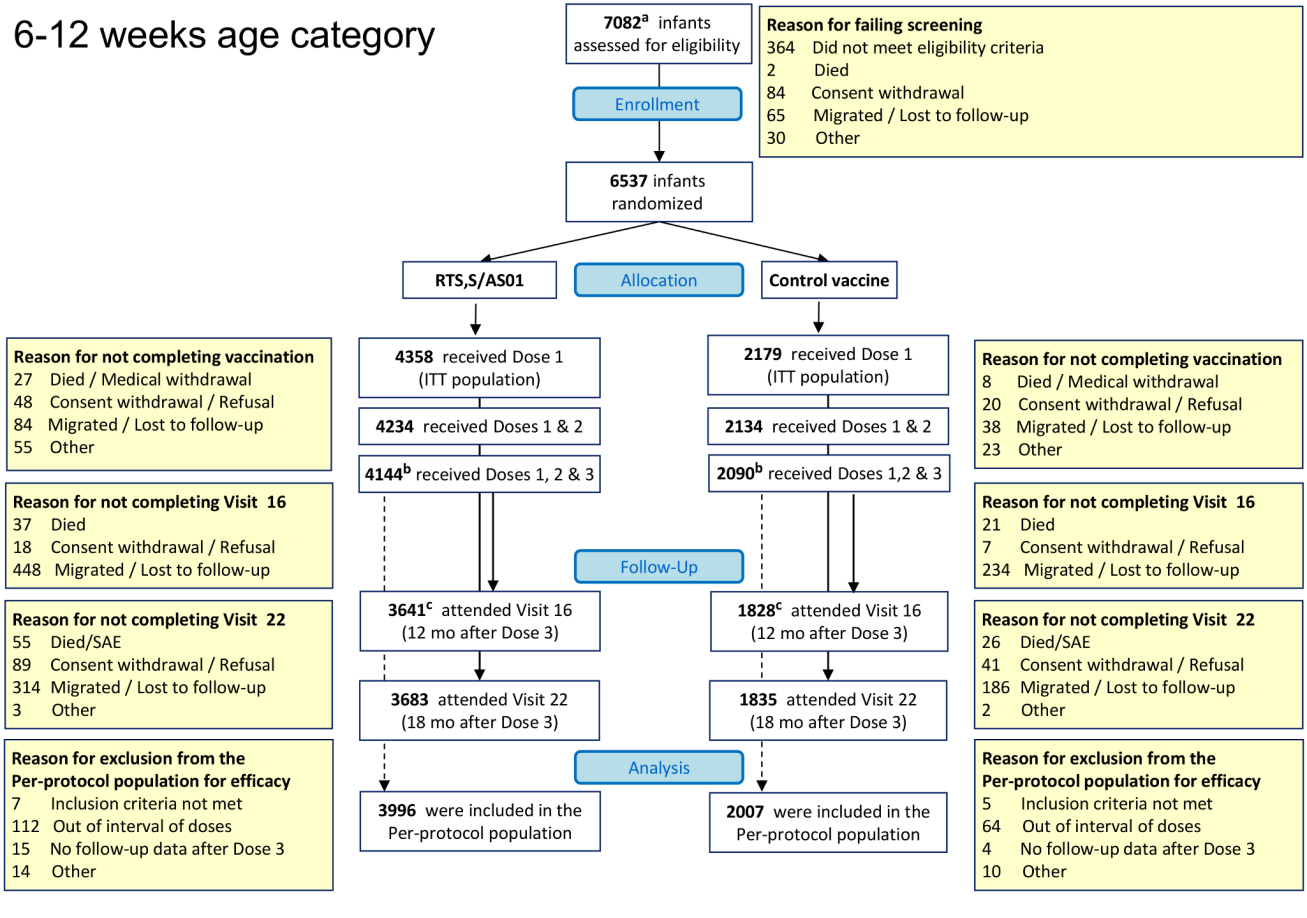
CONSORT diagram of infants aged 6–12 wk at enrollment followed until 18 mo post-vaccination. ^a^Screening data had not been reported before the database freeze for the analysis published in 2011 for 22 infants in the 6–12-wk age category, and these participants were not included in the 2011 CONSORT chart. ^b^One infant enrolled in the 6–12-wk age category who was reported to have received three doses of study vaccine (RTS,S/AS01 or comparator vaccine) was included in the per-protocol analyses reported previously, but was subsequently found to have received only the first dose of study vaccine. ^c^Two infants enrolled in the 6–12-wk age category who had been reported as “attending Visit 16 (12 months post dose-3)” in the 2012 CONSORT chart are reported as “migrated/lost to follow-up” in this CONSORT chart. In addition, one infant who was reported as “migrated/lost to follow-up” in the 2012 CONSORT chart has been recorded as “consent withdrawal” in this CONSORT chart [15].

Overall, 8,923 children and 6,537 young infants were enrolled. All randomized children and young infants were included in the ITT population, while 6,885 (77%) children and 6,003 (92%) young infants were included in the per-protocol population (Figures 2 and 3; Table S3). Baseline characteristics were similar in the two study groups but differed by site. ITN use was 78% in children and 86% in young infants (Figure S2, ITT). Malaria incidence in young infants in the control group, an approximation of the force of infection, ranged across sites from 0.03 to 4.27 episodes per infant per year (Table S4, per protocol).

### Vaccine Efficacy in Children

**Figure 4:**
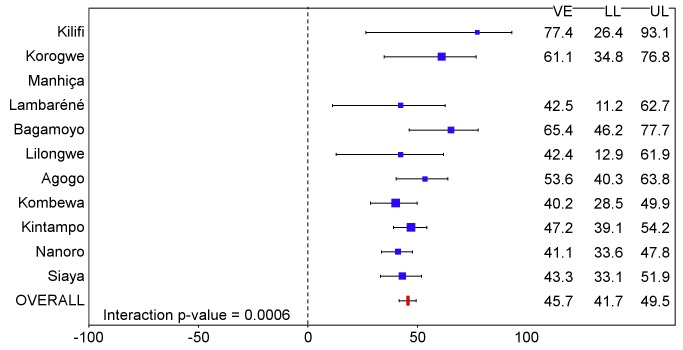
Vaccine efficacy against all episodes of clinical malaria (primary case definition) during an 18-mo follow-up period after dose 3 in children 5–17 mo of age at enrollment, ordered by increasing malaria incidence at each study site (per-protocol population). Interaction *p-*value = 0.0006. The size of each blue square reflects the relative number of participants enrolled at each study site; the horizontal bars show the lower limit (LL) and upper limit (UL) of the 95% confidence interval. Study sites are ordered from lowest (Kilifi) to highest (Siaya) incidence of clinical malaria, defined as a measured or reported fever within previous 24 h and parasite density >0 parasites/mm^3^ (i.e., clinical malaria secondary case definition), measured in control infants 6–12 wk of age at enrollment during 12 mo of follow-up. VE is VE against all episodes of clinical malaria meeting the primary case definition, unadjusted for covariates. Clinical malaria primary case definition: illness in a child brought to a study facility with a temperature of ≥37.5°C and *P. falciparum* asexual parasitemia at a density of >5,000 parasites/mm^3^ or a case of malaria meeting the primary case definition of severe malaria.

**Figure 5:**
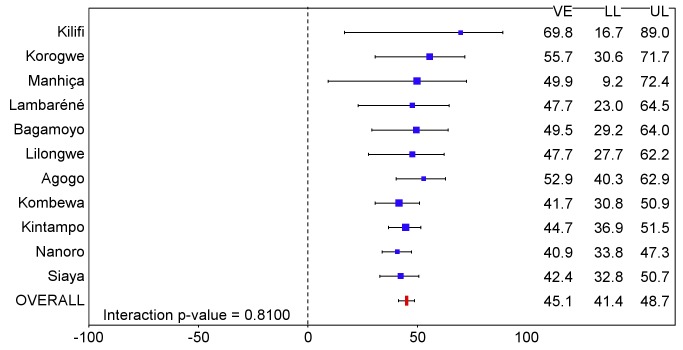
Vaccine efficacy against all episodes of clinical malaria (primary case definition) during an 18-mo follow-up period after dose 3 in children 5–17 mo of age at enrollment, ordered by increasing malaria incidence at each study site (intention-to-treat population). Interaction *p-*value = 0.8100. The size of each blue square reflects the relative number of participants enrolled at each study site; the horizontal bars show the lower limit (LL) and upper limit (UL) of the 95% confidence interval. Study sites are ordered from lowest (Kilifi) to highest (Siaya) incidence of clinical malaria, defined as a measured or reported fever within previous 24 h and parasite density >0 parasites/mm^3^ (i.e., clinical malaria secondary case definition), measured in control infants 6–12 wk of age at enrollment during 12 mo of follow-up. VE is VE against all episodes of clinical malaria meeting the primary case definition, unadjusted for covariates. Clinical malaria primary case definition: illness in a child brought to a study facility with a temperature of ≥37.5°C and *P. falciparum* asexual parasitemia at a density of >5,000 parasites/mm^3^ or a case of malaria meeting the primary case definition of severe malaria.

The incidence of all episodes of clinical malaria meeting the primary case definition during the 18 mo of follow-up in the per-protocol population was 0.69/person-year in the RTS,S/AS01 group and 1.17/person-year in the control group, resulting in a VE of 46% (95% CI 42% to 50%), with overall estimates of VE consistent across case definitions and with the ITT analysis (ITT, VE = 45% [95% CI 41% to 49%]) (Tables 1 and S5; Figures 4, 5, and S3). Significant heterogeneity in VE was seen across sites, with VE ranging from 40% to 77% (interaction test, *p*<0.001; VE, *p<*0.01 at all sites). VE in the ITT population ranged across sites from 41% to 70%. Analysis of site-specific VE in the per-protocol population did not suggest that this heterogeneity was driven by variations in transmission intensity (interaction between study group and transmission intensity, *p* = 0.66). Lower VE was associated with moderate anemia at baseline (*p = *0.04) (Table S6). VE varied over time, being highest close to vaccination (Schoenfeld residuals *p<*0.001) (Figure S4), but it persisted throughout the observation period. The reduction in the incidence of clinical malaria by 6-mo periods was 68% (95% CI 64% to 72%) during months 1–6, 41% (95% CI 36% to 46%) during months 7–12, and 26% (95% CI 19% to 33%) during months 13–18 after vaccine dose 3. Corresponding values in the ITT population were 60% (95% CI 56% to 64%), 41% (95% CI 36% to 46%), and 28% (95% CI 21% to 35%) (Figures S5 and S6). VE against clinical malaria and severe malaria during the first 12 mo of follow-up is presented in Figure S8 and Table S15. VE against prevalent parasitemia, 18 mo after vaccination, was 31% (95% CI 17% to 42%) (ITT, VE = 29% [95% CI 15% to 40%]) (Table S7). VE was 36% (95% CI 15% to 51%) against severe malaria (ITT, VE against severe malaria = 34% [95% CI 15% to 48%]), 42% (95% CI 29% to 52%) against malaria hospitalization (ITT, VE against malaria hospitalization = 41% [95% CI 30% to 50%]), and 19% (95% CI 9% to 28%) against all-cause hospitalization (ITT, VE against all-cause hospitalization = 19% [95% CI 11% to 27%]) (Table 1). The incidence of severe malaria by 6-mo periods is shown in Figure S5.

**Figure 6:**
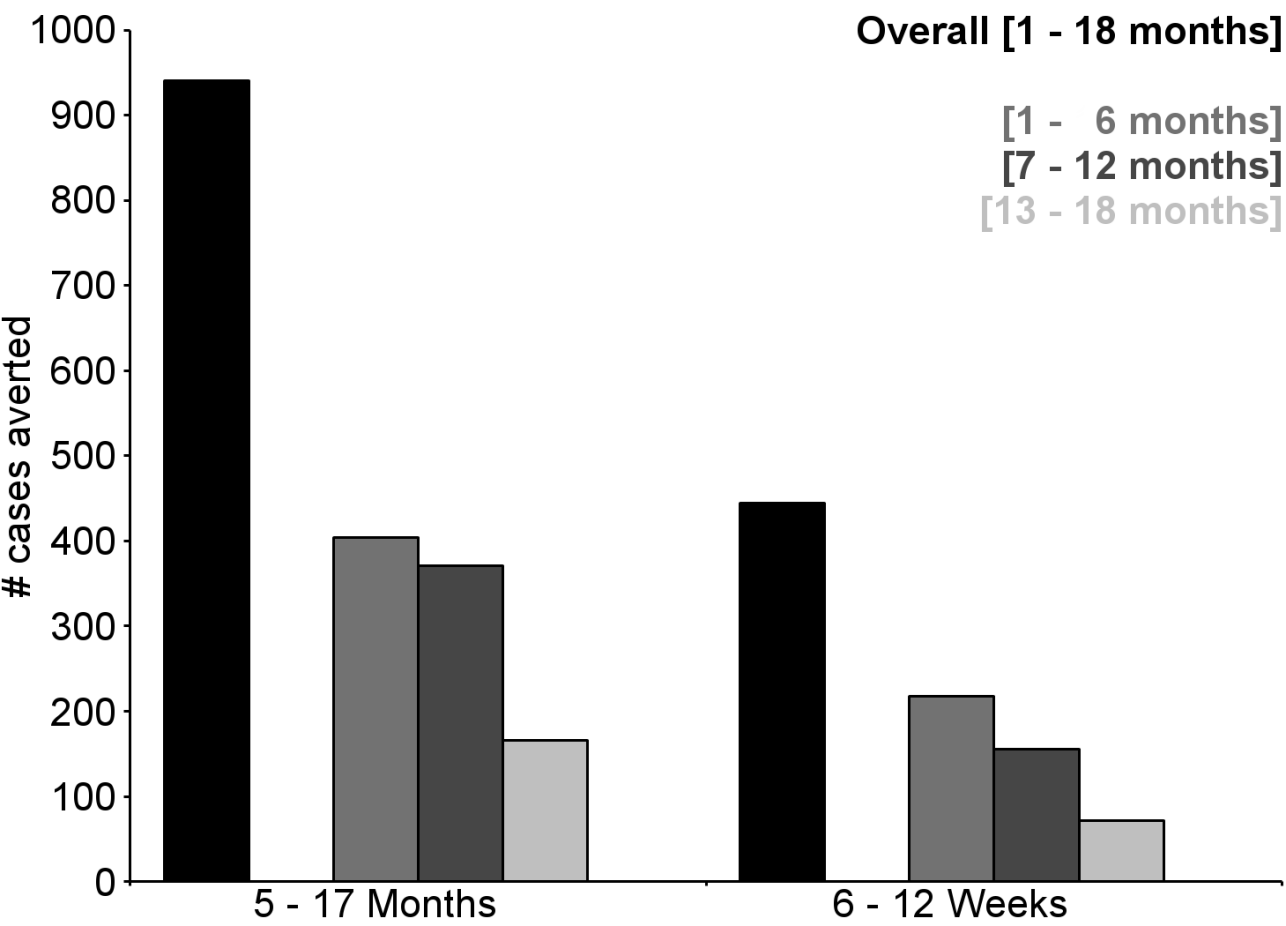
Number of cases of clinical malaria (secondary case definition) averted per 1,000 participants vaccinated during an 18-mo follow-up period (per-protocol population). Clinical malaria secondary case definition: illness in a child brought to a study facility with a measured temperature of ≥37.5°C or reported fever within the last 24 h and *P. falciparum* asexual parasitemia at a density of >0 parasites/mm^3^. This definition was used for this analysis because during routine clinical practice these children would normally receive a full course of anti-malarial treatment.

**Figure 7:**
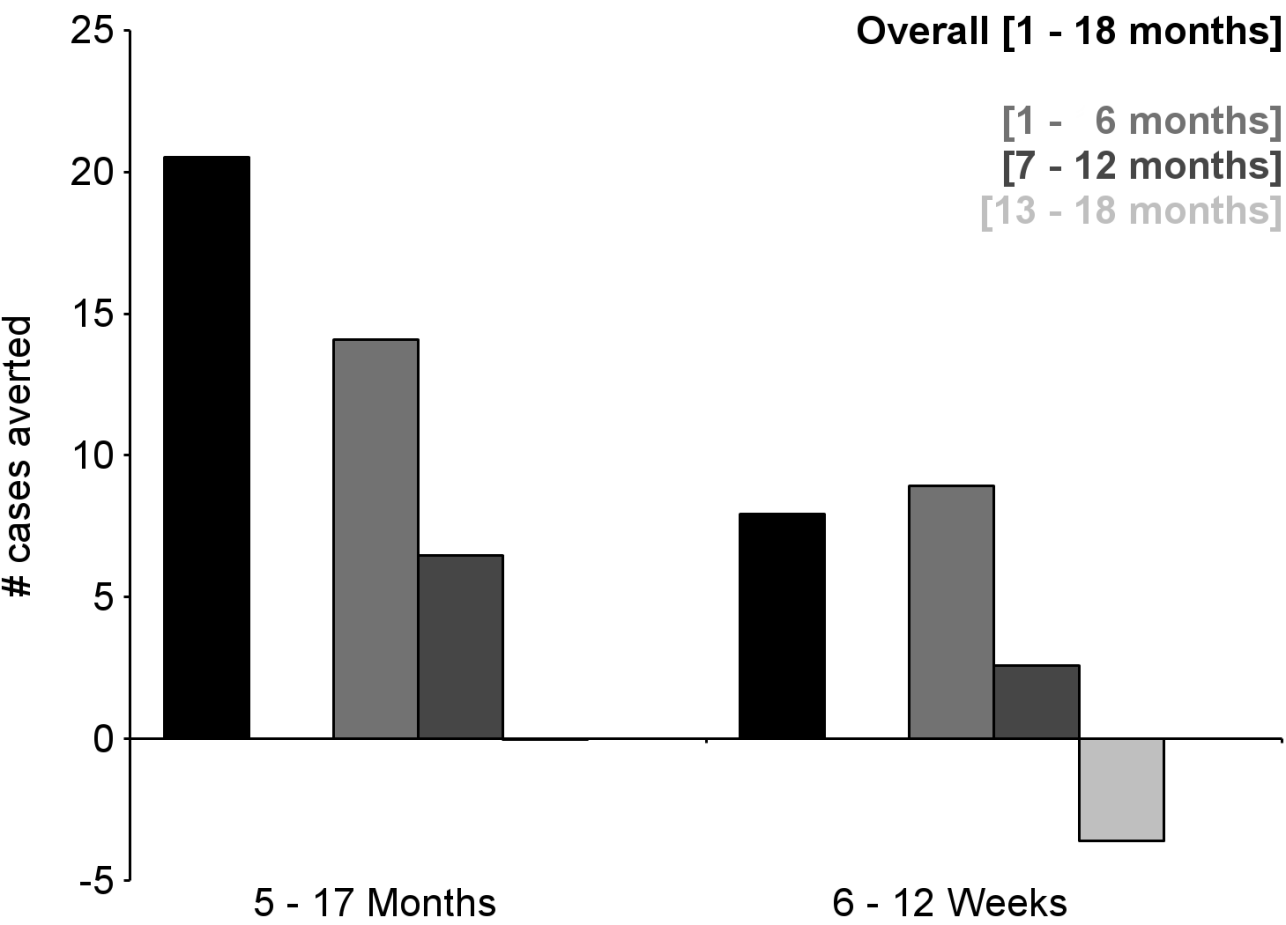
Number of cases of severe malaria (secondary case definition) averted per 1,000 participants vaccinated during an 18-mo follow-up period (per-protocol population). Severe malaria secondary case definition: *P. falciparum* asexual parasitemia at a density of >5,000 parasites/mm^3^ with one or more markers of disease severity, including cases in which a coexisting illness was present or could not be ruled out. Markers of severe disease were prostration, respiratory distress, a Blantyre coma score of ≤2 (on a scale of 0 to 5, with higher scores indicating a higher level of consciousness), two or more observed or reported seizures, hypoglycemia, acidosis, elevated lactate level, or a hemoglobin level of <5 g/dl. Coexisting illnesses were defined as radiographically proven pneumonia, meningitis established by analysis of cerebrospinal fluid, bacteremia, or gastroenteritis with severe dehydration.

**Figure 8:**
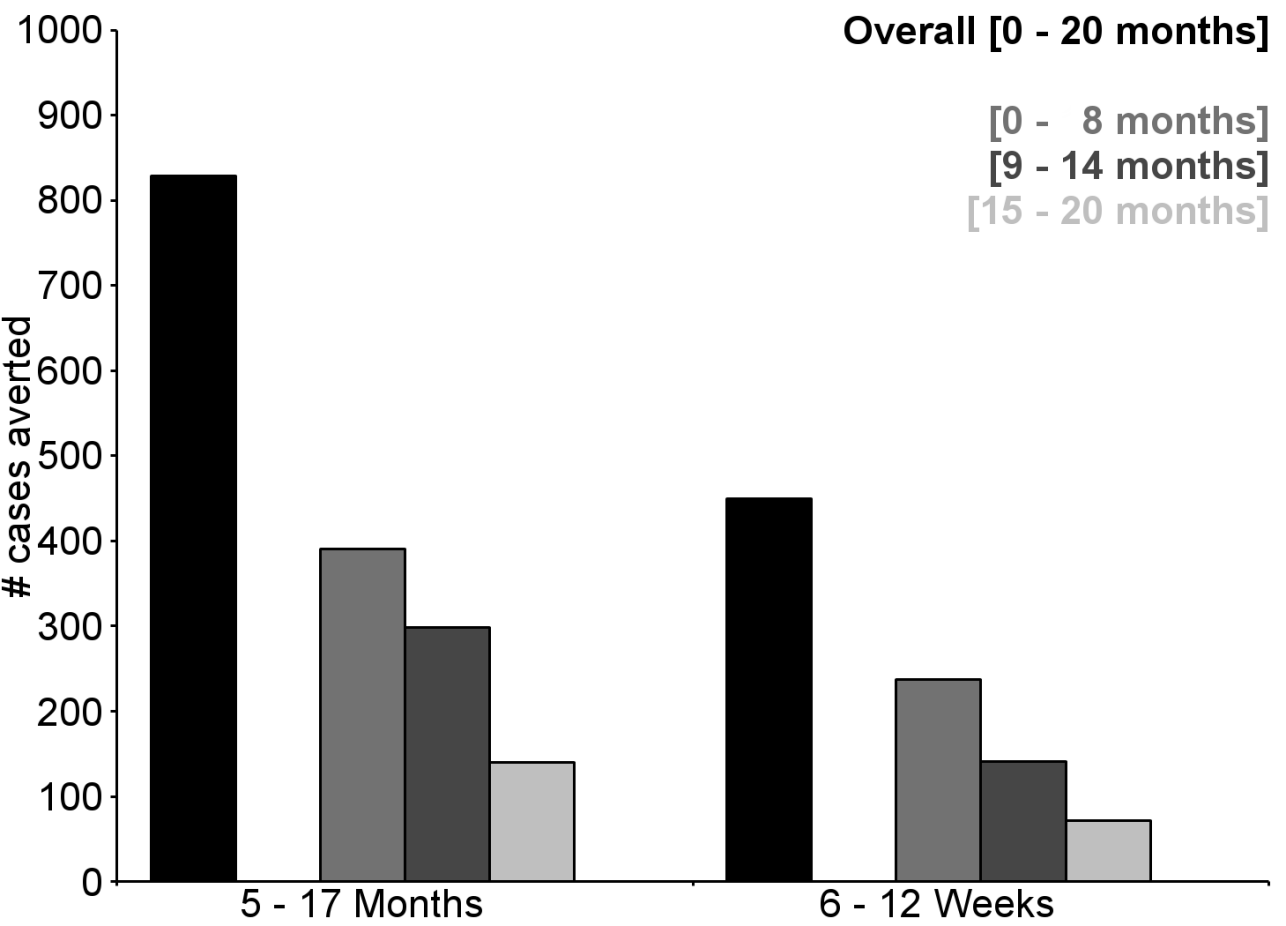
Number of cases of clinical malaria (secondary case definition) averted per 1,000 participants vaccinated during a 20-mo follow-up period (intention-to-treat population). Clinical malaria secondary case definition: illness in a child brought to a study facility with a measured temperature of ≥37.5°C or reported fever within the last 24 h and *P. falciparum* asexual parasitemia at a density of >0 parasites/mm^3^. This definition was used for this analysis because during routine clinical practice these children would normally receive a full course of anti-malarial treatment.

**Figure 9:**
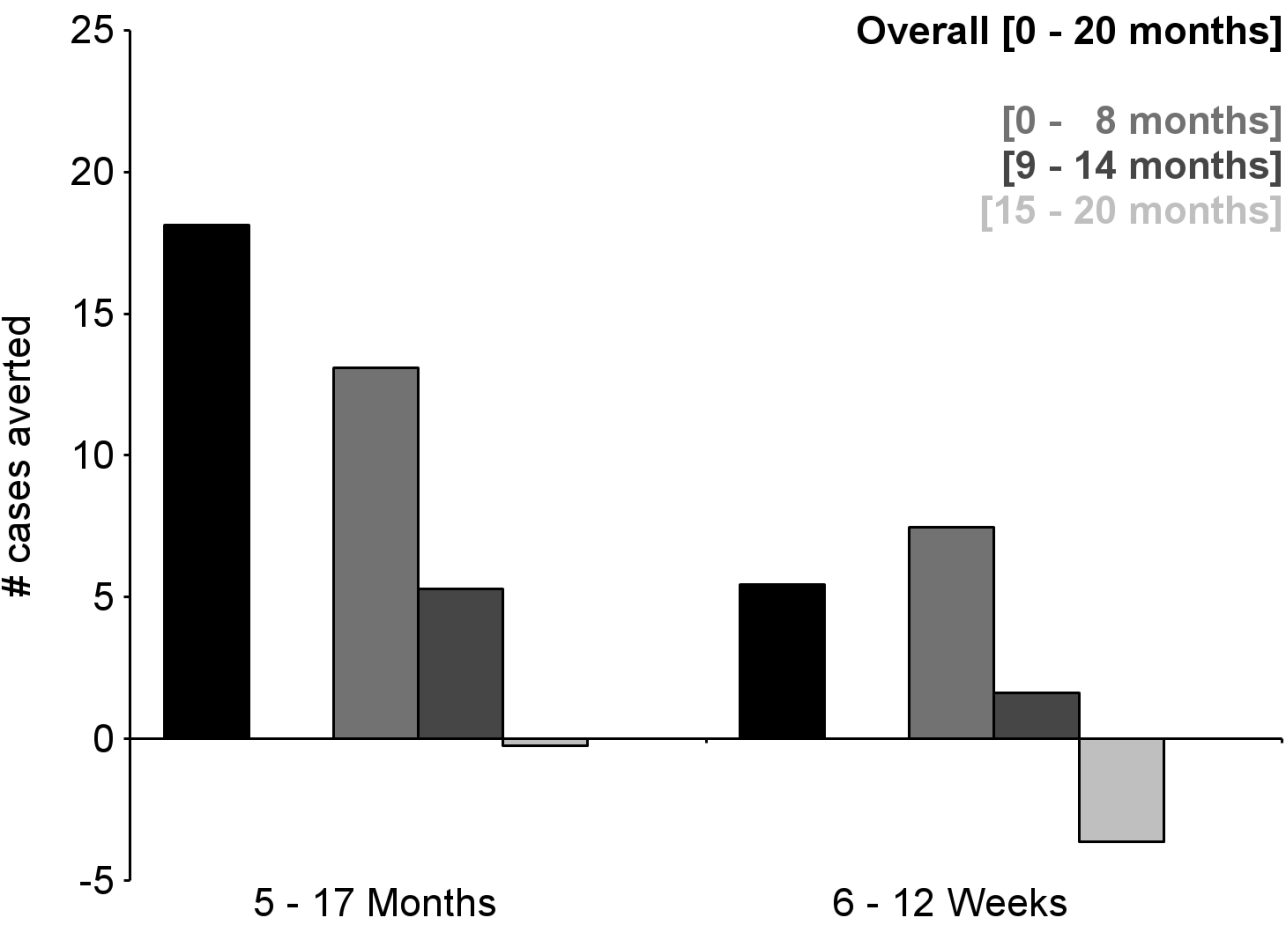
Number of cases of severe malaria (secondary case definition) averted per 1,000 participants vaccinated during a 20-mo follow-up period (intention-to-treat population). Severe malaria secondary case definition: *P. falciparum* asexual parasitemia at a density of >5,000 parasites/mm^3^ with one or more markers of disease severity, including cases in which a coexisting illness was present or could not be ruled out. Markers of severe disease were prostration, respiratory distress, a Blantyre coma score of ≤2 (on a scale of 0 to 5, with higher scores indicating a higher level of consciousness), two or more observed or reported seizures, hypoglycemia, acidosis, elevated lactate level, or a hemoglobin level of <5 g/dl. Coexisting illnesses were defined as radiographically proven pneumonia, meningitis established by analysis of cerebrospinal fluid, bacteremia, or gastroenteritis with severe dehydration.

**Figure 10:**
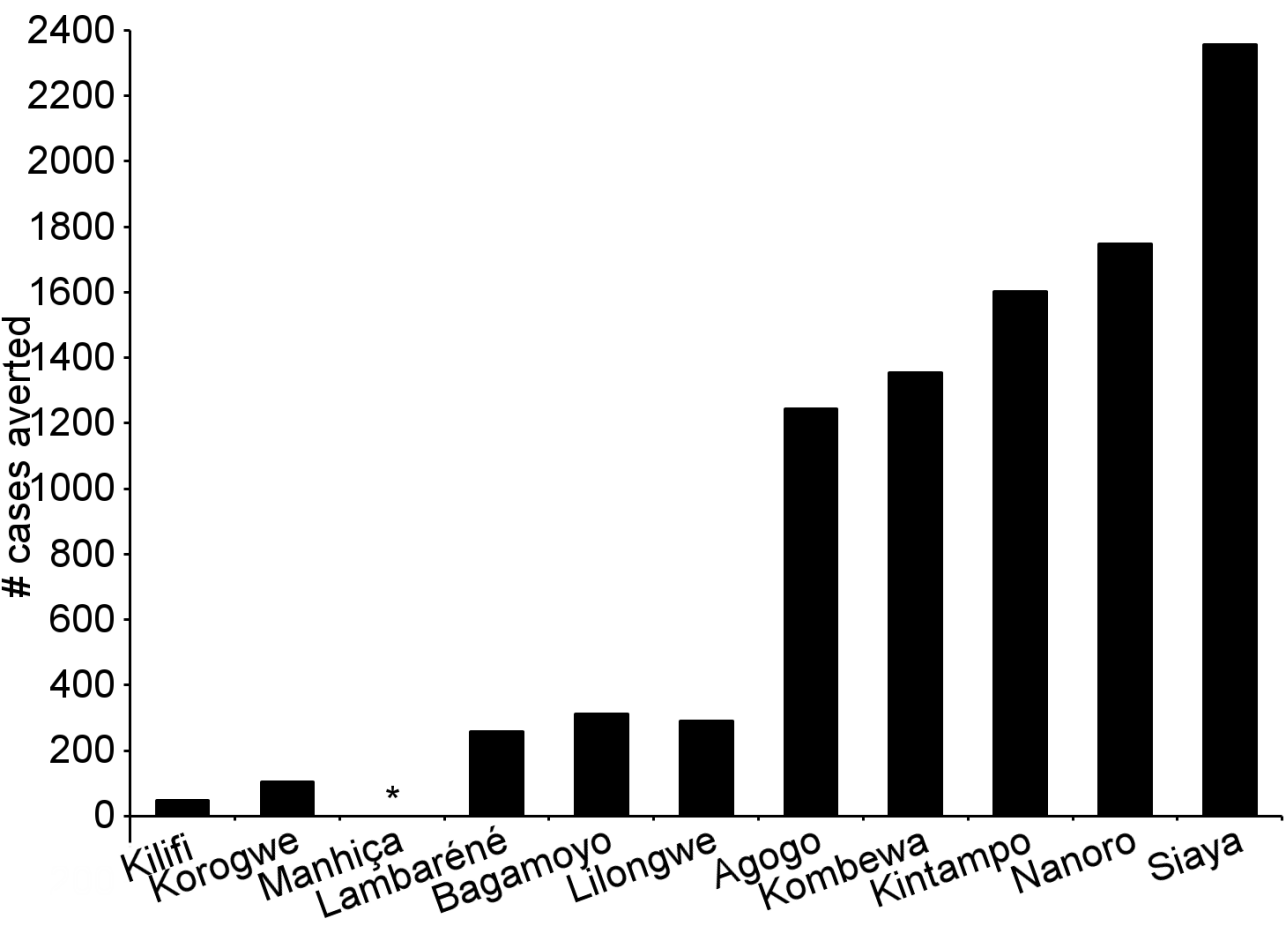
Number of cases of clinical malaria (secondary case definition) averted during an 18-mo follow-up period in children 5–17 mo of age at enrollment, by study site, ordered by increasing malaria incidence at each site (per-protocol population). Clinical malaria secondary case definition: illness in a child brought to a study facility with a measured temperature of ≥37.5°C or reported fever within the last 24 h and *P. falciparum* asexual parasitemia at a density of >0 parasites/mm^3^. This definition was used for this analysis because during routine clinical practice these children would normally receive a full course of anti-malarial treatment. Study sites are ordered from lowest (Kilifi) to highest (Siaya) incidence of clinical malaria, defined as a measured or reported fever within previous 24 h and parasite density >0 parasites/mm^3^ (i.e., clinical malaria secondary case definition), measured in control infants 6–12 wk of age at enrollment during 12 mo of follow-up. A deviation pertaining to study vaccine exposure to temperatures outside recommended ranges resulted in the exclusion from the per-protocol population of all children 5–17 mo old enrolled in Manhiça, Mozambique, therefore no data are presented in this age category for Manhiça (asterisk).

**Figure 11:**
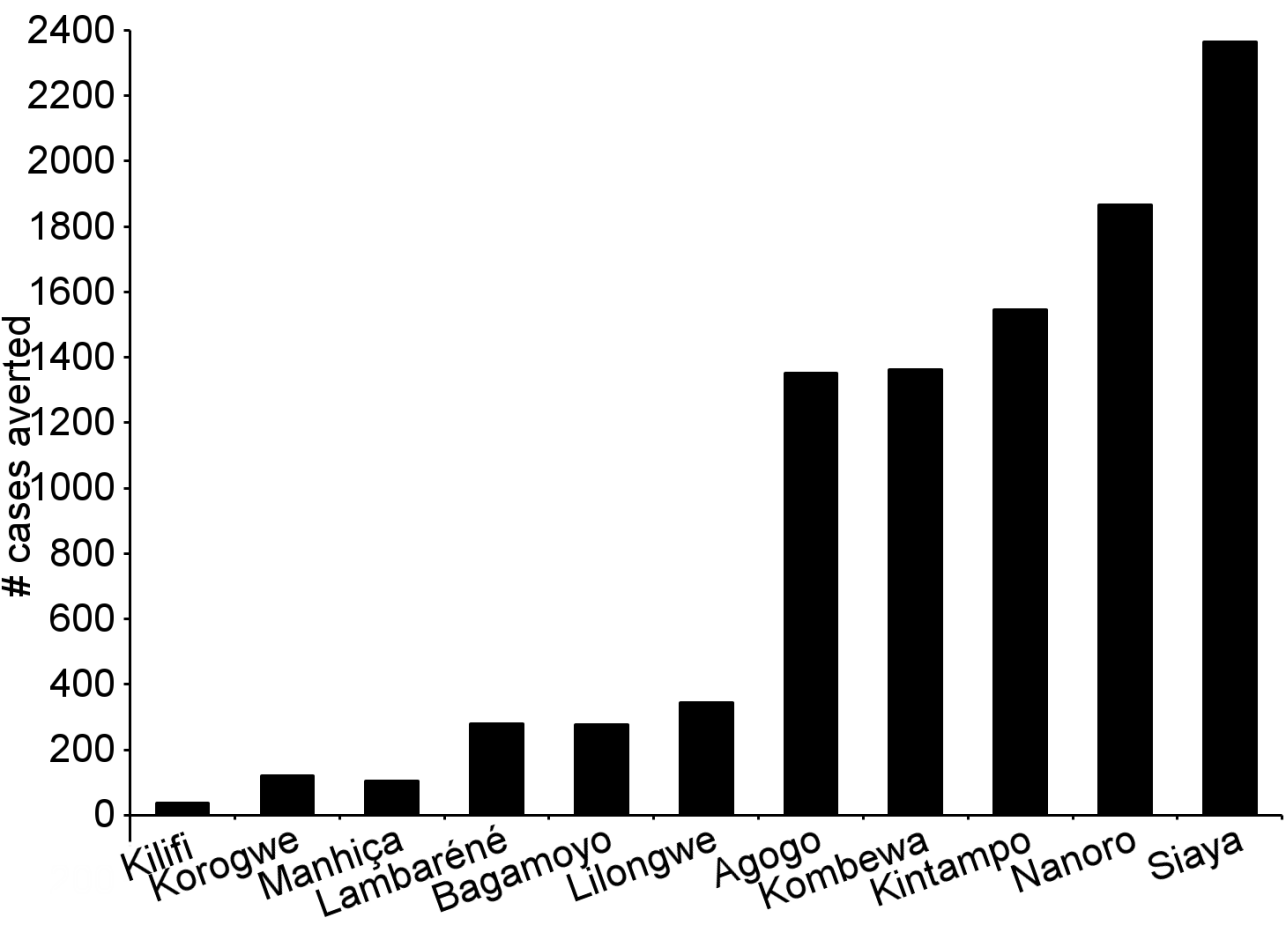
Cases of clinical malaria (secondary case definition) averted during an 18-mo follow-up period in children 5–17 mo of age at enrollment, by study site, ordered by increasing malaria incidence at each site (intention-to-treat population). Clinical malaria secondary case definition: illness in a child brought to a study facility with a measured temperature of ≥37.5°C or reported fever within the last 24 h and *P. falciparum* asexual parasitemia at a density of >0 parasites/mm^3^. This definition was used for this analysis because during routine clinical practice these children would normally receive a full course of anti-malarial treatment. Study sites are ordered from lowest (Kilifi) to highest (Siaya) incidence of clinical malaria, defined as a measured or reported fever within previous 24 h and parasite density >0 parasites/mm^3^ (i.e., clinical malaria secondary case definition), measured in control infants 6–12 wk of age at enrollment during 12 mo of follow-up.

During the 18-mo follow-up period, the number of cases of clinical malaria averted per 1,000 children vaccinated with RTS,S/AS01 in the ITT population ranged across sites from 37 to 2,365 (average for the 11 study sites: 829), and the number of severe malaria cases averted ranged from −1 to 49 (average across all sites: 18) (Figures 6–11; Table S8). The number of malaria hospitalizations averted per 1,000 children vaccinated ranged from 0 to 138 across sites (average across all sites: 43), and all-cause hospitalizations averted ranged from −3 to 132 (average across all sites: 52) during the same period (Table S8). Mortality was low; 107 children (1.2%) died during the observation period, 74/5,949 (1.2%) children in the RTS,S/AS01 group and 33/2,974 (1.1%) in the control group. VE was not demonstrated against all-cause mortality, malaria mortality, hospitalized pneumonia, septicemia, or prevalent anemia, nor was there a detectable effect on childhood nutritional status or growth (Tables S7, S9, and S10; Figure S7).

### Vaccine Efficacy in Young Infants

**Figure 12:**
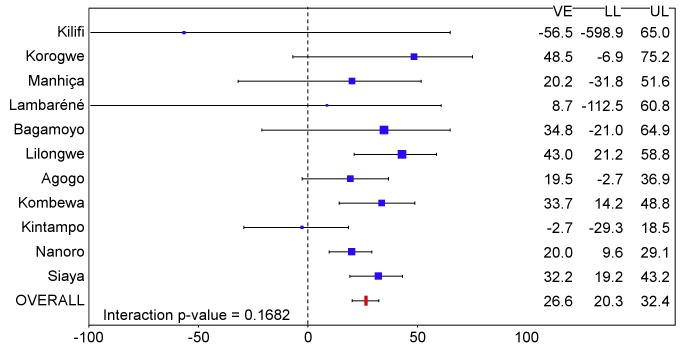
Vaccine efficacy against all episodes of clinical malaria (primary case definition) during an 18-mo follow-up period after dose 3 in infants 6–12 wk of age at enrollment, ordered by increasing malaria incidence at each study site (per-protocol population). Interaction *p-*value = 0.1682. The size of each blue square reflects the relative number of participants enrolled at each study site; the horizontal bars show the lower limit (LL) and upper limit (UL) of the 95% confidence interval. Study sites are ordered from lowest (Kilifi) to highest (Siaya) incidence of clinical malaria, defined as a measured or reported fever within previous 24 h and parasite density >0 parasites/mm^3^ (i.e., clinical malaria secondary case definition), measured in control infants 6–12 wk of age at enrollment during 12 mo of follow-up. VE is VE against all episodes of clinical malaria meeting the primary case definition, unadjusted for covariates. Clinical malaria primary case definition: illness in a child brought to a study facility with a temperature of ≥37.5°C and *P. falciparum* asexual parasitemia at a density of >5,000 parasites/mm^3^ or a case of malaria meeting the primary case definition of severe malaria.

**Figure 13:**
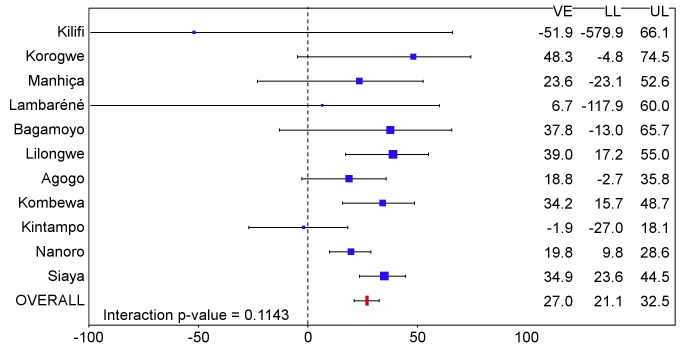
Vaccine efficacy against all episodes of clinical malaria (primary case definition) during an 18-mo follow-up period after dose 3 in infants 6–12 wk of age at enrollment, ordered by increasing malaria incidence at each study site (intention-to-treat population). Interaction *p-*value = 0.1143. The size of each blue square reflects the relative number of participants enrolled at each study site; the horizontal bars show the lower limit (LL) and upper limit (UL) of the 95% confidence interval. Study sites are ordered from lowest (Kilifi) to highest (Siaya) incidence of clinical malaria, defined as a measured or reported fever within previous 24 h and parasite density >0 parasites/mm^3^ (i.e., clinical malaria secondary case definition), measured in control infants 6–12 wk of age at enrollment during 12 mo of follow-up. VE is VE against all episodes of clinical malaria meeting the primary case definition, unadjusted for covariates. Clinical malaria primary case definition: illness in a child brought to a study facility with a temperature of ≥37.5°C and *P. falciparum* asexual parasitemia at a density of >5,000 parasites/mm^3^ or a case of malaria meeting the primary case definition of severe malaria.

The incidence of all episodes of clinical malaria meeting the primary case definition during the 18 mo of follow-up in the per-protocol population was 0.71/person-year in the RTS,S/AS01 group and 0.92/person-year in the control group, resulting in a VE of 27% (95% CI 20% to 32%), with overall estimates of VE consistent across case definitions and the ITT analysis (ITT, VE = 27% [95% CI 21% to 33%]) (Tables 2 and S5; Figures 12, 13, and S3). VE did not differ by study site (interaction between study group and site, *p = *0.17). VE waned over time (Schoenfeld residuals *p<*0.001). The reduction in incidence of clinical malaria by 6-mo period was 47% (95% CI 39% to 54%) during months 1–6, 23% (95% CI 15% to 31%) during months 7–12, and 12% (95% CI 1% to 21%) during months 13–18 following dose 3. Corresponding values in the ITT population were 44% (95% CI 37% to 50%), 23% (95% CI 15% to 31%), and 13% (95% CI 2% to 22%) (Figures S5 and S6). VE was 15% (95% CI −20% to 39%) against severe malaria (ITT, VE against severe malaria = 8% [95% CI −26% to 33%]), 17% (95% CI −7% to 36%) against malaria hospitalization (ITT, VE against malaria hospitalization = 13% [95% CI −10% to 31%]), and 6% (95% CI −7% to 17%) against all-cause hospitalization (ITT, VE against all-cause hospitalization = 5% [95% CI −7% to 15%]). A statistically non-significant increase in the incidence of severe malaria was observed in RTS,S/AS01 recipients in the last 6 mo of follow-up (*p* = 0.17) (Figure S5).

**Figure 14:**
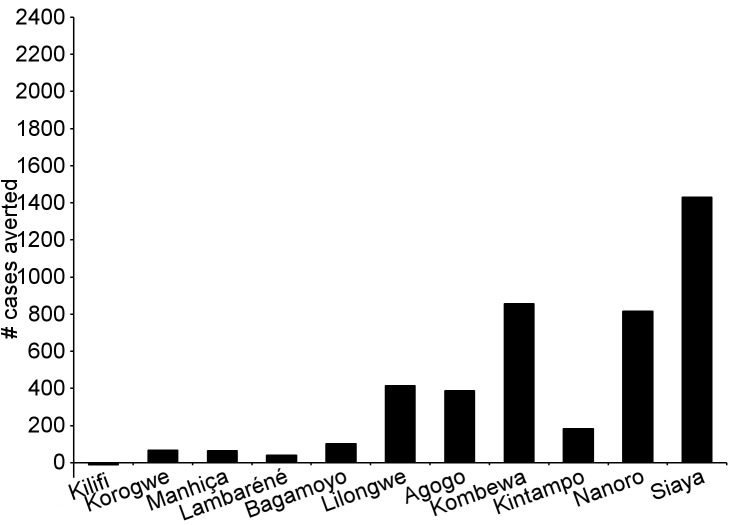
Cases of clinical malaria (secondary case definition) averted during an 18-mo follow-up period in infants 6–12 wk of age at enrollment, by study site, ordered by increasing malaria incidence at each site (per-protocol population). Clinical malaria secondary case definition: illness in a child brought to a study facility with a measured temperature of ≥37.5°C or reported fever within the last 24 h and *P. falciparum* asexual parasitemia at a density of >0 parasites/mm^3^. This definition was used for this analysis because during routine clinical practice these children would normally receive a full course of anti-malarial treatment. Study sites are ordered from lowest (Kilifi) to highest (Siaya) incidence of clinical malaria, defined as a measured or reported fever within previous 24 h and parasite density >0 parasites/mm^3^ (i.e., clinical malaria secondary case definition), measured in control infants 6–12 wk of age at enrollment during 12 mo of follow-up.

**Figure 15:**
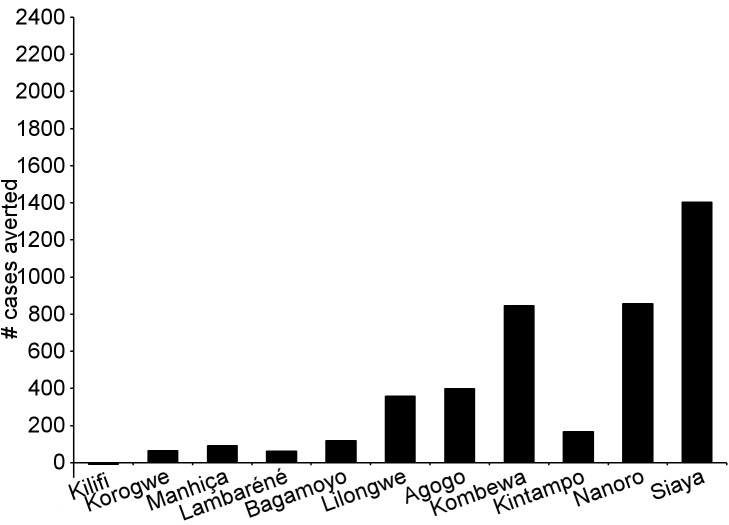
Cases of clinical malaria (secondary case definition) averted during an 18-mo follow-up period in infants 6–12 wk of age at enrollment, by study site, ordered by increasing malaria incidence at each site (intention-to-treat population). Clinical malaria secondary case definition: illness in a child brought to a study facility with a measured temperature of ≥37.5°C or reported fever within the last 24 h and *P. falciparum* asexual parasitemia at a density of >0 parasites/mm^3^. This definition was used for this analysis because during routine clinical practice these children would normally receive a full course of anti-malarial treatment. Study sites are ordered from lowest (Kilifi) to highest (Siaya) incidence of clinical malaria, defined as a measured or reported fever within previous 24 h and parasite density >0 parasites/mm^3^ (i.e., clinical malaria secondary case definition), measured in control infants 6–12 wk of age at enrollment during 12 mo of follow-up.

The number of cases of clinical malaria averted per 1,000 young infants vaccinated with RTS,S/AS01 in the ITT population ranged across sites from −10 to 1,402 (average for the 11 study sites: 449), and the number of severe malaria cases averted ranged from −13 to 37 (average across all sites: 6) during the 18-mo follow-up period (Figures 6–9, 14, and 15; Table S8). One hundred seventeen young infants (1.8%) died during the 20-mo observation period, 83/4,358 (1.9%) infants in the RTS,S/AS01 group and 34/2,179 (1.6%) in the control group. VE was not demonstrated against all-cause mortality, malaria mortality, hospitalized pneumonia, septicemia, or prevalent anemia, nor was there a detectable effect on nutritional status or growth (Tables S7, S9, and S10).

### Immunogenicity

**Figure 16:**
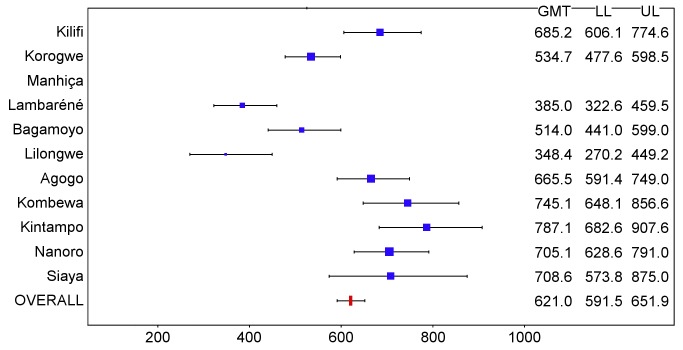
Anti-CS antibody geometric mean titers (EU/ml) in RTS,S/AS01 recipients 1 mo after dose 3 in children 5–17 mo of age at enrollment, ordered by increasing malaria incidence at each study site (per-protocol population). The blue squares reflect the number of participants in the per-protocol population with a valid assay result available 1 mo after dose 3 in each study site. The horizontal bars show the lower limit (LL) and upper limit (UL) of the 95% confidence interval. Study sites are ordered from lowest (Kilifi) to highest (Siaya) incidence of clinical malaria, defined as a measured or reported fever within previous 24 h and parasite density >0 parasites/mm^3^ (i.e., clinical malaria secondary case definition), measured in control infants 6–12 wk of age at enrollment during 12 mo of follow-up.

**Figure 17:**
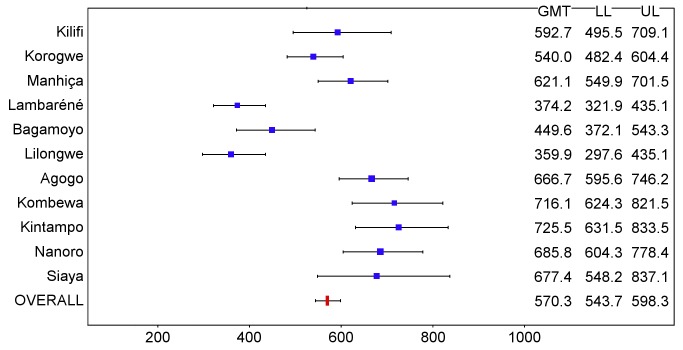
Anti-CS antibody geometric mean titers (EU/ml) in RTS,S/AS01 recipients 1 mo after dose 3 in children 5–17 mo of age at enrollment, ordered by increasing malaria incidence at each study site (intention-to-treat population). The blue squares reflect the number of participants in the per-protocol population with a valid assay result available 1 mo after dose 3 in each study site. The horizontal bars show the lower limit (LL) and upper limit (UL) of the 95% confidence interval. Study sites are ordered from lowest (Kilifi) to highest (Siaya) incidence of clinical malaria, defined as a measured or reported fever within previous 24 h and parasite density >0 parasites/mm^3^ (i.e., clinical malaria secondary case definition), measured in control infants 6–12 wk of age at enrollment during 12 mo of follow-up.

**Figure 18:**
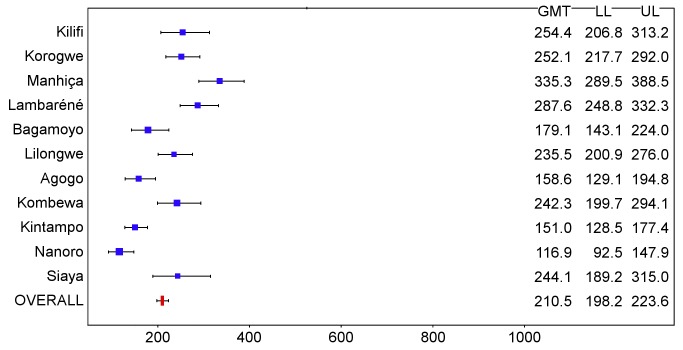
Anti-CS antibody geometric mean titers (EU/ml) in RTS,S/AS01 recipients 1 mo after dose 3 in infants 6–12 wk of age at enrollment, ordered by increasing malaria incidence at each study site (per-protocol population). The blue squares reflect the number of participants in the per-protocol population with a valid assay result available 1 mo after dose 3 in each study site. The horizontal bars show the lower limit (LL) and upper limit (UL) of the 95% confidence interval. Study sites are ordered from lowest (Kilifi) to highest (Siaya) incidence of clinical malaria, defined as a measured or reported fever within previous 24 h and parasite density >0 parasites/mm^3^ (i.e., clinical malaria secondary case definition), measured in control infants 6–12 wk of age at enrollment during 12 mo of follow-up.

**Figure 19:**
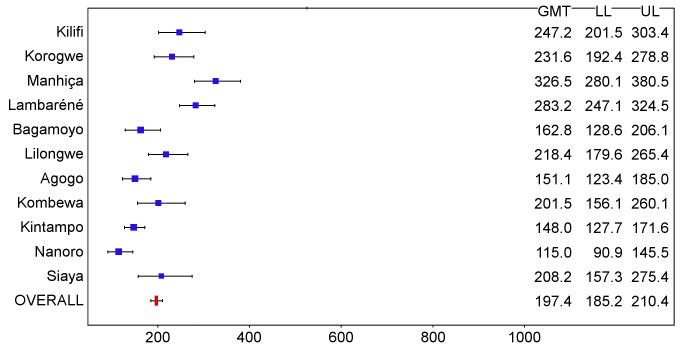
Anti-CS antibody geometric mean titers (EU/ml) in RTS,S/AS01 recipients 1 mo after dose 3 in infants 6–12 wk of age at enrollment, ordered by increasing malaria incidence at each study site (intention-to-treat population). The blue squares reflect the number of participants in the per-protocol population with a valid assay result available 1 mo after dose 3 in each study site. The horizontal bars show the lower limit (LL) and upper limit (UL) of the 95% confidence interval. Study sites are ordered from lowest (Kilifi) to highest (Siaya) incidence of clinical malaria, defined as a measured or reported fever within previous 24 h and parasite density >0 parasites/mm^3^ (i.e., clinical malaria secondary case definition), measured in control infants 6–12 wk of age at enrollment during 12 mo of follow-up.

**Figure 20:**
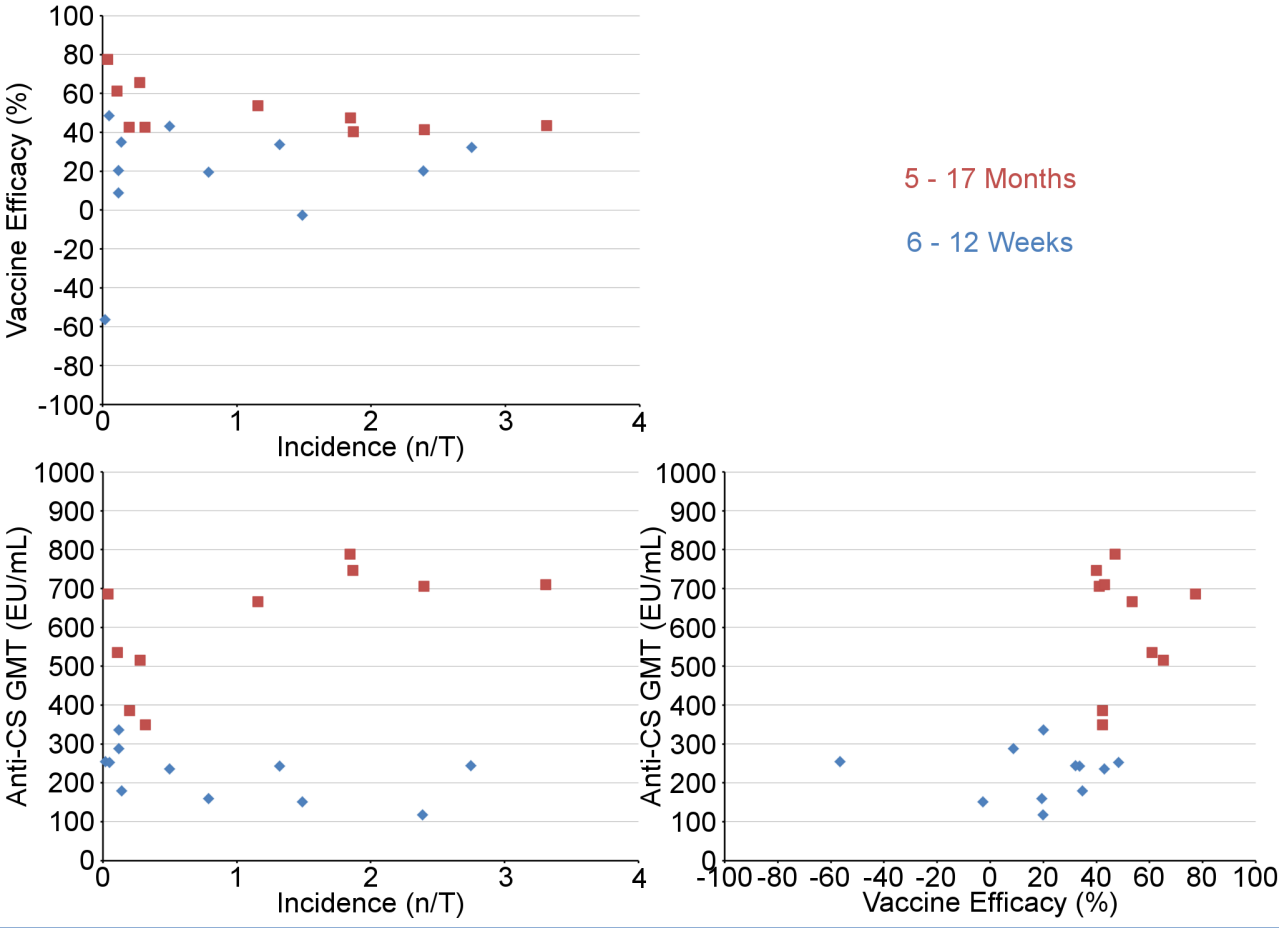
Graphical representation of anti-CS geometric mean titers, vaccine efficacy, and malaria incidence (per-protocol population). Upper left panel: VE against clinical malaria versus malaria incidence (per-protocol population); lower left panel: anti-CS response versus malaria incidence (per-protocol population); lower right panel: anti-CS response versus VE against clinical malaria (per-protocol population). Blue diamonds (infants 6–12 wk) and red squares (children 5–17 mo) represent the study sites. VE (percent) is VE against all episodes of clinical malaria meeting the primary case definition over 18 mo after dose 3. Anti-CS antibody GMT (EU/ml) was measured at 1 mo after dose 3 in the first 200 participants enrolled at each site. Incidence (*n*/total [n/T]: episodes per person-year at risk) is the incidence of clinical malaria (primary case definition) in the control group in the corresponding age category over 18 mo after dose 3.

In children, post-vaccination anti-CS antibody geometric mean titer (GMT) varied from 348 to 787 EU/ml across sites in the per-protocol population (Figures 16 and 17; Table S16), but post-vaccination titers did not explain the differences in level of protection (*p = *0.24) (Table S11). Previous hepatitis B vaccination was not associated with a higher post-vaccination anti-CS antibody response (*p* = 0.73; Table S12). However, younger age at the time of vaccination (5–11 mo versus 12–17 mo) and living in a higher transmission setting were associated significantly with higher post-vaccination anti-CS responses (*p<*0.001 and *p<*0.001, respectively) (Table S12). Young infants had a lower post-vaccination anti-CS antibody GMT than children (anti-CS antibody GMT range across sites: 117 to 335 EU/ml, per-protocol population), and, in contrast to what was observed in children, higher anti-CS antibody titers were associated with lower malaria incidence (*p<*0.001) (Figures 18 and 19; Tables S11 and S16). Younger age (6 wk versus 7–12 wk) and the presence of anti-CS antibodies prior to vaccination were associated with a lower post-vaccination anti-CS antibody response (*p* = 0.003 and *p<*0.001, respectively) (Tables S12 and S13). A graphic depiction of the relationships between anti-CS antibody GMT, malaria incidence, and VE is shown in Figure 20.

### Safety

SAEs were less frequent in the children vaccinated with RTS,S/AS01 than in control children: 18.6% (95% CI 17.6% to 19.6%) versus 22.7% (95% CI 21.2% to 24.3%), while no difference in frequency was found between vaccinated and control young infants: 22.0% (95% CI 20.8% to 23.3%) versus 23.1% (95% CI 21.3% to 24.9%) (Table S14). The previously reported imbalance in the incidence of meningitis persisted [15],[16]. Meningitis was reported as an SAE in 17 children (16 cases among the 5,949 children in the RTS,S/AS01 group and one case among the 2,974 children in the control group [RR = 8.0 (95% CI 1.1 to 60.3)]). No pathogen was identified in 11 cases, ten in the RTS,S/AS01 group and one in the control group. A pathogen was identified in six cases (four meningococcal, one pneumococcal, and one *Haemophilus influenzae*), all in the RTS,S/AS01 group. Six children with reported meningitis died, five in the RTS,S/AS01 group and one in the control group. Meningitis was reported as an SAE in 12 young infants (nine cases among 4,358 young infants in the RTS,S/AS01 group and three among 2,179 in the control group [RR = 1.50 (95% CI 0.41 to 5.55)]). No pathogen was identified in five cases, three in the RTS,S/AS01 group and two in the control group. A pathogen was identified in seven cases (four pneumococcus and three salmonella), six in the RTS,S/AS01 group and one in the control group. Four infants with reported meningitis died, two in the RTS,S/AS01 group and two in the control group. Meningitis cases were not temporally related to vaccination (Figure S9A). Most study sites reported 1–3 cases of meningitis during the 18-mo follow-up period, while the site in Lilongwe, Malawi, reported 11 cases and the site in Kombewa, Kenya, reported five cases (Figure S9B). An overview of the analyses reported here is given in Table S17.

## Discussion

RTS,S/AS01 provided protection against a range of predefined end points, including clinical and severe malaria, over an 18-mo follow-up period among children aged 5–17 mo at first vaccination across a wide range of malaria transmission settings. VE against clinical malaria was 40% or higher in each setting but varied significantly between sites. In contrast to previous phase 2 trials, which found that efficacy decreased with increasing exposure [21],[22], the variation in VE between study sites seen in this trial could not be explained by differences in the intensity of malaria transmission between sites, as measured by the incidence of clinical malaria in control children or control young infants applied as an average transmission level for all participants at a given site. However, there are several caveats to this finding. First, the presence of moderate anemia at enrollment, which may reflect malaria exposure at the individual level, was negatively associated with VE. Second, in children, while there was no correlation found between VE and transmission in a multivariate model, VE appeared higher at sites with a lower incidence of malaria (Figure 4). Differences in the incidence of clinical malaria between sites may be influenced by various genetic and environmental factors independent of the transmission level. Differences in anti-CS antibody GMT across sites did not explain differences in efficacy.

VE against clinical malaria was lower in young infants than in children. Severe malaria was more frequent, although the difference was not significant statistically, among young infant RTS,S/AS01 recipients compared with controls during the final follow-up period in this analysis, 12–18 mo after dose 3. This could be a chance finding or could indicate that the vaccine interfered with the acquisition of natural immunity [23], an effect perhaps compounded by prompt access to diagnosis and treatment. Further follow-up is planned, and will include close monitoring of the 1/3 of participants who received the RTS,S/AS01 primary vaccination series but not the RTS,S/AS01 booster dose.

The current analysis has provided some clues as to why young infants respond less effectively to RTS,S/AS01 than children. Maternal antibodies are likely to have played a role, as young infants with detectable anti-CS antibodies at enrollment had a lower post-vaccination anti-CS response than young infants without detectable anti-CS antibodies at enrollment, and a high post-vaccination anti-CS antibody titer was associated with VE in young infants. However, maternal antibodies cannot explain fully the lower anti-CS response in young infants, as those without detectable maternally derived anti-CS antibodies still had a lower post-vaccination anti-CS antibody GMT than did vaccinated children. Immune interference due to administration of RTS,S/AS01 at the same time as EPI vaccines remains a possible factor. The fact that phase 2 trials showed that co-administration in one setting was associated with lower anti-CS responses than staggered administration in another setting supports this hypothesis [9],[10]. A suppressive effect from exposure to malaria antigens in utero might be more marked in young infants than in older children, who have had a longer period to acquire immunity [24],[25]. Finally, the immature immune system of young infants may not respond as well as the immune system of older children. In contrast with findings from a phase 2 trial [26], we found no evidence that priming with hepatitis B vaccine in children explained their enhanced anti-CS antibody response.

VE waned over time in both young infants and children, as reported previously [15],[16]. An analysis by 6-mo period showed that the vaccine provided protection against clinical malaria throughout the follow-up period, but that there was a progressive decline in efficacy. However, these analyses need to be treated with caution; although the study groups were well matched during the first 6-mo period, this was not the case subsequently as children in the control group experienced more clinical malaria than children in the RTS,S/AS01 group, and thereby may have acquired natural immunity, making the vaccine appear less effective by comparison. It will be important to determine whether VE can be restored by a vaccine booster dose, which has been given after the 18-mo follow-up to half of the RTS,S/AS01 recipients.

The trial was conducted to a high ethical and clinical standard, with harmonization of procedures across sites [27],[28]. Nonetheless, some anomalous findings were noted. The lack of VE among young infants in Kintampo, Ghana, despite 47% VE among children and a post-vaccination anti-CS antibody GMT within the range seen at other sites, was unexpected, as was the lack of efficacy against prevalent malaria infection 18 mo post-vaccination in children at Siaya, Kenya. These may be chance findings but warrant further observation.

Conducting this trial to high standards required facilitating access of trial participants to both outpatient and inpatient care; purchasing additional clinical and laboratory equipment; ensuring reliable supplies of essential medications, oxygen, and blood; and increasing clinical staffing. Consequently, mortality was very low in both study arms. This may explain why, in contrast to the results of trials of other effective malaria control tools, such as ITNs or prophylactic drugs, we did not find any impact of RTS,S/AS01 on overall mortality [29],[30] or on nutritional status [31]. Neither did we find an impact of the vaccine on the incidence of hospitalized pneumonia or septicemia. The impact of RTS,S/AS01 on mortality and co-infections might be greater were the vaccine to be deployed in non-trial situations, where prompt, high-quality care is less readily available.

The observed interaction between moderate anemia and decreased VE in older children is intriguing. As stated previously, anemia may be a sensitive indicator of individual exposure to malaria. Alternatively, anemia might indicate active malaria infection, which can interfere with the development of a robust immune response to vaccination [32]. Helminth infection is another frequent cause of anemia in toddlers and young children in sub-Saharan Africa, and helminth infection can also result in a reduced immunological response to vaccination [33]. Malnutrition is frequently associated with anemia, but malnutrition was not found to be a determinant of VE in our multivariate model.

The incidence of SAEs overall was similar in participants in each group, but the imbalance in reported cases of meningitis, noted previously [15],[16], has persisted, and the difference between groups has become statistically significant in older children. No obvious explanation for this association has been found, a temporal relationship to vaccination is lacking, and biological plausibility is low. A causal relationship cannot be confirmed or excluded at this point. The occurrence of meningitis will be followed closely during the remainder of the trial, with particular attention paid to the possible impact of a booster dose of vaccine.

As with all large-scale, multicenter trials, this study had some weaknesses. Although strenuous efforts were made to standardize clinical and laboratory methods across study sites, there may have been some variations in the clinical characteristics of cases recruited at individual centers that could have contributed to the variations in the level of VE seen across sites. It is possible that variations in factors such as the prevalence of co-infections (including HIV), micronutrient deficiencies, or socio-economic status could have contributed to the heterogeneity in immunogenicity and VE seen between sites, but these factors were not evaluated systematically. Finally, the incidence of clinical malaria in infants was used as an indirect measure of the intensity of malaria transmission at each site, and it is possible that this is not a very discriminatory factor, as it may be influenced by other variables such as the transfer of maternal antibodies against malaria or access to treatment. A direct measure of exposure such as the entomological inoculation rate might have provided a different ranking order between sites. Variations in the immunogenicity and VE of RTS,S/AS01 between sites could not be explained solely by variations in the level of transmission of malaria, but the nature of other factors that may be involved remains to be determined.

During 18 mo of follow-up, RTS,S/AS01 prevented many cases of clinical and severe malaria across the 11 sites in the trial. Despite its lower efficacy in young infants, RTS,S/AS01 also prevented a substantial number of cases of clinical malaria in young infants. Translated to the population at risk of malaria, reductions in clinical cases on this scale as a result of vaccination with RTS,S/AS01 would have a major public health impact.

## Supporting Information

Figure S1 **Study design.**
(DOCX)

Figure S2 **Baseline characteristics and malaria control measures in place at each study site, ordered by increasing malaria incidence (intention-to-treat population).**
(DOCX)

Figure S3 **Cumulative incidence of first or only episodes of clinical malaria (primary case definition) (per-protocol population).**
(DOCX)

Figure S4 **Model of vaccine efficacy against all episodes of clinical malaria (primary case definition) over time (per-protocol population).**
(DOCX)

Figure S5 **Overall incidence of clinical and severe malaria (primary case definitions) by 6-mo periods (per-protocol and intention-to-treat populations).**
(DOCX)

Figure S6 **Reduction in incidence of all episodes of clinical malaria (primary case definition) during each 6-mo period ordered by malaria incidence (per-protocol and intention-to-treat populations).**
(DOCX)

Figure S7 **Overall survival curves (intention-to-treat population).**
(DOCX)

Figure S8 **Vaccine efficacy against all episodes of clinical malaria (primary case definition) during a 12-mo follow-up period after dose 3 ordered by increasing malaria incidence.**
(DOCX)

Figure S9 **Time-to-onset distribution of the meningitis cases after dose 1, dose 2, and dose 3 within 600 d post-vaccination for both age categories (intention-to-treat population).**
(DOCX)

Table S1 **List of ethics committees and review boards, and investigational centers and affiliated partners.**
(DOCX)

Table S2 **Case definitions of severe malaria and algorithm for the evaluation of a hospital admission as a potential case of severe malaria.**
(DOCX)

Table S3 **Number of children and infants enrolled at each study site and number of participants contributing to the per-protocol population, ordered by increasing malaria incidence.**
(DOCX)

Table S4 **Incidence of clinical malaria (secondary case definition) in the 6–12-wk age category during a 12-mo follow-up period after dose 3, ordered by increasing malaria incidence.**
(DOCX)

Table S5 **Vaccine efficacy against all episodes of clinical malaria (primary and secondary case definitions) during an 18-mo follow-up period after dose 3 in the 5–17-mo and 6–12-wk age categories, ordered by increasing malaria incidence.**
(DOCX)

Table S6 **Determinants of vaccine efficacy (incidence of clinical malaria primary case definition, all episodes) full models and final models during an 18-mo follow-up period in the 5–17-mo and 6–12-wk age categories (per-protocol population).**
(DOCX)

Table S7 **Protective efficacy against the prevalence of** ***P. falciparum*** **parasitemia and anemia at 18 mo after dose 3 in the 5–17-mo and 6–12-wk age categories, ordered by increasing malaria incidence.**
(DOCX)

Table S8 **Cases of clinical malaria, severe malaria, malaria hospitalization, and all-cause hospitalization averted per 1,000 infants or children vaccinated with RTS,S/AS01 by 6-mo periods and overall in the 5–17-mo and 6–12-wk age categories, ordered by increasing malaria incidence at each site.**
(DOCX)

Table S9 **Vaccine effect on growth in the 5–17-mo and 6–12-wk age categories.**
(DOCX)

Table S10 **Overall vaccine efficacy against hospitalization with pneumonia or with sepsis, and malaria mortality and all-cause mortality during an 18-mo follow-up period after dose 3 in the 5–17-mo and 6–12-wk age categories.**
(DOCX)

Table S11 **Effect of anti-CS response on the incidence of clinical malaria (primary case definition) in RTS,S/AS01 recipients during an 18-mo follow-up period in the 5–17-mo and 6–12-wk age categories (per-protocol population).**
(DOCX)

Table S12 **Determinants of anti-CS response during an 18-mo follow-up period in the 5–17-mo and 6–12-wk age categories, results from linear regression analysis (per-protocol population).**
(DOCX)

Table S13 **Univariate analysis of effect of covariates on anti-CS responses 1 mo after dose 3 in RTS,S/AS01 recipients in the 5–17-mo and 6–12-wk age categories (per-protocol population).**
(DOCX)

Table S14 **Percentage of infants/children in the 5–17-mo and 6–12-wk age categories reporting a serious adverse event during 20 mo after dose 1 by MedDRA preferred term (intention-to-treat population).**
(DOCX)

Table S15 **Vaccine efficacy against clinical and severe malaria in all children enrolled in the 5–17-mo age category during a 12-mo follow-up period after dose 3.**
(DOCX)

Table S16 **Seropositivity rates and geometric mean titers for anti-CS antibodies at baseline and 1 mo after dose 3 in the 5–17-mo and 6–12-wk age categories by site and overall.**
(DOCX)

Table S17 **Overview of the analyses reported.**
(DOCX)

Checklist S1 **CONSORT checklist.**
(DOCX)

Protocol S1 **A phase III, double-blind (observer-blind), randomized, controlled multicenter study to evaluate, in infants and children, the efficacy of the RTS,S/AS01 candidate vaccine against malaria disease caused by** ***P. falciparum*** **infection, across diverse malaria transmission settings in Africa.**
(PDF)

Text S1 **Supplementary methods.**
(DOCX)

Text S2 **List of institutional review boards.**
(DOC)

## Abbreviations

anti-CS: anti-circumsporozoite
EPI: Expanded Program on Immunization
GMT: geometric mean titer
ITN: insecticide-treated net
ITT: intention to treat
MedDRA: Medical Dictionary for Regulatory Activities
RR: relative risk
SAE: serious adverse event
VE: vaccine efficacy
