# Supplementary material for: Efficacy and Safety of the RTS,S/AS01 Malaria Vaccine during 18 Months after Vaccination: A Phase 3 Randomized, Controlled Trial in Children and Young Infants at 11 African Sites
Source: PLoS Med. 2014 Jul 29;11(7):e1001685. doi: 10.1371/journal.pmed.1001685 (PMC4114488; doi:10.1371/journal.pmed.1001685)
Supplement: Figure S1 — Study design. (DOCX) [file pmed.1001685.s001.docx]

## Supplementary figure 1. Study design


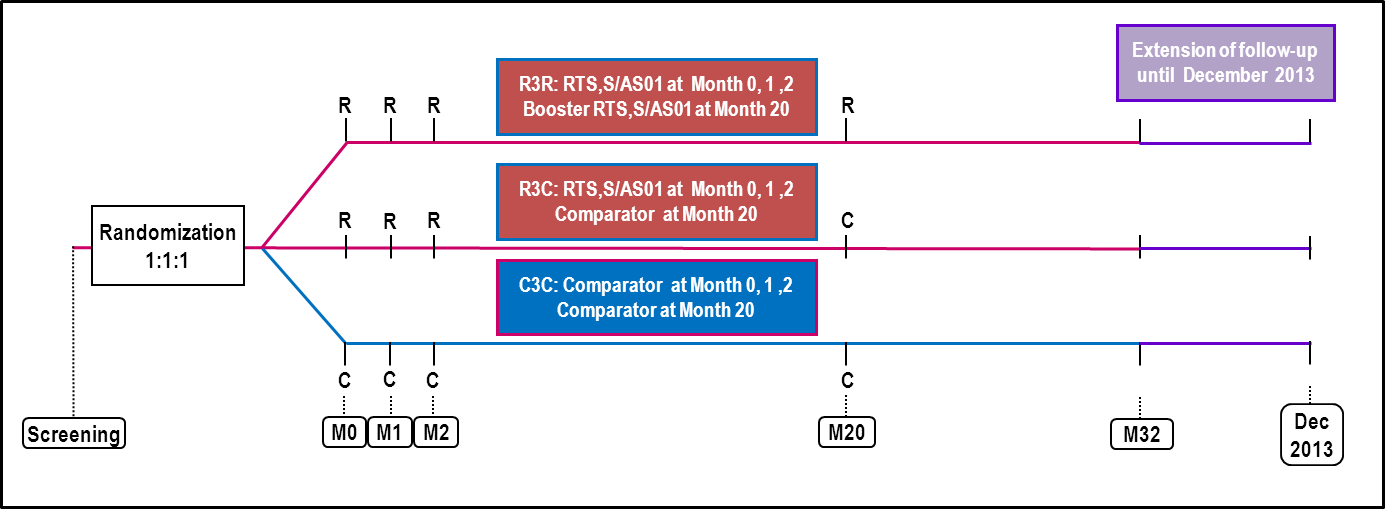


M = Study Month.

The study has been extended until 31 December 2013. The total follow-up time of participant will vary depending on their enrollment date. The mean follow-up time will be 49 months post Dose 1 (range: 41-55) for the children enrolled at the age of 5-17 months and 41 months post Dose 1 (range: 32-48) for the infants enrolled at the age of 6-12 weeks.
