# Supplementary material for: Efficacy and Safety of the RTS,S/AS01 Malaria Vaccine during 18 Months after Vaccination: A Phase 3 Randomized, Controlled Trial in Children and Young Infants at 11 African Sites
Source: PLoS Med. 2014 Jul 29;11(7):e1001685. doi: 10.1371/journal.pmed.1001685 (PMC4114488; doi:10.1371/journal.pmed.1001685)
Supplement: Figure S2 — Baseline characteristics and malaria control measures in place at each study site, ordered by increasing malaria incidence (intention-to-treat population). (DOCX) [file pmed.1001685.s002.docx]

## Supplementary figure 2. Baseline characteristics and malaria control measures in place at each study site, ordered by increasing malaria incidence (intention-to-treat population)

| **A.** Children 5-17 months of age at enrollment | | **B.** Infants 6-12 weeks of age at enrollment | | | |
| --- | --- | --- | --- | --- | --- |
| **Age (Months)** | | **Age (Weeks)** | | | |
| **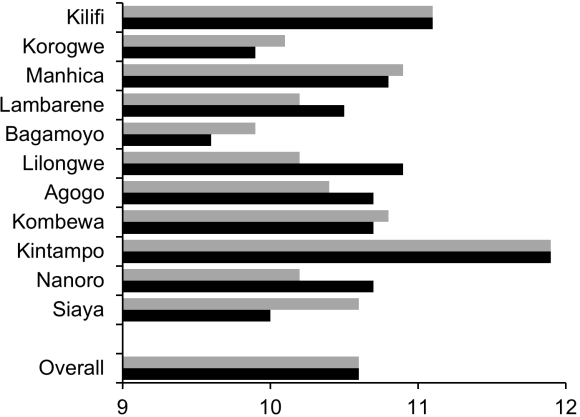** | | **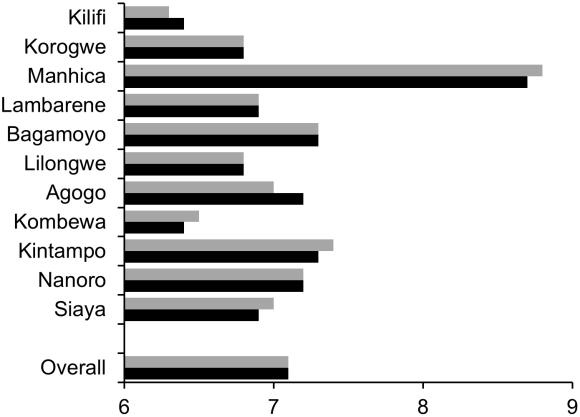** | | | |
| **Gender (%Male)** | | **Gender (%Male)** | | | |
| **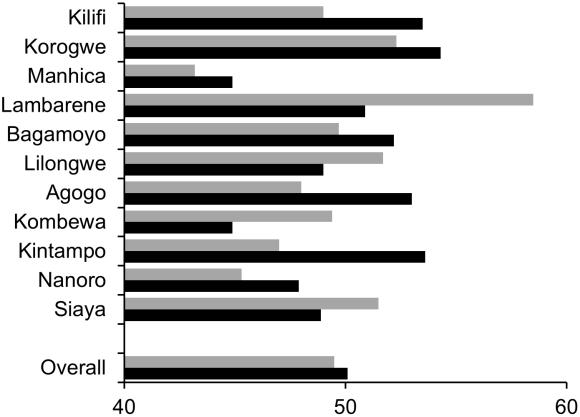** | | **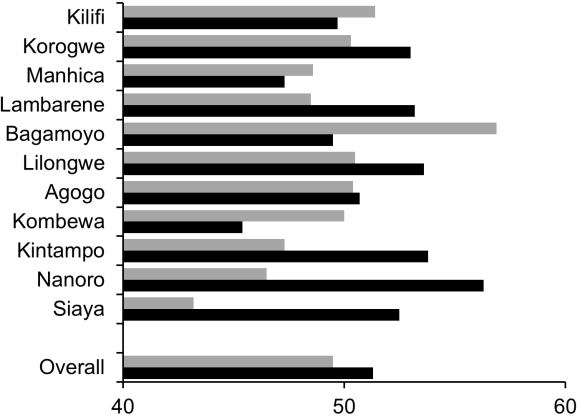** | | | |
| **Height-for-age Z-score** | | **Height-for-age Z-score** | | | |
| **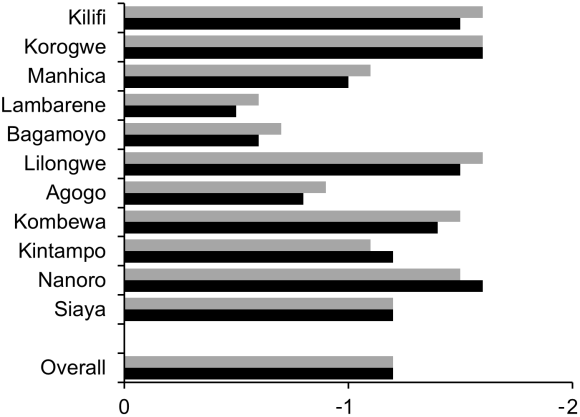** | | **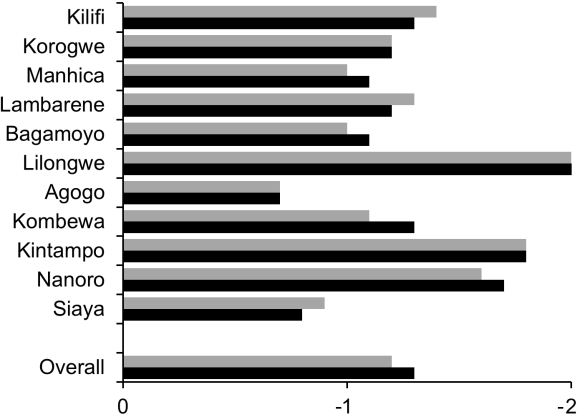** | | | |
|  | RTS,S/AS01 vaccine | |  | Control vaccine |  |
| *Figure continues on next page* | | |  | |  |
| **A.** Children 5-17 months of age at enrollment | | | **B.** Infants 6-12 weeks of age at enrollment | |  |
| **Weight-for-age Z-score** | | | **Weight-for-age Z-score** | |  |
| 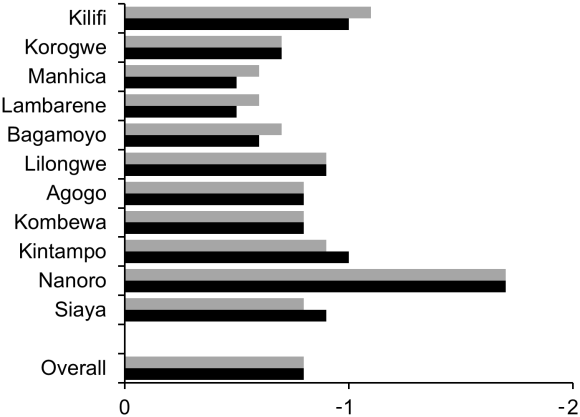 | | | 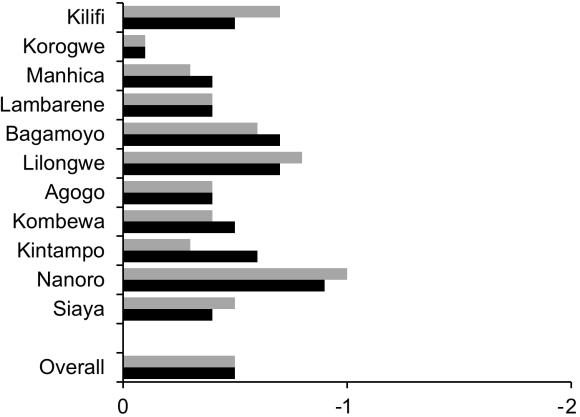 | |  |
| **Distance from outpatient facility (km)** | | | **Distance from outpatient facility (km)** | |  |
| 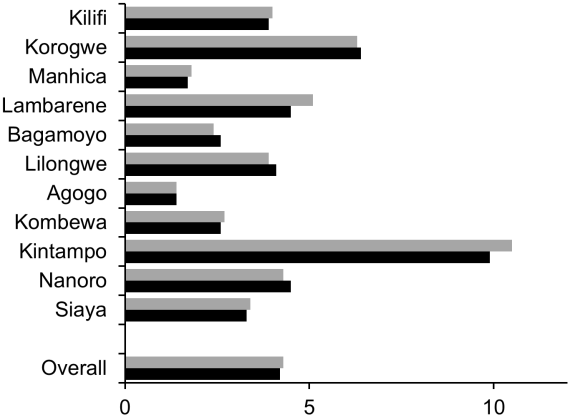 | | | **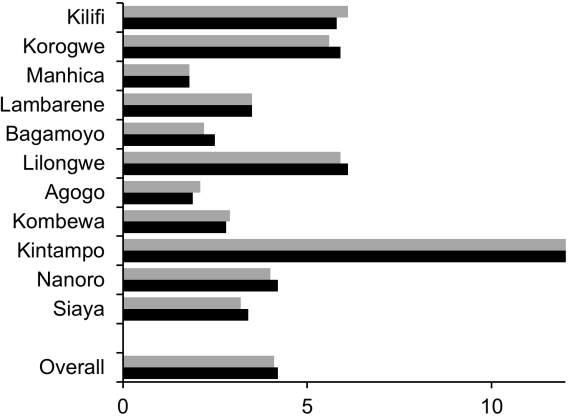** | |  |
| **Distance from inpatient facility (km)** | | | **Distance from inpatient facility (km)** | |  |
| 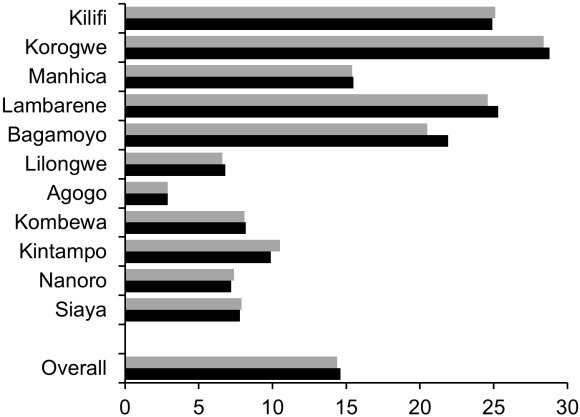 | | | **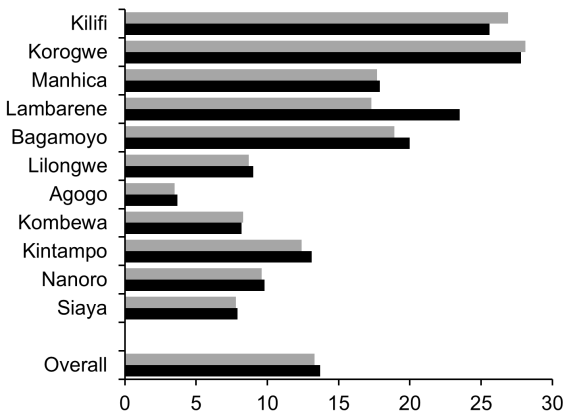** | |  |
| **Hemoglobin at baseline (g/dL)** | | | **Hemoglobin at baseline (g/dL)** | |  |
| 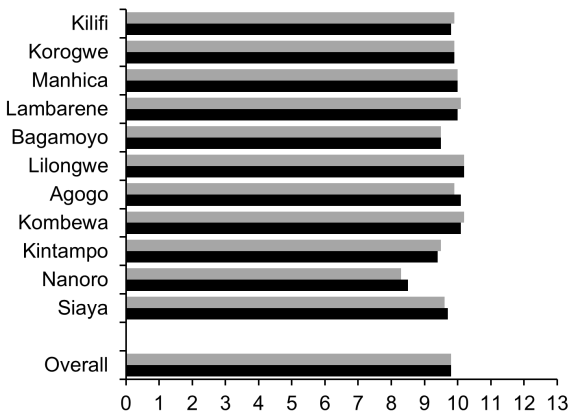 | | | **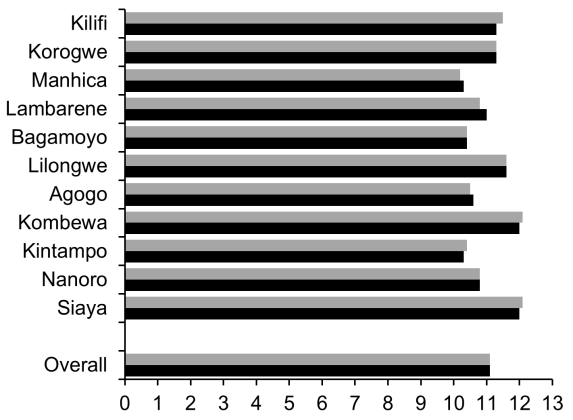** | |  |
|  | RTS,S/AS01 vaccine | |  | Control vaccine |  |
| *Figure continues on next page* | | |  | |  |
| **A.** Children 5-17 months of age at enrollment | | | **B.** Infants 6-12 weeks of age at enrollment | |  |
| **Prevalence of moderate anemia at enrollment (%)** | | | **Prevalence of moderate anemia at enrollment (%)** | |  |
| 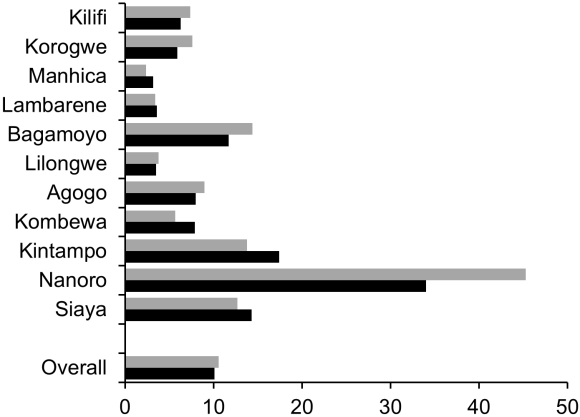 | | | 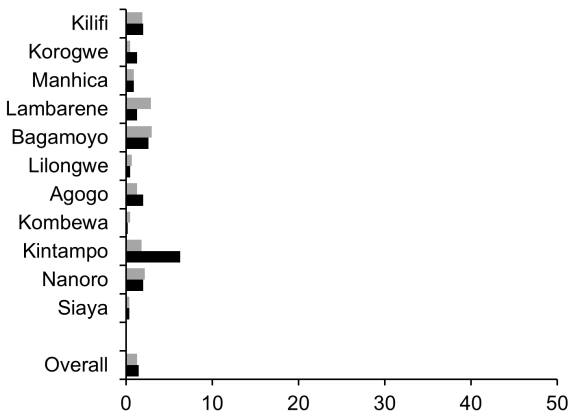 | |  |
| **No prior HepB vaccination (%)** | | | **No prior HepB vaccination (%)** | |  |
| 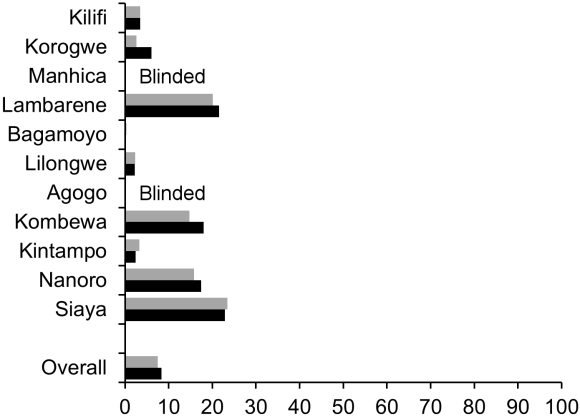 | | | 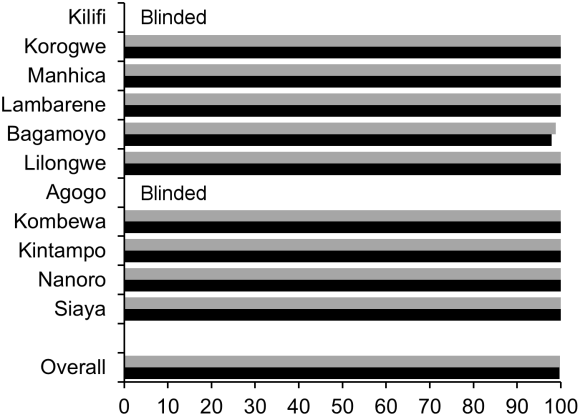 | |  |
| **IPTi Coverage (%)** | | | **IPTi Coverage (%)** | |  |
| 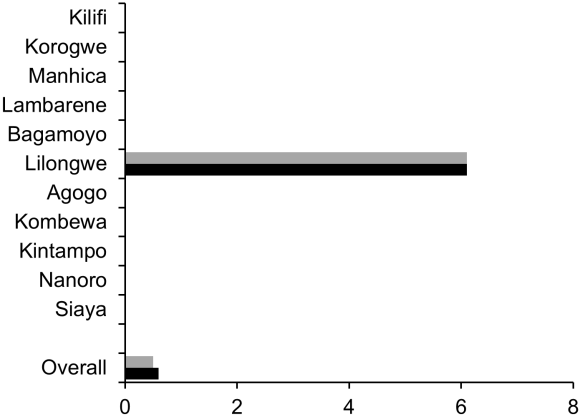 | | | 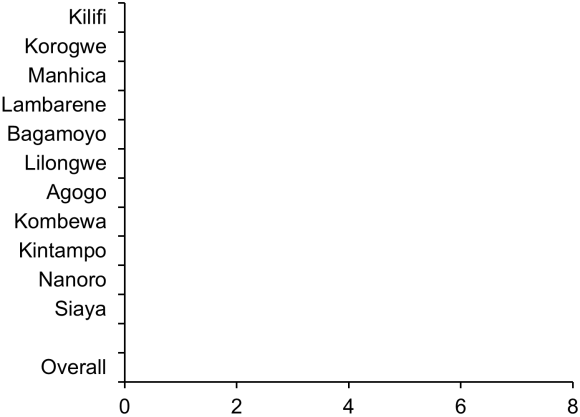 | |  |
| **ITN Coverage (%)** | | | **ITN Coverage (%)** | |  |
| 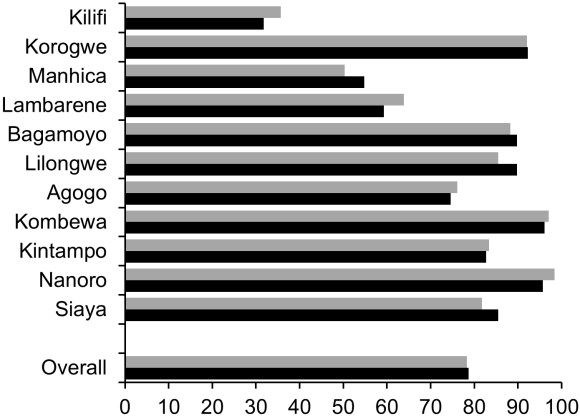 | | | 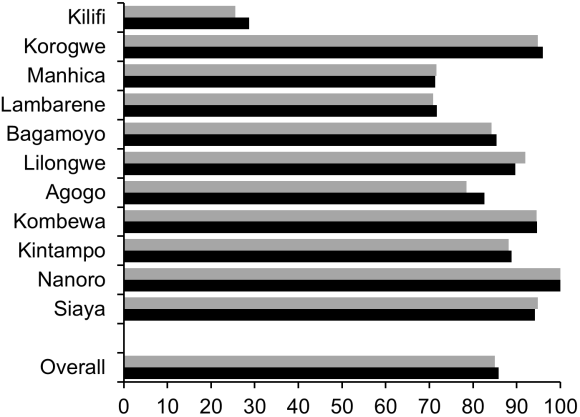 | |  |
|  | RTS,S/AS01 vaccine | |  | Control vaccine |  |
| *Figure continues on next page* | | |  | |  |
| **A.** Children 5-17 months of age at enrollment | | | **B.** Infants 6-12 weeks of age at enrollment | |  |
| **IRS Coverage (%)** | | | **IRS Coverage (%)** | |  |
| 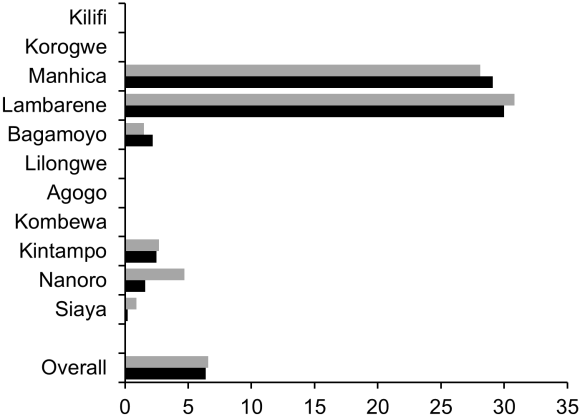 | | | 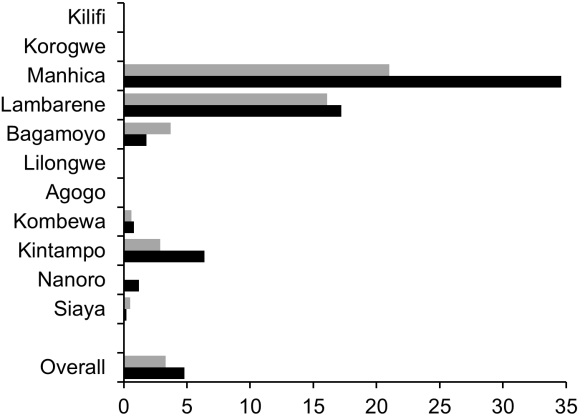 | |  |
|  | RTS,S/AS01 vaccine | |  | Control vaccine |  |

Study sites are ordered from lowest (Kilifi) to highest (Siaya) incidence of clinical malaria, defined as a measured or reported fever within previous 24h and parasite density >0 parasites per cubic millimeter (i.e. clinical malaria secondary case definition), measured in control infants 6-12 weeks of age at enrollment during 12 months of follow-up.

Blinded = data are not presented to maintain individual subject blinding.
