# Supplementary material for: Efficacy and Safety of the RTS,S/AS01 Malaria Vaccine during 18 Months after Vaccination: A Phase 3 Randomized, Controlled Trial in Children and Young Infants at 11 African Sites
Source: PLoS Med. 2014 Jul 29;11(7):e1001685. doi: 10.1371/journal.pmed.1001685 (PMC4114488; doi:10.1371/journal.pmed.1001685)
Supplement: Figure S3 — Cumulative incidence of first or only episodes of clinical malaria (primary case definition) (per-protocol population). (DOCX) [file pmed.1001685.s003.docx]

## Supplementary figure 3. Cumulative incidence of first or only episodes of clinical malaria (primary case definition) (per-protocol population)

| **A.** Children 5-17 months of age at enrollment | **B.** Infants 6-12 weeks of age at enrollment |
| --- | --- |
|  |  |

R3R+R3C = RTS,S/AS01 primary schedule with or without booster.

C3C = Control.

Clinical malaria primary case definition: Illness in a child brought to a study facility with a temperature of ≥ 37.5°C and *P. falciparum* asexual parasitemia at a density of > 5000 parasites per cubic millimeter or a case of malaria meeting the primary case definition of severe malaria.
