# Supplementary material for: Efficacy and Safety of the RTS,S/AS01 Malaria Vaccine during 18 Months after Vaccination: A Phase 3 Randomized, Controlled Trial in Children and Young Infants at 11 African Sites
Source: PLoS Med. 2014 Jul 29;11(7):e1001685. doi: 10.1371/journal.pmed.1001685 (PMC4114488; doi:10.1371/journal.pmed.1001685)
Supplement: Figure S4 — Model of vaccine efficacy against all episodes of clinical malaria (primary case definition) over time (per-protocol population). (DOCX) [file pmed.1001685.s004.docx]

## Supplementary figure 4. Model of vaccine efficacy against all episodes of clinical malaria (primary case definition) over time (per-protocol population)

| **A.** Children 5-17 months of age at enrollment | **B.** Infants 6-12 weeks of age at enrollment |
| --- | --- |
| **Overall - model=group*(stop**(0.5))** | **Overall - model=group*(stop**(0.5))** |
| 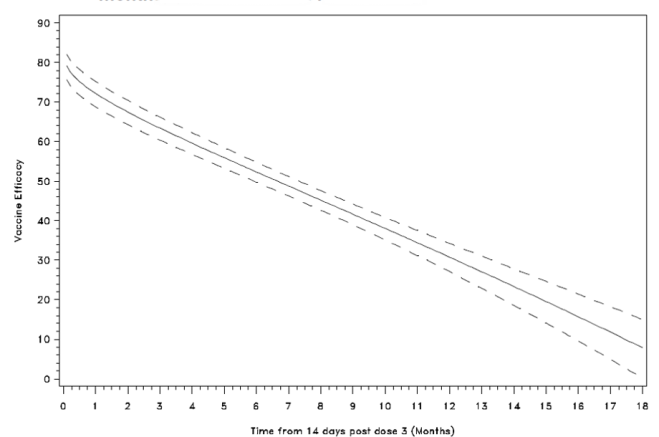 | 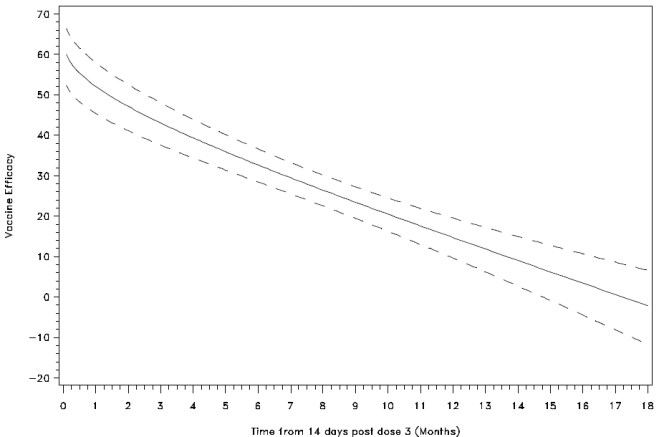 |
|  |  |
| **Kilifi - model=No time-varying covariates** | **Kilifi - model=No time-varying covariates** |
| 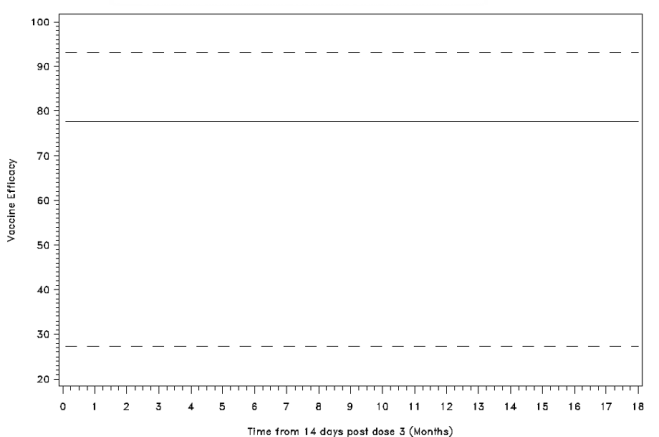 | 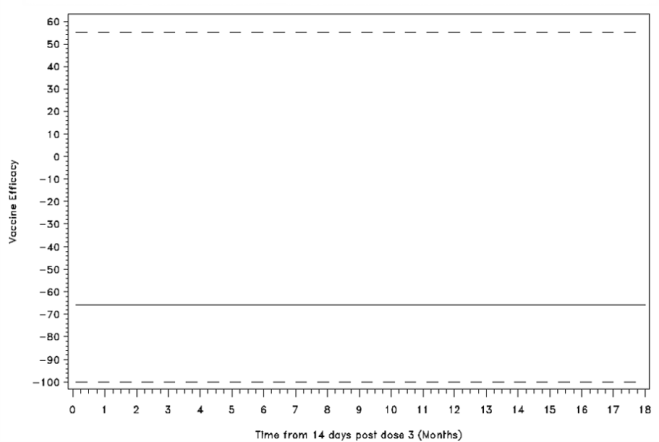 |
| **Korogwe - model=No time-varying covariates** | **Korogwe - model=No time-varying covariates** |
| 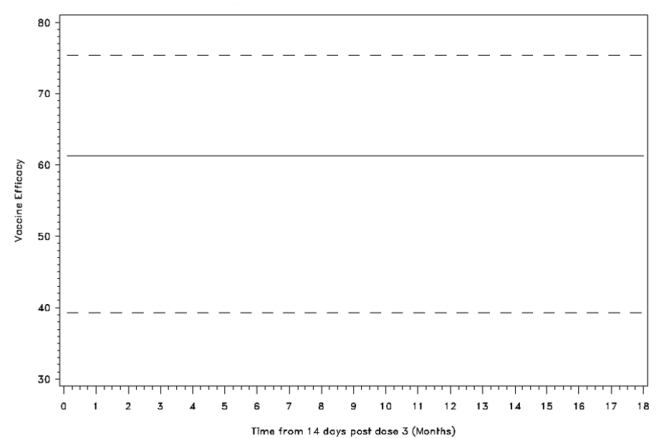 | 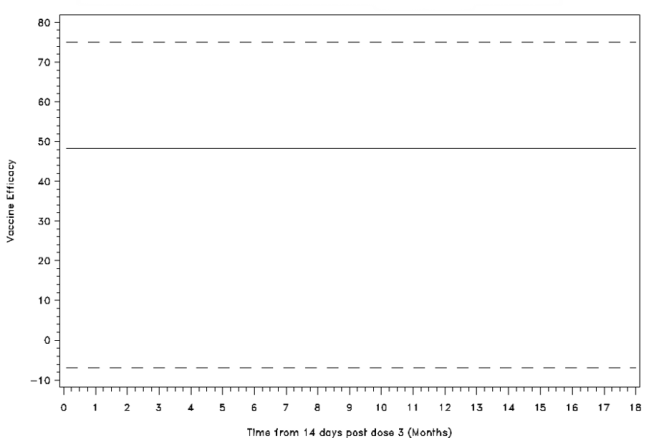 |

*Figure continues on next page*

| **A.** Children 5-17 months of age at enrollment | | | **B.** Infants 6-12 weeks of age at enrollment | | |
| --- | --- | --- | --- | --- | --- |
|  | | | **Manhiça - model=No time-varying covariates** | | |
|  | | | 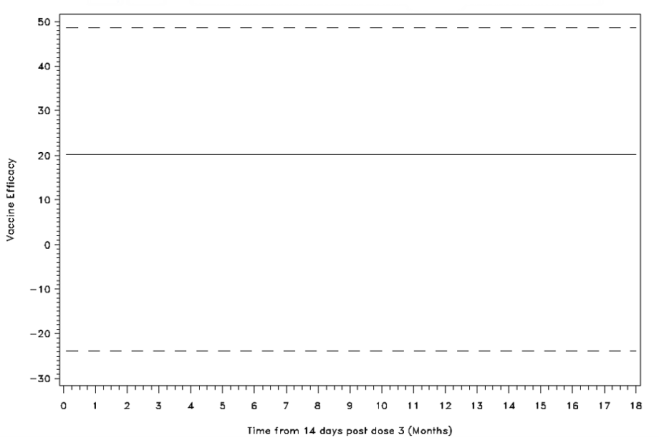 | | |
| **Lambarene - model=group*(stop**(-1))** | | | **Lambarene - model=group*(stop**(-2))** | | |
| 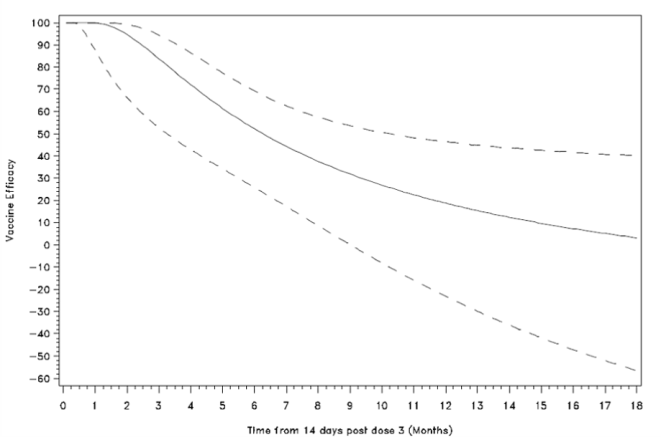 | | | 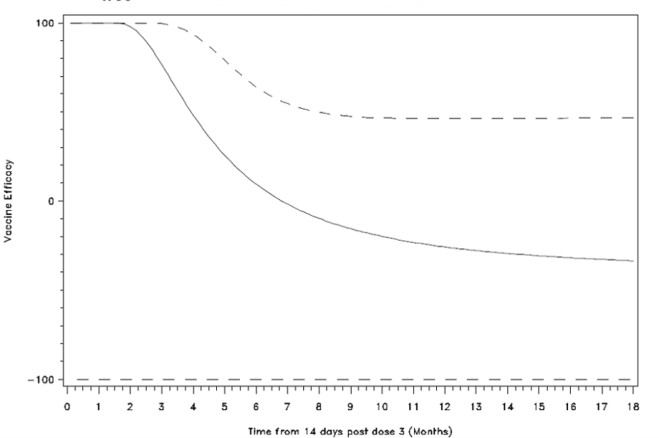 | | |
| **Bagamoyo - model=group*(stop)** | | | **Bagamoyo - model=No time-varying covariates** | | |
| 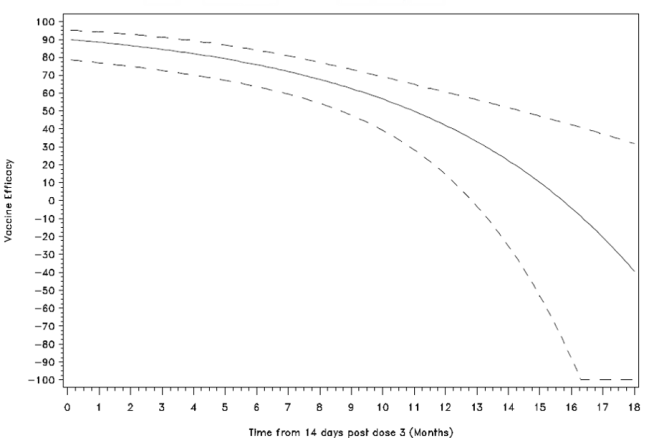 | | | 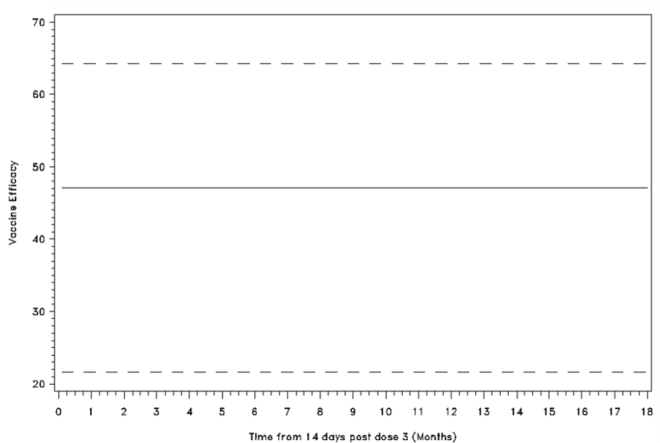 | | |
| **Lilongwe - model=No time-varying covariates** | | | **Lilongwe - model=group*(stop**(0.5))** | | |
| 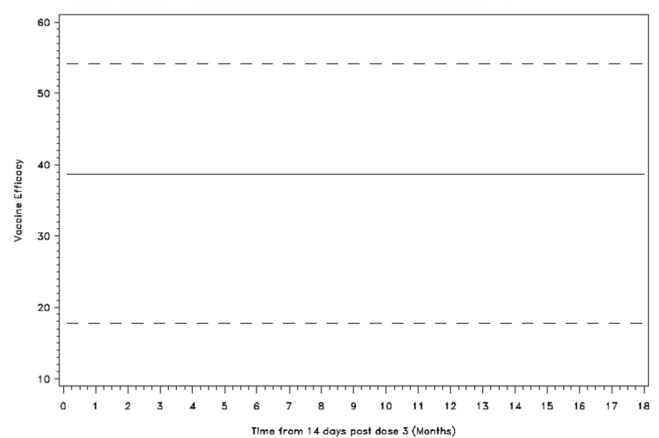 | | | 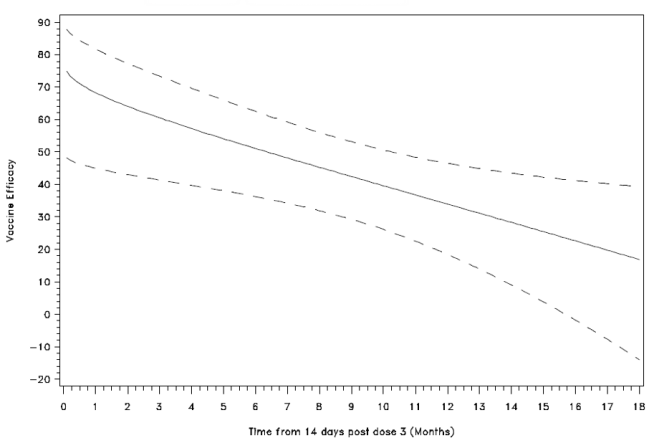 | | |
| *Figure continues on next page* | | | | | |
| **A.** Children 5-17 months of age at enrollment | | | **B.** Infants 6-12 weeks of age at enrollment | | |
| **Agogo - model=group*(stop**(0.5))** | | | **Agogo - model=group*(stop**(0.5))** | | |
| 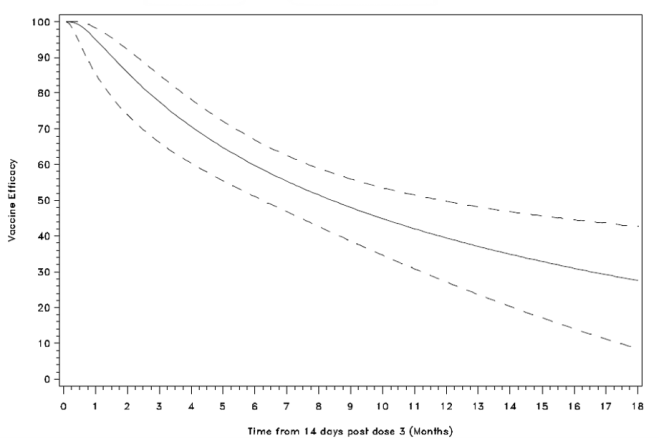 | | | 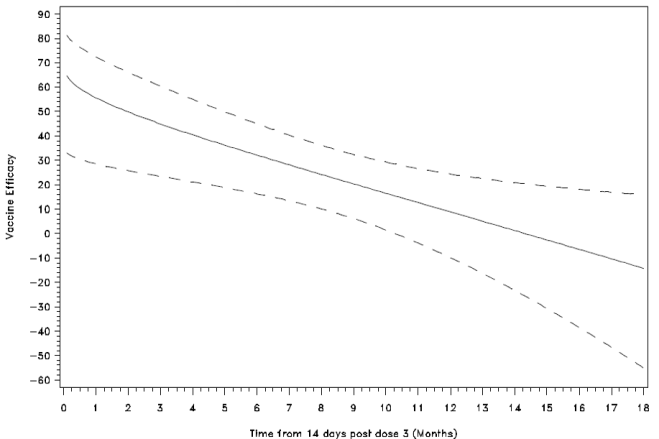 | | |
| **Kombewa - model=group*(stop**(0.5))** | | | **Kombewa - model=group*(stop)** | | |
| 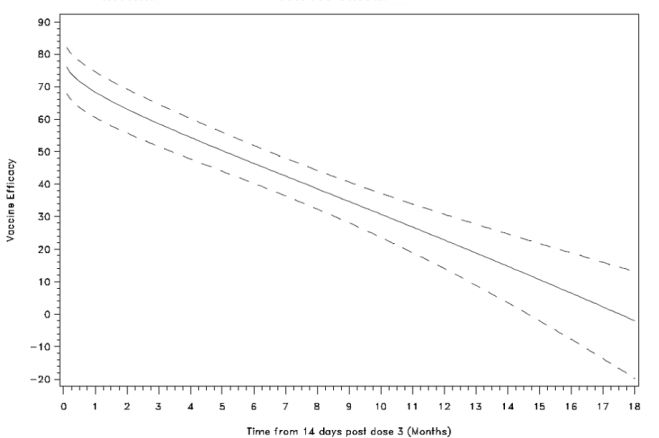 | | | 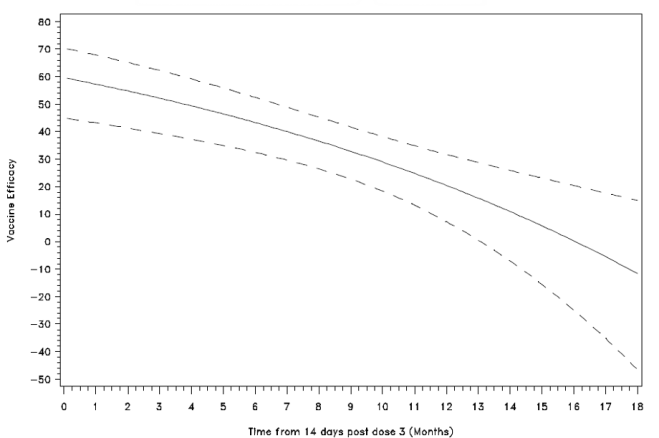 | | |
| **Kintampo - model=group*(log(stop))** | | | **Kintampo - model=No time-varying covariates** | | |
| 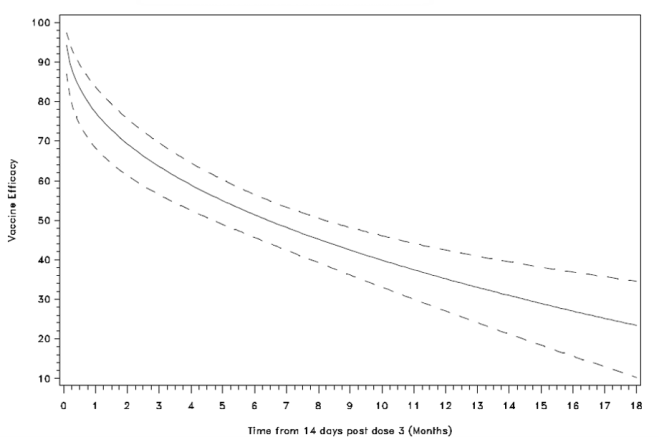 | | | 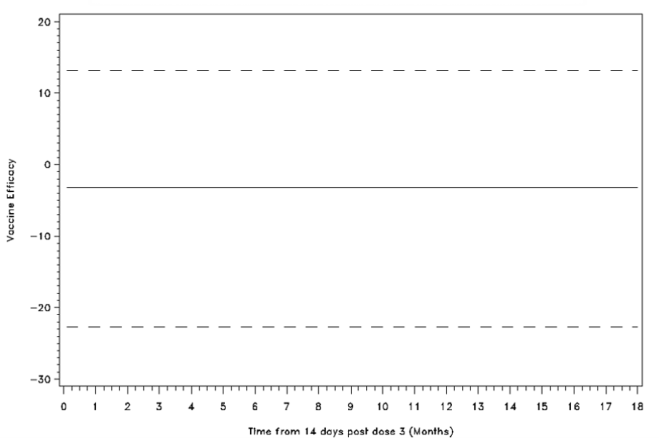 | | |
| **Nanoro - model=group*(log(stop))** | | | **Nanoro - model=group*(stop**(0.5))** | | |
| 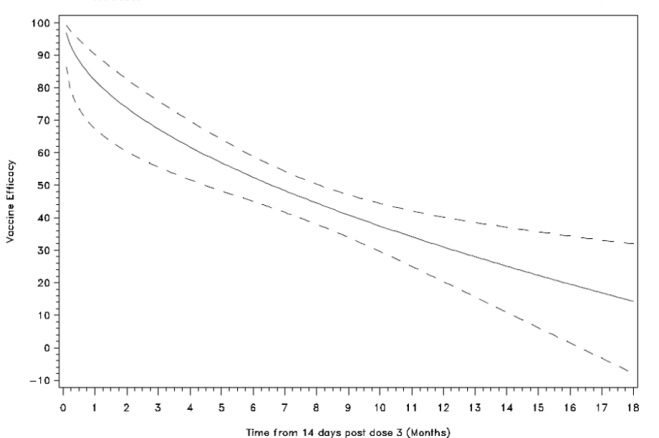 | | | 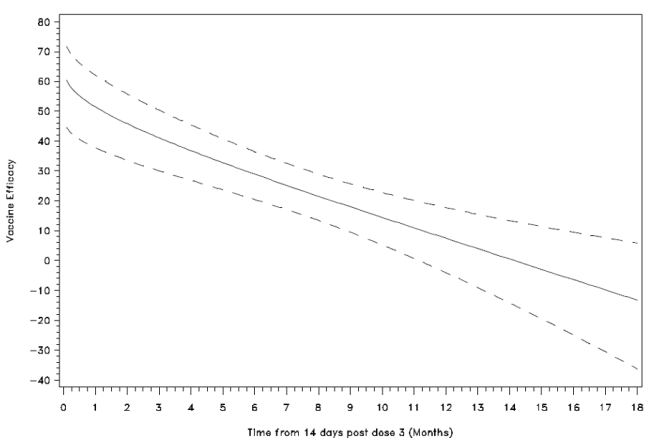 | | |
| *Figure continues on next page* | | |  | | |
| **A.** Children 5-17 months of age at enrollment | | | **B.** Infants 6-12 weeks of age at enrollment | | |
| **Siaya - model=group*(stop**(0.5))** | | | **Siaya - model=group*(log(stop))** | | |
| 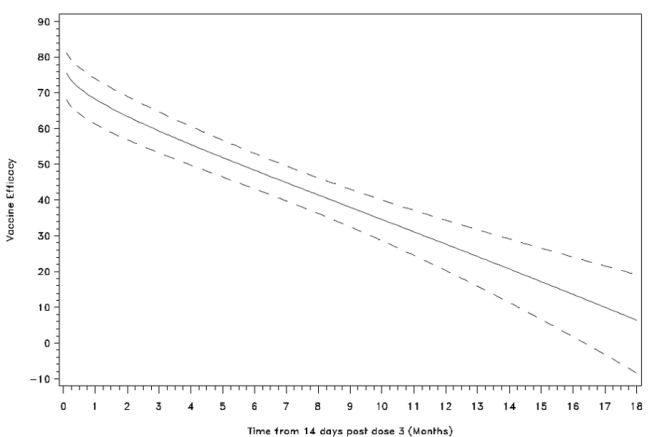 | | | 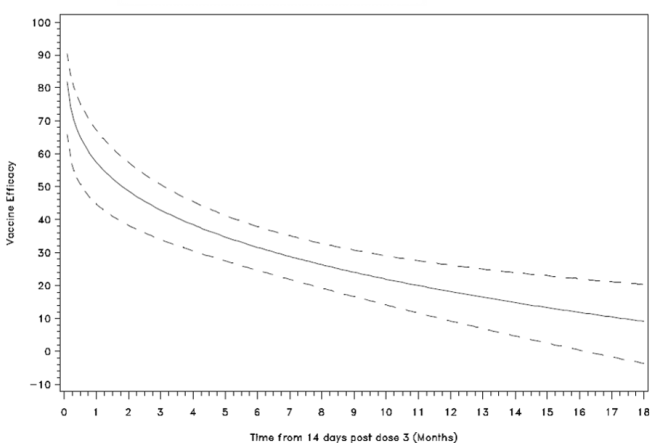 | | |

Note that analyses to detect differences from constant efficacy are dependent on the number of malaria episodes. As a (mathematical) result, low transmission sites are less likely to show waning efficacy.
