# Supplementary material for: Efficacy and Safety of the RTS,S/AS01 Malaria Vaccine during 18 Months after Vaccination: A Phase 3 Randomized, Controlled Trial in Children and Young Infants at 11 African Sites
Source: PLoS Med. 2014 Jul 29;11(7):e1001685. doi: 10.1371/journal.pmed.1001685 (PMC4114488; doi:10.1371/journal.pmed.1001685)
Supplement: Figure S5 — Overall incidence of clinical and severe malaria (primary case definitions) by 6-mo periods (per-protocol and intention-to-treat populations). (DOCX) [file pmed.1001685.s005.docx]

## Supplementary figure 5a. Overall incidence of clinical and severe malaria (primary case definitions) by 6-month periods (per-protocol population)

| **A.** Children 5-17 months of age at enrollment - clinical malaria | **B.** Infants 6-12 weeks of age at enrollment - clinical malaria |
| --- | --- |
|  |  |
| **C.** Children 5-17 months of age at enrollment - severe malaria | **D.** Infants 6-12 weeks of age at enrollment - severe malaria |
|  |  |

The vertical black lines show the 95% confidence interval.

Clinical malaria primary case definition: Illness in a child brought to a study facility with a temperature of ≥ 37.5°C and *P. falciparum* asexual parasitemia at a density of > 5000 parasites per cubic millimeter or a case of malaria meeting the primary case definition of severe malaria.

Severe malaria primary case definition: *P. falciparum* asexual parasitemia at a density of > 5000 parasites per cubic millimeter with one or more markers of disease severity and without diagnosis of a coexisting illness. Markers of severe disease were prostration, respiratory distress, a Blantyre coma score of ≤ 2 (on a scale of 0 to 5, with higher scores indicating a higher level of consciousness), two or more observed or reported seizures, hypoglycemia, acidosis, elevated lactate level, or hemoglobin level of < 5 g per deciliter. Coexisting illnesses were defined as radiographically proven pneumonia, meningitis established by analysis of cerebrospinal fluid, bacteremia, or gastroenteritis with severe dehydration.

Incidence = number of events reported in each group per person year.

[1-6 months] = 14 days post dose-3 until 6 months post dose-3.

[7-12 months] = 6 months post dose-3 until 12 months post dose-3.

[13-18 months] = 12 months post dose-3 until 18 months post dose-3.

## Supplementary figure 5b. Overall incidence of clinical and severe malaria (primary case definitions) by 6-month periods (intention-to-treat population)

| **A.** Children 5-17 months of age at enrollment - clinical malaria | **B.** Infants 6-12 weeks of age at enrollment - clinical malaria |
| --- | --- |
| 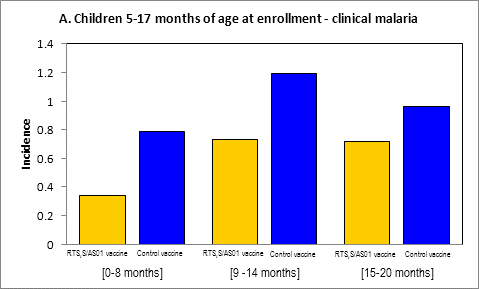 | 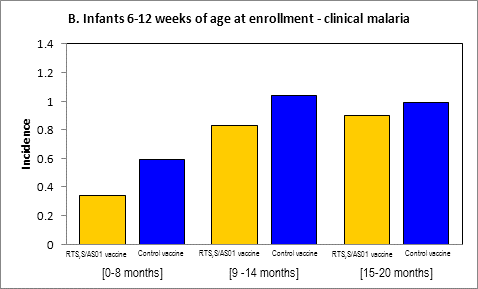 |
| **C.** Children 5-17 months of age at enrollment - severe malaria | **D.** Infants 6-12 weeks of age at enrollment - severe malaria |
| 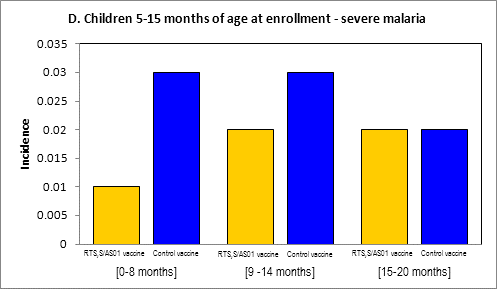 | 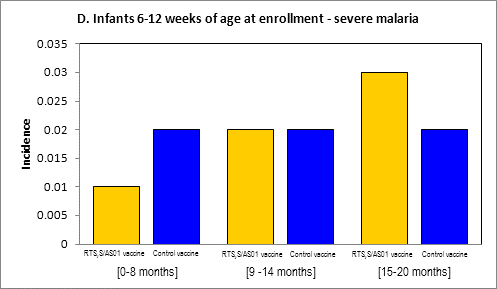 |

The vertical black lines show the 95% confidence interval.

Clinical malaria primary case definition: Illness in a child brought to a study facility with a temperature of ≥ 37.5°C and *P. falciparum* asexual parasitemia at a density of > 5000 parasites per cubic millimeter or a case of malaria meeting the primary case definition of severe malaria.

Severe malaria primary case definition: *P. falciparum* asexual parasitemia at a density of > 5000 parasites per cubic millimeter with one or more markers of disease severity and without diagnosis of a coexisting illness. Markers of severe disease were prostration, respiratory distress, a Blantyre coma score of ≤ 2 (on a scale of 0 to 5, with higher scores indicating a higher level of consciousness), two or more observed or reported seizures, hypoglycemia, acidosis, elevated lactate level, or hemoglobin level of < 5 g per deciliter. Coexisting illnesses were defined as radiographically proven pneumonia, meningitis established by analysis of cerebrospinal fluid, bacteremia, or gastroenteritis with severe dehydration.

Incidence = number of events reported in each group per person year.

[0-8 months] = from dose-1 until 8 months post dose-1.

[9-14 months] = 8 months post dose-1 until 14 months post dose-1.

[15-20 months] = 14 months post dose-1 until 20 months post dose-1.
