# Supplementary material for: Efficacy and Safety of the RTS,S/AS01 Malaria Vaccine during 18 Months after Vaccination: A Phase 3 Randomized, Controlled Trial in Children and Young Infants at 11 African Sites
Source: PLoS Med. 2014 Jul 29;11(7):e1001685. doi: 10.1371/journal.pmed.1001685 (PMC4114488; doi:10.1371/journal.pmed.1001685)
Supplement: Figure S6 — Reduction in incidence of all episodes of clinical malaria (primary case definition) during each 6-mo period ordered by malaria incidence (per-protocol and intention-to-treat populations). (DOCX) [file pmed.1001685.s006.docx]

## Supplementary figure 6a. Reduction in incidence of all episodes of clinical malaria (primary case definition) during each 6-month period ordered by malaria incidence (per-protocol population)

| **A.** Children 5-17 months: [1-6 months] | **B.** Children 5-17 months: [7-12 months] | **C.** Children 5-17 months: [13-18 months] |
| --- | --- | --- |
| 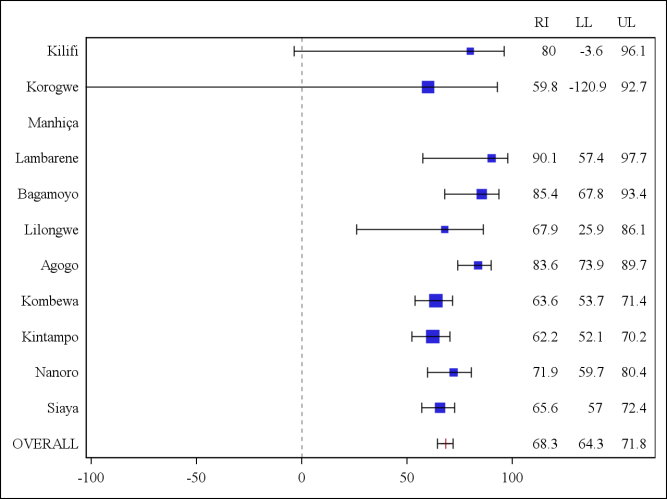 | 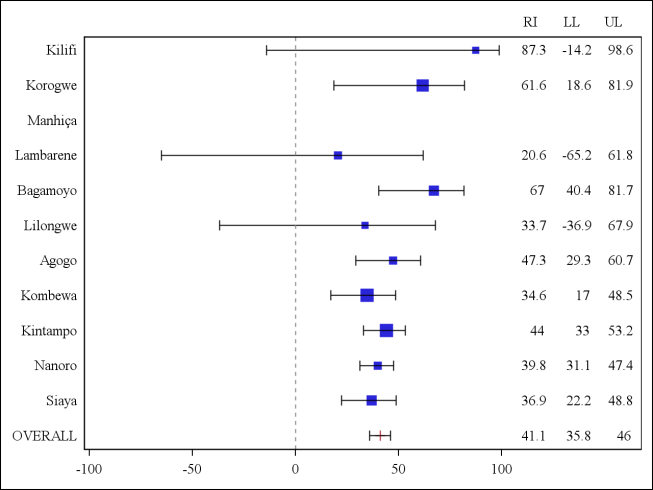 | 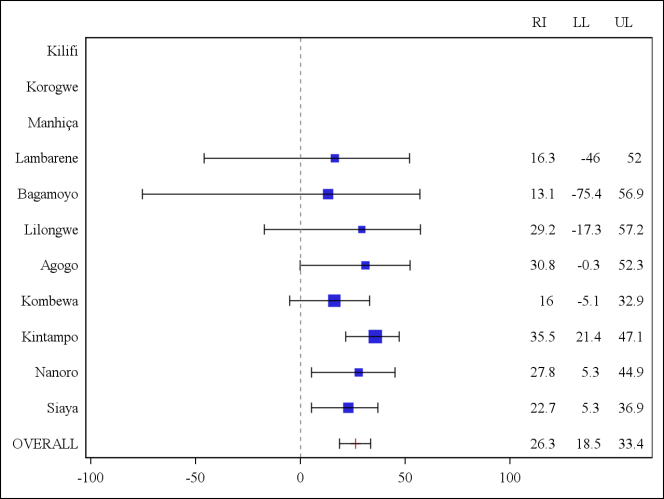 |
| **D.** Infants 6-12 weeks: [1-6 months] | **E.** Infants 6-12 weeks: [7-12 months] | **F**. Infants 6-12 weeks: [13-18 months] |
| 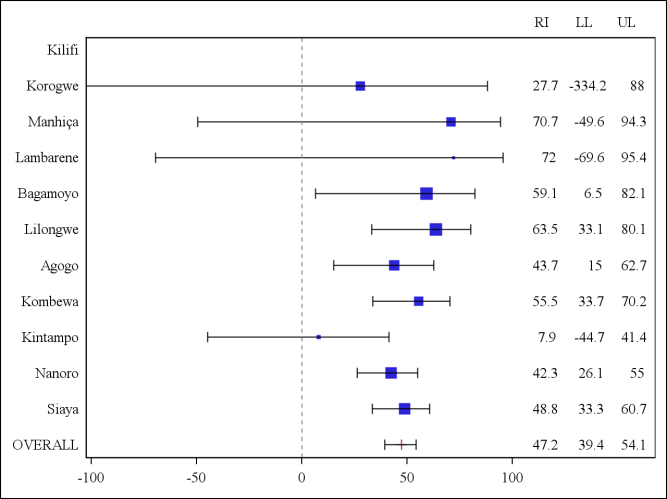 | 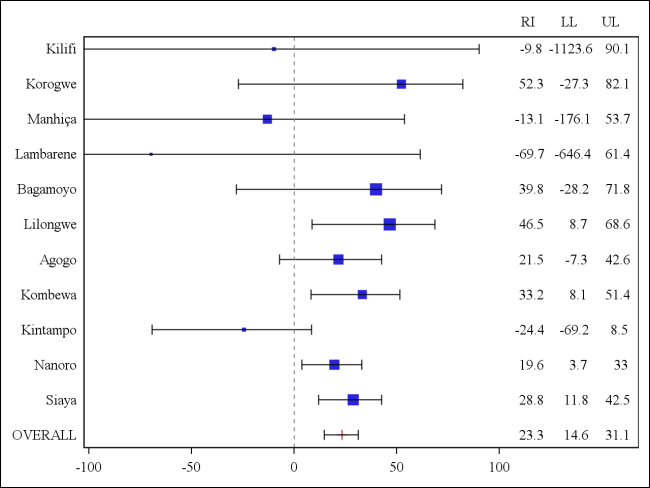 | 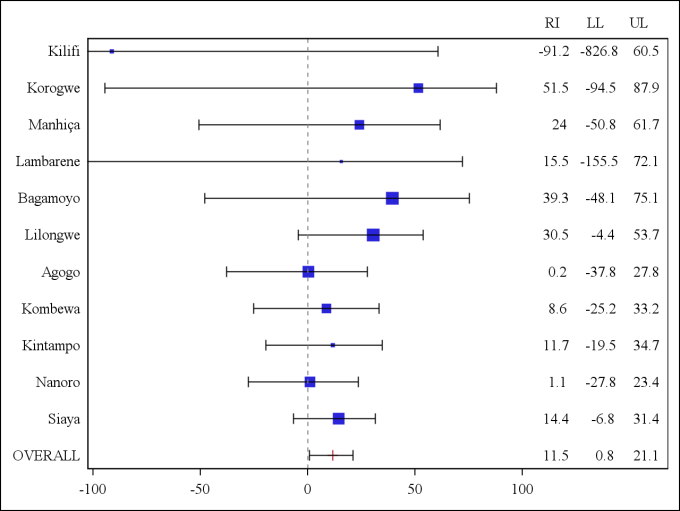 |

Clinical malaria primary case definition: Illness in a child brought to a study facility with a temperature of ≥ 37.5°C and *P. falciparum* asexual parasitemia at a density of > 5000 parasites per cubic millimeter or a case of malaria meeting the primary case definition of severe malaria.

RI: percentage of reduction in incidence of all episodes of clinical malaria (primary case definition) over the 6-month period.

LL = lower limit of the 95% confidence interval.

UL = Upper limit of the 95% confidence interval.

[1-6 months] = 14 days post dose-3 until 6 months post dose-3.

[7-12 months] = 6 months post dose-3 until 12 months post dose-3.

[13-18 months] = 12 months post dose-3 until 18 months post dose-3.

The size of each blue square reflects the relative number of subjects enrolled at each study site. The horizontal bars show the lower limit and upper limit of the 95% confidence interval.

Study sites are ordered from lowest (Kilifi) to highest (Siaya) incidence of clinical malaria, defined as a measured or reported fever within previous 24h and parasite density >0 parasites per cubic millimeter (i.e. clinical malaria secondary case definition), measured in control infants 6-12 weeks of age at enrollment during 12 months of follow-up.

The incidence of clinical malaria (total number of episodes/person year) and the reduction in incidence was calculated by 6-monthly periods and presented with 95% CIs. These results are expressed as a reduction in incidence and not a vaccine efficacy because the randomization is lost when dividing the analysis by 6-month breakdown. Children who begin the analysis at 6 months or 12 months post dose-3 are not the same group of children than those who were randomized at month zero.

## Supplementary figure 6b. Reduction in incidence of all episodes of clinical malaria (primary case definition) during each 6-month period ordered by malaria incidence (intention-to-treat population)

| **A.** Children 5-17 months: [0-8 months] | **B.** Children 5-17 months: [9-14 months] | **C.** Children 5-17 months: [15-20 months] |
| --- | --- | --- |
| 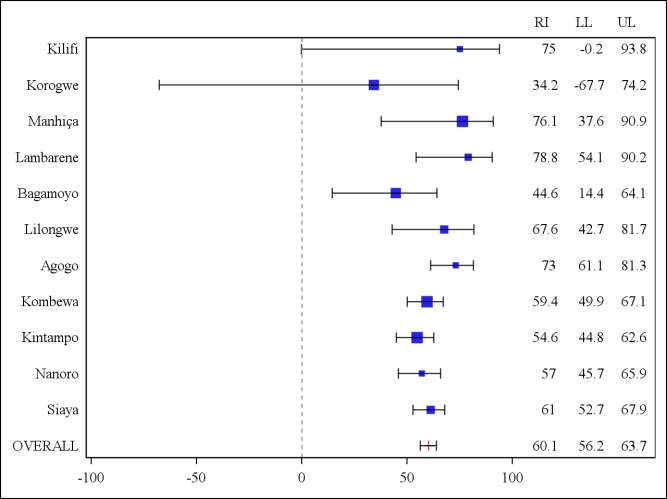 | 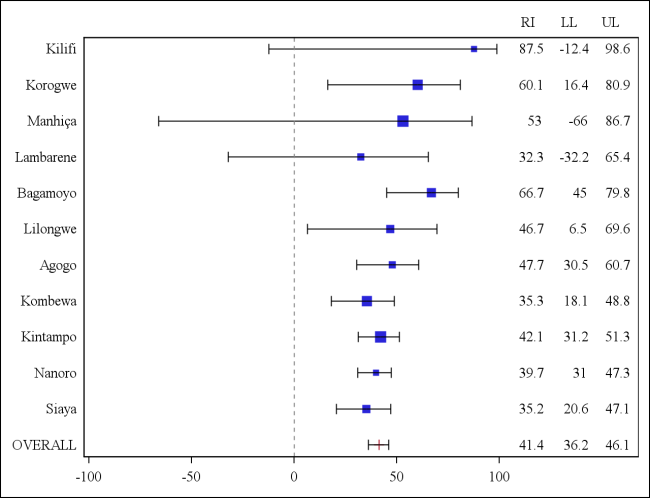 | 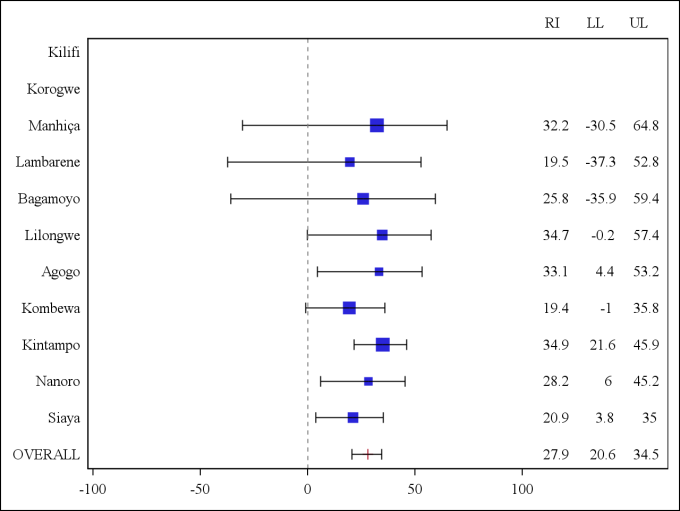 |
| **D.** Infants 6-12 weeks: [0-8 months] | **E.** Infants 6-12 weeks: [9-14 months] | **F**. Infants 6-12 weeks: [15-20 months] |
| 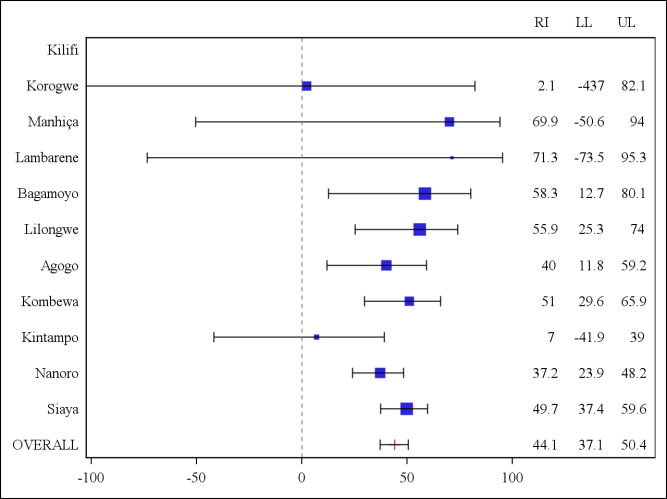 | 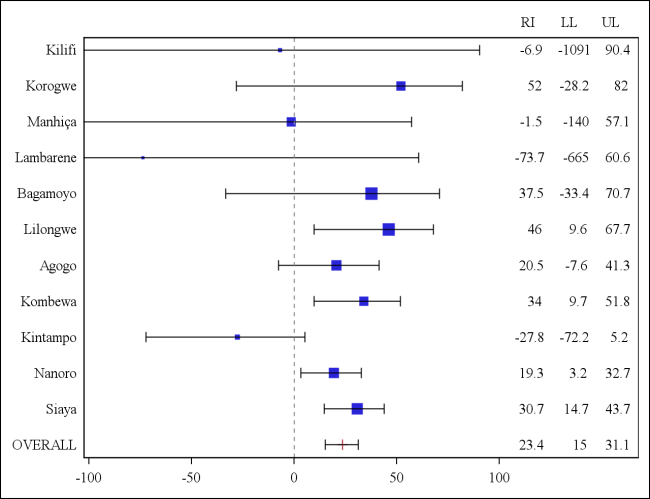 | 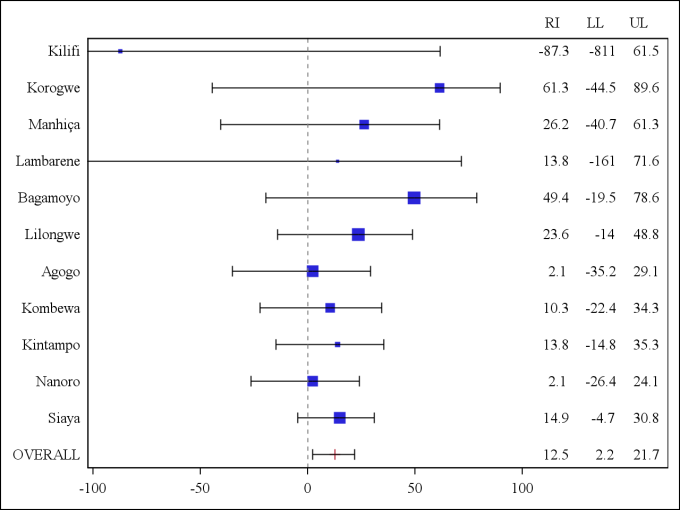 |

Clinical malaria primary case definition: Illness in a child brought to a study facility with a temperature of ≥ 37.5°C and *P. falciparum* asexual parasitemia at a density of > 5000 parasites per cubic millimeter or a case of malaria meeting the primary case definition of severe malaria.

RI: percentage of reduction in incidence of all episodes of clinical malaria (primary case definition) over the 6-month period.

LL = lower limit of the 95% confidence interval.

UL = Upper limit of the 95% confidence interval.

[0-8 months] = from dose-1 until 8 months post dose-1.

[9-14 months] = 8 months post dose-1 until 14 months post dose-1.

[15-20 months] = 14 months post dose-1 until 20 months post dose-1.

The size of each blue square reflects the relative number of subjects enrolled at each study site. The horizontal bars show the lower limit and upper limit of the 95% confidence interval.

Study sites are ordered from lowest (Kilifi) to highest (Siaya) incidence of clinical malaria, defined as a measured or reported fever within previous 24h and parasite density >0 parasites per cubic millimeter (i.e. clinical malaria secondary case definition), measured in control infants 6-12 weeks of age at enrollment during 12 months of follow-up.

The incidence of clinical malaria (total number of episodes/person year) and the reduction in incidence was calculated by 6-monthly periods and presented with 95% CIs. These results are expressed as a reduction in incidence and not a vaccine efficacy because the randomization is lost when dividing the analysis by 6-month breakdown. Children who begin the analysis at 6 months or 12 months post dose-3 are not the same group of children than those who were randomized at month zero.
