# Supplementary material for: Efficacy and Safety of the RTS,S/AS01 Malaria Vaccine during 18 Months after Vaccination: A Phase 3 Randomized, Controlled Trial in Children and Young Infants at 11 African Sites
Source: PLoS Med. 2014 Jul 29;11(7):e1001685. doi: 10.1371/journal.pmed.1001685 (PMC4114488; doi:10.1371/journal.pmed.1001685)
Supplement: Figure S7 — Overall survival curves (intention-to-treat population). (DOCX) [file pmed.1001685.s007.docx]

## Supplementary figure 7. Overall survival curves (intention-to-treat population)

| **A.** Children 5-17 months of age at enrollment | **B.** Infants 6-12 weeks of age at enrollment |
| --- | --- |
|  |  |
| R3R+R3C = group receiving RTS,S/AS01 primary schedule.  C3C = Control group.  All-cause mortality (case definition 1): A fatality of any cause (includes mortality in the community and in hospital). | |
