# Supplementary material for: Efficacy and Safety of the RTS,S/AS01 Malaria Vaccine during 18 Months after Vaccination: A Phase 3 Randomized, Controlled Trial in Children and Young Infants at 11 African Sites
Source: PLoS Med. 2014 Jul 29;11(7):e1001685. doi: 10.1371/journal.pmed.1001685 (PMC4114488; doi:10.1371/journal.pmed.1001685)
Supplement: Figure S8 — Vaccine efficacy against all episodes of clinical malaria (primary case definition) during a 12-mo follow-up period after dose 3 ordered by increasing malaria incidence. (DOCX) [file pmed.1001685.s008.docx]

## Supplementary figure 8. Vaccine efficacy against all episodes of clinical malaria (primary case definition) during a 12-month follow-up period post dose-3 ordered by increasing malaria incidence

| **A.** Children 5-17 months of age at enrollment (per-protocol population) | **B.** Infants 6-12 weeks of age at enrollment (per-protocol population) |
| --- | --- |
| 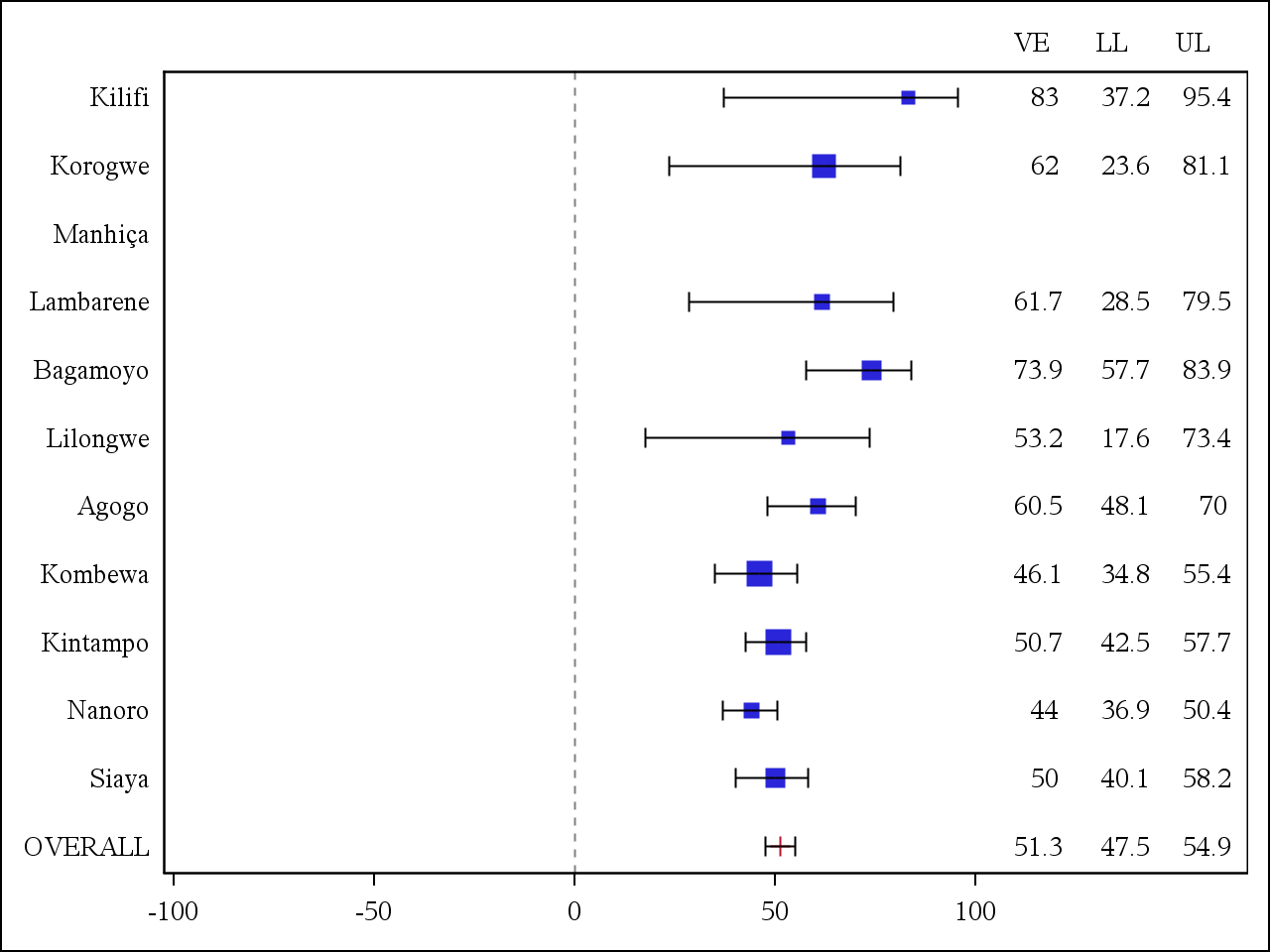 | 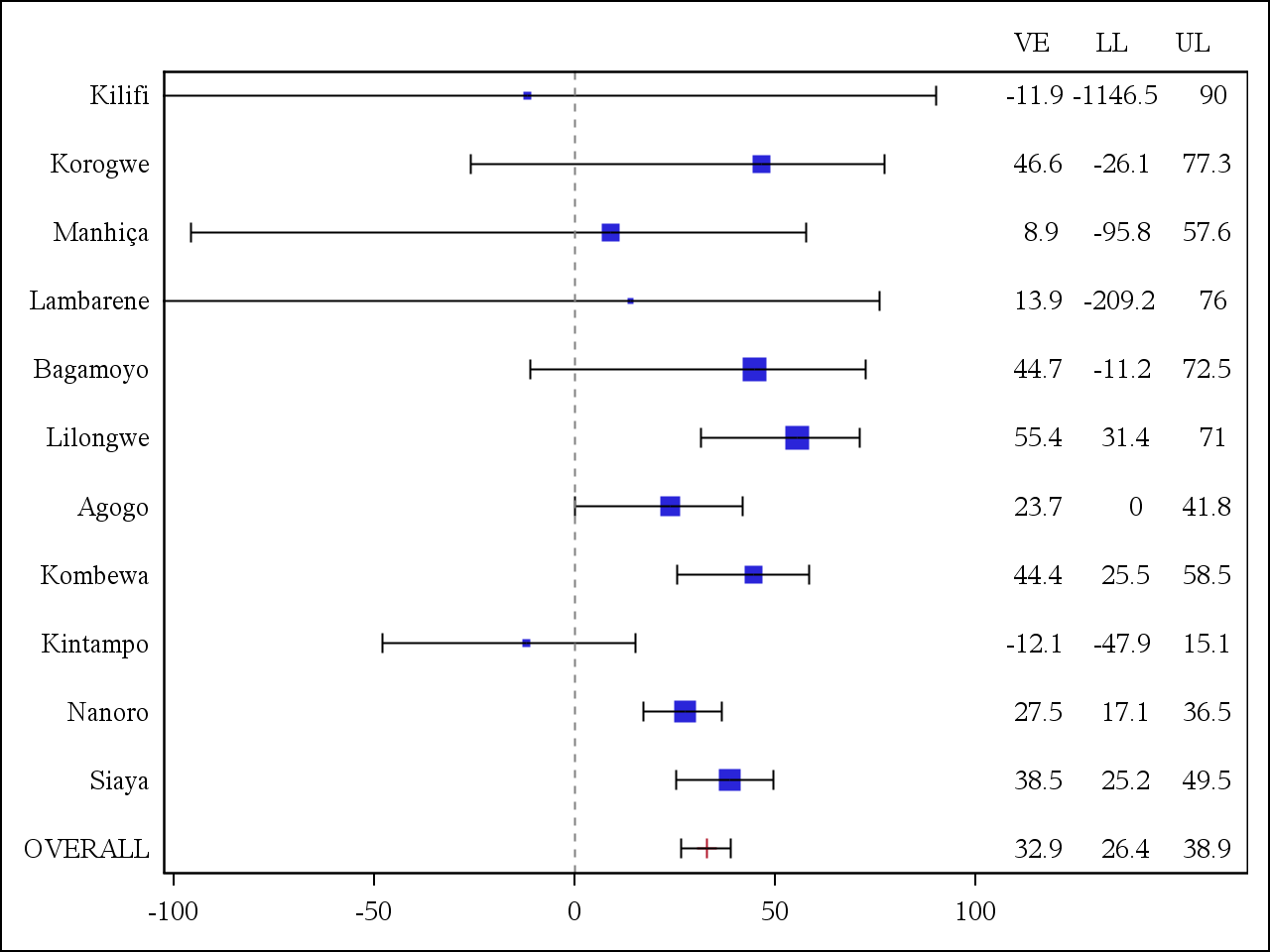 |
| **C.** Children 5-17 months of age at enrollment (intention-to-treat population) | **D.** Infants 6-12 weeks of age at enrollment (intention-to-treat population) |
| 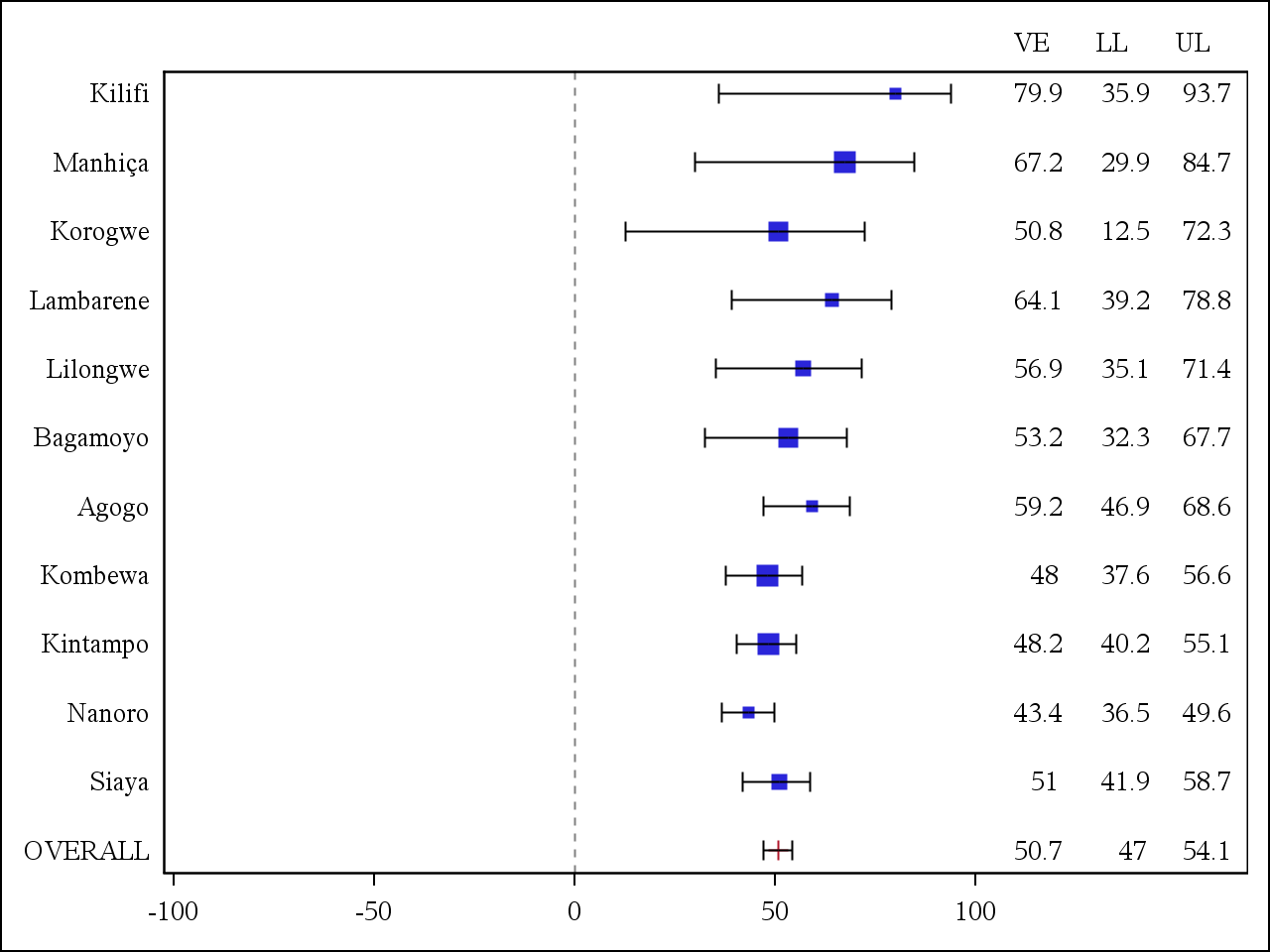 | 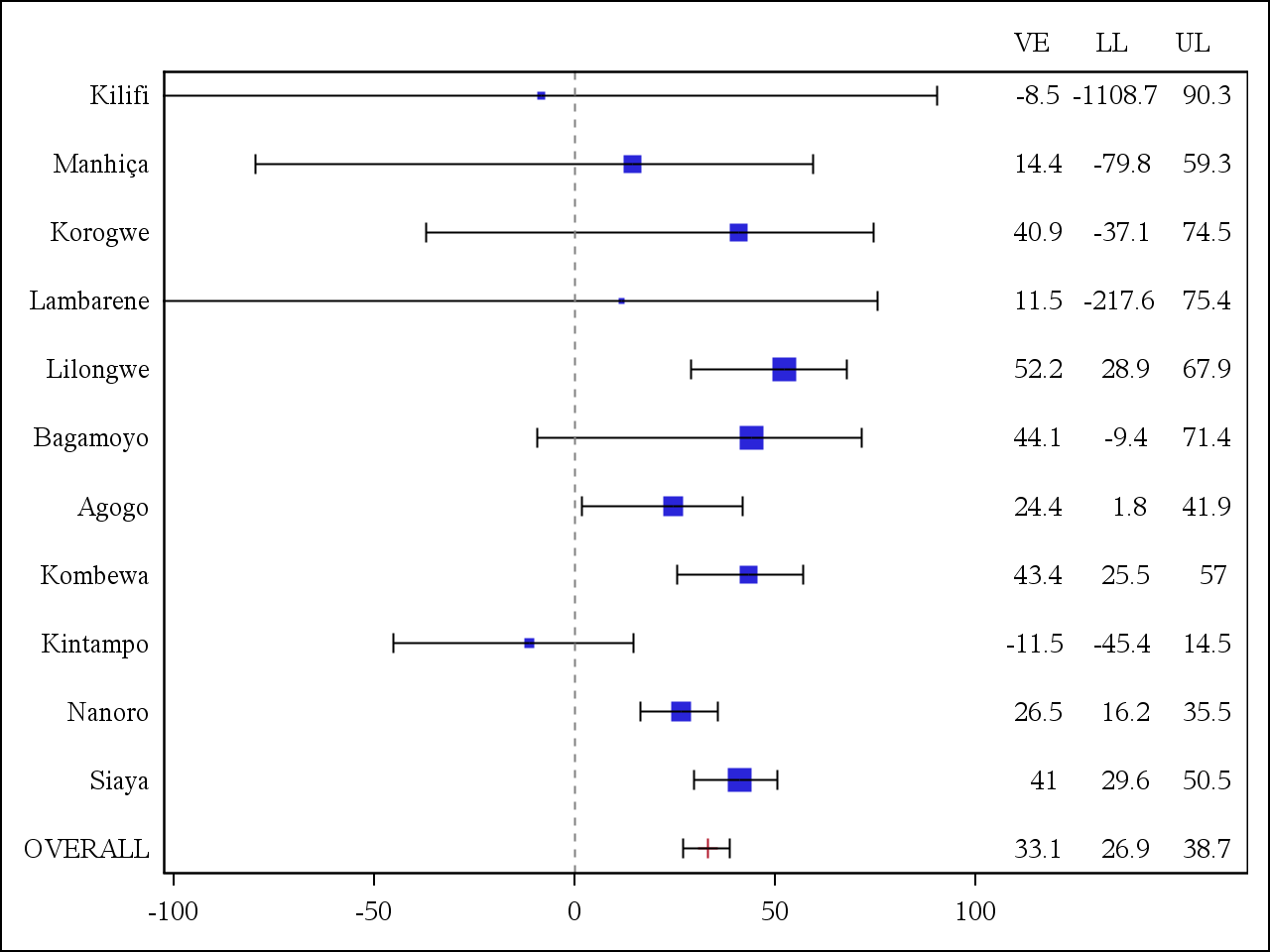 |
| VE = Vaccine efficacy against all episodes of clinical malaria primary case definition unadjusted for covariates.  Clinical malaria primary case definition: Illness in a child brought to a study facility with a temperature of ≥ 37.5°C and *P. falciparum* asexual parasitemia at a density of > 5000 parasites per cubic millimeter or a case of malaria meeting the primary case definition of severe malaria.  LL = lower limit of the 95% confidence interval.  UL = Upper limit of the 95% confidence interval.  The size of each blue square reflects the relative number of subjects enrolled at each study site. The horizontal bars show the lower limit and upper limit of the 95% confidence interval.  Study sites are ordered from lowest (Kilifi) to highest (Siaya) incidence of clinical malaria, defined as a measured or reported fever within previous 24h and parasite density >0 parasites per cubic millimeter (i.e. clinical malaria secondary case definition), measured in control infants 6-12 weeks of age at enrollment during 12 months of follow-up. | |
