# Supplementary material for: Efficacy and Safety of the RTS,S/AS01 Malaria Vaccine during 18 Months after Vaccination: A Phase 3 Randomized, Controlled Trial in Children and Young Infants at 11 African Sites
Source: PLoS Med. 2014 Jul 29;11(7):e1001685. doi: 10.1371/journal.pmed.1001685 (PMC4114488; doi:10.1371/journal.pmed.1001685)
Supplement: Figure S9 — Time-to-onset distribution of the meningitis cases after dose 1, dose 2, and dose 3 within 600 d post-vaccination for both age categories (intention-to-treat population). (DOCX) [file pmed.1001685.s009.docx]

## Supplementary figure 9a. Time-to-onset distribution of the meningitis cases post dose-1, dose-2 and dose-3 within 600 days post vaccination for both age categories (intention-to-treat population)

In total 29 cases of meningitis were reported (17 cases in children 5-17 months and 12 cases in infants 6-12 weeks). Among the 29 events, 26 cases occurred after dose-2 and 23 cases occurred after dose-3.

## Supplementary figure 9b. Meningitis cases distribution by study site and etiology during 20 months post dose-1(blinded) in both age categories (intention-to-treat population)


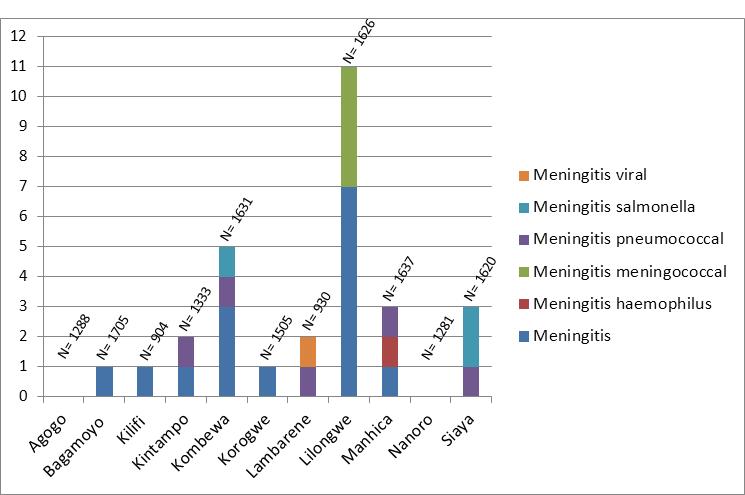


N= total number of subjects (6-12 weeks + 5-17 months) enrolled at a particular site.
