# Supplementary material for: Efficacy and Safety of the RTS,S/AS01 Malaria Vaccine during 18 Months after Vaccination: A Phase 3 Randomized, Controlled Trial in Children and Young Infants at 11 African Sites
Source: PLoS Med. 2014 Jul 29;11(7):e1001685. doi: 10.1371/journal.pmed.1001685 (PMC4114488; doi:10.1371/journal.pmed.1001685)
Supplement: Table S1 — List of ethics committees and review boards, and investigational centers and affiliated partners. (DOCX) [file pmed.1001685.s010.docx]

## Supplementary table 1a. List of Ethics Committees and Review Boards

| **Study Sites** | **Ethics Review Body** |
| --- | --- |
| Institut de Recherche en Science de la Santé, Nanoro, Burkina Faso | Western Institutional Review Board (WIRB) |
|  | Comité d’Ethique Institutionnel du Centre Muraz (Institutional Ethics Committee of Muraz Center) |
|  | Comite d’Ethique pour la Recherche en Santé (Ethics Committee for Health Research) |
| Albert Schweitzer Hospital, Lambarene, Gabon | Western Institutional Review Board (WIRB) |
|  | Comité d’Ethique Régional Indépendant de Lambaréné (CERIL)  (Independent Regional Ethics Committee of Lambaréné) |
|  | Comité National d’Ethique pour la Recherche (National Ethics Committee for Research  The Board) |
| School of Medical Sciences, Kumasi (Agogo), Ghana | Western Institutional Review Board (WIRB) |
|  | Ghana Health Service (GHS) Ethical Review Committee (ERC)  Research and Development Division |
|  | Committee on Human Research Publication and Ethics (CHRPE) |
| Kintampo Health Research Center, Kintampo, Ghana | Western Institutional Review Board (WIRB) |
|  | Kintampo Health Research Centre (KHRC) Institutional Ethics Committee (IEC) |
|  | London School of Hygiene and Tropical Medicine Research Ethics Committee |
|  | Ghana Health Service (GHS) Ethical Review Committee (ERC) Research and Development Division |
| KEMRI - Walter Reed Project, Kombewa, Kenya | Western Institutional Review Board (WIRB) |
|  | Kenya Medical Research Institute (KEMRI) National Ethics Review Committee |
|  | Walter Reed Army Institute of Research (WRAIR) IRB |
| KEMRI - Wellcome Trust Research Program, Kilifi, Kenya | Western Institutional Review Board (WIRB) |
|  | Kenya Medical Research Institute (KEMRI) National Ethics Review Committee |
| KEMRI/CDC Research and Public Health Collaboration, Siaya, Kenya | Western Institutional Review Board (WIRB) |
|  | Kenya Medical Research Institute (KEMRI) National Ethics Review Committee |
|  | Centers for Disease Control and Prevention(CDC) – IRB |
| University of North Carolina Project, Lilongwe, Malawi | Western Institutional Review Board (WIRB) |
|  | National Health Sciences Research Committee |
|  | Office of Human Research Ethics |
| Centro de Investigação em Saúde de Manhiça, Manhiça, Mozambique | Western Institutional Review Board (WIRB) |
|  | Comitè Etic Investigació Clinica (Hospital Clinic (Barcelona University) Ethics Committee) |
|  | Comité Nacional de Bioética para a Saúde (National Bioethical Health Committee, Mozambique) |
| Ifakara Health Institute, Bagamoyo, Tanzania | Western Institutional Review Board (WIRB) |
|  | Tanzanian Medical Research Coordinating Committee (MRCC) operating within the National Institute for Medical Research (NIMR) |
|  | Ethikkommission beider Basel (EKBB)  (Ethics Committee of the local government responsible for the Swiss Tropical and public Health Institute and the University of Basel, Switzerland) |
|  | Ifakara Health Institute IRB |
| National Institute for Medical Research, Korogwe, Tanzania | Western Institutional Review Board (WIRB) |
|  | London School of Hygiene and Tropical Medicine Research Ethics Committee |
|  | Tanzania Medical Research Coordinating Committee (MRCC) operating within National Institute for Medical Research (NIMR) |
|  | The Danish National Committee on Biomedical Research Ethics |

## Supplementary table 1b. Investigational centers and affiliated partners

| **Country** | **Investigational center** | **Abbreviated name** | **Affiliated partner** |
| --- | --- | --- | --- |
| Burkina Faso | Institut de Recherche en Science de la Santé | Nanoro | Prince Leopold Institute of Tropical Medicine, Belgium |
| Gabon | Albert Schweitzer Hospital, Medical Research Unit | Lambaréné | University of Tübingen, Germany |
| Ghana | Kwame Nkrumah University of Science and Technology, School of Medical Sciences, Kumasi | Agogo |  |
| Ghana | Kintampo Health Research Centre | Kintampo | London School of Hygiene and Tropical Medicine, UK |
| Kenya | KEMRI - Wellcome Trust Research Program | Kilifi | University of Oxford, UK |
| Kenya | KEMRI - Walter Reed Project | Kombewa | Walter Reed Army Institute of Research, USA |
| Kenya | KEMRI/CDC Research and Public Health Collaboration | Siaya | US Centers for Disease Control and Prevention, USA |
| Malawi | University of North Carolina Project | Lilongwe | University of North Carolina at Chapel Hill, USA |
| Mozambique | Centro de Investigação em Saúde de Manhiça | Manhiça | Barcelona Centre for International Health Research (CRESIB), Hospital Clinic - Universitat de Barcelona |
| Tanzania | Ifakara Health Institute (IHI), Bagamoyo Branch | Bagamoyo | Swiss Tropical and Public Health Institute, Switzerland |
| Tanzania | National Institute for Medical Research, Korogwe Branch | Korogwe | London School of Hygiene and Tropical Medicine, UK  Center for Medical Parasitology at University of Copenhagen and Copenhagen University Hospital, Denmark  Kilimanjaro Christian Medical College, Tanzania |
