# Supplementary material for: Efficacy and Safety of the RTS,S/AS01 Malaria Vaccine during 18 Months after Vaccination: A Phase 3 Randomized, Controlled Trial in Children and Young Infants at 11 African Sites
Source: PLoS Med. 2014 Jul 29;11(7):e1001685. doi: 10.1371/journal.pmed.1001685 (PMC4114488; doi:10.1371/journal.pmed.1001685)
Supplement: Table S2 — Case definitions of severe malaria and algorithm for the evaluation of a hospital admission as a potential case of severe malaria. (DOCX) [file pmed.1001685.s011.docx]

## Supplementary table 2a. Case definitions of severe malaria

| **Primary definition** | *P. falciparum* > 5000 parasites per mm^3^ | **AND** one or more marker of disease severity:   - Prostration - Respiratory distress - Blantyre score ≤ 2 - Seizures 2 or more - Hypoglycemia < 2.2 mmol/L - Acidosis BE ≤-10.0 mmol/L - Lactate ≥ 5.0 mmol/L - Anemia < 5.0 g/dL   **AND** without diagnosis of a co-morbidity:   - Radiographically proven pneumonia - Meningitis on CSF examination - Positive blood culture - Gastroenteritis with dehydration |
| --- | --- | --- |
| **Secondary definition**  without excluding co-morbidity | *P. falciparum* > 5000 parasites per mm^3^ | **AND** one or more marker of disease severity |

**Prostration:** in an acutely sick child, the inability to perform previously-acquired motor function: in a child previously able to stand, inability to stand; in a child previously able to sit, inability to sit and in a very young child, inability to suck.

**Respiratory distress:** lower chest wall indrawing or abnormally deep breathing.

**2 or more seizures:** occurring in the total time period including 24 hours prior to admission time in the emergency room and during hospitalization.

**Radiographically proven pneumonia:** a consolidation or pleural effusion defined per protocol on a chest x-ray taken within 72 hours of admission.

**Meningitis on CSF examination:** WC ≥ 50 x10 ^6^/L or positive culture of compatible organism or latex agglutination test positive for Hib, pneumococci or meningococci.

**Gastroenteritis with dehydration:** history of 3 or more loose or watery stools in previous 24 hours, an observed watery stool and decreased skin turgor (> 2 seconds for skin to return following skin pinch).

**Positive blood culture:** defined per protocol on a blood culture taken within 72 hours of admission.

## Supplementary table 2b. Algorithm for the evaluation of a hospital admission as a potential case of severe malaria

| For all acute hospital admissions (i.e. except planned admissions for medical investigation/care or elective surgery or trauma admissions), a blood sample was taken for evaluation of: | |
| --- | --- |
|  | Malaria parasite density |
|  | Blood culture |
|  | Hemoglobin |
|  | Blood glucose, lactate and base excess |
| **Lumbar Puncture was indicated by the presence of:** | |
|  | Seizure except simple febrile seizure (defined as a seizure associated with fever, which lasts for 5 minutes or less, generalized as opposed to focal, not followed by transient or persistent neurological abnormalities, occurring in a child ≥ 6 months of age, with full recovery within 1 hour) |
|  | Blantyre Coma Score < 5 (children ≤ 9 months of age < 4 [in association with best motor response of 1])^1^ |
|  | Prostration in child < 3 year of age |
|  | Meningism/stiff neck/bulging fontanelle |
|  | Clinician’s judgment |
| **Chest X-ray (CXR) was indicated by the presence of:** | |
|  | Tachypnea (≥50 breaths per minute in a child < 1 year and ≥ 40 breaths per minute in a child ≥ 1 year)^2^ |
|  | Lower chest wall indrawing |
|  | Abnormally deep breathing |
|  | Clinician’s judgment |

1. Molyneux ME, Taylor TE, Wirima JJ, Borgstein A. Clinical features and prognostic indicators in paediatric cerebral malaria: a study of 131 comatose Malawian children. Q J Med 1989;71:441-59.
2. Berkley JA, Ross A, Mwangi I et al. Prognostic indicators of early and late death in children admitted to district hospital in Kenya: cohort study. BMJ 2003;326:361-366.
