# Supplementary material for: Efficacy and Safety of the RTS,S/AS01 Malaria Vaccine during 18 Months after Vaccination: A Phase 3 Randomized, Controlled Trial in Children and Young Infants at 11 African Sites
Source: PLoS Med. 2014 Jul 29;11(7):e1001685. doi: 10.1371/journal.pmed.1001685 (PMC4114488; doi:10.1371/journal.pmed.1001685)
Supplement: Table S3 — Number of children and infants enrolled at each study site and number of participants contributing to the per-protocol population, ordered by increasing malaria incidence. (DOCX) [file pmed.1001685.s012.docx]

## Supplementary table 3. Number of children and infants enrolled at each study site and number of participants contributing to the per-protocol population, ordered by increasing malaria incidence

|  | **Children 5-17 months of age at enrollment** | | | | **Infants 6-12 weeks of age at enrollment** | | | |
| --- | --- | --- | --- | --- | --- | --- | --- | --- |
|  | **Number enrolled** | | **Per-protocol population** | | **Number enrolled** | | **Per-protocol population** | |
| **Study site** | **RTS,S/AS01 vaccine (%)** | **Control vaccine (%)** | **RTS,S/AS01 vaccine (%)** | **Control vaccine (%)** | **RTS,S/AS01 vaccine (%)** | **Control vaccine (%)** | **RTS,S/AS01 vaccine (%)** | **Control vaccine (%)** |
| Kilifi | 398 (6.7) | 202 (6.8) | 336 (7.4) | 171 (7.3) | 199 (4.6) | 105 (4.8) | 186 (4.7) | 102 (5.1) |
| Korogwe | 608 (10.2) | 304 (10.2) | 568 (12.5) | 293 (12.6) | 398 (9.1) | 195 (8.9) | 382 (9.6) | 183 (9.1) |
| Manhiça | 664 (11.2) | 338 (11.4) | - | - | 423 (9.7) | 212 (9.7) | 381 (9.5) | 188 (9.4) |
| Lambarene | 470 (7.9) | 234 (7.9) | 380 (8.3) | 196 (8.4) | 158 (3.6) | 68 (3.1) | 147 (3.7) | 62 (3.1) |
| Bagamoyo | 605 (10.2) | 298 (10.0) | 462 (10.1) | 235 (10.1) | 533 (12.2) | 269 (12.3) | 502 (12.6) | 244 (12.2) |
| Lilongwe | 539 (9.1) | 261 (8.8) | 359 (7.9) | 183 (7.9) | 547 (12.6) | 279 (12.8) | 500 (12.5) | 258 (12.9) |
| Agogo | 400 (6.7) | 200 (6.7) | 371 (8.1) | 192 (8.2) | 458 (10.5) | 230 (10.6) | 418 (10.5) | 221 (11.0) |
| Kombewa | 668 (11.2) | 332 (11.2) | 609 (13.4) | 311 (13.4) | 421 (9.7) | 210 (9.6) | 387 (9.7) | 196 (9.8) |
| Kintampo | 668 (11.2) | 334 (11.2) | 602 (13.2) | 296 (12.7) | 221 (5.1) | 110 (5.0) | 199 (5.0) | 99 (4.9) |
| Nanoro | 397 (6.7) | 203 (6.8) | 389 (8.5) | 198 (8.5) | 453 (10.4) | 228 (10.5) | 441 (11.0) | 225 (11.2) |
| Siaya | 532 (8.9) | 268 (9.0) | 481 (10.6) | 253 (10.9) | 547 (12.6) | 273 (12.5) | 453 (11.3) | 229 (11.4) |
| **Overall** | **5949** | **2974** | **4557** | **2328** | **4358** | **2179** | **3996** | **2007** |

A deviation pertaining to study vaccine exposure to temperatures outside recommended ranges resulted in the exclusion from the per-protocol population of all children 5-17 months old enrolled in Manhiça. This deviation was reported previously in: The RTS,S Clinical Trials Partnership. First Results of a Phase 3 Trial of RTS,S/AS01 Malaria Vaccine in African Children. N Engl J Med 2011;365:1863-1875.
