# Supplementary material for: Efficacy and Safety of the RTS,S/AS01 Malaria Vaccine during 18 Months after Vaccination: A Phase 3 Randomized, Controlled Trial in Children and Young Infants at 11 African Sites
Source: PLoS Med. 2014 Jul 29;11(7):e1001685. doi: 10.1371/journal.pmed.1001685 (PMC4114488; doi:10.1371/journal.pmed.1001685)
Supplement: Table S4 — Incidence of clinical malaria (secondary case definition) in the 6–12-wk age category during a 12-mo follow-up period after dose 3, ordered by increasing malaria incidence. (DOCX) [file pmed.1001685.s013.docx]

## Supplementary table 4. Incidence of clinical malaria (secondary case definition) in the 6-12 weeks age category during a 12-month follow-up period post dose-3 ordered by increasing malaria incidence

|  | **Control vaccine** | | | |
| --- | --- | --- | --- | --- |
| **All episodes of clinical malaria secondary case definition (per-protocol population)** | **Number of children (N)** | **Number of episodes (n)** | **Person years at risk (T)** | **n/T** |
| Kilifi | 102 | 3 | 95.9 | 0.03 |
| Korogwe | 183 | 16 | 170.8 | 0.09 |
| Manhiça | 188 | 22 | 175.4 | 0.13 |
| Lambarene | 62 | 11 | 57.1 | 0.19 |
| Bagamoyo | 244 | 47 | 227.9 | 0.21 |
| Lilongwe | 258 | 149 | 210.6 | 0.71 |
| Agogo | 221 | 298 | 209.9 | 1.42 |
| Kombewa | 196 | 372 | 166.5 | 2.23 |
| Kintampo | 99 | 194 | 84.7 | 2.29 |
| Nanoro | 225 | 605 | 182.2 | 3.32 |
| Siaya | 229 | 749 | 175.3 | 4.27 |
| **Overall** | **2007** | **2466** | **1756.2** | **1.40** |
| **All episodes of clinical malaria secondary case definition (intention-to-treat population)** | **N** | **n** | **T (year)** | **n/T** |
| Kilifi | 105 | 3 | 116.7 | 0.03 |
| Korogwe | 195 | 16 | 220.6 | 0.07 |
| Manhiça | 212 | 27 | 237.2 | 0.11 |
| Lambarene | 68 | 14 | 71.2 | 0.2 |
| Bagamoyo | 269 | 53 | 296.9 | 0.18 |
| Lilongwe | 279 | 164 | 283.2 | 0.58 |
| Agogo | 230 | 325 | 263.7 | 1.23 |
| Kombewa | 210 | 424 | 213.1 | 1.99 |
| Kintampo | 110 | 227 | 114.6 | 1.98 |
| Nanoro | 228 | 693 | 231.4 | 2.99 |
| Siaya | 273 | 945 | 253.6 | 3.73 |
| **Overall** | **2179** | **2891** | **2302.2** | **1.26** |

The incidence of clinical malaria meeting the secondary case definition in infants in the control group during 12 months of follow-up was used to categorize malaria incidence across study sites. For all tables and figures reported here, study sites are presented from the lowest to the highest incidence of clinical malaria.

Clinical malaria secondary case definition: Illness in a child brought to a study facility with a measured (temperature of ≥37.5°C) or reported fever within the last 24 hours and *P. falciparum* asexual parasitemia at a density of > 0 parasites per cubic millimeter.

N = number of subjects included in each group.

n = number of episodes included in each group.

T(year) = person years at risk.

n/T = person year rate in each group.
