# Supplementary material for: Efficacy and Safety of the RTS,S/AS01 Malaria Vaccine during 18 Months after Vaccination: A Phase 3 Randomized, Controlled Trial in Children and Young Infants at 11 African Sites
Source: PLoS Med. 2014 Jul 29;11(7):e1001685. doi: 10.1371/journal.pmed.1001685 (PMC4114488; doi:10.1371/journal.pmed.1001685)
Supplement: Table S5 — Vaccine efficacy against all episodes of clinical malaria (primary and secondary case definitions) during an 18-mo follow-up period after dose 3 in the 5–17-mo and 6–12-wk age categories, ordered by increasing malaria incidence. (DOCX) [file pmed.1001685.s014.docx]

## Supplementary table 5a. Vaccine efficacy against all episodes of clinical malaria (primary and secondary case definitions) during a 18-month follow-up period post dose-3 in the 5-17 months age category, ordered by increasing malaria incidence

|  | **RTS,S/AS01 vaccine** | | | | **Control vaccine** | | | | **Protective Efficacy** | |
| --- | --- | --- | --- | --- | --- | --- | --- | --- | --- | --- |
| **Clinical malaria - primary case definition  (per-protocol population)** | **Number of children (N)** | **Number of episodes (n)** | **Person years at risk (T)** | **n/T** | **Number of children (N)** | **Number of episodes (n)** | **Person years at risk (T)** | **n/T** | **% (95% CI)** | **p-value** |
| Kilifi | 336 | 4 | 450.7 | 0.01 | 171 | 9 | 229.3 | 0.04 | 77.4 (26.4-93.1) | 0.013 |
| Korogwe | 568 | 33 | 817.3 | 0.04 | 293 | 44 | 418.7 | 0.11 | 61.1 (34.8-76.8) | <0.001 |
| Lambarene | 380 | 63 | 551.0 | 0.11 | 196 | 57 | 283.4 | 0.20 | 42.5 (11.2-62.7 | 0.012 |
| Bagamoyo | 462 | 68 | 648.4 | 0.10 | 235 | 89 | 321.7 | 0.28 | 65.4 (46.2-77.7) | <0.001 |
| Lilongwe | 359 | 99 | 498.5 | 0.20 | 183 | 82 | 253.6 | 0.32 | 42.4 (12.9-61.9) | 0.009 |
| Agogo | 371 | 288 | 511.7 | 0.56 | 192 | 296 | 256.2 | 1.16 | 53.6 (40.3-63.8) | <0.001 |
| Kombewa | 609 | 978 | 811.1 | 1.21 | 311 | 762 | 407.7 | 1.87 | 40.2 (28.5-49.9) | <0.001 |
| Kintampo | 602 | 801 | 793.3 | 1.01 | 296 | 702 | 379.2 | 1.85 | 47.2 (39.1-54.2) | <0.001 |
| Nanoro | 389 | 707 | 498.4 | 1.42 | 198 | 596 | 247.9 | 2.40 | 41.1 (33.6-47.8) | <0.001 |
| Siaya | 481 | 1216 | 605.8 | 2.01 | 253 | 1002 | 302.6 | 3.31 | 43.3 (33.1-51.9) | <0.001 |
| **Overall** | **4557** | **4257** | **6186.0** | **0.69** | **2328** | **3639** | **3100.4** | **1.17** | **45.7 (41.7-49.5)** | **<0.001** |
| **Clinical malaria - secondary case definition 1 (per-protocol population)** | **N** | **n** | **T (year)** | **n/T** | **N** | **n** | **T (year)** | **n/T** | **% (95% CI)** | **p-value** |
| Kilifi | 336 | 6 | 450.6 | 0.01 | 171 | 11 | 229.2 | 0.05 | 72.3 (22.8-90.0) | 0.014 |
| Korogwe | 568 | 44 | 816.9 | 0.05 | 293 | 53 | 418.3 | 0.13 | 56.8 (30.9-73.0) | <0.001 |
| Lambarene | 380 | 107 | 549.3 | 0.19 | 196 | 105 | 281.6 | 0.37 | 49.8 (26.0-65.9) | <0.001 |
| Bagamoyo | 462 | 103 | 647.1 | 0.16 | 235 | 122 | 320.4 | 0.38 | 61.4 (44.1-73.4) | <0.001 |
| Lilongwe | 359 | 137 | 497.1 | 0.28 | 183 | 121 | 252.2 | 0.48 | 46.6 (23.2-62.8) | <0.001 |
| Agogo | 371 | 497 | 503.6 | 0.99 | 192 | 476 | 249.4 | 1.91 | 50.2 (38.2-59.8) | <0.001 |
| Kombewa | 609 | 1594 | 787.3 | 2.02 | 311 | 1196 | 390.7 | 3.06 | 41.0 (29.8-50.4) | <0.001 |
| Kintampo | 602 | 1260 | 775.5 | 1.62 | 296 | 1049 | 365.6 | 2.87 | 46.3 (38.8-52.9) | <0.001 |
| Nanoro | 389 | 887 | 491.5 | 1.80 | 198 | 749 | 242.0 | 3.09 | 41.7 (35.5-47.4) | <0.001 |
| Siaya | 481 | 1981 | 575.8 | 3.44 | 253 | 1527 | 282.3 | 5.41 | 41.4 (31.8-49.7) | <0.001 |
| **Overall** | **4557** | **6616** | **6094.5** | **1.09** | **2328** | **5409** | **3031.8** | **1.78** | **45.4 (41.6-48.9)** | **<0.001** |
| **Clinical malaria - secondary case definition 2 (per-protocol population)** | **N** | **n** | **T (year)** | **n/T** | **N** | **n** | **T (year)** | **n/T** | **% (95% CI)** | **p-value** |
| Kilifi | 336 | 4 | 450.7 | 0.01 | 171 | 9 | 229.3 | 0.04 | 77.4 (26.4-93.1) | 0.013 |
| Korogwe | 568 | 38 | 817.1 | 0.05 | 293 | 48 | 418.5 | 0.11 | 59.1 (33.4-75.0) | <0.001 |
| Lambarene | 380 | 71 | 550.6 | 0.13 | 196 | 64 | 283.1 | 0.23 | 42.9 (13.0-62.5) | 0.009 |
| Bagamoyo | 462 | 76 | 648.1 | 0.12 | 235 | 103 | 321.2 | 0.32 | 65.5 (47.9-77.1) | <0.001 |
| Lilongwe | 359 | 113 | 498.0 | 0.23 | 183 | 92 | 253.3 | 0.36 | 42.0 (14.4-60.8) | 0.006 |
| Agogo | 371 | 343 | 509.6 | 0.67 | 192 | 333 | 254.8 | 1.31 | 49.9 (37.0-60.2) | <0.001 |
| Kombewa | 609 | 1127 | 805.4 | 1.4 | 311 | 866 | 403.7 | 2.15 | 40.1 (28.9-49.6) | <0.001 |
| Kintampo | 602 | 873 | 790.5 | 1.1 | 296 | 757 | 377.1 | 2.01 | 46.7 (38.8-53.5) | <0.001 |
| Nanoro | 389 | 789 | 495.2 | 1.59 | 198 | 672 | 245.0 | 2.74 | 42.0 (35.2-48.1) | <0.001 |
| Siaya | 481 | 1346 | 600.8 | 2.24 | 253 | 1076 | 299.7 | 3.59 | 41.4 (31.2-50.0) | <0.001 |
| **Overall** | **4557** | **4780** | **6165.8** | **0.78** | **2328** | **4020** | **3085.6** | **1.3** | **45.0 (41.1-48.6)** | **<0.001** |
| **Clinical malaria - secondary case definition 3 (per-protocol population)** | **N** | **n** | **T (year)** | **n/T** | **N** | **n** | **T (year)** | **n/T** | **% (95% CI)** | **p-value** |
| Kilifi | 336 | 3 | 450.7 | 0.01 | 171 | 9 | 229.3 | 0.04 | 83.0 (37.2-95.4) | 0.008 |
| Korogwe | 568 | 24 | 817.6 | 0.03 | 293 | 37 | 419.0 | 0.09 | 67.2 (40.4-81.9) | <0.001 |
| Lambarene | 380 | 50 | 551.4 | 0.09 | 196 | 44 | 283.9 | 0.15 | 40.1 (3.4-62.9) | 0.035 |
| Bagamoyo | 462 | 52 | 649.0 | 0.08 | 235 | 75 | 322.3 | 0.23 | 69.1 (49.1-81.2) | <0.001 |
| Lilongwe | 359 | 84 | 499.1 | 0.17 | 183 | 68 | 254.2 | 0.27 | 38.5 (5.0-60.1) | 0.028 |
| Agogo | 371 | 226 | 514.0 | 0.44 | 192 | 232 | 258.6 | 0.9 | 53.6 (38.8-64.9) | <0.001 |
| Kombewa | 609 | 818 | 817.2 | 1 | 311 | 656 | 411.8 | 1.59 | 41.2 (29.2-51.2) | <0.001 |
| Kintampo | 602 | 709 | 796.8 | 0.89 | 296 | 621 | 382.3 | 1.62 | 47.0 (38.2-54.6) | <0.001 |
| Nanoro | 389 | 604 | 502.2 | 1.2 | 198 | 507 | 251.2 | 2.02 | 40.6 (32.3-47.9) | <0.001 |
| Siaya | 481 | 1082 | 610.9 | 1.77 | 253 | 895 | 306.8 | 2.92 | 43.0 (32.3-51.9) | <0.001 |
| **Overall** | **4557** | **3652** | **6208.9** | **0.59** | **2328** | **3144** | **3119.3** | **1.01** | **45.9 (41.6-49.8)** | **<0.001** |
| **Clinical malaria - primary case definition  (intention-to-treat population)** | **N** | **n** | **T (year)** | **n/T** | **N** | **n** | **T (year)** | **n/T** | **% (95% CI)** | **p-value** |
| Kilifi | 398 | 6 | 578.1 | 0.01 | 202 | 10 | 291.3 | 0.03 | 69.8 (16.7-89.0) | 0.020 |
| Korogwe | 608 | 46 | 994.8 | 0.05 | 304 | 53 | 497.2 | 0.11 | 55.7 (30.6-71.7) | <0.001 |
| Manhiça | 664 | 49 | 1066.2 | 0.05 | 338 | 51 | 548.2 | 0.09 | 49.9 (9.2-72.4) | 0.022 |
| Lambarene | 470 | 77 | 724.0 | 0.11 | 234 | 74 | 366.4 | 0.2 | 47.7 (23.0-64.5) | 0.001 |
| Bagamoyo | 605 | 157 | 913.1 | 0.17 | 298 | 146 | 443.6 | 0.33 | 49.5 (29.2-64.0) | <0.001 |
| Lilongwe | 539 | 154 | 845.4 | 0.18 | 261 | 139 | 412.4 | 0.34 | 47.7 (27.7-62.2) | <0.001 |
| Agogo | 400 | 345 | 637.7 | 0.54 | 200 | 341 | 307.9 | 1.11 | 52.9 (40.3-62.9) | <0.001 |
| Kombewa | 668 | 1096 | 978.9 | 1.12 | 332 | 858 | 482.8 | 1.78 | 41.7 (30.8-50.9) | <0.001 |
| Kintampo | 668 | 968 | 999.9 | 0.97 | 334 | 821 | 482.3 | 1.7 | 44.7 (36.9-51.5) | <0.001 |
| Nanoro | 397 | 798 | 584.7 | 1.36 | 203 | 669 | 290.1 | 2.31 | 40.9 (33.8-47.3) | <0.001 |
| Siaya | 532 | 1410 | 736.4 | 1.91 | 268 | 1143 | 362.3 | 3.15 | 42.4 (32.8-50.7) | <0.001 |
| **Overall** | **5949** | **5106** | **9059.1** | **0.56** | **2974** | **4305** | **4484.4** | **0.96** | **45.1 (41.4-48.7)** | **<0.001** |
| **Clinical malaria - secondary case definition 1 (intention-to-treat population)** | **N** | **n** | **T (year)** | **n/T** | **N** | **n** | **T (year)** | **n/T** | **% (95% CI)** | **p-value** |
| Kilifi | 398 | 9 | 578.0 | 0.02 | 202 | 12 | 291.2 | 0.04 | 62.2 (8.4-84.4) | 0.031 |
| Korogwe | 608 | 59 | 994.3 | 0.06 | 304 | 66 | 496.7 | 0.13 | 53.6 (28.9-69.8) | <.001 |
| Manhiça | 664 | 93 | 1064.5 | 0.09 | 338 | 84 | 547.0 | 0.15 | 46.5 (15.5-66.1) | 0.007 |
| Lambarene | 470 | 135 | 721.8 | 0.19 | 234 | 134 | 364.1 | 0.37 | 51.6 (31.5-65.8) | <0.001 |
| Bagamoyo | 605 | 245 | 909.6 | 0.27 | 298 | 201 | 441.5 | 0.46 | 43.7 (24.8-57.8) | <0.001 |
| Lilongwe | 539 | 227 | 842.7 | 0.27 | 261 | 200 | 410.1 | 0.49 | 47.2 (29.8-60.3) | <0.001 |
| Agogo | 400 | 606 | 627.5 | 0.97 | 200 | 551 | 300.0 | 1.84 | 49.3 (38.1-58.6) | <0.001 |
| Kombewa | 668 | 1838 | 950.1 | 1.93 | 332 | 1347 | 463.7 | 2.9 | 40.8 (29.8-50.0) | <0.001 |
| Kintampo | 668 | 1593 | 975.8 | 1.63 | 334 | 1260 | 465.2 | 2.71 | 42.2 (34.9-48.7) | <0.001 |
| Nanoro | 397 | 1049 | 575.6 | 1.82 | 203 | 867 | 282.5 | 3.07 | 40.6 (34.7-45.9) | <0.001 |
| Siaya | 532 | 2353 | 699.7 | 3.36 | 268 | 1760 | 338.6 | 5.2 | 39.2 (29.8-47.4) | <0.001 |
| **Overall** | **5949** | **8207** | **8939.1** | **0.92** | **2974** | **6482** | **4400.6** | **1.47** | **43.1 (39.5-46.5)** | **<0.001** |
| **Clinical malaria - secondary case definition 2 (intention-to-treat population)** | **N** | **n** | **T (year)** | **n/T** | **N** | **n** | **T (year)** | **n/T** | **% (95% CI)** | **p-value** |
| Kilifi | 398 | 6 | 578.1 | 0.01 | 202 | 10 | 291.3 | 0.03 | 69.8 (16.7-89.0) | 0.020 |
| Korogwe | 608 | 53 | 994.5 | 0.05 | 304 | 58 | 497.0 | 0.12 | 52.8 (27.3-69.4) | <0.001 |
| Manhiça | 664 | 54 | 1066.0 | 0.05 | 338 | 52 | 548.2 | 0.09 | 45.7 (3.9-69.3) | 0.036 |
| Lambarene | 470 | 87 | 723.6 | 0.12 | 234 | 83 | 366.0 | 0.23 | 48.4 (24.7-64.6) | <0.001 |
| Bagamoyo | 605 | 182 | 912.1 | 0.2 | 298 | 166 | 442.9 | 0.37 | 47.6 (27.9-62.0) | <0.001 |
| Lilongwe | 539 | 180 | 844.4 | 0.21 | 261 | 154 | 411.9 | 0.37 | 45.6 (25.7-60.2) | <0.001 |
| Agogo | 400 | 415 | 634.9 | 0.65 | 200 | 388 | 306.1 | 1.27 | 49.9 (37.7-59.8) | <0.001 |
| Kombewa | 668 | 1278 | 971.9 | 1.31 | 332 | 973 | 478.5 | 2.03 | 40.7 (29.9-49.8) | <0.001 |
| Kintampo | 668 | 1078 | 995.7 | 1.08 | 334 | 890 | 479.6 | 1.86 | 43.2 (35.5-49.9) | <0.001 |
| Nanoro | 397 | 917 | 580.1 | 1.58 | 203 | 770 | 286.2 | 2.69 | 41.3 (34.9-47.1) | <0.001 |
| Siaya | 532 | 1575 | 730.1 | 2.16 | 268 | 1231 | 358.9 | 3.43 | 40.1 (30.6-48.4) | <0.001 |
| **Overall** | **5949** | **5825** | **9031.4** | **0.64** | **2974** | **4775** | **4466.5** | **1.07** | **43.6 (39.9-47.0)** | **<0.001** |
| **Clinical malaria - secondary case definition 3 (intention-to-treat population)** | **N** | **n** | **T (year)** | **n/T** | **N** | **n** | **T (year)** | **n/T** | **% (95% CI)** | **p-value** |
| Kilifi | 398 | 4 | 578.2 | 0.01 | 202 | 9 | 291.3 | 0.03 | 77.6 (27.1-93.1) | 0.013 |
| Korogwe | 608 | 33 | 995.3 | 0.03 | 304 | 42 | 497.6 | 0.08 | 60.0 (32.3-76.4) | <0.001 |
| Manhiça | 664 | 40 | 1066.5 | 0.04 | 338 | 41 | 548.6 | 0.07 | 47.2 (-1.7-72.6) | 0.056 |
| Lambarene | 470 | 60 | 724.6 | 0.08 | 234 | 53 | 367.2 | 0.14 | 42.0 (11.0-62.2) | 0.012 |
| Bagamoyo | 605 | 120 | 914.5 | 0.13 | 298 | 121 | 444.6 | 0.27 | 53.6 (32.4-68.1) | <0.001 |
| Lilongwe | 539 | 128 | 846.4 | 0.15 | 261 | 115 | 413.4 | 0.28 | 46.9 (24.7-62.6) | <0.001 |
| Agogo | 400 | 273 | 640.4 | 0.43 | 200 | 268 | 310.6 | 0.86 | 52.8 (38.5-63.7) | <0.001 |
| Kombewa | 668 | 904 | 986.1 | 0.92 | 332 | 735 | 487.4 | 1.51 | 43.1 (32.0-52.4) | <0.001 |
| Kintampo | 668 | 852 | 1004.3 | 0.85 | 334 | 717 | 486.3 | 1.47 | 44.0 (35.5-51.4) | <0.001 |
| Nanoro | 397 | 667 | 589.6 | 1.13 | 203 | 564 | 294.0 | 1.92 | 41.2 (33.3-48.2) | <0.001 |
| Siaya | 532 | 1243 | 742.8 | 1.67 | 268 | 1012 | 367.3 | 2.76 | 42.0 (32.0-50.6) | <0.001 |
| **Overall** | **5949** | **4324** | **9088.6** | **0.48** | **2974** | **3677** | **4508.3** | **0.82** | **45.3 (41.3-49.0)** | **<0.001** |

Clinical malaria primary case definition: Illness in a child brought to a study facility with a temperature of ≥ 37.5°C and *P. falciparum* asexual parasitemia at a density of > 5000 parasites per cubic millimeter or a case of malaria meeting the primary case definition of severe malaria.

Severe malaria primary case definition: *P. falciparum* asexual parasitemia at a density of > 5000 parasites per cubic millimeter with one or more markers of disease severity and without diagnosis of a coexisting illness.

Clinical malaria secondary case definition 1: Illness in a child brought to a study facility with a measured temperature of ≥37.5°C or reported fever within the last 24 hours and *P. falciparum* asexual parasitemia at a density of > 0 parasites per cubic millimeter.

Clinical malaria secondary case definition 2: Illness in a child brought to a study facility with a measured temperature of ≥37.5°C and *P. falciparum* asexual parasitemia at a density of > 500 parasites per cubic millimeter.

Clinical malaria secondary case definition 3: Illness in a child brought to a study facility with a measured temperature of ≥37.5°C and *P. falciparum* asexual parasitemia at a density of > 20,000 parasites per cubic millimeter.

P-value from negative binomial regression.

## Supplementary table 5b. Vaccine efficacy against all episodes of clinical malaria (primary and secondary case definitions) during a 18-month follow-up period post dose-3 in the 6-12 week age category, ordered by increasing malaria incidence

|  | **RTS,S/AS01 vaccine** | | | | **Control vaccine** | | | | **Protective Efficacy** | |
| --- | --- | --- | --- | --- | --- | --- | --- | --- | --- | --- |
| **Clinical malaria - primary case definition  (per-protocol population)** | **Number of children (N)** | **Number of episodes (n)** | **Person years at risk (T)** | **n/T** | **Number of children (N)** | **Number of episodes (n)** | **Person years at risk (T)** | **n/T** | **% (95% CI)** | **p-value** |
| Kilifi | 186 | 9 | 253.2 | 0.04 | 102 | 3 | 139.0 | 0.02 | -56.5 (-599-65.0) | 0.556 |
| Korogwe | 382 | 15 | 531.0 | 0.03 | 183 | 14 | 255.5 | 0.05 | 48.5 (-7.0-75.2) | 0.075 |
| Manhiça | 381 | 52 | 524.4 | 0.10 | 188 | 32 | 259.1 | 0.12 | 20.2 (-31.8-51.6) | 0.377 |
| Lambarene | 147 | 23 | 208.2 | 0.11 | 62 | 11 | 88.3 | 0.12 | 8.7 (-113-60.8) | 0.831 |
| Bagamoyo | 502 | 52 | 693.2 | 0.08 | 244 | 48 | 340.0 | 0.14 | 34.8 (-21.064.9) | 0.174 |
| Lilongwe | 500 | 211 | 698.3 | 0.30 | 258 | 177 | 355.8 | 0.50 | 43.0 (21.2-58.8) | <0.001 |
| Agogo | 418 | 365 | 573.3 | 0.64 | 221 | 238 | 302.9 | 0.79 | 19.5 (-2.7-36.9) | 0.080 |
| Kombewa | 387 | 505 | 535.0 | 0.94 | 196 | 339 | 256.2 | 1.32 | 33.7 (14.2-48.8) | 0.001 |
| Kintampo | 199 | 390 | 255.5 | 1.53 | 99 | 192 | 128.9 | 1.49 | -2.7 (-29.3-18.5) | 0.822 |
| Nanoro | 441 | 1055 | 547.2 | 1.93 | 225 | 649 | 271.6 | 2.39 | 20.0 (9.6-29.1) | <0.001 |
| Siaya | 453 | 1171 | 577.3 | 2.03 | 229 | 761 | 276.8 | 2.75 | 32.2 (19.2-43.2) | <0.001 |
| **Overall** | **3996** | **3848** | **5396.8** | **0.71** | **2007** | **2464** | **2674.0** | **0.92** | **26.6 (20.3-32.4)** | **<0.001** |
| **Clinical malaria - secondary case definition 1 (per-protocol population)** | **N** | **n** | **T (year)** | **n/T** | **N** | **n** | **T (year)** | **n/T** | **% (95% CI)** | **p-value** |
| Kilifi | 186 | 15 | 253.0 | 0.06 | 102 | 7 | 138.8 | 0.05 | -16.3 (-246-60.9) | 0.785 |
| Korogwe | 382 | 21 | 530.8 | 0.04 | 183 | 22 | 255.2 | 0.09 | 54.3 (15.1-75.3) | 0.013 |
| Manhiça | 381 | 86 | 523.1 | 0.16 | 188 | 54 | 258.3 | 0.21 | 21.0 (-19.0-47.6) | 0.258 |
| Lambarene | 147 | 39 | 207.7 | 0.19 | 62 | 19 | 88.0 | 0.22 | 9.5 (-86.8-56.2) | 0.785 |
| Bagamoyo | 502 | 90 | 691.8 | 0.13 | 244 | 69 | 339.2 | 0.20 | 29.3 (-15.8-56.8) | 0.168 |
| Lilongwe | 500 | 336 | 693.6 | 0.48 | 258 | 275 | 352.2 | 0.78 | 43.5 (23.4-58.3) | <0.001 |
| Agogo | 418 | 649 | 562.4 | 1.15 | 221 | 426 | 295.6 | 1.44 | 21.5 (3.0-36.4) | 0.024 |
| Kombewa | 387 | 808 | 523.1 | 1.54 | 196 | 540 | 248.2 | 2.18 | 36.2 (18.5-50.0) | <0.001 |
| Kintampo | 199 | 573 | 248.3 | 2.31 | 99 | 305 | 124.3 | 2.45 | 5.5 (-17.7-24.2) | 0.611 |
| Nanoro | 441 | 1372 | 534.8 | 2.57 | 225 | 850 | 263.7 | 3.22 | 20.9 (12.1-28.7) | <0.001 |
| Siaya | 453 | 1792 | 552.9 | 3.24 | 229 | 1151 | 261.2 | 4.41 | 32.9 (20.7-43.1) | <0.001 |
| **Overall** | **3996** | **5781** | **5321.4** | **1.09** | **2007** | **3718** | **2624.6** | **1.42** | **27.8 (22.0-33.1)** | **<0.001** |
| **Clinical malaria - secondary case definition 2 (per-protocol population)** | **N** | **n** | **T (year)** | **n/T** | **N** | **n** | **T (year)** | **n/T** | **% (95% CI)** | **p-value** |
| Kilifi | 186 | 10 | 253.2 | 0.04 | 102 | 4 | 138.9 | 0.03 | -45.7 (-521-65.9) | 0.610 |
| Korogwe | 382 | 17 | 531.0 | 0.03 | 183 | 21 | 255.2 | 0.08 | 61.1 (25.6-79.7) | 0.004 |
| Manhiça | 381 | 54 | 524.3 | 0.1 | 188 | 35 | 259.0 | 0.14 | 24.6 (-22.1-53.5) | 0.249 |
| Lambarene | 147 | 27 | 208.1 | 0.13 | 62 | 12 | 88.3 | 0.14 | 2.7 (-114-55.7) | 0.946 |
| Bagamoyo | 502 | 74 | 692.4 | 0.11 | 244 | 58 | 339.6 | 0.17 | 28.7 (-20.2-57.7) | 0.203 |
| Lilongwe | 500 | 246 | 697.0 | 0.35 | 258 | 203 | 354.9 | 0.57 | 42.9 (22.5-58.0) | <0.001 |
| Agogo | 418 | 434 | 570.7 | 0.76 | 221 | 273 | 301.6 | 0.91 | 16.8 (-4.77-34.0) | 0.117 |
| Kombewa | 387 | 571 | 532.4 | 1.07 | 196 | 391 | 254.1 | 1.54 | 35.3 (17.4-49.3) | <0.001 |
| Kintampo | 199 | 429 | 254.0 | 1.69 | 99 | 212 | 128.1 | 1.66 | -2.1 (-28.0-18.5) | 0.856 |
| Nanoro | 441 | 1213 | 541.1 | 2.24 | 225 | 750 | 267.6 | 2.8 | 20.6 (11.4-28.8) | <0.001 |
| Siaya | 453 | 1277 | 573.1 | 2.23 | 229 | 814 | 274.7 | 2.96 | 30.3 (17.1-41.4) | <0.001 |
| **Overall** | **3996** | **4352** | **5377.3** | **0.81** | **2007** | **2773** | **2662.0** | **1.04** | **26.2 (20.2-31.8)** | **<0.001** |
| **Clinical malaria - secondary case definition 3 (per-protocol population)** | **N** | **n** | **T (year)** | **n/T** | **N** | **n** | **T (year)** | **n/T** | **% (95% CI)** | **p-value** |
| Kilifi | 186 | 4 | 253.4 | 0.02 | 102 | 3 | 139.0 | 0.02 | 35.9 (-278-89.1) | 0.622 |
| Korogwe | 382 | 13 | 531.1 | 0.02 | 183 | 11 | 255.6 | 0.04 | 43.1 (-27.2-74.6) | 0.168 |
| Manhiça | 381 | 31 | 525.2 | 0.06 | 188 | 24 | 259.4 | 0.09 | 39.6 (-13.7-67.9) | 0.118 |
| Lambarene | 147 | 17 | 208.5 | 0.08 | 62 | 7 | 88.4 | 0.08 | -3.1 (-175-61.3) | 0.951 |
| Bagamoyo | 502 | 40 | 693.7 | 0.06 | 244 | 42 | 340.3 | 0.12 | 47.3 (-5.23-73.6) | 0.069 |
| Lilongwe | 500 | 169 | 670.0 | 0.24 | 258 | 143 | 357.1 | 0.4 | 42.6 (17.5-60.1) | 0.002 |
| Agogo | 418 | 270 | 577.1 | 0.47 | 221 | 177 | 305.2 | 0.58 | 21.5 (-3.1-40.2) | 0.081 |
| Kombewa | 387 | 418 | 538.4 | 0.78 | 196 | 280 | 258.4 | 1.08 | 34.2 (13.4-50.0) | 0.002 |
| Kintampo | 199 | 345 | 257.3 | 1.34 | 99 | 158 | 130.3 | 1.21 | -10.7 (-43.3-14.5) | 0.438 |
| Nanoro | 441 | 850 | 555.0 | 1.53 | 225 | 542 | 275.7 | 1.97 | 22.8 (11.7-32.5) | <0.001 |
| Siaya | 453 | 1014 | 583.5 | 1.74 | 229 | 656 | 280.8 | 2.34 | 31.8 (17.9-43.4) | <0.001 |
| **Overall** | **3996** | **3171** | **5423.0** | **0.58** | **2007** | **2043** | **2690.1** | **0.76** | **27.8 (21.0-34.0)** | **<0.001** |
| **Clinical malaria - primary case definition  (intention-to-treat population)** | **N** | **n** | **T (year)** | **n/T** | **N** | **n** | **T (year)** | **n/T** | **% (95% CI)** | **p-value** |
| Kilifi | 199 | 9 | 299.5 | 0.03 | 105 | 3 | 160.2 | 0.02 | -51.9 (-580-66.1) | 0.583 |
| Korogwe | 398 | 16 | 634.1 | 0.03 | 195 | 15 | 307.6 | 0.05 | 48.3 (-4.8-74.5) | 0.067 |
| Manhiça | 423 | 54 | 656.57 | 0.08 | 212 | 35 | 327.4 | 0.11 | 23.6 (-23.1-52.6) | 0.268 |
| Lambarene | 158 | 23 | 240.7 | 0.1 | 68 | 11 | 104.2 | 0.11 | 6.7 (-118-60.0) | 0.872 |
| Bagamoyo | 533 | 55 | 820.5 | 0.07 | 269 | 55 | 416.6 | 0.13 | 37.8 (-13.0-65.7) | 0.118 |
| Lilongwe | 547 | 231 | 838.6 | 0.28 | 279 | 186 | 437.1 | 0.43 | 39.0 (17.2-55.0) | 0.001 |
| Agogo | 458 | 402 | 718.1 | 0.56 | 230 | 247 | 358.6 | 0.69 | 18.8 (-2.7-35.8) | 0.082 |
| Kombewa | 421 | 548 | 640.9 | 0.86 | 210 | 366 | 305.8 | 1.2 | 34.2 (15.7-48.7) | 0.001 |
| Kintampo | 221 | 438 | 326.7 | 1.34 | 110 | 215 | 164.1 | 1.31 | -1.9 (-27.0-18.1) | 0.863 |
| Nanoro | 453 | 1134 | 649.9 | 1.74 | 228 | 698 | 322.6 | 2.16 | 19.8 (9.8-28.7) | <0.001 |
| Siaya | 547 | 1342 | 758.0 | 1.77 | 273 | 920 | 369.5 | 2.49 | 34.9 (23.6-44.5) | <0.001 |
| **Overall** | **4358** | **4252** | **6583.6** | **0.65** | **2179** | **2751** | **3273.6** | **0.84** | **27.0 (21.1-32.5)** | **<0.001** |
| **Clinical malaria - secondary case definition 1 (intention-to-treat population)** | **N** | **n** | **T (year)** | **n/T** | **N** | **n** | **T (year)** | **n/T** | **% (95% CI)** | **p-value** |
| Kilifi | 199 | 15 | 299.3 | 0.05 | 105 | 7 | 160.0 | 0.04 | -13.1 (-238-62.2) | 0.825 |
| Korogwe | 398 | 22 | 633.9 | 0.03 | 195 | 23 | 307.3 | 0.07 | 54.1 (14.7-75.3) | 0.013 |
| Manhiça | 423 | 89 | 655.2 | 0.14 | 212 | 63 | 326.3 | 0.19 | 29.5 (-3.6-52.1) | 0.075 |
| Lambarene | 158 | 41 | 240.1 | 0.17 | 68 | 22 | 103.8 | 0.21 | 17.2 (-61.7-57.6) | 0.579 |
| Bagamoyo | 533 | 97 | 818.9 | 0.12 | 269 | 81 | 415.6 | 0.19 | 32.4 (-7.9-57.6) | 0.100 |
| Lilongwe | 547 | 365 | 833.6 | 0.44 | 279 | 291 | 433.2 | 0.67 | 39.8 (20.0-54.6) | <0.001 |
| Agogo | 458 | 721 | 706.0 | 1.02 | 230 | 449 | 350.8 | 1.28 | 21.2 (3.5-35.6) | 0.021 |
| Kombewa | 421 | 907 | 627.0 | 1.45 | 210 | 598 | 296.6 | 2.02 | 35.2 (18.4-48.5) | <0.001 |
| Kintampo | 221 | 666 | 317.9 | 2.1 | 110 | 351 | 158.7 | 2.21 | 5.7 (-15.4-23.0) | 0.568 |
| Nanoro | 453 | 1521 | 635.1 | 2.39 | 228 | 941 | 313.2 | 3 | 20.6 (12.5-28.0) | <0.001 |
| Siaya | 547 | 2120 | 727.8 | 2.91 | 273 | 1395 | 350.8 | 3.98 | 32.9 (22.0-42.3) | <0.001 |
| **Overall** | **4358** | **6564** | **6494.6** | **1.01** | **2179** | **4221** | **3216.4** | **1.31** | **27.7 (22.3-32.7)** | **<0.001** |
| **Clinical malaria - secondary case definition 2 (intention-to-treat population)** | **N** | **n** | **T (year)** | **n/T** | **N** | **n** | **T (year)** | **n/T** | **% (95% CI)** | **p-value** |
| Kilifi | 199 | 10 | 299.5 | 0.03 | 105 | 4 | 160.2 | 0.02 | -41.5 (-504-66.9) | 0.638 |
| Korogwe | 398 | 18 | 634.0 | 0.03 | 195 | 22 | 307.3 | 0.07 | 60.4 (25.6-78.9) | 0.004 |
| Manhiça | 423 | 56 | 656.5 | 0.09 | 212 | 38 | 327.3 | 0.12 | 27.3 (-15.2-54.2) | 0.174 |
| Lambarene | 158 | 28 | 240.6 | 0.12 | 68 | 12 | 104.2 | 0.12 | -3.3 (-125-52.6) | 0.935 |
| Bagamoyo | 533 | 79 | 819.6 | 0.1 | 269 | 67 | 416.1 | 0.16 | 31.2 (-13.0-58.1) | 0.139 |
| Lilongwe | 547 | 269 | 837.2 | 0.32 | 279 | 214 | 436.1 | 0.49 | 39.0 (18.6-54.3) | <0.001 |
| Agogo | 458 | 481 | 715.2 | 0.67 | 230 | 284 | 357.2 | 0.8 | 15.8 (-5.2-32.6) | 0.129 |
| Kombewa | 421 | 629 | 637.8 | 0.99 | 210 | 430 | 303.2 | 1.42 | 35.9 (19.2-49.2) | <0.001 |
| Kintampo | 221 | 494 | 324.6 | 1.52 | 110 | 244 | 163.0 | 1.5 | -1.3 (-25.0-17.9) | 0.901 |
| Nanoro | 453 | 1325 | 642.6 | 2.06 | 228 | 817 | 318.0 | 2.57 | 20.1 (11.3-28.0) | <0.001 |
| Siaya | 547 | 1484 | 752.6 | 1.97 | 273 | 983 | 367.1 | 2.68 | 31.8 (20.2-41.7) | <0.001 |
| **Overall** | **4358** | **4873** | **6560.0** | **0.74** | **2179** | **3115** | **3259.6** | **0.96** | **26.1 (20.5-31.4)** | **<0.001** |
| **Clinical malaria - secondary case definition 3 (intention-to-treat population)** | **N** | **n** | **T (year)** | **n/T** | **N** | **n** | **T (year)** | **n/T** | **% (95% CI)** | **p-value** |
| Kilifi | 199 | 4 | 299.7 | 0.01 | 105 | 3 | 160.2 | 0.02 | 37.8 (-267-89.5) | 0.599 |
| Korogwe | 398 | 14 | 634.2 | 0.02 | 195 | 12 | 307.7 | 0.04 | 43.4 (-22.6-73.9) | 0.148 |
| Manhiça | 423 | 31 | 657.4 | 0.05 | 212 | 26 | 327.8 | 0.08 | 43.9 (-3.7-69.6) | 0.065 |
| Lambarene | 158 | 17 | 240.9 | 0.07 | 68 | 7 | 104.4 | 0.07 | -5.1 (-181-60.6) | 0.920 |
| Bagamoyo | 533 | 42 | 821.0 | 0.05 | 269 | 49 | 416.8 | 0.12 | 51.2 (4.7-75.0) | 0.035 |
| Lilongwe | 547 | 182 | 840.4 | 0.22 | 279 | 149 | 438.5 | 0.34 | 39.4 (14.4-57.1) | 0.004 |
| Agogo | 458 | 293 | 722.4 | 0.41 | 230 | 183 | 361.0 | 0.51 | 22.3 (-1.1-40.3) | 0.060 |
| Kombewa | 421 | 450 | 644.7 | 0.7 | 210 | 296 | 308.4 | 0.96 | 33.6 (13.4-49.1) | 0.002 |
| Kintampo | 221 | 378 | 329.0 | 1.15 | 110 | 176 | 165.6 | 1.06 | -8.0 (-37.6-15.3) | 0.533 |
| Nanoro | 453 | 903 | 658.6 | 1.37 | 228 | 576 | 327.3 | 1.76 | 22.6 (11.8-32.0) | <0.001 |
| Siaya | 547 | 1144 | 765.7 | 1.49 | 273 | 784 | 374.7 | 2.09 | 35.1 (22.9-45.4) | <0.001 |
| **Overall** | **4358** | **3458** | **6614.0** | **0.52** | **2179** | **2261** | **3292.2** | **0.69** | **28.7 (22.3-34.5)** | **<0.001** |

Clinical malaria primary case definition: Illness in a child brought to a study facility with a temperature of ≥ 37.5°C and *P. falciparum* asexual parasitemia at a density of > 5000 parasites per cubic millimeter or a case of malaria meeting the primary case definition of severe malaria.

Severe malaria primary case definition: *P. falciparum* asexual parasitemia at a density of > 5000 parasites per cubic millimeter with one or more markers of disease severity and without diagnosis of a coexisting illness.

Clinical malaria secondary case definition 1: Illness in a child brought to a study facility with a measured temperature of ≥37.5°C or reported fever within the last 24 hours and *P. falciparum* asexual parasitemia at a density of > 0 parasites per cubic millimeter.

Clinical malaria secondary case definition 2: Illness in a child brought to a study facility with a measured temperature of ≥37.5°C and *P. falciparum* asexual parasitemia at a density of > 500 parasites per cubic millimeter.

Clinical malaria secondary case definition 3: Illness in a child brought to a study facility with a measured temperature of ≥37.5°C and *P. falciparum* asexual parasitemia at a density of > 20,000 parasites per cubic millimeter.

P-value from negative binomial regression.
