# Supplementary material for: Efficacy and Safety of the RTS,S/AS01 Malaria Vaccine during 18 Months after Vaccination: A Phase 3 Randomized, Controlled Trial in Children and Young Infants at 11 African Sites
Source: PLoS Med. 2014 Jul 29;11(7):e1001685. doi: 10.1371/journal.pmed.1001685 (PMC4114488; doi:10.1371/journal.pmed.1001685)
Supplement: Table S6 — Determinants of vaccine efficacy (incidence of clinical malaria primary case definition, all episodes) full models and final models during an 18-mo follow-up period in the 5–17-mo and 6–12-wk age categories (per-protocol population). (DOCX) [file pmed.1001685.s015.docx]

## Supplementary table 6a. Determinants of vaccine efficacy (incidence of clinical malaria primary case definition, all episodes) full model during an 18-month follow-up period in the 5-17 months age category (per-protocol population)

| **Parameter** | **Incident rate ratio** | **95% CI** | | **p-value** |
| --- | --- | --- | --- | --- |
|  |  | **LL** | **UL** |  |
| Treatment | 0.54 | 0.49 | 0.59 | <.001 |
| Male versus female | 1.08 | 0.97 | 1.21 | 0.170 |
| Anti-CS study site average | 18.43 | 6.78 | 50.07 | <.001 |
| Indoor Residual Spraying | 1.00 | 0.66 | 1.51 | 0.994 |
| Bednet use | 1.06 | 0.89 | 1.26 | 0.531 |
| Outpatient distance within 5 kilometers | 1.01 | 0.89 | 1.14 | 0.884 |
| Age 5-11 months versus 12-17 months | 0.89 | 0.80 | 1.00 | 0.056 |
| Low HAZ versus normal HAZ | 1.03 | 0.89 | 1.18 | 0.739 |
| Low WAZ versus normal WAZ | 0.84 | 0.71 | 1.00 | 0.045 |
| Moderate anemia | 0.96 | 0.82 | 1.14 | 0.652 |
| Incidence in controls | 2.14 | 1.98 | 2.30 | <.001 |
| Treatment x Male versus female | 0.95 | 0.82 | 1.10 | 0.520 |
| Treatment x Anti-CS study site average | 2.51 | 0.65 | 9.74 | 0.182 |
| Treatment x Indoor Residual Spraying | 0.94 | 0.54 | 1.65 | 0.838 |
| Treatment x Bednet use | 1.02 | 0.81 | 1.27 | 0.896 |
| Treatment x Outpatient distance within 5 kilometers | 1.11 | 0.94 | 1.30 | 0.228 |
| Treatment x Age 5-11 months versus 12-17 months | 1.05 | 0.91 | 1.22 | 0.513 |
| Treatment x Low HAZ versus normal HAZ | 0.93 | 0.77 | 1.12 | 0.455 |
| Treatment x Low WAZ versus normal WAZ | 1.20 | 0.96 | 1.49 | 0.106 |
| Treatment x Moderate anemia | 1.21 | 0.98 | 1.50 | 0.071 |
| Treatment x Incidence in controls | 0.98 | 0.89 | 1.08 | 0.655 |
| Dispersion | 2.21 | 2.06 | 2.38 | _ |

The model included an intercept (not presented in the table above).

Incident rate ratios >1 corresponds to a factor associated with an increased risk of malaria.

LL = Lower limit of the 95% confidence interval.

UL = Upper limit of the 95% confidence interval.

HAZ = Height-for-age Z-score.

WAZ = Weight-for-age Z-score.

P-value from negative binomial regression.

## Supplementary table 6b. Determinants of vaccine efficacy (incidence of clinical malaria primary case definition, all episodes) final model during an 18-month follow-up period in the 5-17 months age category (per-protocol population)

| **Parameter** | **Incident rate ratio** | **95% CI** | | **p-value** |
| --- | --- | --- | --- | --- |
|  |  | **LL** | **UL** |  |
| Treatment | 0.56 | 0.52 | 0.60 | <.001 |
| Male versus female | 1.05 | 0.98 | 1.13 | 0.168 |
| Anti-CS study site average | 29.92 | 15.22 | 58.80 | <.001 |
| Indoor Residual Spraying | 0.98 | 0.74 | 1.29 | 0.864 |
| Bednet use | 1.07 | 0.96 | 1.19 | 0.246 |
| Outpatient distance within 5 kilometers | 1.07 | 0.99 | 1.16 | 0.097 |
| Age 5-11 months versus 12-17 months | 0.92 | 0.85 | 0.99 | 0.021 |
| Low HAZ versus. normal HAZ | 0.99 | 0.90 | 1.08 | 0.742 |
| Low WAZ versus normal WAZ | 0.94 | 0.84 | 1.04 | 0.228 |
| Moderate anemia | 0.95 | 0.80 | 1.11 | 0.502 |
| Incidence in controls | 2.11 | 2.01 | 2.21 | <.001 |
| Treatment x Moderate anemia | 1.24 | 1.01 | 1.52 | 0.041 |
| Dispersion | 2.22 | 2.07 | 2.39 | _ |

The model included an intercept (not presented in the table above).

The full model includes all treatment-covariate interaction and the final model was obtained by stepwise removal of non-significant (>0.05) interaction terms.

Incident rate ratios >1 corresponds to a factor associated with an increased risk of malaria.

LL = Lower limit of the 95% confidence interval.

UL = Upper limit of the 95% confidence interval.

HAZ = Height-for-age Z-score.

WAZ = Weight-for-age Z-score.

P-value from negative binomial regression.

## Supplementary table 6c. Determinants of vaccine efficacy (incidence of clinical malaria primary case definition, all episodes) full model during an 18-month follow-up period in the 6-12 weeks age category (per-protocol population)

| **Parameter** | **Incident rate ratio** | **95% CI** | | **p-value** |
| --- | --- | --- | --- | --- |
|  |  | **LL** | **UL** |  |
| Treatment | 0.76 | 0.69 | 0.84 | <.001 |
| Male versus female | 1.07 | 0.93 | 1.23 | 0.336 |
| Anti-CS study site average | 0.57 | 0.33 | 1.00 | 0.051 |
| Indoor Residual Spraying | 0.74 | 0.44 | 1.26 | 0.271 |
| Bednet use | 0.95 | 0.75 | 1.22 | 0.717 |
| Outpatient distance within 5 kilometers | 0.80 | 0.69 | 0.94 | 0.005 |
| Age 6 weeks versus 7-12 weeks | 0.89 | 0.78 | 1.02 | 0.103 |
| Low HAZ versus normal HAZ | 1.10 | 0.92 | 1.31 | 0.305 |
| Low WAZ versus normal WAZ | 1.04 | 0.80 | 1.35 | 0.765 |
| Moderate anemia | 0.91 | 0.50 | 1.65 | 0.750 |
| Incidence in controls | 2.87 | 2.65 | 3.10 | <.001 |
| Treatment x Male versus female | 1.07 | 0.90 | 1.28 | 0.420 |
| Treatment x Anti-CS study site average | 0.61 | 0.31 | 1.21 | 0.154 |
| Treatment x Indoor Residual Spraying | 1.16 | 0.62 | 2.18 | 0.636 |
| Treatment x Bednet use | 0.94 | 0.69 | 1.27 | 0.675 |
| Treatment x Outpatient distance within 5 kilometers | 0.99 | 0.82 | 1.20 | 0.931 |
| Treatment x Age 6 weeks versus 7-12 weeks | 1.17 | 0.99 | 1.39 | 0.070 |
| Treatment x Low HAZ versus normal HAZ | 0.93 | 0.75 | 1.16 | 0.521 |
| Treatment x Low WAZ versus normal WAZ | 1.00 | 0.72 | 1.40 | 0.981 |
| Treatment x Moderate anemia | 1.47 | 0.73 | 2.99 | 0.284 |
| Treatment x Incidence in controls | 0.99 | 0.90 | 1.10 | 0.902 |
| Dispersion | 2.49 | 2.29 | 2.74 | _ |

The model included an intercept (not presented in the table above).

Incident rate ratios >1 corresponds to a factor associated with an increased risk of malaria.

LL = Lower limit of the 95% confidence interval.

UL = Upper limit of the 95% confidence interval.

HAZ = Height-for-age Z-score.

WAZ = Weight-for-age Z-score.

P-value from negative binomial regression.

## Supplementary table 6d. Determinants of vaccine efficacy (incidence of clinical malaria primary case definition, all episodes) final model during an 18-month follow-up period in the 6-12 weeks age category (per-protocol population)

| **Parameter** | **Incident rate ratio** | **95% CI** | | **p-value** |
| --- | --- | --- | --- | --- |
|  |  | **LL** | **UL** |  |
| Treatment | 0.77 | 0.71 | 0.84 | <.001 |
| Male versus female | 1.12 | 1.03 | 1.22 | 0.007 |
| Anti-CS study site average | 0.42 | 0.30 | 0.58 | <.001 |
| Indoor Residual Spraying | 0.82 | 0.62 | 1.09 | 0.174 |
| Bednet use | 0.92 | 0.80 | 1.07 | 0.284 |
| Outpatient distance within 5 kilometers | 0.80 | 0.73 | 0.88 | <.001 |
| Age 6 weeks versus 7-12 weeks | 0.99 | 0.91 | 1.07 | 0.768 |
| Low HAZ versus normal HAZ | 1.05 | 0.95 | 1.17 | 0.365 |
| Low WAZ versus normal WAZ | 1.04 | 0.89 | 1.22 | 0.605 |
| Moderate anemia | 1.18 | 0.86 | 1.63 | 0.301 |
| Incidence in controls | 2.86 | 2.73 | 2.99 | <.001 |
| Dispersion | 2.50 | 2.29 | 2.75 | _ |

The model included an intercept (not presented in the table above).

The full model includes all treatment-covariate interaction and the final model was obtained by stepwise removal of non-significant (>0.05) interaction terms.

Incident rate ratios >1 corresponds to a factor associated with an increased risk of malaria.

LL = Lower limit of the 95% confidence interval.

UL = Upper limit of the 95% confidence interval.

HAZ = Height-for-age Z-score.

WAZ = Weight-for-age Z-score.

P-value from negative binomial regression.
