# Supplementary material for: Efficacy and Safety of the RTS,S/AS01 Malaria Vaccine during 18 Months after Vaccination: A Phase 3 Randomized, Controlled Trial in Children and Young Infants at 11 African Sites
Source: PLoS Med. 2014 Jul 29;11(7):e1001685. doi: 10.1371/journal.pmed.1001685 (PMC4114488; doi:10.1371/journal.pmed.1001685)
Supplement: Table S7 — Protective efficacy against the prevalence of P. falciparum parasitemia and anemia at 18 mo after dose 3 in the 5–17-mo and 6–12-wk age categories, ordered by increasing malaria incidence. (DOCX) [file pmed.1001685.s016.docx]

## Supplementary table 7a. Protective efficacy against the prevalence of *P. falciparum* parasitemia and anemia at 18 months post dose-3 in the 5-17 months age category

|  | **RTS,S/AS01 vaccine** | | | **Control vaccine** | | | **Protective Efficacy** | |
| --- | --- | --- | --- | --- | --- | --- | --- | --- |
| **Per-protocol population** | **N** | **n** | **Percent**  **affected** | **N** | **n** | **Percent**  **affected** | **% (95% CI)** | **p-value** |
| **Kilifi** |  |  |  |  |  |  |  |  |
| Prevalent parasitemia | 304 | 1 | 0.3 | 152 | 1 | 0.7 | 50.0 (-3825.0-99.4) | 1.000 |
| Prevalent severe anemia | * | * | * | * | * | * |  |  |
| Prevalent moderate anemia | 300 | 2 | 0.7 | 150 | 2 | 1.3 | 50.0 (-590.0-96.4) | 0.603 |
| **Korogwe** |  |  |  |  |  |  |  |  |
| Prevalent parasitemia | 545 | 1 | 0.2 | 271 | 1 | 0.4 | 50.3 (-3803.0-99.4) | 0.554 |
| Prevalent severe anemia | * | * | * | * | * | * |  |  |
| Prevalent moderate anemia | 545 | 7 | 1.3 | 271 | 1 | 0.4 | -248.1 (-16E3-55.3) | 0.281 |
| **Lambarene** |  |  |  |  |  |  |  |  |
| Prevalent parasitemia | 329 | 8 | 2.4 | 172 | 6 | 3.5 | 30.3 (-144.0-78.8) | 0.570 |
| Prevalent severe anemia | * | * | * | * | * | * |  |  |
| Prevalent moderate anemia | 330 | 10 | 3.0 | 172 | 4 | 2.3 | -30.3 (-469.0-62.4) | 0.780 |
| **Bagamoyo** |  |  |  |  |  |  |  |  |
| Prevalent parasitemia | 439 | 4 | 0.9 | 218 | 3 | 1.4 | 33.8 (-352.0-88.8) | 0.690 |
| Prevalent severe anemia | * | * | * | * | * | * |  |  |
| Prevalent moderate anemia | 439 | 13 | 3.0 | 218 | 4 | 1.8 | -61.4 (-580.0-50.1) | 0.448 |
| **Lilongwe** |  |  |  |  |  |  |  |  |
| Prevalent parasitemia | 330 | 10 | 3.0 | 167 | 7 | 4.2 | 27.7 (-124.0-75.2) | 0.602 |
| Prevalent severe anemia | * | * | * | * | * | * |  |  |
| Prevalent moderate anemia | 330 | 3 | 0.9 | 166 | 3 | 1.8 | 49.7 (-276.0-93.3) | 0.407 |
| **Agogo** |  |  |  |  |  |  |  |  |
| Prevalent parasitemia | 354 | 27 | 7.6 | 184 | 25 | 13.6 | 43.9 (-0.8-68.6) | 0.031 |
| Prevalent severe anemia | * | * | * | * | * | * |  |  |
| Prevalent moderate anemia | 354 | 2 | 0.6 | 184 | 2 | 1.1 | 48.0 (-617.0-96.2) | 0.609 |
| **Kombewa** |  |  |  |  |  |  |  |  |
| Prevalent parasitemia | 514 | 63 | 12.3 | 266 | 45 | 16.9 | 27.5 (-8.7-51.4) | 0.080 |
| Prevalent severe anemia | * | * | * | * | * | * |  |  |
| Prevalent moderate anemia | 516 | 15 | 2.9 | 266 | 11 | 4.1 | 29.7 (-69.2-69.8) | 0.401 |
| **Kintampo** |  |  |  |  |  |  |  |  |
| Prevalent parasitemia | 554 | 78 | 14.1 | 275 | 56 | 20.4 | 30.9 (0.7-51.6) | 0.027 |
| Prevalent severe anemia | * | * | * | * | * | * |  |  |
| Prevalent moderate anemia | 555 | 43 | 7.8 | 275 | 17 | 6.2 | -25.3 (-134.0-30.0) | 0.477 |
| **Nanoro** |  |  |  |  |  |  |  |  |
| Prevalent parasitemia | 354 | 35 | 9.9 | 182 | 40 | 22.0 | 55.0 (27.4-72.3) | <0.001 |
| Prevalent severe anemia | * | * | * | * | * | * |  |  |
| Prevalent moderate anemia | 354 | 9 | 2.5 | 182 | 12 | 6.6 | 61.4 (0.3-85.7) | 0.032 |
| **Siaya** |  |  |  |  |  |  |  |  |
| Prevalent parasitemia | 417 | 79 | 18.9 | 213 | 40 | 18.8 | -0.9 (-51.5-31.9) | 1.000 |
| Prevalent severe anemia | * | * | * | * | * | * |  |  |
| Prevalent moderate anemia | 416 | 9 | 2.2 | 213 | 9 | 4.2 | 48.8 (-45.6-82.0) | 0.204 |
| **Overall** |  |  |  |  |  |  |  |  |
| Prevalent parasitemia | 4140 | 306 | 7.4 | 2100 | 224 | 10.7 | 30.7 (17.3-41.9) | <0.001 |
| Prevalent severe anemia | 4139 | 4 | 0.1 | 2097 | 1 | 0.0 | -102.7 (-9880.0-79.9) | 0.669 |
| Prevalent moderate anemia | 4139 | 113 | 2.7 | 2097 | 65 | 3.1 | 11.9 (-21.4-35.6) | 0.421 |
| **Hemoglobin levels (g/dL)**  **Per-protocol population** | **N** | **Mean** | **SD** | **N** | **Mean** | **SD** |  | **p-value** |
| Kilifi | 300 | 10.9 | 1.0 | 150 | 10.8 | 1.1 |  | 0.304 |
| Korogwe | 545 | 10.8 | 1.4 | 271 | 10.8 | 1.4 |  | 0.576 |
| Manhiça | - | - | - | - | - | - |  | - |
| Lambarene | 330 | 10.5 | 2.9 | 172 | 10.4 | 1.2 |  | 0.548 |
| Bagamoyo | 439 | 10.6 | 1.2 | 218 | 10.6 | 1.1 |  | 0.794 |
| Lilongwe | 330 | 11.5 | 1.1 | 166 | 11.2 | 1.2 |  | 0.040 |
| Agogo | 354 | 11.1 | 1.1 | 184 | 10.9 | 1.2 |  | 0.035 |
| Kombewa | 516 | 10.4 | 1.2 | 266 | 10.2 | 1.2 |  | 0.052 |
| Kintampo | 555 | 10.0 | 1.3 | 275 | 10.0 | 1.2 |  | 0.848 |
| Nanoro | 354 | 10.4 | 1.2 | 182 | 10.3 | 1.3 |  | 0.137 |
| Siaya | 416 | 10.7 | 1.4 | 213 | 10.8 | 1.4 |  | 0.772 |
| **Overall** | 4139 | 10.6 | 1.5 | 2097 | 10.6 | 1.3 |  | 0.034 |
| **Intention-to-treat population** | **N** | **n** | **Percent**  **affected** | **N** | **n** | **Percent**  **affected** | **% (95% CI)** | **p-value** |
| **Kilifi** |  |  |  |  |  |  |  |  |
| Prevalent parasitemia | 332 | 1 | 0.3 | 161 | 1 | 0.6 | 51.5 (-3707-99.4) | 0.546 |
| Prevalent severe anemia | * | * | * | * | * | * |  | . |
| Prevalent moderate anemia | 328 | 4 | 1.2 | 159 | 2 | 1.3 | 3.0 (-972-86.1) | 1.000 |
| **Korogwe** |  |  |  |  |  |  |  |  |
| Prevalent parasitemia | 567 | 1 | 0.2 | 278 | 1 | 0.4 | 51.0 (-3749-99.4) | 0.550 |
| Prevalent severe anemia | * | * | * | * | * | * |  | . |
| Prevalent moderate anemia | 567 | 7 | 1.2 | 278 | 1 | 0.4 | -243.2 (-15E3-55.9) | 0.283 |
| **Manhiça** |  |  |  |  |  |  |  |  |
| Prevalent parasitemia | 404 | 3 | 0.7 | 214 | 5 | 2.3 | 68.2 (-63.4-95.1) | 0.133 |
| Prevalent severe anemia | * | * | * | * | * | * |  | . |
| Prevalent moderate anemia | 404 | 3 | 0.7 | 214 | 3 | 1.4 | 47.0 (-296-92.9) | 0.422 |
| **Lambarene** |  |  |  |  |  |  |  |  |
| Prevalent parasitemia | 379 | 10 | 2.6 | 196 | 6 | 3.1 | 13.8 (-189-71.6) | 0.792 |
| Prevalent severe anemia | * | * | * | * | * | * |  | . |
| Prevalent moderate anemia | 379 | 12 | 3.2 | 197 | 7 | 3.6 | 10.9 (-167-67.7) | 0.809 |
| **Lilongwe** |  |  |  |  |  |  |  |  |
| Prevalent parasitemia | 477 | 21 | 4.4 | 234 | 8 | 3.4 | -28.8 (-236-45.2) | 0.687 |
| Prevalent severe anemia | * | * | * | * | * | * |  | . |
| Prevalent moderate anemia | 476 | 8 | 1.7 | 233 | 3 | 1.3 | -30.5 (-664-68.7) | 1.000 |
| **Agogo** |  |  |  |  |  |  |  |  |
| Prevalent parasitemia | 381 | 27 | 7.1 | 191 | 25 | 13.1 | 45.9 (2.7-69.8) | 0.021 |
| Prevalent severe anemia | * | * | * | * | * | * |  | . |
| Prevalent moderate anemia | 381 | 2 | 0.5 | 191 | 2 | 1.0 | 49.9 (-592-96.4) | 0.604 |
| **Kombewa** |  |  |  |  |  |  |  |  |
| Prevalent parasitemia | 523 | 66 | 12.6 | 272 | 45 | 16.5 | 23.7 (-14.0-48.6) | 0.132 |
| Prevalent severe anemia | * | * | * | * | * | * |  | . |
| Prevalent moderate anemia | 524 | 17 | 3.2 | 272 | 11 | 4.0 | 19.8 (-89.5-64.6) | 0.549 |
| **Kintampo** |  |  |  |  |  |  |  |  |
| Prevalent parasitemia | 598 | 85 | 14.2 | 300 | 63 | 21.0 | 32.3 (4.7-51.7) | 0.012 |
| Prevalent severe anemia | * | * | * | * | * | * |  | . |
| Prevalent moderate anemia | 599 | 45 | 7.5 | 300 | 18 | 6.0 | -25.2 (-130-29.0) | 0.488 |
| **Nanoro** |  |  |  |  |  |  |  |  |
| Prevalent parasitemia | 356 | 35 | 9.8 | 183 | 40 | 21.9 | 55.0 (27.4-72.3) | <0.001 |
| Prevalent severe anemia | * | * | * | * | * | * |  | . |
| Prevalent moderate anemia | 356 | 9 | 2.5 | 183 | 12 | 6.6 | 61.4 (0.3-85.7) | 0.032 |
| **Siaya** |  |  |  |  |  |  |  |  |
| Prevalent parasitemia | 442 | 84 | 19.0 | 222 | 41 | 18.5 | -2.9 (-53.4-30.0) | 0.916 |
| Prevalent severe anemia | * | * | * | * | * | * |  | . |
| Prevalent moderate anemia | 441 | 10 | 2.3 | 222 | 10 | 4.5 | 49.7 (-34.8-81.2) | 0.147 |
| **Overall** |  |  |  |  |  |  |  |  |
| Prevalent parasitemia | 4987 | 338 | 6.8 | 2508 | 238 | 9.5 | 28.6 (15.3-39.7) | <0.001 |
| Prevalent severe anemia | 4983 | 4 | 0.08 | 2506 | 2 | 0.08 | -0.6 (-1012-85.6) | 1.000 |
| Prevalent moderate anemia | 4983 | 133 | 2.7 | 2506 | 74 | 3.0 | 9.6 (-21.8-32.5) | 0.501 |
| **Hemoglobin levels (g/dL)**  **Intention-to-treat population** | **N** | **Mean** | **SD** | **N** | **Mean** | **SD** |  | **p-value** |
| Kilifi | 328 | 10.9 | 1.1 | 159 | 10.8 | 1.1 |  | 0.519 |
| Korogwe | 567 | 10.8 | 1.3 | 278 | 10.9 | 1.4 |  | 0.307 |
| Manhiça | 404 | 10.3 | 1.1 | 214 | 10.5 | 2.8 |  | 0.394 |
| Lambarene | 379 | 10.5 | 2.8 | 197 | 10.3 | 1.2 |  | 0.372 |
| Bagamoyo | 528 | 10.6 | 1.2 | 257 | 10.6 | 1.1 |  | 0.988 |
| Lilongwe | 476 | 11.4 | 1.2 | 233 | 11.3 | 1.2 |  | 0.307 |
| Agogo | 381 | 11.1 | 1.1 | 191 | 10.9 | 1.2 |  | 0.033 |
| Kombewa | 524 | 10.4 | 1.2 | 272 | 10.2 | 1.2 |  | 0.071 |
| Kintampo | 599 | 10.0 | 1.3 | 300 | 10.0 | 1.2 |  | 0.969 |
| Nanoro | 356 | 10.4 | 1.1 | 183 | 10.3 | 1.3 |  | 0.155 |
| Siaya | 441 | 10.7 | 1.4 | 222 | 10.7 | 1.4 |  | 0.932 |
| **Overall** | 4983 | 10.6 | 1.4 | 2506 | 10.6 | 1.5 |  | 0.127 |

* Cases of severe anemia are not provided by site to maintain the blinding of the trial.

Study sites are ordered from lowest (Kilifi) to highest (Siaya) incidence of clinical malaria, defined as a measured or reported fever within previous 24h and parasite density >0 parasites per cubic millimeter (i.e. clinical malaria secondary case definition), measured in control infants 6-12 weeks of age at enrollment during 12 months of follow-up.

N = number of subjects included in each group (without missing values).

n = number of subjects reporting at least one event in each group.

Percent affected = percentage of subjects reporting at least one event.

95% CI = Lower (LL) and upper (UL) confidence limits of 95% confidence interval.

SD = standard derivation.

P-value = two-sided Fisher Exact test for prevalence data, Student 2-sample Test for hemoglobin levels.

Prevalent parasitemia was defined as a documented *P. falciparum* asexual parasite at a density of > 0 identified at a cross sectional survey performed at 18 months post dose-3.

Prevalent severe anemia was defined as a documented hemoglobin concentration < 5.0 g per deciliter identified at a cross sectional survey performed at 18 months post dose-3.

Prevalent moderate anemia was defined as a documented hemoglobin concentration < 8.0 g per deciliter identified at a cross sectional survey performed at 18 months post dose-3.

## Supplementary table 7b. Protective efficacy against the prevalence of *P. falciparum* parasitemia and anemia at 18 months post dose-3 in the 6-12 weeks age category

|  | **RTS,S/AS01 vaccine** | | | **Control vaccine** | | | **Protective Efficacy** | |
| --- | --- | --- | --- | --- | --- | --- | --- | --- |
| **Per-protocol population** | **N** | **N** | **Percent**  **affected** | **N** | **n** | **Percent**  **affected** | **% (95% CI)** | **p-value** |
| **Kilifi** |  |  |  |  |  |  |  |  |
| Prevalent parasitemia | 173 | *4* | *-* | 97 | *4* | *-* |  |  |
| Prevalent severe anemia | 171 | *1* | *-* | 94 | *1* | *-* |  |  |
| Prevalent moderate anemia | 171 | 3 | 1.8 | 94 | 5 | 5.3 | 67.0 (-69.5-94.9) | 0.136 |
| **Korogwe** |  |  |  |  |  |  |  |  |
| Prevalent parasitemia | 342 | *1* | *-* | 167 | *1* | *-* |  |  |
| Prevalent severe anemia | 342 | *0* | *-* | 167 | *0* | *-* |  |  |
| Prevalent moderate anemia | 342 | 10 | 2.9 | 167 | 5 | 3.0 | 2.3 (-264.0-69.6) | 1.000 |
| **Manhiça** |  |  |  |  |  |  |  |  |
| Prevalent parasitemia | 284 | 5 | 1.8 | 129 | 3 | 2.3 | 24.3 (-388.0-85.3) | 0.709 |
| Prevalent severe anemia | 285 | *0* | *-* | 129 | *0* | *-* |  |  |
| Prevalent moderate anemia | 285 | 4 | 1.4 | 129 | 2 | 1.5 | 9.5 (-901.0-87.0) | 1.000 |
| **Lambarene** |  |  |  |  |  |  |  |  |
| Prevalent parasitemia | 138 | 6 | 4.3 | 55 | 2 | 3.6 | -19.6 (-1111.0-78.6) | 1.000 |
| Prevalent severe anemia | 138 | *0* | *-* | 55 | *0* | *-* |  |  |
| Prevalent moderate anemia | 138 | 7 | 5.1 | 55 | 2 | 3.6 | -39.5 (-1276.0-73.4) | 1.000 |
| **Bagamoyo** |  |  |  |  |  |  |  |  |
| Prevalent parasitemia | 460 | *3* | *-* | 231 | *3* | *-* |  |  |
| Prevalent severe anemia | 460 | *0* | *-* | 231 | *0* | *-* |  |  |
| Prevalent moderate anemia | 460 | 9 | 2.0 | 231 | 14 | 6.1 | 67.7 (19.9-87.7) | 0.006 |
| **Lilongwe** |  |  |  |  |  |  |  |  |
| Prevalent parasitemia | 472 | 38 | 8.1 | 247 | 15 | 6.1 | -32.6 (-159.0-28.8) | 0.370 |
| Prevalent severe anemia | 472 | *0* | *-* | 247 | *0* | *-* |  |  |
| Prevalent moderate anemia | 472 | 14 | 3.0 | 247 | 4 | 1.6 | -83.2 (-664.0-42.5) | 0.324 |
| **Agogo** |  |  |  |  |  |  |  |  |
| Prevalent parasitemia | 401 | 34 | 8.5 | 213 | 21 | 9.9 | 14.0 (-55.9-51.5) | 0.556 |
| Prevalent severe anemia | 401 | *0* | *-* | 213 | *0* | *-* |  |  |
| Prevalent moderate anemia | 401 | 9 | 2.2 | 213 | 7 | 3.3 | 31.7 (-116.0-77.4) | 0.436 |
| **Kombewa** |  |  |  |  |  |  |  |  |
| Prevalent parasitemia | 350 | 43 | 12.3 | 159 | 18 | 11.3 | -8.5 (-99.9-38.7) | 0.883 |
| Prevalent severe anemia | 350 | *0* | *-* | 161 | *0* | *-* |  |  |
| Prevalent moderate anemia | 350 | 18 | 5.1 | 161 | 4 | 2.5 | -107.0 -741.0-31.8) | 0.240 |
| **Kintampo** |  |  |  |  |  |  |  |  |
| Prevalent parasitemia | 182 | 26 | 14.3 | 86 | 13 | 15.1 | 5.5 (-100.0-53.2) | 0.854 |
| Prevalent severe anemia | 182 | *0* | *-* | 86 | *0* | *-* |  |  |
| Prevalent moderate anemia | 182 | 20 | 11.0 | 86 | 7 | 8.1 | -35.0 (-278.0-45.2) | 0.522 |
| **Nanoro** |  |  |  |  |  |  |  |  |
| Prevalent parasitemia | 384 | 39 | 10.2 | 191 | 21 | 11.0 | 7.6 (-65.3-47.0) | 0.773 |
| Prevalent severe anemia | 385 | *0* | *-* | 191 | *0* | *-* |  |  |
| Prevalent moderate anemia | 385 | 19 | 4.9 | 191 | 11 | 5.8 | 14.3 (-99.3-61.2) | 0.692 |
| **Siaya** |  |  |  |  |  |  |  |  |
| Prevalent parasitemia | 385 | 52 | 13.5 | 191 | 40 | 20.9 | 35.5 (0.0-58.1) | 0.029 |
| Prevalent severe anemia | 385 | *0* | *-* | 191 | *0* | *-* |  |  |
| Prevalent moderate anemia | 385 | 16 | 4.2 | 191 | 8 | 4.2 | 0.8 (-168.0-59.9) | 1.000 |
| **Overall** |  |  |  |  |  |  |  |  |
| Prevalent parasitemia | 3571 | 247 | 6.9 | 1766 | 140 | 7.9 | 12.7 (-8.1-29.4) | 0.178 |
| Prevalent severe anemia | 3571 | *1* | *-* | 1765 | *1* | *-* | ***** | * |
| Prevalent moderate anemia | 3571 | 129 | 3.6 | 1765 | 69 | 3.9 | 7.6 (-25.6-31.5) | 0.590 |
| **Hemoglobin levels (g/dL)**  **Per-protocol population** | **N** | **Mean** | **SD** | **N** | **Mean** | **SD** |  | **p-value** |
| Kilifi | 171 | 10.0 | 1.1 | 94 | 9.9 | 1.3 |  | 0.520 |
| Korogwe | 342 | 10.2 | 1.2 | 167 | 18.0 | 101.4 |  | 0.155 |
| Manhiça | 285 | 10.2 | 1.8 | 129 | 10.2 | 1.3 |  | 0.864 |
| Lambarene | 138 | 9.9 | 1.1 | 55 | 9.9 | 1.0 |  | 0.955 |
| Bagamoyo | 460 | 10.3 | 1.5 | 231 | 10.2 | 2.1 |  | 0.448 |
| Lilongwe | 472 | 10.8 | 1.3 | 247 | 10.8 | 1.3 |  | 0.917 |
| Agogo | 401 | 10.6 | 1.1 | 213 | 10.7 | 1.2 |  | 0.695 |
| Kombewa | 350 | 10.1 | 1.2 | 161 | 10.2 | 1.0 |  | 0.490 |
| Kintampo | 182 | 9.7 | 1.3 | 86 | 9.6 | 1.3 |  | 0.674 |
| Nanoro | 385 | 10.0 | 1.1 | 191 | 10.0 | 1.1 |  | 0.939 |
| Siaya | 385 | 10.3 | 1.3 | 191 | 10.3 | 1.4 |  | 0.776 |
| **Overall** | 3571 | 10.3 | 1.3 | 1765 | 11.0 | 31.2 |  | 0.159 |
| **Intention-to-treat population** | **N** | **n** | **Percent**  **affected** | **N** | **n** | **Percent**  **affected** | **% (95% CI)** | **p-value** |
| **Kilifi** |  |  |  |  |  |  |  |  |
| Prevalent parasitemia | 178 | *4* | *-* | 98 | *4* | *-* |  |  |
| Prevalent severe anemia | 176 | *1* | *-* | 95 | *1* | *-* |  |  |
| Prevalent moderate anemia | 176 | 3 | 1.7 | 95 | 5 | 5.3 | 67.6 (-66.5-95.0) | 0.133 |
| **Korogwe** |  |  |  |  |  |  |  |  |
| Prevalent parasitemia | 353 | *1* | *-* | 171 | *1* | *-* |  |  |
| Prevalent severe anemia | 353 | *0* | *-* | 172 | *0* | *-* |  |  |
| Prevalent moderate anemia | 353 | 10 | 2.8 | 172 | 5 | 2.9 | 2.5 (-263-69.7) | 1.000 |
| **Manhiça** |  |  |  |  |  |  |  |  |
| Prevalent parasitemia | 285 | 5 | 1.8 | 129 | 3 | 2.3 | 24.6 (-386-85.3) | 0.708 |
| Prevalent severe anemia | 286 | *0* | *-* | 129 | *0* | *-* |  |  |
| Prevalent moderate anemia | 286 | 4 | 1.4 | 129 | 2 | 1.6 | 9.8 (-897-87.1) | 1.000 |
| **Lambarene** |  |  |  |  |  |  |  |  |
| Prevalent parasitemia | 141 | 6 | 4.3 | 58 | 3 | 5.2 | 17.7 (-408-82.4) | 0.721 |
| Prevalent severe anemia | 141 | *0* | *-* | 58 | *0* | *-* |  |  |
| Prevalent moderate anemia | 141 | 7 | 5.0 | 58 | 3 | 5.2 | 4.0 (-475-78.1) | 1.000 |
| **Bagamoyo** |  |  |  |  |  |  |  |  |
| Prevalent parasitemia | 472 | *6* | *-* | 244 | *6* | *-* |  |  |
| Prevalent severe anemia | 472 | *0* | *-* | 244 | *0* | *-* |  |  |
| Prevalent moderate anemia | 472 | 9 | 1.9 | 244 | 16 | 6.6 | 70.9 (30.1-88.7) | 0.002 |
| **Lilongwe** |  |  |  |  |  |  |  |  |
| Prevalent parasitemia | 492 | 40 | 8.1 | 261 | 16 | 6.1 | -32.6 (-154-27.4) | 0.381 |
| Prevalent severe anemia | 492 | *0* | *-* | 261 | *0* | *-* |  |  |
| Prevalent moderate anemia | 492 | 14 | 2.8 | 261 | 4 | 1.5 | -85.7 (-675-41.7) | 0.323 |
| **Agogo** |  |  |  |  |  |  |  |  |
| Prevalent parasitemia | 425 | 36 | 8.5 | 216 | 21 | 9.7 | 12.9 (-57.1-50.5) | 0.659 |
| Prevalent severe anemia | 425 | *0* | *-* | 216 | *0* | *-* |  |  |
| Prevalent moderate anemia | 425 | 10 | 2.4 | 216 | 7 | 3.2 | 27.4 (-125-75.1) | 0.604 |
| **Kombewa** |  |  |  |  |  |  |  |  |
| Prevalent parasitemia | 358 | 45 | 12.6 | 163 | 18 | 11.0 | -13.8 (-109-35.4) | 0.666 |
| Prevalent severe anemia | 358 | *0* | *-* | 165 | *0* | *-* |  |  |
| Prevalent moderate anemia | 358 | 18 | 5.0 | 165 | 4 | 2.4 | -107.4 (-743-31.7) | 0.240 |
| **Kintampo** |  |  |  |  |  |  |  |  |
| Prevalent parasitemia | 198 | 27 | 13.6 | 94 | 13 | 13.8 | 1.4 (-108-50.8) | 1.000 |
| Prevalent severe anemia | 198 | *0* | *-* | 94 | *0* | *-* |  |  |
| Prevalent moderate anemia | 198 | 21 | 10.6 | 94 | 7 | 7.4 | -42.4 (-297-41.7) | 0.524 |
| **Nanoro** |  |  |  |  |  |  |  |  |
| Prevalent parasitemia | 385 | 39 | 10.1 | 192 | 21 | 10.9 | 7.4 (-65.7-46.8) | 0.773 |
| Prevalent severe anemia | 386 | *0* | *-* | 192 | *0* | *-* |  |  |
| Prevalent moderate anemia | 386 | 19 | 4.9 | 192 | 11 | 5.7 | 14.1 (-99.8-61.1) | 0.693 |
| **Siaya** |  |  |  |  |  |  |  |  |
| Prevalent parasitemia | 426 | 60 | 14.1 | 215 | 45 | 20.9 | 32.7 (-1.4-55.0) | 0.031 |
| Prevalent severe anemia | 426 | *1* | *-* | 215 | *1* | *-* |  |  |
| Prevalent moderate anemia | 426 | 18 | 4.2 | 215 | 10 | 4.7 | 9.2 (-120-60.2) | 0.838 |
| **Overall** |  |  |  |  |  |  |  |  |
| Prevalent parasitemia | 3713 | 262 | 7.1 | 1841 | 147 | 8.0 | 11.6 (-8.9-28.1) | 0.229 |
| Prevalent severe anemia | 3713 | 1 | 0.03 | 1841 | 1 | 0.05 | 50.4 (-3792-99.4) | 0.553 |
| Prevalent moderate anemia | 3713 | 133 | 3.6 | 1841 | 74 | 4.0 | 10.9 (-20.1-33.4) | 0.409 |
| **Hemoglobin levels (g/dL)**  **Intention-to-treat population** | **N** | **Mean** | **SD** | **N** | **Mean** | **SD** |  | **p-value** |
| Kilifi | 176 | 10.0 | 1.1 | 95 | 9.9 | 1.3 |  | 0.473 |
| Korogwe | 353 | 10.2 | 1.2 | 172 | 17.8 | 100 |  | 0.155 |
| Manhiça | 286 | 10.2 | 1.8 | 129 | 10.2 | 1.3 |  | 0.858 |
| Lambarene | 141 | 9.9 | 1.1 | 58 | 9.8 | 1.0 |  | 0.653 |
| Bagamoyo | 472 | 10.3 | 1.5 | 244 | 10.2 | 2.1 |  | 0.631 |
| Lilongwe | 492 | 10.8 | 1.3 | 261 | 10.8 | 1.3 |  | 0.999 |
| Agogo | 425 | 10.6 | 1.1 | 216 | 10.7 | 1.2 |  | 0.646 |
| Kombewa | 358 | 10.1 | 1.2 | 165 | 10.2 | 1.0 |  | 0.410 |
| Kintampo | 198 | 9.8 | 1.3 | 94 | 9.7 | 1.2 |  | 0.574 |
| Nanoro | 386 | 10.0 | 1.1 | 192 | 10.0 | 1.1 |  | 0.860 |
| Siaya | 426 | 10.3 | 1.3 | 215 | 10.2 | 1.4 |  | 0.417 |
| **Overall** | 3713 | 10.3 | 1.3 | 1841 | 11.0 | 30.6 |  | 0.169 |

Study sites are ordered from lowest (Kilifi) to highest (Siaya) incidence of clinical malaria, defined as a measured or reported fever within previous 24h and parasite density >0 parasites per cubic millimeter (i.e. clinical malaria secondary case definition), measured in control infants 6-12 weeks of age at enrollment during 12 months of follow-up.

N = number of subjects included in each group (without missing values).

n = number of subjects reporting at least one event in each group.

Percent affected = percentage of subjects reporting at least one event.

95% CI = Lower (LL) and upper (UL) confidence limits of 95% confidence interval.

SD = standard derivation.

P-value = two-sided Fisher Exact test for prevalence data, Student 2-sample Test for hemoglobin levels.

Prevalent parasitemia was defined as a documented *P. falciparum* asexual parasite at a density of > 0 identified at a cross sectional survey performed at 18 months post dose-3.

Prevalent severe anemia was defined as a documented hemoglobin concentration < 5.0 g per deciliter identified at a cross sectional survey performed at 18 months post dose-3.

Prevalent moderate anemia was defined as a documented hemoglobin concentration < 8.0 g per deciliter identified at a cross sectional survey performed at 18 months post dose-3.
