# Supplementary material for: Efficacy and Safety of the RTS,S/AS01 Malaria Vaccine during 18 Months after Vaccination: A Phase 3 Randomized, Controlled Trial in Children and Young Infants at 11 African Sites
Source: PLoS Med. 2014 Jul 29;11(7):e1001685. doi: 10.1371/journal.pmed.1001685 (PMC4114488; doi:10.1371/journal.pmed.1001685)
Supplement: Table S8 — Cases of clinical malaria, severe malaria, malaria hospitalization, and all-cause hospitalization averted per 1,000 infants or children vaccinated with RTS,S/AS01 by 6-mo periods and overall in the 5–17-mo and 6–12-wk age categories, ordered by increasing malaria incidence at each site. (DOCX) [file pmed.1001685.s017.docx]

## Supplementary table 8a. Cases of clinical malaria, severe malaria, malaria hospitalization and all-cause hospitalization averted per 1000 children vaccinated with RTS,S/AS01 by 6-month periods and overall in the 5-17 months age category ordered by increasing malaria incidence

|  | **Children 5-17 months of age at enrollment**  **(per-protocol population)** | | | | **Children 5-17 months of age at enrollment**  **(intention-to-treat population)** | | | |
| --- | --- | --- | --- | --- | --- | --- | --- | --- |
| **Study site** | **Cases averted [1-6 months]** | **Cases averted [7-12 months]** | **Cases averted [13-18 months]** | **Total**  **Cases averted** | **Cases averted [1-6 months]** | **Cases averted [7-12 months]** | **Cases averted [13-18 months]** | **Total**  **Cases averted** |
| **Clinical malaria**  **primary case definition** |  |  |  |  |  |  |  |  |
| Kilifi | 23.6 | 21.0 | -3.2 | 41.5 | 22.5 | 17.5 | -5.1 | 35.0 |
| Korogwe | 8.4 | 40.6 | 44.7 | 93.7 | 14.8 | 39.4 | 44.6 | 98.8 |
| Manhiça | - | - | - | - | 35.3 | 8.6 | 30.9 | 74.8 |
| Lambarene | 94.2 | 13.1 | 19.6 | 126.9 | 106.1 | 22.1 | 20.1 | 148.3 |
| Bagamoyo | 122.3 | 114.5 | 5.2 | 242.0 | 94 | 124.9 | 16.5 | 235.4 |
| Lilongwe | 81.6 | 31.8 | 64.0 | 177.4 | 111.1 | 59.9 | 74.5 | 245.4 |
| Agogo | 346.0 | 334.7 | 133.9 | 814.6 | 420.5 | 333.4 | 141.2 | 895.0 |
| Kombewa | 522.7 | 245.3 | 127.2 | 895.2 | 577.4 | 233.5 | 144.3 | 955.1 |
| Kintampo | 395.7 | 447.2 | 273.4 | 1116.3 | 421.4 | 402.4 | 262.2 | 1086.0 |
| Nanoro | 301.1 | 894.6 | 142.5 | 1338.2 | 436.6 | 850.1 | 136.0 | 1422.6 |
| Siaya | 825.7 | 504.4 | 325.5 | 1655.5 | 986.3 | 443.4 | 262.0 | 1691.7 |
| Average across sites | 286.0 | 258.5 | 121.1 | 665.6 | 286.4 | 209.8 | 104.8 | 601.0 |
| **Clinical malaria**  **secondary case definition** |  |  |  |  |  |  |  |  |
| Kilifi | 23.6 | 30.0 | -6.3 | 47.3 | 22.5 | 22.5 | -7.6 | 37.4 |
| Korogwe | 16.9 | 49.0 | 39.3 | 105.2 | 32.9 | 47.7 | 39.6 | 120.2 |
| Manhiça | - | - | - | - | 41.0 | 18.7 | 45 | 104.7 |
| Lambarene | 168.6 | 55.4 | 34.7 | 258.7 | 172.5 | 63.9 | 43.0 | 279.5 |
| Bagamoyo | 139.7 | 151.3 | 21.3 | 312.3 | 113.8 | 137.2 | 26.2 | 277.2 |
| Lilongwe | 106.3 | 53.6 | 131.1 | 291.0 | 127.3 | 87.1 | 130.8 | 345.2 |
| Agogo | 495.0 | 515.7 | 232.4 | 1243.1 | 589.4 | 524.2 | 238.1 | 1351.7 |
| Kombewa | 756.3 | 384.0 | 214.3 | 1354.6 | 788.9 | 352.4 | 222.1 | 1363.4 |
| Kintampo | 640.3 | 615.6 | 346.3 | 1602.1 | 671.34 | 561.6 | 313.1 | 1546.1 |
| Nanoro | 361.8 | 1199.8 | 185.1 | 1746.7 | 548.1 | 1141.3 | 175.9 | 1865.2 |
| Siaya | 1166.1 | 804.3 | 386.0 | 2356.4 | 1346.0 | 715.2 | 303.5 | 2364.7 |
| Average across sites | 404.2 | 371.2 | 165.4 | 940.8 | 391.1 | 298.3 | 139.4 | 828.9 |
| **Severe malaria**  **primary case definition** |  |  |  |  |  |  |  |  |
| Kilifi | 0 | 0 | 0 | 0 | 0 | 0 | 0 | 0 |
| Korogwe | 0 | 1.7 | 1.7 | 3.4 | 0 | 4.9 | 1.6 | 6.6 |
| Manhiça | - | - | - | - | 3.0 | 3.0 | 2.6 | 8.5 |
| Lambarene | 5.1 | 5.1 | 15.8 | 26.0 | 6.3 | 4.2 | 12.6 | 23.1 |
| Bagamoyo | 8.5 | 2.2 | 4.5 | 15.3 | 11.7 | 1.7 | 3.4 | 16.9 |
| Lilongwe | 5.5 | 2.7 | -6.0 | 2.2 | 3.9 | 3.8 | -5.4 | 2.3 |
| Agogo | 15.7 | -0.4 | 2.4 | 17.7 | 12.7 | -2.2 | 0.4 | 10.9 |
| Kombewa | 20.9 | 16.2 | 2.8 | 39.9 | 18.1 | 14.6 | 1.1 | 33.8 |
| Kintampo | 6.9 | 8.8 | -3.1 | 12.7 | 3.2 | 7.7 | -5.9 | 5.0 |
| Nanoro | 2.5 | 4.5 | 0.0 | 6.9 | 7.4 | 4.3 | 0 | 11.7 |
| Siaya | 34.9 | 10.8 | -12.0 | 33.7 | 47.5 | 4.4 | -9.9 | 42 |
| Average across sites | 10.7 | 5.9 | 0.5 | 17.0 | 10.4 | 4.8 | -0.1 | 15.1 |
| **Severe malaria**  **secondary case definition** |  |  |  |  |  |  |  |  |
| Kilifi | 0 | 0 | 0 | 0 | 0 | 0 | 0 | 0 |
| Korogwe | 0 | 1.7 | 3.4 | 5.1 | 0 | 4.9 | 3.3 | 8.2 |
| Manhiça | - | - | - | - | 3.0 | -0.1 | 8.4 | 11.3 |
| Lambarene | 5.1 | 2.4 | 13.1 | 20.6 | 10.6 | 2.1 | 10.5 | 23.1 |
| Bagamoyo | 12.8 | 2.2 | 4.5 | 19.6 | 15.1 | 1.7 | 3.4 | 20.2 |
| Lilongwe | 2.6 | 5.4 | -12.0 | -3.9 | 5.8 | 5.7 | -12.9 | -1.3 |
| Agogo | 15.5 | 2.2 | -0.4 | 17.4 | 12.7 | 0.3 | -2.0 | 11.0 |
| Kombewa | 27.3 | 12.8 | 1.00 | 41.1 | 24.1 | 11.6 | -0.4 | 35.3 |
| Kintampo | 18.9 | 17.5 | 0.5 | 36.8 | 9.3 | 18.3 | -4.4 | 23.3 |
| Nanoro | 2.5 | -0.9 | 0.0 | 1.6 | 7.4 | -0.7 | 0 | 6.7 |
| Siaya | 42.7 | 12.8 | -12.0 | 43.5 | 52.9 | 6.2 | -9.9 | 49.3 |
| Average across sites | 14.1 | 6.5 | -0.0 | 20.5 | 13.1 | 5.3 | -0.2 | 18.1 |
| **Malaria hospitalization**  **Case definition 1** |  |  |  |  |  |  |  |  |
| Kilifi | 0 | 0 | 0 | 0 | 0 | 0 | 0 | 0 |
| Korogwe | 3.4 | 12.0 | 17.2 | 32.6 | 4.9 | 13.1 | 16.5 | 34.5 |
| Manhiça | - | - | - | - | 13.3 | -0.1 | 12.7 | 25.9 |
| Lambarene | 33.0 | 10.0 | 12.9 | 55.9 | 35.9 | 12.5 | 10.4 | 58.8 |
| Bagamoyo | 21.4 | -2.2 | 9.0 | 28.2 | 20.1 | -1.6 | 8.6 | 27.1 |
| Lilongwe | 2.6 | 2.3 | -12.1 | -7.3 | 13.5 | 4.0 | -8.7 | 8.7 |
| Agogo | 20.8 | 15.3 | 15.7 | 51.8 | 30.3 | 10.7 | 13.3 | 54.3 |
| Kombewa | 25.5 | 20.9 | -3.0 | 43.5 | 27.2 | 18.9 | -3.6 | 42.5 |
| Kintampo | 42.7 | 17.7 | -6.3 | 54.1 | 27.6 | 18.5 | -10.3 | 35.8 |
| Nanoro | 2.5 | -1.2 | 5.3 | 6.5 | 7.4 | -1 | 4.9 | 11.3 |
| Siaya | 98.1 | 4.8 | -10.6 | 92.3 | 132.7 | -0.5 | -12.2 | 120.0 |
| Average across sites | 26.9 | 9.1 | 2.7 | 38.8 | 28.7 | 7.4 | 2.6 | 38.7 |
| **Malaria hospitalization**  **Case definition 2** |  |  |  |  |  |  |  |  |
| Kilifi | 0 | 0 | 0 | 0 | 0 | 0 | 0 | 0 |
| Korogwe | 3.4 | 3.3 | 17.2 | 24.0 | 4.9 | 4.9 | 16.4 | 26.3 |
| Manhiça | - | - | - | - | 19.2 | -0.1 | 15.6 | 34.7 |
| Lambarene | 45.7 | 10.0 | 10.2 | 65.9 | 46.5 | 12.5 | 8.2 | 67.2 |
| Bagamoyo | 21.4 | -4.4 | 13.6 | 30.5 | 21.8 | 0.2 | 12.0 | 34.0 |
| Lilongwe | 8.0 | -0.6 | -21.0 | -13.5 | 23.0 | 2.1 | -10.5 | 14.7 |
| Agogo | 36.5 | 25.8 | 13.0 | 75.3 | 60.6 | 20.9 | 16.0 | 97.4 |
| Kombewa | 33.6 | 14.0 | 2.0 | 49.5 | 31.7 | 12.8 | 0.8 | 45.3 |
| Kintampo | 44.5 | 5.8 | -11.4 | 38.9 | 26.3 | 9.5 | -14.8 | 21.0 |
| Nanoro | -2.7 | -11.9 | 7.8 | -6.8 | 4.7 | -11.1 | 7.3 | 0.9 |
| Siaya | 112.1 | 17.1 | -22.2 | 107.0 | 149.6 | 10.5 | -21.9 | 138.2 |
| Average across sites | 32.1 | 6.4 | 1.1 | 39.5 | 35.0 | 5.9 | 2.4 | 43.2 |
| **All-cause hospitalization**  **Primary case definition** |  |  |  |  |  |  |  |  |
| Kilifi | -8.9 | 2.9 | 5.8 | -0.2 | -7.5 | 2.5 | 2.2 | -2.8 |
| Korogwe | 26.2 | 7.9 | 26.7 | 60.8 | 28.1 | 14.6 | 26.3 | 69.0 |
| Manhiça |  |  |  |  | 18.0 | 11.4 | 36.3 | 65.7 |
| Lambarene | 72.2 | 25.4 | -1.0 | 96.5 | 56.3 | 28.3 | -0.7 | 83.9 |
| Bagamoyo | 24.9 | -6.4 | 22.8 | 41.2 | 11.1 | 12.8 | 8.1 | 32.0 |
| Lilongwe | 45.8 | -3.8 | -12.6 | 29.3 | 65.6 | 2.3 | -2.4 | 65.5 |
| Agogo | 36.0 | 30.7 | 12.8 | 79.5 | 81.4 | 31.5 | 18.8 | 131.6 |
| Kombewa | 36.2 | -3.8 | -8.2 | 24.2 | 19.9 | -5.8 | -7.4 | 6.7 |
| Kintampo | 71.1 | 6.3 | -0.3 | 77.1 | 44.1 | 0.8 | -4 | 40.9 |
| Nanoro | -18.5 | -13.0 | 20.7 | -10.8 | 13.9 | -12.0 | 19.3 | 21.1 |
| Siaya | 56.3 | 1.4 | -18.1 | 39.6 | 87.9 | -6.4 | -17.8 | 63.6 |
| Average across sites | 36.5 | 4.5 | 5.0 | 46.0 | 37.5 | 7.0 | 7.3 | 51.7 |

Cases averted = Cases averted per 1000 children followed-up during the defined follow-up period.

[1-6 months] = 14 days post dose-3 until 6 months post dose-3.

[7-12 months] = 6 months post dose-3 until 12 months post dose-3.

[13-18 months] = 12 months post dose-3 until 18 months post dose-3.

Clinical malaria primary case definition: Illness in a child brought to a study facility with a temperature of ≥ 37.5°C and *P. falciparum* asexual parasitemia at a density of > 5000 parasites per cubic millimeter or a case of malaria meeting the primary case definition of severe malaria.

Clinical malaria secondary case definition: Illness in a child brought to a study facility with a measured temperature of ≥37.5°C or reported fever within the last 24 hours and *P. falciparum* asexual parasitemia at a density of > 0 parasites per cubic millimeter.

Severe malaria primary case definition: *P. falciparum* asexual parasitemia at a density of > 5000 parasites per cubic millimeter with one or more markers of disease severity and without diagnosis of a coexisting illness.

Severe malaria secondary case definition: *P. falciparum* asexual parasitemia at a density of > 5000 parasites per cubic millimeter with one or more markers of disease severity, including cases in which a coexisting illness was present or could not be ruled out.

Markers of severe disease were prostration, respiratory distress, a Blantyre coma score of ≤ 2 (on a scale of 0 to 5, with higher scores indicating a higher level of consciousness), two or more observed or reported seizures, hypoglycemia, acidosis, elevated lactate level, or hemoglobin level of < 5 g per deciliter. Coexisting illnesses were defined as radiographically proven pneumonia, meningitis established by analysis of cerebrospinal fluid, bacteremia, or gastroenteritis with severe dehydration.

Malaria hospitalization case definition 1: A medical hospitalization with confirmed *P. falciparum* asexual parasitemia at a density of > 5000 parasites per cubic millimeter

Malaria hospitalization case definition 2: A hospitalization which, in the judgment of the principal investigator, *P. falciparum* infection was the sole or a major contributing factor to the presentation.

All-cause hospitalization primary case definition: A medical hospitalization of any cause, excluding planned admissions for medical investigation/care or elective surgery and trauma.

Study sites are ordered from lowest (Kilifi) to highest (Siaya) incidence of clinical malaria, defined as a measured or reported fever within previous 24h and parasite density >0 parasites per cubic millimeter (i.e. clinical malaria secondary case definition), measured in control infants 6-12 weeks of age at enrollment during 12 months of follow-up.

## Supplementary table 8b. Cases of clinical malaria, severe malaria, malaria hospitalization and all-cause hospitalization averted per 1000 infants vaccinated with RTS,S/AS01 by 6-month periods and overall in the 6-12 weeks age category ordered by increasing malaria incidence at each site

|  | **Infants 6-12 weeks of age at enrollment  (per-protocol population)** | | | | **Infants 6-12 weeks of age at enrollment**  **(intention-to-treat population)** | | | |
| --- | --- | --- | --- | --- | --- | --- | --- | --- |
| **Study site** | **Cases averted [1-6 months]** | **Cases averted [7-12 months]** | **Cases averted [13-18 months]** | **Total**  **Cases averted** | **Cases averted [1-6 months]** | **Cases averted [7-12 months]** | **Cases averted [13-18 months]** | **Total**  **Cases averted** |
| **Clinical malaria**  **primary case definition** |  |  |  |  |  |  |  |  |
| Kilifi | 0.0 | -1.0 | -18.5 | -19.5 | 0 | -0.7 | -16.4 | -17.1 |
| Korogwe | 3.0 | 23.3 | 11.6 | 37.9 | 0.2 | 21.6 | 15.9 | 37.7 |
| Manhiça | 18.6 | -4.9 | 21.4 | 35.1 | 16.4 | 0.4 | 22.2 | 39.0 |
| Lambarene | 34.9 | -27.0 | 12.0 | 19.8 | 31.4 | -25.9 | 9.5 | 15 |
| Bagamoyo | 31.5 | 42.5 | 19.4 | 93.4 | 36.9 | 36.4 | 26.9 | 100.2 |
| Lilongwe | 99.1 | 93.7 | 82.0 | 274.7 | 91.1 | 85.8 | 53.7 | 230.5 |
| Agogo | 104.0 | 95.6 | 5.4 | 205.0 | 101.9 | 90.5 | 10.8 | 203.1 |
| Kombewa | 282.7 | 222.6 | 20.4 | 525.7 | 283.9 | 212.4 | 26.2 | 522.5 |
| Kintampo | 28.7 | -175.3 | 102.2 | -44.4 | 23.9 | -187.4 | 116.6 | -46.9 |
| Nanoro | 312.7 | 267.1 | 1.5 | 581.3 | 348.8 | 247.8 | 8.9 | 605.4 |
| Siaya | 418.5 | 346.5 | 167.0 | 931.9 | 518.6 | 327.7 | 147.0 | 993.3 |
| Average across sites | 141.1 | 105.1 | 38.8 | 285.0 | 157.2 | 98.5 | 39.0 | 294.7 |
| **Clinical malaria**  **secondary case definition** |  |  |  |  |  |  |  |  |
| Kilifi | -5.4 | 13.4 | -20.4 | -12.4 | -5.1 | 13.2 | -17.7 | -9.6 |
| Korogwe | 6.0 | 40.3 | 20.1 | 66.4 | 2.9 | 37.5 | 23.7 | 64.1 |
| Manhiça | 24.0 | -7.4 | 48.4 | 64.9 | 32.9 | 2.9 | 54 | 89.8 |
| Lambarene | 86.3 | -52.2 | 5.5 | 39.6 | 116.0 | -55.9 | 2.5 | 62.6 |
| Bagamoyo | 44.1 | 34.7 | 24.1 | 102.9 | 46.1 | 30.3 | 40.9 | 117.3 |
| Lilongwe | 172.2 | 128.9 | 112.1 | 413.1 | 164.3 | 111.5 | 81.1 | 356.8 |
| Agogo | 187.7 | 200.4 | -1.6 | 386.4 | 195.4 | 193.3 | 10.2 | 399.0 |
| Kombewa | 434.5 | 249.4 | 172.7 | 856.6 | 472.4 | 215.8 | 156.1 | 844.2 |
| Kintampo | 198.6 | -45.3 | 28.3 | 181.6 | 121.1 | -28.5 | 72.9 | 165.6 |
| Nanoro | 382.5 | 340.6 | 91.3 | 814.4 | 456.3 | 310.8 | 88.6 | 855.7 |
| Siaya | 647.6 | 536.2 | 245.6 | 1429.4 | 718.0 | 468.8 | 215.2 | 1401.9 |
| Average across sites | 217.5 | 155.3 | 71.4 | 444.2 | 237.2 | 140.9 | 71.3 | 449.4 |
| **Severe malaria**  **primary case definition** |  |  |  |  |  |  |  |  |
| Kilifi | 0 | 0 | 0 | 0 | 0 | 0 | 0 | 0 |
| Korogwe | 0 | 0 | -2.73 | -2.73 | 0 | 0 | -2.5 | -2.5 |
| Manhiça | 0 | -5.4 | -5.6 | -11.0 | 0 | -4.7 | -4.7 | -9.4 |
| Lambarene | 0 | 0 | -13.8 | -13.8 | 0 | 0 | -12.7 | -12.7 |
| Bagamoyo | 8.2 | 4.2 | 4.3 | 16.6 | 7.4 | 3.7 | 3.6 | 14.7 |
| Lilongwe | -4.0 | 1.9 | -4.6 | -6.7 | -3.7 | 1.7 | -6.3 | -8.3 |
| Agogo | -2.7 | -0.6 | -7.5 | -10.8 | -2.2 | -0.1 | -6.5 | -8.9 |
| Kombewa | 46.0 | 0.4 | -15.9 | 30.5 | 50.1 | 0.4 | -13.9 | 36.9 |
| Kintampo | 25.1 | -16.6 | 16.6 | 25.2 | 18.2 | -19.0 | 13.9 | 13.1 |
| Nanoro | -4.7 | 18.6 | 2.5 | 16.5 | -2.4 | 17.6 | 2.2 | 17.5 |
| Siaya | 19.9 | 9.9 | -16.3 | 13.5 | 8.7 | 0.3 | -12.8 | -3.8 |
| Average across sites | 7.7 | 2.6 | -4.4 | 5.9 | 6.8 | 1.2 | -4.1 | 3.9 |
| **Severe malaria**  **secondary case definition** |  |  |  |  |  |  |  |  |
| Kilifi | 0 | 0 | 0 | 0 | 0 | 0 | 0 | 0 |
| Korogwe | 0 | 0 | -2.7 | -2.7 | 0 | 0 | -2.5 | -2.5 |
| Manhiça | 0 | -5.4 | -5.6 | -11.0 | 0 | -4.7 | -4.7 | -9.4 |
| Lambarene | 0 | 0 | -13.8 | -13.8 | 0 | 0 | -12.7 | -12.7 |
| Bagamoyo | 8.2 | 4.2 | 8.5 | 20.9 | 7.4 | 3.7 | 7.3 | 18.4 |
| Lilongwe | -0.1 | -2.1 | -2.7 | -4.9 | -0.2 | -2.0 | -4.7 | -6.8 |
| Agogo | -5.1 | -0.6 | -7.5 | -13.2 | -4.4 | -0.1 | -6.5 | -11.0 |
| Kombewa | 46.0 | 5.8 | -18.6 | 33.2 | 50.1 | 5.3 | -18.2 | 37.1 |
| Kintampo | 25.1 | -27.0 | 11.3 | 9.4 | 13.7 | -28.2 | 9.4 | -5.2 |
| Nanoro | -6.9 | 23.3 | 2.5 | 18.9 | -4.6 | 22.1 | 2.2 | 19.7 |
| Siaya | 30.9 | 9.9 | -11.5 | 29.4 | 15.9 | 4.0 | -9.1 | 10.8 |
| Average across sites | 8.9 | 2.6 | -3.6 | 7.9 | 7.5 | 1.6 | -3.6 | 5.5 |
| **Malaria hospitalization**  **Case definition 1** |  |  |  |  |  |  |  |  |
| Kilifi | 0 | 0 | 0 | 0 | 0 | 0 | 0 | 0 |
| Korogwe | -2.6 | 0 | -8.2 | -10.8 | -5.0 | 0 | -7.5 | -12.5 |
| Manhiça | 0 | -8.1 | -5.6 | -13.7 | 0 | -7.1 | -4.7 | -11.8 |
| Lambarene | 16.1 | 25.4 | 2.4 | 44.0 | 29.4 | 23.0 | 1.9 | 54.3 |
| Bagamoyo | 8.2 | 4.2 | 8.5 | 20.9 | 7.4 | 3.7 | 7.3 | 18.4 |
| Lilongwe | 3.8 | -2.1 | -0.7 | 0.9 | 1.5 | -2.0 | -3 | -3.5 |
| Agogo | 1.3 | 1.5 | -14.8 | -12 | -2.3 | 2.1 | -13.0 | -13.3 |
| Kombewa | 58.9 | 6.2 | -26.4 | 38.6 | 62.0 | 3.4 | -24.9 | 40.5 |
| Kintampo | 15.0 | -37.8 | 32.9 | 10.1 | 4.6 | -42.1 | 27.6 | -9.9 |
| Nanoro | -6.9 | 16.2 | 10.0 | 19.3 | -4.6 | 15.4 | 8.9 | 19.7 |
| Siaya | 46.5 | 3.8 | -27.6 | 22.6 | 33.9 | 2.4 | -14.5 | 21.8 |
| Average across sites | 12.9 | 1.3 | -4.9 | 9.4 | 11.3 | 0.5 | -3.8 | 8.0 |
| **Malaria hospitalization**  **Case definition 2** |  |  |  |  |  |  |  |  |
| Kilifi | 0 | 0 | -5.5 | -5.5 | 0 | 0 | -5.1 | -5.1 |
| Korogwe | -2.6 | 0 | 0.2 | -2.5 | -5.0 | 0 | 0.2 | -4.9 |
| Manhiça | 0 | -8.1 | -2.7 | -10.8 | 0 | -7.1 | -2.3 | -9.4 |
| Lambarene | 32.3 | 18.6 | -18.3 | 32.6 | 44.1 | 16.6 | -17.2 | 43.6 |
| Bagamoyo | 8.2 | 2.1 | 8.5 | 18.8 | 7.4 | 1.8 | 11.0 | 20.2 |
| Lilongwe | 3.8 | 1.8 | -2.9 | 2.7 | 1.5 | 1.5 | -5.1 | -2.0 |
| Agogo | 5.8 | -5.7 | -17.2 | -17.2 | -0.2 | -4.5 | -15.2 | -19.8 |
| Kombewa | 71.7 | 14.4 | -34.6 | 51.5 | 74.0 | 10.8 | -26.8 | 58.0 |
| Kintampo | 15.0 | -38.5 | 27.5 | 4.1 | 4.6 | -47.1 | 23.1 | -19.4 |
| Nanoro | -11.6 | 18.6 | 7.5 | 14.5 | -9.2 | 17.7 | 6.6 | 15.1 |
| Siaya | 64.3 | 1.8 | -34.8 | 31.3 | 48.4 | 4.4 | -21.9 | 30.9 |
| Average across sites | 16.7 | 1.3 | -7.5 | 10.5 | 14.5 | 0.7 | -5.4 | 9.8 |
| **All-cause hospitalization**  **Primary case definition** |  |  |  |  |  |  |  |  |
| Kilifi | -2.0 | -38.1 | -17.6 | -57.6 | -7.1 | -40.4 | -15.8 | -63.3 |
| Korogwe | -20.6 | 28.2 | 41.3 | 48.9 | -10.9 | 28.5 | 28.0 | 45.5 |
| Manhiça | -28.8 | 25.0 | -7.8 | -11.7 | -19.5 | 17.1 | -4.5 | -6.9 |
| Lambarene | 67.4 | 44.7 | -66.6 | 45.4 | 77.9 | 39.3 | -61.7 | 55.4 |
| Bagamoyo | 7.7 | 51.1 | 8.9 | 67.7 | 2.9 | 43.6 | 17.4 | 63.9 |
| Lilongwe | 34.3 | -5.1 | -5.9 | 23.4 | 16.3 | -7.3 | -8.3 | 0.8 |
| Agogo | -4.9 | 11.0 | -13.8 | -7.7 | -7 | 12.8 | -10.8 | -4.9 |
| Kombewa | 89.5 | 38.8 | 11.3 | 139.5 | 110.6 | 28.5 | 6.5 | 145.5 |
| Kintampo | -5.5 | -129.9 | 7.4 | -128.0 | -18.0 | -122.4 | -13.0 | -153.3 |
| Nanoro | -26.3 | -5.1 | 7.5 | -23.8 | -8.4 | -4.5 | 6.7 | -6.2 |
| Siaya | 44.8 | 19.0 | -23.5 | 40.4 | 6.2 | 9.4 | 2.3 | 17.9 |
| Average across sites | 12.2 | 9.2 | -2.2 | 19.2 | 10.4 | 5.9 | -0.2 | 16.1 |

Cases averted = Cases averted per 1000 children followed-up during the defined follow-up period.

[1-6 months] = 14 days post dose-3 until 6 months post dose-3.

[7-12 months] = 6 months post dose-3 until 12 months post dose-3.

[13-18 months] = 12 months post dose-3 until 18 months post dose-3.

Clinical malaria primary case definition: Illness in a child brought to a study facility with a temperature of ≥ 37.5°C and *P. falciparum* asexual parasitemia at a density of > 5000 parasites per cubic millimeter or a case of malaria meeting the primary case definition of severe malaria.

Clinical malaria secondary case definition: Illness in a child brought to a study facility with a measured temperature of ≥37.5°C or reported fever within the last 24 hours and *P. falciparum* asexual parasitemia at a density of > 0 parasites per cubic millimeter.

Severe malaria primary case definition: *P. falciparum* asexual parasitemia at a density of > 5000 parasites per cubic millimeter with one or more markers of disease severity and without diagnosis of a coexisting illness.

Severe malaria secondary case definition: *P. falciparum* asexual parasitemia at a density of > 5000 parasites per cubic millimeter with one or more markers of disease severity, including cases in which a coexisting illness was present or could not be ruled out.

Markers of severe disease were prostration, respiratory distress, a Blantyre coma score of ≤ 2 (on a scale of 0 to 5, with higher scores indicating a higher level of consciousness), two or more observed or reported seizures, hypoglycemia, acidosis, elevated lactate level, or hemoglobin level of < 5 g per deciliter. Coexisting illnesses were defined as radiographically proven pneumonia, meningitis established by analysis of cerebrospinal fluid, bacteremia, or gastroenteritis with severe dehydration.

Malaria hospitalization case definition 1: A medical hospitalization with confirmed *P. falciparum* asexual parasitemia at a density of > 5000 parasites per cubic millimeter

Malaria hospitalization case definition 2: A hospitalization which, in the judgment of the principal investigator, *P. falciparum* infection was the sole or a major contributing factor to the presentation.

All-cause hospitalization primary case definition: A medical hospitalization of any cause, excluding planned admissions for medical investigation/care or elective surgery and trauma.

Study sites are ordered from lowest (Kilifi) to highest (Siaya) incidence of clinical malaria, defined as a measured or reported fever within previous 24h and parasite density >0 parasites per cubic millimeter (i.e. clinical malaria secondary case definition), measured in control infants 6-12 weeks of age at enrollment during 12 months of follow-up.
