# Supplementary material for: Efficacy and Safety of the RTS,S/AS01 Malaria Vaccine during 18 Months after Vaccination: A Phase 3 Randomized, Controlled Trial in Children and Young Infants at 11 African Sites
Source: PLoS Med. 2014 Jul 29;11(7):e1001685. doi: 10.1371/journal.pmed.1001685 (PMC4114488; doi:10.1371/journal.pmed.1001685)
Supplement: Table S9 — Vaccine effect on growth in the 5–17-mo and 6–12-wk age categories. (DOCX) [file pmed.1001685.s018.docx]

## Supplementary table 9a. Vaccine effect on growth in the 5-17 months age category

| **Per-protocol population** | **RTS,S/AS01 vaccine** | | | **Control vaccine** | | |  |
| --- | --- | --- | --- | --- | --- | --- | --- |
| **Parameter** | **N** | **Mean** | **SD** | **N** | **Mean** | **SD** | **p-value** |
| Height-for-age z-score | 4557 | -1.6 | 1.0 | 2328 | -1.6 | 1.0 | 0.595 |
| Weight for age z-score | 4557 | -1.0 | 1.0 | 2328 | -1.0 | 0.9 | 0.141 |
| Mid upper arm circumference z-score | 4557 | -0.3 | 0.9 | 2328 | -0.3 | 0.9 | 0.111 |
| **Intention-to treat population** | **RTS,S/AS01 vaccine** | | | **Control vaccine** | | |  |
| **Parameter** | **N** | **Mean** | **SD** | **N** | **Mean** | **SD** | **p-value** |
| Height-for-age z-score | 5949 | -1.6 | 1.0 | 2974 | -1.6 | 1.0 | 0.721 |
| Weight for age z-score | 5949 | -1.0 | 1.0 | 2974 | -1.0 | 1.0 | 0.294 |
| Mid upper arm circumference z-score | 5949 | -0.3 | 0.9 | 2974 | -0.3 | 0.9 | 0.189 |

Length/height, weight and mid-upper arm circumference measured at Month 20 (18 months post dose-3).

N= number of subjects.

SD = Standard deviation.

P-value = Student 2-Sample test.

## Supplementary table 9b. Vaccine effect on growth in the 6-12 weeks age category

| **Per-protocol population** | **RTS,S/AS01 vaccine** | | | | | **Control vaccine** | | | |  |
| --- | --- | --- | --- | --- | --- | --- | --- | --- | --- | --- |
| **Parameter** | **N** | **Mean** | | **SD** | | **N** | | **Mean** | **SD** | **p-value** |
| Height (cm) | 3996 | 79.7 | | 3.5 | | 2007 | | 79.8 | 3.5 | 0.847 |
| Height-for-age z-score | 3996 | -1.7 | | 1.1 | | 2007 | | -1.7 | 1.2 | 0.506 |
| Weight for age z-score | 3996 | -0.9 | | 1.0 | | 2007 | | -0.9 | 1.1 | 0.582 |
| Mid upper arm circumference z-score | 3996 | -0.1 | | 1.0 | | 2007 | | -0.1 | 1.0 | 0.864 |
| **Intention-to treat population** | **RTS,S/AS01 vaccine** | | | | | | **Control vaccine** | | |  |
| **Parameter** | **N** | | **Mean** | | **SD** | | **N** | **Mean** | **SD** | **p-value** |
| Height (cm) | 4358 | 79.8 | | 3.5 | | 2179 | | 79.8 | 3.5 | 0.781 |
| Height-for-age z-score | 4358 | | -1.7 | | 1.1 | | 2179 | -1.7 | 1.2 | 0.441 |
| Weight for age z-score | 4358 | | -0.9 | | 1.0 | | 2179 | -0.9 | 1.0 | 0.684 |
| Mid upper arm circumference z-score | 4358 | | -0.1 | | 1.0 | | 2179 | -0.1 | 1.0 | 0.962 |

Length/height, weight and mid-upper arm circumference measured at Month 20 (18 months post dose-3).

N= number of subjects.

SD = Standard deviation.

P-value = Student 2-Sample test.
