# Supplementary material for: Efficacy and Safety of the RTS,S/AS01 Malaria Vaccine during 18 Months after Vaccination: A Phase 3 Randomized, Controlled Trial in Children and Young Infants at 11 African Sites
Source: PLoS Med. 2014 Jul 29;11(7):e1001685. doi: 10.1371/journal.pmed.1001685 (PMC4114488; doi:10.1371/journal.pmed.1001685)
Supplement: Table S10 — Overall vaccine efficacy against hospitalization with pneumonia or with sepsis, and malaria mortality and all-cause mortality during an 18-mo follow-up period after dose 3 in the 5–17-mo and 6–12-wk age categories. (DOCX) [file pmed.1001685.s019.docx]

## Supplementary table 10a. Overall vaccine efficacy against (a) hospitalization with pneumonia or with sepsis and (b) malaria mortality and all-cause mortality during an 18-month follow-up period post dose-3 in the 5-17 months age category

|  | **RTS,S/AS01 vaccine** | | | **Control vaccine** | | | **Protective Efficacy** | | |
| --- | --- | --- | --- | --- | --- | --- | --- | --- | --- |
| **Per-protocol population** | **N** | **n** | **Proportion affected (%)** | **N** | **n** | **Proportion affected (%)** | **% (95% CI)** | **p-value** | |
| Pneumonia primary case def. | 4557 | 122 | 2.7 | 2328 | 66 | 2.8 | 5.6 (-29.4-30.6) | | 0.696 |
| Sepsis case def. 1 | 4557 | 71 | 1.6 | 2328 | 43 | 1.8 | 15.6 (-26.2-43.0) | | 0.370 |
| Fatal malaria primary case def. | 4553 | 0 | 0.0 | 2327 | 0 | 0.0 |  | | - |
| Fatal malaria secondary case def. | 4557 | 4 | 0.0 | 2328 | 2 | 0.0 | -2.2 (-1030-85.4) | | 1.000 |
| All-cause mortality case def. 1 | 4557 | 40 | 0.8 | 2328 | 22 | 0.9 | -7.1 (-64.1-46.1) | | 0.788 |
| **Intention-to-treat population** | **N** | **n** | **Proportion affected (%)** | **N** | **n** | **Proportion affected (%)** | **% (95% CI)** | | **p-value** |
| Pneumonia primary case def. | 5949 | 197 | 3.3 | 2974 | 107 | 3.6 | 8.0 (-17.6-27.6) | | 0.496 |
| Sepsis case def. 1 | 5949 | 98 | 1.6 | 2974 | 59 | 2.0 | 17.0 (-16.7-40.5) | | 0.267 |
| Fatal malaria primary case def. | 5949 | 0 | 0.0 | 2974 | 0 | 0.0 |  | | - |
| Fatal malaria secondary case def. | 5949 | 6 | 0.0 | 2974 | 2 | 0.0 | -50.0 (-1419-73.2) | | 1.000 |
| All-cause mortality case def. 1 | 5949 | 74 | 1.2 | 2974 | 33 | 1.1 | -12.1 (-74.5-26.6) | | 0.607 |

N = number of subjects included in each group (without missing values).

n = number of subjects reporting at least one event in each group.

Proportion affected = percentage of subjects reporting at least one event.

95% CI = Lower (LL) and upper (UL) confidence limits of 95% confidence interval.

P-value = two-sided Fisher Exact test.

Pneumonia primary case definition: Cough or difficulty breathing (on history) and tachypnea (≥ 50 breaths per minute < 1 year, ≥ 40 breaths per minute ≥ 1year) and lower chest wall indrawing in a hospitalized child.

Sepsis case definition 1: A child with a positive blood culture taken within 72 hours of admission.

Fatal malaria primary case definition: A case of severe malaria meeting the primary case definition of severe malaria with a fatal outcome. Severe malaria primary case definition was defined as *P. falciparum* asexual parasitemia at a density of > 5000 parasites per cubic millimeter with one or more markers of disease severity and without diagnosis of a coexisting illness. Markers of severe disease were prostration, respiratory distress, a Blantyre coma score of ≤ 2 (on a scale of 0 to 5, with higher scores indicating a higher level of consciousness), two or more observed or reported seizures, hypoglycemia, acidosis, elevated lactate level, or hemoglobin level of < 5 g per deciliter. Coexisting illnesses were defined as radiographically proven pneumonia, meningitis established by analysis of cerebrospinal fluid, bacteremia, or gastroenteritis with severe dehydration.

Fatal malaria secondary case definition: A case of severe malaria meeting the secondary case definition of severe malaria with a fatal outcome. Severe malaria secondary case definition: *P. falciparum* asexual parasitemia at a density of > 5000 parasites per cubic millimeter with one or more markers of disease severity, including cases in which a coexisting illness was present or could not be ruled out.

All-cause mortality case definition 1: A fatality of any cause, including mortality in the community and in hospital.

## Supplementary table 10b. Overall vaccine efficacy against (a) hospitalization with pneumonia or with sepsis and (b) malaria mortality and all-cause mortality during an 18-month follow-up period post dose-3 in the 6-12 weeks age category

|  | **RTS,S/AS01 vaccine** | | | **Control vaccine** | | | **Protective Efficacy** | |
| --- | --- | --- | --- | --- | --- | --- | --- | --- |
| **Per-protocol population** | **N** | **n** | **Proportion affected (%)** | **N** | **n** | **Proportion affected (%)** | **% (95% CI)** | **p-value** |
| Pneumonia primary case def. | 3996 | 153 | 3.8 | 2007 | 86 | 4.3 | 10.6 (-17.8-31.8) | 0.401 |
| Sepsis case def. 1 | 3996 | 63 | 1.6 | 2007 | 26 | 1.3 | -21.7 (-100.0-24.1) | 0.429 |
| Fatal malaria primary case def. | 3993 | 0 | 0.0 | 2007 | 0 | 0.0 |  | - |
| Fatal malaria secondary case def. | 3996 | *2* | *-* | 2007 | *2* | *-* | *-* | *-* |
| All-cause mortality case def. 1 | 3996 | 52 | 1.3 | 2007 | 23 | 1.1 | -13.6 (-94.4-31.7) | 0.712 |
| **Intention-to-treat population** | **N** | **n** | **Proportion affected (%)** | **N** | **n** | **Proportion affected (%)** | **% (95% CI)** | **p-value** |
| Pneumonia primary case def. | 4358 | 226 | 5.2 | 2179 | 114 | 5.2 | 0.9 (-25.3-21.2) | 0.952 |
| Sepsis case def. 1 | 4358 | 77 | 1.8 | 2179 | 36 | 1.7 | -6.9 (-63.6-28.9) | 0.763 |
| Fatal malaria primary case def. | 4358 | 0 | 0.0 | 2179 | 0 | 0.0 |  | - |
| Fatal malaria secondary case def. | 4358 | 1 | 0.0 | 2179 | 2 | 0.0 | 75.0 (-380-99.6) | 0.259 |
| All-cause mortality case def. 1 | 4358 | 83 | 1.9 | 2179 | 34 | 1.6 | -22.1 (-87.7-19.0) | 0.373 |

* Sum of the fatal malaria pooled over the two study group to maintain the blinding of the trial.

N = number of subjects included in each group (without missing values).

n = number of subjects reporting at least one event in each group.

Proportion affected = percentage of subjects reporting at least one event.

95% CI = Lower (LL) and upper (UL) confidence limits of 95% confidence interval.

P-value = two-sided Fisher Exact test.

Pneumonia primary case definition: Cough or difficulty breathing (on history) and tachypnea (≥ 50 breaths per minute < 1 year, ≥ 40 breaths per minute ≥ 1year) and lower chest wall indrawing in a hospitalized young infant.

Sepsis case definition 1: A child with a positive blood culture taken within 72 hours of admission.

Fatal malaria primary case definition: A case of severe malaria meeting the primary case definition of severe malaria with a fatal outcome. Severe malaria primary case definition was defined as *P. falciparum* asexual parasitemia at a density of > 5000 parasites per cubic millimeter with one or more markers of disease severity and without diagnosis of a coexisting illness. Markers of severe disease were prostration, respiratory distress, a Blantyre coma score of ≤ 2 (on a scale of 0 to 5, with higher scores indicating a higher level of consciousness), two or more observed or reported seizures, hypoglycemia, acidosis, elevated lactate level, or hemoglobin level of < 5 g per deciliter. Coexisting illnesses were defined as radiographically proven pneumonia, meningitis established by analysis of cerebrospinal fluid, bacteremia, or gastroenteritis with severe dehydration.

Fatal malaria secondary case definition: A case of severe malaria meeting the secondary case definition of severe malaria with a fatal outcome. Severe malaria secondary case definition: *P. falciparum* asexual parasitemia at a density of > 5000 parasites per cubic millimeter with one or more markers of disease severity, including cases in which a coexisting illness was present or could not be ruled out.

All-cause mortality case definition 1: A fatality of any cause, including mortality in the community and in hospital.
