# Supplementary material for: Efficacy and Safety of the RTS,S/AS01 Malaria Vaccine during 18 Months after Vaccination: A Phase 3 Randomized, Controlled Trial in Children and Young Infants at 11 African Sites
Source: PLoS Med. 2014 Jul 29;11(7):e1001685. doi: 10.1371/journal.pmed.1001685 (PMC4114488; doi:10.1371/journal.pmed.1001685)
Supplement: Table S11 — Effect of anti-CS response on the incidence of clinical malaria (primary case definition) in RTS,S/AS01 recipients during an 18-mo follow-up period in the 5–17-mo and 6–12-wk age categories (per-protocol population). (DOCX) [file pmed.1001685.s020.docx]

## Supplementary table 11a. Effect of anti-CS response on the incidence of clinical malaria (primary case definition) in RTS,S/AS01 recipients during an 18-month follow-up period in the 5-17 months age category (per-protocol population)

| **Parameter** | **Incident rate ratio** | **95%CI** | | **p-value** |
| --- | --- | --- | --- | --- |
|  |  | **LL** | **UL** |  |
| Male versus female | 0.97 | 0.79 | 1.19 | 0.787 |
| Anti-CS Positive at Baseline | 1.50 | 1.14 | 1.98 | 0.004 |
| Anti-CS titer at Month 3 | 0.84 | 0.62 | 1.13 | 0.242 |
| Age 5-11 months versus 12-17 months | 0.86 | 0.70 | 1.06 | 0.152 |
| Incidence in controls | 2.32 | 2.08 | 2.59 | <.001 |
| Vitamin A usage | 0.90 | 0.72 | 1.13 | 0.384 |
| Low HAZ versus normal HAZ | 1.06 | 0.82 | 1.37 | 0.636 |
| Low WAZ versus normal WAZ | 0.85 | 0.64 | 1.13 | 0.262 |
| Hepatitis B priming Yes versus No | 1.05 | 0.73 | 1.52 | 0.787 |
| Dispersion | 3.26 | 2.60 | 4.33 | _ |

The model included an intercept (not presented in the table above).

Incident rate ratios >1 corresponds to a factor associated with an increased risk of malaria.

LL = Lower limit of the 95% confidence interval.

UL = Upper limit of the 95% confidence interval.

HAZ = Height-for-age Z-score.

WAZ = Weight-for-age Z-score.

P-value from negative binomial regression.

## Supplementary table 11b. Effect of anti-CS response on the incidence of clinical malaria (primary case definition) in RTS,S/AS01 recipients during an 18-month follow-up period in the 6-12 weeks age category (per-protocol population)

| **Parameter** | **Incident rate ratio** | **95%CI** | | **p-value** |
| --- | --- | --- | --- | --- |
|  |  | **LL** | **UL** |  |
| Male versus female | 1.10 | 0.92 | 1.31 | 0.301 |
| Anti-CS Positive at Baseline | 1.46 | 1.20 | 1.76 | <.001 |
| Anti-CS titer at Month 3 | 0.69 | 0.57 | 0.84 | <.001 |
| Age 6 weeks versus 7-12 weeks | 0.90 | 0.76 | 1.08 | 0.262 |
| Incidence in controls | 3.08 | 2.78 | 3.41 | <.001 |
| Vitamin A usage | 0.72 | 0.46 | 1.13 | 0.154 |
| Low HAZ versus normal HAZ | 1.00 | 0.80 | 1.25 | 0.968 |
| Low WAZ versus normal WAZ | 1.18 | 0.83 | 1.69 | 0.358 |
| Dispersion | 2.47 | 2.08 | 3.05 | _ |

The model included an intercept (not presented in the table above).

Incident rate ratios >1 corresponds to a factor associated with an increased risk of malaria.

LL = Lower limit of the 95% confidence interval.

UL = Upper limit of the 95% confidence interval.

HAZ = Height-for-age Z-score.

WAZ = Weight-for-age Z-score.

P-value from negative binomial regression.
