# Supplementary material for: Efficacy and Safety of the RTS,S/AS01 Malaria Vaccine during 18 Months after Vaccination: A Phase 3 Randomized, Controlled Trial in Children and Young Infants at 11 African Sites
Source: PLoS Med. 2014 Jul 29;11(7):e1001685. doi: 10.1371/journal.pmed.1001685 (PMC4114488; doi:10.1371/journal.pmed.1001685)
Supplement: Table S12 — Determinants of anti-CS response during an 18-mo follow-up period in the 5–17-mo and 6–12-wk age categories, results from linear regression analysis (per-protocol population). (DOCX) [file pmed.1001685.s021.docx]

## Supplementary table 12a. Determinants of anti-CS response during an 18-month follow-up period in the 5-17 months age category, results from linear regression analysis (per-protocol population)

| **Parameter** | **Parameter Estimate** | **Std. Error** | **VIF** | **p-value** |
| --- | --- | --- | --- | --- |
| N | 1057 |  |  |  |
| Root MSE | 0.34 |  |  |  |
| Adjusted R-squared | 0.04 |  |  |  |
| Intercept | 2.68 | 0.05 | 0.00 | <.001 |
| Male versus female | 0.03 | 0.02 | 1.02 | 0.173 |
| Anti-CS Positive at Baseline versus negative | 0.06 | 0.04 | 1.10 | 0.086 |
| Age 5-11 months versus 12-17 months | 0.08 | 0.02 | 1.03 | <.001 |
| Incidence in controls | 0.04 | 0.01 | 1.25 | <.001 |
| Vitamin A usage | -0.02 | 0.02 | 1.10 | 0.425 |
| Low HAZ versus normal HAZ | 0.01 | 0.03 | 1.28 | 0.686 |
| Low WAZ versus normal WAZ | 0.02 | 0.03 | 1.29 | 0.425 |
| Hepatitis B priming Yes versus No | -0.02 | 0.04 | 1.11 | 0.725 |

Positive parameter estimates correspond to factors having a positive correlation with outcome.

Std. Error = Standard Error.

VIF = Variance Inflation Factor.

HAZ = Height-for-age Z-score.

WAZ = Weight-for-age Z-score.

P-value from linear regression.

## Supplementary table 12b. Determinants of anti-CS response during an 18-month follow-up period in the 6-12 weeks age category, results from linear regression analysis (per-protocol population)

| **Parameter** | **Parameter Estimate** | **Std. Error** | **VIF** | **p-value** |
| --- | --- | --- | --- | --- |
| N | 1222 |  |  |  |
| Root MSE | 0.44 |  |  |  |
| Adjusted R-squared | 0.11 |  |  |  |
| Intercept | 2.45 | 0.03 | 0.00 | <.001 |
| Male versus female | 0.03 | 0.03 | 1.01 | 0.278 |
| Anti-CS Positive at Baseline versus negative | -0.31 | 0.03 | 1.12 | <.001 |
| Age 6 weeks versus 7-12 weeks | -0.08 | 0.03 | 1.02 | 0.003 |
| Incidence in controls | -0.02 | 0.01 | 1.14 | 0.101 |
| Vitamin A usage | -0.02 | 0.08 | 1.04 | 0.770 |
| Low HAZ versus normal HAZ | 0.05 | 0.03 | 1.17 | 0.150 |
| Low WAZ versus normal WAZ | -0.05 | 0.05 | 1.17 | 0.292 |

Positive parameter estimates correspond to factors having a positive correlation with outcome.

Std. Error = Standard Error.

VIF = Variance Inflation Factor.

HAZ = Height-for-age Z-score.

WAZ = Weight-for-age Z-score.

P-value from linear regression.
