# Supplementary material for: Efficacy and Safety of the RTS,S/AS01 Malaria Vaccine during 18 Months after Vaccination: A Phase 3 Randomized, Controlled Trial in Children and Young Infants at 11 African Sites
Source: PLoS Med. 2014 Jul 29;11(7):e1001685. doi: 10.1371/journal.pmed.1001685 (PMC4114488; doi:10.1371/journal.pmed.1001685)
Supplement: Table S13 — Univariate analysis of effect of covariates on anti-CS responses 1 mo after dose 3 in RTS,S/AS01 recipients in the 5–17-mo and 6–12-wk age categories (per-protocol population). (DOCX) [file pmed.1001685.s022.docx]

## Supplementary table 13a. Univariate analysis of effect of covariates on anti-CS responses one month post dose-3 in RTS,S/AS01 recipients in the 5-17 months age category (per-protocol population)

| **Parameter** | **Parameter Values** | **N** | **GMT**  **(EU/mL)** | **95% CI** | |
| --- | --- | --- | --- | --- | --- |
|  |  |  |  | **LL** | **UL** |
| Male | No | 499 | 588.5 | 546.7 | 633.3 |
| Male | Yes | 558 | 632.8 | 593.2 | 675.1 |
| Anti-CS Positive at Baseline | No | 935 | 593.6 | 564.8 | 623.9 |
| Anti-CS Positive at Baseline | Yes | 122 | 767.7 | 643.8 | 915.6 |
| Age category | 12-17m | 510 | 555.8 | 519.8 | 594.2 |
| Age category | 5-11m | 547 | 668.4 | 623.4 | 716.7 |
| Vitamin A Usage | No | 640 | 643.9 | 604.1 | 686.3 |
| Vitamin A Usage | Yes | 417 | 564.9 | 524.3 | 608.7 |
| HAZ under -2 | No | 764 | 605.6 | 572.5 | 640.5 |
| HAZ under -2 | Yes | 293 | 627.2 | 568.9 | 691.4 |
| WAZ under -2 | No | 852 | 597.1 | 566.0 | 630.0 |
| WAZ under -2 | Yes | 205 | 674.9 | 601.5 | 757.3 |
| Hepatitis B priming | No | 72 | 737.8 | 619.2 | 879.1 |
| Hepatitis B priming | Yes | 985 | 603.1 | 573.4 | 634.4 |

N = number of subjects included in each group (without missing values).

GMT = Geometric mean titer.

EU/mL = Elisa Unit per milliliter.

HAZ = Height-for-age Z-score.

WAZ = Weight-for-age Z-score.

95% CI = 95% confidence interval; LL = Lower Limit, UL = Upper Limit.

## Supplementary table 13b. Univariate analysis of effect of covariates on anti-CS responses one month post dose-3 in RTS,S/AS01 recipients in the 6-12 weeks age category (per-protocol population)

| **Parameter** | **Parameter Values** | **N** | **GMT**  **(EU/mL)** | **95% CI** | |
| --- | --- | --- | --- | --- | --- |
|  |  |  |  | **LL** | **UL** |
| Male | No | 583 | 204.8 | 187.1 | 224.1 |
| Male | Yes | 639 | 210.8 | 194.3 | 228.8 |
| Anti-CS Positive at Baseline | No | 829 | 264.3 | 248.2 | 281.5 |
| Anti-CS Positive at Baseline | Yes | 393 | 125.4 | 111.3 | 141.2 |
| Age category | 7-12w | 659 | 227.7 | 209.6 | 247.4 |
| Age category | 6w | 563 | 186.9 | 171.2 | 204.2 |
| Vitamin A Usage | No | 1188 | 209.8 | 197.6 | 222.8 |
| Vitamin A Usage | Yes | 34 | 151.8 | 85.46 | 269.5 |
| HAZ under -2 | No | 913 | 203.3 | 188.9 | 218.7 |
| HAZ under -2 | Yes | 309 | 222.4 | 201.0 | 246.0 |
| WAZ under -2 | No | 1126 | 207.9 | 195.1 | 221.7 |
| WAZ under -2 | Yes | 96 | 207.8 | 174.3 | 247.7 |

N = number of subjects included in each group (without missing values).

GMT = Geometric mean titer.

EU/mL = Elisa Unit per milliliter.

HAZ = Height-for-age Z-score.

WAZ = Weight-for-age Z-score.

95% CI = 95% confidence interval; LL = Lower Limit, UL = Upper Limit.
