# Supplementary material for: Efficacy and Safety of the RTS,S/AS01 Malaria Vaccine during 18 Months after Vaccination: A Phase 3 Randomized, Controlled Trial in Children and Young Infants at 11 African Sites
Source: PLoS Med. 2014 Jul 29;11(7):e1001685. doi: 10.1371/journal.pmed.1001685 (PMC4114488; doi:10.1371/journal.pmed.1001685)
Supplement: Table S14 — Percentage of infants/children in the 5–17-mo and 6–12-wk age categories reporting a serious adverse event during 20 mo after dose 1 by MedDRA preferred term (intention-to-treat population). (DOCX) [file pmed.1001685.s023.docx]

## Supplementary table 14a. Percentage of children in the 5-17 months age category reporting a serious adverse event during 20 months post dose-1 by MedDRA Preferred Term (intention-to-treat population)

|  | **RTS,S/AS01 vaccine**  **N=5949** | | | | **Control vaccine**  **N=2974** | | | |
| --- | --- | --- | --- | --- | --- | --- | --- | --- |
|  |  |  | **95% CI** | |  |  | **95% CI** | |
|  | **n** | **%** | **LL** | **UL** | **n** | **%** | **LL** | **UL** |
| At least one SAE | 1108 | 18.6 | 17.6 | 19.6 | 676 | 22.7 | 21.2 | 24.3 |
| At least one SAE excluding Malaria | 1047 | 17.6 | 16.6 | 18.6 | 630 | 21.2 | 19.7 | 22.7 |
| At least one fatal SAE | 74 | 1.2 | 1.0 | 1.6 | 33 | 1.1 | 0.8 | 1.6 |
| At least one related SAE | 10 | 0.2 | 0.1 | 0.3 | 1 | 0.0 | 0.0 | 0.2 |
| **SAE by MedDRA Preferred Term** |  |  |  |  |  |  |  |  |
| Anemia | 190 | 3.2 | 2.8 | 3.7 | 155 | 5.2 | 4.4 | 6.1 |
| Disseminated intravascular coagulation | *1* |  |  |  | *1* |  |  |  |
| Hypochromic anemia | *1* |  |  |  | *1* |  |  |  |
| Leukaemoid reaction | *1* |  |  |  | *1* |  |  |  |
| Lymphadenitis | 4 | 0.1 | 0.0 | 0.2 | 1 | 0.0 | 0.0 | 0.2 |
| Neutropenia | *2* |  |  |  | *2* |  |  |  |
| Pancytopenia | *1* |  |  |  | *1* |  |  |  |
| Cardiac failure | 1 | 0.0 | 0.0 | 0.1 | 1 | 0.0 | 0.0 | 0.2 |
| Atrial septal defect | *1* |  |  |  | *1* |  |  |  |
| Cerebral palsy | *1* |  |  |  | *1* |  |  |  |
| Congenital megacolon | *1* |  |  |  | *1* |  |  |  |
| Glucose-6-phosphate dehydrogenase deficiency | *1* |  |  |  | *1* |  |  |  |
| Sickle cell anemia | *4* |  |  |  | *4* |  |  |  |
| Sickle cell anemia with crisis | 4 | 0.1 | 0.0 | 0.2 | 1 | 0.0 | 0.0 | 0.2 |
| Ventricular septal defect | *2* |  |  |  | *2* |  |  |  |
| Conjunctivitis | *5* |  |  |  | *5* |  |  |  |
| Colitis | *1* |  |  |  | *1* |  |  |  |
| Constipation | *1* |  |  |  | *1* |  |  |  |
| Enteritis | 21 | 0.4 | 0.2 | 0.5 | 12 | 0.4 | 0.2 | 0.7 |
| Food poisoning | *1* |  |  |  | *1* |  |  |  |
| Gastritis | 1 | 0.0 | 0.0 | 0.1 | 1 | 0.0 | 0.0 | 0.2 |
| Gastrointestinal hemorrhage | *1* |  |  |  | *1* |  |  |  |
| Gastrooesophageal reflux disease | *1* |  |  |  | *1* |  |  |  |
| Mouth ulceration | *1* |  |  |  | *1* |  |  |  |
| Stomatitis | *1* |  |  |  | *1* |  |  |  |
| Stress ulcer | *1* |  |  |  | *1* |  |  |  |
| Umbilical hernia | *1* |  |  |  | *1* |  |  |  |
| Umbilical hernia, obstructive | *1* |  |  |  | *1* |  |  |  |
| Upper gastrointestinal hemorrhage | *1* |  |  |  | *1* |  |  |  |
| Death | *4* |  |  |  | *4* |  |  |  |
| Drowning | 4 | 0.1 | 0.0 | 0.2 | 1 | 0.0 | 0.0 | 0.2 |
| Generalized oedema | *1* |  |  |  | *1* |  |  |  |
| Hypothermia | *2* |  |  |  | *2* |  |  |  |
| Injection site reaction | *1* |  |  |  | *1* |  |  |  |
| Pyrexia | 20 | 0.3 | 0.2 | 0.5 | 9 | 0.3 | 0.1 | 0.6 |
| Hepatitis | 2 | 0.0 | 0.0 | 0.1 | 1 | 0.0 | 0.0 | 0.2 |
| Hepatitis acute | *1* |  |  |  | *1* |  |  |  |
| Hepatitis toxic | *1* |  |  |  | *1* |  |  |  |
| Hypersensitivity | *1* |  |  |  | *1* |  |  |  |
| Abscess | 10 | 0.2 | 0.1 | 0.3 | 4 | 0.1 | 0.0 | 0.3 |
| Abscess limb | *2* |  |  |  | *2* |  |  |  |
| Acarodermatitis | *1* |  |  |  | *1* |  |  |  |
| Amoebiasis | *1* |  |  |  | *1* |  |  |  |
| Arthritis bacterial | 4 | 0.1 | 0.0 | 0.2 | 1 | 0.0 | 0.0 | 0.2 |
| Ascariasis | *1* |  |  |  | *1* |  |  |  |
| Bacteremia | *1* |  |  |  | *1* |  |  |  |
| Bacterial infection | *1* |  |  |  | *1* |  |  |  |
| Bronchiolitis | 35 | 0.6 | 0.4 | 0.8 | 18 | 0.6 | 0.4 | 1.0 |
| Bronchitis | 24 | 0.4 | 0.3 | 0.6 | 17 | 0.6 | 0.3 | 0.9 |
| Bronchopneumonia | 55 | 0.9 | 0.7 | 1.2 | 37 | 1.2 | 0.9 | 1.7 |
| Burn infection | *1* |  |  |  | *1* |  |  |  |
| Cellulitis | 9 | 0.2 | 0.1 | 0.3 | 5 | 0.2 | 0.1 | 0.4 |
| Cellulitis pharyngeal | *1* |  |  |  | *1* |  |  |  |
| Cerebral malaria | *3* |  |  |  | *3* |  |  |  |
| Cholera | *2* |  |  |  | *2* |  |  |  |
| Conjunctivitis bacterial | 1 | 0.0 | 0.0 | 0.1 | 1 | 0.0 | 0.0 | 0.2 |
| Croup infectious | 1 | 0.0 | 0.0 | 0.1 | 1 | 0.0 | 0.0 | 0.2 |
| Dermatitis infected | *1* |  |  |  | *1* |  |  |  |
| Disseminated tuberculosis | 1 | 0.0 | 0.0 | 0.1 | 1 | 0.0 | 0.0 | 0.2 |
| Dysentery | 20 | 0.3 | 0.2 | 0.5 | 8 | 0.3 | 0.1 | 0.5 |
| Eczema infected | *1* |  |  |  | *1* |  |  |  |
| Encephalitic infection | *1* |  |  |  | *1* |  |  |  |
| Encephalitis viral | *1* |  |  |  | *1* |  |  |  |
| Enterococcal sepsis | *1* |  |  |  | *1* |  |  |  |
| Escherichia urinary tract infection | *2* |  |  |  | *2* |  |  |  |
| Furuncle | *1* |  |  |  | *1* |  |  |  |
| Gastroenteritis | 268 | 4.5 | 4.0 | 5.1 | 165 | 5.5 | 4.8 | 6.4 |
| Gastroenteritis salmonella | *5* |  |  |  | *5* |  |  |  |
| Gastroenteritis shigella | 1 | 0.0 | 0.0 | 0.1 | 1 | 0.0 | 0.0 | 0.2 |
| Gastroenteritis viral | *1* |  |  |  | *1* |  |  |  |
| Gastrointestinal candidiasis | *2* |  |  |  | *2* |  |  |  |
| Giardiasis | *1* |  |  |  | *1* |  |  |  |
| Groin abscess | *1* |  |  |  | *1* |  |  |  |
| Helminthic infection | 10 | 0.2 | 0.1 | 0.3 | 6 | 0.2 | 0.1 | 0.4 |
| HIV infection | 35 | 0.6 | 0.4 | 0.8 | 20 | 0.7 | 0.4 | 1.0 |
| HIV infection who clinical stage III | *1* |  |  |  | *1* |  |  |  |
| Impetigo | 3 | 0.1 | 0.0 | 0.1 | 3 | 0.1 | 0.0 | 0.3 |
| Infected skin ulcer | *1* |  |  |  | *1* |  |  |  |
| Klebsiella sepsis | *1* |  |  |  | *1* |  |  |  |
| Laryngitis | 1 | 0.0 | 0.0 | 0.1 | 1 | 0.0 | 0.0 | 0.2 |
| Lobar pneumonia | 5 | 0.1 | 0.0 | 0.2 | 5 | 0.2 | 0.1 | 0.4 |
| Lower respiratory tract infection | 5 | 0.1 | 0.0 | 0.2 | 6 | 0.2 | 0.1 | 0.4 |
| Ludwig angina | 2 | 0.0 | 0.0 | 0.1 | 1 | 0.0 | 0.0 | 0.2 |
| Lymph node abscess | *2* |  |  |  | *2* |  |  |  |
| Lymph node tuberculosis | 1 | 0.0 | 0.0 | 0.1 | 1 | 0.0 | 0.0 | 0.2 |
| Lymphadenitis bacterial | *1* |  |  |  | *1* |  |  |  |
| Malaria | 400 | 6.7 | 6.1 | 7.4 | 313 | 10.5 | 9.4 | 11.7 |
| Mastoiditis | *2* |  |  |  | *2* |  |  |  |
| Measles | 5 | 0.1 | 0.0 | 0.2 | 4 | 0.1 | 0.0 | 0.3 |
| Meningitis | 9 | 0.2 | 0.1 | 0.3 | 1 | 0.0 | 0.0 | 0.2 |
| Meningitis haemophilus | 1^‡^ |  |  |  | 0^‡^ |  |  |  |
| Meningitis meningococcal | 4^‡^ |  |  |  | 0^‡^ |  |  |  |
| Meningitis pneumococcal | 1^‡^ |  |  |  | 0^‡^ |  |  |  |
| Meningitis viral | 1^‡^ |  |  |  | 0^‡^ |  |  |  |
| Nasopharyngitis | *1* |  |  |  | *1* |  |  |  |
| Oral candidiasis | 11 | 0.2 | 0.1 | 0.3 | 4 | 0.1 | 0.0 | 0.3 |
| Oropharyngeal candidiasis | *2* |  |  |  | *2* |  |  |  |
| Osteomyelitis | 3 | 0.1 | 0.0 | 0.1 | 1 | 0.0 | 0.0 | 0.2 |
| Otitis media | 24 | 0.4 | 0.3 | 0.6 | 19 | 0.6 | 0.4 | 1.0 |
| Otitis media acute | 3 | 0.1 | 0.0 | 0.1 | 1 | 0.0 | 0.0 | 0.2 |
| Parotitis | *2* |  |  |  | *2* |  |  |  |
| Perineal abscess | *1* |  |  |  | *1* |  |  |  |
| Periorbital cellulitis | *1* |  |  |  | *1* |  |  |  |
| Pneumococcal sepsis | 6 | 0.1 | 0.0 | 0.2 | 3 | 0.1 | 0.0 | 0.3 |
| Pneumocystis jiroveci pneumonia | 1 | 0.0 | 0.0 | 0.1 | 1 | 0.0 | 0.0 | 0.2 |
| Pneumonia | 353 | 5.9 | 5.3 | 6.6 | 189 | 6.4 | 5.5 | 7.3 |
| Postoperative wound infection | *1* |  |  |  | *1* |  |  |  |
| Pseudomonal sepsis | *1* |  |  |  | *1* |  |  |  |
| Pulmonary tuberculosis | 4 | 0.1 | 0.0 | 0.2 | 4 | 0.1 | 0.0 | 0.3 |
| Pyelonephritis | *1* |  |  |  | *1* |  |  |  |
| Pyoderma | 2 | 0.0 | 0.0 | 0.1 | 3 | 0.1 | 0.0 | 0.3 |
| Pyomyositis | *3* |  |  |  | *3* |  |  |  |
| Respiratory tract infection | 4 | 0.1 | 0.0 | 0.2 | 2 | 0.1 | 0.0 | 0.2 |
| Salmonella sepsis | 43 | 0.7 | 0.5 | 1.0 | 27 | 0.9 | 0.6 | 1.3 |
| Salmonellosis | 4 | 0.1 | 0.0 | 0.2 | 2 | 0.1 | 0.0 | 0.2 |
| Schistosomiasis | *1* |  |  |  | *1* |  |  |  |
| Sepsis | 50 | 0.8 | 0.6 | 1.1 | 37 | 1.2 | 0.9 | 1.7 |
| Shigella infection | *1* |  |  |  | *1* |  |  |  |
| Skin bacterial infection | 2 | 0.0 | 0.0 | 0.1 | 2 | 0.1 | 0.0 | 0.2 |
| Skin infection | *3* |  |  |  | *3* |  |  |  |
| Staphylococcal sepsis | *6* |  |  |  | *6* |  |  |  |
| Staphylococcal skin infection | 2 | 0.0 | 0.0 | 0.1 | 2 | 0.1 | 0.0 | 0.2 |
| Streptococcal infection | *1* |  |  |  | *1* |  |  |  |
| Streptococcal sepsis | 1 | 0.0 | 0.0 | 0.1 | 1 | 0.0 | 0.0 | 0.2 |
| Subcutaneous abscess | 3 | 0.1 | 0.0 | 0.1 | 1 | 0.0 | 0.0 | 0.2 |
| Tinea capitis | *1* |  |  |  | *1* |  |  |  |
| Tonsillitis | 1 | 0.0 | 0.0 | 0.1 | 2 | 0.1 | 0.0 | 0.2 |
| Tracheobronchitis | *1* |  |  |  | *1* |  |  |  |
| Tuberculosis | 9 | 0.2 | 0.1 | 0.3 | 5 | 0.2 | 0.1 | 0.4 |
| Typhoid fever | 2 | 0.0 | 0.0 | 0.1 | 3 | 0.1 | 0.0 | 0.3 |
| Upper respiratory tract infection | 59 | 1.0 | 0.8 | 1.3 | 39 | 1.3 | 0.9 | 1.8 |
| Urinary tract infection | 39 | 0.7 | 0.5 | 0.9 | 21 | 0.7 | 0.4 | 1.1 |
| Varicella | *1* |  |  |  | *1* |  |  |  |
| Wound infection | *2* |  |  |  | *2* |  |  |  |
| Arthropod sting | *1* |  |  |  | *1* |  |  |  |
| Bronchitis chemical | 4 | 0.1 | 0.0 | 0.2 | 1 | 0.0 | 0.0 | 0.2 |
| Burns first degree | 4 | 0.1 | 0.0 | 0.2 | 1 | 0.0 | 0.0 | 0.2 |
| Burns second degree | 2 | 0.0 | 0.0 | 0.1 | 1 | 0.0 | 0.0 | 0.2 |
| Chemical injury | *1* |  |  |  | *1* |  |  |  |
| Chemical poisoning | 2 | 0.0 | 0.0 | 0.1 | 7 | 0.2 | 0.1 | 0.5 |
| Disinfectant poisoning | *1* |  |  |  | *1* |  |  |  |
| Eye injury | *1* |  |  |  | *1* |  |  |  |
| Femur fracture | 1 | 0.0 | 0.0 | 0.1 | 1 | 0.0 | 0.0 | 0.2 |
| Foreign body | *3* |  |  |  | *3* |  |  |  |
| Foreign body aspiration | *1* |  |  |  | *1* |  |  |  |
| Head injury | *1* |  |  |  | *1* |  |  |  |
| Herbal toxicity | 4 | 0.1 | 0.0 | 0.2 | 2 | 0.1 | 0.0 | 0.2 |
| Humerus fracture | *1* |  |  |  | *1* |  |  |  |
| Joint injury | *1* |  |  |  | *1* |  |  |  |
| Laceration | 1 | 0.0 | 0.0 | 0.1 | 1 | 0.0 | 0.0 | 0.2 |
| Limb traumatic amputation | *1* |  |  |  | *1* |  |  |  |
| Petroleum distillate poisoning | 3 | 0.1 | 0.0 | 0.1 | 4 | 0.1 | 0.0 | 0.3 |
| Pneumonitis chemical | 4 | 0.1 | 0.0 | 0.2 | 3 | 0.1 | 0.0 | 0.3 |
| Poisoning | *1* |  |  |  | *1* |  |  |  |
| Skin injury | *1* |  |  |  | *1* |  |  |  |
| Snake bite | *1* |  |  |  | *1* |  |  |  |
| Thermal burn | 17 | 0.3 | 0.2 | 0.5 | 7 | 0.2 | 0.1 | 0.5 |
| Dehydration | 2 | 0.0 | 0.0 | 0.1 | 1 | 0.0 | 0.0 | 0.2 |
| Failure to thrive | 1 | 0.0 | 0.0 | 0.1 | 2 | 0.1 | 0.0 | 0.2 |
| Hypoglycemia | 13 | 0.2 | 0.1 | 0.4 | 17 | 0.6 | 0.3 | 0.9 |
| Hypokalaemia | *1* |  |  |  | *1* |  |  |  |
| Hypoproteinaemia | 1 | 0.0 | 0.0 | 0.1 | 1 | 0.0 | 0.0 | 0.2 |
| Kwashiorkor | 13 | 0.2 | 0.1 | 0.4 | 16 | 0.5 | 0.3 | 0.9 |
| Malnutrition | 48 | 0.8 | 0.6 | 1.1 | 20 | 0.7 | 0.4 | 1.0 |
| Marasmus | 14 | 0.2 | 0.1 | 0.4 | 4 | 0.1 | 0.0 | 0.3 |
| Underweight | *1* |  |  |  | *1* |  |  |  |
| Arthritis | *2* |  |  |  | *2* |  |  |  |
| Myositis | *1* |  |  |  | *1* |  |  |  |
| Brain neoplasm | *1* |  |  |  | *1* |  |  |  |
| Arachnoid cyst | *1* |  |  |  | *1* |  |  |  |
| Cerebral atrophy | 1 | 0.0 | 0.0 | 0.1 | 1 | 0.0 | 0.0 | 0.2 |
| Convulsion | 62 | 1.0 | 0.8 | 1.3 | 41 | 1.4 | 1.0 | 1.9 |
| Encephalitis | 2 | 0.0 | 0.0 | 0.1 | 1 | 0.0 | 0.0 | 0.2 |
| Epilepsy | 5 | 0.1 | 0.0 | 0.2 | 1 | 0.0 | 0.0 | 0.2 |
| Febrile convulsion | 224 | 3.8 | 3.3 | 4.3 | 112 | 3.8 | 3.1 | 4.5 |
| Hemiparesis | 1 | 0.0 | 0.0 | 0.1 | 1 | 0.0 | 0.0 | 0.2 |
| Hemiplegia | *1* |  |  |  | *1* |  |  |  |
| Hydrocephalus | *1* |  |  |  | *1* |  |  |  |
| Meningism | 1 | 0.0 | 0.0 | 0.1 | 2 | 0.1 | 0.0 | 0.2 |
| Nephrotic syndrome | *1* |  |  |  | *1* |  |  |  |
| Acquired phimosis | *1* |  |  |  | *1* |  |  |  |
| Asphyxia | 1 | 0.0 | 0.0 | 0.1 | 1 | 0.0 | 0.0 | 0.2 |
| Aspiration | *1* |  |  |  | *1* |  |  |  |
| Asthma | 11 | 0.2 | 0.1 | 0.3 | 6 | 0.2 | 0.1 | 0.4 |
| Bronchospasm | 1 | 0.0 | 0.0 | 0.1 | 3 | 0.1 | 0.0 | 0.3 |
| Cough | *1* |  |  |  | *1* |  |  |  |
| Epistaxis | *1* |  |  |  | *1* |  |  |  |
| Pneumonia aspiration | 7 | 0.1 | 0.0 | 0.2 | 5 | 0.2 | 0.1 | 0.4 |
| Pulmonary oedema | *1* |  |  |  | *1* |  |  |  |
| Respiratory acidosis | *1* |  |  |  | *1* |  |  |  |
| Respiratory disorder | *1* |  |  |  | *1* |  |  |  |
| Dermatitis | 2 | 0.0 | 0.0 | 0.1 | 2 | 0.1 | 0.0 | 0.2 |
| Dermatitis allergic | *1* |  |  |  | *1* |  |  |  |
| Rash | *1* |  |  |  | *1* |  |  |  |
| Rash papular | *1* |  |  |  | *1* |  |  |  |
| Skin lesion | *1* |  |  |  | *1* |  |  |  |
| Urticaria | *1* |  |  |  | *1* |  |  |  |
| Child abuse | *1* |  |  |  | *1* |  |  |  |
| Haematoma | *1* |  |  |  | *1* |  |  |  |
| Hypovolaemic shock | *1* |  |  |  | *1* |  |  |  |
| Shock | 2 | 0.0 | 0.0 | 0.1 | 4 | 0.1 | 0.0 | 0.3 |

At least one symptom = at least one symptom experienced, regardless of the MedDRA Preferred Term.

At least one symptom excluding malaria = at least one symptom experienced (regardless of the MedDRA Preferred Term), excluding malaria, *P. falciparum* infection, and cerebral malaria.

N = number of children with at least one administered dose.

n/% = number/percentage of children reporting the symptom at least once.

95% CI = exact 95% confidence interval; LL = Lower Limit, UL = Upper Limit.

Tabulations that present single or multiple SAEs in one study group (RTS,S/AS01 or control) are presented in both study groups as *n*, indicating that there are n events in one of the study groups, to preserve the blind of the study.

^‡^ These cases of meningitis were unblinded by the Sponsor Safety Physician as this information was important for safety evaluation.

## Supplementary table 14b. Percentage of children in the 6-12 weeks age category reporting a serious adverse event during 20 months post dose-1 by MedDRA Preferred Term (intention-to-treat population)

|  | **RTS,S/AS01 vaccine**  **N=4358** | | | | **Control vaccine**  **N=2179** | | | |
| --- | --- | --- | --- | --- | --- | --- | --- | --- |
|  |  | | **95% CI** | |  | | **95% CI** | |
|  | **n** | **%** | **LL** | **UL** | **n** | **%** | **LL** | **UL** |
| At least one SAE | 959 | 22.0 | 20.8 | 23.3 | 503 | 23.1 | 21.3 | 24.9 |
| At least one SAE excluding Malaria | 933 | 21.4 | 20.2 | 22.7 | 482 | 22.1 | 20.4 | 23.9 |
| At least one fatal SAE ^§^ | 86 | 2.0 | 1.6 | 2.4 | 34 | 1.6 | 1.1 | 2.2 |
| At least one related SAE | 4 | 0.1 | 0.0 | 0.2 | 3 | 0.1 | 0.0 | 0.4 |
| **SAE by MedDRA Preferred Term** |  |  |  |  |  |  |  |  |
| Anemia | 124 | 2.8 | 2.4 | 3.4 | 73 | 3.4 | 2.6 | 4.2 |
| Hemolytic anemia | 1 | 0.0 | 0.0 | 0.1 | 1 | 0.0 | 0.0 | 0.3 |
| Lymphadenitis | *2* |  |  |  | *2* |  |  |  |
| Pericardial effusion | *1* |  |  |  | *1* |  |  |  |
| Cerebral Palsy | *1* |  |  |  | *1* |  |  |  |
| Congenital megacolon | *1* |  |  |  | *1* |  |  |  |
| Fallot’s tetralogy | *1* |  |  |  | *1* |  |  |  |
| Glucose-6-phosphate dehydrogenase deficiency | *1* |  |  |  | *1* |  |  |  |
| Sickle cell anemia | 4 | 0.1 | 0.0 | 0.2 | 4 | 0.2 | 0.1 | 0.5 |
| Sickle cell anemia with crisis | 4 | 0.1 | 0.0 | 0.2 | 3 | 0.1 | 0.0 | 0.4 |
| Trisomy 21 | *1* |  |  |  | *1* |  |  |  |
| Urethral valves | *1* |  |  |  | *1* |  |  |  |
| Conjunctivitis | *1* |  |  |  | *1* |  |  |  |
| Periorbital oedema | *1* |  |  |  | *1* |  |  |  |
| Constipation | *1* |  |  |  | *1* |  |  |  |
| Enteritis | 15 | 0.3 | 0.2 | 0.6 | 16 | 0.7 | 0.4 | 1.2 |
| Gastritis | 3 | 0.1 | 0.0 | 0.2 | 3 | 0.1 | 0.0 | 0.4 |
| Inguinal hernia | 1 | 0.0 | 0.0 | 0.1 | 3 | 0.1 | 0.0 | 0.4 |
| Intestinal obstruction | *2* |  |  |  | *2* |  |  |  |
| Intussusception | *1* |  |  |  | *1* |  |  |  |
| Rectal prolapse | *1* |  |  |  | *1* |  |  |  |
| Stomatitis | *1* |  |  |  | *1* |  |  |  |
| Death | 1 | 0.0 | 0.0 | 0.1 | 2 | 0.1 | 0.0 | 0.3 |
| Drowning | *1* |  |  |  | *1* |  |  |  |
| Hypothermia | 2 | 0.0 | 0.0 | 0.2 | 1 | 0.0 | 0.0 | 0.3 |
| Injection site reaction | *1* |  |  |  | *1* |  |  |  |
| Pyrexia | 17 | 0.4 | 0.2 | 0.6 | 13 | 0.6 | 0.3 | 1.0 |
| Hepatitis | *1* |  |  |  | *1* |  |  |  |
| Anaphylactic reaction | *1* |  |  |  | *1* |  |  |  |
| Drug hypersensitivity | *1* |  |  |  | *1* |  |  |  |
| Immune reconstitution inflammatory syndrome | *1* |  |  |  | *1* |  |  |  |
| Abscess | 10 | 0.2 | 0.1 | 0.4 | 5 | 0.2 | 0.1 | 0.5 |
| Abscess limb | 1 | 0.0 | 0.0 | 0.1 | 1 | 0.0 | 0.0 | 0.3 |
| Abscess neck | *1* |  |  |  | *1* |  |  |  |
| Amoebiasis | *1* |  |  |  | *1* |  |  |  |
| Arthritis bacterial | *5* |  |  |  | *5* |  |  |  |
| Atypical pneumonia | *1* |  |  |  | *1* |  |  |  |
| Bacterial infection | *1* |  |  |  | *1* |  |  |  |
| Bronchiolitis | 29 | 0.7 | 0.4 | 1.0 | 23 | 1.1 | 0.7 | 1.6 |
| Bronchitis | 13 | 0.3 | 0.2 | 0.5 | 3 | 0.1 | 0.0 | 0.4 |
| Bronchopneumonia | 46 | 1.1 | 0.8 | 1.4 | 26 | 1.2 | 0.8 | 1.7 |
| Bullous impetigo | *1* |  |  |  | *1* |  |  |  |
| Burn infection | *1* |  |  |  | *1* |  |  |  |
| Candidiasis | *1* |  |  |  | *1* |  |  |  |
| Cellulitis | 8 | 0.2 | 0.1 | 0.4 | 3 | 0.1 | 0.0 | 0.4 |
| Central nervous system viral infection | *1* |  |  |  | *1* |  |  |  |
| Cerebral malaria | 2 | 0.0 | 0.0 | 0.2 | 1 | 0.0 | 0.0 | 0.3 |
| Conjunctivitis bacterial | *2* |  |  |  | *2* |  |  |  |
| Dysentery | 6 | 0.1 | 0.1 | 0.3 | 6 | 0.3 | 0.1 | 0.6 |
| Encephalitis viral | *1* |  |  |  | *1* |  |  |  |
| Escherichia sepsis | 2 | 0.0 | 0.0 | 0.2 | 2 | 0.1 | 0.0 | 0.3 |
| Escherichia urinary tract infection | *2* |  |  |  | *2* |  |  |  |
| Exanthema subitum | *1* |  |  |  | *1* |  |  |  |
| Febrile infection | *1* |  |  |  | *1* |  |  |  |
| Gastroenteritis | 306 | 7.0 | 6.3 | 7.8 | 163 | 7.5 | 6.4 | 8.7 |
| Gastroenteritis salmonella | 2 | 0.0 | 0.0 | 0.2 | 2 | 0.1 | 0.0 | 0.3 |
| Gastroenteritis shigella | *1* |  |  |  | *1* |  |  |  |
| Giardiasis | *1* |  |  |  | *1* |  |  |  |
| Helminthic infection | 1 | 0.0 | 0.0 | 0.1 | 1 | 0.0 | 0.0 | 0.3 |
| HIV infection | 33 | 0.8 | 0.5 | 1.1 | 11 | 0.5 | 0.3 | 0.9 |
| HIV infection who clinical stage iii | *2* |  |  |  | *2* |  |  |  |
| HIV infection who clinical stage iv | *1* |  |  |  | *1* |  |  |  |
| Impetigo | *4* |  |  |  | *4* |  |  |  |
| Injection site abscess | *1* |  |  |  | *1* |  |  |  |
| Listeria sepsis | *1* |  |  |  | *1* |  |  |  |
| Lobar pneumonia | 13 | 0.3 | 0.2 | 0.5 | 6 | 0.3 | 0.1 | 0.6 |
| Lower respiratory tract infection | 4 | 0.1 | 0.0 | 0.2 | 2 | 0.1 | 0.0 | 0.3 |
| Ludwig angina | *1* |  |  |  | *1* |  |  |  |
| Lymph node abscess | *1* |  |  |  | *1* |  |  |  |
| Malaria | 264 | 6.1 | 5.4 | 6.8 | 148 | 6.8 | 5.8 | 7.9 |
| Mastoiditis (10026900) | *1* |  |  |  | *1* |  |  |  |
| Measles | 23 | 0.5 | 0.3 | 0.8 | 8 | 0.4 | 0.2 | 0.7 |
| Meningitis | 3 | 0.1 | 0.0 | 0.2 | 2 | 0.1 | 0.0 | 0.3 |
| Meningitis pneumococcal | 3 | 0.1 | 0.0 | 0.2 | 1 | 0.0 | 0.0 | 0.3 |
| Meningitis salmonella | 3^‡^ |  |  |  | 0^‡^ |  |  |  |
| Moraxella infection | *1* |  |  |  | *1* |  |  |  |
| Oral candidiasis | 3 | 0.1 | 0.0 | 0.2 | 1 | 0.0 | 0.0 | 0.3 |
| Oropharyngeal candidiasis | *1* |  |  |  | *1* |  |  |  |
| Osteomyelitis | 2 | 0.0 | 0.0 | 0.2 | 2 | 0.1 | 0.0 | 0.3 |
| Otitis externa | *2* |  |  |  | *2* |  |  |  |
| Otitis media | 18 | 0.4 | 0.2 | 0.7 | 5 | 0.2 | 0.1 | 0.5 |
| Otitis media acute | 3 | 0.1 | 0.0 | 0.2 | 1 | 0.0 | 0.0 | 0.3 |
| Parotitis | *1* |  |  |  | *1* |  |  |  |
| Periorbital cellulitis | *1* |  |  |  | *1* |  |  |  |
| Peritonitis | *1* |  |  |  | *1* |  |  |  |
| Pneumococcal sepsis | 7 | 0.2 | 0.1 | 0.3 | 3 | 0.1 | 0.0 | 0.4 |
| Pneumocystis jiroveci pneumonia | *5* |  |  |  | *5* |  |  |  |
| Pneumonia | 369 | 8.5 | 7.7 | 9.3 | 178 | 8.2 | 7.1 | 9.4 |
| Pneumonia pneumococcal | *1* |  |  |  | *1* |  |  |  |
| Pneumonia viral | *1* |  |  |  | *1* |  |  |  |
| Pulmonary tuberculosis | 12 | 0.3 | 0.1 | 0.5 | 1 | 0.0 | 0.0 | 0.3 |
| Respiratory tract infection | *1* |  |  |  | *1* |  |  |  |
| Rubella | *1* |  |  |  | *1* |  |  |  |
| Salmonella sepsis | 40 | 0.9 | 0.7 | 1.2 | 23 | 1.1 | 0.7 | 1.6 |
| Sepsis | 33 | 0.8 | 0.5 | 1.1 | 12 | 0.6 | 0.3 | 1.0 |
| Septic shock | *1* |  |  |  | *1* |  |  |  |
| Staphylococcal sepsis | 8 | 0.2 | 0.1 | 0.4 | 1 | 0.0 | 0.0 | 0.3 |
| Staphylococcal skin infection | 1 | 0.0 | 0.0 | 0.1 | 1 | 0.0 | 0.0 | 0.3 |
| Streptococcal sepsis | 1 | 0.0 | 0.0 | 0.1 | 1 | 0.0 | 0.0 | 0.3 |
| Subcutaneous abscess | 5 | 0.1 | 0.0 | 0.3 | 3 | 0.1 | 0.0 | 0.4 |
| Tonsillitis | *2* |  |  |  | *2* |  |  |  |
| Tuberculosis | 5 | 0.1 | 0.0 | 0.3 | 4 | 0.2 | 0.1 | 0.5 |
| Upper respiratory tract infection | 44 | 1.0 | 0.7 | 1.4 | 22 | 1.0 | 0.6 | 1.5 |
| Urinary tract infection | 21 | 0.5 | 0.3 | 0.7 | 14 | 0.6 | 0.4 | 1.1 |
| Vaginal infection | *1* |  |  |  | *1* |  |  |  |
| Varicella | 1 | 0.0 | 0.0 | 0.1 | 1 | 0.0 | 0.0 | 0.3 |
| Viral infection | *1* |  |  |  | *1* |  |  |  |
| Burns first degree | 2 | 0.0 | 0.0 | 0.2 | 1 | 0.0 | 0.0 | 0.3 |
| Burns second degree | 5 | 0.1 | 0.0 | 0.3 | 3 | 0.1 | 0.0 | 0.4 |
| Clavicle fracture | *1* |  |  |  | *1* |  |  |  |
| Femur fracture | *2* |  |  |  | *2* |  |  |  |
| Greenstick fracture | *1* |  |  |  | *1* |  |  |  |
| Head injury | *3* |  |  |  | *3* |  |  |  |
| Herbal toxicity | 1 | 0.0 | 0.0 | 0.1 | 3 | 0.1 | 0.0 | 0.4 |
| Human bite | *1* |  |  |  | *1* |  |  |  |
| Petroleum distillate poisoning | *1* |  |  |  | *1* |  |  |  |
| Pneumonitis chemical | *3* |  |  |  | *3* |  |  |  |
| Soft tissue injury | *2* |  |  |  | *2* |  |  |  |
| Thermal burn | 13 | 0.3 | 0.2 | 0.5 | 8 | 0.4 | 0.2 | 0.7 |
| Wrist fracture | *1* |  |  |  | *1* |  |  |  |
| Failure to thrive | 1 | 0.0 | 0.0 | 0.1 | 1 | 0.0 | 0.0 | 0.3 |
| Hypoglycemia | 3 | 0.1 | 0.0 | 0.2 | 3 | 0.1 | 0.0 | 0.4 |
| Hypokalaemia | *1* |  |  |  | *1* |  |  |  |
| Kwashiorkor | 7 | 0.2 | 0.1 | 0.3 | 3 | 0.1 | 0.0 | 0.4 |
| Malnutrition | 42 | 1.0 | 0.7 | 1.3 | 11 | 0.5 | 0.3 | 0.9 |
| Marasmus | 10 | 0.2 | 0.1 | 0.4 | 7 | 0.3 | 0.1 | 0.7 |
| Arthritis | *1* |  |  |  | *1* |  |  |  |
| Dactylitis | 1 | 0.0 | 0.0 | 0.1 | 1 | 0.0 | 0.0 | 0.3 |
| Myositis | *1* |  |  |  | *1* |  |  |  |
| Rickets | *1* |  |  |  | *1* |  |  |  |
| Torticollis | *1* |  |  |  | *1* |  |  |  |
| Acute promyelocytic leukaemia | *1* |  |  |  | *1* |  |  |  |
| Inflammatory pseudotumour | *1* |  |  |  | *1* |  |  |  |
| Langerhans’ cell histiocytosis | *1* |  |  |  | *1* |  |  |  |
| Cerebellar ataxia | *1* |  |  |  | *1* |  |  |  |
| Convulsion | 54 | 1.2 | 0.9 | 1.6 | 21 | 1.0 | 0.6 | 1.5 |
| Encephalitis | 1 | 0.0 | 0.0 | 0.1 | 1 | 0.0 | 0.0 | 0.3 |
| Encephalomalacia | *1* |  |  |  | *1* |  |  |  |
| Encephalopathy | *1* |  |  |  | *1* |  |  |  |
| Epilepsy | *1* |  |  |  | *1* |  |  |  |
| Febrile convulsion | 117 | 2.7 | 2.2 | 3.2 | 65 | 3.0 | 2.3 | 3.8 |
| Loss of consciousness | *1* |  |  |  | *1* |  |  |  |
| Meningism | *1* |  |  |  | *1* |  |  |  |
| Metabolic encephalopathy | *1* |  |  |  | *1* |  |  |  |
| Myoclonus | *1* |  |  |  | *1* |  |  |  |
| Glomerulonephritis | *1* |  |  |  | *1* |  |  |  |
| Glomerulonephritis acute | *1* |  |  |  | *1* |  |  |  |
| Hydronephrosis | *1* |  |  |  | *1* |  |  |  |
| Renal tubular necrosis | *1* |  |  |  | *1* |  |  |  |
| Urinary retention | *1* |  |  |  | *1* |  |  |  |
| Acquired phimosis | *1* |  |  |  | *1* |  |  |  |
| Asthma | 7 | 0.2 | 0.1 | 0.3 | 5 | 0.2 | 0.1 | 0.5 |
| Bronchial hyperreactivity | 1 | 0.0 | 0.0 | 0.1 | 1 | 0.0 | 0.0 | 0.3 |
| Bronchospasm | 4 | 0.1 | 0.0 | 0.2 | 2 | 0.1 | 0.0 | 0.3 |
| Obstructive airways disorder | *1* |  |  |  | *1* |  |  |  |
| Pneumonia aspiration | 3 | 0.1 | 0.0 | 0.2 | 4 | 0.2 | 0.1 | 0.5 |
| Pneumonitis | *1* |  |  |  | *1* |  |  |  |
| Respiratory arrest | *1* |  |  |  | *1* |  |  |  |
| Dermatitis exfoliative | *1* |  |  |  | *1* |  |  |  |
| Drug eruption | *1* |  |  |  | *1* |  |  |  |
| Urticaria | *1* |  |  |  | *1* |  |  |  |
| Hypovolaemic shock | *1* |  |  |  | *1* |  |  |  |
| Shock | 2 | 0.0 | 0.0 | 0.2 | 4 | 0.2 | 0.1 | 0.5 |

At least one symptom = at least one symptom experienced, regardless of the MedDRA Preferred Term.

At least one symptom excluding malaria = at least one symptom experienced (regardless of the MedDRA Preferred Term), excluding malaria, *P. falciparum* infection, and cerebral malaria.

N = number of infants with at least one administered dose.

n/% = number/percentage of infants reporting the symptom at least once.

95% CI = exact 95% confidence interval; LL = Lower Limit, UL = Upper Limit.

Tabulations that present single or multiple SAEs in one study group (RTS,S/AS01 or control) are presented in both study groups as *n*, indicating that there are n events in one of the study groups, to preserve the blind of the study.

^§^ It should be noted that the number of fatal SAEs in the safety analysis represents the SAEs that started within the time period on which the paper reports, while the all-cause mortality analysis (presented in Supplementary table 11) reports on the fatalities that occurred within that same time period. Because 3 infants had an SAE with fatal outcome that started in the time period for which we report, but died after this time period, the SAE tables on the infants reports 120 subjects with a fatal SAE, but the all-cause mortality analysis only reports 117 cases.

^‡^ These cases of meningitis were unblinded by the Sponsor Safety Physician as this information was important for safety evaluation.
