# Supplementary material for: Efficacy and Safety of the RTS,S/AS01 Malaria Vaccine during 18 Months after Vaccination: A Phase 3 Randomized, Controlled Trial in Children and Young Infants at 11 African Sites
Source: PLoS Med. 2014 Jul 29;11(7):e1001685. doi: 10.1371/journal.pmed.1001685 (PMC4114488; doi:10.1371/journal.pmed.1001685)
Supplement: Table S15 — Vaccine efficacy against clinical and severe malaria in all children enrolled in the 5–17-mo age category during a 12-mo follow-up period after dose 3. (DOCX) [file pmed.1001685.s024.docx]

## Supplementary table 15. Vaccine efficacy against clinical and severe malaria in all children enrolled in the 5-17 months age category during a 12-month follow-up period post dose-3

|  | **RTS,S/AS01 vaccine** | | | | | **Control vaccine** | | | | | **Protective Efficacy** | |
| --- | --- | --- | --- | --- | --- | --- | --- | --- | --- | --- | --- | --- |
| **Clinical Malaria** | **Number of children (N)** | **Number of episodes (n)** | **Person years at risk (T)** | | **n/T** | **Number of children (N)** | **Number of episodes (n)** | **Person years at risk (T)** | | **n/T** | **% (95% CI)** | **p-value*** |
| **12 months after third dose of vaccine  (per-protocol population)** |  |  |  | |  |  |  |  | |  |  |  |
| **First or only episode** |  |  |  | |  |  |  |  | |  |  |  |
| >5000 parasites/mm^3^ and temperature ≥37.5°C | 4553 | 1348 | 3537.6 | | 0.38 | 2327 | 1035 | 1538.5 | | 0.67 | 54.6 (50.7-58.1) | <0.001 |
| > 0 parasites/mm^3^ and measured or reported fever | 4553 | 1705 | 3307.6 | | 0.52 | 2327 | 1200 | 1406.5 | | 0.85 | 53.3 (49.7-56.7) | <0.001 |
| **All episodes** |  |  |  | |  |  |  |  | |  |  |  |
| >5000 parasites/mm^3^ and temperature ≥37.5°C | 4553 | 2558 | 4035.9 | | 0.63 | 2327 | 2489 | 2024.6 | | 1.23 | 51.3 (47.5-54.9) | <0.001 |
| > 0 parasites/mm^3^ and measured or reported fever | 4553 | 4002 | 3980.1 | | 1.01 | 2327 | 3704 | 1977.8 | | 1.87 | 51.9 (48.4-55.2) | <0.001 |
| **14 months after first dose of vaccine (intention-to-treat population)** |  |  |  | |  |  |  |  | |  |  |  |
| **First or only episode** |  |  |  | |  |  |  |  | |  |  |  |
| >5000 parasites/mm^3^ and temperature ≥37.5°C | 5949 | 1653 | 5481.4 | | 0.30 | 2974 | 1204 | 2439.3 | | 0.49 | 49.1 (45.2-52.8) | <0.001 |
| **All episodes** |  |  |  | |  |  |  |  | |  |  |  |
| >5000 parasites/mm^3^ and temperature ≥37.5°C | 5949 | 3248 | 6330.9 | | 0.51 | 2974 | 3045 | 3138.4 | | 0.97 | 50.7 (47.0-54.1) | <0.001 |
| **Severe malaria** | **N** | **n** |  | **Proportion Affected (%)** | | **N** | **n** |  | **Proportion Affected (%)** | | **% (95% CI)** | **p-value**** |
| **12 months after third dose of vaccine  (per-protocol population)** |  |  |  |  | |  |  |  |  | |  |  |
| Primary case definition | 4553 | 77 |  | 1.7 | | 2327 | 71 |  | 3.1 | | 44.6 (22.4-60.4) | <0.001 |
| Secondary case definition | 4553 | 96 |  | 2.1 | | 2327 | 90 |  | 3.9 | | 45.5 (26.5-59.5) | <0.001 |
| **14 months after first dose of vaccine (intention-to-treat population)** |  |  |  |  | |  |  |  |  | |  |  |
| Primary case definition | 5949 | 106 |  | 1.8 | | 2974 | 92 |  | 3.1 | | 42.4 (23.0-56.8) | <0.001 |
| Secondary case definition | 5949 | 131 |  | 2.2 | | 2974 | 114 |  | 3.8 | | 42.6 (25.5-55.6) | <0.001 |

VE reported previously for children aged 5-17 months included only the first 6000 children enrolled^3^. Here we calculate VE during 12 months follow-up on all children (N= 8923) enrolled in the older age-category for all primary case definitions of clinical and severe malaria to provide efficacy figures for comparison to the reported 18 months follow-up.

Clinical malaria primary case definition: Illness in a child brought to a study facility with a temperature of ≥ 37.5°C and *P. falciparum* asexual parasitemia at a density of > 5000 parasites per cubic millimeter or a case of malaria meeting the primary case definition of severe malaria.

Clinical malaria secondary case definition: Illness in a child brought to a study facility with a measured temperature of ≥37.5°C or reported fever within the last 24 hours and *P. falciparum* asexual parasitemia at a density of > 0 parasites per cubic millimeter.

Severe malaria primary case definition: *P. falciparum* asexual parasitemia at a density of > 5000 parasites per cubic millimeter with one or more markers of disease severity and without diagnosis of a coexisting illness.

Severe malaria secondary case definition: *P. falciparum* asexual parasitemia at a density of > 5000 parasites per cubic millimeter with one or more markers of disease severity, including cases in which a coexisting illness was present or could not be ruled out. Markers of severe disease were prostration, respiratory distress, a Blantyre coma score of ≤ 2 (on a scale of 0 to 5, with higher scores indicating a higher level of consciousness), two or more observed or reported seizures, hypoglycemia, acidosis, elevated lactate level, or hemoglobin level of < 5 g per deciliter. Coexisting illnesses were defined as radiographically proven pneumonia, meningitis established by analysis of cerebrospinal fluid, bacteremia, or gastroenteritis with severe dehydration.

* For clinical malaria: p-value from negative binomial regression.

** For severe malaria, malaria hospitalization and all-cause hospitalization: p-value from two-sided Fisher exact test.
