# Supplementary material for: Efficacy and Safety of the RTS,S/AS01 Malaria Vaccine during 18 Months after Vaccination: A Phase 3 Randomized, Controlled Trial in Children and Young Infants at 11 African Sites
Source: PLoS Med. 2014 Jul 29;11(7):e1001685. doi: 10.1371/journal.pmed.1001685 (PMC4114488; doi:10.1371/journal.pmed.1001685)
Supplement: Table S16 — Seropositivity rates and geometric mean titers for anti-CS antibodies at baseline and 1 mo after dose 3 in the 5–17-mo and 6–12-wk age categories by site and overall. (DOCX) [file pmed.1001685.s025.docx]

## Supplementary table 16a. Seropositivity rates and GMTs for anti-CS antibodies at baseline and one month post dose-3 in the 5-17 months age category by site and overall

|  | | | **Per-protocol population** | | | | | | | | | | **Intention-to-treat population** | | | | | | | | | |
| --- | --- | --- | --- | --- | --- | --- | --- | --- | --- | --- | --- | --- | --- | --- | --- | --- | --- | --- | --- | --- | --- | --- |
|  | | |  | **Seropositivity** | | | | **GMT** | | |  | |  | **Seropositivity** | | | | **GMT** | | |  | |
|  |  |  |  |  | | **95% CI** | |  | **95% CI** | |  |  |  |  | | **95% CI** | |  | **95% CI** | |  |  |
| **Site** | **Group** | **Timing** | **N** | **n** | **%** | **LL** | **UL** | **value** | **LL** | **UL** | **Min** | **Max** | **N** | **n** | **%** | **LL** | **UL** | **value** | **LL** | **UL** | **Min** | **Max** |
| Kilifi | RTS,S/AS01 | Screening | 94 | 0 | 0.0 | 0.0 | 3.8 | 0.3 | 0.3 | 0.3 | <0.5 | <0.5 | 114 | 0 | 0.0 | 0.0 | 3.2 | 0.3 | 0.3 | 0.3 | <0.5 | <0.5 |
|  |  | One month post dose-3 | 109 | 109 | 100 | 96.7 | 100 | 685.2 | 606.1 | 774.6 | 170.3 | 2818.7 | 125 | 125 | 100 | 97.1 | 100 | 592.7 | 495.5 | 709.1 | 2.7 | 2818.7 |
|  | Control vaccine | Screening | 39 | 1 | 2.6 | 0.1 | 13.5 | 0.3 | 0.2 | 0.3 | <0.5 | 0.5 | 46 | 1 | 2.2 | 0.1 | 11.5 | 0.3 | 0.2 | 0.3 | <0.5 | 0.5 |
|  |  | One month post dose-3 | 52 | 1 | 1.9 | 0.0 | 10.3 | 0.3 | 0.2 | 0.3 | <0.5 | 0.5 | 54 | 1 | 1.9 | 0.0 | 9.9 | 0.3 | 0.2 | 0.3 | <0.5 | 0.5 |
| Korogwe | RTS,S/AS01 | Screening | 125 | 0 | 0.0 | 0.0 | 2.9 | 0.3 | 0.3 | 0.3 | <0.5 | <0.5 | 134 | 0 | 0.0 | 0.0 | 2.7 | 0.3 | 0.3 | 0.3 | <0.5 | <0.5 |
|  |  | One month post dose-3 | 122 | 122 | 100 | 97.0 | 100 | 534.7 | 477.6 | 598.5 | 118.6 | 2356.8 | 128 | 128 | 100 | 97.2 | 100 | 540.0 | 482.4 | 604.4 | 118.6 | 2608.8 |
|  | Control vaccine | Screening | 56 | 0 | 0.0 | 0.0 | 6.4 | 0.3 | 0.3 | 0.3 | <0.5 | <0.5 | 64 | 0 | 0.0 | 0.0 | 5.6 | 0.3 | 0.3 | 0.3 | <0.5 | <0.5 |
|  |  | One month post dose-3 | 56 | 2 | 3.6 | 0.4 | 12.3 | 0.3 | 0.2 | 0.4 | <0.5 | 329.9 | 64 | 2 | 3.1 | 0.4 | 10.8 | 0.3 | 0.2 | 0.4 | <0.5 | 329.9 |
| Manhiça | RTS,S/AS01 |  | - | - | - | - | - | - | - | - | - | - | 136 | 1 | 0.7 | 0.0 | 4.0 | 0.3 | 0.2 | 0.3 | <0.5 | 15.3 |
|  |  |  | - | - | - | - | - | - | - | - | - | - | 123 | 123 | 100 | 97.0 | 100 | 621.1 | 549.9 | 701.5 | 137.9 | 3724.8 |
|  | Control vaccine |  | - | - | - | - | - | - | - | - | - | - | 64 | 2 | 3.1 | 0.4 | 10.8 | 0.3 | 0.2 | 0.3 | <0.5 | 1.7 |
|  |  |  | - | - | - | - | - | - | - | - | - | - | 57 | 1 | 1.8 | 0.0 | 9.4 | 0.3 | 0.2 | 0.4 | <0.5 | 705.0 |
| Lambarene | RTS,S/AS01 | Screening | 86 | 5 | 5.8 | 1.9 | 13.0 | 0.3 | 0.3 | 0.3 | <0.5 | 1.7 | 132 | 8 | 6.1 | 2.7 | 11.6 | 0.3 | 0.3 | 0.3 | <0.5 | 1.7 |
|  |  | One month post dose-3 | 80 | 80 | 100 | 95.5 | 100 | 385.0 | 322.6 | 459.5 | 41.3 | 3141.1 | 114 | 114 | 100 | 96.8 | 100 | 374.2 | 321.9 | 435.1 | 18.0 | 3141.1 |
|  | Control vaccine | Screening | 50 | 3 | 6.0 | 1.3 | 16.5 | 0.3 | 0.2 | 0.3 | <0.5 | 2.5 | 65 | 3 | 4.6 | 1.0 | 12.9 | 0.3 | 0.2 | 0.3 | <0.5 | 2.5 |
|  |  | One month post dose-3 | 49 | 2 | 4.1 | 0.5 | 14.0 | 0.3 | 0.2 | 0.3 | <0.5 | 2.4 | 61 | 2 | 3.3 | 0.4 | 11.3 | 0.3 | 0.2 | 0.3 | <0.5 | 2.4 |
| Bagamoyo | RTS,S/AS01 | Screening | 84 | 6 | 7.1 | 2.7 | 14.9 | 0.3 | 0.3 | 0.3 | <0.5 | 2.0 | 133 | 12 | 9.0 | 4.7 | 15.2 | 0.3 | 0.3 | 0.3 | <0.5 | 3.0 |
|  |  | One month post dose-3 | 84 | 84 | 100 | 95.7 | 100 | 514.0 | 441.0 | 599.0 | 55.9 | 2495.5 | 121 | 120 | 99.2 | 95.5 | 100 | 449.6 | 372.1 | 543.3 | <0.5 | 3380.8 |
|  | Control vaccine | Screening | 45 | 2 | 4.4 | 0.5 | 15.1 | 0.3 | 0.2 | 0.3 | <0.5 | 2.1 | 67 | 3 | 4.5 | 0.9 | 12.5 | 0.3 | 0.2 | 0.3 | <0.5 | 2.1 |
|  |  | One month post dose-3 | 45 | 3 | 6.7 | 1.4 | 18.3 | 0.3 | 0.2 | 0.3 | <0.5 | 1.3 | 63 | 4 | 6.3 | 1.8 | 15.5 | 0.3 | 0.2 | 0.3 | <0.5 | 2.9 |
| Lilongwe | RTS,S/AS01 | Screening | 47 | 2 | 4.3 | 0.5 | 14.5 | 0.3 | 0.2 | 0.3 | <0.5 | 1.3 | 137 | 5 | 3.6 | 1.2 | 8.3 | 0.3 | 0.3 | 0.3 | <0.5 | 3.4 |
|  |  | One month post dose-3 | 45 | 45 | 100 | 92.1 | 100 | 348.4 | 270.2 | 449.2 | 6.1 | 1129.2 | 124 | 123 | 99.2 | 95.6 | 100 | 359.9 | 297.6 | 435.1 | <0.5 | 1958.8 |
|  | Control vaccine | Screening | 27 | 2 | 7.4 | 0.9 | 24.3 | 0.3 | 0.2 | 0.3 | <0.5 | 1.0 | 63 | 2 | 3.2 | 0.4 | 11.0 | 0.3 | 0.2 | 0.3 | <0.5 | 1.0 |
|  |  | One month post dose-3 | 27 | 1 | 3.7 | 0.1 | 19.0 | 0.3 | 0.2 | 0.5 | <0.5 | 79.5 | 62 | 2 | 3.2 | 0.4 | 11.2 | 0.3 | 0.2 | 0.4 | <0.5 | 1217.8 |
| Agogo | RTS,S/AS01 | Screening | 120 | 11 | 9.2 | 4.7 | 15.8 | 0.3 | 0.3 | 0.3 | <0.5 | 4.8 | 132 | 11 | 8.3 | 4.2 | 14.4 | 0.3 | 0.3 | 0.3 | <0.5 | 4.8 |
|  |  | One month post dose-3 | 119 | 119 | 100 | 96.9 | 100 | 665.5 | 591.4 | 749.0 | 186.6 | 4286.0 | 131 | 131 | 100 | 97.2 | 100 | 666.7 | 595.6 | 746.2 | 186.6 | 4286.0 |
|  | Control vaccine | Screening | 62 | 4 | 6.5 | 1.8 | 15.7 | 0.3 | 0.2 | 0.3 | <0.5 | 3.7 | 68 | 4 | 5.9 | 1.6 | 14.4 | 0.3 | 0.2 | 0.3 | <0.5 | 3.7 |
|  |  | One month post dose-3 | 61 | 2 | 3.3 | 0.4 | 11.3 | 0.3 | 0.2 | 0.3 | <0.5 | 0.8 | 67 | 3 | 4.5 | 0.9 | 12.5 | 0.3 | 0.2 | 0.3 | <0.5 | 0.8 |
| Kombewa | RTS,S/AS01 | Screening | 113 | 19 | 16.8 | 10.4 | 25.0 | 0.3 | 0.3 | 0.4 | <0.5 | 7.7 | 125 | 19 | 15.2 | 9.4 | 22.7 | 0.3 | 0.3 | 0.4 | <0.5 | 7.7 |
|  |  | One month post dose-3 | 109 | 109 | 100 | 96.7 | 100 | 745.1 | 648.1 | 856.6 | 32.9 | 8147.2 | 116 | 116 | 100 | 96.9 | 100 | 716.1 | 624.3 | 821.5 | 32.9 | 8147.2 |
|  | Control vaccine | Screening | 72 | 8 | 11.1 | 4.9 | 20.7 | 0.3 | 0.3 | 0.3 | <0.5 | 1.3 | 75 | 8 | 10.7 | 4.7 | 19.9 | 0.3 | 0.3 | 0.3 | <0.5 | 1.3 |
|  |  | One month post dose-3 | 70 | 3 | 4.3 | 0.9 | 12.0 | 0.3 | 0.2 | 0.3 | <0.5 | 260.7 | 72 | 3 | 4.2 | 0.9 | 11.7 | 0.3 | 0.2 | 0.3 | <0.5 | 260.7 |
| Kintampo | RTS,S/AS01 | Screening | 115 | 17 | 14.8 | 8.9 | 22.6 | 0.3 | 0.3 | 0.3 | <0.5 | 4.2 | 132 | 21 | 15.9 | 10.1 | 23.3 | 0.3 | 0.3 | 0.3 | <0.5 | 4.2 |
|  |  | One month post dose-3 | 114 | 114 | 100 | 96.8 | 100 | 787.1 | 682.6 | 907.6 | 14.4 | 4811.6 | 130 | 130 | 100 | 97.2 | 100 | 725.5 | 631.5 | 833.5 | 14.4 | 4811.6 |
|  | Control vaccine | Screening | 60 | 7 | 11.7 | 4.8 | 22.6 | 0.3 | 0.3 | 0.4 | <0.5 | 16.2 | 68 | 9 | 13.2 | 6.2 | 23.6 | 0.3 | 0.3 | 0.4 | <0.5 | 16.2 |
|  |  | One month post dose-3 | 56 | 9 | 16.1 | 7.6 | 28.3 | 0.4 | 0.3 | 0.5 | <0.5 | 10.4 | 64 | 11 | 17.2 | 8.9 | 28.7 | 0.4 | 0.3 | 0.5 | <0.5 | 10.4 |
| Nanoro | RTS,S/AS01 | Screening | 138 | 41 | 29.7 | 22.2 | 38.1 | 0.4 | 0.3 | 0.5 | <0.5 | 6.5 | 140 | 41 | 29.3 | 21.9 | 37.6 | 0.4 | 0.3 | 0.5 | <0.5 | 6.5 |
|  |  | One month post dose-3 | 138 | 138 | 100 | 97.4 | 100 | 705.1 | 628.6 | 791.0 | 112.0 | 4503.6 | 139 | 139 | 100 | 97.4 | 100 | 685.8 | 604.3 | 778.4 | 14.9 | 4503.6 |
|  | Control vaccine | Screening | 60 | 11 | 18.3 | 9.5 | 30.4 | 0.4 | 0.3 | 0.4 | <0.5 | 4.6 | 60 | 11 | 18.3 | 9.5 | 30.4 | 0.4 | 0.3 | 0.4 | <0.5 | 4.6 |
|  |  | One month post dose-3 | 59 | 4 | 6.8 | 1.9 | 16.5 | 0.3 | 0.2 | 0.3 | <0.5 | 1.2 | 59 | 4 | 6.8 | 1.9 | 16.5 | 0.3 | 0.2 | 0.3 | <0.5 | 1.2 |
| Siaya | RTS,S/AS01 | Screening | 114 | 31 | 27.2 | 19.3 | 36.3 | 0.4 | 0.3 | 0.4 | <0.5 | 5.1 | 132 | 33 | 25.0 | 17.9 | 33.3 | 0.4 | 0.3 | 0.4 | <0.5 | 8.9 |
|  |  | One month post dose-3 | 114 | 113 | 99.1 | 95.2 | 100 | 708.6 | 573.8 | 875.0 | <0.5 | 6242.9 | 127 | 126 | 99.2 | 95.7 | 100 | 677.4 | 548.2 | 837.1 | <0.5 | 6242.9 |
|  | Control vaccine | Screening | 55 | 9 | 16.4 | 7.8 | 28.8 | 0.3 | 0.3 | 0.4 | <0.5 | 2.9 | 66 | 10 | 15.2 | 7.5 | 26.1 | 0.3 | 0.3 | 0.4 | <0.5 | 2.9 |
|  |  | One month post dose-3 | 54 | 4 | 7.4 | 2.1 | 17.9 | 0.3 | 0.2 | 0.3 | <0.5 | 8.2 | 63 | 5 | 7.9 | 2.6 | 17.6 | 0.3 | 0.3 | 0.3 | <0.5 | 8.2 |
| Overall | RTS,S/AS01 | Screening | 1036 | 132 | 12.7 | 10.8 | 14.9 | 0.3 | 0.3 | 0.3 | <0.5 | 7.7 | 1447 | 151 | 10.4 | 8.9 | 12.1 | 0.3 | 0.3 | 0.3 | <0.5 | 15.3 |
|  |  | One month post dose-3 | 1034 | 1033 | 99.9 | 99.5 | 100 | 621.0 | 591.5 | 651.9 | <0.5 | 8147.2 | 1378 | 1375 | 99.8 | 99.4 | 100 | 570.3 | 543.7 | 598.3 | <0.5 | 8147.2 |
|  | Control vaccine | Screening | 526 | 47 | 8.9 | 6.6 | 11.7 | 0.3 | 0.3 | 0.3 | <0.5 | 16.2 | 706 | 53 | 7.5 | 5.7 | 9.7 | 0.3 | 0.3 | 0.3 | <0.5 | 16.2 |
|  |  | One month post dose-3 | 529 | 31 | 5.9 | 4.0 | 8.2 | 0.3 | 0.3 | 0.3 | <0.5 | 329.9 | 686 | 38 | 5.5 | 3.9 | 7.5 | 0.3 | 0.3 | 0.3 | <0.5 | 1217.8 |

GMT= geometric mean antibody titer calculated on all subjects.

Seropositivity = anti-CS titer equal or greater than 0.5 EU/mL.

N = number of subjects with available results.

n/% = number/percentage of subjects with titer equal to or above specified value.

95% CI = 95% confidence interval; LL = Lower Limit, UL = Upper Limit.

Min/Max = Minimum/Maximum.

## Supplementary table 16b. Seropositivity rates and GMTs for anti-CS antibodies at baseline and one month post dose-3 in the 6-12 weeks age category by site and overall

|  | | | **Per-protocol population** | | | | | | | | | | **Intention-to-treat population** | | | | | | | | | |
| --- | --- | --- | --- | --- | --- | --- | --- | --- | --- | --- | --- | --- | --- | --- | --- | --- | --- | --- | --- | --- | --- | --- |
|  | | |  | **Seropositivity** | | | | **GMT** | | |  | |  | **Seropositivity** | | | | **GMT** | | |  | |
|  |  |  |  |  | | **95% CI** | |  | **95% CI** | |  |  |  |  | | **95% CI** | |  | **95% CI** | |  |  |
| **Site** | **Group** | **Timing** | **N** | **n** | **%** | **LL** | **UL** | **value** | **LL** | **UL** | **Min** | **Max** | **N** | **n** | **%** | **LL** | **UL** | **value** | **LL** | **UL** | **Min** | **Max** |
| Kilifi | RTS,S/AS01 | Screening | 107 | 35 | 32.7 | 24.0 | 42.5 | 0.4 | 0.3 | 0.4 | <0.5 | 4.0 | 117 | 38 | 32.5 | 24.1 | 41.8 | 0.4 | 0.3 | 0.4 | <0.5 | 9.5 |
|  |  | One month post dose-3 | 109 | 109 | 100 | 96.7 | 100 | 254.4 | 206.7 | 313.2 | 0.9 | 2493.9 | 115 | 115 | 100 | 96.8 | 100 | 247.2 | 201.5 | 303.4 | 0.9 | 2493.9 |
|  | Control vaccine | Screening | 54 | 12 | 22.2 | 12.0 | 35.6 | 0.4 | 0.3 | 0.4 | <0.5 | 5.6 | 58 | 14 | 24.1 | 13.9 | 37.2 | 0.4 | 0.3 | 0.5 | <0.5 | 5.6 |
|  |  | One month post dose-3 | 61 | 2 | 3.3 | 0.4 | 11.3 | 0.3 | 0.2 | 0.3 | <0.5 | 0.6 | 63 | 2 | 3.2 | 0.4 | 11.0 | 0.3 | 0.2 | 0.3 | <0.5 | 0.6 |
| Korogwe | RTS,S/AS01 | Screening | 111 | 30 | 27.0 | 19.0 | 36.3 | 0.3 | 0.3 | 0.4 | <0.5 | 1.8 | 127 | 32 | 25.2 | 17.9 | 33.7 | 0.3 | 0.3 | 0.4 | <0.5 | 1.8 |
|  |  | One month post dose-3 | 106 | 106 | 100 | 96.6 | 100 | 252.1 | 217.7 | 292.0 | 17.9 | 1279.7 | 117 | 116 | 99.1 | 95.3 | 100 | 231.6 | 192.4 | 278.8 | <0.5 | 1279.7 |
|  | Control vaccine | Screening | 66 | 17 | 25.8 | 15.8 | 38.0 | 0.4 | 0.3 | 0.4 | <0.5 | 3.2 | 73 | 19 | 26.0 | 16.5 | 37.6 | 0.3 | 0.3 | 0.4 | <0.5 | 3.2 |
|  |  | One month post dose-3 | 63 | 2 | 3.2 | 0.4 | 11.0 | 0.3 | 0.2 | 0.3 | <0.5 | 4.5 | 65 | 2 | 3.1 | 0.4 | 10.7 | 0.3 | 0.2 | 0.3 | <0.5 | 4.5 |
| Manhiça | RTS,S/AS01 | Screening | 120 | 6 | 5.0 | 1.9 | 10.6 | 0.3 | 0.3 | 0.3 | <0.5 | 3.1 | 136 | 8 | 5.9 | 2.6 | 11.3 | 0.3 | 0.3 | 0.3 | <0.5 | 3.1 |
|  |  | One month post dose-3 | 108 | 108 | 100 | 96.6 | 100 | 335.3 | 289.5 | 388.5 | 36.0 | 2306.7 | 116 | 116 | 100 | 96.9 | 100 | 326.5 | 280.1 | 380.5 | 12.7 | 2306.7 |
|  | Control vaccine | Screening | 61 | 4 | 6.6 | 1.8 | 15.9 | 0.3 | 0.3 | 0.3 | <0.5 | 0.7 | 64 | 4 | 6.3 | 1.7 | 15.2 | 0.3 | 0.3 | 0.3 | <0.5 | 0.7 |
|  |  | One month post dose-3 | 53 | 1 | 1.9 | 0.0 | 10.1 | 0.3 | 0.2 | 0.3 | <0.5 | 0.6 | 54 | 1 | 1.9 | 0.0 | 9.9 | 0.3 | 0.2 | 0.3 | <0.5 | 0.6 |
| Lambarene | RTS,S/AS01 | Screening | 101 | 12 | 11.9 | 6.3 | 19.8 | 0.3 | 0.3 | 0.3 | <0.5 | 4.0 | 112 | 13 | 11.6 | 6.3 | 19.0 | 0.3 | 0.3 | 0.3 | <0.5 | 4.0 |
|  |  | One month post dose-3 | 110 | 110 | 100 | 96.7 | 100 | 287.6 | 248.8 | 332.3 | 22.8 | 1900.7 | 121 | 121 | 100 | 97.0 | 100 | 283.2 | 247.1 | 324.5 | 22.8 | 1900.7 |
|  | Control vaccine | Screening | 41 | 2 | 4.9 | 0.6 | 16.5 | 0.3 | 0.2 | 0.3 | <0.5 | 1.1 | 54 | 3 | 5.6 | 1.2 | 15.4 | 0.3 | 0.2 | 0.3 | <0.5 | 1.1 |
|  |  | One month post dose-3 | 45 | 5 | 11.1 | 3.7 | 24.1 | 0.3 | 0.3 | 0.4 | <0.5 | 3.7 | 53 | 8 | 15.1 | 6.7 | 27.6 | 0.3 | 0.3 | 0.4 | <0.5 | 7.4 |
| Bagamoyo | RTS,S/AS01 | Screening | 120 | 31 | 25.8 | 18.3 | 34.6 | 0.3 | 0.3 | 0.4 | <0.5 | 1.6 | 136 | 35 | 25.7 | 18.6 | 33.9 | 0.3 | 0.3 | 0.4 | <0.5 | 1.7 |
|  |  | One month post dose-3 | 116 | 114 | 98.3 | 93.9 | 99.8 | 179.1 | 143.1 | 224.0 | <0.5 | 1011.1 | 127 | 124 | 97.6 | 93.3 | 99.5 | 162.8 | 128.6 | 206.1 | <0.5 | 1011.1 |
|  | Control vaccine | Screening | 48 | 12 | 25.0 | 13.6 | 39.6 | 0.3 | 0.3 | 0.4 | <0.5 | 1.6 | 60 | 15 | 25.0 | 14.7 | 37.9 | 0.3 | 0.3 | 0.4 | <0.5 | 1.6 |
|  |  | One month post dose-3 | 49 | 3 | 6.1 | 1.3 | 16.9 | 0.3 | 0.2 | 0.4 | <0.5 | 62.8 | 58 | 4 | 6.9 | 1.9 | 16.7 | 0.3 | 0.2 | 0.4 | <0.5 | 62.8 |
| Lilongwe | RTS,S/AS01 | Screening | 95 | 26 | 27.4 | 18.7 | 37.5 | 0.4 | 0.3 | 0.4 | <0.5 | 3.6 | 115 | 31 | 27.0 | 19.1 | 36.0 | 0.4 | 0.3 | 0.4 | <0.5 | 4.8 |
|  |  | One month post dose-3 | 100 | 100 | 100 | 96.4 | 100 | 235.5 | 200.9 | 276.0 | 6.2 | 5210.1 | 113 | 113 | 100 | 96.8 | 100 | 218.4 | 179.6 | 265.4 | 0.5 | 5210.1 |
|  | Control vaccine | Screening | 49 | 18 | 36.7 | 23.4 | 51.7 | 0.4 | 0.3 | 0.5 | <0.5 | 3.1 | 60 | 18 | 30.0 | 18.8 | 43.2 | 0.4 | 0.3 | 0.5 | <0.5 | 3.1 |
|  |  | One month post dose-3 | 55 | 2 | 3.6 | 0.4 | 12.5 | 0.3 | 0.2 | 0.3 | <0.5 | 1.9 | 65 | 4 | 6.2 | 1.7 | 15.0 | 0.3 | 0.3 | 0.3 | <0.5 | 2.5 |
| Agogo | RTS,S/AS01 | Screening | 114 | 44 | 38.6 | 29.6 | 48.2 | 0.4 | 0.4 | 0.5 | <0.5 | 3.2 | 130 | 50 | 38.5 | 30.1 | 47.4 | 0.4 | 0.4 | 0.5 | <0.5 | 3.2 |
|  |  | One month post dose-3 | 114 | 114 | 100 | 96.8 | 100 | 158.6 | 129.1 | 194.8 | 0.6 | 1115.0 | 127 | 127 | 100 | 97.1 | 100 | 151.1 | 123.4 | 185.0 | 0.6 | 1115.0 |
|  | Control vaccine | Screening | 68 | 34 | 50.0 | 37.6 | 62.4 | 0.5 | 0.4 | 0.6 | <0.5 | 5.2 | 70 | 36 | 51.4 | 39.2 | 63.6 | 0.5 | 0.4 | 0.7 | <0.5 | 5.2 |
|  |  | One month post dose-3 | 68 | 6 | 8.8 | 3.3 | 18.2 | 0.3 | 0.3 | 0.3 | <0.5 | 3.2 | 69 | 6 | 8.7 | 3.3 | 18.0 | 0.3 | 0.3 | 0.3 | <0.5 | 3.2 |
| Kombewa | RTS,S/AS01 | Screening | 118 | 32 | 27.1 | 19.3 | 36.1 | 0.4 | 0.3 | 0.4 | <0.5 | 6.0 | 135 | 36 | 26.7 | 19.4 | 35.0 | 0.4 | 0.3 | 0.4 | <0.5 | 6.0 |
|  |  | One month post dose-3 | 117 | 117 | 100 | 96.9 | 100 | 242.3 | 199.7 | 294.1 | 6.0 | 4312.0 | 123 | 121 | 98.4 | 94.2 | 99.8 | 201.5 | 156.1 | 260.1 | <0.5 | 4312.0 |
|  | Control vaccine | Screening | 62 | 21 | 33.9 | 22.3 | 47.0 | 0.4 | 0.3 | 0.5 | <0.5 | 3.6 | 65 | 22 | 33.8 | 22.6 | 46.6 | 0.4 | 0.3 | 0.5 | <0.5 | 3.6 |
|  |  | One month post dose-3 | 60 | 4 | 6.7 | 1.8 | 16.2 | 0.3 | 0.2 | 0.3 | <0.5 | 3.1 | 61 | 4 | 6.6 | 1.8 | 15.9 | 0.3 | 0.2 | 0.3 | <0.5 | 3.1 |
| Kintampo | RTS,S/AS01 | Screening | 117 | 68 | 58.1 | 48.6 | 67.2 | 0.7 | 0.6 | 0.8 | <0.5 | 5.0 | 134 | 75 | 56.0 | 47.1 | 64.5 | 0.6 | 0.5 | 0.7 | <0.5 | 5.0 |
|  |  | One month post dose-3 | 115 | 115 | 100 | 96.8 | 100 | 151.0 | 128.5 | 177.4 | 9.4 | 1731.5 | 127 | 127 | 100 | 97.1 | 100 | 148.0 | 127.7 | 171.6 | 9.4 | 1731.5 |
|  | Control vaccine | Screening | 54 | 32 | 59.3 | 45.0 | 72.4 | 0.6 | 0.5 | 0.8 | <0.5 | 3.0 | 65 | 38 | 58.5 | 45.6 | 70.6 | 0.6 | 0.5 | 0.8 | <0.5 | 3.0 |
|  |  | One month post dose-3 | 51 | 1 | 2.0 | 0.0 | 10.4 | 0.3 | 0.2 | 0.3 | <0.5 | 1.8 | 59 | 1 | 1.7 | 0.0 | 9.1 | 0.3 | 0.2 | 0.3 | <0.5 | 1.8 |
| Nanoro | RTS,S/AS01 | Screening | 132 | 95 | 72.0 | 63.5 | 79.4 | 1.0 | 0.8 | 1.2 | <0.5 | 45.0 | 135 | 97 | 71.9 | 63.5 | 79.2 | 1.0 | 0.8 | 1.2 | <0.5 | 45.0 |
|  |  | One month post dose-3 | 131 | 131 | 100 | 97.2 | 100 | 116.9 | 92.5 | 147.8 | 1.1 | 1574.8 | 132 | 132 | 100 | 97.2 | 100 | 115.0 | 90.9 | 145.5 | 1.1 | 1574.8 |
|  | Control vaccine | Screening | 64 | 42 | 65.6 | 52.7 | 77.1 | 0.8 | 0.6 | 1.0 | <0.5 | 6.2 | 65 | 43 | 66.2 | 53.4 | 77.4 | 0.8 | 0.6 | 1.0 | <0.5 | 7.9 |
|  |  | One month post dose-3 | 64 | 3 | 4.7 | 1.0 | 13.1 | 0.3 | 0.2 | 0.3 | <0.5 | 0.9 | 65 | 4 | 6.2 | 1.7 | 15.0 | 0.3 | 0.3 | 0.3 | <0.5 | 0.9 |
| Siaya | RTS,S/AS01 | Screening | 99 | 45 | 45.5 | 35.4 | 55.8 | 0.5 | 0.4 | 0.6 | <0.5 | 6.5 | 129 | 57 | 44.2 | 35.5 | 53.2 | 0.5 | 0.4 | 0.5 | <0.5 | 6.5 |
|  |  | One month post dose-3 | 95 | 94 | 98.9 | 94.3 | 100 | 244.1 | 189.2 | 315.0 | <0.5 | 3366.4 | 111 | 109 | 98.2 | 93.6 | 99.8 | 208.2 | 157.3 | 275.4 | <0.5 | 3366.4 |
|  | Control vaccine | Screening | 60 | 27 | 45.0 | 32.1 | 58.4 | 0.5 | 0.4 | 0.6 | <0.5 | 5.9 | 71 | 31 | 43.7 | 31.9 | 56.0 | 0.5 | 0.4 | 0.6 | <0.5 | 5.9 |
|  |  | One month post dose-3 | 58 | 7 | 12.1 | 5.0 | 23.3 | 0.3 | 0.3 | 0.4 | <0.5 | 9.7 | 66 | 8 | 12.1 | 5.4 | 22.5 | 0.3 | 0.3 | 0.4 | <0.5 | 9.7 |
| Overall | RTS,S/AS01 | Screening | 1234 | 424 | 34.4 | 31.7 | 37.1 | 0.4 | 0.4 | 0.4 | <0.5 | 45.0 | 1406 | 472 | 33.6 | 31.1 | 36.1 | 0.4 | 0.4 | 0.4 | <0.5 | 45.0 |
|  |  | One month post dose-3 | 1221 | 1218 | 99.8 | 99.3 | 99.9 | 210.5 | 198.2 | 223.6 | <0.5 | 5210.1 | 1329 | 1321 | 99.4 | 98.8 | 99.7 | 197.4 | 185.2 | 210.4 | <0.5 | 5210.1 |
|  | Control vaccine | Screening | 627 | 221 | 35.2 | 31.5 | 39.1 | 0.4 | 0.4 | 0.5 | <0.5 | 6.2 | 705 | 243 | 34.5 | 31.0 | 38.1 | 0.4 | 0.4 | 0.4 | <0.5 | 7.9 |
|  |  | One month post dose-3 | 627 | 36 | 5.7 | 4.1 | 7.9 | 0.3 | 0.3 | 0.3 | <0.5 | 62.8 | 678 | 44 | 6.5 | 4.8 | 8.6 | 0.3 | 0.3 | 0.3 | <0.5 | 62.8 |

GMT= geometric mean antibody titer calculated on all subjects.

Seropositivity = anti-CS titer equal or greater than 0.5 EU/mL.

N = number of subjects with available results.

n/% = number/percentage of subjects with titer equal to or above specified value.

95% CI = 95% confidence interval; LL = Lower Limit, UL = Upper Limit.

Min/Max = Minimum/Maximum.
