# Supplementary material for: Efficacy and Safety of the RTS,S/AS01 Malaria Vaccine during 18 Months after Vaccination: A Phase 3 Randomized, Controlled Trial in Children and Young Infants at 11 African Sites
Source: PLoS Med. 2014 Jul 29;11(7):e1001685. doi: 10.1371/journal.pmed.1001685 (PMC4114488; doi:10.1371/journal.pmed.1001685)
Supplement: Table S17 — Overview of the analyses reported. (DOCX) [file pmed.1001685.s026.docx]

## Supplementary table 17. Overview of the analyses reported

| **Protocol analyses performed at Month 20** | **Reported in this manuscript** |
| --- | --- |
| Vaccine efficacy against all episodes of clinical malaria (primary case definition and secondary 1, 2 and 3) over 18 months post dose-3 by site and overall (per-protocol & ITT populations) | Presented in Table 1, Figure 3 and Supplementary table 6 |
| Vaccine efficacy against first or only episode of clinical malaria (primary case definition and secondary 1) over 18 months post dose-3 by site and overall (per-protocol & ITT populations) | Primary case definition _ Overall (per-protocol population) presented in Supplementary figure 3 |
| Vaccine efficacy against severe malaria (primary case definition and secondary 1, 2 and 3), incident anemia (case definition 1, 2, 3), malaria hospitalization (case definition 1 and 2) and fatal malaria (primary case definition and secondary 1, 2 and 3) over 18 months post dose-3 by site and overall (per-protocol & ITT populations) | Severe malaria and malaria hospitalization_Overall presented in Table 1  Fatal malaria _Overall presented in Supplementary table 11 |
| VE against prevalent parasitemia and prevalent anemia at Month 20 by site and overall (per-protocol & ITT populations) | Presented in Supplementary table 8 |
| Evolution of VE over time against all episodes of clinical malaria (primary case definition) by site and overall (per-protocol population) | Presented in Supplementary figure 4 |
| Evolution of VE over time against first or only episode of clinical malaria (primary case definition) by site and overall (per-protocol population) | Not presented ^1^ |
| Effect on growth: anthropometry at Month 20 by site and overall (per-protocol & ITT populations) | Overall presented in Supplementary table 10 |
| Vaccine efficacy against sepsis (case definition 1 and 2) over 18 months post dose-3 by site and overall (per-protocol & ITT populations) | Case definition 1_Overall presented in Supplementary table 11 |
| Vaccine efficacy against pneumonia (primary case definition and secondary 1, 2 and 3) over 18 months post dose-3 by site and overall (per-protocol & ITT populations) | Primary case definition_Overall presented in Supplementary table 11 |
| Vaccine efficacy against all-cause hospitalization (primary case definition) over 18 months post dose-3 by site and overall (per-protocol & ITT populations) | Primary case definition_Overall presented in Table 1 |
| Vaccine efficacy against all-cause mortality (case definition 1 and 2) over 18 months post dose-3 by site and overall (per-protocol & ITT populations) | Case definition 1_Overall presented in Supplementary table 11 |
| Anti-CS seropositivity rates and GMTs at baseline and one month post dose-3 by site and overall (per-protocol & ITT populations) | Presented in Figure 5 and Supplementary table 17 |
| Occurence of serious adverse events (SAEs) from dose-1 up to Month 20 (ITT population) | Presented in Supplementary table 15 |
| Occurence of fatal SAEs from dose-1 up to Month 20 (ITTpopulation) | Overall_Presented in Supplementary table 15 |
| Occurence of related SAEs from dose-1 up to Month 20 (ITT population) | Overall_Presented in Supplementary table 15 |
| Anti-CS seropositivity rates and GMTs at baseline and one month post dose-3 by weight for age at baseline (per-protocol & ITT populations) | Not presented ^2^ |
| Occurence of SAEs in low-weight for age and very low weight for age subjects (ITT population) | Not presented ^2^ |
| Occurence of fatal SAEs in low-weight for age and very low weight for age subjects (ITT population) | Not presented ^2^ |
| Occurence of related SAEs in low-weight for age and very low weight for age subjects (ITT population) | Not presented-^2^ |
| **Additional* analyses performed at Month 20** | **Reported in this manuscript** |
| Vaccine efficacy against all episodes of clinical malaria (primary case definition and secondary 1) over 12 months post dose-3 by site and overall (per-protocol & ITT populations) | Presented in Supplementary figure 8 (Primary case def) and Supplementary table 16 |
| Vaccine efficacy against first or only episode of clinical malaria (primary case definition and secondary 1) over 12 months post dose-3 by site and overall (per-protocol & ITT populations) | Overall_ presented in Supplementary table 16 |
| Vaccine efficacy against severe malaria (primary case definition and secondary 1), incident anemia (case definition 1, 2, 3), malaria hospitalization (case definition 1 and 2) and fatal malaria (primary case definition and secondary 1, 2, 3) over 12 months post dose-3 by site and overall (per-protocol & ITT populations) | Not presented ^3^ |
| All episodes of clinical malaria (primary case definition and secondary 1) by 6-monthly periods by site and overall (per-protocol & ITT populations) | Primary case definition (per-protocol population) presented in Supplementary figure 5 |
| All episodes of severe malaria (primary case definition and secondary 1) by 6-monthly periods by site and overall (per-protocol & ITT populations) | Primary case definition (per-protocol population) presented in Supplementary figure 5 |
| Cases averted of clinical malaria (primary case definition and secondary 1) by 6-monhly period by site and overall (per-protocol & ITT populations) | Presented in Figure 2 and Supplementary table 9 |
| Cases averted of severe malaria (primary case definition and secondary 1) by 6-monhly period by site and overall (per-protocol & ITT populations) | Presented in Figure 2 and Supplementary table 9 |
| Cases averted of malaria hospitalization (case definition 1 and 2) by 6-monhly period by site and overall (per-protocol & ITT populations) | Presented in Figure 2 and Supplementary table 9 |
| **Cases averted of all-cause hospitalization (primary case definition) by 6-monhly period by site and overall (per-protocol & ITT populations) | Presented in Figure 2 and Supplementary table 9 |
| Exploratory model: Determinants of incidence of clinical malaria over 18 months post dose-3 (primary case definition, all episodes) full model + final model (per-protocol population) | Presented in Supplementary table 7a,b,c,d |
| Exploratory model: Determinants of incidence of clinical malaria over 12 months post dose-3 (primary case definition, all episodes) final model + final model (per-protocol population) | Not presented ^3^ |
| Exploratory model: determinants of anti-CS over 18 months post dose-3 (per-protocol population) | Presented in Supplementary tables 12, 13 and 14 |
| Exploratory model: determinants of anti-CS over 12 months post dose-3 (per-protocol population) | Not presented ^3^ |

* The additional analyses were added during the development of the statistical analysis plan, prior to the performance of any statistical analysis for the Month 20.

1. Analysis no presented because the VE over time against multiple episodes is presented.

2. Analyses in low weight for age children and infants are not presented because they were less relevant for this manuscript that aims at presenting efficacy, safety and efficacy by site in the whole population of subjects enrolled. These results are available on GSK Clinical Study Register (Study ID: 110021): <http://www.gsk-clinicalstudyregister.com/>

3. Analyses not presented over 12 months post dose-3 because the same analyses are presented over a longer follow-up period (18 months post dose-3).
