# Supplementary material for: Efficacy and Safety of the RTS,S/AS01 Malaria Vaccine during 18 Months after Vaccination: A Phase 3 Randomized, Controlled Trial in Children and Young Infants at 11 African Sites
Source: PLoS Med. 2014 Jul 29;11(7):e1001685. doi: 10.1371/journal.pmed.1001685 (PMC4114488; doi:10.1371/journal.pmed.1001685)
Supplement: Protocol S1 — A phase III, double-blind (observer-blind), randomized, controlled multicenter study to evaluate, in infants and children, the efficacy of the RTS,S/AS01 candidate vaccine against malaria disease caused by P. falciparum infection, across diverse malaria transmission settings in Africa. (PDF) [file pmed.1001685.s028.pdf]

Sponsor:
GlaxoSmithKline Biologicals
Rue de l'Institut 89, 1330 Rixensart,
Belgium

**Study vaccine number**	257049 (GlaxoSmithKline Biologicals' candidate *Plasmodium falciparum* malaria vaccine RTS,S/AS01E).
**Study vaccines**	Cell Culture Rabies Vaccine from two manufacturers:<br>HDC or Purified Vero cell Rabies Vaccine (Sanofi Pasteur); PCEC Rabies Vaccine (Rabipur or equivalent, Novartis).<br>DTPwHepB/Hib vaccine: Tritanrix HepB™/Hib (GSK)<br>Oral Polio Vaccine: Polio Sabin™ (GSK).<br>Meningococcal C Conjugate Vaccine from three manufacturers:<br>Meningitec (Wyeth); NeisVac-C (Baxter); Menjugate (Novartis).<br>Amended 24 October 2008
**eTrack study number and abbreviated title**	110021 (Malaria-055 PRI)
**Date of protocol**	Final 10 March 2008
**Date of amendments**	Amendment 1, 19 August 2008<br>Amendment 2, 24 October 2008<br>Amendment 3, 26 November 2009<br>Amendment 4, 01 December 2010<br>Amendment 5, 23 January 2012<br>***Amendment 6, 08 August 2012***
**Title**	Efficacy of GSK Biologicals' candidate malaria vaccine (257049) against malaria disease caused by *P. falciparum* infection in infants and children in Africa.
**Detailed Title**	A phase III, double blind (observer-blind), randomized, controlled multi-center study to evaluate, in infants and children, the efficacy of the RTS,S/AS01E candidate vaccine against malaria disease caused by *P. falciparum* infection, across diverse malaria transmission settings in Africa.

GlaxoSmithKline Biologicals

Co-ordinating author *PII - blinded*, Scientific Writer.

Contributing authors

- *PII - blinded*, Central Study Coordinator.
- *PII - blinded*, Vice President–Global Clinical R&D.
- *PII - blinded*, Clinical Data Management.
- *PII - blinded*, Vice President–Vaccines for Emerging Diseases.
- *PII - blinded*, Clinical Immunology Read Outs.
- *PII - blinded*, Project Manager.
- *PII - blinded*, Clinical Development Manager.
- *PII - blinded*, Statistician.
- *PII - blinded*, Clinical Development Manager.
- *PII - blinded*, Central Study Coordinator.
- *PII - blinded*, Scientific Writer.
- *PII - blinded*, Central Study Coordinator.
- *PII - blinded*, Central Study Coordinator.
- *PII - blinded*, Quality Assurance.
- *PII - blinded*, Safety Physician.
- *PII - blinded*, Regulatory Affairs.
- *PII - blinded*, US Regulatory Affairs.
- *PII - blinded*, Safety Physician.
- *PII - blinded*, Clinical Regulatory Affairs.
- *PII - blinded*, Clinical Development Manager.
- *PII - blinded*, Clinical Planning Manager.

GSK Biologicals' Protocol DS V 12.4

Copyright 2008-2012 the GlaxoSmithKline group of companies.

**eTrack study number and abbreviated title**	110021 (Malaria-055 PRI)
**Date of protocol**	Final 10 March 2008
**Date of amendments**	Amendment 1, 19 August 2008<br>Amendment 2, 24 October 2008<br>Amendment 3, 26 November 2009<br>Amendment 4, 01 December 2010<br>Amendment 5, 23 January 2012<br>***Amendment 6, 08 August 2012***

Program for Appropriate Technology in Health (PATH) Malaria Vaccine Initiative (MVI) Contributing Authors

Contributing authors

- *PII - blinded*, Technical Coordinator.
- *PII - blinded*, Senior Program Officer.
- *PII - blinded*, Program Officer.
- *PII - blinded*, Technical Coordinator.
- *PII - blinded*, Director, Portfolio Management.

GlaxoSmithKline Biologicals will act as regulatory sponsor for this trial

Protocol Amendment 6 Sponsor Signatory Approval

**eTrack study number and abbreviated title**	110021 (Malaria-055 PRI).
**Date of protocol amendment**	Amendment 6 Final: 08 August 2012
**Detailed title**	A phase III, double blind (observer-blind), randomized, controlled multi-center study to evaluate, in infants and children, the efficacy of the RTS,S/AS01E candidate vaccine against malaria disease caused by *P. falciparum* infection, across diverse malaria transmission settings in Africa.
**Sponsor signatory**	*PII - blinded*,<br><br>Director, Clinical Development Manager
**Signature**	--
**Date**	--

Protocol Amendment 6 Rationale

**Amendment number:**	Amendment 6
**Rationale/background for changes:**<br><br>At the European Medicines Agency's (EMA) request, GSK Biologicals has updated its procedure for emergency unblinding during the conduct of a clinical study.<br><br>According to the revised procedure, the responsibility and the decision to break the treatment code in emergency situations resides solely with the investigator and consequently, the investigator will have full authority to break the treatment code.<br><br>The text in section 6.5 "Method of blinding and breaking the study blind" has been modified accordingly.<br><br>In addition, the timings for the intervals between study visits during the extension were not consistent within the document and have been corrected or more specified to increase clarity.	
**Amended text has been included in *bold italics*, deletions by ~~strikethrough~~.**	

Protocol Amendment 6 Investigator Agreement

I agree:

- To assume responsibility for the proper conduct of the study at this site.
- To conduct the study in compliance with this protocol, any mutually agreed future protocol amendments, and with any other study conduct procedures provided by GlaxoSmithKline Biologicals (GSK Biologicals).
- To ensure that all persons assisting me with the study are adequately informed about the GSK Biologicals investigational product(s) and other study-related duties and functions as described in the protocol.
- Not to implement any changes to the protocol without agreement from the sponsor and prior review and written approval from the Institutional Review Board (IRB) or Independent Ethics Committee (IEC), except where necessary to eliminate an immediate hazard to the subjects, or where permitted by all applicable regulatory requirements (for example, for administrative aspects of the study).
- That I am thoroughly familiar with the appropriate use of the vaccine(s), as described in this protocol, and any other information provided by the sponsor, including, but not limited to, the following: the current Investigator's Brochure (IB) or equivalent document, IB supplement (if applicable), prescribing information (in the case of a marketed vaccine).
- That I am aware of, and will comply with, "Good Clinical Practice" (GCP) and all applicable regulatory requirements.
- That I have been informed that certain regulatory authorities require the sponsor to obtain and supply, as necessary, details about the investigator's ownership interest in the sponsor or the investigational product, and more generally about his/her financial ties with the sponsor. GSK Biologicals will use and disclose the information solely for the purpose of complying with regulatory requirements.

Hence I:

- Agree to supply GSK Biologicals with any necessary information regarding ownership interest and financial ties (including those of my spouse and dependent children).
- Agree to promptly update this information if any relevant changes occur during the course of the study and for 1 year following completion of the study.
- Agree that GSK Biologicals may disclose any information it has about such ownership interests and financial ties to regulatory authorities.
- Agree to provide GSK Biologicals with an updated Curriculum Vitae and other FDA required documents.

**eTrack study number and abbreviated title**	110021 (Malaria-055 PRI).
**Date of protocol amendment**	Amendment 6 Final: 08 August 2012
**Detailed title**	A phase III, double blind (observer-blind), randomized, controlled multi-center study to evaluate, in infants and children, the efficacy of the RTS,S/AS01E candidate vaccine against malaria disease caused by *P. falciparum* infection, across diverse malaria transmission settings in Africa.
**Investigator signatory**	
**Name**	--
**Signature**	--
**Date**	--

Synopsis

Synopsis Amended 19 August 2008
Synopsis Amended 24 October 2008
Synopsis Amended 26 November 2009
Synopsis Amended 01 December 2010
Synopsis Amended 23 January 2012

**Detailed Title**	A phase III, double blind (observer-blind), randomized, controlled multi-center study to evaluate, in infants and children, the efficacy of the RTS,S/AS01E candidate vaccine against malaria disease caused by *P. falciparum* infection, across diverse malaria transmission settings in Africa.
**Study population**	Male and female infants and children aged 6-12 weeks and 5-17 months of age at first vaccination if eligible according to inclusion and exclusion criteria.
**Study population of the extension**	Male and female infants and children enrolled in Malaria-055 PRI and having received at least one dose of study or control vaccine during the primary trial phase.
**Rationale for the study**	This Phase III study of GSK Biologicals candidate malaria vaccine RTS,S/AS01E has been designed to address the key safety and efficacy information required for vaccine licensure. In addition, other disease endpoints that allow the evaluation of the full public health impact and cost effectiveness of vaccine implementation are included. Co-primary objectives will investigate the efficacy against clinical disease in children from 5-17 months of age at first dose and the efficacy in infants 6-12 weeks of age who receive the vaccine in co-administration with EPI antigens (i.e. DTPw Hep B/Hib).<br><br>All participating centers will use standardized case definitions for efficacy endpoints and a structured approach to case-assessment. Cases of clinical disease will be pooled across participating centers to determine the primary endpoint of efficacy against clinical disease in each age category. Secondary objectives will assess the efficacy of the vaccine on severe malaria disease, severe anemia and malaria hospitalization. Analysis by site will allow the evaluation of efficacy under different conditions of malaria transmission and randomization to a booster dose will allow the evaluation of the duration of efficacy of a primary course and the requirement for boosting. Additional objectives include determination of efficacy of the vaccine against all medical hospitalization, non-malaria serious illness, fatal malaria and all-cause mortality. In addition immunological correlates of protection will be investigated.

Analyses are planned at the following timepoints during the trial:

- A primary analysis to investigate efficacy against clinical malaria disease after approximately 6000 children 5-17 months of age at first dose (4000 RTS,S/AS01E recipients) have completed 14 months of follow up post dose 1.
- A primary analysis to investigate efficacy against clinical malaria disease after approximately 6000 infants 6 to 12 weeks of age at first dose (4000 RTS,S/AS01E recipients) have completed 14 months of follow up post dose 1.
- A secondary analysis for evaluation of efficacy against severe disease when there are 250 accumulated cases, or at study end, whichever occurs earlier.
- A secondary analysis for evaluation of efficacy, safety and immunogenicity when all children have completed 18 month follow-up post Dose 3 (Visit 22).
- A secondary analysis for evaluation of the antibody responses to polio serotypes 1, 2 and 3 in the 6-12 weeks age category one month post boost (Visit 23).
- A final analysis will be carried out at the end of the trial and will include further investigations of vaccine efficacy against a range of manifestations of malaria disease and evaluate the booster dose.
- An additional analysis will be conducted at the end of the extension including an evaluation of safety and efficacy against clinical malaria, severe malaria and prevalent parasitemia.

Rationale for the extension

The strategy underlying the development of the RTS,S/AS01E malaria vaccine is that it would target the most vulnerable age group: infants and young children. It would provide protection from the most severe forms of the disease whilst allowing natural blood stage immunity to develop due to continued exposure to infections. However, there is a theoretical concern that the protection conferred by a malaria vaccine in young children may impair their ability to acquire natural immunity to the blood stage form of *P. falciparum* and that, if the vaccine efficacy decreases over time, these children would become vulnerable to malaria. This could result in a higher susceptibility to severe malaria and/or a higher frequency of clinical malaria disease in vaccinated children compared to children of the same age that were not vaccinated. This long term follow-up probably represents the last opportunity to evaluate the duration of vaccine efficacy and the long term safety of RTS,S/AS01E relative to a control group in a large multi-country study.

Mean follow-up time: The last study visit of the extension (Visit 38) is scheduled in December 2013 (interval: Nov 2013-Jan 2014). This means a variable number of months after vaccination by individual children. Based on the actual enrolment, the mean follow-up time will be:

- 5-17 months: 49 months post Dose 1 (range: 41-55)
- 6-12 weeks: 41 months post Dose 1 (range: 32-48)

Objectives **Primary**

Efficacy coprimary objectives: Efficacy against clinical malaria disease when primary immunization starts at 6-12 weeks, or 5-17 months of age

- To evaluate the protective efficacy of RTS,S/AS01E against clinical malaria disease caused by *Plasmodium falciparum* in African children whose age at first dose will be from 6-12 weeks and will receive vaccine in co-administration with DTPwHepB/Hib antigens (Tritanrix HepB/Hib) and OPV. Duration of follow up will be for a minimum of 12 months and a maximum of 18 months after completion of the primary course (Primary Analysis).
- To evaluate the protective efficacy of RTS,S/AS01E against clinical malaria disease caused by *Plasmodium falciparum* in African children whose age at first dose will be from 5-17 months. Duration of follow up will be for a minimum of 12 months and a maximum of 18 months after completion of the primary course (Primary Analysis).

Objectives **Secondary**

Efficacy against severe malaria disease

- To evaluate the protective efficacy of RTS,S/AS01E on a primary schedule with and without booster dose against severe malaria disease. Duration of follow up will extend to 30 months after completion of primary course.

Efficacy against incident severe anemia and malaria hospitalization

- To evaluate the protective efficacy of RTS,S/AS01E on a primary schedule with and without booster dose against incident severe anemia and malaria hospitalization. Duration of follow up will extend to 30 months after completion of primary course.

Efficacy; duration of efficacy of a primary course

- To evaluate the duration of protective efficacy of a primary schedule of RTS,S/AS01E with no booster dose against clinical malaria disease. Duration of follow up will extend to 30 months after completion of primary course.

Efficacy; the role of a booster

- To evaluate the protective efficacy of RTS,S/AS01E against clinical malaria disease with and without a booster dose given 18 months after completion of a primary schedule. Duration of follow up will be for 12 months after a booster dose.

Efficacy under different transmission settings

- To evaluate the protective efficacy of RTS,S/AS01E on a primary schedule with and without booster dose against clinical malaria disease under different conditions of malaria transmission. Field site will be used as a proxy for transmission setting. Duration of follow up will extend to 30 months after completion of primary course.

Efficacy against secondary case definitions of clinical malaria disease

- To evaluate the protective efficacy of RTS,S/AS01E on a primary schedule with and without booster dose against secondary case definitions of clinical malaria disease. Duration of follow up will extend to 30 months after completion of primary course.

Efficacy against prevalence of parasitemia

- To evaluate the protective efficacy of RTS,S/AS01E against prevalence of parasitemia 18 and 30 months after a primary schedule and 12 months after a booster dose.

Efficacy against prevalence of moderate and severe anemia

- To evaluate the protective efficacy of RTS,S/AS01E against prevalence of moderate and severe anemia 18 and 30 months after a primary schedule and 12 months after a booster dose.

Safety of a primary course

- For each age category, to evaluate the safety of a primary schedule of RTS,S/AS01E in African children. Duration of follow up will be for 14 months after receipt of the first dose (Primary Analysis).
- For each age category, to evaluate the safety of a primary schedule of RTS,S/AS01E without a boost. Duration of

follow up will be for 32 months after receipt of first dose.

Safety of a booster dose

- For each age category, to evaluate the safety of a booster dose of RTS,S/AS01E administered 18 months after completion of primary course. Duration of follow up will be for 12 months after receipt of boost.

Immunogenicity of a primary course

- For children aged 5-17 months at first vaccination, to evaluate the anti-CS and anti-HBs immunogenicity of a primary schedule of RTS,S/AS01E without a boost. Duration of follow up will be for 32 months after receipt of first dose.
- For children aged 6-12 weeks at first vaccination, to evaluate the anti-CS and anti-HBs immunogenicity of a primary schedule of RTS,S/AS01E without a boost when coadministered with DTPwHepB/Hib (Tritanrix HepB/Hib). Duration of follow up will be for 32 months after receipt of first dose.

Immunogenicity of a booster dose

- For children aged 5-17 months at first vaccination, to evaluate the anti-CS and anti-HBs immunogenicity of a booster dose of RTS,S/AS01E administered 18 months after completion of primary course. Duration of follow up will be for 12 months after receipt of boost.
- For children aged 6-12 weeks at first vaccination, to evaluate the anti-CS and anti-HBs immunogenicity of a booster dose of RTS,S/AS01E administered 18 months after completion of primary course of RTS,S/AS01E coadministered with DTPwHepB/Hib (Tritanrix HepB/Hib). Duration of follow up will be for 12 months after receipt of boost.

Immunogenicity of polio serotypes 1, 2 and 3

- To describe antibody responses to polio serotypes 1, 2 and 3 when OPV is given on a primary schedule co-administered with DTPwHepB/Hib (Tritanrix HepB/Hib) with or without RTS,S/AS01E at one month post dose 3.
- To describe antibody responses to polio serotypes 1, 2 and 3 when OPV is given on a primary schedule (co-administered with DTPwHepB/Hib with or without RTS,S/AS01E) and a booster dose at 18 months post primary (with or without RTS,S/AS01E) at one month post boost.

Efficacy against other serious illness

- To evaluate the protective efficacy of RTS,S/AS01E against other serious illness when given on a primary schedule with or without boost. Other serious illness is all-cause hospitalization, sepsis and pneumonia. Duration of follow-up will extend to 30 months after completion of primary course.

Efficacy against fatal malaria and all-cause mortality

- To evaluate the protective efficacy of RTS,S/AS01E against fatal malaria and all-cause mortality when given on a primary schedule with or without boost. Duration of follow up will extend to 30 months after completion of primary course.

Effect on growth

- To evaluate the effect of RTS,S/AS01E when given on a primary schedule with and without a booster dose on growth.

Gender-specific efficacy

- To evaluate the vaccine efficacy of RTS,S/AS01E in male and female children when given on a primary schedule with and without a boost.

Immunological correlates of protection

- To evaluate the association between CS-antibody response and protection against malaria disease.

Safety and immunogenicity in 'special' subgroups

- To evaluate safety and immunogenicity of a primary course or a booster dose of RTS,S/AS01E in low weight for age (i.e. z-score ≤ -2) and very low weight for age (i.e. z-score ≤ -3) children.
- To evaluate safety and immunogenicity of a primary course or a booster dose of RTS,S/AS01E in HIV-infected children.
  - *Note: HIV testing is not a trial procedure. This analysis will include all children known to be HIV-infected at enrollment or subsequently diagnosed.*

Objectives of the extension

Secondary

Efficacy

In children from 6 weeks to 17 months of age at primary immunisation for the period 14 days post Dose 3 to the end of extension:

- For each of the RTS,S/AS01E immunisation regimens primary alone or primary plus boost, to evaluate the

protective efficacy against clinical malaria disease caused by *Plasmodium falciparum* in each of at least 3 transmission settings.

- For each of the RTS,S/AS01E immunisation regimens primary alone or primary plus boost, to evaluate the total cases of clinical malaria disease caused by *Plasmodium falciparum* averted in each of at least 3 transmission settings.
- To evaluate the protective efficacy of RTS,S/AS01E (pooled immunisation regimens) against severe malaria disease.
- To evaluate the total cases of severe malaria disease averted by RTS,S/AS01E (pooled immunisation regimens).
- To evaluate the protective efficacy of RTS,S/AS01E (pooled immunisation regimens) against malaria hospitalization.
- To evaluate the total cases of malaria hospitalization averted by RTS,S/AS01E (pooled immunisation regimens).

In children from 6 weeks to 17 months of age at primary immunisation, at annual timepoints during the extension:

- To evaluate the protective efficacy of RTS,S/AS01E against prevalence of parasitemia.
- To evaluate the protective efficacy of RTS,S/AS01E against prevalence of moderate and severe anemia.

In children from 6 weeks to 17 months of age at primary immunisation for the period 14 days post Dose 3 to the end of extension:

- To evaluate the protective efficacy of RTS,S/AS01E (pooled immunisation regimens) against all-cause mortality and fatal malaria.
- To evaluate the total cases of all-cause mortality and fatal malaria averted by RTS,S/AS01E (pooled immunisation regimens).
- To evaluate the protective efficacy of RTS,S/AS01E (pooled immunisation regimens) against all medical hospitalization.
- To evaluate the total cases of all medical hospitalization averted by RTS,S/AS01E (pooled immunisation regimens).
- To evaluate the protective efficacy of RTS,S/AS01E (pooled immunisation regimens) against pneumonia.
- To evaluate the total cases of pneumonia averted by RTS,S/AS01E (pooled immunisation regimens).
- To evaluate the protective efficacy of RTS,S/AS01E (pooled

immunisation regimens) against sepsis.

- To evaluate the total cases of sepsis averted by RTS,S/AS01E (pooled immunisation regimens).

Safety

In children from 6 weeks to 17 months of age at primary immunisation for the time period from study start to the end of extension:

- To evaluate the safety of the RTS,S/AS01E vaccine.

Immunogenicity

- To analyze the anti-CS antibodies titers at annual timepoints during the extension.

Effect on growth

In children from 6 weeks to 17 months of age at primary immunisation:

- To evaluate the effect of RTS,S/AS01E on growth at the end of extension.

Study design

- Experimental design: Phase III, multi center, double blind (observer-blind), randomized, controlled trial with three groups in each of two age categories.
- Males and females enrolled in two age categories: children aged 6-12 weeks and children aged 5-17 months. Children must be > 28 days of age at screening. Those determined to be eligible based on the inclusion and exclusion criteria will be enrolled in the study.
- A maximum of 16 000 children (a *minimum* of 6000 in each age category) will be randomized.
- Treatment Groups and study vaccination schedule **children 5-17 months of age**:

Primary Vaccination on a 0, 1, 2 month schedule	Boost at M 20
RTS,S/AS01E	RTS,S/AS01E
RTS,S/AS01E	MCC Vaccine*
Rabies Vaccine** †	MCC Vaccine*

*Meningococcal C Conjugate vaccine: Meningitec (Wyeth), NeisVac-C (Baxter) or Menjugate (Novartis)
**Cell culture rabies vaccine: Human Diploid Cell Rabies Vaccine (Sanofi Pasteur) or Purified Vero cell culture rabies vaccine (VeroRab) (Sanofi Pasteur) or Purified Chick Embryo Cell Culture Vaccine (Rabipur or equivalent) (Novartis).
† each child will receive all 3 doses of rabies vaccine from the same manufacturer

- Treatment Groups and vaccination schedule
  children 6-12 weeks of age:

Primary Vaccination on a 0, 1, 2 month schedule	Boost at M 20
RTS,S/AS01E + Tritanrix HepB/Hib** + OPV	RTS,S/AS01E + OPV
RTS,S/AS01E + Tritanrix HepB/Hib** + OPV	MCC vaccine* + OPV
MCC vaccine * † + Tritanrix HepB/Hib** + OPV	MCC vaccine* + OPV

*Meningococcal C Conjugate vaccine: Meningitec (Wyeth), NeisVac-C (Baxter) or Menjugate (Novartis)
OPV: Oral Polio Vaccine (Polio Sabin [GSK])
† each child will receive all 3 doses of MCC from the same manufacturer.
** DTPwHepB/Hib

- **Route of administration:**
  - Children 5-17 months of age at enrollment will receive primary immunizations intramuscularly into the left deltoid.
  - Children 6-12 weeks of age at enrollment will receive primary immunizations intramuscularly into the anterolateral thigh
    (Left: RTS,S/AS01E and Meningococcal C conjugate vaccine; Right: Tritanrix HepB/Hib).
    NOTE: OPV will be administered orally.
  - Route of administration for the booster dose comparator will be into the left deltoid for ALL children.
- BCG vaccine, neonatal dose of OPV, measles and yellow fever vaccines will be given according to local EPI policy.
- There will be no routine testing for HIV in this trial. Tests will be performed when clinically indicated to guide subject management. Voluntary counseling and testing, highly active anti-retroviral therapy (HAART) and prevention of mother to child transmission (PMCT) are available at all study centers according to national policies.
- The research teams at each study center will ensure that insecticide treated bednet use is optimized in each study population.
- **Surveillance for Efficacy:**
  - All subjects presenting to health facilities in the study area will be evaluated as potential cases of clinical malaria disease. A blood sample will be taken for evaluation of malaria parasites in all children who are reported to have had a fever within 24 hours of presentation or have a measured axillary temperature of ≥ 37.5°C.

- All subjects presenting for admission through the outpatient and emergency departments of hospitals in the study areas will be evaluated as potential cases of severe malaria disease following a protocol-defined algorithm.
- During any hospitalization, the subject's course will be monitored to capture the signs and blood parameters indicative of severe malaria disease. If the subject's condition changes from admission and he/she meets one of the criteria for additional investigation, these will be performed.
- Harmonization of case evaluation across centers will be assured by training of clinicians in the assessment of clinical signs and the standardization of equipment and processes used for laboratory investigations.

- **Surveillance for Safety:**

*(*n *assumes 6000 enrolled, of which 90% will complete primary immunization and 85% complete booster dose and 10 participating sites)*

- SAEs will be collected for the duration of the study from all subjects
(*to have a minimum of 3620 recipients of a primary course of RTS,S/AS01E and 1680 recipients of a booster dose in each age category*).
- Unsolicited AEs (defined as those AEs judged to be related to vaccination, or leading to drop-out) will be collected for 1 month post each dose in all subjects
(*to have a minimum of 3620 recipients of a primary course of RTS,S/AS01E and 1680 recipients of a booster dose in each age category*).
- Unsolicited AEs (all) will be collected for 1 month following each vaccine dose for the first 200 subjects in each age category enrolled at each center
(to have approximately 1200 recipients of a primary course of RTS,S/AS01E and 560 recipients of a booster dose in each age category).
- Solicited AEs will be collected for one week following each vaccine dose in the first 200 subjects in each age category enrolled at each center
(*to have approximately 1200 recipients of a primary course of RTS,S/AS01E and 560 recipients of a booster dose in each age category*).
- Verbal autopsy will be performed on all cases of mortality occurring outside hospital.

- **Surveillance for Immunogenicity:**

(n *assumes 6000 enrolled, of which 90% will complete primary immunization and 85% complete booster dose and 10 participating sites)*

  - All children will be sampled for anti-HBs and anti-CS testing.
  - Immunogenicity of a primary course will be evaluated pre vaccination, 1 month post primary course and 18 months post primary course in the first 200 subjects in each age category enrolled at each center (*to have approximately 1200 recipients of a primary course of RTS,S/AS01E in each age category*).
  - Duration of immunogenicity of a primary course will be evaluated 18 and 30 months post primary course in the first 200 subjects in each age category enrolled at each center (*to have approximately 560 recipients of a primary course of RTS,S/AS01E in each age category*).
  - Immunogenicity of a booster dose will be evaluated pre-boost vaccination, 1 month post boost and 12 months post boost in the first 200 subjects in each age category enrolled at each center (*to have approximately 560 recipients of a primary course of RTS,S/AS01E in each age category*).
  - Immune correlates of protection will be explored by sampling all subjects 1 month post primary course and 1 month post boost and the full details of the analysis will be presented in the SAP to compare subjects affected and unaffected by malaria disease.
  - Surveillance for Cellular Immunology: At some study centers, subjects may be sampled for evaluation of the cellular immune response induced by the RTS,S/AS01E vaccine. CMI procedures will be detailed in separate protocol, ancillary to this study.
  - Immunogenicity of the polio serotypes 1, 2 and 3 will be evaluated when OPV is given on a primary schedule with DTPwHepB/Hib (Tritanrix HepB/Hib) with or without RTS,S/AS01E at screening, at 1 month post primary course (Visit 5) and following a booster dose at 18 months post primary (with or without RTS,S/AS01E) at 1 month post boost (Visit 23) in a subset of subjects in the 6-12 weeks age category enrolled at each center.

- Data collection: where technically possible, this study will use electronic case report form (eCRF) by remote data entry

(RDE) in preference to conventional paper case report form (CRF).

- Subjects will be randomized using an internet-based system.
- Duration of the study: the primary study will continue for 32 months per child, plus a screening period of 1 month.
- Analysis of co-primary endpoint: clinical disease
  - The analysis of efficacy of a primary schedule of RTS,S/AS01E will be carried out after the first 14 months of follow-up have been completed for the first 6000 subjects (approximately) enrolled in the age category under evaluation.
  - It is anticipated that enrolment of the 5-17 months age category will be faster than in the infant age category and thus analysis of the two co-primary efficacy endpoints may take place at different times. In order to control the overall alpha-level (5%) of the trial both evaluations will be performed at a 2.5% alpha level (Bonferroni correction) thus, for the evaluation of the co-primary endpoint leading to 97.5% confidence intervals (CI).
  - In both age categories, efficacy against clinical malaria of the primary immunization schedule will be measured when at least 6000 subjects have 14 months of follow-up. Assuming at least 5400 evaluable subjects (randomized 2:1), an attack rate in controls of 10/100 cyr (child years at risk) over the follow-up period and a true vaccine efficacy of 30%, the study has 90% power to detect a lower limit of the 97.5% CI around estimated VE above 0%.
  - In the event that the attack rate is lower than anticipated and there have not been 450 accumulated cases in the observation period 14 days to 12 months post Dose 3, then the period of observation contributing to the primary analysis will be extended. The analysis will be conducted when 450 cases have accumulated, or until boost (visit 22) (approximately 18 months post dose 3) whichever occurs earlier.
- Analysis of secondary endpoint: severe disease
  - A secondary analysis of severe malaria will be performed when 250 episodes of severe malaria meeting the primary case definition, pooled over the study centers and age categories, have occurred, or at the end of the follow-up period (Month 32), whichever occurs first.

- 250 episodes give 80% power to detect 30% VE with a lower limit of the 95% CI above 0%. Assuming 50% VE 250 episodes give 90% power to detect a lower limit of the 95% CI above 25%.

- Analysis of secondary endpoint: antibodies to polio serotypes 1, 2 and 3
  - A secondary analysis of the immune response for polio serotypes 1, 2 and 3 when OPV is given on a primary schedule co-administered with DTPwHepB/Hib (Tritanrix HepB/Hib) with or without RTS,S/AS01E at screening, at 1 month post primary course (Visit 5) and following a booster dose at 18 months post primary (with or without RTS,S/AS01E) at one month post boost.
- The first 200 subjects enrolled at each center, in each age category, will form a subset for additional safety and immunology endpoints. Safety evaluations comprise the collection of solicited reactogenicity and the use of antipyretic, analgesic and systemic antibiotics. Immunogenicity evaluations comprise responses to anti-CS and anti-HBs antigens and responses to OPV only in the 6-12 weeks age category.

Study design of the extension

- Experimental design: Extension to the Phase III, multi-center, randomized, controlled trial Malaria-055
- Blinding:
  - Initial study (D0 to Month 32): Double-Blind
  - Extension: Single blind
- Study groups: 3 groups in children 6 weeks to 17 months at first vaccination.
  - Primary schedule + booster R3R
  - Primary schedule + control vaccine R3C
  - Comparator group C3C
- Number of subjects: A maximum of 16 000 children are planned to be enrolled in the initial study. All subjects enrolled in the primary trial phase for whom parents/guardians will give their consent for participation in the extension, who received at least one dose of study or control vaccine in the primary trial phase and whose Visit 35 took place before (and including) 30 September 2013, will be enrolled.
- Malaria control: Bednet usage and indoor residual spraying will be documented for each subject before the last extension

visit using the same methodology of the primary trial phase.

- Sampling timepoints: Blood samples will be taken at annual intervals during the extension (maximum 2 blood samples).
- Data collection: This study will use electronic case report form (eCRF) by remote data entry (RDE).
- Interval between study visits: Depending on the date of the last visit in the primary trial phase (Visit 34), subjects will have up to 3 clinic visits plus one field workers visit during this extension (Refer to Figure 2). If a subject does not attend Visit 34, the schedule of the following visit(s) will be based on the assumption that the Visit 34 occurred 32 months post Dose 1.
    - Subjects that have their last visit in the primary trial phase (Visit 34) BEFORE (and including) 30 June 2012 will have 3 clinic visits plus one field workers visit in the extension.
    - Subjects that have their last visit in the primary trial phase (Visit 34) between 1 July 2012 and 30 September 2013 will have 2 clinic visits plus one field workers visit in the extension.
    - No subjects will be enrolled in the extension study (Visit 35) after 30 September 2013.

Synopsis table 1 Intervals between study visits for subjects that have their last visit in the primary trial phase (Visit 34) <u>BEFORE (and including) 30 Jun 2012</u>

Interval	Optimal time for study visit (Study Months)	~~Maximum interval allowed~~
**Visit 35**	On same day as Visit 34* ~~(Study Month 32)~~	***Maximum interval:*** Visit 2 + 44 months
**Visit 34→Visit 36**	12 months** ~~(Study Month 44)~~	***Range:*** 11 to 13 months
**Visit 34→Visit 37§ (Field workers visit)**	***Nov 2013*** ~~23 months**~~ ~~(Study Month 55)~~	***Date:*** Oct 2013 to Dec 2013
**Visit 34→Visit 38**	***Dec 2013*** ~~24 months**~~ ~~(Study Month 56)~~	***Date:*** Nov 2013 to Jan 2014

* If subjects are not present at Visit 34, Visit 35 should be scheduled as soon as possible and up to a maximum of 12 months after Visit 34, assuming Visit 34 should have occurred at Visit 2 + 32 months

** If subjects are not present at Visit 34, Visit 36, 37 and 38 should be scheduled assuming Visit 34 should have occurred at Visit 2 + 32 months

§Visit 37 (Field workers visit) has to be conducted within one month before Visit 38

Amended 08 August 2012

Synopsis table 2 Intervals between study visits for subjects that have their last visit in the primary trial phase (Visit 34) after 30 Jun 2012 and BEFORE (and including) 30 Sep 2013

Interval	Optimal time for study visit (Study Months)	~~Maximum interval allowed~~
**Visit 35**	On same day as Visit 34* ~~(Study Month 32)~~	***Maximum interval:*** Visit 2 + 44 months or 30 Sep 2013 at the latest
**Visit 34→Visit 37§ (Field workers visit)**	***Nov 2013*** ~~11 months**~~ ~~(Study Month 43)~~	***Date:*** Oct 2013 to Dec 2013
**Visit 34→Visit 38**	***Dec 2013*** ~~12 months**~~ ~~(Study Month 44)~~	***Date:*** Nov 2013 to Jan 2014

* If subjects are not present at Visit 34, Visit 35 should be scheduled as soon as possible and up to a maximum of 12 months after Visit 34, assuming Visit 34 should have occurred at Visit 2 + 32 months. No subjects will be enrolled for Visit 35 after 30 September 2013.
** If subjects are not present at Visit 34, Visit 37 and 38 should be scheduled assuming Visit 34 should have occurred at Visit 2 + 32 months
§Visit 37 (Field workers visit) has to be conducted between 1 and 30 days before Visit 38.

Amended 08 August 2012

- Informed consent: Freely given informed consent should be obtained from subjects' parent(s)/LAR(s) prior to participation in the extension. The informed consent must be signed at the first visit of the extension, Visit 35. To avoid any gap in data collection between the initial study and the extension, Visit 35 should ideally take place on the same day as Visit 34. If subjects are not present at Visit 34, Visit 35 and signature of informed consent should be scheduled as soon as possible and up to a maximum 12 months after Visit 34 should have occurred.
- **Surveillance for Efficacy during the extension:**
  - Surveillance for severe malaria disease will be maintained at all inpatient facilities in the study areas. Children will be evaluated according to an algorithm to accurately define cases.
  - Surveillance for clinical malaria disease will be maintained at the outpatient facilities in the study areas of at least 3 participating centers of varying malaria transmission intensity.
  - Parasite prevalence and haemoglobin will be assessed annually on all subjects.
- **Surveillance for Immunogenicity during the extension:**
  - Anti-CS antibodies will be assessed in a subset of 200 subjects in each age category in at least three centers

evaluating efficacy to clinical disease.

- **Surveillance for Safety during the extension:**
  - Surveillance to detect SAEs will be maintained at all inpatient facilities in the study areas.
  - Verbal autopsy will be performed on all cases of mortality occurring outside hospital.

Case Definitions **Clinical malaria**

**1° definition**	*P. falciparum asexual parasitemia > 5000 parasites/µL*<br>***AND** presence of fever (axillary temperature ≥ 37.5°C) at the time of presentation*<br>***AND** occurring in a child who is unwell and brought for treatment to a healthcare facility*<br>***OR***<br>*a case of malaria meeting the primary case definition of severe malaria disease* (refer to Table 6)
**2° definition 1**	*P. falciparum asexual parasitemia > 0*<br>***AND** presence of fever (axillary temperature ≥ 37.5°C) at the time of presentation or history of fever within 24 hours of presentation*<br>***AND** occurring in a child who is unwell and brought for treatment to a healthcare facility*
**2° definition 2**	*P. falciparum* asexual parasitemia > 500 parasites/µL<br>**AND** presence of fever (axillary temperature ≥ 37.5°C) at the time of presentation<br>**AND** occurring in a child who is unwell and brought for treatment to a healthcare facility
**2° definition 3**	*P. falciparum asexual parasitemia > 20 000 parasites/µL*<br>***AND** presence of fever (axillary temperature ≥ 37.5°C) at the time of presentation*<br>***AND** occurring in a child who is unwell and brought for treatment to a healthcare facility*

Case Definitions **Primary case definition of severe malaria disease**

P. falciparum > 5000 parasites per µL	
**AND** with one or more marker of disease severity	• Prostration<br>• Respiratory distress<br>• Blantyre score ≤ 2<br>• Seizures 2 or more<br>• Hypoglycemia < 2.2 mmol/L<br>• Acidosis BE ≤-10.0 mmol/L<br>• Lactate ≥ 5.0 mmol/L<br>• Anemia < 5.0 g/dL
**AND** without diagnosis of a co-morbidity	• Radiographically proven pneumonia<br>• Meningitis on CSF examination<br>• Positive blood culture<br>• Gastroenteritis with dehydration

FOOTNOTES

Prostration is defined as, in an acutely sick child, the inability to perform previously-acquired motor function: in a child previously able to stand, inability to stand; in a child previously able to sit, inability to sit and in a very young child, inability to suck.

Respiratory distress is defined as lower chest wall indrawing or abnormally deep breathing.

2 or more seizures occurring in the total time period including 24 hours prior to admission, the emergency

room and the hospitalization.
Radiographically proven pneumonia is a consolidation or pleural effusion as defined in Appendix G on a chest x-ray taken within 72 h of admission.
Meningitis on CSF examination is defined as WC $\geq$ 50 x10 ^{6}/L or positive culture of compatible organism or latex agglutination positive for Hib, pneumococci or meningococci [Berkley, 2001].
Gastroenteritis with dehydration is defined as a history of 3 or more loose or watery stools in previous 24 h and an observed watery stool with decreased skin turgor (> 2 seconds for skin to return following skin pinch).
Positive blood culture is defined in Appendix F on a blood culture taken within 72 h of admission.

Case Definitions Secondary case definitions of severe malaria disease

**2° definition 1**<br>"with co-morbidity"	*P. falciparum* > 5000 parasites per µL<br>**AND** with one or more marker of disease severity
**2° definition 2**<br>"without a density threshold"	*P. falciparum* > 0<br>**AND** with one or more marker of disease severity<br>**AND** without diagnosis of a co-morbidity
**2° definition 3**<br>"without HIV"	*P. falciparum* > 5000 parasites per µL<br>**AND** with one or more marker of disease severity<br>**AND** without diagnosis of a co-morbidity<br>**AND** without a confirmed diagnosis of HIV infection

Case Definitions Malaria hospitalization

**Definition 1**	A medical hospitalization with confirmed *P. falciparum* > 5000 parasites/µL<br>(excludes planned admissions for medical investigation/care or elective surgery and trauma)
**Definition 2**	A hospitalization which, in the judgment of the principal investigator, *P. falciparum* infection was the sole or a major contributing factor to the presentation

Case Definitions Fatal malaria

**1° definition**	A case of severe malaria meeting the primary case definition of severe malaria disease (see *primary case definition of severe malaria disease* [above]) with a fatal outcome<br>(i.e. is restricted to hospital mortality and does not include cause ascribed by verbal autopsy).
**2° definition 1**<br>"with co-morbidity"	A case of severe malaria meeting the secondary case definition 1 severe malaria disease (see *secondary case definitions of severe malaria disease* [above]) with a fatal outcome
**2° definition 2**<br>"without a density threshold"	A case of severe malaria meeting the secondary case definition 2 severe malaria disease (see *secondary case definitions of severe malaria disease* [above]) with a fatal outcome
**2° definition 3**<br>"without HIV"	A case of severe malaria meeting the secondary case definition 3 severe malaria disease (see *secondary case definitions of severe malaria disease* [above]) with a fatal outcome

Case Definitions Incident severe anemia

**Definition 1**	A documented hemoglobin < 5.0 g/dL identified at clinical presentation to morbidity surveillance system in association with a *P. falciparum* parasitemia > 5000 parasites/µL
**Definition 2**	A documented hemoglobin < 5.0 g/dL identified at clinical presentation to morbidity surveillance system in association with a *P. falciparum* parasitemia > 0 parasites/µL
**Definition 3**	A documented hemoglobin < 5.0 g/dL identified at clinical presentation to morbidity surveillance system

Case Definitions Prevalent anemia

**Prevalent severe anemia**	A documented hemoglobin < 5.0 g/dL identified at a cross sectional survey
**Prevalent moderate anemia**	A documented hemoglobin < 8.0 g/dL identified at a cross sectional survey

Case Definitions All medical hospitalization

**1° definition**	A medical hospitalization of any cause<br>(*excludes* planned admissions for medical investigation/care or elective surgery and trauma)

Case Definitions All-cause mortality

**Definition 1**	A fatality (of any cause)<br>(*includes* mortality in the community and in hospital)
**Definition 2**	A fatality (medical cause)<br>(*includes* mortality in the community and in hospital)<br>(*excludes* trauma, which may be diagnosed by verbal autopsy)

Case Definitions Pneumonia

**1° definition**	cough or difficulty breathing (on history)<br>**AND** tachypnea<br>**AND** lower chest wall indrawing
**2° definition 1**	1° case definition of pneumonia<br>**AND** CXR consolidation or pleural effusion (as defined in Appendix G on a chest x-ray taken within 72 h of admission)
**2° definition 2**	1° case definition of pneumonia<br>**AND** CXR consolidation or pleural effusion or other infiltrates (as defined in Appendix G on a chest x-ray taken within 72 h of admission)
**2° definition 3**	1° case definition of pneumonia<br>**AND** an oxygen saturation less than 90%

FOOTNOTES
Tachypnea (≥ 50 breaths per minute < 1 year, ≥ 40 breaths per minute ≥ 1year).

Case Definitions **Sepsis**

**1° definition**	A child with positive blood culture

FOOTNOTES
Positive blood culture as defined in Appendix H on a blood culture taken within 72 h of admission.

Endpoints **Primary**

Efficacy against clinical malaria disease when primary immunization starts at 6-12 weeks, or 5-17 months of age

- For a primary schedule, the occurrence of cases of malaria meeting the primary case definition for clinical malaria disease over a period starting 14 days post Dose 3 (Visit 4 +14 days) for 12 months in children aged 6-12 weeks at the time of Dose 1, or until the time at which 450 subjects experience a case, whichever occurs later. If 450 are not accumulated by the time of boost (Visit 22), the analysis will proceed.
- For a primary schedule, the occurrence of cases of malaria meeting the primary case definition for clinical malaria disease over a period starting 14 days post Dose 3 (Visit 4 +14 days) for 12 months in children aged 5-17 months at the time of Dose 1, or until the time at which 450 subjects experience a case, whichever occurs later. If 450 are not accumulated by the time of boost (Visit 22), the analysis will proceed.

Endpoints **Secondary**

Efficacy against severe malaria disease

- For a primary schedule (pooled with and without a boost), the occurrence of severe malaria disease meeting the primary and secondary case definitions up to the time at which 250 subjects are diagnosed with severe malaria meeting the primary case definition.
- For a primary schedule with and without a boost, the occurrence of severe malaria disease meeting the primary and secondary case definitions analyzed over the time periods starting 14 days post Dose 3 (Visit 4 +14 days) until boost (Visit 22), boost (Visit 22) until study end (Visit 34) and 14 days post Dose 3 (Visit 4 +14 days) until study end (Visit 34).

Efficacy against incident severe anemia and malaria hospitalization

- For a primary schedule with and without a boost, the occurrence of incident severe anemia and malaria

hospitalization meeting the primary and secondary case definitions analyzed over the time periods starting 14 days post Dose 3 (Visit 4 +14 days) until boost (Visit 22), boost (Visit 22) until study end (Visit 34) and 14 days post Dose 3 (Visit 4 +14 days) until study end (Visit 34).

Efficacy; duration of efficacy of a primary course

- For a primary schedule without a boost, the occurrence of clinical malaria disease meeting the primary case definition analyzed over the time periods starting 14 days post Dose 3 (Visit 4 +14 days) until boost (Visit 22), boost (Visit 22) until study end (Visit 34) and 14 days post Dose 3 (Visit 4 +14 days) until study end (Visit 34).

Efficacy; role of a booster

- For a primary schedule with and without a boost, the occurrence of clinical malaria disease meeting the primary case definition analyzed over the time period starting at boost (Visit 22) until study end (Visit 34).

Efficacy under different transmission settings

- For each site, for a primary schedule with and without a boost, the occurrence of clinical malaria disease meeting the primary case definition analyzed over the time periods starting 14 days post Dose 3 (Visit 4 +14 days) until boost (Visit 22), boost (Visit 22) until study end (Visit 34) and 14 days post Dose 3 (Visit 4 +14 days) until study end (Visit 34).

Efficacy against secondary case definitions of clinical malaria disease

- For a primary schedule with and without a boost, the occurrence of clinical malaria disease meeting the secondary case definitions analyzed over the time periods starting 14 days post Dose 3 (Visit 4 +14 days) until boost (Visit 22), boost (Visit 22) until study end (Visit 34) and 14 days post Dose 3 (Visit 4 +14 days) until study end (Visit 34).

Efficacy against prevalence of parasitemia

- For a primary schedule without a boost, the presence of parasitemia at 18 months (Visit 22) and 30 months (Visit 34) after a primary schedule.
- For a booster schedule, the presence of parasitemia 12 months after a boost (Visit 34).

Efficacy against prevalence of moderate and severe anemia

- For a primary schedule without a boost, the presence of moderate and severe anemia at 18 months (Visit 22) and 30 months (Visit 34) after a primary schedule.
- For a booster schedule, the presence of moderate and severe anemia 12 months after a boost (Visit 34).

Safety of a primary course

- For each age category, for a primary schedule, the occurrence of SAEs from Dose 1 (Visit 2) until 14 months post Dose 1 (Visit 16).
- For each age category, for a primary schedule, the occurrence of SAEs over a 30-day follow-up period (day of vaccination and 29 subsequent days) after each vaccination.
- For each age category, for a primary schedule without boost, the occurrence of SAEs analyzed over the time periods starting at Dose 1 (Visit 2) until boost (Visit 22), boost (Visit 22) until study end (Visit 34) and Dose 1 (Visit 2) until study end (Visit 34).
- For each age category, for a primary schedule, the occurrence of solicited symptoms over a 7-day follow-up period (day of vaccination and 6 subsequent days) after each vaccination.
- For each age category, for a primary schedule, the occurrence of all unsolicited symptoms over a 30-day follow-up period (day of vaccination and 29 subsequent days) after each vaccination.
- For each age category, for a primary schedule, the occurrence of unsolicited symptoms related to vaccination or leading to withdrawal over a 30-day follow-up period (day of vaccination and 29 subsequent days) after each vaccination.

Safety of a booster dose

- For each age category, for a booster dose, the occurrence of SAEs analyzed over the time period starting at boost (Visit 22) until study end (Visit 34).
- For each age category, for a booster dose, the occurrence of SAEs over a 30-day follow-up period (day of vaccination and 29 subsequent days) after boost.
- For each age category, for a booster dose, the occurrence of solicited symptoms over a 7-day follow-up period (day of vaccination and 6 subsequent days) after the boost.
- For each age category, for a booster dose, the occurrence of

all unsolicited symptoms over a 30-day follow-up period (day of vaccination and 29 subsequent days) after each vaccination.

- For each age category, for a booster dose, the occurrence of unsolicited symptoms over a 30-day follow-up period (day of vaccination and 29 subsequent days) after the boost.

Immunogenicity of a primary course

- For each age category, for a primary schedule without a boost, the anti-CS antibody titers at screening, 1 month post Dose 3 (Visit 5), 18 months post Dose 3 (Visit 22) and 30 months post Dose 3 (Visit 34).
- For each age category, for a primary schedule without a boost, the anti-HBs antibody titers at screening, 1 month post Dose 3 (Visit 5), 18 months post Dose 3 (Visit 22) and 30 months post Dose 3 (Visit 34).

Immunogenicity of a booster dose

- For each age category, for a booster schedule, the anti-CS antibody titers at boost (Visit 22), 1 month post boost (Visit 23), 12 months post boost (Visit 34).
- For each age category, for a booster schedule, the anti-HBs antibody titers at boost (Visit 22), 1 month post boost (Visit 23), 12 months post boost (Visit 34).

Immunogenicity of polio serotypes 1, 2 and 3

- To analyze in a subset of African children whose age at first dose will be 6-12 weeks the percentage of subjects with seroprotective levels of anti-polio 1, 2, 3 antibodies when OPV is given on a primary schedule co-administered with DTPwHepB/Hib (Tritanrix HepB/Hib) with or without RTS,S/AS01E at one month post dose 3 (Visit 5).
- To analyze in a subset of African children whose age at first dose will be 6-12 weeks the percentage of subjects with seroprotective levels of anti-polio 1, 2, 3 antibodies when OPV is given on a primary schedule (co-administered with DTPwHepB/Hib with or without RTS,S/AS01E) and a booster dose at 18 months post primary (with or without RTS,S/AS01E) at one month post boost (Visit 23).

Efficacy against other serious illness

- For a primary schedule with and without a boost, the occurrence of other serious illness meeting the primary and secondary case definitions analyzed over the time periods starting 14 days post Dose 3 (Visit 4 + 14 days) until boost

(Visit 22), boost (Visit 22) until study end (Visit 34) and 14 days post Dose 3 (Visit 4 + 14 days) until study end (Visit 34). Other serious illness is all medical hospitalization, sepsis and pneumonia.

Efficacy against fatal malaria and all-cause mortality

- For a primary schedule with and without a boost, the occurrence of fatal malaria (meeting the case definitions) and all-cause mortality analyzed over the time periods starting 14 days post Dose 3 (Visit 4 +14 days) until study end (Visit 34).

Effect on growth

- For a primary schedule with and without a boost, to compare the height/length for age z-score at study end (Visit 34).
- For a primary schedule with and without a boost, to compare the weight for age z-score at study end (Visit 34).
- For a primary schedule with and without a boost, to compare the mid upper arm circumference for age z-score at study end (Visit 34).
- For children 6-12 weeks at first vaccination, for a primary schedule with and without boost, to compare the absolute height at study end (Visit 34).

Gender-specific efficacy

- In male and female children, for a primary schedule with and without a boost the occurrence of clinical malaria disease meeting the primary case definition analyzed over the time periods starting 14 days post Dose 3 (Visit 4 +14 days) until boost (Visit 22), boost (Visit 22) until study end (Visit 34) and 14 days post Dose 3 (Visit 4 +14 days) until study end (Visit 34).

If important differences in efficacy are observed between male and female children, then all primary and secondary immunological and efficacy endpoints will be presented for male and female children.

Immunological correlates of protection

- In cases and non-cases of malaria disease, CS-antibody titers.

Safety and immunogenicity in 'special' subgroups

Note: HIV testing is not a trial procedure. This analysis will include all children known to be HIV-infected at enrollment or subsequently diagnosed

- In HIV-infected children, for each age category, for a primary schedule with and without a boost, the occurrence of SAEs analyzed over the time periods starting at Dose 1 (Visit 2) until boost (Visit 22), boost (Visit 22) until study end (Visit 34) and Dose 1 (Visit 2) until study end (Visit 34).
- In HIV-infected children, for each age category, for a primary schedule and a booster dose, the occurrence of unsolicited symptoms related to vaccination or leading to withdrawal over a 30-day follow-up period (day of vaccination and 29 subsequent days) after each vaccination.
- In HIV-infected children, for each age category, for a primary schedule with and without a boost, the anti-CS antibody titers at screening, 1 month post Dose 3 (Visit 5), 18 months post Dose 3 (Visit 22) and 30 months post Dose 3 (Visit 34).
- In HIV-infected children for each age category, for a primary schedule with and without a boost, the anti-HBs antibody titers at screening, 1 month post Dose 3 (Visit 5), 18 months post Dose 3 (Visit 22) and 30 months post Dose 3 (Visit 34).
- In low weight for age children (weight for age z-score ≤-2) and in very low weight for age children (weight for age z-score ≤-3), for each age category, for a primary schedule with and without a boost, the occurrence of SAEs analyzed over the time periods starting at Dose 1 (Visit 2) until boost (Visit 22), boost (Visit 22) until study end (Visit 34) and Dose 1 (Visit 2) until study end (Visit 34).
- In low weight for age children (weight for age z-score ≤-2) and in very low weight for age children (weight for age z-score ≤-3), for each age category, for a primary schedule and a boost, the occurrence of unsolicited symptoms related to vaccination or leading to withdrawal over a 30-day follow-up period (day of vaccination and 29 subsequent days) after each vaccination.
- In low weight for age children (weight for age z-score ≤-2) and in very low weight for age children (weight for age z-score ≤-3), for each age category, for a primary schedule with and without a boost, the anti-CS antibody titers at screening, 1 month post Dose 3 (Visit 5), 18 months post Dose 3 (Visit 22) and 30 months post Dose 3 (Visit 34).
- In low weight for age children (weight for age z-score ≤-2) and in very low weight for age children (weight for age z-score ≤-3), for each age category, for a primary schedule with and without a boost, the anti-HBs antibody titers at screening, 1 month post Dose 3 (Visit 5), 18 months post Dose 3 (Visit 22) and 30 months post Dose 3 (Visit 34).

Endpoints for the extension

Secondary

Efficacy

- In each of at least three study centers, the occurrence of clinical malaria disease meeting the primary and secondary case definitions for clinical malaria over a period starting 14 days post Dose 3 until the last visit of the extension.
- Pooled across at least three study centers, the occurrence of clinical malaria disease meeting the primary and secondary case definitions for clinical malaria over annual time periods.
- Pooled across all participating study centers, the occurrence of severe malaria disease meeting the primary and secondary case definitions for severe malaria over a period starting 14 days post Dose 3 until the last visit of the extension.
- Pooled across all participating study centers, the occurrence of malaria hospitalization meeting the primary and secondary case definitions for malaria hospitalization over a period starting 14 days post Dose 3 until the last visit of the extension.
- Pooled across all participating study centers, the presence of parasitemia at annual timepoints during the extension.
- Pooled across all participating study centers, the presence of moderate and severe anemia at annual timepoints during the extension.
- Pooled across all participating study centers, the occurrence of all-cause mortality and fatal malaria meeting the primary and secondary case definitions over a period starting 14 days post Dose 3 until the last visit of the extension.
- Pooled across all participating study centers, the occurrence of all-medical hospitalization meeting the primary case definition over a period starting 14 days post Dose 3 until the last visit of the extension.
- Pooled across all participating study centers, the occurrence of pneumonia meeting the primary and secondary case definitions over a period starting 14 days post Dose 3 until the last visit of the extension.
- Pooled across all participating study centers, the occurrence of sepsis meeting the primary case definition over a period starting 14 days post Dose 3 until the last visit of the extension).

Safety

- In all participating study centers, the occurrence of SAEs from Dose 1 (Day 0) until the end of extension.

Immunogenicity

- In a subset of subjects, the anti-CS antibody titers at annual timepoints during the extension.

Effect on growth

- In all subjects at all participating study centers, to compare the height for age z-score at the end of extension.

Relevant efficacy and immunogenicity endpoints will also be analyzed by annual time periods, age category and by site.

TABLE OF CONTENTS

PAGE

SYNOPSIS ... 7

LIST OF ABBREVIATIONS ... 44

GLOSSARY OF TERMS ... 48

1. INTRODUCTION ... 52
 1.1. Malaria ... 52
 1.2. Hepatitis ... 52
 1.3. RTS,S candidate vaccine ... 53
 1.4. Key clinical, efficacy and immunogenicity data ... 53
  1.4.1. The RTS,S/AS02A candidate malaria vaccine; key clinical efficacy, safety and immunogenicity data in children ... 54
  1.4.2. The RTS,S/AS02D candidate malaria vaccine; key clinical efficacy, safety and immunogenicity data in infants ... 57
  1.4.3. RTS,S/AS01 vaccine; key clinical efficacy, safety and immunogenicity data ... 58
 1.5. Rationale for the study ... 60
  1.5.1. Rationale for the choice of adjuvant ... 61
  1.5.2. Rationale for evaluation of booster ... 62
  1.5.3. Rationale for analysis by site ... 62
  1.5.4. Rationale for the primary case definition of clinical malaria disease ... 62
  1.5.5. Rationale for the primary case definition of severe malaria disease ... 63
  1.5.6. Rationale for the methodology of analysis of clinical malaria endpoint ... 65
  1.5.7. Rationale for sample size ... 65
  1.5.8. Rationale for the methodology of analysis of severe malaria endpoint ... 66
  1.5.9. Rationale for choice of cohort for primary analysis ... 66
  1.5.10. Choice of comparator vaccines ... 66
   1.5.10.1. Rationale for the use of Meningococcal C conjugate vaccine as a control ... 66
   1.5.10.2. Rationale for the use of rabies vaccine as a control ... 68
 1.6. Rationale for the extension ... 69

2. OBJECTIVES ... 70
 2.1. Primary objectives ... 70
  2.1.1. Efficacy coprimary objectives: Efficacy against clinical malaria disease when primary immunization starts at 6-12 weeks, or 5-17 months of age ... 70
 2.2. Secondary objectives ... 70
  2.2.1. Efficacy secondary objectives ... 70
   2.2.1.1. Efficacy against severe malaria disease ... 70
   2.2.1.2. Efficacy against incident severe anemia and malaria hospitalization ... 70

2.2.1.3. Efficacy; duration of efficacy of a primary course 70
2.2.1.4. Efficacy; the role of a booster 71
2.2.1.5. Efficacy under different transmission settings 71
2.2.1.6. Efficacy against secondary case definitions of clinical malaria disease 71
2.2.1.7. Efficacy against prevalence of parasitemia 71
2.2.1.8. Efficacy against prevalence of moderate and severe anemia 71
2.2.2. Safety secondary objectives 71
2.2.2.1. Safety of a primary course 71
2.2.2.2. Safety of a booster dose 72
2.2.3. Immunogenicity secondary objectives 72
2.2.3.1. Immunogenicity of a primary course 72
2.2.3.2. Immunogenicity of a booster dose 72
2.2.3.3. Antibodies to polio serotypes 1, 2 and 3 72
2.3. Additional Secondary Objectives 73
2.3.1. Additional efficacy secondary objectives 73
2.3.1.1. Efficacy against other serious illness 73
2.3.1.2. Efficacy against fatal malaria and all-cause mortality 73
2.3.1.3. Effect on growth 73
2.3.1.4. Gender-specific efficacy 73
2.3.2. Additional immunogenicity secondary objectives 73
2.3.2.1. Immunological correlates of protection 73
2.3.3. Additional safety and immunogenicity secondary objectives 73
2.3.3.1. Safety and immunogenicity in ‘special’ subgroups 73
2.4. Secondary objectives for the extension 74
2.4.1. Efficacy 74
2.4.2. Safety 75
2.4.3. Immunogenicity 75
2.4.4. Effect on growth 75

3. STUDY DESIGN OVERVIEW 76
3.1. Study design 78
3.2. Study design of the extension 82

4. STUDY COHORT 84
4.1. Number of subjects / centers 84
4.2. Inclusion criteria for the primary trial phase 84
4.2.1. Inclusion criteria for the extension 85
4.3. Exclusion criteria for enrolment for the primary trial phase 85
4.4. Elimination criteria during the study 86
4.5. Contraindications to subsequent vaccination 87
4.5.1. Indications for deferral of vaccination 87
4.5.2. Absolute contraindications to further vaccination 87

5. CONDUCT OF STUDY 88
5.1. Ethics and regulatory considerations 88

5.1.1. Institutional Review Board/Independent Ethics Committee (IRB/IEC) 88
5.1.2. Informed consent 89
5.1.3. Storage of study documentation at investigator's sites 92
5.1.4. Safety Monitoring Plan 92
5.1.4.1. Data Safety Monitoring Board (DSMB)/Independent Data Monitoring Committee (IDMC) 92
5.1.4.1.1. Data Reviewed by the DSMB 93
5.1.4.2. Local Safety Monitor (LSM) 93
5.1.4.2.1. Data Reviewed by the LSM 93
5.1.4.3. Process if the trial is suspended 94
5.1.4.4. Rescue plan for HBs 94
5.1.4.5. Monitoring of polio serotypes 1, 2 and 3 seroprotection 94
5.1.4.6. Completion of immunization course for infants withdrawn during vaccination course 95
5.2. Case definitions 95
5.2.1. Clinical malaria disease 95
5.2.2. Severe malaria disease 95
5.2.3. Malaria hospitalization 97
5.2.4. Fatal malaria 97
5.2.5. Severe anemia 98
5.2.6. All hospitalizations 98
5.2.7. All-cause mortality 98
5.2.8. Pneumonia 99
5.2.9. Sepsis 99
5.3. Efficacy endpoint capture 99
5.3.1. Surveillance for clinical malaria disease 99
5.3.2. Surveillance for severe malaria disease 100
5.3.3. Cross-sectional surveys 100
5.3.4. Cross-sectional survey in the extension 100
5.3.5. Verbal autopsy process 101
5.4. Subject identification 101
5.5. Outline of study procedures 102
5.5.1. Intervals between study visits for the extension 107
5.6. Detailed description of study stages/visits 108
5.7. Detailed description of study visits during the extension 117
5.8. Sample handling and analysis 119
5.8.1. Treatment and storage of biological samples 119
5.8.2. Laboratory assays 119
5.8.2.1. Hematology 119
5.8.2.2. Biochemistry 119
5.8.2.3. Microbiology 120
5.8.2.4. Parasitology 120
5.8.2.5. Radiology 120
5.8.2.6. Humoral Immunology 120
5.8.3. Blood sampling 120
5.8.3.1. Ancillary studies 124

6. INVESTIGATIONAL PRODUCTS AND ADMINISTRATION 124
6.1. Study vaccines 124

6.1.1. RTS,S/AS01E ... 125
6.1.1.1. RTS,S antigen presentation: ... 125
6.1.1.2. AS01E adjuvant presentation: ... 125
6.1.2. Tritanrix HepB/Hib ... 125
6.1.3. Oral Polio Vaccine ... 126
6.1.4. Meningococcal C conjugate vaccines ... 126
6.1.4.1. Meningitec ... 126
6.1.4.2. NeisVac-C ... 126
6.1.4.3. Menjugate ... 126
6.1.5. Cell-culture rabies vaccines ... 127
6.1.5.1. Sanofi-Pasteur’s Human Diploid Cell Rabies Vaccine ... 127
6.1.5.2. Rabipur/Rabavert Rabies Vaccine ... 127
6.1.5.3. Sanofi-Pasteur’s Vero-cell Rabies Vaccine ... 127
6.2. Dosage and administration ... 128
6.2.1. RTS,S/AS01E (0.5 mL dose) ... 128
6.2.2. TritanrixHepB/Hib (0.5 mL dose) ... 128
6.2.3. OPV ... 128
6.2.4. Meningococcal C conjugate vaccines ... 129
6.2.4.1. Meningitec ... 129
6.2.4.2. NeisVac-C ... 129
6.2.4.3. Menjugate ... 129
6.2.5. Cell-culture rabies vaccines ... 129
6.2.5.1. Sanofi-Pasteur’s Human Diploid Cell Rabies Vaccine (1.0 mL dose) ... 129
6.2.5.2. Rabipur/Rabavert Rabies Vaccine (1.0 mL dose) ... 130
6.2.5.3. Sanofi-Pasteur’s Vero-cell Rabies Vaccine (0.5 mL dose) ... 130
6.3. Storage ... 130
6.4. Treatment allocation and randomization ... 131
6.4.1. Randomization of supplies ... 131
6.4.2. Randomization of subjects ... 131
6.5. Method of blinding and breaking the study blind ... 132
6.6. Replacement of unusable vaccine doses ... 134
6.6.1. Replacement of unusable doses ... 134
6.7. Packaging ... 134
6.8. Vaccine accountability ... 134
6.9. Concomitant medication/treatment ... 135
6.9.1. Concomitant medication/treatment during the primary trial phase ... 135
6.9.2. Concomitant medication/treatment during the extension ... 135

7. HEALTH ECONOMICS ... 135

8. ADVERSE EVENTS AND SERIOUS ADVERSE EVENTS ... 136
8.1. Definition of an adverse event ... 136
8.2. Definition of a serious adverse event ... 137
8.3. Clinical laboratory parameters and other abnormal assessments qualifying as adverse events and serious adverse events ... 138
8.4. Time period, frequency, and method of detecting adverse events and serious adverse events ... 138

8.4.1. Adverse events of specific interest 140
8.4.1.1. Seizures within 30 days of vaccination 140
8.4.1.2. Immune-mediated disorders (IMD) 141
8.4.1.3. Rashes and mucocutaneous lesions 141
8.4.2. Solicited adverse events 141
8.5. Evaluating adverse events and serious adverse events 142
8.5.1. Assessment of intensity 142
8.5.2. Assessment of causality 143
8.6. Follow-up of adverse events and serious adverse events and assessment of outcome 144
8.7. Prompt reporting of serious adverse events to GSK Biologicals 146
8.7.1. Time frames for submitting serious adverse event reports to GSK Biologicals 146
8.7.1.1. For centers using paper CRF 146
8.7.1.2. For centers using RDE with eCRF 146
8.7.2. Completion and transmission of serious adverse event reports to GSK Biologicals 146
8.7.2.1. For centers using paper CRF 146
8.7.2.2. For centers using RDE with eCRF 147
8.8. Regulatory reporting requirements for serious adverse events 148
8.9. Post-study adverse events and serious adverse events 149
8.10. Pregnancy 149
8.11. Treatment of adverse events 149

9. SUBJECT COMPLETION AND WITHDRAWAL 150
9.1. Subject completion 150
9.2. Subject withdrawal 150
9.2.1. Subject withdrawal from the study 150
9.2.2. Subject withdrawal from investigational product 151
9.3. Screen and Baseline Failures 151

10. DATA EVALUATION: CRITERIA FOR EVALUATION OF OBJECTIVES 151
10.1. Primary endpoints 151
10.1.1. Efficacy against clinical malaria disease when primary immunization starts at 6-12 weeks, or 5-17 months of age 151
10.2. Secondary endpoints 152
10.2.1. Efficacy secondary endpoints 152
10.2.1.1. Efficacy against severe malaria disease 152
10.2.1.2. Efficacy against incident severe anemia and malaria hospitalization 152
10.2.1.3. Efficacy; duration of efficacy of a primary course 152
10.2.1.4. Efficacy; role of a booster 152
10.2.1.5. Efficacy under different transmission settings 152
10.2.1.6. Efficacy against secondary case definitions of clinical malaria disease 153
10.2.1.7. Efficacy against prevalence of parasitemia 153
10.2.1.8. Efficacy against prevalence of moderate and severe anemia 153
10.2.2. Safety secondary endpoints 153
10.2.2.1. Safety of a primary course 153
10.2.2.2. Safety of a booster dose 154

10.2.3. Immunogenicity secondary endpoints 154
10.2.3.1. Immunogenicity of a primary course 154
10.2.3.2. Immunogenicity of a booster dose 154
10.2.3.3. Immunogenicity of polio serotypes 1, 2 and 3 155
10.3. Additional Secondary endpoints 155
10.3.1. Additional efficacy secondary endpoints 155
10.3.1.1. Efficacy against other serious illness 155
10.3.1.2. Efficacy against fatal malaria and all-cause mortality 155
10.3.1.3. Effect on growth 155
10.3.1.4. Gender-specific efficacy 156
10.3.2. Additional immunogenicity secondary endpoint 156
10.3.2.1. Immunological correlates of protection 156
10.3.3. Additional safety and immunogenicity secondary endpoints 156
10.3.3.1. Safety and immunogenicity in ‘special’ subgroups 156
10.4. Additional secondary endpoints for the extension 157
10.5. Estimated sample size 159
10.5.1. Sample size and power: primary analysis 159
10.5.1.1. Co-primary endpoint - Efficacy clinical malaria 159
10.5.1.2. Secondary endpoint - Safety 159
10.5.2. Sample size and power: secondary analysis 160
10.5.3. Sample size and power: final analyses 160
10.5.3.1. Efficacy: Evaluation of clinical malaria 160
10.5.3.2. Efficacy: Evaluation of booster 160
10.5.3.3. Evaluation of safety 161
10.5.4. Sample size and power: extension analysis 162
10.5.4.1. Efficacy: evaluation of clinical malaria disease 162
10.5.4.2. Efficacy: evaluation of severe malaria disease 163
10.5.4.3. Safety: evaluation of uncommon safety events 163
10.6. Study cohorts to be evaluated 163
10.6.1. Intention to Treat (ITT) cohort 163
10.6.2. According-To-Protocol (ATP) cohort for analysis of immunogenicity 164
10.6.3. According To Protocol (ATP) cohort for analysis of efficacy 164
10.7. Analyses 164
10.7.1. Covariates 164
10.7.2. Primary analyses at 14.5 months post Dose 1 (for the first 6000 children in each age category) 164
10.7.2.1. Efficacy 164
10.7.2.2. Safety 165
10.7.2.3. Immunogenicity 166
10.7.3. Secondary analyses at the time 250 cases of severe malaria have accumulated 166
10.7.3.1. Efficacy 166
10.7.3.2. Safety 167
10.7.4. Secondary analysis at Month 20 (Visit 22) 167
10.7.5. Secondary analysis at one month post booster (Month 21) 168
10.7.5.1. Polio serotypes 1, 2 and 3 antibody responses 168
10.7.6. Final analyses 168
10.7.6.1. Final analyses - Efficacy 168

10.7.6.2. Final analyses - Safety 169
10.7.6.3. Final analyses - Immunogenicity 169
10.7.7. Extension analyses 170
10.7.7.1. Extension analysis – Risk benefits over time 170
10.7.7.2. Additional analyses 171

11. ADMINISTRATIVE MATTERS 171

12. REFERENCES 172

LIST OF TABLES

		PAGE
Table 1	Formulations of RTS,S	53
Table 2	Approximate number of doses of RTS,S vaccine administered with number of recipients (up to March 2008)	54
Table 3	Summary of pediatric clinical trials in Africa with the candidate malaria vaccines RTS,S (up to March 2008)	55
Table 4	Immunogenicity of Hepatitis B antigens in coadministration with DTPw Hib and MCC	68
Table 5	Intervals between study visits for subjects that have their last visit in the primary trial phase (Visit 34) BEFORE (and including) 30 Jun 2012	83
Table 6	Intervals between study visits for subjects that have their last visit in the primary trial phase (Visit 34) after 30 Jun 2012 and BEFORE (and including) 30 Sep 2013	83
Table 7	Case definitions for clinical malaria	95
Table 8	Primary case definition of severe malaria disease	96
Table 9	Secondary case definitions of severe malaria disease	97
Table 10	Case definitions for malaria hospitalization	97
Table 11	Case definitions for fatal malaria	97
Table 12	Case definitions of incident severe anemia	98
Table 13	Case definitions of prevalent anemia	98
Table 14	Case definitions for all medical hospitalization	98
Table 15	Case definitions for all-cause mortality	98
Table 16	Case definitions for pneumonia	99
Table 17	Case definitions for sepsis	99
Table 18	List of study procedures	102
Table 19	List of study procedures of the extension	105
Table 20	Intervals between study visits	107

Table 21 Intervals between study visits for subjects that have their last visit in the primary trial phase (Visit 34) BEFORE (and including) 30 Jun 2012 107

Table 22 Intervals between study visits for subjects that have their last visit in the primary trial phase (Visit 34) after 30 Jun 2012 and BEFORE (and including) 30 Sep 2013 108

Table 23 Laboratory immunological assays to be performed 120

Table 24 Summary of blood sampling timepoints/immunological assays and volumes to be collected in children 6-12 weeks of age at first vaccination 121

Table 25 Summary of blood sampling timepoints/immunological assays and volumes to be collected in children 5-17 months of age at first vaccination 122

Table 26 Summary of blood sampling timepoints/immunological assays and volumes to be collected in children of both age categories during extension 123

Table 27 Reporting periods for adverse events and serious adverse events 139

Table 28 Reporting periods for adverse events and serious adverse events, during the extension 139

Table 29 Intensity scales for solicited symptoms in infants/toddlers and children less than 6 years of age 142

Table 30 Power to detect fold-differences in SAE increases at primary analysis at Month 14.5 160

Table 31 Power to detect fold-differences in SAE increases at final analysis at Month 32 162

Table 32 Power to detect VE against severe malaria disease 163

Table 33 Power to detect SAEs 163

LIST OF FIGURES

PAGE

Figure 1 Study design 76

Figure 2 Study design of the extension 77

LIST OF APPENDICES

		PAGE
Appendix A	Administrative Matters	179
Appendix B	Handling of Biological Samples Collected by the Investigator	186
Appendix C	Shipment of Biological Samples	189
Appendix D	Vaccine supplies, packaging and accountability	190
Appendix E	Assessment of a potential case of severe malaria disease	192
Appendix F	Determination of *Plasmodium falciparum* asexual parasite density	197
Appendix G	Radiography	200
Appendix H	Microbiology methodology and interpretation of results	205
Appendix I	The WHO pediatric clinical staging system (for HIV disease)	208
Appendix J	Documentation of bednet usage and indoor residual spraying	210
Appendix K	Anthropometry methods	211

LIST OF ABBREVIATIONS

List of abbreviations amended 26 November 2009, 23 January 2012

Note: Abbreviations for vaccination groups

The abbreviations below are used to describe the vaccination groups in some sections of the protocol.

R3R: Children to receive 3 doses of RTS,S/AS01E on a 0, 1, 2 schedule with an RTS,S/AS01E booster.

R3C: Children to receive 3 doses of RTS,S/AS01E on a 0, 1, 2 schedule without an RTS,S/AS01E booster.

C3C: Children to receive 3 doses of a comparator vaccine on a 0, 1, 2 schedule with a dose of comparator vaccine at Visit 22.

AE	Adverse event
ALT	Alanine aminotransferase
anti-CS	Antibody to the *P. falciparum* circumsporozoite (CS) repeat domain
anti-HBs	Antibody to the hepatitis B surface antigen
ATP	According to protocol
BIOMNIS	Biomnis Recherche Clinique, Lyon, France
CEVAC	Center for Vaccinology, Ghent University, Belgium
CI	Confidence interval
CMI	Cell-mediated immunity
CRF	Case report form
CS	Circumsporozoite protein of *P. falciparum*
CSF	Cerebro-spinal fluid
CXR	Chest X Ray
DTPw/Hib	Diphtheria, Tetanus, Pertussis (whole cell) and *Hemophilus influenzae* type B conjugate vaccine

DSMB	Data safety monitoring board
eCRF	electronic Case Report Form
ELISA	Enzyme-linked immunosorbent assay
EPI	Expanded program on immunization
EU/mL	ELISA unit per milliliter
FDA	Food and Drug Administration, United States
GCP	Good clinical practice
GMT	Geometric mean titer
GSK	GlaxoSmithKline
HBsAg	Hepatitis B surface antigen
HBV	Hepatitis B virus
Hep B	Hepatitis B
HDC Rabies Vaccine	Human diploid cell rabies vaccine (manufactured by Sanofi Pasteur, previously marketed as 'Imovax®' the product is now marketed as 'HDC Rabies Vaccine')
HIV	Human immunodeficiency virus
HSPH	Harvard School of Public Health, Cambridge, Massachusetts, USA
IB	Investigator's brochure
ICF	Informed consent form
ICH	International Committee on Harmonization
IDMC	Independent Data Monitoring Committee
IEC	Independent ethics committee
IFN-gamma	Interferon gamma
IM	Intramuscular
IMD	Immune-mediated disorder
IPTi	Intermittent preventative treatment for malaria (Infants)

IRB	Institutional review board
IU	International unit
ITN	Insecticide-treated bednet
kg	Kilogram
LLN	Lower limit of normal
LP	Lumbar puncture
LSM	Local safety monitor
MCC	Meningococcal C conjugate vaccine
MedDRA	Medical Dictionary for Regulatory Activities
mg	Milligram
mL	Milliliter
MPL®	3-deacylated monophosphoryl lipid A
MVI	Malaria Vaccine Initiative
P. falciparum	*Plasmodium falciparum*
PATH	Program for Appropriate Technology in Health
PCD	Passive case detection
PCEC	Purified chick embryo cell culture vaccine: previously manufactured by Chiron Behring, the product is now produced by Novartis. The product is marketed in the USA as RabAvert®, elsewhere as Rabipur®
PFS	Pre-filled syringe
PI	Principal Investigator
PMCT	Prevention of mother-to-child transmission
QS 21	‘*Quillaja saponaria* 21’: a triterpene glycoside purified from the bark of the soap bark tree, *Quillaja saponaria*
RBC	Red blood cell
RDE	Remote Data Entry

RTS,S	Particulate antigen, containing both RTS and HBs proteins
SAE	Serious adverse event
SAP	Statistical analysis plan
SOP	Standard operating procedure
SP	Sulfadoxine-pyrimethamine
ULN	Upper limit of normal
VE	Vaccine efficacy
WC	White cells
WRAIR	Walter Reed Army Institute of Research

GLOSSARY OF TERMS

Glossary of terms amended 26 November 2009, 01 December 2010

Adverse event:	Any untoward medical occurrence in a patient or clinical investigation subject, temporally associated with the use of a medicinal product, whether or not considered related to the medicinal product.<br><br>An AE can therefore be any unfavorable and unintended sign (including an abnormal laboratory finding), symptom, or disease (new or exacerbated) temporally associated with the use of a medicinal product. For marketed medicinal products, this also includes failure to produce expected benefits (i.e. lack of efficacy), abuse or misuse.
Blinding:	A procedure in which one or more parties to the trial are kept unaware of the treatment assignment in order to reduce the risk of biased study outcomes. In a single-blind trial, the investigator and/or his staff are aware of the treatment assignment but the subject is not. In an observer-blind study, the subject and the study personnel involved in the clinical evaluation of the subjects are blinded while other study personnel may be aware of the treatment allocation. When the investigator and sponsor staff who are involved in the treatment or clinical evaluation of the subjects and review/analysis of data are also unaware of the treatment assignments, the study is double blind. Partially-blind is to be used for study designs with different blinding levels between different groups, e.g. double-blinded consistency lots which are open with respect to the control group. The level of blinding is maintained throughout the conduct of the trial, and only when the data are cleaned to an acceptable level of quality will appropriate personnel be unblinded or when required in case of a serious adverse event.
Central Study Coordinator:	An individual assigned by and centrally located at GSK Biologicals at Rixensart who is responsible for assuring the co-ordination of the operational aspects and proper conduct of a clinical study, including compliance with International Conference on Harmonization (ICH) Harmonized Tripartite Guideline for Good Clinical Practice (GCP) and GSK policies and standard operating procedures.

Data Safety Monitoring Board (DSMB): Note: called IDMC since October 2008	The DSMB is an independent committee appointed to oversee ethical and safety aspects of the conduct of the study. See Section 5.1.4.1 for a full overview of the role and structure of the DSMB.
Eligible:	Qualified for enrolment into the study based upon strict adherence to inclusion/exclusion criteria.
eTrack:	GSK’s clinical trials tracking tool.
Evaluable:	Meeting all eligibility criteria, complying with the procedures defined in the protocol, and, therefore, included in the according-to-protocol (ATP) analysis (see Sections 4.4 and 10.6 for details on criteria for evaluability).
Independent Data Monitoring Committee (IDMC) (previously named DSMB):	The IDMC is an independent committee appointed to oversee ethical and safety aspects of the conduct of the study. See Section 5.1.4.1 for a full overview of the role and structure of the IDMC.
Investigational product:	A pharmaceutical form of an active ingredient or placebo being tested or used as a reference in a clinical trial, including a product with a marketing authorization when used in a way different from the approved form, or when used for an unapproved indication, or when used to gain further information about an approved use.
Local Safety Monitor (LSM):	The overall role of the Local Safety Monitor, an experienced physician based in-country, will be to support the study investigators and to act as a link between the investigators and the Data Safety Monitoring Board (DSMB) (see Section 5.1.4.2 for further details on the LSM).
Medical Monitor:	An individual medically qualified to assume the responsibilities of the sponsor (GSK Biologicals) especially in regards to the ethics, clinical safety of a study and the assessment of adverse events.
Pre-patent Period (PPP):	The time in days between experimental sporozoite challenge and first detection of parasitemia by peripheral blood thick smear.
Protocol amendment:	ICH defines a protocol amendment as: “A written description of a change(s) to or formal clarification of a protocol.” GSK Biologicals further details this to include a change to an approved protocol that affects the safety of subjects, scope of the investigation, study design, or

	scientific integrity of the study.
Protocol administrative change:	A protocol administrative change addresses changes to only logistical or administrative aspects of the study. N.B. Any change that falls under the definition of a protocol amendment (e.g. a change that affects the safety of subjects, scope of the investigation, study design, or scientific integrity of the study) MUST be prepared as an amendment to the protocol.
Randomization:	Process of random attribution of treatment to subjects in order to reduce bias of selection.
Serious Adverse Event:	A serious adverse event (SAE) is any untoward medical occurrence that:<br>a. results in death;<br>b. is life-threatening;<br>c. requires hospitalization or prolongation of existing hospitalization;<br>d. results in disability/incapacity;<br>e. a seizure within 30 days of vaccination.<br>*NOTE: In this study all seizures occurring within a 30-day period of vaccination will be notified as SAEs. Key information pertaining to seizures occurring within 7 days of vaccination will be documented in the CRF/eCRF [refer to Bonhoeffer, 2004].*<br>f. medical or scientific judgment should be exercised in deciding whether reporting is appropriate in other situations, such as important medical events that may not be immediately life-threatening or result in death or hospitalization but may jeopardize the subject or may require medical or surgical intervention to prevent one of the other outcomes listed in the above definition. These should also be considered serious. Examples of such events are invasive or malignant cancers, intensive treatment in an emergency room or at home for allergic bronchospasm and blood dyscrasias.<br>A full definition of the events that constitute SAEs can be found in Section 8.2).

Amended 01 December 2010

Solicited adverse event:	Adverse events (AEs) to be recorded as endpoints in the clinical study. The presence/occurrence/intensity of these events is actively solicited from the subject or an observer during a specified post-vaccination follow-up period.
Study End:	Throughout the document, “study end” means the end of the primary trial phase at Visit 34 (Month 32). When the end of the extension phase at Visit 38 is meant, it will be described as such.<br>Amended 01 December 2010
Study Monitor:	An individual assigned by the sponsor who is responsible for assuring proper conduct of a clinical study.
Subject:	Term used throughout the protocol to denote an individual that has been contacted in order to participate or participates in the clinical study, either as a recipient of the investigational product(s) or as a control.
Treatment:	Term used throughout the clinical study to denote a set of investigational product(s) or marketed product(s) or placebo intended to be administered to a subject, identified by a unique number, according to the study randomization or treatment allocation.
Treatment number:	A unique number identifying a treatment to a subject, according to the study randomization or treatment allocation.
Unsolicited adverse event:	Any adverse event (AE) reported in addition to those solicited during the clinical study. Also any “solicited” symptom with onset outside the specified period of follow-up for solicited symptoms will be reported as an unsolicited adverse event.

1. INTRODUCTION

1.1. Malaria

Four species of the *Plasmodium* protozoan parasite cause malaria in humans (*P. falciparum*, *P. vivax*, *P. ovale* and *P. malariae*). Of these four parasites, *P. falciparum* is the major cause of severe morbidity and mortality.

There is no doubt of the importance of *P. falciparum* malaria as a major cause of human suffering and economic drain across sub-Saharan Africa [Breman 2001a; Gallup 2001]. In this region, it causes the deaths of between 0.5 and 2.0 million children every year and is a common reason for admission to hospital, leading each year to about 300 million clinical episodes in children under five years [Breman 2001a].

The incidence of malaria in much of Africa is increasing for a variety of reasons: changes in agricultural practices, armed conflicts, migration of refugees, increasing drug resistance to conventional anti-malarial drugs, and insecticide resistance of the mosquito vectors. It is estimated that without effective control the number of cases of clinical malaria will more than double over the next 20 years. The burden of malaria at the country level correlates closely with the rate of economic development even after adjustment for confounding factors, indicating that malaria is an important constraint on economic progress [Breman 2001b].

Clinical manifestations of *P. falciparum* disease appear as a result of the parasite infection of the red blood cells (RBC). Initial symptoms may include fever, chills, headache, joint and muscle pain, sweating, and vomiting. Acute complications may result from hemolysis leading to anemia and the propensity of infected RBCs to become adhesive and to be sequestered in capillaries thus causing local inflammatory reactions and damage to vital organs, leading to cerebral, hepatic, renal or pulmonary malaria. In *P. falciparum* malaria, an untreated acute attack can progress very rapidly and death may occur within a short timeframe.

Efforts to develop vaccines that target each stage of the parasite life cycle, to identify protective antigens and to understand the nature of the protective immune responses have been ongoing for the past three decades. The approach of GlaxoSmithKline (GSK) Biologicals has been to focus on vaccines that target the sporozoite and intra-hepatic stages of the parasites (i.e. the pre-erythrocytic stage).

1.2. Hepatitis

Hepatitis B is an infection of the liver due to hepatitis B virus (HBV); it is an important public health problem across the developing world. World-wide approximately 350 million people carry HBV and about 1 million chronic carriers die annually [Vryheid 2001]. The age at infection determines the likelihood of the infection becoming chronic: 90% if infected in infancy, 30% to 50% if infected between the ages of 1 to 4 years, and low in adulthood. For those that become chronically infected during childhood the risk of

death from HBV-related liver cancer or cirrhosis in adult life is approximately 25% [WHO 2003].

1.3. RTS,S candidate vaccine

The candidate vaccine antigen consists of sequences of the circumsporozoite (CS) protein and the hepatitis B surface antigen (HBsAg). The HBsAg contained in the RTS,S candidate malaria vaccine is encoded by the hepatitis B virus S protein gene that is identical to the gene used to express HBsAg in GSK Biologicals' *Engerix-B*® vaccine against hepatitis B. As a result, vaccines containing RTS,S are expected to also provide protection against hepatitis B.

RTS,S has been evaluated to demonstration of field efficacy in the pediatric population with the AS02 adjuvant (proprietary oil-in-water emulsion formulated with MPL® and Stimulon® QS21 immunostimulants). The RTS,S antigen has more recently been evaluated with the AS01 adjuvant (liposome formulation with MPL and QS21 immunostimulants) in a strategy to improve vaccine efficacy (VE) and the duration of efficacy. Both the AS02 and AS01 adjuvant systems have a number of similar key components (Table 1) and therefore safety data collected with RTS,S administered with the AS02 family of adjuvants is supportive of the RTS,S/AS01E development.

Table 1 Formulations of RTS,S

	Freeze-dried fraction	Liquid fraction				
**Formulation**	**RTS,S (µg)**		**MPL (µg)**	**QS21 (µg)**	**Dose Volume**	
RTS,S/AS02A (0.5 mL dose)	50	Oil-in-water emulsion	50	50	0.5 mL	Efficacy demonstrated in adults in The Gambia (Malaria-005[a])
RTS,S/AS02A (0.25 mL dose)	25	Oil-in-water emulsion	25	25	0.25 mL	Efficacy against clinical disease in children in Mozambique (Malaria-026[b])
RTS,S/AS02D	25	Oil-in-water emulsion	25	25	0.5 mL	Pediatric formulation (Malaria-034[c], -038[d], -040[e])
RTS,S/AS01B	50	Liposomes	50	50	0.5 mL	Efficacy in challenge model, adults (Malaria-027[f]), endemic countries (Malaria-044[g])
RTS,S/AS01E	25	Liposomes	25	25	0.5 mL	Pediatric formulation (Malaria-046[h], -047[j], -049[k], -050[m])

a Bojang 2001; Alloueche 2003; Pinder 2004; Reece 2004; GSK data on file, Malaria-005, Clinical Study Report, 2006
b GSK data on file, Malaria-026, Clinical Study Report, 2004; Alonso 2004; Alonso 2005
c GSK data on file, Malaria-034, Clinical Study Report, 2005
d GSK data on file, Malaria-038, Clinical Study Protocol, 2005
e GSK data on file, Malaria-040, Clinical Study Protocol, 2005
f GSK data on file, Malaria-027, Clinical Study Protocol, 2003
g GSK data on file, Malaria-044, Clinical Study Protocol, 2005
h GSK data on file, Malaria-046, Clinical Study Protocol, 2006
j GSK data on file, Malaria-047, Clinical Study Protocol, 2006
k GSK data on file, Malaria-049, Clinical Study Protocol, 2006
m GSK data on file, Malaria-050, Clinical Study Protocol, 2007

1.4. Key clinical, efficacy and immunogenicity data

A comprehensive summary of the results of reported trials to date can be found in the current version of the Malaria Vaccine Investigator Brochure. A brief summary of results to date is presented below. The numbers of doses of RTS,S-containing vaccines and the

number of recipients to date (March 2008) is tabulated in Table 2. A summary of clinical studies of the RTS,S vaccine in children is provided in Table 3.

Amended 19 August 2008

Table 2 Approximate number of doses of RTS,S vaccine administered with number of recipients (up to March 2008)

Candidate Malaria Vaccine	Subject population	Recipients	Doses administered
RTS,S/AS02A	Malaria-naïve adults [a]	302	723
	Adults in sub-Saharan Africa	424	1030
	Children in sub-Saharan Africa	1572	4562
RTS,S/AS02D	Children in sub-Saharan Africa	190	550
	Infants in sub-Saharan Africa	276	802
RTS,S/AS01B	Malaria-naïve adults [a]	52	146
	Adults in sub-Saharan Africa	85	242
RTS,S/AS01E	Children in sub-Saharan Africa	90	261

a subjects who took part in studies conducted in the USA and Belgium, where there is no naturally occurring transmission of malaria

1.4.1. The RTS,S/AS02A candidate malaria vaccine; key clinical efficacy, safety and immunogenicity data in children

Early clinical development of the RTS,S malaria candidate vaccine was initiated in studies in malaria-naïve adults in collaboration with the WRAIR in which confirmation of the efficacy, safety and immunogenicity of the RTS,S/AS02A vaccine formulation was demonstrated [Stoute 1997; Kester 2001]. Two doses of RTS,S/AS02A (0.5 mL) provided protection to 37.8% healthy non-immune volunteers against homologous sporozoite challenge (pooled results for WRMAL-004 [Report, GSK data on file] & -005 [Report, GSK data on file]); 3 doses demonstrated protection of 43.2% of subjects (pooled results for WRMAL-004 & Malaria-012 [Report, GSK data on file]). In subjects not protected, the prepatent period was significantly prolonged in the RTS,S/AS02A group compared to control (WRMAL-004, WRMAL-005 & Malaria-012). Protective efficacy was low following re-challenge six months after Dose 3 of RTS,S/AS02A, but a statistically significant difference between prepatent period for vaccinees compared to infectivity control was observed (WRMAL-004, WRMAL-005 & Malaria-012). A strong humoral immune response to the RTS,S/AS02A vaccine in terms of anti-CS and anti-HBs antibodies was demonstrated in all the adult studies in malaria-naïve individuals. Evaluations of the CMI response showed consistently that administration of RTS,S/AS02A induced strong cellular Th1 T-cell responses, specific to the vaccine antigen [Lalvani, 1999; Epstein, 2004; Sun, 2003].

Table 3 Summary of pediatric clinical trials in Africa with the candidate malaria vaccines RTS,S (up to March 2008)

Study no.	Study design	Study population	Objectives	Study groups	Status (March 2008)
Malaria -015	Double-blind, randomized, controlled	Children 6-11y<br>*The Gambia*	Primary: safety & immunogenicity<br>Secondary: humoral responses	RTS,S/AS02A (n = 60) ‡<br>Rabies vaccine (n = 30)	Completed
Malaria-020	Double-blind, randomized, controlled	Children 1-5y<br>*The Gambia*	Primary: safety & immunogenicity<br>Secondary: humoral responses	RTS,S/AS02A (n = 90) ‡<br>Rabies vaccine (n = 45)	Completed
Malaria-025	Double-blind, randomized, controlled	Children 1-4 y<br>*Mozambique*	Primary: safety & reactogenicity<br>Secondary: humoral responses	RTS,S/AS02A (n = 30)<br>Engerix B (n = 30)	Completed
Malaria-026	Double-blind, randomized, controlled	Children 1-4 y<br>*Mozambique*	Primary: efficacy against clinical disease<br>Secondary: safety and immunogenicity	Cohort 1: RTS,S/AS02A (n = 782)<br>Engerix-B or Prevnar and Hiberix (n = 782)<br>Cohort 2: RTS,S/AS02A (n = 208)<br>Engerix-B or Prevnar and Hiberix (n = 208)	Completed
Malaria-034	Double-blind, randomized, controlled	Children 3-5 y<br>*Mozambique*	Primary: safety, non-inferiority of ab response to CS<br>Secondary: non-inferiority of ab response to HBsAg	RTS,S/AS02D (n = 100)<br>RTS,S/AS02A (n = 100)	Completed
Malaria-038	Double-blind, randomized, controlled	Infants 6-12 w<br>*Mozambique*	Primary: safety<br>Secondary: immunogenicity	RTS,S/AS02D (n = 110) †<br>Engerix-B (n = 110) †	Ongoing; interim data available
Malaria-039*	Single-blind, randomized, controlled	Children 1-4 y **<br>*Mozambique*	Primary: safety<br>Secondary: immunogenicity	n/a	Completed
Malaria-040	Double-blind, randomized, controlled	Infants 6-10 w<br>*Tanzania*	Primary: safety & non-inferiority of EPI antigens<br>Secondary: immunogenicity & efficacy vs infection	RTS,S/AS02D + TETRActHib (n = 170)<br>Engerix-B + TETRActHib (n = 170)	Ongoing; interim data available
Malaria-046	Double-blind, randomized, controlled	Children 18 m - 4 y<br>*Gabon*	Primary: safety, non-inferiority of ab response to CS<br>Secondary: safety and immunogenicity	RTS,S/AS01E (n = 90)<br>RTS,S/AS02D (n = 90)	Completed
Malaria-047	Partially-blind, randomized, controlled	Children 5-17 m<br>*Ghana*	Primary: safety<br>Secondary: safety and immunogenicity	RTS,S/AS01E at 0, 1 m (n = 90)<br>RTS,S/AS02D at 0, 1 m (n = 90)<br>RTS,S/AS01E at 0, 1, 2 m (n = 90)<br>RTS,S/AS02D at 0, 1, 2 m (n = 45)<br>Rabies vaccine at 0, 1, 2 m (n = 45)<br>RTS,S/AS01E at 0, 1, 7 m (n = 90)<br>RTS,S/AS02D at 0, 1, 7 m (n = 90)	Ongoing; interim data available
Malaria-049	Double-blind, randomized, controlled	Children 5-17 m<br>*Tanzania* & *Kenya*	Primary: efficacy against clinical disease<br>Secondary: safety and immunogenicity	RTS,S/AS01E (n = 445)<br>Rabies vaccine (n = 445)	Ongoing
Malaria-050	Open, randomized, controlled	Infants 6-10 w<br>*Tanzania, Ghana & Gabon*	Primary: safety<br>Secondary: safety and immunogenicity	EPI vaccines alone (n=170)<br>EPI vaccines & RTS,S/AS01E at 0, 1, 2 m (n=170)<br>EPI vaccines & RTS,S/AS01E at 0, 1, 7 m (n=170)	Ongoing

* Malaria-039 is the 2-year follow-up of the Malaria-026 study. No study vaccines were given during the follow-up period.
** age at time of primary vaccination
n: number of enrolled (to be enrolled) subjects ab: antibody

† Vaccines staggered with TETRActHib doses
‡ A third of subjects enrolled to receive RTS,S/AS02A got a 1/5 dose volume, a third 1/2 dose volume and a third full dose volume. The '1/2 dose volume' contained 25 µg antigen and 0.25µL adjuvant.

Amended 19 August 2008

The RTS,S/AS02A vaccine progressed to evaluation in subjects under conditions of natural transmission. In adult males from The Gambia, VE against infection adjusted for covariates was 71% (95% CI: 46 to 85; p< 0.001) during the first 2 months and 34% (95% CI: 8.0 to 53, p=0.014) for the entire 15 week surveillance period (Malaria-005 [Report, GSK data on file], [Bojang 2001]). VE, adjusted for covariates, following a booster dose given during a second year malaria season was 47% (95% CI: 3 to 71; p=0.039). Protection was not limited to the NF54 parasite genotype from which the vaccine was derived [Alloueche, 2003]. Safety surveillance over approximately 5 years showed no safety signal (Malaria-016, -017 & -018 [Report, GSK data on file]). A strong humoral immune response to the RTS,S/AS02A vaccine in terms of anti-CS and anti-HBs antibodies was demonstrated. Overall, the kinetics of the humoral immune response induced by vaccination with RTS,S/AS02A were similar in malaria-naïve and experienced populations, while the absolute GMT values appeared to be higher in malaria-naïve volunteers. The vaccine induced and boosted Th1-like cellular immunity to several T-cell epitopes in a population naturally exposed to malaria [Pinder 2004; Reece 2004].

The RTS,S/AS02A candidate vaccine progressed to clinical evaluation in children. Two age de-escalation and dose comparison trials (which compared doses with 10 µg, 25 µg and 50 µg of antigen corresponding to volume fractions of RTS,S/AS02A: 0.1 mL, 0.25 mL and 0.5 mL respectively) enrolled a total of 225 children aged 1 to 11 years from The Gambia (Malaria-015 [Report, GSK data on file] & -020 [Report, GSK data on file], [Bojang 2001]). From these trials, the 0.25 mL dose was selected due to equivalent immunogenicity and slightly less reactogenicity in the 0.25 mL group compared to the 0.5 mL group; immunogenicity was consistently lowest in the 0.1 mL group. The safety and immunogenicity of the RTS,S/AS02A 0.25 mL dose was further confirmed in another study, Malaria-025 [Report, GSK data on file], conducted in 1 to 4 year old children in Mozambique.

Subsequently, a large safety, immunogenicity and efficacy trial in children aged 1 to 4 years from an area of high transmission was conducted in Mozambique, enrolling a total of 2022 subjects (Malaria-026 [Report, GSK data on file]; [Alonso 2004]; [Alonso 2005]; extension study Malaria-039). In this study, 3 doses of RTS,S/AS02A (0.25 mL dose) were administered according to a 0, 1, 2-month schedule to approximately 1000 children. The primary endpoint of this trial investigated vaccine efficacy against the onset of clinical malaria disease. Up to 18 months post Dose 3, the VE determined as the time to the first clinical episode (after adjustment for covariates) was 35.3% (95% CI: 21.6 to 46.6; p=0.001). VE against multiple attacks was 32.4% (95% CI: 17.6 to 44.5; p=0.0001) up to 18 months post Dose 3.

In this Phase IIb study, severe malaria disease was not specified per-protocol, but cases were collected prospectively and evaluated according to an agreed case definition to meet safety-monitoring guidelines. The analysis was defined in the SAP. Against severe malaria, VE was 48.6% (95% CI 12.3 to 71.0; p=0.02) over 18 months post Dose 3.

Over the period of months 21 to 33, efficacy appeared to wane when assessed as efficacy against first clinical episode (VE 16.6% [95% CI -2.9 to 32.4; p=0.09]), against multiple clinical episodes (VE 22.3% [95% CI 0.8 to 39.2; p=0.04).

In the overall surveillance period (Months 0 to 33), there was a tendency for the proportion of children experiencing a SAE to be lower in the RTS,S/AS02A group compared to the control groups in both Cohorts 1 and 2 (23.0% vs. 31.7%, respectively). The pattern of morbidity was similar to that previously observed at the study site and described in the region.

Anti-CS antibody GMTs of 180.3 (95% CI 164.0 to 198.2) were recorded 1 month post Dose 3 from subjects in Cohort 1. GMTs waned progressively at Month 8½ (GMT 42.9 [95% CI 38.7 to 47.5]), Month 21 (GMT 16.2 [95% CI 14.5 to 18.1]) and Month 33 (GMT 10.1 [95% CI 8.8 to 11.4]), but remained significantly higher than those observed in recipients of control vaccine (GMT 0.3 [95% CI 0.3 to 0.4] at all timepoints).

1.4.2. The RTS,S/AS02D candidate malaria vaccine; key clinical efficacy, safety and immunogenicity data in infants

The target population for the candidate malaria vaccine is infants living in malaria endemic parts of Africa. With this target population in mind, a 0.5 mL variant of the 0.25 mL dose of RTS,S/AS02A (RTS,S/AS02D) was developed for compatibility with standard auto-disable EPI syringe. Following demonstration of proof-of-concept of efficacy in children in Mozambique, 2 trials have looked at the RTS,S/AS02D vaccine in infants in Africa.

In Malaria-038, a dose of RTS,S/AS02D was given at 10, 14 and 18 weeks of age, with an EPI vaccine, *TETRActHib* (DTPw/Hib vaccine, Aventis Pasteur, Lyon, France) at 8, 12 and 16 weeks of age. Results of this trial have been published [Aponte, 2007]. The vaccine was shown to have a good safety profile and was well-tolerated: no safety signal was identified and the reactogenicity was similar to that of the DTPw/Hib vaccine administered as part of the trial. Grade 3 reactogenicity was rare and no Grade 3 local symptoms were reported. 33 SAEs were reported for 17 recipients of RTS,S/AS02D and 35 SAEs for 17 recipients of *Engerix-B* control vaccine.

RTS,S/AS02D was shown to be immunogenic; anti-CS Geometric Mean antibody Titers (GMT) of 199.9 EU/mL (95% CI: 150.9 to 264.7) were achieved. Efficacy assessed as time to first infection over a 3 month period after the third dose was 65.9% (95% CI: 42.6 to 79.8, p < 0. 0001).

Malaria-040 was initiated following an assessment of safety of the first 3 doses of vaccine in Malaria-038. In Malaria-040, a dose of RTS,S/AS02D was given at 6, 10 and 14 weeks of age, in co-administration with an EPI vaccine, *TETRActHib* (DTPw/Hib vaccine).

RTS,S/AS02D and *TETRActHib* in co-administration demonstrated an acceptable general reactogenicity profile that was similar to that observed in Malaria-038 when the vaccines were administered in a staggered fashion. RTS,S/AS02D local reactogenicity in co-administration was similar to that observed when it was given alone in Malaria-038. Reactogenicity of *TETRActHib* was similar whether it was administered in a co-administered fashion with RTS,S/AS02D or with *Engerix-B*. Grade 3 reactogenicity was rare. 13 SAEs were reported for 13 recipients of RTS,S/AS02D and 19 SAEs for 19 recipients of *Engerix-B*.

Non-inferiority criteria of all co-administered EPI antigens (DTPw/Hib) were met for all the co-administered EPI antigens (D, T, Pw, Hib): anti-HBs antibody titers were 667.4 mIU/mL (95% CI 533.8 to 834.4). Anti-CS antibody GMTs in co-administration were 69.5 ELU/mL (95% CI 53.9 to 89.6).

1.4.3. RTS,S/AS01 vaccine; key clinical efficacy, safety and immunogenicity data

The RTS,S/AS01 vaccine has been developed with the aim of improving the immune response to the antigen while retaining a similar safety profile to that of RTS,S/AS02A. Data from three trials of RTS,S/AS01 are currently available—Malaria-027, carried out in malaria-naive adults in the USA, Malaria-044 in adults in a malaria-endemic region of Kenya and Malaria-046 in children 18 months to 4 years of age in a malaria-endemic region of Gabon.

Preliminary clinical data for the RTS,S/AS01B vaccine in malaria-naïve adults are available for Malaria-027 [Report GSK data on file], a double-blind comparison of RTS,S/AS02A and RTS,S/AS01B, with mosquito challenge followed by re-challenge 6 months after primary challenge. Following primary challenge, VE tended to be higher in the RTS,S/AS01B group compared to the RTS,S/AS02A group (50.0% [95% CI: 32.9, 67.1] vs 31.8% [95% CI: 17.6, 47.6], respectively); VE was significantly greater in both vaccine groups compared to infectivity control ($p \leq 0.001$). Following re-challenge 5/9 subjects (55.6%) in the RTS,S/AS01B and RTS,S/AS02A groups overall were infected; all infectivity control subjects became infected; the point estimate for VE was 44.4% [95% CI: 10.9, 79.2]. Safety and reactogenicity were comparable in both the RTS,S/AS01B and RTS,S/AS02A groups. Two SAEs were reported in two subjects from the RTS,S/AS01B group and one SAE was reported in a subject from the challenge control group; none were fatal and none were considered to be related to study vaccine. RTS,S/AS01B and RTS,S/AS02A were both highly immunogenic for anti-CS antibodies and anti-HBs antibodies. Anti-CS antibody GMTs were significantly greater in the RTS,S/AS01B group compared to the RTS,S/AS02A group at 1 month post dose 2 ($p<0.0001$), 2 weeks post dose 3 ($p=0.0079$) and 3 months post dose 3 ($p=0.0173$). GMTs for anti-CS antibodies were significantly greater in subjects who were protected compared to those not protected following primary challenge at 1 month post dose 1 ($p=0.0076$), 1 month post dose 2 ($p=0.0004$), 2 weeks post dose 3 ($p<0.0001$) and 3 months post dose 3 ($p=0.0018$). In subjects who underwent re-challenge (i.e. protected subjects), all were still seropositive for anti-CS antibodies and had seroprotective anti-HBs titers at 10 months post dose 3. CS-specific CD4+ T-cell responses were greater in subjects from the RTS,S/AS01B group compared to the RTS,S/AS02A group, and in subjects who were protected compared to non-protected subjects following primary challenge.

Clinical data for the RTS,S/AS01B vaccine in semi-immune adults are available for Malaria-044, a double-blind, controlled study of RTS,S/AS02A and RTS,S/AS01B in adults living in Kenya. The two vaccines RTS,S/AS02A and RTS,S/AS01B in Kenya, induced similar levels of protective efficacy against infection over a 14 week period (29.5% [95% CI: -15.4 to 56.9, $p=0.164$] and 31.7% [95% CI: -11.6 to 58.2, $p=0.128$], respectively); pooled data yielded an unadjusted VE of 31% (95% CI: -4.7 to 54.4, $p=0.081$). The efficacy results for RTS,S/AS02A were consistent with previous studies in

semi-immune adults. Safety and reactogenicity were comparable in both the RTS,S/AS01B and RTS,S/AS02A groups. In total, 1/85 (1.2%) subjects from the RTS,S/AS02A group, 5/85 (5.9%) subjects from the RTS,S/AS01B group and 5/85 (5.9%) subjects from the rabies group reported a SAE. None were considered to be related to study vaccine and none were fatal. A strong humoral immune response to the RTS,S/AS01B and RTS,S/AS02A vaccines in terms of anti-CS and anti-HBs antibodies was demonstrated. At each post vaccination timepoint GMTs for anti-CS antibodies were higher in the RTS,S/AS01B group compared to the RTS,S/AS02A group. Although GMTs for anti-CS antibodies decreased by 10 months post Dose 3, at least 96% of subjects were seropositive. GMTs for anti-HBs antibodies were comparable in the RTS,S/AS01B group and RTS,S/AS02A group up to 10 months post Dose 3. GMTs against HBs decreased by 10 months post Dose 3, however, seroprotection rates remained above 94% of subjects in both vaccine groups.

Clinical data for the RTS,S/AS01E vaccine in semi-immune children are available for Malaria-046, a double-blind, controlled study of RTS,S/AS01E and RTS,S/AS02D in children aged 18 months to 4 years from Gabon. Both RTS,S/AS01E and RTS,S/AS02D were well tolerated. Local reactogenicity of Grade 3 severity was rare. One child in each group had Grade 3 swelling and there was no Grade 3 pain. There were no Grade 3 solicited general symptoms reported. No unsolicited symptom was considered to be causally related to vaccine in either group. In total, to 1 month post Dose 3, 8/90 (8.9%) subjects reported SAEs in the RTS,S/AS01E group and 5/90 (5.6%) subjects reported SAEs in the RTS,S/AS02D group; none were fatal and none were considered to be related to study vaccine. RTS,S/AS01E and RTS,S/AS02D were both highly immunogenic for anti-CS antibodies. Anti-CS antibody GMTs were similar at one month post Dose 3 in both the RTS,S/AS01E (207.3 EU/mL [95% CI: 172.0 to 249.9]) and RTS,S/AS02D (183.1 EU/mL [95% CI: 150.6 to 222.5]) vaccine groups. Anti-HBs antibody GMTs in both vaccine groups post Dose 3 were high and similar (> 20 000 mIU/mL). Seroprotection for anti-HBs antibodies following both RTS,S/AS01E and RTS,S/AS02D vaccines was 100% post Dose 3.

Partial clinical data for the RTS,S/AS01E vaccine in infants aged 6 to 10 weeks at first dose are available for Malaria-050, a phase II randomized, open, controlled study of the safety and immunogenicity of RTS,S/AS01E, when incorporated into an Expanded Program on Immunization (EPI) regimen that includes DTPwHepB/Hib, OPV, measles and yellow fever vaccination in infants living in malaria-endemic regions on two schedules, either at 6, 10 and 14 weeks of age (012 schedule) or at 6, 10 weeks and 9 months of age (017 schedule) (where the third dose of RTS,S/AS01E was co-administered with measles vaccine). RTS,S/AS01E showed a satisfactory safety profile with no SAEs that were judged by the investigators to be related to vaccination. The overall profile of adverse events was compatible with morbidity patterns in sub-Saharan Africa. This study also analyzed the antibody responses to all EPI antigens. Pre-defined non-inferiority criteria were met for all antigens in the RTS,S/AS01E 012 and 017 schedules, except for the polio 3 responses in the RTS,S/AS01E 012 schedule. After receiving a neonatal dose of OPV followed by 3 doses given at the time of the DTPwHepB/Hib seroprotection rates for polio 3 were 87% in the group receiving RTS,S/AS01E in co-administration and 96% in the comparator group. A figure of 87% is consistent with seroprotection rates in the literature [WHO 1995, WHO 1997]. This unexpected finding will be further evaluated in this study. The consistency of this finding

will be assessed by measuring the polio serotypes 1, 2 and 3 responses induced by a primary series of OPV (4 doses commencing in the neonatal period) in a subset of children at all centers in the 6 to 12 weeks age category. All subjects in this age category will also receive a booster dose of OPV at study month 20 to assess the value of a booster dose when RTS,S/AS01E is given in co-administration with EPI antigens. The DSMB will review the analysis of polio 1, 2 and 3 titres and advice on the need to offer additional doses to children in the trial.

Amended 19 August 2008
Amended 24 October 2008

1.5. Rationale for the study

The study has been designed to address the key safety and efficacy information required for vaccine licensure. In addition, other disease endpoints that allow the evaluation of the full public health impact and cost effectiveness of vaccine implementation are included. Co-primary objectives will investigate efficacy against clinical disease in children from 5-17 months of age at first dose and the efficacy in infants 6-12 weeks of age who receive the vaccine in co-administration with EPI antigens (i.e. DTPwHepB/Hib; TritanrixHepB/Hib).

All participating centers will use standardized case definitions for efficacy endpoints and a structured approach to case-assessment. Cases of clinical disease will be pooled across participating centers to evaluate the primary endpoint of efficacy against clinical disease in each age category. Secondary objectives will assess the efficacy of the vaccine on severe malaria disease, severe anemia and malaria hospitalization. Analysis by site will allow the evaluation of efficacy under different conditions of malaria transmission and randomization to a booster dose will allow the evaluation of the duration of efficacy of a primary course and the requirement for boosting. Additional objectives include determination of efficacy of the vaccine against all medical hospitalization, non-malaria serious illness, fatal malaria and all-cause mortality. In addition immunological correlates of protection will be investigated.

Analyses are planned at the following timepoints during the trial:

- A primary analysis to investigate efficacy against clinical malaria disease after approximately 6000 children 5-17 months of age at first dose (4000 RTS,S/AS01E recipients) have completed 14 months of follow up post dose 1.
- A primary analysis to investigate efficacy against clinical malaria disease after approximately 6000 infants 6 to 12 weeks of age at first dose (4000 RTS,S/AS01E recipients) have completed 14 months of follow up post dose 1.
- A secondary analysis for evaluation of efficacy against severe disease when there are 250 accumulated cases, or at study end, whichever occurs earlier.
- A secondary analysis for evaluation of efficacy, safety and immunogenicity when all children have completed 18 month follow-up post Dose 3 (Visit 22).
- A secondary analysis for evaluation of the antibody responses to polio serotypes 1, 2 and 3 in the 6-12 weeks age category one month post boost (Visit 23).

Amended 19 August 2008
Amended 24 October 2008
Amended 23 January 2012

Further details of these planned analyses are provided in Sections 10.5.1, 10.5.2, 10.7.2, 10.7.3, 10.7.4 and *10.7.5.*

- A final analysis will be carried out at the end of the trial and will include further investigations of vaccine efficacy against a range of manifestations of malaria disease and evaluate the booster dose. Further details of the final analysis may be found in Sections 10.5.3 and 10.7.6.
- An additional analysis will be conducted at the end of the extension: including an evaluation of safety and efficacy against clinical malaria, severe malaria and prevalent parasitemia.

Amended 23 January 2012

1.5.1. Rationale for the choice of adjuvant

Safety, reactogenicity, immunogenicity and efficacy have previously been evaluated for RTS,S/AS02A and RTS,S/AS02D (refer to Section 1.4.1) and RTS,S/AS01B and RTS,S/AS01E (refer to Section 1.4.3).

Both adult formulations (RTS,S/AS02A and RTS,S/AS01B) and both pediatric formulations (RTS,S/AS02D and RTS,S/AS01E) have been shown to be equally reactogenic with a similar safety profile in their respective target age groups.

Compared to RTS,S/AS02A, RTS,S/AS01B has been shown to be substantially more immunogenic for the anti-CS antibody response in malaria-naive adults (Malaria-027) and adults in a malaria-endemic region (Malaria-044). CS-specific CD4+ T-cell responses induced by RTS,S/AS01B were higher in malaria-naïve adults (Malaria-027). In a bridging study in children in Gabon (Malaria-046) RTS,S/AS01E induced equivalent humoral immune responses compared to RTS,S/AS02D (with a trend for greater immune response in recipients of RTS,S/AS01E).

In the challenge trial in malaria naïve adults, there was a trend for efficacy to be higher with the RTS,S/AS01B formulation and both the humoral and cellular immune responses tended to be higher in the protected than the non-protected vaccinees. In the subsequent trial conducted in Kenya looking at protection against infection under natural transmission, efficacy of the two vaccine formulations was similar.

AS01 has been chosen as the adjuvant of this trial based on the data available to date which shows an equivalent safety and reactogenicity profile compared to AS02 and in most studies enhanced humoral and cellular responses. With regard to efficacy, currently the available information is protection against infection in the challenge model at WRAIR (Malaria-027) and natural challenge in adults in Kenya (Malaria-044). It is expected that in children the improved immunogenicity profile seen in adult studies, will translate into enhanced level of protective efficacy and duration of protection against clinical disease.

1.5.2. Rationale for evaluation of booster

In this study, some children will receive a booster dose of RTS,S/AS01E at Month 20.

Evidence that a booster dose may be necessary comes from the long-term follow up of children enrolled in the Malaria-026 study in Mozambique who received RTS,S/AS02A (0.25mL dose). In that study, VE was determined over a 33 month period commencing 14 days post Dose 3. While the vaccine retained efficacy in the period between Months 21 and 33 of the study, efficacy was lower than observed previously (Malaria Vaccine Investigator Brochure).

Amended 19 August 2008

The duration of efficacy associated with the RTS,S/AS01E formulation may be superior to that of RTS,S/AS02. In this study, randomization to a booster dose will allow definitive evaluation of the duration of efficacy of a primary course of the RTS,S/AS01E vaccine and the requirement for boosting.

1.5.3. Rationale for analysis by site

Field sites have been selected to represent the range of malaria transmission settings across sub-Saharan Africa including the intense seasonal transmission of West Africa and perennial transmission in East Africa. To determine efficacy under different conditions of malaria transmission, clinical malaria disease will be analyzed by site.

1.5.4. Rationale for the primary case definition of clinical malaria disease

The primary and secondary case definitions of clinical malaria disease are presented in Section 5.2.1.

The primary case definition reflects the burden of disease presenting to outpatient and inpatient departments across Africa and will have a minimum specificity of 80% at each site based on substantial previous research. Cases meeting the case definition of severe malaria disease will be included in the analysis of clinical malaria disease (whether or not they meet the case definition for clinical malaria disease as above).

The case detection methodology will be passive case detection at health facilities within the study area. In the participating centers High quality health care, to which all subjects will have access, will be available at all study centers ensuring that a high proportion of clinically significant cases will be captured by the surveillance system. This approach will capture cases representative of those presenting to outpatient and inpatient departments.

The challenge in developing a case definition is to achieve adequate specificity. This is because the symptomatology of malaria overlaps with that of common childhood infections. When a child presents with a febrile illness in an endemic country, it is often difficult on clinical grounds alone to know whether the malaria infection is the cause of the child’s illness or an incidental finding. A clinical diagnosis based on symptoms and a

blood slide reading is therefore sensitive but not specific. As parasite density increases, the likelihood that the clinical presentation is due to the *P. falciparum* infection increases. Smith first proposed methodology to calculate the specificity and sensitivity associated with threshold values of parasitemia in clinical case definitions [Smith, 1994]. This methodology has been adopted frequently for malaria research and has been evaluated across Africa and used in the evaluation of malaria interventions [Dicko, 2005; Greenwood, 1987; McGuinness, 1998; Mwangi, 2005; Saúte, 2003; Smith, 1994].

In children aged 1 to 5 years of age the parasite density thresholds for clinical case definitions, have been measured as 2500 in Kenya [Mwangi, 2005], 3200 in Mali [Dicko, 2005], 3500 Ghana [McGuinness, 1998], and 5000 in Gambia, Tanzania and Mozambique [Greenwood, 1987; Smith, 1994; Saúte, 2003]. Fewer estimates are available in children under the age of one year, but threshold values tend to be lower [Mwangi, 2005; Saúte, 2003].

A parasite density threshold of 5000 parasites/μL will be used for this study, because it will guarantee a minimum specificity in all transmission settings and age groups of 80%. The approach of applying one threshold value across age groups and sites is practical for pooling and interpretation of data.

The primary case definition has the requirement of the objective criterion of documented temperature ≥ 37.5°C; this is to further increase the specificity by reducing the number of cases of asymptomatic parasitemia that are captured by the definition.

We intend to evaluate as secondary case definitions 3 additional definitions. The first definition is highly sensitive allowing for the possibility of a history of fever as well objective fever in the presence of parasitemia >0. The second definition has a cut off for parasitemia of >500; the limited research done to date suggests that this will be adequately specific in children under the age of 1 year. The third definition is highly specific, requiring an objective fever and a parasitemia of >20 000.

The approach we present here is in full accord with the recommendation of the WHO consultation on measures of malaria vaccine efficacy [Moorthy, 2007].

1.5.5. Rationale for the primary case definition of severe malaria disease

The primary and secondary case definitions of severe malaria disease are presented in Section 5.2.2.

A case definition which reflects the picture of severe malaria disease that typically leads to hospitalization across Africa has been chosen. The analysis of the primary case definition will include cases occurring in children who are HIV infected or malnourished and the data collected will be highly relevant to policy makers.

The markers of severity of malaria disease will include those that:

- are associated with a poor outcome (mortality or sequelae)

- can be standardized across sites (with adequate training)
- minimize redundancy.

Other markers of severity were considered, but not included because:

- they apply only to a small fraction of the number of cases
- methodology is difficult to implement and standardize across sites
- they are not part of accepted practice or research methodology.

As described in 1.5.4, a parasite density threshold has been implemented to improve the specificity of the primary case definition by reducing the number of cases of co-morbidity.

In children admitted to hospital in Africa, concurrent co-morbid infections are frequent. It is not clear whether the parasitemia is co-incidental and does not contribute to the clinical picture, whether this is the chance concurrence of two common childhood diseases, or whether there is a definite synergy in the etiology of the two diseases. This is complicated to disentangle, because the symptomatology of the common infections in a seriously sick child is broadly overlapping [O'Dempsey, 1993; Mulholland, 2005]. What would be the effect of the vaccine on serious illnesses, where malaria is a co-infection is unpredictable. Therefore to ensure high specificity of the primary case definition and a clearly interpretable result, other important co-morbidity will be actively sought for and excluded. Therefore in the evaluation of a child hospitalized for an acute medical condition, an algorithm will be followed that ensures that the disease picture is well-characterised and the appropriate diagnostic tests are taken (including blood and cerebro-spinal fluid [CSF] culture and chest X-rays [CXR]). The intention is to exclude cases of pneumonia, meningitis, sepsis and gastroenteritis with severe dehydration. Whilst excluding co-morbidity ensures that the primary case definition is maximally specific, in public health terms whether the vaccine also has an effect on severe disease with concurrent malaria infection and other morbidity is very important. Therefore a secondary case definition that examines the effect of the vaccine on severe malaria disease with co-morbidity included will be examined (refer to Table 8).

One of the greatest challenges in developing this definition is to distinguish between severe gastroenteritis and malaria as a cause of prostration. The mechanism underlying the prostration is thought to be fundamentally different in the two diseases: in malaria it is primarily intravascular volume depletion, whereas in gastroenteritis it is predominantly intracellular fluid depletion. The WHO definition of 3 or more loose or watery stools in 24 hours defines gastroenteritis of any severity. However, only severe gastroenteritis associated with severe dehydration (≥ 10% loss of body weight) causes prostration, and therefore criteria characterizing the severity have been added to the definition. The Integrated Management of Childhood Illness (IMCI) approach defines severe dehydration as diarrhea plus two or more of: lethargy / altered consciousness (=prostration), sunken eyes, skin pinch > 2 seconds, unable to drink/drinks poorly [WHO, 2001]. However, a recent review found that the best signs of severe dehydration were prolonged capillary refill time, reduced skin turgor, abnormal respiratory pattern (tachypnea and deep/acidotic breathing) [Steiner, 2004]. Of these three signs only an assessment of skin

turgor discriminates between intravascular and intracellular volume depletion. The case definition therefore includes a decrease in skin turgor with the WHO definition of gastroenteritis in order to identify and exclude from the case definition of severe malaria cases of prostration due to severe gastroenteritis.

In order to ensure harmonization of case assessment across sites, physicians will follow a standard algorithm for case assessment (Appendix E) and there will be training, monitoring and evaluation of performance at the start and during the trial. A full discussion of the merits of this case definition is made in the WHO consultation report [Moorthy, 2007].

1.5.6. Rationale for the methodology of analysis of clinical malaria endpoint

The incidence of episodes of clinical malaria up to 12 months follow-up will be analyzed separately for the two age categories (6-12 weeks and 5-17 months at first vaccination).

As the primary analysis, first or only episodes of clinical malaria over a 12 months follow-up period for all subjects will be evaluated. Episodes after the first will not be included in the primary analysis and subjects will be censored at their first or only episode, consent withdrawal, death or end of follow-up period, whichever occurs first.

As an analysis method for evaluating first or only episodes a Cox regression model will be used, modeling the time to first or only episode as a function of group assignment, and study site as a covariate. Vaccine efficacy (VE) will be defined as 1 minus Hazard Rate (HR). 95% Confidence intervals (CI) and p-values (likelihood Ratio test) on VE estimates will be calculated from the Cox model. VE estimates resulting from HR are appropriate as it is likely the vaccine protects by reducing the risk of disease following an infectious bite by a fixed proportion. Cox regression allows for seasonal variation of malaria incidence and adding study site as a covariate allows for different levels of baseline risk and different patterns of seasonality. The primary analysis will be unadjusted for other covariates; however both unadjusted and adjusted estimates of VE will be presented. As a secondary analysis, estimates of VE including multiple episodes will also be calculated.

1.5.7. Rationale for sample size

This is the definitive Phase 3 trial of the RTS,S/AS01E that will support the licensure application to the European Medicines Agency (EMEA) and African national regulatory authorities. It is designed with conservative estimates of malaria disease rates to deliver reliably the primary endpoint of efficacy against clinical malaria disease over one year in each of the two age categories. In the event that the rate of disease is higher than predicted, then the estimate of effect will be delivered with greater precision.

The secondary endpoints of this trial are also important for regulators and health policy decision makers; this sample size will allow the vaccine effect on severe malaria disease and malaria hospitalization, and will define vaccine efficacy under different malaria transmission patterns and booster strategy. In addition, a safety database of this size is

required as this is the first time that this novel adjuvant has been presented to regulatory authorities in a pediatric vaccine for wide spread implementation.

1.5.8. Rationale for the methodology of analysis of severe malaria endpoint

In a secondary analysis, VE against severe malaria disease will first be evaluated when 250 subjects (both age categories together) have reported an episode of severe malaria meeting the primary case definition. The primary analysis will compare the pooled RTS,S/AS01E groups (with or without RTS,S/AS01E booster dose) versus control. As severe malaria disease is relatively rare and most subjects are unlikely to experience more than 1 episode over the course of the trial, primary analysis will evaluate the proportion of subjects affected by severe malaria disease. VE against severe malaria disease will be estimated as 1-RR where RR is the risk ratio (proportion of subjects reporting severe malaria disease in the RTS,S/AS01E group over the proportion in controls) together with 95% CIs. The primary analysis will be unadjusted for covariates. Secondary analyses adjusted for covariates and analysis of multiple episodes of severe malaria disease will also be performed.

1.5.9. Rationale for choice of cohort for primary analysis

As the trial primarily generates data to support registration of a malaria vaccine given as 3 doses within specified time intervals, in eligible children, ATP analyses will be considered primary. However, both ITT and ATP analyses will be performed.

1.5.10. Choice of comparator vaccines

The choice of comparator vaccine is guided by the principles of benefit to the control group without compromising the evaluation of clinical study endpoints.

1.5.10.1. Rationale for the use of Meningococcal C conjugate vaccine as a control

Children aged 6-12 weeks in the control group will receive 3 doses of a Meningococcal C Conjugate Vaccine (MCC) on a 0, 1, 2-month schedule followed by a booster dose of MCC at Month 20.

A number of alternative comparator vaccines were considered for use in the 6 to 12 week age group in this study but were deemed not to be suitable. One key consideration in this age group was that it is necessary to have a vaccine that can safely be co-administered with EPI antigens; for this reason rabies vaccine, the control vaccine for the older age category, was not selected. Also considered were vaccines against pneumococcal infection, which is a common cause of pneumonia children in Africa. However, for reasons that are not clear malaria and pneumococcal pneumonia often occur together in African children [O'Dempsey 1993; Mulholland, 2005]. If malaria infection contributes to the pathogenicity of pneumococcal disease and other common infections, then controlling malaria may have additional benefits beyond the effect on malaria disease alone. A suggestion that this is the case was seen in studies of bednets, which showed

non-specific beneficial affects on childhood morbidity and mortality. Understanding the full potential impact of this vaccine on malaria and other morbidities will be an asset for public health authorities evaluating its usefulness.

MCC was chosen because it is acceptably safe and immunogenic when given on a 0, 1, 2-month schedule, can be coadministered with DTPw Hep B/Hib + OPV and does not compromise the evaluation of study endpoints. Although meningitis C disease is not common in sub-Saharan Africa, there have been outbreaks of meningococcal C disease in the 1970s and early 1980s in Nigeria [Whittle, 1975] and Burkina Faso [Broome, 1983]. A proportion of cases seen in outbreaks in Nigeria and Ethiopia in the 1980s were also due to serogroup C and some serogroup C cases have been described from The Gambia and Mozambique [Kwara, 1998]. The MCC vaccine will therefore be of benefit to the participants in the event of an outbreak.

While a 2-dose plus boost regimen is now in place for MCC in the UK, the safety of a 3-dose co-administered regimen in infancy is well-established, having been implemented as national policy between 1999 and 2005. During this period, all three MCCs proposed for use in this study (Menjugate [Novartis], NeisVac-C [Baxter] and Meningitec [Wyeth]) were used in the UK [Miller, 2002]. A booster in the second year of life has been shown to be necessary when children receive primary immunization in early life [Miller, 2002]. All children who receive a primary course of MCC will receive a booster dose.

Children in the 6-12 week age category who received a primary course of RTS,S/AS01E may be randomized to receive a dose of MCC at time of boost. Similarly, children in the 5-17 month category may be randomized to receive a dose of MCC at time of boost. A single dose of MCC in the second year of life (or later) is also an immunizing regimen.

To ensure vaccine availability, MCC from three manufacturers will be sourced for this trial (Novartis, Baxter and Wyeth). It will be ensured that an individual child will receive all 3 doses of MCC from the same manufacturer.

Product data from Wyeth on Meningitec indicates that it may be coadministered with Hepatitis B vaccine. However, one trial of GSK's DTPwHepB/Hib vaccine (Tritanrix HepB™/Hib) showed a trend towards lower anti-HBs antigen seroprotection rate when the vaccine was coadministered with Meningitec in children in the Philippines (refer to Table 4). There is no product data to support the co-administration of Menjugate [Novartis] and NeisVac-C [Baxter] with hepatitis B antigen. Therefore for all three MCC vaccines the anti-HBs antibody response will be monitored and a rescue plan defined in the study protocol (refer to Section 5.1.4.4).

Table 4 Immunogenicity of Hepatitis B antigens in coadministration with DTPw Hib and MCC

Group	Seroprotection %	95% CI		GMT	95% CI	
		LL	UL		LL	UL
TritanrixHepB + Hiberix	91.6	84.1	96.3	104.5	76.0	143.7
TritanrixHepB/Hib + Meningitec	83.2	74.4	89.9	71.1	52.1	97.1

1.5.10.2. Rationale for the use of rabies vaccine as a control

Children aged 5-17 months randomized to receive control vaccine will receive 3 doses of a cell-culture rabies vaccine on a 0, 1, 2-month schedule.

Without vaccination or post exposure treatment, infection with rabies invariably leads to death. Children are a group at particular risk; rabies is most common in people under the age of 15 years, and approximately 40% of all cases occur in children aged between 5 and 14 years [Plotkin, 2004].

Estimates of risks of infection with rabies in Africa are available from the WHO. The WHO no longer publish the ‘World survey of rabies’ (the last edition was 1998), but data is available through Rabnet (http://www.who.int/globalatlas/default.asp). A recent paper evaluating the burden of rabies in Africa estimated that 294 million people in Africa are at risk of exposure to rabies, with models indicating that 23 000 people a year in Africa die as a result of infection [Knobel, 2005].

Rabies vaccine has been evaluated on a number of different vaccination schedules. According to product information distributed by Sanofi Pasteur Inc, PA, USA in December 2005 for their Human Diploid Cell Rabies Vaccine, “high titer antibody responses of the Sanofi Pasteur SA Rabies Vaccine made in human diploid cells have been demonstrated in trials conducted in England [Aoki 1975], Germany [Cox, 1976; Kuwert, 1978], France [Ajjan, 1978] and Belgium [Costy-Berger, 1978]. Seroconversion was often obtained with only one dose. With two doses one month apart, 100% of the recipients developed specific antibody and the geometric mean titer of the group was approximately 10 international units. In the US, Sanofi Pasteur SA Rabies Vaccine resulted in geometric mean titers (GMT) of 12.9 IU/mL at Day 49 and 5.1 IU/mL at Day 90 when three doses were given intramuscularly during the course of one month. The range of antibody responses was 2.8 to 55.0 IU/mL at Day 49 and 1.8 to 12.4 IU at Day 90 [Bernard 1982]. The definition of a minimally accepted antibody titer varies among laboratories and is influenced by the type of test conducted. CDC currently specifies a 1:5 titer (complete inhibition) by the rapid fluorescent focus inhibition test (RFFIT) as acceptable. The World Health Organization (WHO) specifies a titer of 0.5 IU”.

To ensure vaccine availability, three cell culture rabies vaccines from two manufacturers will be sourced for this trial (Sanofi-Pasteur and Novartis). While the cell-culture sources of the vaccines differ, they are largely equivalent in terms of safety profile,

immunogenicity and efficacy [Plotkin, 2004]. It will be ensured that an individual child will receive all 3 doses of cell culture rabies vaccine from the same product.

Amended 24 October 2008

1.6. Rationale for the extension

The strategy underlying the development of the RTS,S/AS01E malaria vaccine is that it would target the most vulnerable age group: infants and young children. It would provide protection from the most severe forms of the disease whilst allowing natural blood stage immunity to develop due to continued exposure to infections. However, there is a theoretical concern that the protection conferred by a malaria vaccine in young children may impair their ability to acquire natural immunity to the blood stage form of *P. falciparum* and that, if the vaccine efficacy decreases over time, these children would become vulnerable to malaria. This could result in a higher susceptibility to severe malaria and/or a higher frequency of clinical malaria disease in vaccinated children compared to children of the same age that were not vaccinated. This long term follow-up probably represents the last opportunity to evaluate the duration of vaccine efficacy and the long term safety of RTS,S/AS01E relative to a control group in a large multi-country study.

The design of the extension study takes advantage of the fact that enrolment to the primary trial phase will take approximately 18 months to complete and continues surveillance until December 2013 (corresponding to last subject last visit - estimated December 2013). Importantly this extension does not follow up subjects beyond the point of the final analysis. However the extension is described as single blind because cleaning of the data may take place after unblinding.

The extension will collect data on severe malaria disease; malaria hospitalization and parasite prevalence in the 11 participating centres using the same methodologies and case definitions as in the primary trial phase. Occurrence of SAEs will be monitored in all 11 centres. Surveillance for clinical malaria disease will take place at least at 3 centres with varying transmission levels. Immunogenicity endpoints will also be collected on a subset of individuals from both age categories at least in these 3 centres.

Mean follow-up time: The last study visit of the extension (Visit 38) is scheduled in December 2013 (interval: Nov 2013-Jan 2014). This means a variable number of months after vaccination by individual children. Based on the actual enrolment, the mean follow-up time will be:

- 5-17 months: 49 months post Dose 1 (range: 41-55)
- 6-12 weeks: 41 months post Dose 1 (range: 32-48)

Amended 01 December 2010

2. OBJECTIVES

2.1. Primary objectives

2.1.1. Efficacy coprimary objectives: Efficacy against clinical malaria disease when primary immunization starts at 6-12 weeks, or 5-17 months of age

- To evaluate the protective efficacy of RTS,S/AS01E against clinical malaria disease caused by *Plasmodium falciparum* in African children whose age at first dose will be 6-12 weeks and will receive vaccine in coadministration with DTPwHepB/Hib antigens (Tritanrix HepB/Hib) and OPV. Duration of follow up will be for a minimum of 12 months and a maximum of 18 months after completion of the primary course (Primary Analysis).
- To evaluate the protective efficacy of RTS,S/AS01E against clinical malaria disease caused by *Plasmodium falciparum* in African children whose age at first dose will be from 5-17 months. Duration of follow up will be for a minimum of 12 months and a maximum of 18 months after completion of the primary course (Primary Analysis).

Amended 19 August 2008

Refer to Section 10.1 for definition of the primary endpoints.

Refer to Section 5.2 for case definitions for efficacy endpoints.

2.2. Secondary objectives

2.2.1. Efficacy secondary objectives

2.2.1.1. Efficacy against severe malaria disease

- To evaluate the protective efficacy of RTS,S/AS01E on a primary schedule with and without booster dose against severe malaria disease. Duration of follow up will extend to 30 months after completion of primary course.

2.2.1.2. Efficacy against incident severe anemia and malaria hospitalization

- To evaluate the protective efficacy of RTS,S/AS01E on a primary schedule with and without booster dose against incident severe anemia and malaria hospitalization. Duration of follow up will extend to 30 months after completion of primary course.

2.2.1.3. Efficacy; duration of efficacy of a primary course

- To evaluate the duration of protective efficacy of a primary schedule of RTS,S/AS01E with no booster dose against clinical malaria disease. Duration of follow up will extend to 30 months after completion of primary course.

2.2.1.4. Efficacy; the role of a booster

- To evaluate the protective efficacy of RTS,S/AS01E against clinical malaria disease with and without a booster dose given 18 months after completion of a primary schedule. Duration of follow up will be for 12 months after a booster dose.

2.2.1.5. Efficacy under different transmission settings

- To evaluate the protective efficacy of RTS,S/AS01E on a primary schedule with and without booster dose against clinical malaria disease under different conditions of malaria transmission. Field site will be used as a proxy for transmission setting. Duration of follow up will extend to 30 months after completion of primary course.

2.2.1.6. Efficacy against secondary case definitions of clinical malaria disease

- To evaluate the protective efficacy of RTS,S/AS01E on a primary schedule with and without booster dose against secondary case definitions of clinical malaria disease. Duration of follow up will extend to 30 months after completion of primary course.

2.2.1.7. Efficacy against prevalence of parasitemia

- To evaluate the protective efficacy of RTS,S/AS01E against prevalence of parasitemia 18 and 30 months after a primary schedule and 12 months after a booster dose.

2.2.1.8. Efficacy against prevalence of moderate and severe anemia

- To evaluate the protective efficacy of RTS,S/AS01E against prevalence of moderate and severe anemia 18 and 30 months after a primary schedule and 12 months after a booster dose.

Refer to Section 5.2 for case definitions for efficacy endpoints.

2.2.2. Safety secondary objectives

2.2.2.1. Safety of a primary course

- For each age category, to evaluate the safety of a primary schedule of RTS,S/AS01E in African children. Duration of follow up will be for 14 months after receipt of the first dose (Primary Analysis).
- For each age category, to evaluate the safety of a primary schedule of RTS,S/AS01E without a boost. Duration of follow up will be for 32 months after receipt of first dose.

2.2.2.2. Safety of a booster dose

- For each age category, to evaluate the safety of a booster dose of RTS,S/AS01E administered 18 months after completion of primary course. Duration of follow up will be for 12 months after receipt of boost.

2.2.3. Immunogenicity secondary objectives

2.2.3.1. Immunogenicity of a primary course

- For children aged 5-17 months at first vaccination, to evaluate the anti-CS and anti-HBs immunogenicity of a primary schedule of RTS,S/AS01E without a boost. Duration of follow up will be for 32 months after receipt of first dose.
- For children aged 6-12 weeks at first vaccination, to evaluate the anti-CS and anti-HBs immunogenicity of a primary schedule of RTS,S/AS01E without a boost when coadministered with DTPwHepB/Hib (Tritanrix HepB/Hib). Duration of follow up will be for 32 months after receipt of first dose.

2.2.3.2. Immunogenicity of a booster dose

- For children aged 5-17 months at first vaccination, to evaluate the anti-CS and anti-HBs immunogenicity of a booster dose of RTS,S/AS01E administered 18 months after completion of primary course. Duration of follow up will be for 12 months after receipt of boost.
- For children aged 6-12 weeks at first vaccination, to evaluate the anti-CS and anti-HBs immunogenicity of a booster dose of RTS,S/AS01E administered 18 months after completion of primary course of RTS,S/AS01E coadministered with DTPwHepB/Hib (Tritanrix HepB/Hib). Duration of follow up will be for 12 months after receipt of boost.

2.2.3.3. Antibodies to polio serotypes 1, 2 and 3

- To describe antibody responses to polio serotypes 1, 2 and 3 when OPV is given on a primary schedule co-administered with DTPwHepB/Hib (Tritanrix HepB/Hib) with or without RTS,S/AS01E at one month post dose 3.
- To describe antibody responses to polio serotypes 1, 2 and 3 when OPV is given on a primary schedule (co-administered with DTPwHepB/Hib with or without RTS,S/AS01E) and a booster dose at 18 months post primary (with or without RTS,S/AS01E) at one month post boost.

Amended 19 August 2008
Amended 24 October 2008

Refer to Section 10.2 for definitions of secondary endpoints.

2.3. Additional Secondary Objectives

2.3.1. Additional efficacy secondary objectives

Amended 19 August 2008

2.3.1.1. Efficacy against other serious illness

- To evaluate the protective efficacy of RTS,S/AS01E against other serious illness when given on a primary schedule with or without boost. Other serious illness is all-cause hospitalization, sepsis and pneumonia. Duration of follow-up will extend to 30 months after completion of primary course.

Refer to Section 5.2 for case definitions for efficacy endpoints.

2.3.1.2. Efficacy against fatal malaria and all-cause mortality

- To evaluate the protective efficacy of RTS,S/AS01E against fatal malaria and all-cause mortality when given on a primary schedule with or without boost. Duration of follow up will extend to 30 months after completion of primary course.

2.3.1.3. Effect on growth

- To evaluate the effect of RTS,S/AS01E when given on a primary schedule with and without a booster dose on growth.

2.3.1.4. Gender-specific efficacy

- To evaluate the vaccine efficacy of RTS,S/AS01E in male and female children when given on a primary schedule with and without a boost.

2.3.2. Additional immunogenicity secondary objectives

Amended 19 August 2008

2.3.2.1. Immunological correlates of protection

- To evaluate the association between CS-antibody response and protection against malaria disease.

2.3.3. Additional safety and immunogenicity secondary objectives

Amended 19 August 2008

2.3.3.1. Safety and immunogenicity in ‘special’ subgroups

- To evaluate safety and immunogenicity of a primary course or a booster dose of RTS,S/AS01E in low weight for age (i.e. z-score ≤ -2) and very low weight for age (i.e. z-score ≤ -3) children.
- To evaluate safety and immunogenicity of a primary course or a booster dose of RTS,S/AS01E in HIV-infected children.
    - *Note: HIV testing is not a trial procedure. This analysis will include all children known to be HIV-infected at enrollment or subsequently diagnosed.*

Refer to Section 10.3 for definitions of these additional secondary endpoints.

Amended 19 August 2008

2.4. Secondary objectives for the extension

2.4.1. Efficacy

In children from 6 weeks to 17 months of age at primary immunisation for the period 14 days post Dose 3 to the end of extension:

- For each of the RTS,S/AS01E immunisation regimens primary alone or primary plus boost, to evaluate the protective efficacy against clinical malaria disease caused by *Plasmodium falciparum* in each of at least 3 transmission settings.
- For each of the RTS,S/AS01E immunisation regimens primary alone or primary plus boost, to evaluate the total cases of clinical malaria disease caused by *Plasmodium falciparum* averted in each of at least 3 transmission settings.
- To evaluate the protective efficacy of RTS,S/AS01E (pooled immunisation regimens) against severe malaria disease.
- To evaluate the total cases of severe malaria disease averted by RTS,S/AS01E (pooled immunisation regimens).
- To evaluate the protective efficacy of RTS,S/AS01E (pooled immunisation regimens) against malaria hospitalization.
- To evaluate the total cases of malaria hospitalization averted by RTS,S/AS01E (pooled immunisation regimens).

In children from 6 weeks to 17 months of age at primary immunisation, at annual timepoints during the extension:

- To evaluate the protective efficacy of RTS,S/AS01E against prevalence of parasitemia.
- To evaluate the protective efficacy of RTS,S/AS01E against prevalence of moderate and severe anemia.

In children from 6 weeks to 17 months of age at primary immunisation for the period 14 days post Dose 3 to the end of extension:

- To evaluate the protective efficacy of RTS,S/AS01E (pooled immunisation regimens) against all-cause mortality and fatal malaria.
- To evaluate the total cases of all-cause mortality and fatal malaria averted by RTS,S/AS01E (pooled immunisation regimens).
- To evaluate the protective efficacy of RTS,S/AS01E (pooled immunisation regimens) against all medical hospitalization.
- To evaluate the total cases of all medical hospitalization averted by RTS,S/AS01E (pooled immunisation regimens).
- To evaluate the protective efficacy of RTS,S/AS01E (pooled immunisation regimens) against pneumonia.

- To evaluate the total cases of pneumonia averted by RTS,S/AS01E (pooled immunisation regimens).
- To evaluate the protective efficacy of RTS,S/AS01E (pooled immunisation regimens) against sepsis.
- To evaluate the total cases of sepsis averted by RTS,S/AS01E (pooled immunisation regimens).

2.4.2. Safety

In children from 6 weeks to 17 months of age at primary immunisation for the time period from study start to the end of extension:

- To evaluate the safety of the RTS,S/AS01E vaccine.

2.4.3. Immunogenicity

- To analyze the anti-CS antibodies titers at annual timepoints during the extension.

2.4.4. Effect on growth

In children from 6 weeks to 17 months of age at primary immunisation:

- To evaluate the effect of RTS,S/AS01E on growth at the end of extension.

Amended 01 December 2010

3. STUDY DESIGN OVERVIEW

Figure 1 Study design

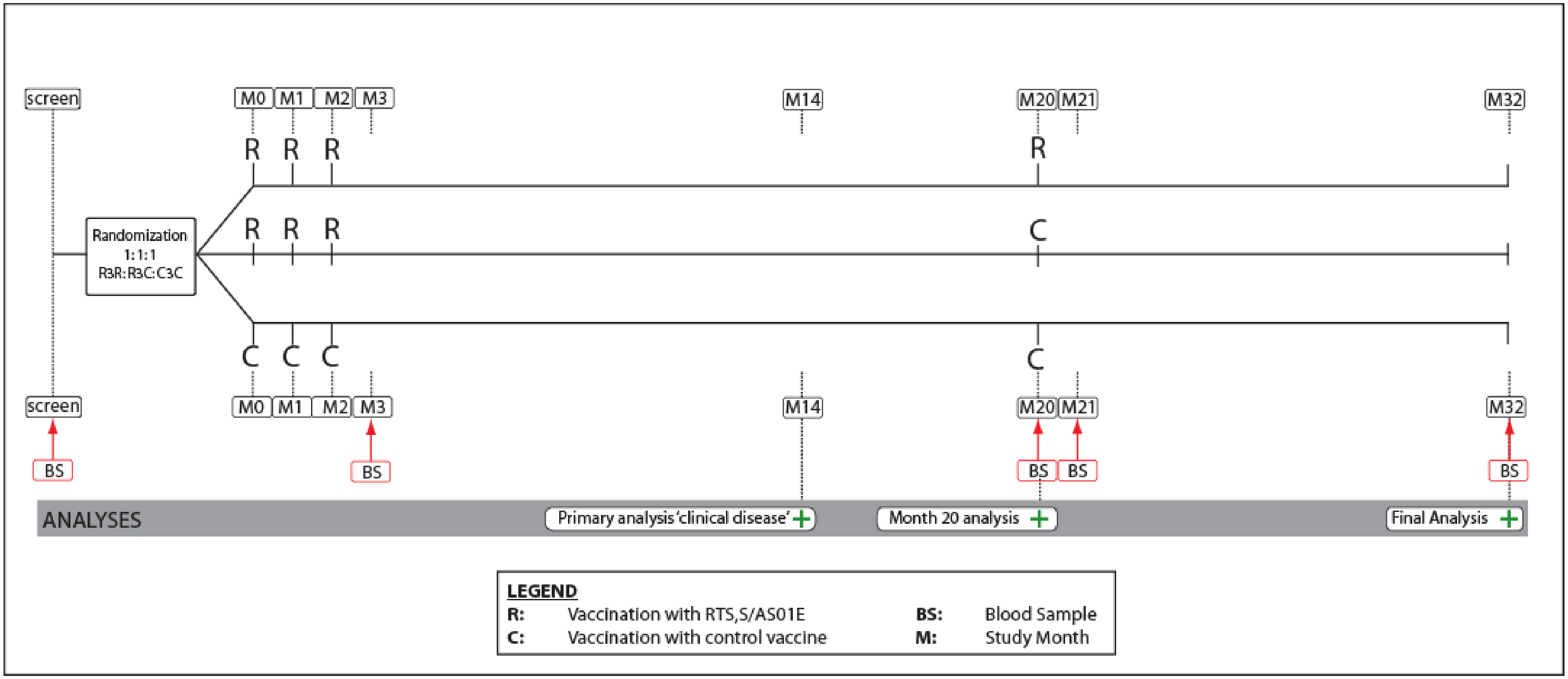

Amended 23 January 2012

Figure 2 Study design of the extension

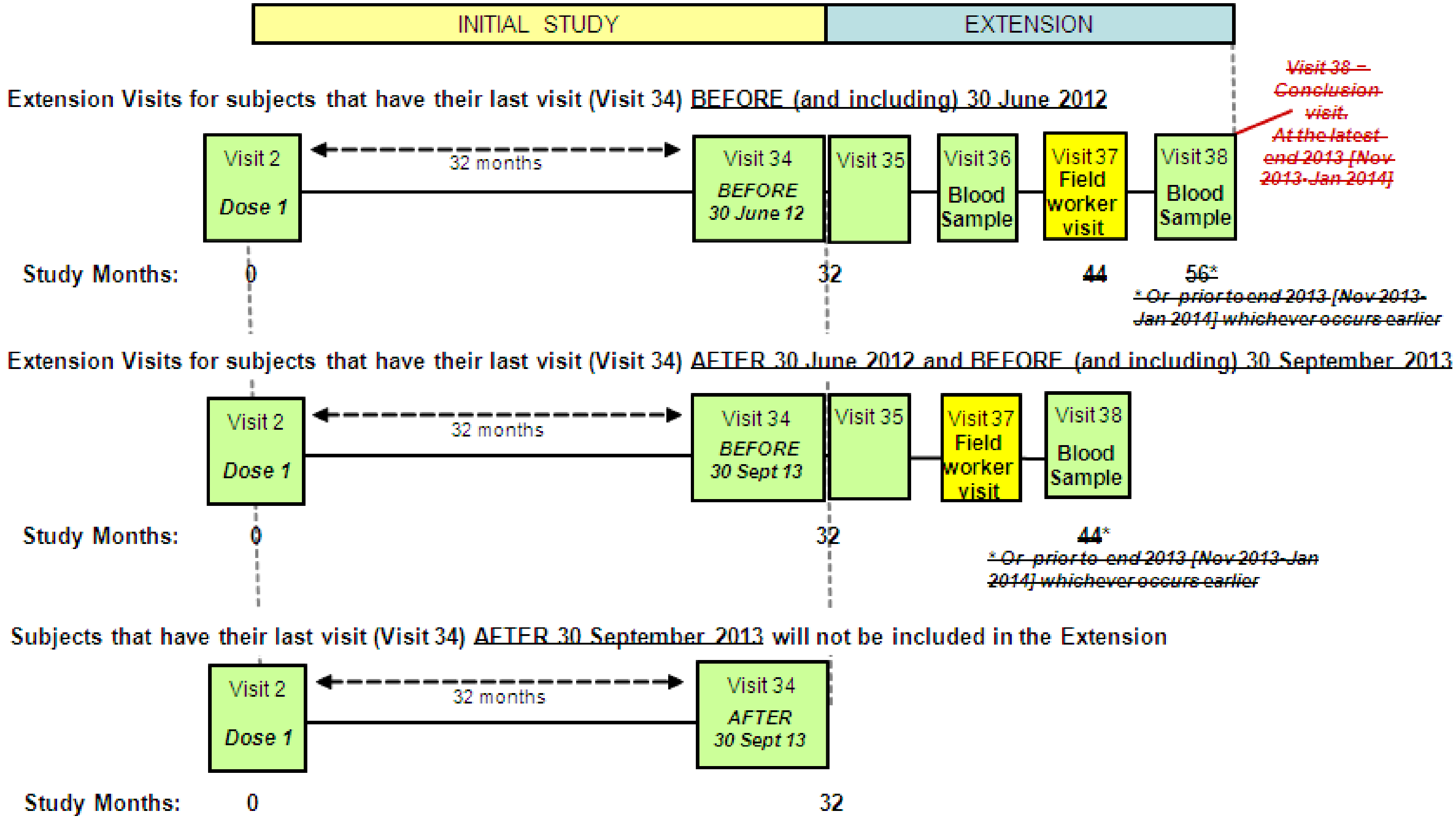

Amended 01 December 2010
Amended 08 August 2012

Study design Amended 19 August 2008
Study design Amended 24 October 2008
Study design Amended 26 November 2009
Study design Amended 01 December 2010

3.1. Study design

- Experimental design: Phase III, multi center, double blind (observer-blind), randomized, controlled trial with three groups in each of two age categories.
- Males and females enrolled in two age categories: children aged 6-12 weeks and children aged 5-17 months. Children must be > 28 days of age at screening. Those determined to be eligible based on the inclusion and exclusion criteria will be enrolled in the study.
- A maximum of 16 000 children (a *minimum* of 6000 in each age category) will be randomized.
- Treatment Groups and study vaccination schedule
children 5-17 months of age:

Primary Vaccination on a 0, 1, 2 month schedule	Boost at M 20
RTS,S/AS01E	RTS,S/AS01E
RTS,S/AS01E	MCC Vaccine*
Rabies Vaccine** †	MCC Vaccine*

*Meningococcal C Conjugate vaccine: Meningitec (Wyeth), NeisVac-C (Baxter) or Menjugate (Novartis).
**Cell culture rabies vaccine: Human Diploid Cell Rabies Vaccine (Sanofi Pasteur) or Purified Chick Embryo Cell Culture Vaccine (Rabipur or equivalent) (Novartis) or Purified Vero cell culture rabies vaccine (VeroRab or equivalent) (Sanofi Pasteur).
† each child will receive all 3 doses of rabies vaccine from the same manufacturer

- Treatment Groups and vaccination schedule
children 6-12 weeks of age:

Primary Vaccination on a 0, 1, 2 month schedule	Boost at M 20
RTS,S/AS01E + Tritanrix HepB/Hib** + OPV	RTS,S/AS01E + OPV
RTS,S/AS01E + Tritanrix HepB/Hib** + OPV	MCC vaccine* + OPV
MCC vaccine * † + Tritanrix HepB/Hib** + OPV	MCC vaccine* + OPV

*Meningococcal C Conjugate vaccine: Meningitec (Wyeth), NeisVac-C (Baxter) or Menjugate (Novartis)
OPV: Oral Polio Vaccine (Polio Sabin [GSK])
† each child will receive all 3 doses of MCC from the same manufacturer.
**** DTPwHepB/Hib

- **Route of administration:**
  - Children 5-17 months of age at enrollment will receive primary immunizations intramuscularly into the left deltoid.
  - Children 6-12 weeks of age at enrollment will receive primary immunizations intramuscularly into the anterolateral thigh
(Left: RTS,S/AS01E and Meningococcal C conjugate vaccine; Right: Tritanrix HepB/Hib).

NOTE: OPV will be administered orally.

- Route of administration for the booster dose comparator will be into the left deltoid for ALL children.

- BCG vaccine, neonatal dose of OPV, measles and yellow fever vaccines will be given according to local EPI policy.
- There will be no routine testing for HIV in this trial. Tests will be performed when clinically indicated to guide subject management. Voluntary counseling and testing, Highly Active Anti-Retroviral Therapy (HAART) and Prevention of Mother to Child Transmission (PMCT) are available at all study centers according to national policies.
- The research teams at each study center will ensure that insecticide treated bednet use is optimized in each study population.
- **Surveillance for Efficacy:**
  - All subjects presenting to health facilities in the study area will be evaluated as potential cases of clinical malaria disease. A blood sample will be taken for evaluation of malaria parasites in all children who are reported to have had a fever within 24 hours of presentation or have a measured axillary temperature of ≥ 37.5°C.
  - All subjects presenting for admission through the outpatient and emergency departments of hospitals in the study areas will be evaluated as potential cases of severe malaria disease following a protocol-defined algorithm.
  - During any hospitalization, the subject's course will be monitored to capture the signs and blood parameters indicative of severe malaria disease. If the subject's condition changes from admission and he/she meets one of the criteria for additional investigation, these will be performed.
  - Harmonization of case evaluation across centers will be assured by training of clinicians in the assessment of clinical signs and the standardization of equipment and processes used for laboratory investigations.
- **Surveillance for Safety:**

*(*n *assumes 6000 enrolled, of which 90% will complete primary immunization and 85% complete booster dose and 10 participating sites).*

  - SAEs will be collected for the duration of the study from all subjects (*to have a minimum of 3620 recipients of a primary course of RTS,S/AS01E and 1680 recipients of a booster dose in each age category*).
  - Unsolicited AEs (defined as those AEs judged to be related to vaccination, or leading to drop-out) will be collected for 1 month post each dose in all subjects (*to have a minimum of 3620 recipients of a primary course of RTS,S/AS01E and 1680 recipients of a booster dose in each age category*).
  - Unsolicited AEs (all) will be collected for 1 month following each vaccine dose for the first 200 subjects in each age category enrolled at each center (to have approximately 1200 recipients of a primary course of RTS,S/AS01E and

560 recipients of a booster dose in each age category).

- Solicited AEs will be collected for one week following each vaccine dose in the first 200 subjects in each age category enrolled at each center (*to have approximately 1200 recipients of a primary course of RTS,S/AS01E and 560 recipients of a booster dose in each age category*).
- Verbal autopsy will be performed on all cases of mortality occurring outside hospital.

- **Surveillance for Immunogenicity:**

*(*n *assumes 6000 enrolled, of which 90% will complete primary immunization and 85% complete booster dose and 10 participating sites)*

- All children will be sampled for anti-HBs and anti-CS testing.
- Immunogenicity of a primary course will be evaluated pre vaccination, 1 month post primary course and 18 months post primary course in the first 200 subjects in each age category enrolled at each center (*to have approximately 1200 recipients of a primary course of RTS,S/AS01E in each age category*).
- Duration of immunogenicity of a primary course will be evaluated 18 and 30 months post primary course in the first 200 subjects in each age category enrolled at each center (*to have approximately 560 recipients of a primary course of RTS,S/AS01E in each age category*).
- Immunogenicity of a booster dose will be evaluated pre-boost vaccination, 1 month post boost and 12 months post boost in the first 200 subjects in each age category enrolled at each center (*to have approximately 560 recipients of a primary course of RTS,S/AS01E in each age category*).
- Immune correlates of protection will be explored by sampling all subjects 1 month post primary course and 1 month post boost and the full details of the analysis will be presented in the SAP to compare subjects affected and unaffected by malaria disease.
- Surveillance for Cellular Immunology: At some study centers, subjects may be sampled for evaluation of the cellular immune response induced by the RTS,S/AS01E vaccine. CMI procedures will be detailed in separate protocol, ancillary to this study.
- Immunogenicity of the polio serotypes 1, 2 and 3 will be evaluated when OPV is given on a primary schedule with DTPwHepB/Hib (Tritanrix HepB/Hib) with or without RTS,S/AS01E at screening, at 1 month post primary course (Visit 5) and following a booster dose at 18 months post primary (with or without RTS,S/AS01E) at 1 month post boost (Visit 23) in a subset of subjects in the 6-12 weeks age category enrolled at each center.

- Data collection: where technically possible, this study will use electronic case report form (eCRF) by remote data entry (RDE) in preference to conventional paper case report form (CRF).
- Subjects will be randomized using an internet-based system.

- Duration of the study: the primary study will continue for 32 months per child, plus a screening period of 1 month.
- Analysis of co-primary endpoint: clinical disease
  - The analysis of efficacy of a primary schedule of RTS,S/AS01E will be carried out after the first 14 months of follow-up have been completed for the first 6000 subjects (approximately) enrolled in the age category under evaluation.
  - It is anticipated that enrolment of the 5-17 months age category will be faster than in the infant age category and thus analysis of the two co-primary efficacy endpoints may take place at different times. In order to control the overall alpha-level (5%) of the trial both evaluations will be performed at a 2.5% alpha level (Bonferroni correction) thus, for the evaluation of the co-primary endpoint leading to 97.5% Confidence Intervals (CI).
  - In both age categories, efficacy against clinical malaria of the primary immunization schedule will be measured when at least 6000 subjects have 14 months of follow-up. Assuming at least 5400 evaluable subjects (randomized 2:1), an attack rate in controls of 10/100 cyr (children years at risk) over the follow-up period and a true vaccine efficacy of 30%, the study has 90% power to detect a lower limit of the 97.5% CI around estimated VE above 0%.
  - In the event that the attack rate is lower than anticipated and there have not been 450 accumulated cases in the observation period 14 days to 12 months post dose 3, then the period of observation contributing to the primary analysis will be extended. The analysis will be conducted when 450 cases have accumulated, or until boost (visit 22) (approximately 18 months post dose 3) whichever occurs earlier.
- Analysis of secondary endpoint: severe disease
  - A secondary analysis of severe malaria will be performed when 250 episodes of severe malaria meeting the primary case definition, pooled over the study centers and age categories, have occurred, or at the end of the follow-up period (Month 32), whichever occurs first.
  - 250 episodes give 80% power to detect 30% VE with a lower limit of the 95% CI above 0%. Assuming 50% VE 250 episodes give 90% power to detect a lower limit of the 95% CI above 25%.
- Analysis of secondary endpoint: antibodies to polio serotypes 1, 2 and 3
  - A secondary analysis of the immune response for polio serotypes 1, 2 and 3 when OPV is given on a primary schedule co-administered with DTPwHepB/Hib (Tritanrix HepB/Hib) with or without RTS,S/AS01E at screening, at 1 month post primary course (Visit 5) and following a booster dose at 18 months post primary (with or without RTS,S/AS01E) at one month post boost.
- The first 200 subjects enrolled at each center, in each age category, will form a subset for additional safety and immunology endpoints. Safety evaluations comprise the collection of solicited reactogenicity and the use of antipyretic,

analgesic and systemic antibiotics. Immunogenicity evaluations comprise responses to anti-CS and anti-HBs antigens and responses to OPV only in the 6-12 weeks age category.

3.2. Study design of the extension

- Experimental design: Extension to the Phase III, multi-center, randomized, controlled trial Malaria-055
- Blinding:
  - Initial study (D0 to Month 32): Double-Blind
  - Extension: Single-blind
- Study groups: 3 groups in children 6 weeks to 17 months at first vaccination.
  - Primary schedule + booster R3R
  - Primary schedule + control vaccine R3C
  - Comparator group C3C
- Number of subjects: A maximum of 16 000 children are planned to be enrolled in the initial study. All subjects enrolled in the initial study for whom parents/guardians will give their consent for participation in the extension, who received at least one dose of study or control vaccine in the primary trial phase and whose Visit 35 took place before (and including) 30 September 2013, will be enrolled.
- Malaria control: Bednet usage and indoor residual spraying will be documented once for each subject before the last extension visit using the same methodology of the primary trial phase.
- Sampling timepoints: Blood samples will be taken at annual intervals during the extension (maximum 2 blood samples).
- Data collection: This study will use electronic case report form (eCRF) by remote data entry (RDE).
- Interval between study visits: Depending on the date of the last visit of the primary trial phase (Visit 34), subjects will have up to 3 clinic visits plus one field workers visit during this extension (Refer to Figure 2). If a subject does not attend Visit 34, the schedule of the following visit(s) will be based on the assumption that the Visit 34 occurred 32 months post Dose 1.
  - Subjects that have their last visit in the primary trial phase (Visit 34) BEFORE (and including) 30 June 2012 will have 3 clinic visits plus one field workers visit in the extension.
  - Subjects that have their last visit in the primary trial phase (Visit 34) between 1 July 2012 and 30 September 2013 will have 2 clinic visits plus one field workers visit in the extension.
  - No subjects will be enrolled in the extension study (Visit 35) after 30 September 2013.

Table 5 Intervals between study visits for subjects that have their last visit in the primary trial phase (Visit 34) BEFORE (and including) 30 Jun 2012

Interval	Optimal time for study visit (Study Months)	~~Maximum interval allowed~~
**Visit 35**	On same day as Visit 34*<br>~~(Study Month 32)~~	***Maximum interval:*** Visit 2 + 44 months
**Visit 34→Visit 36**	12 months**<br>~~(Study Month 44)~~	***Range:*** 11 to 13 months
**Visit 34→Visit 37§<br>(Field workers visit)**	***Nov 2013***<br>~~23 months**~~<br>~~(Study Month 55)~~	***Date:*** Oct 2013 to Dec 2013
**Visit 34→Visit 38**	***Dec 2013***<br>~~24 months**~~<br>~~(Study Month 56)~~	***Date:*** Nov 2013 to Jan 2014

* If subjects are not present at Visit 34, Visit 35 should be scheduled as soon as possible and up to a maximum of 12 months after Visit 34, assuming Visit 34 should have occurred at Visit 2 + 32 months

** If subjects are not present at Visit 34, Visit 36, 37 and 38 should be scheduled assuming Visit 34 should have occurred at Visit 2 + 32 months

§Visit 37 (Field workers visit) has to be conducted between 1 and 30 days before Visit 38.

Amended 08 August 2012

Table 6 Intervals between study visits for subjects that have their last visit in the primary trial phase (Visit 34) after 30 Jun 2012 and BEFORE (and including) 30 Sep 2013

Interval	Optimal time for study visit (Study Months)	~~Maximum interval allowed~~
**Visit 35**	On same day as Visit 34*<br>~~(Study Month 32)~~	***Maximum interval:*** Visit 2 + 44 months or 30 Sep 2013 at the latest
**Visit 34→Visit 37§<br>(Field workers visit)**	***Nov 2013***<br>~~11 months**~~<br>~~(Study Month 43)~~	***Date:*** Oct 2013 to Dec 2013
**Visit 34→Visit 38**	***Dec 2013***<br>~~12 months**~~<br>~~(Study Month 44)~~	***Date:*** Nov 2013 to Jan 2014

* If subjects are not present at Visit 34, Visit 35 should be scheduled as soon as possible and up to a maximum of 12 months after Visit 34, assuming Visit 34 should have occurred at Visit 2 + 32 months. No subjects will be enrolled for Visit 35 after 30 September 2013.

** If subjects are not present at Visit 34, Visit 37 and 38 should be scheduled assuming Visit 34 should have occurred at Visit 2 + 32 months

§Visit 37 (Field workers visit) has to be conducted between 1 and 30 days before Visit 38.

Amended 08 August 2012

- Informed consent: Freely given informed consent should be obtained from subjects' parent(s)/LAR(s) prior to participation in the extension. The informed consent must be signed at the first visit of the extension, Visit 35. To avoid any gap in data collection between the initial study and the extension, Visit 35 should ideally take place on the same day as Visit 34. If subjects are not present at Visit 34, Visit 35 and signature of informed consent should be scheduled as soon as possible and up to a maximum 12 months after Visit 34 should have occured.

- Surveillance for Efficacy during the extension:
  - Surveillance for severe malaria disease will be maintained at all inpatient facilities in the study areas. Children will be evaluated according to an algorithm to accurately define cases.
  - Surveillance for clinical malaria disease will be maintained at the outpatient facilities in the study areas of at least 3 participating centers of varying malaria transmission intensity.
  - Parasite prevalence and haemoglobin will be assessed annually on all subjects.
- Surveillance for Immunogenicity during the extension:
  - Anti-CS antibodies will be assessed in a subset of 200 subjects in each age category in at least three centers evaluating efficacy to clinical disease.
- Surveillance for Safety during the extension:
  - Surveillance to detect SAEs will be maintained at all inpatient facilities in the study areas.
  - Verbal autopsy will be performed on all cases of mortality occurring outside hospital.

Amended 01 December 2010

4. STUDY COHORT

4.1. Number of subjects / centers

This study will be conducted at a number of malaria-endemic countries in sub Saharan Africa. Full details of the geography, demographics, healthcare provision and relevant site-specific information for each center are provided in documentation accompanying this protocol.

A maximum of 16 000 children (a *minimum* of 6000 in each age category [i.e. children 6-12 weeks of age and children 5-17 months of age at first vaccination]) will be randomized.

4.2. Inclusion criteria for the primary trial phase

Amended 01 December 2010

All subjects must satisfy the following criteria at study entry:

- A male or female child of:
  - 5-17 months (inclusive) of age at time of first vaccination,
  - or between 6-12 weeks (inclusive) of age at time of first vaccination
    and
    NOT have already received a dose of vaccine against diphtheria, tetanus or pertussis or *Hemophilus influenzae* type B

and
must be > 28 days of age at screening.

Amended 19 August 2008

- Signed informed consent or thumb-printed and witnessed informed consent obtained from the parent(s)/guardian(s) of the child.
- Subjects who the investigator believes that their parents/guardians can and will comply with the requirements of the protocol (e.g. return for follow-up visits) should be enrolled in the study.

4.2.1. Inclusion criteria for the extension

To take part in the extension, all subjects must satisfy the following criteria:

- Subjects who were enrolled and who received at least one vaccine dose in the primary trial phase.
- Subjects who were present for Visit 35 on or before 30 September 2013.
- Subjects who the investigator believes that their parents/guardians can and will comply with the requirements of the protocol (e.g. return for follow-up visits) should be enrolled in the study.

Amended 01 December 2010

4.3. Exclusion criteria for enrolment for the primary trial phase

Amended 01 December 2010

The following criteria should be checked at the time of study entry. If any apply, the subject must not be included in the study:

- Acute disease at the time of enrolment (acute disease is defined as the presence of a moderate or severe illness with or without fever). All vaccines can be administered to persons with a minor illness such as diarrhea, mild upper respiratory infection with or without low-grade febrile illness, i.e. axillary temperature < 37.5°C).
- Acute or chronic, clinically significant pulmonary, cardiovascular, hepatic or renal functional abnormality, as determined by physical examination or laboratory screening tests.
- Anemia defined as:
  - hemoglobin < 5.0 g/dL.
  - or hemoglobin < 8 g/dL associated with clinical signs of heart failure or severe respiratory distress.

Amended 19 August 2008
Amended 24 October 2008
Amended 26 November 2009

- Major congenital defects.
- History of allergic reactions, significant IgE-mediated events or anaphylaxis to previous immunizations.
- Children with a past history of a neurological disorder or atypical febrile seizure (a febrile seizure is atypical if it meets one of the following criteria: not associated with fever; lasts > 5 minutes; focal (not generalized); followed by transient or persistent neurological abnormality; occurs in a child < 6 months of age).
- Children with malnutrition requiring hospital admission.
- Children currently meeting the criteria for HIV disease of Stage III or Stage IV severity as defined by the World Health Organization [WHO, 2005] (please refer to Appendix I for current guidelines). NB: a previous history of having Stage III or Stage IV HIV disease is NOT an exclusion criterion.
- History of allergic disease or reactions likely to be exacerbated by any component of the vaccine.
- Concurrently participating in another clinical study, at any time during the study period, in which the subject has been or will be exposed to a drug or vaccine that is not licensed for that indication (by one of the following authorities: FDA or EU member state or WHO [with respect to prequalification]) with the exception of studies with the objective of improving the drug treatment or clinical management of severe malaria disease.
- Use of a drug or vaccine that is not approved for that indication (by one of the following authorities: FDA or EU member state or WHO [with respect to prequalification]) other than the study vaccines within 30 days preceding the first dose of study vaccine, or planned use during the study period.
- Previous participation in any other malaria vaccine trial.
- Receipt of a vaccine within the preceding 7 days

Amended 19 August 2008

- Any other findings that the investigator feels would increase the risk of having an adverse outcome from participation in the trial.
- Any other findings that the investigator feels would result in data collected being incomplete or of poor quality.

4.4. Elimination criteria during the study

The following criteria should be checked at each visit subsequent to the first visit. If any become applicable during the study, it will not require withdrawal of the subject from the study but may determine a subject’s evaluability in the according-to-protocol (ATP) analysis. See Section 10.6 for definition of study cohorts to be evaluated.

- Use of a drug or vaccine that is not licensed for that indication (by one of the following authorities: FDA or EU member state or WHO [with respect to prequalification]) other than the study vaccine during the study period with the

exception of studies with the objective of improving the drug treatment or clinical management of severe malaria disease.

- Administration of a vaccine not foreseen by the study protocol within 30 days of any dose of RTS,S/AS01E or control vaccine with the exception of vaccines against polio, diphtheria, tetanus, pertussis, hepatitis B, *Hemophilus influenzae* type b, BCG, measles, pneumococcal disease or yellow fever which may not be given within one week of vaccination.

4.5. Contraindications to subsequent vaccination

4.5.1. Indications for deferral of vaccination

The following events constitute contraindications to administration of RTS,S/AS01E or control vaccine at that point in time; if any one of these AEs occur at the time scheduled for vaccination, the subject may be vaccinated at a later date, within the time window specified in the protocol, or withdrawn at the discretion of the investigator. AEs should be followed-up according to the instructions in Section 8.6:

- Acute disease at the time of vaccination (acute disease is defined as the presence of a moderate or severe illness with or without fever. All vaccines can be administered to persons with a minor illness such as diarrhea, mild upper respiratory infection with or without low-grade febrile illness, i.e. Axillary temperature <37.5°C).
- Axillary temperature of ≥ 37.5°C.
- Administration of a vaccine not foreseen by the study protocol within 30 days of any dose of RTS,S/AS01E or control vaccine with the exception of vaccines against polio, diphtheria, tetanus, pertussis, hepatitis B, *Hemophilus influenzae* type b, BCG, measles, pneumococcal disease or yellow fever which may not be given within one week of vaccination.
- Children currently meeting the criteria for HIV disease of Stage III or Stage IV severity as defined by the World Health Organization [WHO, 2005] (please refer to Appendix I for current guidelines).

4.5.2. Absolute contraindications to further vaccination

The following AEs constitute absolute contraindications to further administration of, RTS,S/AS01E or comparator vaccine; if any of these AEs occur during the study, the subject must not receive additional doses of the RTS,S/AS01E vaccine, but may continue other study procedures at the discretion of the investigator. AEs should be followed-up according to the instructions in Section 8.6. It is expected that the subject would continue full safety monitoring procedures, as per protocol. In children enrolled to the 6-12 week age category, the completion of infant immunization schedules is covered in Section 5.1.4.6.

- Acute allergic reaction, significant IgE-mediated event or anaphylactic shock following the administration of vaccine investigational product.

- Infants enrolled in the 6-12 week age category who receive a dose of a DTP-combination vaccine not foreseen in the protocol prior to the completion of the TritanrixHepB/Hib series, must not receive further doses of RTS,S/AS01E.
- Any other findings that the investigator feels would increase the risk of having an adverse outcome from participation in the trial.

5. CONDUCT OF STUDY

5.1. Ethics and regulatory considerations

The study will be conducted according to Good Clinical Practice (GCP), the Declaration of Helsinki and local rules and regulations of the country.

Amended 19 August 2008

Submission of the protocol and any protocol amendments to regulatory agencies will occur in accordance with local regulatory requirements. For some countries, submission to the local regulatory authority may not be required. When submission to the local regulatory authority is required, the timing of the submission relative to IEC/IRB submission or approval and whether or not the authority will provide their approval of or favorable opinion on the protocol or amendment before it can be implemented will depend on local regulatory requirements.

5.1.1. Institutional Review Board/Independent Ethics Committee (IRB/IEC)

The IRB/IEC must be constituted according to the local laws/customs of each participating country. The ICH Harmonized Tripartite Guideline for Good Clinical Practice recommends that the IRB/IEC should include:

a. At least five members.

b. At least one member whose primary area of interest is in a non-scientific area.

c. At least one member who is independent of the institution/study site.

Only those IRB/IEC members who are independent of the investigator and the sponsor of the study should vote/ provide opinion on a study-related matter.

A list of the professions of the IRB/IEC members will be obtained by the Principal Investigators (PIs) or their delegates.

This protocol and any other documents that the IRB/IEC may need to fulfill its responsibilities, including subject recruitment procedures and information about payments and compensation available to subjects will be submitted to each IRB/IEC by the PIs or their delegates. Written and dated unconditional approval from the IRB/IEC of the protocol and amendment (if any and applicable), written informed consent form, consent form updates (if any), subject recruitment procedure(s) (e.g. advertisements), and any other written information to be provided to subjects must be in the possession of the investigator and GSK before commencement of the study. This approval must refer to the

study by study title and number with exact protocol version and date, and should identify the documents reviewed and state the date of review. Relevant GSK Biologicals' data will be supplied by the Principal Investigator to the independent IRB/IEC for review and approval of the protocol. Verification of the unconditional approval of the IRB/IEC will be transmitted by the PIs to the GSK Biologicals' Central Study Coordinator using the standard notification form prior to shipment of vaccine supplies and materials to the site.

No deviations from, or changes to, the protocol should be initiated without prior written sponsor and IRB/IEC approval of an appropriate amendment, except when necessary to eliminate immediate hazards to the subjects or where permitted by all applicable regulatory requirements or when the change(s) involves only logistical or administrative aspects of the study (e.g. change of monitor[s], telephone number[s]). Administrative changes and amendments not submitted for approval are submitted to the IRB/IEC for information only. However, written verification that such documents were submitted should be obtained. Approvals/ verifications must be transmitted in writing to the GSK Biologicals clinical study monitor by the PI.

The IRB/IEC must be informed by the PI of:

- all subsequent protocol amendments, informed consent changes or revisions of other documents originally submitted for review
- serious and/or unexpected adverse events occurring during the study, where required
- all subsequent protocol administrative changes (for information)
- new information that may affect adversely the safety of the subjects or the conduct of the study
- regular updates and/or request for re-approval, where required
- when the study has been completed, where required.

If a trial is prematurely terminated or suspended for reasons including, but not limited to, safety or ethical issues or severe non-compliance, the sponsor will promptly inform the regulatory authorities of the termination or suspension and the reason(s) for the termination or suspension. If required by applicable regulations, the investigator must inform the IEC/IRB promptly and provide the reason for the suspension or termination (see Appendix A for further details).

5.1.2. Informed consent

A summary of the recruitment plan and informed consent process to be followed at each site is provided in the accompanying site-specific documents. The following principles will also apply.

In obtaining and documenting informed consent, the investigator should comply with the applicable regulatory requirement(s), and should adhere to GCP and to the ethical principles that have their origin in the Declaration of Helsinki. Prior to the beginning of the trial, the investigator should have the IRB/IEC's written approval/favorable opinion

of the written informed consent form and any other written information to be provided to the subjects and/or subjects' parents/guardians.

Informed consent will be obtained in accordance with 21 CFR 50.25.

Freely given informed consent should be obtained from subjects' parents/guardians prior to clinical trial participation.

Information should be given in both oral and written form whenever possible and as deemed appropriate by the IRB/IEC.

An investigator or designate will describe the protocol to potential subjects' parents/guardians face to face. The Informed Consent Form may be read to the subjects' parents/guardians, but in any event, the investigator or designate shall give the subjects' parents/guardians ample opportunity to inquire about details of the study and ask any questions before dating and signing (or thumbprinting, in the case of illiterate persons) the Informed Consent Form.

While informed consent information can be presented to groups at an initial information session, each subject's parents/guardian must be given the opportunity to individually pose questions to the investigator or designate prior to the subject's parents/guardians dating and signing/thumbprinting the Informed Consent Form.

Informed Consent Forms must be in a language fully comprehensible to the prospective subjects' parents/guardians. Informed consent shall be documented by the use of a written consent form approved by the IRB/IEC and signed and dated by the subjects' parents/guardians and by the person who conducted the informed consent discussion. The signature confirms the consent is based on information that has been understood. All illiterate individuals will have the study and the Informed Consent Form explained to them point by point by the interviewer in the presence of an impartial witness. The witness will personally sign and date the consent form. Oral witnessed consent will replace written consent only in countries where the local custom is contrary or if the subject's parents'/guardians' incapacity precludes this and provided that the local legal obligations are fulfilled.

Each subject's signed informed consent form must be kept on file by the investigator for possible inspection by Regulatory Authorities and/or GSK Biologicals' professional and Regulatory Compliance persons. The subjects' parents/guardians should receive a copy of the signed and dated written informed consent form and any other written information provided to the subjects' parents/guardians, and should receive copies of any signed and dated consent form updates. Any amendments to the written information will be provided to subjects' parents/guardians.

Both the informed consent discussion and the written informed consent form and any other written information to be provided to the subjects' parents/guardians should include explanations of the following:

a. That the trial involves research.

b. The purpose of the trial.

c. The trial treatment(s) and the probability for random assignment to each treatment.

d. The trial procedures to be followed, including all invasive procedures.

e. The subject's parents'/guardians' responsibilities.

f. Those aspects of the trial that are experimental.

g. The reasonably foreseeable risks or inconveniences to the subjects and, when applicable, to an embryo, fetus or nursing infant.

h. The reasonable expected benefits. When there is no intended clinical benefit to subjects, the subjects' parents/guardians should be made aware of this.

i. The alternative procedure(s) or course(s) of treatment/ methods of prevention that may be available to subjects, and their important potential benefits and risks.

j. The compensation and/or treatment available to subjects in the event of trial-related injury.

k. The anticipated prorated payment, if any, to subjects' parents/guardians for participating in the trial.

l. The anticipated expenses, if any, to subjects' parents/guardians for participating in the trial.

m. That the subjects' participation in the trial is voluntary and subjects' parents/guardians may refuse to participate or withdraw from the trial, at any time, without penalty or loss of benefits to which subjects are otherwise entitled.

n. That the monitor(s), the auditor(s), the IRB/IEC, and the regulatory authority(ies) will be granted direct access to the subject's original medical records for verification of clinical trial procedures and/or data, without violating the confidentiality of subjects, to the extent permitted by the applicable laws and regulations and that, by signing a written informed consent, the subject's parents/guardians are authorizing such access.

o. That records identifying subjects will be kept confidential and, to the extent permitted by the applicable laws and/or regulations, will not be made publicly available. If the results of the trial are published, subjects' identity will remain confidential.

p. That the subjects' parents/guardians will be informed in a timely manner if information becomes available that may be relevant to the subjects' parents/guardians willingness for continued participation in the trial.

q. The person(s) to contact for further information regarding the trial and the rights of trial subjects, and who to contact in the event of trial-related injury.

r. The foreseeable circumstances and/or reasons under which a subject's participation in the trial may be terminated.

s. The expected duration of a subject's participation in the trial.

t. The approximate number of subjects involved in the trial.

GSK Biologicals will prepare a model Informed Consent Form which will embody all the elements described above. While it is strongly recommended that this model document be

followed as closely as possible, the informed consent requirements given in this document are not intended to pre-empt any local regulations which require additional information to be disclosed for informed consent to be legally effective. Clinical judgment, local regulations and requirements should guide the final structure and content of the document.

The investigator has the final responsibility for the final presentation of Informed Consent Form, respecting the mandatory requirements of local regulations. The consent form generated by the investigator with the assistance of the sponsor's representative, must be approved (along with the protocol, and any other necessary documentation) by the IRB/IEC and be acceptable to GSK Biologicals.

5.1.3. Storage of study documentation at investigator's sites

All study documentation containing personal information relating to study subjects will be kept in a secure locked area at the investigator's sites. Such documentation will only be made available to authorized personnel. All electronic data kept at the investigator's site are kept secure. Computer access is only available to authorized personnel.

5.1.4. Safety Monitoring Plan

This trial is overseen by a Data Safety Monitoring Board (DSMB) operating under a charter assisted by a Local Safety Monitor (LSM) at each site.

5.1.4.1. Data Safety Monitoring Board (DSMB)/Independent Data Monitoring Committee (IDMC)

The role of the DSMB includes the review of the implementation and progress of the study. It provides initial, regular, and closing advice on safety-related issues to GSK Biologicals. Its advice is based on the interpretation of study data with reference to the study protocol.

The DSMB will meet before the initiation of the study (pre-initiation review), 6-monthly during the enrollment phase and annually thereafter. They will review the Protocol and Statistical Analysis Plan (SAP). Other unscheduled meetings may be required. Meetings may be face to face or via teleconference. Meetings must be documented and minutes made available to the sponsors. The DSMB may, if deemed necessary, convene a meeting with, or request further information from the Principal Investigators, the Medical Monitor/Local Safety Monitors and GSK Biologicals' and MVI's designated project representatives at any stage of the study.

The DSMB may recommend to the sponsor to suspend the enrollment to the trial and/or vaccination across all sites based on their review of safety data arising in this trial or other relevant trials of the same product.

In October 2008, the Data Safety Monitoring Board Terms of Reference (Charter) was revised to match the GSK Biologicals standard template and format. The membership and functioning rules of the DSMB in place have not been changed, but their role has

been clarified and their name modified to Independent Data Monitoring Committee (IDMC).

Amended 26 November 2009
Amended 23 January 2012

5.1.4.1.1. Data Reviewed by the DSMB

The DSMB will be informed of:

- All SAEs. SAEs judged to be related or fatal or occurring within 1 week of vaccination will be sent to the DSMB within 24 hours.
- Unblinded SAE summary tables will be produced 6-monthly during the enrolment phase, and annually thereafter, in advance of DSMB meetings.
- All withdrawals of study subjects by the Principal Investigator or the parent(s)/guardian(s) of a subject due to adverse events.
- New information that may affect adversely the safety of the subjects or the conduct of the study.
- All subsequent protocol amendments, informed consent changes or revisions of other documents originally submitted for review.
- All subsequent protocol modifications (for information).

5.1.4.2. Local Safety Monitor (LSM)

The overall role of the Local Safety Monitors (LSM), who are experienced clinicians based in-country, will be to support the clinical investigators and to act as a link between the investigators and the DSMB.

The LSM's role will include:

- Acting as the study volunteer's advocate.
- Promptly communicating relevant safety information to the DSMB.
- ~~Initiate the discussion of the need~~ ***Request the investigator*** to unblind ***in emergency*** a subject if deemed necessary to allow for adequate treatment.
- Temporary suspension of vaccination at the site (for which they are responsible) for a major safety concern.

Amended 24 October 2008
Amended 26 November 2009
Amended 08 August 2012

5.1.4.2.1. Data Reviewed by the LSM

The relevant LSM must be informed by the investigator on an 'as received' basis of:

- All SAEs within 24 hours.

- All withdrawals of study subjects by the Principal Investigators or the parent(s)/ guardian(s) of a subject due to adverse events.

5.1.4.3. Process if the trial is suspended

If the Principal Investigator or LSM suspends the trial at their site, they must immediately notify the DSMB, and GSK Biologicals (the study sponsor) via the contacts for safety reporting (refer to Section 8.7.2).

If the trial is suspended, the DSMB will review all available information and make a recommendation to the study sponsor (GSK Biologicals) whether to recommence the trial or to stop the trial permanently.

It is the responsibility of the sponsor (GSK Biologicals) to make the decision whether or not the trial should be stopped permanently.

5.1.4.4. Rescue plan for HBs

The rescue plan for this trial will be overseen by the DSMB.

At the time of the primary analysis in infants 6 to 12 weeks of age at enrollment after 14 months of follow-up, anti HBs antibody titers 1 month post Dose 3 will be analyzed in all children in this age category.

Any children who are found not to be seroprotected against anti-HBs will receive a booster dose of Hepatitis B vaccine in a timely manner.

5.1.4.5. Monitoring of polio serotypes 1, 2 and 3 seroprotection

There has been an unexpected finding in a phase 2 trial of lower seroprotection rates to polio 3 when OPV is given in co-administration with RTS,S/AS01E + DTPwHepB/Hib than with DTPwHepB/Hib alone (Section 1.4.3). Polio 3 titers in the co-administration group whilst lower than in the comparator group remain consistent with seroprotection levels in the published literature [WHO 1995, WHO 1997]. The consistency of this finding will be assessed by measuring the polio serotypes 1, 2 and 3 response induced by a primary series of OPV (4 doses commencing in the neonatal period) in a subset of children at all centers. Also all subjects will receive a booster dose of OPV at 20 months of age and seroprotection rates measured afterwards to assess the value of a booster dose when RTS,S/AS01E is given in co-administration with EPI antigens.

The DSMB will review the analysis of polio serotypes 1, 2 and 3 titres and advise on the need to offer additional doses to children in the trial.

Amended 19 August 2008
Amended 24 October 2008

5.1.4.6. Completion of immunization course for infants withdrawn during vaccination course

Infants may be withdrawn by the investigator for medical reasons or by their parent(s)/guardian(s) for any reason. If there is no contraindication, in the opinion of the Principal Investigator, for the infant receiving additional doses of licensed vaccine for D, T, Pw, Hib, hepatitis B and OPV, the importance of completion of a 3 dose regimen will be explained to the parent(s)/guardian(s). The parent(s)/guardian(s) will be offered the choice of completing a course of Tritanrix HepB™/Hib + OPV administered by the study team or completing their country's recommended EPI schedule at their local EPI clinic.

5.2. Case definitions

5.2.1. Clinical malaria disease

Table 7 Case definitions for clinical malaria

**1° definition**	*P. falciparum* asexual parasitemia > 5000 parasites/µL<br>**AND** presence of fever (axillary temperature ≥ 37.5°C) at the time of presentation<br>**AND** occurring in a child who is unwell and brought for treatment to a healthcare facility<br>**OR**<br>a case of malaria meeting the primary case definition of severe malaria disease (refer to Table 8)
**2° definition 1**	*P. falciparum* asexual parasitemia > 0<br>**AND** presence of fever (axillary temperature ≥ 37.5°C) at the time of presentation or history of fever within 24 hours of presentation<br>**AND** occurring in a child who is unwell and brought for treatment to a healthcare facility
**2° definition 2**	*P. falciparum* asexual parasitemia > 500 parasites/µL<br>**AND** presence of fever (axillary temperature ≥ 37.5°C) at the time of presentation<br>**AND** occurring in a child who is unwell and brought for treatment to a healthcare facility
**2° definition 3**	*P. falciparum* asexual parasitemia > 20 000 parasites/µL<br>**AND** presence of fever (axillary temperature ≥ 37.5°C) at the time of presentation<br>**AND** occurring in a child who is unwell and brought for treatment to a healthcare facility

5.2.2. Severe malaria disease

Severe malaria disease will be diagnosed based on symptoms and signs occurring at presentation or developing during admission according to the case definitions in Table 8.

Table 8 Primary case definition of severe malaria disease

P. falciparum > 5000 parasites per µL	
**AND** with one or more marker of disease severity	• Prostration<br>• Respiratory distress<br>• Blantyre score ≤ 2<br>• Seizures 2 or more<br>• Hypoglycemia < 2.2 mmol/L<br>• Acidosis BE ≤-10.0 mmol/L<br>• Lactate ≥ 5.0 mmol/L<br>• Anemia < 5.0 g/dL
**AND** without diagnosis of a co-morbidity	• Radiographically proven pneumonia<br>• Meningitis on CSF examination<br>• Positive blood culture<br>• Gastroenteritis with dehydration

- **Prostration** is defined as, in an acutely sick child, the inability to perform previously-acquired motor function: in a child previously able to stand, inability to stand; in a child previously able to sit, inability to sit and in a very young child, inability to suck.
- **Respiratory distress** is defined as lower chest wall indrawing or abnormally deep breathing.
- **2 or more seizures** occurring in the total time period including 24 h prior to admission, the emergency room and the hospitalization.
- **Radiographically proven pneumonia** is a consolidation or pleural effusion as defined in Appendix G on a chest x-ray taken within 72 h of admission.
- **Meningitis on CSF examination** is defined as WC ≥ 50 x10 ^{6}/L or positive culture of compatible organism or latex agglutination positive for Hib, pneumococci or meningococci [Berkley, 2001].
- **Gastroenteritis with dehydration** is defined as a history of 3 or more loose or watery stools in previous 24 h and an observed watery stool with decreased skin turgor (> 2 seconds for skin to return following skin pinch).
- **Positive blood culture** as defined in Appendix H on a blood culture taken within 72 h of admission.

Secondary case definitions of severe malaria disease are given in Table 9. Note that HIV testing will be guided by clinical judgment and local policy and informed consent and counseling will always be performed.

Table 9 Secondary case definitions of severe malaria disease

**2° definition 1**<br>"with co-morbidity"	*P. falciparum* > 5000 parasites per µL<br>**AND** with one or more marker of disease severity
**2° definition 2**<br>"without a density threshold"	*P. falciparum* > 0<br>**AND** with one or more marker of disease severity<br>**AND** without diagnosis of a co-morbidity
**2° definition 3**<br>"without HIV"	*P. falciparum* > 5000 parasites per µL<br>**AND** with one or more marker of disease severity<br>**AND** without diagnosis of a co-morbidity<br>**AND** without a confirmed diagnosis of HIV infection

5.2.3. Malaria hospitalization

Table 10 Case definitions for malaria hospitalization

**Definition 1**	A medical hospitalization with confirmed *P. falciparum* > 5000 parasites/µL<br>(excludes planned admissions for medical investigation/care or elective surgery and trauma)
**Definition 2**	A hospitalization which, in the judgment of the principal investigator, *P. falciparum* infection was the sole or a major contributing factor to the presentation

5.2.4. Fatal malaria

Table 11 Case definitions for fatal malaria

**1° definition**	A case of severe malaria meeting the primary case definition of severe malaria disease (see Table 8) with a fatal outcome<br>(i.e. is restricted to hospital mortality and does not include cause ascribed by verbal autopsy).
**2° definition 1**<br>"with co-morbidity"	A case of severe malaria meeting the secondary case definition 1 severe malaria disease (see Table 9) with a fatal outcome
**2° definition 2**<br>"without a density threshold"	A case of severe malaria meeting the secondary case definition 2 severe malaria disease (see Table 9) with a fatal outcome
**2° definition 3**<br>"without HIV"	A case of severe malaria meeting the secondary case definition 3 severe malaria disease (see Table 9) with a fatal outcome

5.2.5. Severe anemia

Table 12 Case definitions of incident severe anemia

**Definition 1**	A documented hemoglobin < 5.0 g/dL identified at clinical presentation to morbidity surveillance system in association with a *P. falciparum* parasitemia > 5000 parasites/µL
**Definition 2**	A documented hemoglobin < 5.0 g/dL identified at clinical presentation to morbidity surveillance system in association with a *P. falciparum* parasitemia > 0 parasites/µL
**Definition 3**	A documented hemoglobin < 5.0 g/dL identified at clinical presentation to morbidity surveillance system

Table 13 Case definitions of prevalent anemia

**Prevalent severe anemia**	A documented hemoglobin < 5.0 g/dL identified at a cross sectional survey
**Prevalent moderate anemia**	A documented hemoglobin < 8.0 g/dL identified at a cross sectional survey

5.2.6. All hospitalizations

Table 14 Case definitions for all medical hospitalization

**1° definition**	A medical hospitalization of any cause<br>(*excludes* planned admissions for medical investigation/care or elective surgery and trauma)

5.2.7. All-cause mortality

Table 15 Case definitions for all-cause mortality

**Definition 1**	A fatality (of any cause)<br>(*includes* mortality in the community and in hospital)
**Definition 2**	A fatality (medical cause)<br>(*includes* mortality in the community and in hospital)<br>(*excludes* trauma, which may be diagnosed by verbal autopsy)

5.2.8. Pneumonia

Pneumonia will be diagnosed based on symptoms and signs and CXR occurring at presentation according to the case definitions in Table 16.

Table 16 Case definitions for pneumonia

Amended 26 November 2009

**1° definition**	cough or difficulty breathing (on history)<br>**AND** tachypnea<br>**AND** lower chest wall indrawing
**2° definition 1**	1° case definition of pneumonia<br>**AND** CXR consolidation or pleural effusion as defined in Appendix G on a chest x-ray taken within 72 h of admission.
**2° definition 2**	1° case definition of pneumonia<br>**AND** CXR consolidation or pleural effusion or other infiltrates as defined in Appendix G on a chest x-ray taken within 72 h of admission.
**2° definition 3**	1° case definition of pneumonia<br>**AND** an oxygen saturation less than 90%

- **Tachypnea** (≥ 50 breaths per minute < 1 year, ≥ 40 breaths per minute ≥ 1year).

5.2.9. Sepsis

Table 17 Case definitions for sepsis

**1° definition**	A child with positive blood culture

- **Positive blood** culture as defined in Appendix H, on a blood culture taken within 72 hours of admission.

5.3. Efficacy endpoint capture

5.3.1. Surveillance for clinical malaria disease

All subjects presenting to health facilities in the study area will be evaluated as potential cases of clinical malaria disease. A blood sample for the evaluation of malaria parasites will be taken for all children who are reported to have had a fever within 24 hours of presentation or have a measured axillary temperature of ≥37.5°C.

Data points that will be captured in the CRF/eCRF for all cases of suspected clinical malaria disease are: history of fever; temperature; parasite density; treatment administered.

Note that the data on all subjects investigated for malaria will be captured in the CRF/eCRF regardless of whether the case was confirmed.

The clinical management of subjects presenting with suspected malaria is described in the site-specific documentation accompanying this protocol.

A blood sample for parasite genotyping will be taken for any subject investigated for malaria.

Amended 24 October 2008

5.3.2. Surveillance for severe malaria disease

All subjects presenting for admission through the outpatient and emergency departments of hospitals in the study areas will be evaluated as potential cases of severe malaria disease following the algorithm in Appendix E.

During the hospitalization, the subject's course will be monitored to capture the signs and blood parameters indicative of severe malaria disease. If the subject's condition changes from admission and he/she meets one of the criteria for additional investigations, these will be performed.

Harmonization of case evaluation across centers will be assured by training of clinicians in the assessment of clinical signs and the standardization of equipment and processes used for laboratory investigations (refer to Appendix E to Appendix I). On all admissions, data will be captured to support the endpoints of clinical malaria disease, severe malaria disease, other serious illness, malaria-specific and all medical hospitalization and mortality.

The clinical management of subjects presenting with suspected malaria is described in the site-specific documentation accompanying this protocol.

A blood sample for parasite genotyping will be taken for any subject investigated for malaria.

Amended 24 October 2008

5.3.3. Cross-sectional surveys

Two cross-sectional surveys to assess VE against prevalent parasitemia and anemia will be performed at 18 months post Dose 3 and 12 months post boost.

Children who are symptomatic at cross-sectional surveys will be assessed by a clinician and managed as appropriate.

At these visits, a blood sample will be taken for parasite genotyping.

Amended 24 October 2008

5.3.4. Cross-sectional survey in the extension

Depending on the date of the last visit in the initial study (Visit 34), subjects will have up to 2 blood samples (Visit 36 and Visit 38) during this extension to measure asexual

P. falciparum density, parasite genotyping and hemoglobin, and to perform, in a immunology subset, anti-CS antibody testing.

Children who are symptomatic at these cross-sectional surveys will be assessed by a clinician and managed as appropriate.

Amended 01 December 2010

5.3.5. Verbal autopsy process

Verbal autopsies (VA) will be carried out on all children who die outside a health facility to ascribe the cause of death. The questionnaire used will be based on the INDEPTH standard and adapted to be locally appropriate [WHO, 2007]. To support the timely reporting of SAEs, the diagnoses will be made according to the usual processes of each centre.

At study end and at the end of the extension, all forms will be read by a central panel composed of 3 experienced VA reviewers. Each form will be read by each of the three reviewers independently. They will record 1) the disease or condition directly leading to death, 2) any morbid conditions leading to the condition that directly causes death and 3) any other significant conditions contributing to the death, but not related to the disease or condition causing it [SAVVY, 2006]. The diagnoses will be coded according to an adapted form of the International Classification Disease (ICD). If a minimum of 2 reviewers are in agreement a cause of death is ascribed. If there is no agreement between the three reviewers a consensus meeting will be held where an agreement is reached wherever possible or the cause of death is recorded as unknown.

Amended 19 August 2008
Amended 01 December 2010

5.4. Subject identification

Subject numbers will be assigned sequentially to subjects consenting to participate in the study, according to the range of subject numbers allocated to each study centre.

5.5. Outline of study procedures

Table 18 List of study procedures

	Screen [1]	Dose 1		Dose 2		Dose 3						Boost					End
STUDY DAY	**-30 to 0**	**0**	**1 to 6**	**30**	**31 to 36**	**60**	**61 to 66**						**B +1 to B +6**				
STUDY MONTH	**-1 to 0**	**0**		**1**		**2**		**3**	**4-13**	**14**	**15-19**	**20**		**21**	**22-30**	**31**	**32**
VISIT	*1*	*2*	*a to f*	*3*	*g to l*	*4*	*m to q*	*5*	*6-15* [30]	*16*	*17-21* [30]	*22*	*r to w*	*23*	*24-32* [30]	*33*	*34*
**STUDY PROCEDURES**																	
Informed consent	●																
Assign Subject Number	●																
Issue identification card	○																
Check identification card		○	○	○	○	○	○	○	○	○	○	○	○	○	○	○	○
Collect demographics [2]	○	●															
Medical history	○	● [3]															
Physical examination	○	●		○		○						○					
Anthropometry [4]		●		○ [5]		○ [5]		● [6]				●					●
Temperature (prior to vaccination)		●		●		●						●					
Check inclusion/exclusion criteria	●	●															
Randomization		● [7]															
Assign treatment number		●															
Check elimination criteria				●		●		●	●	●	●	●		●	●	●	●
Check contraindications to vaccination		●		●		●						●					
Administer & document study vaccine (RTS,S or comparator)		●		●		●						●					
Administer & document TritanrixHepB/Hib [6]		●		●		●											
Administer & document OPV [6]		●		●		●						●					
Record previous doses of Hepatitis B vaccine		●															
Record previous doses of BCG and OPV [6]		●															
Recording of pneumococcal vaccine doses [9]		●						●				●					●
Recording of Hemophilus vaccine doses [10]		●						●				●					●
Recording of Vitamin A administration [9]		●						●				●					●
Recording of doses of IPTi [11]		●						●				●					●
Recording of concomitant EPI vaccine doses [12]		●		●		●		●				●		●			
Recording of concomitant medication [13]		●	●	●	●	●	●	●				●	●	●			
Record distance to nearest inpatient facility		●										●					
Record distance to nearest outpatient facility		●										●					
Document feeding history [15]												●					

	Screen [1]	Dose 1		Dose 2		Dose 3						Boost					End
STUDY DAY	**-30 to 0**	**0**	**1 to 6**	**30**	**31 to 36**	**60**	**61 to 66**						**B +1 to B +6**				
STUDY MONTH	**-1 to 0**	**0**		**1**		**2**		**3**	**4-13**	**14**	**15-19**	**20**		**21**	**22-30**	**31**	**32**
VISIT	*1*	*2*	*a to f*	*3*	*g to l*	*4*	*m to q*	*5*	*6-15* [30]	*16*	*17-21* [30]	*22*	*r to w*	*23*	*24-32* [30]	*33*	*34*
Documentation of bednets and residual spraying [16]										●						●	
Participation status review [17]										●		●					●
**SAFETY DATA**																	
Recording of solicited symptoms [18, 19]		●	●	●	●	●	●					●	●				
Recording of unsolicited AEs [20, 21, 28]		●	●	●	●	●	●	●				●	●	●			
Morbidity surveillance/record SAEs [20]	●	●	●	●	●	●	●	●	●	●	●	●	●	●	●	●	●
Recording of AEs of specific interest [29]	●	●	●	●	●	●	●	●	●	●	●	●	●	●	●	●	●
Document rescue plan[22]																	●
**EFFICACY DATA**																	
Clinical Malaria Disease [23]		●	●	●	●	●	●	●	●	●	●	●	●	●	●	●	●
Severe malaria, hospitalization, other serious illness		●	●	●	●	●	●	●	●	●	●	●	●	●	●	●	●
**BLOOD COLLECTION**																	
Blood sample taken	●							●				●		●			●
Parasite density												●					●
Parasite genotyping												●					●
Hemoglobin	●							●				●		●			●
Antibodies to CS [24]	●							●				●		●			●
Antibodies to HBs [25]	●							● [26]				●		●			●
Antibodies to polio serotypes 1, 2 and 3[27]	●							●						●			
**ANALYSIS**																	
Primary analysis 14½ months post Dose 1										●							
Secondary analysis at Month 20												●					
Secondary analysis for polio serotypes 1, 2 and 3 antibody responses														●			
Final analysis																	●

Amended 19 August 2008
Amended 24 October 2008
Amended 01 December 2010
Amended 23 January 2012

Table notes overleaf

Table notes

1. All children must be > 28 days of age at screening.
2. Including sex, date of birth, ethnicity, gestation (gestation to be solicited in children in the 6 to 12 week age category only).
3. Known HIV positivity will be documented at enrollment (not an exclusion criterion).
4. Anthropometry consists of length/height (children < 2 years, measure length: children ≥ 2 years measure standing height), weight, mid-upper arm circumference (Refer to Appendix K).
5. Measure weight only: to be carried out in children in 6 to 12 week age category only.
6. In 6 to 12 week age category only.
7. Randomization *must* occur after informed consent and completion of screening activities, but may be done in preparation for visit 2.
9. Since birth or previous enquiry. Record dates of doses.
10. In children in 5 to 17 month age category only. Since birth or previous enquiry. Record dates of doses.
11. i.e. Intermittent Preventative Treatment for Malaria (Infants). Since birth or previous enquiry. Record dates of doses.
12. Record concomitant vaccine doses not specified per protocol (i.e. within 30 days of a vaccine dose. e.g. measles, polio mass vaccination)
13. For the first 200 children enrolled in each age category at each site, record concomitant medication (anti-pyretics, analgesics, systemic antibiotics on the 6 days subsequent to vaccination)
15. Refer to Section 5.6 for wording of questions.
16. Refer to Appendix J.
17. i.e. Study conclusion page of the primary trial phase, detailed in Section 9.2.1.
18. First 200 subjects enrolled in each age category at each site will be evaluated.
19. Solicited symptoms are listed in Section 8.4.1. For the evaluation of fever, subject's temperature should be recorded in the CRF/eCRF.
20. Seizures occurring within 7 days of vaccination will be recorded in the CRF/eCRF as mentioned in Section 8.2.
21. Ongoing surveillance at health facilities will be captured in appropriate CRF/eCRF module.
22. Document in CRF the date of hepatitis B booster administration if in accordance with rescue plan.
23. Data points to be collected are specified at each presentation with suspected clinical malaria disease (see Section 5.3.1).
24. All subjects will have specimens taken: to evaluate vaccine response, the first 200 subjects in each age category at each site will be analyzed; to evaluate correlates of protection, a subset will be analyzed ; to evaluate the vaccine response in 'special subgroups' a subset will be analyzed.
25. All subjects will have specimens taken. To evaluate vaccine response: the first 200 subjects in each age category at each site at the first 2 timepoints will be analyzed.
to evaluate the vaccine response in 'special subgroups' a subset will be analyzed.
26. All samples in the 6 to 12 week category will be analyzed to support a rescue plan of hepatitis B response in children in the 6 to 12 week age category.
27. First 200 subjects enrolled in the 6 to 12 week age category at each site will be sampled.
28. All unsolicited AEs will be reported for 30 days following each vaccine dose for the first 200 children enrolled in each age category at each site and reported in the CRF/eCRF.
For all subjects, unsolicited AEs judged to be related to vaccination or leading to drop out occurring within 30 days following administration of each dose of vaccine will be recorded in the CRF/eCRF.
29. AEs of specific interest include seizures, rashes and mucocutaneous lesions within 30 days of vaccination, and immune-mediated disorders (IMD) over the entire study period. Refer to Section 8.4.1 for the details on reporting requirements for each AE of specific interest.
30. To fulfill the obligation of monthly contacts with all subjects, where home visits are conducted in addition to the scheduled visits, these should be recorded in the Additional Follow-up Visits module of the CRF/eCRF.
● is used to indicate a study procedure that requires documentation in the individual CRF/eCRF: ○ is used to indicate a study procedure that does not require documentation in the individual CRF/eCRF.

Amended 19 August 2008
Amended 26 November 2009
Amended 01 December 2010

Table 19 List of study procedures of the extension

~~**STUDY DAY**~~				
~~**STUDY MONTH**~~	~~**32**~~	~~**44**~~	~~**55**~~	~~**56**~~
**VISIT**	35	36 [9]	37	38
**STUDY PROCEDURES**				
Informed consent addendum	●			
Check identification card	○	○	○	○
Check inclusion criteria for extension	●			
Check elimination criteria		●		●
Anthropometry [1]		●		●
Recording of pneumococcal vaccine doses [9]		●		●
Recording of Vitamin A administration [2]		●		●
Recording of concomitant medication [3]		●		●
Record distance to nearest inpatient facility				●
Record distance to nearest outpatient facility				●
Documentation of bednets and residual spraying [4]			●	
Participation status review [5]				●
**SAFETY DATA**				
Morbidity surveillance/record SAEs [6]	●	●		●
Recording of IMDs [11]	●	●		●
**EFFICACY DATA**				
Clinical Malaria Disease [7]	●	●		●
Severe malaria, hospitalization, other serious illness	●	●		●
**BLOOD COLLECTION**				
Blood sample taken [10]		●		●
Parasite density		●		●
Parasite genotyping		●		●
Hemoglobin		●		●
Antibodies to CS [8]		●		●
**ANALYSIS**				
Extension analysis				●

Amended 01 December 2010

Amended 08 August 2012

Table notes overleaf

Table notes

1. Anthropometry consists of length/height (children < 2 years, measure length: children ≥ 2 years measure standing height), weight, mid-upper arm circumference (Refer to Appendix K).
2. Since birth or previous enquiry. Record dates of doses.
3. Refer to Section 6.9.2
4. Use of bednets and residual spraying will be checked once during the extension at Visit 37, between 1 and 30 days before Visit 38.
5. i.e. Study conclusion page of the extension, detailed in Section 9.2.1.
6. All SAEs will be recorded in the CRF/eCRF as mentioned in Section 8.2.
7. Data points to be collected are specified at each presentation with suspected clinical malaria disease (see Section 5.3.1). These data will only be collected from all subjects of each of the at least 3 selected sites.
8. Anti-CS antibody samples will be collected only on the first 200 subjects in each age category of the primary trial phase of each of the at least 3 selected sites.
9. Subjects having their Visit 34 after 30 June 2012 and before (and including) 30 September 2013 will not have Visit 36.
10. Subjects having their Visit 34 between 1 July 2012 and 30 September 2013 will have one blood sample taken at Visit 38. The first 200 subjects in each of the at least 3 selected sites will have 1.05 mL blood samples taken, and these samples will be analyzed, to evaluate the immunological vaccine response. From all other subjects, blood samples of 0.45 mL will be analysed.
11. AEs of specific interest include seizures, rashes and mucocutaneous lesions within 30 days of vaccination, and immune-mediated disorders (IMD) over the entire study period. Refer to section 8.4.1 for the details on reporting requirements for each AE of specific interest.
● is used to indicate a study procedure that requires documentation in the individual CRF/eCRF.
○ is used to indicate a study procedure that does not require documentation in the individual CRF/eCRF.

Amended 01 December 2010

It is the investigator's responsibility to ensure that the intervals between visits/contacts, tabulated in Table 20 are strictly followed.

Table 20 Intervals between study visits

Interval	Length of interval
Visit 2 → Visit 3	21 to 42 days
Visit 3 → Visit 4	21 to 42 days
Visit 4 → Visit 5	21 to 42 days
Visit 4 → Visit 16	12 months ± 1 month*,**
Visit 4 → Visit 22	18 months ± 1 month
Visit 22 → Visit 23	21 to 42 days
Visit 22 → Visit 34	12 months ± 1 month***

* For first 6000 5-17 months old children or February 2011 at the latest, even if the length of the interval is less than indicated
** For all 6-12 weeks old infants or April 2012 at the latest, even if the length of the interval is less than indicated
*** For all children: December 2013 at the latest, even if the length of the interval is less than indicated

Amended 01 December 2011

5.5.1. Intervals between study visits for the extension

Table 21 Intervals between study visits for subjects that have their last visit in the primary trial phase (Visit 34) BEFORE (and including) 30 Jun 2012

Interval	Optimal time for study visit (Study Months)	~~Maximum interval allowed~~
**Visit 35**	On same day as Visit 34*<br>~~(Study Month 32)~~	***Maximum interval:*** Visit 2 + 44 months
**Visit 34→Visit 36**	12 months**<br>~~(Study Month 44)~~	***Range:*** 11 to 13 months
**Visit 34→Visit 37§ (Field workers visit)**	***Nov 2013***<br>~~23 months**~~<br>~~(Study Month 55)~~	***Date:*** Oct 2013 to Dec 2013
**Visit 34→Visit 38**	***Dec 2013***<br>~~24 months**~~<br>~~(Study Month 56)~~	***Date:*** Nov 2013 to Jan 2014

* If subjects are not present at Visit 34, Visit 35 should be scheduled as soon as possible and up to a maximum of 12 months after Visit 34, assuming Visit 34 should have occurred at Visit 2 + 32 months
** If subjects are not present at Visit 34, Visit 36, 37 and 38 should be scheduled assuming Visit 34 should have occurred at Visit 2 + 32 months
§Visit 37 (Field workers visit) has to be conducted between 1 and 30 days before Visit 38.

Amended 08 August 2012

Table 22 Intervals between study visits for subjects that have their last visit in the primary trial phase (Visit 34) after 30 Jun 2012 and BEFORE (and including) 30 Sep 2013

Interval	Optimal time for study visit (Study Months)	~~Maximum interval allowed~~
**Visit 35**	On same day as Visit 34* ~~(Study Month 32)~~	***Maximum interval:*** Visit 2 + 44 months or 30 Sep 2013 at the latest
**Visit 34→Visit 37§ (Field workers visit)**	***Nov 2013*** ~~11 months**~~ ~~(Study Month 43)~~	***Date:*** Oct 2013 to Dec 2013
**Visit 34→Visit 38**	***Dec 2013*** ~~12 months**~~ ~~(Study Month 44)~~	***Date:*** Nov 2013 to Jan 2014

* If subjects are not present at Visit 34, Visit 35 should be scheduled as soon as possible and up to a maximum of 12 months after Visit 34, assuming Visit 34 should have occurred at Visit 2 + 32 months. No subjects will be enrolled for Visit 35 after 30 September 2013.
** If subjects are not present at Visit 34, Visit 37 and 38 should be scheduled assuming Visit 34 should have occurred at Visit 2 + 32 months
§Visit 37 (Field workers visit) has to be conducted between 1 and 30 days before Visit 38.

Amended 01 December 2010
Amended 08 August 2012

5.6. Detailed description of study stages/visits

When materials are provided by GSK Biologicals, it is **MANDATORY** that all clinical samples (including serum samples) will be collected and stored using exclusively those materials in the appropriate manner. The use of other materials could result in the exclusion of the subject from the ATP analysis (See Section 10.6 for definition of study cohorts to be evaluated). The investigator must ensure that his/her personnel and the laboratory(ies) under his/her supervision comply with this requirement. However, when GSK Biologicals does not provide material for collecting and storing clinical samples, then appropriate materials from the investigator's site are to be used. Refer to Appendix B and Appendix C.

The vaccinees will be observed closely for at least 30 minutes, with appropriate medical treatment readily available in case of an anaphylactic reaction following the administration of vaccines.

The subjects' parents/guardians will be instructed to contact the investigator immediately should the subject manifest any signs or symptoms they perceive as serious.

Study Visits Amended 19 August 2008
Study Visits Amended 24 October 2008
Study Visits Amended 01 December 2010

Visit 1: Screening
Day -30 to Day 0

- Obtain signed, dated, thumb printed informed consent from the parent(s)/guardian(s).

- Assign subject number.
- Issue identification card.
- Check inclusion/exclusion criteria.
- Collect demographics.
  - Gestation at birth will be solicited in children in the 6 to 12 week age category by the question ‘was the baby born in the 37th week of gestation or later?’. If no, the approximate duration of gestation in weeks, at birth will be recorded.
- Carry out physical examination and take medical history.
- Blood sample to collect 1.8 mL blood for analysis of:
  - Hemoglobin.
  - Serology (antibodies to CS, HBs and polio 1, 2, 3).
- Record any SAEs that may have occurred as a result of study procedures.

Visit 2: Vaccination 1
Day 0

- Check study identification card of vaccinee.
- Collect demographics.
- Carry out physical examination, take medical history, anthropometry.
- Check inclusion/exclusion criteria.
- Check contraindications to vaccination.
- Randomize subject (note: must occur after informed consent).
- Record pre-vaccination body temperature.
- Record previous doses of hepatitis B vaccine and pneumococcal vaccine.
- Record previous doses of Hemophilus vaccine (vaccinees in the 5 to 17 month age category only) since birth.
- Record previous doses of BCG and OPV since birth (vaccinees in the 6 to 12 week age category only).
- Record previous Vitamin A administration since birth.
- Record concomitant vaccine doses not specified per protocol (i.e. within 30 days of a vaccine dose. e.g. measles, polio mass vaccination).
- Record previous doses of IPTi.
- Record distance to nearest inpatient and to nearest outpatient facility.
- Record Treatment Number.
- Administer the first dose of RTS,S/AS01E or comparator vaccine.

- Administer in infants of the 6 to 12 weeks age category, OPV and the first dose of TritanrixHepB/Hib.

Each child will be assessed for at least 30 minutes after vaccination to evaluate and treat any acute adverse events. Advise parents to contact the investigator immediately if their child manifests any signs or symptoms they perceive as serious.

- Record any post-vaccination SAEs.
- Record any post-vaccination unsolicited adverse events.
- In first 200 children in each age category at each site only:
  - Record any post-vaccination solicited symptoms.
  - Record concomitant medication (anti-pyretics, analgesics, systemic antibiotics on the day of vaccination).

Follow-up visits a to f
Days 1, 2, 3, 4, 5 and 6

these visits are only for the first 200 subjects in each age category enrolled at each site

---

- Check study identification card of vaccinee.
- Record local (pain, redness and swelling at the injection site) and general (fever, irritability / fussiness, drowsiness, loss of appetite) solicited adverse events.
- Record SAEs experienced by the vaccinee since the last visit.
- Record unsolicited adverse events experienced by the vaccinee since the last visit.
- Record concomitant medication (anti-pyretics, analgesics, systemic antibiotics on the 6 days subsequent to vaccination).

Visit 3: Vaccination 2
Day 30

- Check study identification card of vaccinee.
- Carry out physical examination.
- Measure body weight in infants from the 6 to 12 weeks age category only.
- Check contraindications to vaccination.
- Check elimination criteria.
- Record pre-vaccination body temperature.
- Record concomitant vaccine doses not specified per protocol (i.e. within 30 days of a vaccine dose. e.g. measles, polio mass vaccination).
- Administer the second dose of RTS,S/AS01E or comparator vaccine.
- Administer in infants of the 6 to 12 weeks age category, OPV and the second dose of TritanrixHepB/Hib.

Each child will be assessed for at least 30 minutes after vaccination to evaluate and treat any acute adverse events. Advise parents to contact the investigator immediately if their child manifests any signs or symptoms they perceive as serious.

- Record any post-vaccination SAEs.
- Record any post-vaccination unsolicited adverse events.
- *In first 200 children in each age category at each site only:*
  - Record any post-vaccination solicited symptoms.
  - Record concomitant medication (anti-pyretics, analgesics, systemic antibiotics on the day of vaccination).

Follow-up visits g to l
Days 31, 32, 33, 34, 35 and 36

these visits are only for the first 200 subjects in each age category enrolled at each site

---

- Check study identification card of vaccinee.
- Record local (pain, redness and swelling at the injection site) and general (fever, irritability / fussiness, drowsiness, loss of appetite) solicited adverse events.
- Record SAEs experienced by the vaccinee since the last visit.
- Record unsolicited adverse events experienced by the vaccinee since the last visit.
- Record concomitant medication (anti-pyretics, analgesics, systemic antibiotics on the 6 days subsequent to vaccination).

Visit 4: Vaccination 3
Day 60

- Check study identification card of vaccinee.
- Carry out physical examination.
- Measure body weight in infants from the 6 to 12 weeks age category only.
- Check contraindications to vaccination.
- Check elimination criteria.
- Record pre-vaccination body temperature.
- Record any post-vaccination SAEs.
- Record concomitant vaccine doses not specified per protocol (i.e. within 30 days of a vaccine dose. e.g. measles, polio mass vaccination).
- Administer the third dose of RTS,S/AS01E or comparator vaccine.
- Administer in infants of the 6 to 12 weeks age category, OPV and the third dose of TritanrixHepB/Hib.

Each child will be assessed for at least 30 minutes after vaccination to evaluate and treat any acute adverse events. Advise parents to contact the investigator immediately if their child manifests any signs or symptoms they perceive as serious.

- Record any post-vaccination SAEs.
- Record any post-vaccination unsolicited adverse events.
- *In first 200 children in each age category at each site only:*
  - Record any post-vaccination solicited symptoms.
  - Record concomitant medication (anti-pyretics, analgesics, systemic antibiotics on the day of vaccination).

Follow-up visits m to q
Days 61, 62, 63, 64, 65 and 66

these visits are only for the first 200 subjects in each age category enrolled at each site

---

- Check study identification card of vaccinee.
- Record local (pain, redness and swelling at the injection site) and general (fever, irritability / fussiness, drowsiness, loss of appetite) solicited adverse events.
- Record SAEs experienced by the vaccinee since the last visit.
- Record unsolicited adverse events experienced by the vaccinee since the last visit.
- Record concomitant medication (anti-pyretics, analgesics, systemic antibiotics on the 6 days subsequent to vaccination).

Visit 5: follow up post Dose 3
Month 3

- Check study identification card of vaccinee.
- Measure anthropometry in infants from the 6 to 12 weeks age category only.
- Record concomitant vaccine doses not specified per protocol (i.e. within 30 days of a vaccine dose. e.g. measles, polio mass vaccination).
- Record unsolicited adverse events experienced by the vaccinee since the last visit.
- Record SAEs experienced by the vaccinee since the last visit.
- Check elimination criteria.
- Record pneumococcal vaccine doses since visit 2.
- Record Hemophilus vaccine doses since visit 2 in vaccinees in the 5 to 17 month old category only.
- Record IPTi doses since visit 2.
- Record Vitamin A administration since visit 2.

- Record doses of IPTi since visit 2.
- Blood sample:
  - First 200 children vaccinated in each site in the 6 to 12 week old category: 1.8 mL blood for analysis of:

    Hemoglobin.

    Serology (antibodies to CS, HBs, polio 1, 2, 3).
  - All other children 1.2 mL blood for analysis of:

    Hemoglobin.

    Serology (antibodies to CS, HBs).
- *In first 200 children in each age category at each site only:*
  - Record concomitant medication (anti-pyretics, analgesics, systemic antibiotics on the 6 days subsequent to vaccination).

Visits 6 to 15: follow up post Dose 3
Months 4 to 13

- Check study identification card of vaccinee.
- Check elimination criteria.
- Record SAEs experienced by the vaccinee since the last visit.

Visits 16: follow up post Dose 3
Month 14
For first 6000 5-17 months old children or February 2011 at the latest, even if the length of the interval is less than indicated
For all 6-12 weeks old infants or April 2012 at the latest, even if the length of the interval is less than indicated

---

- Check study identification card of vaccinee.
- Check elimination criteria.
- Document bednet use and residual spraying (refer to Appendix J).
- Participation status review.
- Record SAEs experienced by the vaccinee since the last visit.

Visits 17 to 21: follow up post Dose 3
Months 15 to 19

- Check study identification card of vaccinee.
- Check elimination criteria.
- Record SAEs experienced by the vaccinee since the last visit.

Visit 22: Booster Vaccination
Month 20

- Check study identification card of vaccinee.
- Carry out physical examination, anthropometry.
- Check contraindications to vaccination.
- Check elimination criteria.
- Record covariates (distance to nearest inpatient and to nearest outpatient facility).
- Document feeding history:
  - record whether or not child is currently breastfeeding.
    if NO: record age (in months) at which breastfeeding was stopped.
  - record age of introduction of solid food.
  - record whether child was bottle-fed with milk formula as a young infant
- Record pneumococcal vaccine doses since visit 5.
- Record Hemophilus vaccine doses since visit 5 in vaccinees in the 5 to 17 month old category only.
- Record IPTi doses since visit 5.
- Record pre-vaccination body temperature.
- Record Vitamin A administration since visit 5.
- Record concomitant vaccine doses not specified per protocol (i.e. within 30 days of a vaccine dose. e.g. measles, polio mass vaccination).
- Record concomitant medication.
- Participation status review.
- Administer the booster dose of RTS,S/AS01E or comparator vaccine.
- Administer in infants of the 6 to 12 weeks age category OPV.

Each child will be assessed for at least 30 minutes after vaccination to evaluate and treat any acute adverse events. Advise parents to contact the investigator immediately if their child manifests any signs or symptoms they perceive as serious.

- Record any post-vaccination SAEs.
- Record any post-vaccination unsolicited adverse events.
- *In first 200 children in each age category at each site only:*
  - Record any post-vaccination solicited symptoms.
  - Record concomitant medication (anti-pyretics, analgesics, systemic antibiotics on the day of vaccination).
- Blood sample to collect 1.2 mL blood for analysis of:

- Hemoglobin.
- Serology (antibodies to CS, HBs).

- *In addition, blood will be drawn from* **all** *subjects for:*
  - Blood sample for malaria parasite density.
  - Blood sample for parasite genotyping.

Follow-up visits r to w
Days Boost +1, +2, +3, +4, +5 and +6

these visits are only for the first 200 subjects in each age category enrolled at each site

---

- Check study identification card of vaccinee.
- Record concomitant medication (anti-pyretics, analgesics, systemic antibiotics on the 6 days subsequent to vaccination).
- Record local (pain, redness and swelling at the injection site) and general (fever, irritability / fussiness, drowsiness, loss of appetite) solicited adverse events.
- Record SAEs experienced by the vaccinee since the last visit.
- Record unsolicited adverse events experienced by the vaccinee since the last visit.

Visit 23: follow up post Booster
Month 21

- Check study identification card of vaccinee.
- Record concomitant vaccine doses not specified per protocol (i.e. within 30 days of a vaccine dose. e.g. measles, polio mass vaccination).
- Check elimination criteria.
- Record unsolicited adverse events experienced by the vaccinee since the last visit.
- Record SAEs experienced by the vaccinee since the last visit.
- In first 200 children in each age category at each site only:
  - Record concomitant medication (anti-pyretics, analgesics, systemic antibiotics on the 6 days subsequent to vaccination).
- Blood sample:
  - First 200 children vaccinated in each site in the 6 to 12 week old category: 1.8 mL blood for analysis of:

    Hemoglobin.

    Serology (antibodies to CS, HBs, polio 1, 2, 3).
  - All other children 1.2 mL blood for analysis of:

    Hemoglobin.

Serology (antibodies to CS, HBs).

Visits 24 to 32: follow up post Booster
Months 22 to 30

- Check study identification card of vaccinee.
- Check elimination criteria.
- Record SAEs experienced by the vaccinee since the last visit.

Visit 33: follow up post Booster
Month 31

- Check study identification card of vaccinee.
- Check elimination criteria.
- Record SAEs experienced by the vaccinee since the last visit.
- Document bednet use and residual spraying.

Visit 34: follow up post Booster
Month 32
For all children: December 2013 at the latest, even if the length of the interval is less than indicated

- Check study identification card of vaccinee.
- Anthropometry.
- Participation status review.
- Record SAEs experienced by the vaccinee since the last visit.
- Check elimination criteria.
- Record pneumococcal vaccine doses since visit 22.
- Record Hemophilus vaccine doses since visit 22 in vaccinees in the 5 to 17 month old category only.
- Record IPTi doses since visit 22.
- Record Vitamin A administration since visit 22.
- Document rescue plan.
- Blood sample to collect 1.2 mL blood for analysis of:
    - Hemoglobin.
    - Serology (antibodies to CS, HBs).
- *In addition, blood will be drawn from* **all** *subjects for:*
    - Blood sample for malaria parasite density.

- Blood sample for parasite genotyping.

5.7. Detailed description of study visits during the extension

Depending on the date of the last visit in the primary trial phase (Visit 34), subjects will have up to 3 clinic visits plus one field workers visit during this extension. If a subject does not attend Visit 34, the schedule of the following visit(s) will be based on the assumption that the Visit 34 occurred 32 months post Dose 1.

- Subjects that have their last visit in the primary trial phase (Visit 34) BEFORE (and including) 30 June 2012 will have 3 clinic visits plus one field workers visit in the extension.
- Subjects that have their last visit in the primary trial phase (Visit 34) between 1 July 2012 and 30 September 2013 will have 2 clinic visits plus one field workers visit in the extension.
- No subjects will be enrolled in the extension study (Visit 35) after 30 September 2013.

Visit 35: extension

~~Month 32~~

Amended 08 August 2012

- Obtain signed, dated, thumb printed informed consent addendum from the parent(s)/guardian(s).
- Check study identification card of vaccinee.
- Check inclusion criteria.
- Record SAEs experienced by the vaccinee since the last visit.
- Record Immune Mediated Diseases (IMDs).

Visit 36: Extension

~~Month 44~~

This visit is only for subjects who had their Visit 34 before (and including) 30 June 2012

Amended 08 August 2012

- Check study identification card of vaccinee.
- Check elimination criteria.
- Anthropometry.
- Record pneumococcal vaccine doses since the last visit.
- Record Vitamin A administration since the last visit.
- Recording of concomitant medication (Refer to Section 6.9.2).

- Record SAEs experienced by the vaccinee since the last visit.
- Record Immune Mediated Diseases (IMDs).
- Blood sample:
  - The first 200 children enrolled in the primary trial phase at each of at least 3 sites collecting data on clinical malaria disease in each of the two age categories (6-12 week old and 5-17 month old): 1.05 mL blood for analysis of:

    Hemoglobin.

    Parasite density.

    Parasite genotyping.

    Serology (antibodies to CS).
  - All other children in the 6-12 week and 5-17 month old categories: 0.45 mL of blood for analysis of:

    Hemoglobin.

    Parasite density.

    Parasite genotyping.

Visit 37: Extension
Field worker visit
~~Month 55 or end 2013 whichever occurs earlier, and within one month before Visit 38~~
November 2013
Amended 08 August 2012

- Check study identification card of vaccinee.
- Document bednet use and residual spraying (refer to Appendix J).

Visit 38: Extension
~~Month 56 or end 2013 whichever occurs earlier~~
December 2013
Amended 08 August 2012

- Check study identification card of vaccinee.
- Check elimination criteria.
- Anthropometry.
- Record pneumococcal vaccine doses since the last visit.
- Record Vitamin A administration since the last visit.
- Recording of concomitant medication (Refer to Section 6.9.2).
- Record distance to nearest inpatient facility.
- Record distance to nearest outpatient facility.
- Participation status review.

- Record SAEs experienced by the vaccinee since the last visit.
- Record Immune Mediated Diseases (IMDs).
- Blood sample:
  - The first 200 children enrolled in the primary trial phase at each of at least 3 sites collecting data on clinical malaria disease in each of the two age categories (6-12 week old and 5-17 month old): 1.05 mL blood for analysis of:

    Hemoglobin.

    Parasite density.

    Parasite genotyping.

    Serology (antibodies to CS).
  - All other children in the 6-12 week and 5-17 month old categories: 0.45 mL of blood for analysis of:

    Hemoglobin.

    Parasite density.

    Parasite genotyping.

5.8. Sample handling and analysis

5.8.1. Treatment and storage of biological samples

See Appendix B of the protocol for details of treatment and storage of biological samples.

See Appendix C for instructions for shipment of biological samples.

5.8.2. Laboratory assays

See Appendix E for indications for laboratory assays in the evaluation of potential cases of severe malaria disease.

5.8.2.1. Hematology

Hematology (hemoglobin, white blood cell counts, platelets) will be assessed using automated machines, in the laboratory, according to manufacturer instructions and standard operating procedures.

5.8.2.2. Biochemistry

Biochemistry (Bilirubin, ALT, creatinine) will be assessed using automated machines, in the laboratory, according to manufacturer instructions and standard operating procedures.

Amended 19 August 2008

Glucose, lactate and base excess determination will be assessed immediately after blood sampling, using bed-side Abbot i-STAT™ portable machines or an equivalent validated machine [Bingham, 1999; Sediame, 1999], according to manufacturer instructions and standard operating procedures.

5.8.2.3. Microbiology

For details of microbiology testing on blood and body fluid, please refer to Appendix H.

5.8.2.4. Parasitology

All slides will be made in duplicate and stored. For each episode, the parasite density will be determined, independently by two readers and in the case of non-concordance a third read will be carried out.

Methods for the sampling, reading, the criteria for concordance and the formula for the attribution of a final parasite density and the QC processes to assure standardization across sites are detailed in Appendix F.

For the investigation of parasite genotypes, blood will be applied to Whatman FTA Mini Cards (or equivalent) for subsequent extraction of nucleic acid material.

Amended 24 October 2008

5.8.2.5. Radiology

Methods for taking and interpreting chest x-rays (CXR) are provided in Appendix G.

5.8.2.6. Humoral Immunology

Table 23 Laboratory immunological assays to be performed

Assay	Marker	Assay method	Test kit/ Manufacturer	Assay unit	Assay cut-off	Laboratory
Anti-CS antibodies	R32LR	ELISA	In-house ELISA	EU/mL	0.5	CEVAC
Anti-HBs antibodies		ELISA	In-house ELISA	mIU/mL	10*	CEVAC
Anti-polio 1, 2, 3 antibodies		Neutralization	In-house	Dilution factor	8	BIOMNIS

*: seroprotective level
CEVAC: Center for Vaccinology, Ghent University, Belgium
BIOMNIS: Biomnis Recherche Clinique, Lyon, France
ELISA: Enzyme-linked Immunosorbent Assay

Amended 19 August 2008
Amended 24 October 2008
Amended 23 January 2012

5.8.3. Blood sampling

Samples will not be labeled with information that directly identifies the subjects but will be coded with the identification number for the subject.

Collected samples will be stored for up to 15 years (counting from when the last subject performed the last study visit), unless local rules, regulations or guidelines require different timeframes or different procedures, which will then be in line with the subject consent. These extra requirements need to be communicated formally to and discussed and agreed with GSK Biologicals.

Table 24 Summary of blood sampling timepoints/immunological assays and volumes to be collected in children 6-12 weeks of age at first vaccination

Blood sampling timepoint		Test	Estimated No. subjects	Laboratory	Priority ranking	Volume of whole blood required
**Study month**	**Clinic Visit No.**					
Screening	1	Hemoglobin	8000	on-site	1	0.2 mL
		Anti-CS antibodies	8000**	CEVAC	2	1.0 mL
		Anti-HBs antibodies	8000**	CEVAC	3	
		Anti-polio 1, 2, 3 antibodies	2000**	BIOMNIS	4	0.6 mL
				**Total for Clinic Visit 1**		**1.8 mL†**
Month 3	5	Hemoglobin	8000	on-site	3	0.2 mL
		Anti-CS antibodies	8000**	CEVAC	2	1.0 mL
		Anti-HBs antibodies	8000**	CEVAC	1	
		Anti-polio 1, 2, 3 antibodies	2000**	BIOMNIS	4	0.6 mL
				**Total for Clinic Visit 5**		**1.2 mL (1.8 mL§)†**
Month 20	22	Parasite density	8000	on-site	1	--‡
		Parasite genotyping		HSPH	5	0.25 mL-
		Hemoglobin	8000	on-site	4	0.2 mL
		Anti-CS antibodies	8000**	CEVAC	2	1.0 mL
		Anti-HBs antibodies	8000**	CEVAC	3	
				**Total for Clinic Visit 22**		**1.45 mL †**
Month 21	23	Hemoglobin	8000	on-site	3	0.2 mL
		Anti-CS antibodies	8000**	CEVAC	1	1.0 mL
		Anti-HBs antibodies	8000**	CEVAC	2	
		Anti-polio 1, 2, 3 antibodies	2000**	BIOMNIS	4	0.6 mL
				**Total for Clinic Visit 23**		**1.2 mL (1.8 mL§)†**
Month 32	34	Parasite density	8000	on-site	1	--‡
		Parasite genotyping		HSPH	5	*0.25 mL*
		Hemoglobin	8000	on-site	4	0.2 mL
		Anti-CS antibodies	8000**	CEVAC	2	1.0 mL
		Anti-HBs antibodies	8000**	CEVAC	3	
				**Total for Clinic Visit 34**		**1.45 mL †**

**: a subset will be analyzed; refer to footnotes of Table 18; †: Refer to Section 5.8.3.1
§ 1.8 mL to be drawn only in the first 200 subjects; ‡ A volume of 16 µL is required for this test
CEVAC: Center for Vaccinology, Ghent University, Belgium
BIOMNIS: Biomnis Recherche Clinique, Lyon, France
HSPH: Harvard School of Public Health, Cambridge, Massachusetts, USA

Amended 19 August 2008, Amended 24 October 2008
Amended 23 January 2012

Table 25 Summary of blood sampling timepoints/immunological assays and volumes to be collected in children 5-17 months of age at first vaccination

Blood sampling timepoint		Test	Estimated No. subjects	Laboratory	Priority ranking	Volume of whole blood required
Study month	Clinic Visit No.					
Screening	1	Hemoglobin	8000	on-site	1	0.2 mL
		Anti-CS antibodies	8000**	CEVAC	2	1.0 mL
		Anti-HBs antibodies	8000**	CEVAC	3	
				**Total for Clinic Visit 1**		**1.2 mL†**
Month 3	5	Hemoglobin	8000	on-site	3	0.2 mL
		Anti-CS antibodies	8000**	CEVAC	1	1.0 mL
		Anti-HBs antibodies	8000**	CEVAC	2	
				**Total for Clinic Visit 5**		**1.2 mL†**
Month 20	22	Parasite density	8000	on-site	1	--‡
		Parasite genotyping		HSPH	5	0.25 mL
		Hemoglobin	8000	on-site	4	0.2 mL
		Anti-CS antibodies	8000**	CEVAC	2	1.0 mL
		Anti-HBs antibodies	8000**	CEVAC	3	
				**Total for Clinic Visit 22**		**1.45 mL†**
Month 21	23	Hemoglobin	8000	on-site	3	0.2 mL
		Anti-CS antibodies	8000**	CEVAC	1	1.0 mL
		Anti-HBs antibodies	8000**	CEVAC	2	
				**Total for Clinic Visit 23**		**1.2 mL†**
Month 32	34	Parasite density	8000	on-site	1	--‡
		Parasite genotyping		HSPH		0.25 mL
		Hemoglobin	8000	on-site	4	0.2 mL
		Anti-CS antibodies	8000**	CEVAC	2	1.0 mL
		Anti-HBs antibodies	8000**	CEVAC	3	
				**Total for Clinic Visit 34**		**1.45 mL †**

: a subset will be analyzed; refer to footnotes of Table 18 ; **†: Refer to Section 5.8.3.1;
‡ A volume of 16 µL is required for this test
CEVAC: Center for Vaccinology, Ghent University, Belgium
HSPH: Harvard School of Public Health, Cambridge, Massachusetts, USA

Amended 19 August 2008
Amended 24 October 2008
Amended 23 January 2012

Table 26 Summary of blood sampling timepoints/immunological assays and volumes to be collected in children of both age categories during extension

Blood sampling timepoint		Test	Estimated No. subjects#	Laboratory	Priority ranking	Volume of whole blood required
~~Study month~~	Clinic Visit No.					
~~Month 44~~	36	Parasite density	12000-16000	on-site	1	--‡
		Parasite genotyping	12000-16000	HSPH	2	0.25 mL
		Hemoglobin	12000-16000	on-site	3	0.2 mL
		Anti-CS antibodies	1200**	CEVAC	4	0.6 mL
				**Total for Clinic Visit 36**		**0.45 mL† (1.05 mL§)**
~~Month 56~~	38	Parasite density	12000-16000	*on-site*	3	--‡
		Parasite genotyping	12000-16000	*HSPH*	2	0.25 mL
		Hemoglobin	12000-16000	*on-site*	1	0.2 mL
		Anti-CS antibodies	1200**	*CEVAC*	4	0.6 mL
				**Total for Clinic Visit 38**		**0.45 mL† (1.05 mL§)**

**: Anti-CS antibody samples will be collected only on the first 200 subjects in each age category of the primary trial phase of each of the at least 3 selected sites.
§ In the subset for immunogenicity.
† Not in the subset for immunogenicity.
‡ A volume of 16 μL is required for this test.
CEVAC: Center for Vaccinology, Ghent University, Belgium
HSPH: Harvard School of Public Health, Cambridge, Massachusetts, USA

Amended 01 December 2010
Amended 23 January 2012
Amended 08 August 2012

GSK Biologicals may use collected samples for purposes related to the quality assurance of the laboratory tests described in this protocol. This may include the management of the quality of these current tests, the maintenance or improvement of these current tests, the development of new test methods for the markers described in this protocol, as well as making sure that new tests are comparable to previous methods and work reliably. The GSK Biologicals' clinical laboratories have established a Quality System supported by procedures. The activities of GSK Biologicals' clinical laboratories are audited regularly for quality assessment by an internal (sponsor-dependent) but laboratory-independent Quality Department.

In addition, GSK Biologicals may, if findings in the present or in other studies necessitate, conduct further investigation into the efficacy or immunogenicity of the RTS,S/AS01E vaccine, AS01E adjuvant, or further research in malaria. Under these circumstances, additional testing on the samples may be performed by GSK Biologicals outside the scope of this protocol. Any sample testing will be done in line with the consent of the individual subject. No human pharmacogenetic testing is planned for this study. Any human pharmacogenetic testing will require additional separate consent from the individual subjects and the ethics committee approval.

5.8.3.1. Ancillary studies

Additional assays may be performed if:

1. They are to further evaluate or understand vaccine effect or the immune responses induced by vaccine; in particular further evaluation of the cellular arm of the immune response.
2. The total blood draw including the ancillary study does not exceed 5 mL.
3. The ancillary study protocol is approved by the relevant IRBs.

Amended 19 August 2008

6. INVESTIGATIONAL PRODUCTS AND ADMINISTRATION

Note: Abbreviations for vaccination groups

The abbreviations below are used to describe the vaccination groups in the sections following:

R3R: Children to receive 3 doses of RTS,S/AS01E on a 0, 1, 2 schedule with an RTS,S/AS01E booster.

R3C: Children to receive 3 doses of RTS,S/AS01E on a 0, 1, 2 schedule without an RTS,S/AS01E booster.

C3C: Children to receive 3 doses of a comparator vaccine on a 0, 1, 2 schedule with a dose of comparator vaccine at Visit 22.

6.1. Study vaccines

The candidate vaccine (i.e. RTS,S/AS01E) to be used has been developed and manufactured by GSK Biologicals.

The Quality Control Standards and Requirements for the candidate vaccine are described in separate release protocols and the required approvals have been obtained.

Commercial vaccines (i.e. Tritanrix HepB™/Hib, Polio Sabin™, MCC vaccines and rabies vaccines) are assumed to comply with the specifications given in the manufacturer’s Summary of Product Characteristics.

Amended 19 August 2008

Note: Meningococcal C conjugate vaccines

To ensure consistent vaccine availability three brands of Meningococcal C Conjugate vaccine will be procured for this trial: Meningitec (Wyeth), NeisVac-C (Baxter) or Menjugate (Novartis). In preference Meningitec, which has product data on co-administration with hepatitis B will be used. It will be ensured that each child randomized

to receive three doses of MCC will receive all three primary immunization doses of MCC from the same manufacturer.

Note: Rabies vaccines

To ensure consistent vaccine availability, three cell-culture rabies vaccines from two manufacturers will be sourced for this trial (Sanofi-Pasteur and Novartis). It will be ensured that an individual child will receive all 3 doses of cell culture rabies vaccine from the same product.

Amended 24 October 2008

6.1.1. RTS,S/AS01E

The RTS,S/AS01E vaccine consists of two fractions: the lyophilized fraction containing the RTS,S antigen in one vial and the liquid fraction, consisting of the AS01E adjuvant in the other vial, to be used for reconstitution just prior to injection. The presentation of the reconstituted RTS,S/AS01E candidate malaria vaccine is an opalescent liquid.

6.1.1.1. RTS,S antigen presentation:

- The RTS,S antigen is presented as a lyophilized pellet in a monodose vial. One dose contains approximately 25 µg of antigen with sucrose as cryoprotectant.

6.1.1.2. AS01E adjuvant presentation:

- AS01E is presented in a monodose vial. One dose contains 25 µg of MPL® and 25 µg of Stimulon® QS21 (a triterpene glycoside purified from the bark of *Quillaja saponaria*) with liposomes.

6.1.2. Tritanrix HepB/Hib

DTPwHepB/Hib (Tritanrix HepB™/Hib, GSK Biologicals) is presented as two individual vaccine components: DTPwHepB (Tritanrix HepB™) as a suspension in a monodose glass vial and Hib (Hiberix™, GSK Biologicals) as a white pellet in a glass vial.

The vaccine contains diphtheria (D) and tetanus (T) toxoids, inactivated whole cell pertussis bacteria (Pw) and the purified major surface antigen of the hepatitis B virus (HBV), adsorbed on aluminum salts and purified polyribosyl-ribitol-phosphate capsular polysaccharide (PRP) of *Hemophilus influenzae* type b (Hib), covalently bound to tetanus toxoid.

Tritanrix HepB™/Hib is prepared by reconstitution of the Hiberix™ pellet with the Tritanrix HepB™ suspension. Each 0.5 mL dose contains not less than 30 International Units (IU) of adsorbed diphtheria toxoid, not less than 60 IU of adsorbed tetanus toxoid, not less than 4 IU of whole cell pertussis, 10µg of recombinant HBsAg protein and 10µg of purified capsular polysaccharide covalently bound to approximately 30µg tetanus toxoid. Tritanrix HepB™ also contains 2-phenoxyethanol, polysorbate 20, sodium chloride, thiomersal and water for injection. Hiberix also contains lactose.

6.1.3. Oral Polio Vaccine

Polio Sabin™ (GSK Biologicals) (OPV MRC5) is a stabilized suspension of types 1, 2 and 3 live attenuated polioviruses (Sabin strains): Type 1 (strain LSc, 2ab), Type 2 (strain P 712 ch, 2ab), Type 3 (strain Leon 12a, 1b). The viruses are propagated in human diploid cells.

The excipients of Polio Sabin™ (oral) comprise magnesium chloride, L-arginine, polysorbate 80, neomycin sulphate (residual), polymyxin B sulphate (residual) and purified water.

6.1.4. Meningococcal C conjugate vaccines

6.1.4.1. Meningitec

Meningitec is (after shaking) a homogenous white suspension presented in a vial.

One dose (0.5 mL) of Wyeth's Meningitec contains:

10 µg *Neisseria meningitidis* (strain C11) Group C oligosaccharide conjugated to 15 µg (approx) *Corynebacterium diptheriae* CRM_{197} protein adsorbed on aluminium phosphate (0.125 mg Al^{3+}).

The excipients include sodium chloride and water for injections.

6.1.4.2. NeisVac-C

NeisVac-C is (after shaking) a semi-opaque white to off-white suspension presented in a pre-filled syringe (PFS).

One dose (0.5 mL) of Baxter's NeisVac-C contains:

10 µg *Neisseria meningitidis* (strain C11) Group C polysaccharide (de-O-acetylated) conjugated to 10-20 µg tetanus toxoid adsorbed on aluminium hydroxide (0.5 mg Al^{3+}).

The excipients include sodium chloride and water for injections.

6.1.4.3. Menjugate

Menjugate is supplied in a single-dose (0.5 mL) presentation consisting of one vial containing a lyophilized powder (vaccine component) and a syringe containing the diluent (containing a suspension of aluminium hydroxide as the adjuvant).

Amended 26 November 2009

One dose (0.5 mL) of Novartis's Menjugate contains:

10 µg *Neisseria meningitidis* (strain C11) Group C oligosaccharide conjugated to 12.5-25 µg (approx) *Corynebacterium diptheriae* CRM_{197} protein adsorbed on aluminium hydroxide (1.0 mg).

The excipients include: in the MenC-CRM197 vial, mannitol, sodium phosphate monobasic monohydrate, sodium phosphate dibasic heptahydrate; in the adjuvant vial, sodium chloride, water for injections.

6.1.5. Cell-culture rabies vaccines

6.1.5.1. Sanofi-Pasteur's Human Diploid Cell Rabies Vaccine

Sanofi-Pasteur's Human Diploid Cell Rabies Vaccine is a sterile freeze-dried suspension of rabies virus prepared from strain PM 1503-3M obtained from the Wistar Institute, Philadelphia, PA.

The potency of one dose (1.0 mL) of Sanofi-Pasteur's Human Diploid Cell Rabies Vaccine is at least 2.5 IU of rabies antigen. Sanofi-Pasteur's Human Diploid Cell Rabies Vaccine is a creamy white to orange, freeze-dried vaccine for reconstitution with the diluent prior to use.

Amended 26 November 2009

A dose of 1.0 mL will be injected. The presentation of the reconstituted vaccine is as a pink to red liquid. One dose of Sanofi-Pasteur's Human Diploid Cell Rabies Vaccine contains less than 100 mg albumin, less than 150 μg neomycin sulphate and 20 μg phenol red indicator. The vaccine contains no preservative or stabilizer.

6.1.5.2. Rabipur/Rabavert Rabies Vaccine

Novartis' Rabipur/Rabavert, is a sterile freeze-dried vaccine obtained by growing the fixed-virus strain Flury LEP in primary cultures of chicken fibroblasts.

The potency of one dose (1.0 mL) Rabipur/Rabavert is at least 2.5 IU of rabies antigen. Rabipur/Rabavert is a white, freeze-dried vaccine for reconstitution with the diluent prior to use; the reconstituted vaccine is a clear to slightly opaque, colorless suspension.

A dose of 1.0 mL will be delivered. The presentation of the reconstituted vaccine is as a clear or slightly opaque suspension.

6.1.5.3. Sanofi-Pasteur's Vero-cell Rabies Vaccine

Sanofi-Pasteur's chromatographically purified Vero cell culture rabies vaccine Verorab is composed of rabies virus Wistar Rabies PM/W138 1503-3M strain (inactivated) (≥ 2.5 IU/0.5 mL dose).

Before reconstitution, the powder has a uniform white color.

A dose of 0.5 mL will be injected.

Amended 24 October 2008

6.2. Dosage and administration

The vaccinees will be observed closely for at least 30 minutes following the administration of all vaccines used in the study, with appropriate medical treatment readily available in case of an anaphylactic reaction.

Children and infants who received an incomplete primary vaccination schedule (not the 3 doses within the expected timings) must not receive the booster dose of RTS,S/AS01E or control vaccine.

Amended 01 December 2010

6.2.1. RTS,S/AS01E (0.5 mL dose)

RTS,S/AS01E will be supplied such that the reconstituted vaccine volume will provide a 0.5 mL pediatric dose. One 0.5 mL dose will be withdrawn from each vial and used.

Disinfect top of vaccine vial (pellet) and adjuvant vial with alcohol swabs and let dry. Withdraw the contents of the adjuvant vial in a syringe and inject adjuvant into the vial of lyophilized antigen. The pellet is then dissolved by gently shaking the vial. Wait for 1 minute to ensure complete dissolution of vial contents before withdrawing the content of the vial (0.5 mL). The reconstituted vaccine should be administered by slow IM injection, using a fresh 25G needle with length of 1 inch (25 mm): for children 6 to 12 weeks of age at enrollment inject into antero lateral thigh for primary course; for children 5 to 17 months of age at enrollment, inject into left deltoid for primary course; all children should be injected in the left deltoid for booster. Vaccine should be injected within 4 hours of reconstitution (storage at +2°C to +8°C).

6.2.2. TritanrixHepB/Hib (0.5 mL dose)

Disinfect top of vaccine vials with alcohol swabs and let dry. Shake the vial of Tritanrix HepB™ in order to obtain a homogenous turbid white suspension. Using a syringe, add the entire contents of the Tritanrix HepB™ vial to the vial containing the Hiberix™ pellet. Shake the mixture until the pellet is completely dissolved. The reconstituted Tritanrix HepB™/Hib vaccine should be inspected visually for any foreign particulate matter prior to administration; if this is observed the vaccine should be discarded. The reconstituted vaccine should be administered by slow IM injection, using a fresh 25G needle with length of 1 inch (25 mm), into the right antero-lateral thigh within 4 hours of reconstitution (storage at +2°C to +8°C).

6.2.3. OPV

The OPV vaccine will be administered orally. For the monodose container (European Pharmacopoeia formulation), one immunizing dose of 0.135 mL is contained in 3 drops. For the multidose container (WHO formulation), one immunizing dose of 0.1 mL is contained in 2 drops.

After opening, monodose containers should be kept in a refrigerator and used within eight hours. Multidose containers which are not completely used should be stored again at +2-8°C and should be used within eight hours.

Amended 24 October 2008
Amended 26 November 2009

6.2.4. Meningococcal C conjugate vaccines

6.2.4.1. Meningitec

Disinfect top of vial with alcohol swabs and let dry. Shake the vial in order to obtain a homogenous turbid white suspension. Visually inspect for any foreign particulate matter and/or variation of physical aspect. If this is observed discard the vaccine. The vaccine should be administered by slow IM injection (into left anterolateral thigh in children < 1 year, into left deltoid in children > 1 year), using a fresh 25G needle with length of 1 inch (25 mm).

Amended 26 November 2009

6.2.4.2. NeisVac-C

The PFS of vaccine should be well shaken in order to obtain a homogenous suspension. Visually inspect for any foreign particulate matter and/or variation of physical aspect. If this is observed discard the vaccine. The reconstituted vaccine should be administered by slow IM injection (into left anterolateral thigh in children < 1 year, into left deltoid in children > 1 year), using a fresh 25G needle with length of 1 inch (25 mm) immediately after reconstitution.

6.2.4.3. Menjugate

Disinfect top of vials with alcohol swabs and let dry. Inject the entire contents of the syringe into the vaccine powder vial. The vaccine is dissolved by gently shaking the vial to obtain a homogeneous suspension of the vaccine. Immediately withdraw 0.5 mL of the suspension and inject. The reconstituted vaccine should be administered by slow IM injection (into left anterolateral thigh in children < 1 year, into left deltoid in children > 1 year), using a fresh 25G needle with length of 1 inch (25 mm) immediately after reconstitution.

Amended 01 December 2010

6.2.5. Cell-culture rabies vaccines

6.2.5.1. Sanofi-Pasteur's Human Diploid Cell Rabies Vaccine (1.0 mL dose)

Disinfect top of vaccine vial with alcohol swabs and let dry. Inject the entire contents of the diluent syringe into the vaccine vial. The freeze-dried vaccine is dissolved by gently shaking the vial. Wait for 1 minute to ensure complete dissolution of vial contents before

withdrawing a sufficient volume to provide a 1.0 mL dose. The reconstituted vaccine should be administered by slow IM injection, using a fresh 25G needle with length of 1 inch (25 mm), into the left deltoid directly after reconstitution.

6.2.5.2. Rabipur/Rabavert Rabies Vaccine (1.0 mL dose)

Disinfect top of vaccine vial with alcohol swabs and let dry. Inject the entire contents of the diluent ampoule into the vaccine vial. The freeze-dried vaccine is dissolved by gently shaking the vial. Wait for 1 minute to ensure complete dissolution of vial contents before withdrawing a sufficient volume to provide a 1.0 mL dose. The original needle should then be replaced with a fresh needle for IM injection. The reconstituted vaccine should be administered by slow IM injection, using a fresh 25G needle with length of 1 inch (25 mm), into the left deltoid directly after reconstitution.

6.2.5.3. Sanofi-Pasteur's Vero-cell Rabies Vaccine (0.5 mL dose)

Disinfect top of vaccine vial with alcohol swabs and let dry. Inject the entire contents of the solvent syringe into the vaccine powder vial. The freeze-dried vaccine is dissolved by gently shaking the vial to obtain a homogeneous suspension of the vaccine. The reconstituted vaccine appears as a limpid liquid. Immediately withdraw 0.5 mL of the suspension and inject. The reconstituted vaccine should be administered by slow IM injection, using a fresh 25G needle with length of 1 inch (25 mm), into the left deltoid directly after reconstitution.

Amended 24 October 2008

6.3. Storage

All study vaccines to be administered to subjects must be stored in a safe and locked place with no access by unauthorized personnel.

A site assessment will be performed during the pre-study activities to ensure that the study vaccines will be stored in appropriate conditions during the study.

Study vaccines will be stored at the defined temperature range (i.e. +2 to +8°C).

The storage temperature of study vaccines will be monitored daily while using validated temperature monitoring devices and the temperature measurements will be recorded during working days, preferably at the same time of the day (e.g. at the beginning of the day). Freezing indication will be continuously controlled by an appropriate device placed close to the study vaccines.

Any temperature deviation, meaning temperature outside the defined range (i.e. +2 to +8°C), must be reported within 2 working days to the Clinical Trial Supplies Unit.

Following exposure to a temperature deviation, the study vaccines will not be used until written approval is given by the sponsor. In case of temperature deviations between 0 and +2°C, the impacted study vaccines can still be administered, but the site must take

adequate actions to get back to the defined range +2 to +8°C and avoid re-occurrence of such a temperature deviation.

Amended 01 December 2010

Storage conditions for transport of the study vaccines from country medical department or dispatch centre to study sites or between sites are described in Appendix D.

6.4. Treatment allocation and randomization

A randomization list will be generated at GSK Biologicals, Rixensart, using a standard SAS® (Statistical Analysis System) program and will be used to number the vaccines. A randomization blocking scheme (1: 1: 1 ratio [R3R: R3C: C3C] for each age category) will be used to ensure that balance between treatments is maintained: a treatment number will identify uniquely the vaccine doses to be administered to the same subject.

The vaccine doses will be distributed to the study centre while respecting the randomization block size. At first vaccination (Day 0 [Month 0]), subjects will be administered the vaccine dose in accordance with the treatment numbers provided by a central randomization system on Internet (SBIR).

The actual treatment number used for first vaccination of the subject must be recorded by the investigator in the CRF/eCRF (Randomization/Treatment Allocation Section).

6.4.1. Randomization of supplies

To allow GSK Biologicals to take advantage of greater rates of recruitment than anticipated at individual centers in this multicenter study and to thus reduce the overall study recruitment period, an over-randomization of supplies will be prepared.

6.4.2. Randomization of subjects

The treatment allocation at the investigator site will be performed using a central randomization system on Internet (SBIR).

Subjects will be allocated a subject number at the screening visit (Clinic Visit 1). Subject numbers will be issued consecutively at each site; there will be no overlap of subject numbers between sites. Prior to vaccination, at Clinic Visit 2, after verification of eligibility criteria, subjects will be allocated a Treatment Number using SBIR. The correspondence between the Subject Number and the Treatment Number will be noted down in the source document and subsequently transferred to the CRF/eCRF. The 1: 1 relationship between Treatment Number and Study Number established at Dose 1 of vaccine will be maintained for subsequent doses.

Amended 19 August 2008

Study subjects will receive study identification cards bearing their subject number.

The actual treatment number used for first vaccination of the subject must be recorded by the investigator in the CRF/eCRF (Randomization/Treatment Allocation Section).

6.5. Method of blinding and breaking the study blind

Data pertaining to RTS,S/AS01E or control vaccines will be collected in a double blinded (observer blind) manner. 'Double blinded (observer blind)' means that the vaccine recipient and their parent(s)/guardian(s) as well as those responsible for the evaluation of study endpoint data will all be unaware which treatment, RTS,S/AS01E or control vaccine, was administered to a particular subject. The vaccines in this study are of different appearance and volume. The contents of the syringe will be masked with an opaque label to ensure that parent(s)/guardian(s) are blinded. The only study staff aware of the vaccine assignment for RTS,S/AS01E or control vaccine will be those responsible for preparation and administration of vaccines; these staff will play no other role in the study except screening or collection of biospecimens which do not require clinical evaluation.

~~The LSM or in the event they are unavailable, the investigator, or person designated by the investigator, should contact GSK Biologicals' Central Safety physician directly or via the local safety contact (see below and Study Contact for Emergency Code Break in Sponsor Information page) to discuss the need for emergency unblinding. The GSK Biologicals' Central Safety Office will be allowed to access the individual randomization code.~~

~~Central Safety, GSK Biologicals, Rixensart will have access to the individual randomization code in electronic format. The code will be broken using the central randomization system (SBIR) by the GSK Biologicals' Central Safety physician (see below and Study Contact for Emergency Code Break in Sponsor Information) only in the case of medical events that the investigator/physician in charge of the subject feels cannot be treated without knowing the identity of the study vaccine(s).~~

Unblinding of a subject's individual treatment code should occur only in the case of a medical emergency, or in the event of a serious medical condition, when knowledge of the study treatment is essential for the clinical management or welfare of the subject, as judged by the investigator.

The emergency unblinding process consists of the automated system that allows the investigator to have unrestricted, immediate and direct access to the subject's individual study treatment.

The investigator has the option of contacting a GSK Biologicals' On-call Central Safety Physician (or Backup) if he/she needs medical advice. The safety physician may also support to perform the unblinding (i.e. he/she cannot access the automated Internet-based system).

Any emergency unblinding must be fully documented by using the Emergency Unblinding Documentation Form, which must be appropriately completed by the investigator and sent within 24 hours to GSK Biologicals.

Amended 26 November 2009
Amended 08 August 2012

Note that all the period of observation of the extension corresponds to the double-blind phase of the primary trial phase. However, cleaning of the data will be performed after the primary trial phase is unblinded. For that reason the extension is referred to as single-blinded. 'Single-blind' means that the vaccine recipient and their parent(s)/guardians will be unaware, and that the persons responsible for the collection of blood samples and the evaluation of study endpoint data will be aware of which treatment, RTS,S/AS01E or control vaccine, was administered to a particular subject.

Amended 01 December 2010

GSK Biologicals' policy (incorporating ICH E2A guidance, EU Clinical Trial Directive and Federal Regulations) is to unblind any serious adverse event (SAE) report associated with the use of the investigational product, which is unexpected and attributable/suspected, prior to regulatory reporting. The Clinical Safety physician is responsible for unblinding the treatment assignment in accordance with specified time frames for expedited reporting of SAEs (Refer to Section 8.7.2).

~~**GSK Biologicals Clinical Safety Physician (Study Contact for Emergency Code Break)**~~
~~**Phones for 7/7 day availability:**~~<br><br>[redacted] ~~(GSK Biologicals Central Safety Physician on-call)~~<br><br>[redacted] ~~(GSK Biologicals Central Safety Physician on-call)~~

GSK Biologicals' Contact information for Emergency Unblinding 24/24 hour and 7/7 day availability
GSK Biologicals' Central Safety Physician:<br><br>[redacted] ***(GSK Biologicals Central Safety Physician on-call)***<br><br>***GSK Biologicals' Central Safety Physician Back-up:***<br><br>[redacted]<br><br>***Emergency Unblinding Documentation Form transmission:***<br><br>***Fax:*** [redacted] ***or*** [redacted]

Amended 01 December 2010
Amended 08 August 2012

6.6. Replacement of unusable vaccine doses

Additional vaccine doses will be provided to replace those that are unusable (see Appendix D for details of supplies).

In addition to the vaccine doses provided for the planned number of subjects (including over-randomization when applicable), at least 3% additional doses will be supplied. In case a vaccine dose is broken or unusable, the investigator should replace it with a replacement vaccine dose. Although the sponsor need not be notified immediately in these cases (except in the case of cold-chain failure), documentation of the use of the replacement vaccine must be recorded by the investigator on the vaccine administration page of the CRF/eCRF and on the vaccine accountability form.

6.6.1. Replacement of unusable doses

The investigator will use the central randomization system (SBIR) to obtain the replacement vial number for any randomized treatment. The system will ensure, in a blinded manner, that the replacement vial is of the same formulation as the randomized vaccine.

Amended 24 October 2008

6.7. Packaging

See Appendix D.

6.8. Vaccine accountability

See Appendix D.

6.9. Concomitant medication/treatment

6.9.1. Concomitant medication/treatment during the primary trial phase

Immune modifying drugs and blood transfusions will be captured through the hospital surveillance system for severe disease for the duration of the trial.

Malaria treatments will be captured through the surveillance system for clinical malaria for the duration of the trial.

Antipyretics, analgesics, systemic antibiotics will be collected from children who are assessed for reactogenicity in the 6-day period following each vaccine dose (i.e. the first 200 children vaccinated in each age category at each study center). The indication for administration (‘prophylaxis’ or ‘treatment’) will be recorded. The reporting period will be from the day of vaccination and the 6 subsequent days.

Some EPI interventions will be recorded from birth to study end (Visit 34, Month 32). In some cases, specific CRF sections will be supplied for the recording of Vitamin A, IPTi, Hepatitis B, pneumococcal vaccines (all subjects), BCG, OPV (6 to12 weeks of age category), Hemophilus vaccines (5 to 17 months of age category).

Other vaccines administered concomitantly with study vaccine, not specified per protocol (i.e. within 30 days of a vaccine dose), will be recorded e.g. measles, polio mass vaccination. General concomitant vaccination pages are supplied for the recording of any other vaccines.

Amended 19 August 2008
Amended 23 January 2012

6.9.2. Concomitant medication/treatment during the extension

During the extension, immune modifying drugs and blood transfusions will be recorded through the hospital surveillance system for severe disease from study end of the primary trial phase (Visit 34, Month 32) until the last visit of the extension. Specific CRF sections will be supplied for the recording of Vitamin A and pneumococcal vaccines (all subjects).

Amended 01 December 2010

7. HEALTH ECONOMICS

Measures of vaccine efficacy on clinical disease endpoints as specified in this protocol may be utilized in an assessment of health economics.

Additional data to support the full health economic evaluation will be collected in an ancillary protocol to this protocol. This may include factors such as assessment of quality of life, subject preferences, the measurement of resource utilization and direct and indirect costs. The ancillary protocol and a subject informed consent will be submitted for IRB review.

8. ADVERSE EVENTS AND SERIOUS ADVERSE EVENTS

The investigators are responsible for the detection and documentation of events meeting the criteria and definition of an adverse event (AE) or serious adverse event (SAE) as provided in this protocol. During the study, when there is a safety evaluation, the investigator or site staff will be responsible for detecting AEs and SAEs, as detailed in this section of the protocol.

Each subject's parents/guardians will be instructed to contact the investigator immediately should the subject manifest any signs or symptoms they perceive as serious.

8.1. Definition of an adverse event

An AE is any untoward medical occurrence in a clinical investigation subject, temporally associated with the use of a medicinal product, whether or not considered related to the medicinal product.

An AE can therefore be any unfavorable and unintended sign (including an abnormal laboratory finding), symptom, or disease (new or exacerbated) temporally associated with the use of a medicinal product.

- Exacerbation of a chronic or intermittent pre-existing condition including either an increase in frequency and/or intensity of the condition.
- New conditions detected or diagnosed after investigational product administration even though it may have been present prior to the start of the study.
- Signs, symptoms, or the clinical sequelae of a suspected interaction.
- Signs, symptoms, or the clinical sequelae of a suspected overdose of either investigational product or a concurrent medication (overdose per se should not be reported as an AE/SAE).

Examples of an AE DO NOT include:

- Medical or surgical procedure (e.g. endoscopy, appendectomy); the condition that leads to the procedure is an AE.
- Situations where an untoward medical occurrence did not occur (social and/or convenience admission to a hospital).
- Anticipated day-to-day fluctuations of pre-existing disease(s) or condition(s) present or detected at the start of the study that do not worsen.
- For therapeutic studies, the disease/disorder being studied, or expected progression, signs, or symptoms of the disease/disorder being studied, unless more severe than expected for the subject's condition.

AEs may include pre- or post-treatment events that occur as a result of protocol-mandated procedures (i.e. invasive procedures, modification of subject's previous therapeutic regimen).

N.B. AEs to be recorded as endpoints (solicited events) are described in Section 8.4.1. All other AEs will be recorded as UNSOLICITED AEs (defined as those AEs judged to be related to vaccination, or leading to drop-out).

Example of events to be recorded in the medical history section of the CRF/eCRF:

- Pre-existing conditions or signs and/or symptoms present in a subject prior to the start of the study (i.e. prior to the first study vaccination).

8.2. Definition of a serious adverse event

A serious adverse event (SAE) is any untoward medical occurrence that:

a. results in death

b. is life-threatening

NOTE: The term 'life-threatening' in the definition of 'serious' refers to an event in which the subject was at risk of death at the time of the event. It does not refer to an event, which hypothetically might have caused death, if it were more severe.

c. requires hospitalization or prolongation of existing hospitalization,

NOTE: In general, hospitalization signifies that the subject has been detained (usually involving at least an overnight stay) at the hospital or emergency ward for observation and/or treatment that would not have been appropriate in the physician's office or out-patient setting. Complications that occur during hospitalization are AEs. If a complication prolongs hospitalization or fulfills any other serious criteria, the event is serious. When in doubt as to whether "hospitalization" occurred or was necessary, the AE should be considered serious.

Hospitalization for elective treatment of a pre-existing condition that did not worsen from baseline is not considered an AE.

d. results in disability/incapacity, or

NOTE: The term disability means a substantial disruption of a person's ability to conduct normal life functions. This definition is not intended to include experiences of relatively minor medical significance such as uncomplicated headache, nausea, vomiting, diarrhea, influenza, and accidental trauma (e.g. sprained ankle) which may interfere or prevent everyday life functions but do not constitute a substantial disruption.

e. a seizure within 30 days of vaccination.

NOTE: In this study all seizures occurring within a 30-day period of vaccination will be notified as SAEs. Key information pertaining to seizures occurring within 7 days of vaccination will be documented in the CRF/eCRF [refer to Bonhoeffer, 2004].

f. Medical or scientific judgment should be exercised in deciding whether reporting is appropriate in other situations, such as important medical events that may not be immediately life-threatening or result in death or hospitalization but may jeopardize the subject or may require medical or surgical intervention to prevent one of the

other outcomes listed in the above definition. These should also be considered serious. Examples of such events are invasive or malignant cancers, intensive treatment in an emergency room or at home for allergic bronchospasm and blood dyscrasias.

Amended 01 December 2010

8.3. Clinical laboratory parameters and other abnormal assessments qualifying as adverse events and serious adverse events

Abnormal laboratory findings (e.g. hematology) that are judged by the investigator to be clinically significant will be recorded as AEs or SAEs if they meet the definition of an AE, as defined in Section 8.1 or SAE, as defined in Section 8.2. Clinically significant abnormal laboratory findings or other abnormal assessments that are detected during the study or are present at baseline and significantly worsen following the start of the study will be reported as AEs or SAEs.

The investigator will exercise his or her medical and scientific judgment in deciding whether an abnormal laboratory finding or other abnormal assessment is clinically significant.

8.4. Time period, frequency, and method of detecting adverse events and serious adverse events

All unsolicited AEs will be reported for 30 days following each vaccine dose for the first 200 subjects in each age category enrolled at each center and reported in the CRF/eCRF. For all the subjects whose AEs judged to be related to vaccination or leading to drop out occurring within 30 days following administration of each dose of vaccine must be recorded on the Adverse Event form of the subject's CRF/eCRF.

Amended 26 November 2009

The standard time period for collecting and recording SAEs will begin at first receipt of vaccine and end at the last study visit. See Section 8.7 for instructions for reporting and recording SAEs.

In addition to the above-mentioned reporting requirements and in order to fulfill international reporting obligations, SAEs that are related to study participation (e.g. protocol-mandated procedures, invasive tests, a change from existing therapy) or are related to a concurrent GSK medication will be collected and recorded from the time the subject consents to participate in the study until she/he is discharged.

An overview of the protocol-required reporting periods for adverse events and serious adverse events is given in Table 27.

Table 27 Reporting periods for adverse events and serious adverse events

Study activity	Pre-vacc (consent obtained)	V1 D0	30 d post-V1 D30	V2 M1	30 d post-V2 M1+30d	V3 M2	30 d post-V3 M2+30d	M3-M19	B M20	30 d post B M20+30d	M21-M32
Reporting of AEs											
Reporting of SAEs											
Reporting of SAEs related to study participation and AEs of specific interest											

Pre-vacc: pre-vaccination; V: vaccination; M: study month d: days
* Refer to Section 8.4.1 for the details on reporting requirements for each AE of specific interest.

Table 28 Reporting periods for adverse events and serious adverse events, during the extension

Study activity	***Visit 35-38*** ~~M32 to M56*~~
Reporting of SAEs	
Reporting of SAEs related to study participation	
Reporting of IMDs**	

~~*Or end 2013 [Nov 2013-Jan 2014] whichever occur earlier.~~
** Refer to Section 8.4.1 for the details on reporting requirements for each AE of specific interest.

Amended 01 December 2010
Amended 08 August 2012

The investigator will inquire about the occurrence of AEs/SAEs at every visit/contact during the study and throughout the follow-up phase as appropriate.

SAEs will be identified by surveillance at the health facilities in the health centers in the study area and through monthly contacts with the study subjects.

The investigator or study clinician will fully document any such events in the CRF/eCRF including, where applicable, information from relevant hospital case records, autopsy reports and verbal autopsies.

All AEs either observed by the investigator or one of his clinical collaborators or identified through surveillance at health facilities in the study area or reported by the subject's parent/guardian spontaneously or in response to a direct question will be evaluated by the investigator. AEs judged by the investigator to be related or leading to drop out that have not been previously documented in the study will be recorded in the Adverse Event form within the subject's CRF/eCRF. The nature of each event, date and time (where appropriate) of onset, outcome, intensity and relationship to vaccination should be established.

As a consistent method of soliciting AEs, the subject or the subject’s parent/guardian should be asked a non-leading question such as:

“Has your child acted differently or felt different in any way since receiving the vaccine or since the last visit?”

N.B. The investigator should record only those AEs having occurred within the time frame defined above.

AEs already documented in the CRF/eCRF, i.e. at a previous assessment, and designated as “not recovered/not resolved” or “recovering/resolving” should be reviewed at subsequent visits, as necessary. If these have resolved, the documentation in the CRF/eCRF should be completed.

When an AE/SAE occurs, it is the responsibility of the investigator to review all documentation (e.g. hospital progress notes, laboratory, and diagnostics reports) relative to the event. The investigator will then record all relevant information regarding an AE/SAE on the CRF/eCRF or SAE Report Form as applicable. **It is not acceptable for the investigator to send photocopies of the subject’s medical records to GSK Biologicals in lieu of the appropriate completed AE/SAE pages. However, there may be instances when copies of medical records for certain cases are requested by GSK Biologicals. In this instance, all subject identifiers will be blinded on the copies of the medical records prior to submission to GSK Biologicals.**

The investigator will attempt to establish a diagnosis of the event based on signs, symptoms, and/or other clinical information. In such cases, the diagnosis should be documented as the AE/SAE and not the individual signs/symptoms.

For centers using RDE with eCRF, the electronic system using SAE screens in the eCRF will be the primary mode for reporting SAEs to GSK Biologicals during the study period. In case this electronic system for reporting SAEs does not work or after freezing of the subject’s eCRF, paper SAE Report Forms and the facsimile (Fax) system should be used to report SAEs. Refer to Section 8.7.2 for details of the back-up reporting system.

8.4.1. Adverse events of specific interest

Adverse events of specific interest for safety monitoring include seizures within 30 days of vaccination, immune-mediated disorders (IMD), rashes and mucocutaneous lesions. In study centers where support for specific laboratory testing and histopathology is not available routinely, support can be provided at a reference laboratory.

8.4.1.1. Seizures within 30 days of vaccination

All seizures occurring within a 30-day period of vaccination will be reported as SAEs and medical documentation of the events will be reported in appropriate targeted follow-up forms included in the CRF/eCRF. Key information pertaining to seizures occurring within 7 days of vaccination will be documented in the CRF/eCRF [refer to Bonhoeffer, 2004].

8.4.1.2. Immune-mediated disorders (IMD)

Occurrence of IMDs such as those listed below will be reported for all subjects over the entire study period. Medical documentation of the events will be reported in appropriate targeted follow-up forms included in the CRF/eCRF. The specific interest in the detailed description of occurrence of IMDs results from the theoretical concern that vaccination may interfere with immunological self-tolerance. These AEs of specific interest will be reported as SAEs in the CRF/eCRF.

- Neuroinflammatory disorders: Optic neuritis, multiple sclerosis, demyelinating disease, transverse myelitis, Guillain-Barré syndrome, myasthenia gravis, encephalitis, neuritis, Bell’s palsy.
- Musculoskeletal disorders: Systemic lupus erythematosus, cutaneous lupus, Sjogren’s syndrome, scleroderma, dermatomyositis, polymyositis, rheumatoid arthritis, juvenile arthritis, polymyalgia rheumatica, reactive arthritis, psoriatic arthropathy, ankylosing spondylitis, undifferentiated spondyloarthropathy.
- Gastrointestinal disorders: Crohn’s disease, ulcerative colitis, ulcerative proctitis, celiac disease.
- Metabolic diseases: Autoimmune thyroiditis, Grave's or Basedow’s disease, Hashimoto thyroiditis, Diabetes Mellitus Type 1, Addison’s disease.
- Skin disorders: Psoriasis, vitiligo, Raynaud’s phenomenon, erythema nodosum, autoimmune bullous skin diseases.
- Others: Auto-immune hemolytic anemia, idiopathic thrombocytopenic purpura, antiphospholipid syndrome, vasculitis, pernicious anemia, autoimmune hepatitis, primary biliary cirrhosis, primary sclerosing cholangitis, autoimmune glomerulonephritis, autoimmune uveitis, autoimmune myocarditis, sarcoidosis, Stevens-Johnson syndrome.

8.4.1.3. Rashes and mucocutaneous lesions

Occurrence of rashes and mucocutaneous lesions that occur within 30 days of vaccination in the first 200 subjects enrolled at each site in the 6 to 12 weeks age category will be reported as AEs/SAEs. Medical documentation of the events will be reported in the CRF/eCRF. Rashes and mucocutaneous lesions that meet the criteria for an SAE are reported in all subjects throughout the study period as usual.

Amended 26 November 2009

8.4.2. Solicited adverse events

Solicited adverse events for the primary immunization course and boost will be collected on the first 200 subjects enrolled in each age category at each site.

Solicited local (injection site) AEs

- Pain at injection site
- Swelling at injection site
- Redness at injection site

Solicited general AEs

- Drowsiness
- Fever
- Irritability/Fussiness
- Loss of appetite

N.B. Temperature will be recorded daily. Should additional temperature measurements be performed at other times of day, the highest temperature will be recorded.

Amended 26 November 2009

8.5. Evaluating adverse events and serious adverse events

8.5.1. Assessment of intensity

Intensity of the following AEs will be assessed as described:

Table 29 Intensity scales for solicited symptoms in infants/toddlers and children less than 6 years of age

Adverse Event	Intensity grade	Parameter
Pain at injection site	0	Absent
	1	Minor reaction to touch
	2	Cries/protests on touch
	3	Cries when limb is moved/spontaneously painful
Swelling at injection site		Record greatest surface diameter in mm
Redness at injection site		Record greatest surface diameter in mm
Fever*		Record temperature in °C
Irritability/Fussiness	0	Behavior as usual
	1	Crying more than usual/ no effect on normal activity
	2	Crying more than usual/ interferes with normal activity
	3	Crying that cannot be comforted/ prevents normal activity
Drowsiness	0	Behavior as usual
	1	Drowsiness easily tolerated
	2	Drowsiness that interferes with normal activity
	3	Drowsiness that prevents normal activity
Loss of appetite	0	Appetite as usual
	1	Eating less than usual/ no effect on normal activity
	2	Eating less than usual/ interferes with normal activity
	3	Not eating at all

*Fever is defined as axillary temperature ≥37.5°C

The maximum intensity of local injection site redness/swelling will be scored at GSK Biologicals as follows:

0	:	None
1	:	< 5 mm
2	:	5 to 20 mm
3	:	> 20 mm

The investigator will make an assessment of the maximum intensity that occurred over the duration of the event for all other AEs, i.e. unsolicited symptoms, including SAEs reported during the study. The assessment will be based on the investigator's clinical judgment.

The intensity of each AE and SAE recorded in the CRF/eCRF or SAE Report Form, as applicable, should be assigned to one of the following categories:

1 (mild)	=	An AE which is easily tolerated by the subject, causing minimal discomfort and not interfering with everyday activities.
2 (moderate)	=	An AE which is sufficiently discomforting to interfere with normal everyday activities.
3 (severe)	=	An AE which prevents normal, everyday activities. (In a young child, such an AE would, for example, prevent attendance at school/ kindergarten/ a day-care centre and would cause the parents/ guardians to seek medical advice. In adults/ adolescents, such an AE would, for example, prevent attendance at work/ school and would necessitate the administration of corrective therapy.)

An AE that is assessed as Grade 3 (severe) should not be confused with a SAE. Grade 3 is a category utilized for rating the intensity of an event; and both AEs and SAEs can be assessed as Grade 3. An event is defined as 'serious' when it meets one of the pre-defined outcomes as described in Section 8.2.

8.5.2. Assessment of causality

The investigator is obligated to assess the relationship between investigational product and the occurrence of each AE/SAE. The investigator will use clinical judgment to determine the relationship. Alternative causes, such as natural history of the underlying diseases, concomitant therapy, other risk factors and the temporal relationship of the event to the investigational product will be considered and investigated. The investigator will also consult the Investigator Brochure and/or Product Information, for marketed products, in the determination of his/her assessment.

There may be situations when a SAE has occurred and the investigator has minimal information to include in the initial report to GSK Biologicals. However, it is very important that the investigator always makes an assessment of causality for every event

prior to submission of the SAE to GSK Biologicals. The investigator may change his/her opinion of causality in light of follow-up information, amending the SAE information accordingly. The causality assessment is one of the criteria used when determining regulatory reporting requirements.

In case of concomitant administration of multiple vaccines, it may not be possible to determine the causal relationship of general AEs to the individual vaccines administered. The investigator should, therefore, assess whether the AE could be causally related to vaccination rather than to the individual vaccines.

All solicited local (injection site) reactions will be considered causally related to vaccination. Causality of all other AEs should be assessed by the investigator using the following question:

Is there a reasonable possibility that the AE may have been caused by the investigational product?

NO	:	The AE is not causally related to administration of the study vaccine(s). There are other, more likely causes and administration of the study vaccine(s) is not suspected to have contributed to the AE.
YES	:	There is a reasonable possibility that the vaccine(s) contributed to the AE.

Non-serious and serious AEs will be evaluated as two distinct events. If an event meets the criteria to be determined “serious” (see Section 8.2 for definition of serious adverse event), it will be examined by the investigator to the extent to be able to determine ALL contributing factors applicable to each serious adverse event.

Other possible contributors include:

- Medical history
- Other medication
- Protocol required procedure
- Other procedure not required by the protocol
- Lack of efficacy of the vaccine(s), if applicable
- Erroneous administration
- Other cause (specify).

8.6. Follow-up of adverse events and serious adverse events and assessment of outcome

After the initial AE/SAE report, the investigator is required to proactively follow each subject and provide further information to GSK Biologicals on the subject’s condition.

All AEs and SAEs documented at a previous visit/contact and designated as not recovered/not resolved or recovering/resolving will be reviewed at subsequent visits/contacts.

Investigators will follow-up subjects:

- with SAEs or subjects withdrawn from the study as a result of an AE, until the event has resolved, subsided, stabilized, disappeared, the event is otherwise explained, or the subject is lost to follow-up;
- or, in the case of other non-serious AEs, until they complete the study or they are lost to follow-up.

Clinically significant laboratory abnormalities will be followed up until they have returned to normal, or a satisfactory explanation has been provided. Additional information (including but not limited to laboratory results) relative to the subsequent course of such an abnormality noted for any subject must be made available.

GSK Biologicals may request that the investigator perform or arrange for the conduct of supplemental measurements and/or evaluations to elucidate as fully as possible the nature and/or causality of the AE or SAE. The investigator is obliged to assist. If a subject dies during participation in the study or during a recognized follow-up period, GSK Biologicals will be provided with a copy of any available post-mortem findings, including histopathology, and any verbal autopsy assessment.

New or updated information will be recorded on the originally completed SAE Report Form. All changes on the SAE Report Form should be signed and dated by the investigator. The updated SAE Report Form should be resent to GSK Biologicals within 24 hours of receipt of the follow-up information as outlined in Section 8.7.1.

Outcome of any non-serious AE occurring within 30 days post-vaccination (i.e. unsolicited AE) or any SAE reported during the entire study will be assessed as:

- Recovered/resolved
- Not recovered/not resolved
- Recovering/resolving
- Recovered with sequelae/resolved with sequelae
- Fatal (SAEs only).

8.7. Prompt reporting of serious adverse events to GSK Biologicals

8.7.1. Time frames for submitting serious adverse event reports to GSK Biologicals

8.7.1.1. For centers using paper CRF

SAEs will be reported promptly to GSK once the investigator determines that the event meets the protocol definition of an SAE. The investigator or designate will fax or email the SAE reports to GSK Biologicals' Study Contact for Serious Adverse Event Reporting **WITHIN 24 HOURS OF HIS/HER BECOMING AWARE OF THESE EVENTS**. Additional or follow-up information relating to the initial SAE report is also to be reported to the GSK Biologicals' Study Contact for Serious Adverse Event Reporting within 24 hours of receipt of such information. This is intended to refer to events that are, in the opinion of the principal investigator, clinically important updates in the deterioration of a child's clinical status, or to more precisely define a diagnosis.

8.7.1.2. For centers using RDE with eCRF

SAEs will be reported promptly to GSK once the investigator determines that the event meets the protocol definition of an SAE. The investigator will complete and submit relevant information on the SAEs in the SAE screens in eCRF **WITHIN 24 HOURS OF HIS/HER BECOMING AWARE OF THESE EVENTS**. Additional or follow-up information relating to the initial SAE report is also to be completed and submitted in the SAE screens in eCRF within 24 hours of receipt of such information.

When paper SAE Report Form is used as back-up system (if electronic SAE reporting system does not work or after freezing of the subject's eCRF), the investigator will fax or email the SAE reports to GSK Biologicals' Central Safety for Serious Adverse Event Reporting. During the study, the investigator should update the SAE screens in eCRF once the electronic system is working again (and not later than 24 hours) and before using it to report additional information.

8.7.2. Completion and transmission of serious adverse event reports to GSK Biologicals

8.7.2.1. For centers using paper CRF

Once an investigator becomes aware that a SAE has occurred in a study subject, she/he will report the information to GSK within 24 hours as outlined in Section 8.7.1. The SAE Report Form will always be completed as thoroughly as possible with all available details of the event, signed by the investigator (or designee), and forwarded to GSK within the designated time frames. If the investigator does not have all information regarding an SAE, he/she will not wait to receive additional information before notifying GSK of the event and completing the form. The form will be updated when additional information is received and forwarded to GSK **WITHIN 24 HOURS** as outlined in Section 8.7.1.

The investigator will always provide an assessment of causality at the time of the initial report as described in Section 8.5.2.

Facsimile (Fax) or electronic transmission of the SAE Report Form are the preferred methods to transmit this information to the Study Contact for Reporting SAEs. In rare circumstances and in the absence of facsimile equipment or electronic connection, notification by telephone is acceptable, with a copy of the SAE Report Form to follow. Initial notification via the telephone does not replace the need for the investigator to complete and sign the SAE Report Form within 24 hours as outlined in Section 8.7.1.

In the event of a death determined by the investigator to be related to vaccination, sending of the fax/electronic transmission must be accompanied by telephone call to the Study Contact for Reporting SAEs.

Please see Sponsor Information Sheet for contact details of who to contact in the event of an SAE.

GSK Biologicals Clinical Safety Physician<br>(Back-up Study contact for reporting SAEs)
**GSK Biologicals Clinical Safety & Pharmacovigilance**<br><br>**Fax:** [redacted] **or** [redacted]<br><br>**24/24 hour and 7/7 day availability**

Amended 26 November 2009
Amended 01 December 2010

8.7.2.2. For centers using RDE with eCRF

Once an investigator becomes aware that a SAE has occurred in a study subject, the investigator will complete and submit the information in the SAE screens in eCRF within 24 hours as outlined in Section 8.7.1. The SAE screens in eCRF will always be completed as thoroughly as possible with all available details of the event and will be submitted by the investigator. If the investigator does not have all information regarding an SAE, he/she will not wait to receive additional information before notifying GSK of the event and completing the SAE screens in eCRF. The SAE screens in eCRF should be updated when additional information is received **WITHIN 24 HOURS** as outlined in Section 8.7.1.

The investigator will always provide an assessment of causality at the time of the initial report as described in Section 8.5.2.

When paper SAE Report Form is used as back-up system (if electronic SAE reporting system does not work or after freezing of the subject’s eCRF), the investigator will report relevant information on SAEs to GSK within the 24 hours as outlined in Section 8.7.1. The SAE Report Form will always be completed as thoroughly as possible with all available details of the event, signed by the investigator, and forwarded to GSK within the designated time frames. If the investigator does not have all information regarding an

SAE, he/she will not wait to receive additional information before notifying GSK of the event and completing the form. When occurring during the study period, the investigator should update the SAE screens in eCRF once the electronic system is working again (and not later than 24 hours). When additional information is received on a SAE reported to GSK using the back-up paper SAE Report Form during the study period, the electronic system should be used to report the additional information **WITHIN 24 HOURS** if the electronic system is working again and only after updating the SAE screens in eCRF once the electronic system was working again.

When additional information is received on a SAE after freezing of the subject's eCRF, new or updated information is to be recorded on the paper SAE Report Form, with all changes signed and dated by the investigator. The updated SAE Report Form should be resent to GSK Biologicals WITHIN 24 HOURS of receipt of the follow-up information as outlined in Section 8.7.1.

In rare circumstances, if the electronic system for reporting SAEs does not work and in the absence of facsimile equipment, notification by telephone is acceptable, with a copy of the SAE Report Form to follow by email or by mail. Initial notification via the telephone does not replace the need for the investigator to complete and submit SAE screens in the eCRF (or complete and sign the SAE Report Form if back-up system need to be used) as outlined in Section 8.7.1.

In the event of a death determined by the investigator to be related to vaccination, completion of SAE screens in the eCRF / sending of the fax (if electronic SAE reporting system does not work or after freezing of the subject’s eCRF) must be accompanied by telephone call to the Study Contact for Reporting SAEs.

Please see Sponsor Information Sheet for contact details of who to contact in the event of an SAE.

**GSK Biologicals Clinical Safety Physician (Back-up Study contact for reporting SAEs)**
**GSK Biologicals Clinical Safety & Pharmacovigilance**<br><br>**Fax:** [redacted] **or** [redacted]<br><br>**24/24 hour and 7/7 day availability**

Amended 26 November 2009
Amended 01 December 2010

8.8. Regulatory reporting requirements for serious adverse events

The investigator will promptly report all SAEs to GSK in accordance with the procedures detailed in Section 8.7. GSK Biologicals has a legal responsibility to promptly notify, as appropriate, both the local regulatory authority and other regulatory agencies about the safety of a product under clinical investigation. Prompt notification of SAEs by the

investigator to the Study Contact for Reporting SAEs is essential so that legal obligations and ethical responsibilities towards the safety of other subjects are met.

The investigator, or responsible person according to local requirements, will comply with the applicable local regulatory requirements related to the reporting of SAEs to the IRB/IEC and, if required, to the applicable government authority.

Investigator safety reports are prepared according to the current GSK policy and are forwarded to investigators as necessary. An investigator safety report is prepared for an SAE(s) that is both attributable to investigational product and unexpected. The purpose of the report is to fulfill specific regulatory and Good Clinical Practice (GCP) requirements, regarding the product under investigation.

An investigator who receives an investigator safety report describing an SAE(s) or other specific safety information (e.g. summary or listing of SAEs) from GSK Biologicals will file it with the Investigator Brochure or other appropriate study documentation and will notify the IRB or IEC, if appropriate according to local requirements.

8.9. Post-study adverse events and serious adverse events

A post-study AE/SAE is defined as any event that occurs outside of the AE/SAE detection period defined in Section 8.4. Investigators are not obligated to actively seek AEs or SAEs in former study participants.

However, if the investigator learns of any SAE, including a death, at any time after a subject has been discharged from the study, and he/she considers the event reasonably related to the investigational product, the investigator will promptly notify the Study Contact for Reporting SAEs.

At centers using RDE with eCRF, after freezing of the subject’s eCRF, if SAE follow-ups or new SAEs have to be reported, the investigators or designate should use paper SAE Report Forms and the fax/email system.

8.10. Pregnancy

Not applicable.

8.11. Treatment of adverse events

Treatment of any adverse event is at the sole discretion of the investigator and according to current good medical practice. Any medication meeting the criteria listed in Section 6.9 administered for the treatment of an AE should be recorded in the subject’s CRF/eCRF.

9. SUBJECT COMPLETION AND WITHDRAWAL

9.1. Subject completion

A subject who returns for the concluding visit/is available for the concluding contact foreseen in the protocol is considered to have completed the study.

9.2. Subject withdrawal

Subjects who are withdrawn because of AEs must be clearly distinguished from subjects who are withdrawn for other reasons. Investigators will follow subjects who are withdrawn as result of a SAE/AE until resolution of the event (see Section 8.6). Further obligations for withdrawn subjects are given in Section 5.1.4.6.

Withdrawals will not be replaced.

9.2.1. Subject withdrawal from the study

From an analysis perspective, a ‘withdrawal’ from the study is any subject who did not come back for the concluding visit/was not available for the concluding contact foreseen in the protocol.

A subject qualifies as a ‘withdrawal’ from the study when no study procedure has occurred, no follow-up has been performed and no further information has been collected for this subject from the date of withdrawal/last contact.

Investigators will make an attempt to contact those subjects who do not return for scheduled visits or follow-up.

Information relative to the withdrawal will be documented on the Study Conclusion page of the CRF/eCRF. The investigator will document whether the decision to withdraw from the study was made by the subject/ the subject’s parent or guardian or the investigator and which of the following possible reasons was responsible for withdrawal:

- death
- serious adverse event
- non-serious adverse event
- protocol violation (specify)
- consent withdrawal, not due to an adverse event
- moved from the study area
- lost to follow-up
- other (specify).

9.2.2. Subject withdrawal from investigational product

A 'withdrawal' from the investigational product is any subject who does not receive the complete treatment, i.e. when no further planned dose is administered from the date of withdrawal. A subject withdrawn from the investigational product may not necessarily be withdrawn from the study as further study procedures or follow-up may be performed (safety or immunogenicity) if planned in the study protocol.

Information relative to premature discontinuation of the investigational product will be documented on the Vaccine Administration page of the CRF/eCRF. The investigator will document whether the decision to discontinue further vaccination/treatment was made by the subject/ the subject's parent or guardian or the investigator and which of the following possible reasons was responsible for withdrawal:

- serious adverse event,
- non-serious adverse event,
- other (specify).

9.3. Screen and Baseline Failures

In all subjects who sign a consent form and subsequently are found to be ineligible for the trial or who withdraw prior to study start the reason the reason for exclusion will be captured in the screening booklet of the CRF/eCRF.

10. DATA EVALUATION: CRITERIA FOR EVALUATION OF OBJECTIVES

10.1. Primary endpoints

10.1.1. Efficacy against clinical malaria disease when primary immunization starts at 6-12 weeks, or 5-17 months of age

- For a primary schedule, the occurrence of cases of malaria meeting the primary case definition for clinical malaria disease over a period starting 14 days post Dose 3 (Visit 4 +14 days) for 12 months in children aged 6-12 weeks at the time of Dose 1, or until the time at which 450 subjects experience a case, whichever occurs later. If 450 are not accumulated by the time of boost (Visit 22), the analysis will proceed.
- For a primary schedule, the occurrence of cases of malaria meeting the primary case definition for clinical malaria disease over a period starting 14 days post Dose 3 (Visit 4 +14 days) for 12 months in children aged 5-17 months at the time of Dose 1, or until the time at which 450 subjects experience a case, whichever occurs later. If 450 are not accumulated by the time of boost (Visit 22), the analysis will proceed.

Amended 19 August 2008

10.2. Secondary endpoints

10.2.1. Efficacy secondary endpoints

10.2.1.1. Efficacy against severe malaria disease

- For a primary schedule (pooled with and without a boost), the occurrence of severe malaria disease meeting the primary and secondary case definitions up to the time at which 250 subjects are diagnosed with severe malaria meeting the primary case definition.
- For a primary schedule with and without a boost, the occurrence of severe malaria disease meeting the primary and secondary case definitions analyzed over the time periods starting 14 days post Dose 3 (Visit 4 +14 days) until boost (Visit 22), boost (Visit 22) until study end (Visit 34) and 14 days post Dose 3 (Visit 4 +14 days) until study end (Visit 34).

10.2.1.2. Efficacy against incident severe anemia and malaria hospitalization

- For a primary schedule with and without a boost, the occurrence of incident severe anemia and malaria hospitalization meeting the primary and secondary case definitions analyzed over the time periods starting 14 days post Dose 3 (Visit 4 +14 days) until boost (Visit 22), boost (Visit 22) until study end (Visit 34) and 14 days post Dose 3 (Visit 4 +14 days) until study end (Visit 34).

10.2.1.3. Efficacy; duration of efficacy of a primary course

- For a primary schedule without a boost, the occurrence of clinical malaria disease meeting the primary case definition analyzed over the time periods starting 14 days post Dose 3 (Visit 4 +14 days) until boost (Visit 22), boost (Visit 22) until study end (Visit 34) and 14 days post Dose 3 (Visit 4 +14 days) until study end (Visit 34).

10.2.1.4. Efficacy; role of a booster

- For a primary schedule with and without a boost, the occurrence of clinical malaria disease meeting the primary case definition analyzed over the time period starting at boost (Visit 22) until study end (Visit 34).

10.2.1.5. Efficacy under different transmission settings

- For each site, for a primary schedule with and without a boost, the occurrence of clinical malaria disease meeting the primary case definition analyzed over the time periods starting 14 days post Dose 3 (Visit 4 +14 days) until boost (Visit 22), boost (Visit 22) until study end (Visit 34) and 14 days post Dose 3 (Visit 4 +14 days) until study end (Visit 34).

10.2.1.6. Efficacy against secondary case definitions of clinical malaria disease

- For a primary schedule with and without a boost, the occurrence of clinical malaria disease meeting the secondary case definitions analyzed over the time periods starting 14 days post Dose 3 (Visit 4 +14 days) until boost (Visit 22), boost (Visit 22) until study end (Visit 34) and 14 days post Dose 3 (Visit 4 +14 days) until study end (Visit 34).

10.2.1.7. Efficacy against prevalence of parasitemia

- For a primary schedule without a boost, the presence of parasitemia at 18 months (Visit 22) and 30 months (Visit 34) after a primary schedule.
- For a booster schedule, the presence of parasitemia 12 months after a boost (Visit 34).

10.2.1.8. Efficacy against prevalence of moderate and severe anemia

- For a primary schedule without a boost, the presence of moderate and severe anemia at 18 months (Visit 22) and 30 months (Visit 34) after a primary schedule.
- For a booster schedule, the presence of moderate and severe anemia 12 months after a boost (Visit 34).

10.2.2. Safety secondary endpoints

10.2.2.1. Safety of a primary course

- For each age category, for a primary schedule, the occurrence of SAEs from Dose 1 (Visit 2) until 14 months post Dose 1 (Visit 16).
- For each age category, for a primary schedule, the occurrence of SAEs over a 30-day follow-up period (day of vaccination and 29 subsequent days) after each vaccination.

Amended 26 November 2009

- For each age category, for a primary schedule without boost, the occurrence of SAEs analyzed over the time periods starting at Dose 1 (Visit 2) until boost (Visit 22), boost (Visit 22) until study end (Visit 34) and Dose 1 (Visit 2) until study end (Visit 34).
- For each age category, for a primary schedule, the occurrence of solicited symptoms over a 7-day follow-up period (day of vaccination and 6 subsequent days) after each vaccination.
- For each age category, for a primary schedule, the occurrence of all unsolicited symptoms over a 30-day follow-up period (day of vaccination and 29 subsequent days) after each vaccination.

Amended 26 November 2009

- For each age category, for a primary schedule, the occurrence of unsolicited symptoms related to vaccination or leading to withdrawal over a 30-day follow-up period (day of vaccination and 29 subsequent days) after each vaccination.

10.2.2.2. Safety of a booster dose

- For each age category, for a booster dose, the occurrence of SAEs analyzed over the time period starting at boost (Visit 22) until study end (Visit 34).
- For each age category, for a booster dose, the occurrence of SAEs over a 30-day follow-up period (day of vaccination and 29 subsequent days) after boost.

Amended 26 November 2009

- For each age category, for a booster dose, the occurrence of solicited symptoms over a 7-day follow-up period (day of vaccination and 6 subsequent days) after the boost.
- For each age category, for a booster dose, the occurrence of all unsolicited symptoms over a 30-day follow-up period (day of vaccination and 29 subsequent days) after each vaccination.

Amended 26 November 2009

- For each age category, for a booster dose, the occurrence of unsolicited symptoms over a 30-day follow-up period (day of vaccination and 29 subsequent days) after the boost.

10.2.3. Immunogenicity secondary endpoints

10.2.3.1. Immunogenicity of a primary course

- For each age category, for a primary schedule without a boost, the anti-CS antibody titers at screening, 1 month post Dose 3 (Visit 5), 18 months post Dose 3 (Visit 22) and 30 months post Dose 3 (Visit 34).
- For each age category, for a primary schedule without a boost, the anti-HBs antibody titers at screening, 1 month post Dose 3 (Visit 5), 18 months post Dose 3 (Visit 22) and 30 months post Dose 3 (Visit 34).

10.2.3.2. Immunogenicity of a booster dose

- For each age category, for a booster schedule, the anti-CS antibody titers at boost (Visit 22), 1 month post boost (Visit 23), 12 months post boost (Visit 34).
- For each age category, for a booster schedule, the anti-HBs antibody titers at boost (Visit 22), 1 month post boost (Visit 23), 12 months post boost (Visit 34).

Amended 26 November 2009

10.2.3.3. Immunogenicity of polio serotypes 1, 2 and 3

- To analyze in a subset of African children whose age at first dose will be 6-12 weeks the percentage of subjects with seroprotective levels of anti-polio 1, 2, 3 antibodies when OPV is given on a primary schedule co-administered with DTPwHepB/Hib (Tritanrix HepB/Hib) with or without RTS,S/AS01E at one month post dose 3 (Visit 5).
- To analyze in a subset of African children whose age at first dose will be 6-12 weeks the percentage of subjects with seroprotective levels of anti-polio 1, 2, 3 antibodies when OPV is given on a primary schedule (co-administered with DTPwHepB/Hib with or without RTS,S/AS01E) and a booster dose at 18 months post primary (with or without RTS,S/AS01E) at one month post boost (Visit 23).

Amended 19 August 2008
Amended 24 October 2008

10.3. Additional Secondary endpoints

Amended 19 August 2008

10.3.1. Additional efficacy secondary endpoints

Amended 19 August 2008

10.3.1.1. Efficacy against other serious illness

- For a primary schedule with and without a boost, the occurrence of other serious illness meeting the primary and secondary case definitions analyzed over the time periods starting 14 days post Dose 3 (Visit 4 + 14 days) until boost (Visit 22), boost (Visit 22) until study end (Visit 34) and 14 days post Dose 3 (Visit 4 + 14 days) until study end (Visit 34). Other serious illness is all medical hospitalization, sepsis and pneumonia.

10.3.1.2. Efficacy against fatal malaria and all-cause mortality

- For a primary schedule with and without a boost, the occurrence of fatal malaria (meeting the case definitions) and all-cause mortality analyzed over the time periods starting 14 days post Dose 3 (Visit 4 +14 days) until study end (Visit 34).

10.3.1.3. Effect on growth

- For a primary schedule with and without a boost, to compare the height/length for age z-score at study end (Visit 34).
- For a primary schedule with and without a boost, to compare the weight for age z-score at study end (Visit 34).

- For a primary schedule with and without a boost, to compare the mid upper arm circumference for age z-score at study end (Visit 34).
- For children 6-12 weeks at first vaccination, for a primary schedule with and without boost, to compare the absolute height at study end (Visit 34).

Amended 26 November 2009

10.3.1.4. Gender-specific efficacy

- In male and female children, for a primary schedule with and without a boost the occurrence of clinical malaria disease meeting the primary case definition analyzed over the time periods starting 14 days post Dose 3 (Visit 4 +14 days) until boost (Visit 22), boost (Visit 22) until study end (Visit 34) and 14 days post Dose 3 (Visit 4 +14 days) until study end (Visit 34).

If important differences in efficacy are observed between male and female children, then all primary and secondary immunological and efficacy endpoints will be presented for male and female children.

10.3.2. Additional immunogenicity secondary endpoint

Amended 19 August 2008

10.3.2.1. Immunological correlates of protection

- In cases and non-cases of malaria disease, CS-antibody titers.

10.3.3. Additional safety and immunogenicity secondary endpoints

Amended 19 August 2008

10.3.3.1. Safety and immunogenicity in 'special' subgroups

Note: HIV testing is not a trial procedure. This analysis will include all children known to be HIV-infected at enrollment or subsequently diagnosed

- In HIV-infected children, for each age category, for a primary schedule with and without a boost, the occurrence of SAEs analyzed over the time periods starting at Dose 1 (Visit 2) until boost (Visit 22), boost (Visit 22) until study end (Visit 34) and Dose 1 (Visit 2) until study end (Visit 34).
- In HIV-infected children, for each age category, for a primary schedule and a booster dose, the occurrence of unsolicited symptoms related to vaccination or leading to withdrawal over a 30-day follow-up period (day of vaccination and 29 subsequent days) after each vaccination.
- In HIV-infected children, for each age category, for a primary schedule with and without a boost, the anti-CS antibody titers at screening, 1 month post Dose 3 (Visit 5), 18 months post Dose 3 (Visit 22) and 30 months post Dose 3 (Visit 34).

- In HIV-infected children for each age category, for a primary schedule with and without a boost, the anti-HBs antibody titers at screening, 1 month post Dose 3 (Visit 5), 18 months post Dose 3 (Visit 22) and 30 months post Dose 3 (Visit 34).
- In low weight for age children (weight for age z-score ≤-2) and in very low weight for age children (weight for age z-score ≤-3), for each age category, for a primary schedule with and without a boost, the occurrence of SAEs analyzed over the time periods starting at Dose 1 (Visit 2) until boost (Visit 22), boost (Visit 22) until study end (Visit 34) and Dose 1 (Visit 2) until study end (Visit 34).
- In low weight for age children (weight for age z-score ≤-2) and in very low weight for age children (weight for age z-score ≤-3), for each age category, for a primary schedule and a boost, the occurrence of unsolicited symptoms related to vaccination or leading to withdrawal over a 30-day follow-up period (day of vaccination and 29 subsequent days) after each vaccination.
- In low weight for age children (weight for age z-score ≤-2) and in very low weight for age children (weight for age z-score ≤-3), for each age category, for a primary schedule with and without a boost, the anti-CS antibody titers at screening, 1 month post Dose 3 (Visit 5), 18 months post Dose 3 (Visit 22) and 30 months post Dose 3 (Visit 34).
- In low weight for age children (weight for age z-score ≤-2) and in very low weight for age children (weight for age z-score ≤-3), for each age category, for a primary schedule with and without a boost, the anti-HBs antibody titers at screening, 1 month post Dose 3 (Visit 5), 18 months post Dose 3 (Visit 22) and 30 months post Dose 3 (Visit 34).

10.4. Additional secondary endpoints for the extension

Efficacy

- In each of at least three study centers, the occurence of clinical malaria disease meeting the primary and secondary case definitions for clinical malaria over a period starting 14 days post Dose 3 until the last visit of the extension.
- Pooled across at least three study centers, the occurrence of severe malaria disease meeting the primary and secondary case definitions for severe malaria over annual time periods.
- Pooled across all participating study centers, the occurrence of severe malaria disease meeting the primary and secondary case definitions for severe malaria over a period starting 14 days post Dose 3 until the last visit of the extension.
- Pooled across all participating study centers, the occurrence of malaria hospitalization meeting the primary and secondary case definitions for malaria hospitalization over a period starting 14 days post Dose 3 until the last visit of the extension.
- Pooled across all participating study centers, the presence of parasitemia at annual timepoints during the extension.

- Pooled across all participating study centers, the presence of moderate and severe anemia at annual timepoints during the extension.
- Pooled across all participating study centers, the occurrence of all-cause mortality and fatal malaria meeting the primary and secondary case definitions over a period starting 14 days post Dose 3 until the last visit of the extension.
- Pooled across all participating study centers, the occurrence of all-medical hospitalization meeting the primary case definition over a period starting 14 days post Dose 3 until the last visit of the extension.
- Pooled across all participating study centers, the occurrence of pneumonia meeting the primary and secondary case definitions over a period starting 14 days post Dose 3 until the last visit of the extension.
- Pooled across all participating study centers, the occurrence of sepsis meeting the primary case definition over a period starting 14 days post Dose 3 until the last visit of the extension.

Safety

- In all participating study centers, the occurrence of SAEs from Dose 1 (Day 0) until the end of extension.

Immunogenicity

- In a subset of subjects, the anti-CS antibody titers at annual timepoints during the extension.

Effect on growth

- In all subjects at all participating study centers, to compare the height for age z-score at the end of extension.

Relevant efficacy and immunogenicity endpoints will also be analyzed by annual time periods, age category and by site.

Amended 01 December 2010

Note: Abbreviations for vaccination groups

The following abbreviations are used to describe the vaccination groups in the next sections:

R3R: Children to receive 3 doses of RTS,S/AS01E on a 0, 1, 2 schedule with an RTS,S/AS01E booster.

R3C: Children to receive 3 doses of RTS,S/AS01E on a 0, 1, 2 schedule without an RTS,S/AS01E booster.

C3C: Children to receive 3 doses of a comparator vaccine on a 0, 1, 2 schedule with a dose of comparator vaccine at Visit 22.

10.5. Estimated sample size

10.5.1. Sample size and power: primary analysis

10.5.1.1. Co-primary endpoint - Efficacy clinical malaria

The analysis of efficacy of a primary schedule of RTS,S/AS01E will be carried out after at least 6000 subjects have been enrolled in the age category under evaluation and followed for 14½ months.

It is anticipated that enrolment of the 5-17 months age category will be faster than in the infant age category and thus analysis of the two co-primary efficacy endpoints may take place at different times. In order to control the overall alpha-level (5%) of the trial both evaluations will be performed at a 2.5% alpha level (Bonferroni correction) thus, for the evaluation of the co-primary endpoint leading to 97.5% Confidence Intervals (CI).

Assuming at least 5400 evaluable subjects (randomized 2:1), an attack rate in controls of 10/100 cyr (children years at risk) over the follow-up period from 2 weeks post Dose 3 to 1 year post Dose 3 and a true vaccine efficacy of 30%, the study has 90% power to detect a lower limit of the 97.5% CI around estimated VE above 0%.

In the event that the attack rate is lower than anticipated and there have not been 450 accumulated cases in the observation period 14 days to 12 months post dose 3, then the period of observation contributing to the primary analysis will be extended. The analysis will be conducted when 450 cases have accumulated, or until boost (visit 22) (approximately 18 months post dose 3) whichever occurs earlier.

Amended 19 August 2008

10.5.1.2. Secondary endpoint - Safety

All available safety data to Month 14½ will be presented at each of the primary analyses. For a primary course, safety will be evaluated by examining SAEs, unsolicited adverse events related to vaccination or leading to drop out, and on a subset of subjects in each study center, reactogenicity and all unsolicited adverse events.

Amended 26 November 2009

Table 30 gives an overview of the power of the trial to detect statistically significant differences between study groups on the incidence of reported adverse events (2-sided Fisher's exact test, alpha 5%).

Table 30 Power to detect fold-differences in SAE increases at primary analysis at Month 14.5

Evaluable child years at risk in controls			2272
Evaluable child years at risk in vaccines			4543
		increase by factor of	*power*
Frequency of preferred term per year in controls	1/100	1.5	35%
		2.0	87%
	1/500	3.5	67%
		4.5	90%
	1/1000	5.0	67%
		7.0	92%

10.5.2. Sample size and power: secondary analysis

A secondary analysis of severe malaria will be performed when 250 episodes of severe malaria meeting the primary case definition, pooled over the study centers and age categories, have occurred, or at the end of the follow-up period (Month 32), whichever occurs first.

250 episodes give 80% power to detect 30% VE with a lower limit of the 95% CI above 0%. Assuming 50% VE 250 episodes give 90% power to detect a lower limit of the 95% CI above 25%.

10.5.3. Sample size and power: final analyses

10.5.3.1. Efficacy: Evaluation of clinical malaria

Recipients of a primary course of RTS,S/AS01E plus booster will be compared to controls (R3R vs C3C) over the time period starting 14 days post Dose 3 (Visit 4 + 14 days) until study end (Visit 34). Analysis will be performed separately for the 2 age categories (6-12 weeks and 5-17 months).

Assuming 2300 evaluable subjects in each age category per group, 30% VE and an attack rate in controls of 10/100 cyr, at the final analysis at Month 32, the trial has 90% power to detect a lower limit of the 95% CI of the VE estimate above 15%. Attack rate in controls of 20/100 cyr and 30/100 cyr lead to lower limits of 22% and 24% respectively.

10.5.3.2. Efficacy: Evaluation of booster

Recipients of a primary course of RTS,S/AS01E plus booster (R3R vs R3C) will be compared over the time period starting from booster at 18 months post primary schedule until study end at Month 32, pooled over the age categories.

Assuming 4500 evaluable subjects per group and 30% VE for the RTS,S/AS01E booster and 10% VE for the control booster and an attack rate in controls of 10/100 cyr, at the final analysis at Month 32, the trial has 88% power to detect a lower limit of the 95% CI above 0%. Attack rate in controls of 20/100 cyr and 30/100 cyr lead to 99% power to detect a lower limit of the 95% CI above 0%. If VE for controls is 15% the trial has 62%, 91% and 99% power to detect a lower limit of the 95%CI above 0% for attack rates in control of 10/100 cyr, 20/100 cyr and 30/100 cyr respectively.

VE will be estimated in recipients of a primary course of RTS,S/AS01E plus booster compared to recipients of control vaccines over the time period starting from booster (R3R vs C3C), 18 month post primary schedule until study end at Month 32, pooled over the age categories. Assuming 4500 evaluable subjects per group, 30% VE and an attack rate in controls of 10/100 cyr, at the final analysis at Month 32, the trial has 90% power to detect a lower limit of the 95% CI of 12%. Attack rate in controls of 20/100 cyr and 30/100 cyr lead to lower limits of 18% and 21% respectively, and 21% respectively.

10.5.3.3. Evaluation of safety

Safety will be evaluated by examining SAEs, unsolicited adverse events related to vaccination or leading to drop out, and on a subset of subjects in each study center, reactogenicity and all unsolicited adverse events. This will be evaluated after a primary course and after a booster dose, separately for both age categories.

Amended 26 November 2009

Table 31 gives an overview of the power of the trial to detect statistically significant differences between study groups on the incidence of reported adverse events (2-sided Fisher’s exact test, alpha 5%).

Safety will also be evaluated over the time period starting from Dose 1 (Visit 2) until boost (Visit 22) (R3R + R3C vs C3C) and from boost (Visit 22) until study end (Visit 34) (R3R vs C3C and R3C vs C3C).

Table 31 gives an overview of the power of the trial to detect statistically significant differences between study groups on the incidence of reported adverse events (2-sided Fisher’s exact test, alpha 5%).

Table 31 Power to detect fold-differences in SAE increases at final analysis at Month 32

Evaluable child years at risk in controls			5902
Evaluable child years at risk in vaccines			11 805
		increase by factor of	*power*
Frequency of preferred term per year in controls	1/100	1.5	77%
		2.0	99%
	1/500	2.0	53%
		3.0	97%
	1/1000	3.0	71%
		4.0	95%

10.5.4. Sample size and power: extension analysis

10.5.4.1. Efficacy: evaluation of clinical malaria disease

The power of the extension phase to demonstrate an overall benefit of all clinical malaria episodes over the total follow up time for each of the vaccine regimens R3C and R3R compared to control is presented below.

The assumptions were an initial enrolment of at least 1200 subjects at each of the at least three centers, 15% drop out in primary trial phase and 10% drop out in extension phase. Results are presented for varying attack rates (0.2, 0.5 and 1.5 episodes per child per year at risk).

0.2 episodes per child per year at risk:

- 96% power to detect 30% benefit
- 80% power to detect 23% benefit and -25% negative impact

0.5 episodes per child per year at risk:

- 99% power to detect 30% benefit
- 80% power to detect 15% benefit and -16% negative impact

1.5 episodes per child per year at risk:

- 99% power to detect 30% benefit
- 80% power to detect 10% benefit and -9% negative impact

For the endpoint evaluating vaccine efficacy by yearly time periods pooled across the at least 3 participating centers, the study has at least 83 % power to detect 30 % VE in the subsequent year of follow up, assuming an attack rate of 0.7 episodes per child per year at risk.

10.5.4.2. Efficacy: evaluation of severe malaria disease

The power of the study to establish an overall benefit on the total number of severe malaria episodes over the total follow up time is calculated. Data are pooled across vaccination regimens (R3C and R3R), age categories and sites and compared to control. Due to the uncertainty in the attack rate some scenarios are presented.

Assumptions: 16000 enrolled in primary trial phase, 15% drop out in primary trial phase and 10% drop out in extension.

Table 32 Power to detect VE against severe malaria disease

Rate (pyr)	power to detect 30% benefit	80% power to detect benefit of:	80% power to detect negative impact of
0.01	97%	22%	-25%
0.005	80%	30%	-38%

10.5.4.3. Safety: evaluation of uncommon safety events

Extending the trial in all study sites for safety monitoring (SAEs) from Month 32 to end of December 2013 corresponds to a total follow up of approximately 73100 person years (20300 in controls and 40700 in RTS,S recipients) from study start to end of December 2013. The power of the study to detect SAEs with the frequency is shown in the table below.

Table 33 Power to detect SAEs

**Evaluable child years at risk in controls**			**20350**
**Evaluable child years at risk in vaccines**			**40700**
		**increase by factor of**	**Power**
Frequency of preferred term per year in controls	1/100	1.2	60%
		1.3	90%
	1/500	1.7	85%
		1.8	92%
	1/1000	2.0	81%
		2.2	91%

Amended 01 December 2010

10.6. Study cohorts to be evaluated

10.6.1. Intention to Treat (ITT) cohort

The ITT cohort will include all subjects that received at least 1 dose of study vaccine. The analyses on the ITT cohort will be performed per treatment assignment.

10.6.2. According-To-Protocol (ATP) cohort for analysis of immunogenicity

The ATP cohort for immunogenicity will include all subjects included in the ITT that received all vaccinations according to protocol procedures within specified intervals and did not take any immune modifying medication or have blood transfusions.

10.6.3. According To Protocol (ATP) cohort for analysis of efficacy

The ATP cohort for efficacy will include all subjects included in the ITT who received all vaccinations according to protocol procedures within specified intervals.

10.7. Analyses

10.7.1. Covariates

Potential covariates will include bednet usage (refer to Appendix J for methodology for collecting bednet data), distance from nearest inpatient health facility, distance from nearest outpatient health facility, pneumococcal/*Hemophilus* vaccination status, ethnicity, anthropometric measurements and feeding history.

10.7.2. Primary analyses at 14.5 months post Dose 1 (for the first 6000 children in each age category)

Two primary analyses will be performed evaluating the efficacy and safety of RTS,S/AS01E over the first 14½ months of follow-up in the two age categories. In order to maintain blinding of the study, the analysis will be carried out by a statistician external to GSK and the investigator teams.

Amended 01 December 2010

10.7.2.1. Efficacy

The analysis of efficacy of the primary schedule will be carried out when approximately 6000 children in an age category have been enrolled and followed to Month 14½. The primary analysis will be performed on the ATP cohort for efficacy however, an ITT analysis will also be performed.

As a primary analysis, VE will be evaluated taking into account first or only episodes using Cox regression, modeling the time to first or only episode as a function of group assignment (R3R+R3C vs C3C), stratified by study site. VE will be defined as 1 minus Hazard Rate (HR). 95% CI and p-values (Likelihood Ratio test) on VE estimates will be calculated from the Cox model. Primary analysis will be unadjusted for covariates, however both adjusted and unadjusted estimates will be presented.

As there are two co-primary endpoints, for decision making, 97.5% CIs on VE will be calculated using the same methodology, ensuring an overall 2-sided 5% alpha-level.

Secondary analysis on the total number of episodes will also be performed. VE will be estimated from the incidence rate ratio using a negative binomial regression model allowing for interdependence between episodes within the same subject. 95% CI and p-values on VE estimates (R3R+R3C vs C3C), defined as 1 *minus* Rate Ratio will be calculated from this model. To avoid counting the same malaria episode more than once, total time at risk will be adjusted by subtracting 14 days following a malaria episode meeting the malaria case definition under evaluation. Both unadjusted and adjusted estimates of VE will be presented.

Amended 19 August 2008

10.7.2.2. Safety

Safety will be evaluated by examining SAEs, unsolicited adverse events related to vaccination or leading to drop out, and, on a subset of subjects in each study center reactogenicity and all unsolicited adverse events. The analysis of safety will be performed on the ITT cohort.

Amended 26 November 2009

Recipients of a primary course of RTS,S/AS01E will be compared to recipients of control (R3R + R3C vs C3C) over the time period starting from Dose 1 (Visit 2) until 14½ months post Dose 1.

The proportion of subjects with an SAE, classified by the MedDRA preferred term level, will be tabulated with exact 95% CI.

The proportion of subjects with fatal SAEs, classified by the MedDRA preferred term level, will be tabulated with exact 95% CI.

The proportion of subjects reporting SAEs within 30 days following each vaccination will be tabulated with exact 95% CI.

Amended 26 November 2009

The proportion of subjects with at least one report of unsolicited AEs related to vaccination or leading to drop out classified by MedDRA preferred term, reported up to 30 days after each vaccination will be tabulated with exact 95% CI. The same tabulation will be performed for Grade 3 unsolicited AEs.

For seizures occurring within 7 days of vaccination, an analysis will be performed based on the Brighton Collaborations guidelines [Bonhoeffer, 2004]. This includes descriptive tables of the time relationship of seizures to vaccination, the duration of seizures, the level of diagnostic certainty and the relationship to episodes of fever.

For rashes and mucocutaneous diseases within 30 days of vaccination, an analysis will be performed based on the Brighton Collaboration Guidelines [Beigel, 2007].

Amended 26 November 2009

For a subset of subjects in each study site, the percentage of subjects with at least one local AE (solicited and unsolicited), with at least one general AE (solicited and

unsolicited) and with any AE during the solicited follow-up period (7 days) will be tabulated with exact 95% CI after each vaccine dose and overall. The percentage of doses followed by at least one local AE (solicited and unsolicited), by at least one general AE (solicited and unsolicited) and by any AE will be tabulated, overall vaccination course, with exact 95% CI.

For a subset of subjects in each study site, the percentage of subjects reporting each individual solicited local and general AE during the solicited follow-up period (7 days) will be tabulated with exact 95% CI. The percentage of doses followed by each individual solicited local and general AE will be tabulated, overall and by vaccination course, with exact 95% CI.

For all solicited symptoms, the same tabulation will be performed for Grade 3 AEs and for AEs with relationship to vaccination.

10.7.2.3. Immunogenicity

Immunogenicity will be evaluated on a subset of subjects in each study site. Analyses of immunogenicity will be performed separately for both age categories. The primary analysis will be based on the ATP cohort for immunogenicity, however, ITT analyses will also be performed.

The percentage of subjects with seropositive levels of anti-CS (proportion of subjects with anti-CS antibody titers greater than or equal to 0.5 EU/mL) with 95% CI will be determined at each blood sampling timepoint. Antibody titers will be summarized by GMT with 95% CI at all timepoints at which serological samples are taken. Antibody titers after the third dose will also be investigated using reverse cumulative curves.

The seroprotective level for anti-HBs is ≥ 10 mIU/mL. The percentage of subjects with protective levels of anti-HBs (≥ 10 mIU/mL) with 95% CI will be determined at each blood sampling timepoint. Antibody titers will be summarized by GMT with 95% CI at all timepoints at which serological samples are taken. Antibody titers after the third dose will also be investigated using reverse cumulative curves.

10.7.3. Secondary analyses at the time 250 cases of severe malaria have accumulated

In order to maintain blinding of the study, the secondary analysis will be carried out by a statistician external to GSK and the investigator teams.

Amended 01 December 2010

10.7.3.1. Efficacy

VE against severe malaria disease will be evaluated when 250 subjects (both age categories together) have reported an episode of severe malaria meeting the primary case definition. The primary analysis of interest is comparing the pooled RTS,S/AS01E groups (with or without RTS,S/AS01E booster dose) versus controls (R3R+R3C vs

C3C). VE against severe malaria disease will be estimated as 1-RR where RR is the risk ratio (proportion of subjects reporting severe malaria disease in the RTS,S/AS01E group over the proportion in controls) together with 95% CIs.

Multiple episodes of severe malaria are considered secondary and will be analyzed the same way as multiple episodes of clinical malaria, by negative binomial regression allowing for interdependence between episodes within the same subject. 95% CI and p-values on VE estimates will be calculated from this model. 14 days following a malaria episode meeting the severe malaria case definition under evaluation will be subtracted from follow-up time. Both unadjusted and adjusted estimates of VE will be presented.

Amended 19 August 2008

10.7.3.2. Safety

Safety will be evaluated by examining SAEs from study start (Dose 1) up to the time when 250 accumulated episodes have occurred. All available safety data for both age categories will be presented for the ITT cohort. All safety analyses will be performed separately for both age categories.

Recipients of a primary course of RTS,S/AS01E with or without booster will be compared to recipients of control (R3R + R3C vs C3C) over the time period starting from Dose 1 (Visit 2) until 250 accumulated episodes have occurred.

The proportion of subjects with an SAE, classified by the MedDRA preferred term level, will be tabulated with exact 95% CI.

The proportion of subjects with fatal SAEs, classified by the MedDRA preferred term level, will be tabulated with exact 95% CI.

10.7.4. Secondary analysis at Month 20 (Visit 22)

An analysis of safety, efficacy and immunogenicity will be performed when all children have completed 18 months follow-up post Dose 3 (Visit 22). The endpoints including an assessment at Visit 22 are:

- Efficacy against severe malaria disease (Refer to 10.2.1.1)
- Efficacy against incident severe anemia and malaria hospitalization (Refer to 10.2.1.2)
- Efficacy; duration of efficacy of a primary course (Refer to 10.2.1.3)
- Efficacy under different transmission settings (Refer to 10.2.1.5)
- Efficacy against secondary case definitions of clinical malaria disease (Refer to 10.2.1.6)
- Efficacy against prevalence of parasitemia (Refer to 10.2.1.7)
- Efficacy against prevalence of moderate and severe anemia (Refer to 10.2.1.8)

- Efficacy against other serious illness (Refer to 10.3.1.1)
- Safety of a primary course (Refer to 10.2.2.1)
- Immunogenicity of a primary course (Refer to 10.2.3.1)
- Safety and immunogenicity in ‘special’ subgroups (Refer to 10.3.3.1)

The detailed analysis of gender-specific vaccine efficacy (as described in 10.3.1.4) will be reported in full at the end of the study (Visit 34).

Amended 23 January 2012

10.7.5. Secondary analysis at one month post booster (Month 21)

10.7.5.1. Polio serotypes 1, 2 and 3 antibody responses

A secondary analysis of the immune response for polio serotypes 1, 2 and 3 when OPV is given on a primary schedule co-administered with DTPwHepB/Hib (Tritanrix HepB/Hib) with or without RTS,S/AS01E at screening, at one month post primary course (Visit 5) and following a booster dose at 18 months post primary (with or without RTS,S/AS01E) at one month post boost will be performed in a subset of children in the 6 to 12 weeks age category. The seroprotective level for anti-polio 1, 2, 3 is ≥ 8 EU/mL. The percentage of subjects with protective levels of anti-polio 1, 2, 3 antibodies with 95% CI will be determined at one month post primary series (Visit 5) and at one month post booster dose (at Visit 23). Antibody titers will be summarized by GMT with 95% CI. Antibody titers will also be investigated using reverse cumulative curves.

Amended 19 August 2008
Amended 24 October 2008

10.7.6. Final analyses

10.7.6.1. Final analyses - Efficacy

Efficacy; role of a Booster

As a secondary endpoint, the benefit of a booster dose of RTS,S/AS01E given 18 months post primary schedule will be evaluated. These evaluations will be performed pooled across age categories. The first analysis of interest will compare the group that received a primary schedule of RTS,S/AS01E and a booster of RTS,S/AS01E to the group receiving a primary schedule of RTS,S/AS01E and a control booster (R3R vs R3C) over a timeframe starting from the booster dose until study end (at Month 32). First or only episodes will be modeled through a Cox regression model stratified by study site to estimate the HR and its 95% CI as well as the likelihood ratio p-value.

Another analysis, using the methodology outlined above will be performed to estimate VE of the group that received a primary schedule of RTS,S/AS01E and a booster of RTS,S/AS01E (R3R vs C3C) and VE of the group receiving a primary schedule of RTS,S/AS01E and a control booster (R3C vs C3C) over a timeframe starting from the

booster dose until study end (at Month 32). These analyses will be performed for both age categories separately. VE estimates, both adjusted and unadjusted, for first or only episodes as well as for multiple episodes will be presented.

Efficacy; duration of efficacy of a primary course

At final analysis at Month 32, evaluations of VE over the entire follow-up period will be performed using the same methodology as outlined above for first or only episodes and multiple episodes (R3C vs C3C).

Efficacy under different transmission settings

Evaluating the entire follow-up period also allows estimating VE in different transmission settings. The study sites being a proxy for transmission setting, VE estimates by study site will be presented.

10.7.6.2. Final analyses - Safety

Safety will be evaluated by examining SAEs from study start (Dose 1) until study end at Month 32. All safety analyses will be performed separately for both age categories on the ITT cohort.

Recipients of a primary course of RTS,S/AS01E with or without a boost will be compared to recipients of control (R3R + R3C vs C3C) over the time period starting from Dose 1 (Visit 2) until study end (Visit 34). This analysis will be repeated for R3R vs C3C and R3C vs C3C.

Safety will also be evaluated over the time period starting from Dose 1 (Visit 2) until boost (Visit 22) (R3R + R3C vs C3C) and from boost (Visit 22) until study end (Visit 34) (R3R vs C3C and R3C vs C3C).

The proportion of subjects with an SAE, classified by the MedDRA preferred term level, will be tabulated with exact 95% CI.

The proportion of subjects with fatal SAEs, classified by the MedDRA preferred term level, will be tabulated with exact 95% CI.

The proportion of subjects reporting SAEs within 30 days following boost will be tabulated with exact 95% CI.

Amended 26 November 2009

10.7.6.3. Final analyses - Immunogenicity

Immunogenicity will be evaluated on a subset of subjects in each study site. Analyses of immunogenicity will be performed separately for both age categories. The primary analysis will be based on the ATP cohort for immunogenicity, however, ITT analyses will also be performed.

The percentage of subjects with seropositive levels of anti-CS (proportion of subjects with anti-CS antibody titers greater than or equal to 0.5 EU/mL) with 95% CI will be

determined at each blood sampling timepoint. Antibody titers will be summarized by GMT with 95% CI at all timepoints at which serological samples are taken. Antibody titers after the third dose will also be investigated using reverse cumulative curves.

The seroprotective level for anti-HBs is ≥ 10 mIU/mL. The percentage of subjects with protective levels of anti-HBs (≥ 10 mIU/mL) with 95% CI will be determined at each blood sampling timepoint. Antibody titers will be summarized by GMT with 95% CI at all timepoints at which serological samples are taken. Antibody titers after the third dose will also be investigated using reverse cumulative curves.

As a secondary analysis, associations between immune responses and VE will be investigated. Full details will be provided in the statistical analysis plan (SAP).

Amended 19 August 2008
Amended 23 January 2012

10.7.7. Extension analyses

10.7.7.1. Extension analysis – Risk benefits over time

A number of statistical analyses will be performed to assess the risk benefit ratio of the RTS,S/AS01E vaccine over time: safety, all episodes, cases averted and vaccine efficacy against prevalent parasitemia.

- Safety will be evaluated by examining all SAEs and fatal SAEs from study start (Dose 1) until study end of extension (Visit 38). SAEs will be classified by the MedDRA preferred term level and tabulated with exact 95% CI. All safety analyses will be performed on the ITT cohort pooled for age categories. Recipients of a primary course of RTS,S/AS01E with or without a boost will be compared to recipients of control (R3R + R3C vs C3C). This analysis will be repeated for R3R vs C3C and R3C vs C3C.
- All severe disease episodes over time will be presented in all sites. It will be analysed by determining vaccine efficacy (VE) by comparison of multiple episodes over the total follow up time post Dose 3. A negative binomial regression model allowing for interdependence between episodes within the same subject will be used. 95% CI and p-values on VE estimates, defined as 1 minus Rate Ratio will be calculated from this model.
- Cases of severe disease averted over time will be calculated in all sites. The calculation will use observed rates in controls and the estimated VE from the model described above and results will be expressed as the number of severe episodes averted per 1000 children at risk per year (cyr) with 95% CIs. Cumulative cases averted over time will be calculated and expressed as per 1000 cyr with 95% CIs and presented graphically.
- Vaccine efficacy against prevalent parasitemia will be calculated in all sites as relative reduction calculated as 1- (prevalence in RTS,S / prevalence in controls).
- All clinical malaria disease episodes over time will be presented in at least three transmission settings. It will be analysed by determining vaccine efficacy (VE) by

comparison of multiple episodes over the total follow up time post Dose 3. A negative binomial regression model allowing for interdependence between episodes within the same subject will be used. 95% CI and p-values on VE estimates, defined as 1 minus Rate Ratio will be calculated from this model.

- Cases of clinical malaria disease averted over time will be calculated in at least three transmission settings. The calculation will use observed rates in controls and the estimated VE from the model described above and results will be expressed as the number of clinical episodes averted per 1000 children at risk per year (cyr) with 95% CIs. Cumulative cases averted over time will be calculated and expressed as per 1000 cyr with 95% CIs and presented graphically.

10.7.7.2. Additional analyses

For consistency with the analyses presented for the primary trial phase the following analysis will also be conducted.

- Vaccine efficacy against first or only episodes of clinical malaria disease will be assessed using Cox regression models. VE is defined as 1 minus R where R was the hazard ratio of the RTS,S/AS01E group versus control. 95% CI and p-values (Likelihood Ratio test) on VE estimates will be calculated from the Cox model. Cox regression assumes proportional hazards throughout the follow-up period. This assumption will be checked by plotting, per group, the log of the cumulative hazard against the log of time. Under the assumption of proportionality of the hazard, both curves should be parallel. A test based on the Schoenfeld residuals will be performed.
- Vaccine efficacy against at least one episode of severe malaria disease will be estimated as 1-RR where RR is the risk ratio (proportion of subjects reporting severe malaria disease in the RTS,S/AS01E group over the proportion in controls) together with 95% CIs.
- Vaccine efficacy against all clinical episodes will be assessed by breaking down the time at risk in annual periods.
- Prevalent Parasitemia and anemia will be analyzed by 2x2 tables and Fisher exact tests. Mean parasite density and mean Hb values will also be tabulated.

Amended 01 December 2010

11. ADMINISTRATIVE MATTERS

To comply with Good Clinical Practice important administrative obligations relating to investigator responsibilities, monitoring, archiving data, audits, confidentiality and publications must be fulfilled. See Appendix A for details.

12. REFERENCES

References amended 19 August 2008
References amended 26 November 2009

Ajjan N, Soulebot JP, Stellmann C, Biron G, Charbonnier C, Triau R et al. Resultats de la vaccination antirabique preventive par le vaccine inactive concentre souche rabies PM/WI38-1503-3M cultivée sur cellules diploids humaines. Obtention d'immunoglobuline hyperimmune mixte antirabique et antitétanique par plasmaphérèse des étudiants vétérinaires vaccinés. *Dev Biol Stand* 1978; 40: 89-100.

Alloueche A, Milligan P, Conway DJ, Pinder M, Bojang K, Doherty T et al. Protective efficacy of the RTS,S/AS02 *Plasmodium falciparum* malaria vaccine is not strain specific. *American Journal of Tropical Medicine and Hygiene.* 2003; 68: 97-101.

Alonso PL, Sacarlal J, Aponte JJ, Leach A, Macete E et al. Efficacy of the RTS,S/AS02A vaccine against *Plasmodium falciparum* infection and disease in young African children: randomised controlled trial. *The Lancet*. 2004; 364: 1411-1420.

Alonso PL, Sacarlal J, Aponte JJ, Leach A, Macete E et al. Duration of protection with RTS,S/AS02A malaria vaccine in prevention of *Plasmodium falciparum* disease in Mozambican children: single-blind extended follow-up of a randomised controlled trial. *The Lancet.* 2005; 366: 2012-2018.

Aoki FY, Tyrell DAJ, Hill LE. Immunogenicity and acceptability of a human diploid cell culture rabies vaccine in volunteers. *The Lancet*. 1975; 660-2.

Aponte JJ, Aide P, Renom M, Mandomando I, Bassat Q et al. Safety of the RTS,S/AS02D candidate malaria vaccine in infants living in a highly endemic area of Mozambique: a double blind randomised controlled phase I/IIb trial. *Lancet* 2007; 370 (9598); 1543-1551.

Beigel J, Kohl K, Khuri-Bulos N, Bravo L, Nell P et al. for the Brighton Collaboration Rash Working Group. Rash including mucosal involvement: Case definition and guidelines for collection, analysis, and presentation of immunization safety data. Vaccine 2007; 25:5697–5706.

Berkley JA, Mwangi I, Ngetsa CJ, Mwarumba S, Lowe BS et al. Diagnosis of acute bacterial meningitis in children at a district hospital in sub-Saharan Africa. *The Lancet*. 2001: 357; 1753-57.

Bernard KW, Roberts MA, Sumner J, Winkler WG, Mallonee J, Baer GM, Chaney R. Human diploid cell rabies vaccine. *Journal of the American Medical Association*. 1982; 247: 1138-42.

Bingham D, Kendall J, Clancy M. The portable laboratory: an evaluation of the accuracy and reproducibility of i-Stat. *Ann Clin Biochem*. 1999; 36 (4): 66-71.

Bojang KA, Milligan PJM, Pinder M, Vigneron L, Alloueche A, Kester KE et al. Efficacy of RTS,S/AS02 Malaria Vaccine against *Plasmodium falciparum* Infection in Semi-Immune Adult Men in the Gambia: A Randomized Trial. *The Lancet*. 2001; 358: 1927-34.

Bonhoeffer J, Menkes J, Gold, MS, de Souza-Brito G, Fisher MC et al. Generalized convulsive seizure as an adverse event following immunization: case definition and guidelines for data collection, analysis and presentation. *Vaccine*. 2004; 22: 557-562.

Breman JG, Egan A, Keusch GT. Introduction and Summary. The Intolerable Burden of Malaria: A New Look at the Numbers. *American Journal of Tropical Medicine and Hygiene*. 2001 a; 64 (1, 2) S: iv-vii.

Breman, JG. The Ears of the Hippopotamus: Manifestations, Determinants and Estimates of the Malaria Burden. *American Journal of Tropical Medicine and Hygiene* 2001 b; 64: 1-11.

Broome CV, Rugh MA, Yada AA, Giat L et al. Epidemic group C meningococcal meningitis in Upper Volta, 1979. *Bulletin of the World Health Organization* 1983; 61 (2): 325-330.

Costy-Berger F. Vaccination antirabique preventive par du vaccine prepare sur cellules diploids humaines. *Dev Biol Stand* 1978; 40: 101-104.

Cox JH, Schneider LG. Prophylactic immunization of humans against rabies by intradermal inoculation of human diploid cell culture vaccine. *J Clin Microbiol* 1976; 3 (2): 96-101.

Dicko A, Mantel C, Kouriba B, Sagara I, Thera MA, Doumbia S et al. Season, fever prevalence and pyrogenic threshold for malaria disease definition in an endemic area of Mali. *Tropical Medicine and International Health.* 2005; 10 (6): 550-556.

Epstein JE, Charoenvit Y, Kester KE, Wang R, Newcomer R, Fitzpatrick S et al. Safety, tolerability, and antibody responses in humans after sequential immunization with a PfCSP DNA vaccine followed by the recombinant protein vaccine RTS,S/AS02A. *Vaccine.* 2004; 22: 1592–1603.

Gallup JL, Sachs JD. The Economic Burden of Malaria. *American Journal of Tropical Medicine and Hygiene*. 2001; 64: 85-96.

Greenwood B. M., A. K. Bradley, A. M. Greenwood, P. Byass, K. Jammeh, K. Marsh, S. Tulloch, F. S. J. Oldfield and R. Hayes. Mortality and morbidity from malaria among children in a rural area of The Gambia, West Africa. *Transactions of the Royal Society of Tropical Medicine and Hygiene.* 1987;81(3):478-486.

GSK data on file: Safety, Immunogenicity, and Preliminary Efficacy of a Lyophilized Formulation of SmithKline Beecham Biologicals' Candidate Malaria Vaccine RTS,S. Report WRMAL-005. 19 August 1999. Data on File at GlaxoSmithKline Biologicals.

GSK data on file: Dosage and Schedule Optimization of RTS,S Malaria Vaccine. Report WRMAL-004. 30 April 1998. Data on File at GlaxoSmithKline Biologicals.

GSK data on file: Phase IIb controlled randomized double-blind study to evaluate the efficacy, safety and immunogenicity of SmithKline Beecham Biologicals' candidate RTS,S malaria vaccine in semi-immune adult males in The Gambia. Report 257049/005 (Malaria-005) 15 February 2006. Data on File at GlaxoSmithKline Biologicals.

GSK data on file: Phase I/IIa safety, immunogenicity, and preliminary efficacy of two short-course schedules of SmithKline Beecham Biologicals' candidate malaria vaccine RTS,S in non-immune adult volunteers. Report 257049/012 (Malaria-012) 01 March 2002. Data on File at GlaxoSmithKline Biologicals.

GSK data on file: A Phase I Double Blind, Randomized, Controlled, Staggered Study to Evaluate the Safety, Reactogenicity and Immunogenicity of 1/5, 1/2 and Full Doses of GlaxoSmithKline Biologicals' RTS,S/AS02 Candidate Malaria Vaccine, Administered IM According to a 0, 1, 3-month Vaccination Schedule in Semi-Immune Children aged 6 to 11 Years in The Gambia, a Malaria Endemic Region. Report 257049/015 (Malaria-015) 01 December 2003. Data on File at GlaxoSmithKline Biologicals.

GSK data on file: Phase II b controlled randomized double-blind study to evaluate the efficacy, safety and immunogenicity of GlaxoSmithKline Biologicals' candidate RTS,S malaria vaccine in semi-immune adult males in The Gambia. Report 257049/016, 257049/017 & 257049/018 (Malaria-016, -017 & -018) 12 January 2005. Data on File at GlaxoSmithKline Biologicals.

GSK data on file: A Phase I Double Blind, Randomized, Controlled, Staggered Study to Evaluate the Safety, Reactogenicity and Immunogenicity of 1/5, 1/2 and Full Doses of GlaxoSmithKline Biologicals' RTS,S/AS02 Candidate Malaria Vaccine, Administered IM According to a 0, 1, 3-month Vaccination Schedule in Semi-Immune Children aged 1 to 5 Years in The Gambia, a Malaria Endemic Region. Report 257049/020 (Malaria-020) 27 February 2003. Data on File at GlaxoSmithKline Biologicals.

GSK data on file: A Phase I Double Blind, Randomized, Controlled, Staggered Study to Evaluate the Safety, Reactogenicity and Immunogenicity of GlaxoSmithKline Biologicals' RTS,S/AS02 Candidate Malaria Vaccine, Administered IM According to a 0, 1, 2-month Vaccination Schedule in Children aged 1 through 4 years in Mozambique, a Malaria Endemic Region. Report 257049/025 (Malaria-025) 10 October 2003. Data on File at GlaxoSmithKline Biologicals.

GSK data on file: A double-blind randomised controlled phase IIb study to evaluate the safety, immunogenicity and efficacy of GlaxoSmithKline Biologicals' candidate malaria vaccine RTS,S/AS02A, administered IM according to a 0, 1 and 2 month vaccination schedule in toddlers and children aged 1 to 4 years in a malaria-endemic region of Mozambique. Report 257049/026 (Malaria-026) 19 November 2004. Data on File at GlaxoSmithKline Biologicals.

GSK data on file: Double-blind, randomized phase I/IIa human challenge study, to evaluate the safety, reactogenicity, immunogenicity, and preliminary efficacy after

primary sporozoite challenge and rechallenge, of GSK Biologicals' candidate malaria vaccines containing the antigen RTS,S adjuvanted with either AS02A or AS01B and administered intramuscularly at months 0, 1, 2 in healthy malaria-naive volunteers aged 18 - 45 years. Protocol 257049/027 (Malaria-027) 24 July 2003. Data on File at GlaxoSmithKline Biologicals.

GSK data on file: Double-blind, randomized phase I/IIa human challenge study, to evaluate the safety, reactogenicity, immunogenicity, and preliminary efficacy after primary sporozoite challenge and rechallenge, of GSK Biologicals' candidate malaria vaccines containing the antigen RTS,S adjuvanted with either AS02A or AS01B and administered intramuscularly at months 0, 1, 2 in healthy malaria-naive volunteers aged 18 - 45 years. Report 257049/027 (Malaria-027) 01 February 2006. Data on File at GlaxoSmithKline Biologicals.

GSK data on file: A Phase I/II randomized double-blind bridging study to evaluate the safety and immunogenicity of GlaxoSmithKline Biologicals' candidate vaccines RTS,S/AS02D (0.5 mL dose) and RTS,S/AS02A (0.25 mL dose) administered IM according to a 0, 1, 2 month vaccination schedule in children aged 3 to 5 years living in a malaria-endemic region of Mozambique. Report 257049/034 (Malaria-034) 06 December 2004. Data on File at GlaxoSmithKline Biologicals.

GSK data on file: A Phase I/IIb randomized, double-blind, controlled study of the safety, immunogenicity and proof-of-concept of RTS,S/AS02D, a candidate malaria vaccine in infants living in a malaria-endemic region. Protocol 103967 (Malaria-038) 05 July 2005. Data on File at GlaxoSmithKline Biologicals.

GSK data on file: A Phase IIb randomized, double-blind, controlled study of the safety, immunogenicity and proof-of-concept of RTS,S/AS02D, a candidate malaria vaccine, when incorporated into an Expanded Program on Immunization (EPI) regimen that includes DTPw/Hib in infants living in a malaria-endemic region. Protocol 104298 (Malaria-040) 01 December 2005. Data on file at GlaxoSmithKline Biologicals.

GSK data on file: A Phase IIb randomized, double-blind, controlled study of the safety, immunogenicity and proof-of-concept of RTS,S/AS02A, and RTS,S/AS01B, two candidate malaria vaccines in malaria-experienced adults living in Western Kenya. Protocol 104743 (Malaria-044) 26 July 2005. Data on File at GlaxoSmithKline Biologicals.

GSK data on file: A Phase II randomized, double-blind bridging study of the safety and immunogenicity of GlaxoSmithKline Biologicals' candidate *Plasmodium falciparum* malaria vaccine RTS,S/AS01E (0.5 mL dose) to RTS,S/AS02D (0.5 mL dose) administered IM according to a 0, 1, 2-month vaccination schedule in children aged 18 months to 4 years living in Gabon. Protocol 105874 (Malaria-046) 16 February 2006. Data on File at GlaxoSmithKline Biologicals.

GSK data on file: A Phase II randomized, controlled, partially-blind study of the safety and immunogenicity of GlaxoSmithKline Biologicals' candidate *Plasmodium falciparum* vaccines RTS,S/AS02D and RTS,S/AS01E, when administered IM according to one of

three dose schedules in children aged 5 to 17 months living in Ghana. Protocol 106357 (Malaria-047) 13 February 2006. Data on File at GlaxoSmithKline Biologicals.

GSK data on file: A Phase IIb randomized, double-blind, controlled study of the efficacy against episodes of clinical malaria due to *Plasmodium falciparum* infection of GlaxoSmithKline Biologicals' candidate vaccine RTS,S/AS01E, administered IM according to a 0, 1, 2-month vaccination schedule in children aged 5 months to 17 months living in Tanzania and Kenya. Protocol 106464 (Malaria-049) 20 December 2006. Data on File at GlaxoSmithKline Biologicals.

GSK data on file: A Phase II randomized, open, controlled study of the safety and immunogenicity of GlaxoSmithKline Biologicals' candidate *Plasmodium falciparum* malaria vaccine RTS,S/AS01E, when incorporated into an Expanded Program on Immunization (EPI) regimen that includes DTPwHepB/Hib, OPV, measles and yellow fever vaccination in infants living in malaria-endemic regions. Protocol 106369 (Malaria-050) 03 January 2007. Data on File at GlaxoSmithKline Biologicals.

Kester KE, McKinney DA, Tornieporth N, Ockenhouse CF, Heppner DG, et al. Efficacy of recombinant circumsporozoite protein vaccine regimens against experimental *Plasmodium falciparum* malaria. *Journal of Infectious Diseases*. 2001; 15:183(4):640-7.

Knobel DL, Cleaveland S, Coleman PG et al. Re-evaluating the burden of rabies in Africa and Asia. *Bulletin of the World Health Organization* 2005: 83 (5): 360-368.

Kuwert EK, Marcus I, Werner J, Iwand A, Thraenhart O. Some experiences with human diploid cell strain-(HDCS) rabies vaccine in pre- and post-exposure vaccinated humans. *Dev Biol Stand* 1978; 40: 79-88.

Kwara A, Adegbola RA, Corrah PT, Weber M et al. Meningitis caused by a serogroup W135 clone of the ET-37 complex of *Neisseriea meningitidis* in West Africa. *Tropical Medicine and International Health.* 1998; 3 (9): 742-746.

Lalvani A, Moris P, Voss G, Kester KE, Brookes R, Koutsoukos M. Potent induction of focused Th-1 type cellular and humoral immune responses by RTS,S/SBAS2 recombinant *P. falciparum* malaria vaccine. *Journal of Infectious Diseases*. 1999; 180:1656-64.

McGuinness D, Koram K, Bennett S, Wagner G, Nkrumah F et al. Clinical case definitions for malaria: clinical malaria associated with very low parasite densities in African infants. *Transactions of the Royal Society of Tropical Medicine and Hygiene*: 1998; 92 (5), 527-531.

Miller E, Salisbury D, Ramsay M. Planning, registration and implementation of an immunization campaign against meningococcal serogroup C disease in the UK: a success story. *Vaccine.* 2002; S58-S67.

Moorthy V, Reed Z. and Smith PG. Measurement of malaria vaccine efficacy in phase III trials: Report of a WHO consultation. *Vaccine*. 2007: 25 (28) 5115-5123.

Mulholland K. Commentary: Comorbididty as a factor in child health and child survival in developing countries. *International Journal of Epidemiology*. 2005; 34: 375-377.

Mwangi TW. Case definitions of clinical malaria under different transmission conditions in Kilifi District Kenya. *Journal of Infectious Diseases* 2005; 191 (11) 1932-1939.

O'Dempsey TJD, McArdle TF, Laurence BE, Lamont AC, Todd JE, Greenwood BM. Overlap in the clinical features of pneumonia and malaria in African children. *Transactions of the Royal Society of Tropical Medicine and Hygiene*. 1993; 87 (6):662-665

Pinder M, Reece WH, Plebanski M et al. Cellular immunity induced by the recombinant *Plasmodium falciparum* malaria vaccine, RTS,S/AS02, in semi-immune adults in The Gambia. *Clin Exptl Immunol* 2004; 135 (2): 286-293.

Plotkin SA, Ruprecht CE, Koprowski H. *Rabies Vaccine*. *in:* Plotkin SA, Orenstein WA. *Vaccines* 4 ed. WB Saunders; Philadelphia, Pensylvania: 2004.

Reece WH, Pinder M, Gothard PK et al. A CD4+ T-cell immune response to a conserved epitope in the circumsporozoite protein correlates with protection from natural *Plasmodium falciparum* infection and disease. *Nature Medicine*. 2004; 10 (4): 406-410.

SAVVY Sample Vital Registration with Verbal Autopsy Verbal Autopsy Coder's Manual 2006.

Saúte F, Aponte J, Almeda J, Ascaso C, Abellana R, et al. Malaria in southern Mozambique: Malariometric indicators and malaria case definition in Manhiça district. *Transactions of the Royal Society of Tropical Medicine and Hygiene*. 2003: 97 (6); 661-666

Sediame S, Zerah-Lancner F, d'ortho MP, Adnot S, Harf A. Accuracy of the i-STAT™ bedside blood gas analyzer. *European Respiratory Journal*. 1999: 14 (1); 214.

Smith T, Armstrong Schellenberg J. Attributable fraction estimates and case definitions for malaria in endemic areas. *Statistics in Medicine*. 1994; 13: 2345-2358.

Steiner MJ, De Walt DA, Byerley JS. Is this Child Dehydrated? *JAMA*. 2004; 291: 2746 – 2754.

Stoute JA, Slaoui M, Heppner DG, Momin P, Kester KE, Desmons P et al. A Preliminary Evaluation of a Recombinant Circumsporozoite Protein Vaccine against *Plasmodium falciparum* Malaria. *N Eng J Med* 1997; 336: 86-91

Sun P, Schwenk R, White K, Stoute JA, Cohen J, Ballou WR et al. Protective immunity induced with malaria vaccine, RTS,S, is linked to *Plasmodium falciparum* circumsporozoite protein-specific CD4(+) and CD8(+) T cells producing IFN-gamma. *Journal of Immunology*. 2003; 171 (12): 6961-7.

Vryheid RE, Kane MA, Muller N, Schatz GC, Bezabeh S. Infant and Adolescent Hepatitis B Immunization up to 1999: A Global Overview. *Vaccine* 2001;19: 1026-1037.

Whittle HC, Evans-Jones G, Onyewotu I, Adjukiewicz et al. Group-C meningococcal meningitis in the northern savanna of Africa. *The Lancet* 1975; 305 1377.

WHO. WHO collaborative study on oral and inactivated poliovirus vaccines. Factors affecting the immunogenicity of oral poliovirus vaccine: a prospective evaluation in Brazil and The Gambia. Journal of infectious Diseases. 1995; 171: 1097-1106.

WHO. WHO collaborative study group on oral and inactivated poliovirus vaccines. COmbined immunization of infants with oral and inactivated poliovirus vaccines: results of a randomized trial in The Gambia, Oman and Thailand. Journal of Infectious Diseases. 1997; 175: S215-S227.

WHO. Pocket book of hospital care for children: guidelines for the management of common illnesses with limited resources. World Health Organization 2005. ISBN 92 4 1546700. (also refer to http://www.who.int/child-adolescent-health/New_Publications/CHILD_HEALTH/PB/00.PB_full_low.pdf)

WHO. Verbal autopsy standards : ascertaining and attributing cause of death. World Health Organization, Geneva, 2007.

WHO. Hepatitis B Fact Sheet WHO/204. 2003. http://www.who.int/mediacentre/factsheets/fs204/en/index.html Accessed 07 March 2008.

WHO. Model Chapter for Textbooks IMCI Integrated Management of Childhood Illness. WHO 2001. http://whqlibdoc.who.int/hq/2001/WHO_FCH_CAH_01.01.pdf. Accessed 07 March 2008.

Appendix A Administrative Matters

I. Responsibilities of the Investigator

- To ensure that he/she has sufficient time to conduct and complete the study and has adequate staff and appropriate facilities and equipment which are available for the duration of the study and to ensure that other studies do not divert essential subjects or facilities away from the study at hand.
- To submit an up-to-date curriculum vitae or Investigator Biography and other credentials (e.g. medical license number in the United States) to GSK and—where required—to relevant authorities. It is recommended that this documentation indicates any previous clinical research experience and history of training in GCP.
- To acquire the reference ranges for laboratory tests performed locally and, if required by local regulations, obtain the laboratory’s current certification or Quality Assurance procedure manual.
- To ensure that no clinical samples (including serum samples) are retained onsite or elsewhere without the approval of GSK Biologicals and the express written informed consent of the subject and/or the subject’s legally authorized representative.
- To perform no other biological assays on the clinical samples except those described in the protocol, its amendment(s) or ancillary protocols connected with this study.
- To prepare and maintain adequate subject source data or raw data designed to record observations, and other data pertinent to the study.
- To conduct the study in compliance with the protocol any amendment and “Good Clinical Practice” (GCP) and all applicable regulatory requirements.
- To co-operate with a representative of GSK Biologicals in the monitoring process of the study and in resolution of queries about the data.
- To permit drug regulatory agencies and GSK audits.

II. Protocol Amendments and Administrative changes

- No changes to the study protocol will be allowed unless discussed in detail with the GSK Biologicals' Clinical Development Manager/Medical Monitor and filed as an amendment/administrative change to this protocol.
- Any amendment/administrative change to the protocol will be adhered to by the participating centre(s) and will apply to all subjects. Written IRB/IEC approval of protocol amendments is required prior to implementation, except where permitted by all applicable regulatory requirements; administrative changes and amendments not submitted for approval are submitted to IRBs/IECs for information only.
- Submission of protocol amendments to regulatory agencies will occur in accordance with local regulatory requirements. For some countries, submission to the local regulatory authority may not be required. When submission to the local regulatory authority is required, the timing of the submission relative to IEC/IRB submission or approval and whether or not the authority will provide their approval of or favorable

opinion on the amendment before it can be implemented will depend on local regulatory requirements.

III. Sponsor’s Termination of Study

GSK Biologicals reserves the right to temporarily suspend or prematurely discontinue this study either at a single site or at all sites at any time for reasons including, but not limited to, safety or ethical issues or severe non-compliance. Reasons for suspension or early termination will be documented in the study file at GSK Biologicals.

If GSK Biologicals determines that suspension or early termination is needed, GSK Biologicals will discuss this with the Investigator (including the reasons for taking such action). When feasible, GSK Biologicals will provide advance notification to the investigator of the impending action prior to it taking effect.

GSK Biologicals will promptly inform, via written communication, all investigators and/or institutions conducting the study, if the study is suspended or terminated for safety reasons, and will also inform the regulatory authorities of the suspension or termination of the study and the reason(s) for the action. If required by applicable regulations, the investigator must inform the IEC/IRB promptly and provide the reason for the suspension or termination.

If the study is prematurely discontinued, all study data must be returned to GSK. In addition, arrangements will be made for all unused investigational product(s) in accordance with the applicable GSK procedures for the study. Financial compensation to investigators and/or institutions will be in accordance with the agreement established between the investigator and/or institutions and GSK.

IV. Case Report Form Instructions / Remote Data Entry Instructions

In this study, where technically possible, study centers will use remote data entry (RDE) with electronic case report form (eCRF) in preference to conventional paper case report form (CRF)

Remote Data Entry

Where technically possible, remote data entry (RDE) will be used as the method for data collection, which means that subject information will be entered into a computer at the investigational site preferably within 5 working days of becoming available. The site will be capable of modifying the data to assure accuracy with source documentation. All new/updated information will be reviewed and verified by a GSK Biologicals’ representative. This information will finally be stored in a central database maintained by GSK Biologicals. At the conclusion of the study, GSK Biologicals will archive the study data in accordance with internal procedures. In addition, the investigator will be provided with a CD-ROM of the final version of the data generated at the investigational site.

Specific instructions for use of RDE will be included in the training material provided to the investigational site.

Case Report Forms

Prior to screening the first potential participant, the investigator will provide the Site Monitor with a list (Site Staff Signature Sheet) showing the name and title, signature and initials of all site staff who have a critical effect on the conduct of the study and to whom the investigator has delegated significant study related duties such as entering data on the CRFs or changing entries on CRFs. If the authorized individuals should change during the study, the investigator is to inform GSK Biologicals GSK Biologicals' representative of the specific change(s).

CRFs (and subject diary cards, if applicable), will be supplied by GSK Biologicals for recording all data. It is the responsibility of the investigator or co-investigator to ensure that study data are legible, accurate, adequately recorded and, when entered on paper copy, completely filled in with a black ink fountain or ballpoint pen.

Errors must be corrected by drawing a single line through the incorrect entry and writing in the new value/data positioned as close to the original as possible. The correction must then be initialed and dated (and justified, whenever possible), where necessary, by the authorized individual making the change. The original entry must not be obliterated, overwritten or erased when a correction is made.

When a subject completes a visit, it is anticipated that relevant sections of the CRF will be completed by the investigator (or designated staff as documented in the Site Staff Signature Sheet) as soon as possible after the last data becoming available (preferably within 5 working days of becoming available). Similarly, when a subject completes a study, it is anticipated that all relevant CRF pages will be completed promptly after the last data becoming available. This also applies to forms for potential study participants who were screened but not randomized to a study group.

As soon as the subject has completed/withdrawn from the study and the CRF is completed, the investigator or medically qualified sub-investigator to whom this task has been delegated will sign the study conclusion pages of the CRF to confirm that they have reviewed the data and that the data are complete and accurate. In all cases the investigator remains accountable for the study data collected.

An original (top copy) CRF or log sheets must be submitted for all subjects who have undergone protocol specific procedures, whether or not the subject completed the study.

While completed CRFs are reviewed by a GSK Biologicals' professional monitor at the study site, errors detected by subsequent in-house CRF review may necessitate clarification or correction of errors with documentation and approval by the investigator or appropriately qualified designee as documented on the Site Staff Signature Sheet. In all cases, the investigator remains accountable for the study data. Wherever possible the investigator should assist in the clarification or correction of errors detected after study finalization promptly after being brought to the attention of the investigator (preferably within 5 working days).

Any questions or comments related to the CRF should be directed to the assigned Site Monitor.

V. Monitoring by GSK Biologicals

To ensure compliance with the protocol, monitoring visits by a professional representative of the sponsor will be scheduled to take place early in the study, during the study at appropriate intervals and after the last subject has completed the study. It is anticipated that monitoring visits will occur at a frequency defined and communicated to the investigator before study start.

These visits are for the purpose of confirming that GSK Biologicals' sponsored studies are being conducted in accordance with the ethical principles that have their origins in the Declaration of Helsinki and that are consistent with Good Clinical practice (GCP) and the applicable regulatory requirement(s) (verifying continuing compliance with the protocol, amendment(s), reviewing the investigational product accountability records, verifying that the site staff and facilities continue to be adequate to conduct the study. Direct access to all study-related site and source data/ documents is mandatory for the purpose of monitoring review. The monitor will perform a CRF review and a Source Document verification (verifying CRF/ RDE entries by comparing them with the source data/documents that will be made available by the investigator for this purpose: any data item for which the CRF will serve as the source must be identified, agreed and documented. Data to be recorded directly into the CRF pages/RDE screens will be specified in writing preferably in the source documentation agreement form that is contained in both the monitor's and investigator's study file. For RDE, the monitor will mark completed and approved screens at each visit. The investigator must ensure provision of reasonable time, space and adequate qualified personnel for monitoring visits. Source data verification (SDV) must be conducted using a GSK standard SDV sampling strategy (as defined at the study start in the study specific monitoring guidelines) in which monitors will perform partial SDV for all subjects and full SDV for selected subjects.

VI. Archiving of Data

Following closure of the study, the investigator must maintain all site study records in a safe and secure location. The records must be maintained to allow easy and timely retrieval, when needed (e.g. audit or inspection), and, whenever feasible, to allow any subsequent review of data in conjunction with assessment of the facility, supporting systems, and staff. Where permitted by applicable laws/regulations or institutional policy, some or all of these records can be maintained in a validated format other than hard copy (e.g. microfiche, scanned); however, caution needs to be exercised before such action is taken. The investigator must assure that all reproductions are legible and are a true and accurate copy of the original, and meet accessibility and retrieval standards, including re-generating a hard copy, if required. Furthermore, the investigator must ensure there is an acceptable back-up of these reproductions and that an acceptable quality control process exists for making these reproductions.

GSK will inform the investigator/ institution of the time period for retaining these records to comply with all applicable regulatory requirements. However, the investigator/ institution should seek the written approval of the sponsor before proceeding with the disposal of these records. The minimum retention time will meet the strictest standard

applicable to that site for the study, as dictated by ICH GCP E6 Section 4.9, any institutional requirements or applicable laws or regulations, or GSK standards/procedures; otherwise, the minimum retention period will default to 15 years.

The investigator/ institution must notify GSK of any changes in the archival arrangements, including, but not limited to, the following: archival at an off-site facility, transfer of ownership of the records in the event the investigator leaves the site.

VII. Audits

For the purpose of compliance with Good Clinical Practice and Regulatory Agency Guidelines it may be necessary for GSK or a Drug Regulatory Agency to conduct a site audit. This may occur at any time from start to after conclusion of the study.

When an investigator signs the protocol, he agrees to permit drug regulatory agencies and GSK audits, providing direct access to source data/ documents. Furthermore, if an investigator refuses an inspection, his data will not be accepted in support of a New Drug Registration and/or Application, Biologics Licensing Application.

Having the highest quality data and studies are essential aspects of vaccine development. GSK has a Regulatory Compliance staff who audit investigational sites. Regulatory Compliance assesses the quality of data with regard to accuracy, adequacy and consistency. In addition, Regulatory Compliance assures that GSK Biologicals' sponsored studies are in accordance with GCP and that relevant regulations/guidelines are being followed.

To accomplish these functions, Regulatory Compliance selects investigational sites to audit. These audits usually take 1 to 2 days. GSK's audits entail review of source documents supporting the adequacy and accuracy of CRFs, review of documentation required to be maintained, and checks on vaccine accountability. GSK's audit therefore helps prepare an investigator for a possible regulatory agency inspection as well as assuring GSK Biologicals of the validity of the database across investigational sites.

The Inspector will be especially interested in the following items:

- Log of visits from the sponsor's representatives
- Study personnel
- Study file
- Safety reporting
- IRB/IEC and regulatory authority approvals
- Facilities
- monitoring
- Vaccine accountability
- Approved study protocol and amendments and investigator brochure

- Informed consent of the subjects (written consent [or witnessed oral if applicable])
- Medical records and other source documents supportive of CRF data
- Reports to the IRB/IEC and the sponsor
- Record retention.

GSK Biologicals will gladly help investigators prepare for an inspection.

VIII. Ownership, Confidentiality and Publication

Ownership:

All information provided by GSK and all data and information generated by the site as part of the study (other than a subject's medical records) are the sole property of GSK.

All rights, title, and interests in any inventions, know-how or other intellectual or industrial property rights which are conceived or reduced to practice by site staff during the course of or as a result of the study are the sole property of GSK, and are hereby assigned to GSK.

If a written contract for the conduct of the study which includes ownership provisions inconsistent with this statement is executed between GSK and the study site, that contract's ownership provisions shall apply rather than this statement.

Tripartite agreements are in place for the conduct of the study the signatories to which are the investigator groups, MVI and GSK. The operation of the investigator network is guided by Clinical Trials Partnership Committee (CTPC) charter.

Confidentiality:

Documented evidence that a potential investigator is aware and agrees to the confidential nature of the information related to the study must be obtained by means of a confidentiality agreement.

All information provided by GSK and all data and information generated by the site as part of the study (other than a subject's medical records) will be kept confidential by the investigator and other site staff. This information and data will not be used by the investigator or other site personnel for any purpose other than conducting the study. These restrictions do not apply to: (1) information which becomes publicly available through no fault of the investigator or site staff; (2) information which it is necessary to disclose in confidence to an IEC or IRB solely for the evaluation of the study; (3) information which it is necessary to disclose in order to provide appropriate medical care to a study subject; or (4) study results which may be published as described in the next paragraph. If a written contract for the conduct of the study which includes confidentiality provisions inconsistent with this statement is executed, that contract's confidentiality provisions shall apply rather than this statement.

Publication:

For multicenter studies, the first publication or disclosure of study results shall be a complete, joint multicenter publication or disclosure coordinated by GSK. Thereafter, any secondary publications will reference the original publication(s).

All publications will be based on a pre-agreed Statistical analysis plan (SAP). In the event that GSK and MVI make or coordinate a publication of results from a study in the CDP, then authorship shall be determined in accordance with GSK and MVI's respective policies and generally accepted standards for authorship. Principles of authorship for publications on multi-site studies shall be determined by the CTPC prior to the commencement of each trial.

Prior to submitting for publication, presentation, use for instructional purposes, or otherwise disclosing the study results generated by the site (collectively, a "Publication"), the investigator shall provide GSK with a copy of the proposed Publication and allow GSK a period to review the proposed Publication (at least 21 [twenty-one] days, or at least 15 [fifteen] working days for abstracts/posters/presentations). Proposed Publications shall not include either GSK, MVI or another investigator group's confidential information other than the study results or personal data on any subject, such as name or initials and according to the terms of the clinical trial agreement.

At GSK's request, the submission or other disclosure of a proposed Publication will be delayed a sufficient time to allow GSK to seek patent or similar protection of any inventions, know-how or other intellectual or industrial property rights disclosed in the proposed Publication.

If a written contract for the conduct of the study, which includes publication provisions inconsistent with this statement is executed, that contract's publication provisions shall apply rather than this statement.

Amended 23 January 2012

Appendix B Handling of Biological Samples Collected by the Investigator

Instructions for Handling of Serum Samples

When materials are provided by GSK Biologicals, it is MANDATORY that all clinical samples (including serum samples) will be collected and stored using exclusively those materials in the appropriate manner. The use of other materials could result in the exclusion of the subject from the ATP analysis. The investigator must ensure that his/her personnel and the laboratory(ies) under his/her supervision comply with this requirement. However, when GSK Biologicals does not provide material for collecting and storing clinical samples, then appropriate materials from the investigator's site are to be used.

1. Collection

The whole blood (by capillary or venous route) should be collected observing appropriate aseptic conditions. It is recommended that Vacutainer® tubes WITH integrated serum separator (e.g. Becton-Dickinson Vacutainer® SST or Corvac® Sherwood Medical) be used to minimize the risk of hemolysis and to avoid blood cell contamination of the serum when transferring to standard serum tubes.

2. Serum separation

These guidelines aim to ensure high quality serum by minimizing the risk of hemolysis, blood cell contamination of the serum or serum adverse cell toxicity at testing.

- For separation of serum using Vacutainer® tubes, the instructions provided by the manufacturer should be followed. Often the manufacturer's instruction states that the relative centrifugal acceleration known also as "G" must be "between 1000 and 1300 G" with tubes spinning for ten minutes. Error in calculation of centrifuge speed can occur when laboratory personnel confuse "G" acceleration with "RPM" (revolutions per minute). The speed of centrifugation must be calculated using the "G" rate provided in the manufacturer's instructions and the radius of the centrifuge head. After measuring the radius of the centrifuge machine, a speed/acceleration nomograph must be employed to determine the centrifuge speed in "RPM".
- Following separation, the serum should be aseptically transferred to the appropriate standard tubes using a sterile disposable pipette. The serum should be transferred as gently as possible to avoid blood cell contamination.
- The tube should not be overfilled (max. 3/4 of the total volume) to allow room for expansion upon freezing.
- The tube should be identified by the appropriate label provided by GSK Biologicals (see point 3).

3. Labeling

- The standard labels provided by GSK Biologicals should be used to label each serum sample.

- If necessary, any hand-written additions to the labels should be made using indelible ink.
- The label should be attached to the tube as follows (see diagram):
  - first attach the paper part of the label to the tube
  - then wrap the label around the tube so that the transparent, plastic part of the label overlaps with the label text and bar code and shields them.

This will ensure optimal label attachment.

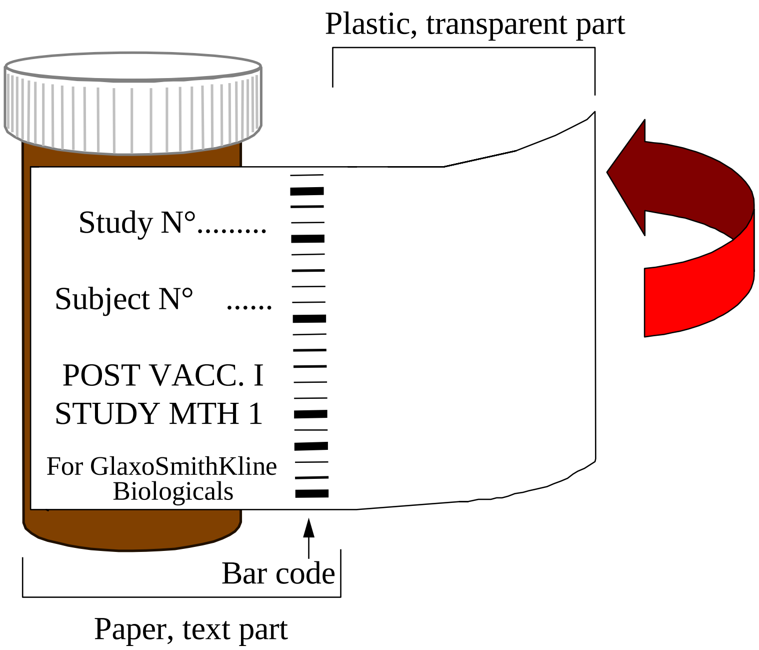

- Labels should not be attached to caps.

4. Sorting and storage

- Tubes should be placed in the GSK Biologicals' cardboard boxes in numerical order from left to right, starting from the lower left hand corner, beginning with the pre-vaccination samples series, then with the post-vaccination sample series.
- The tubes of serum should be stored in a vertical position at approximately -20°C (alternatively at approximately -70°/80°C is also acceptable) until shipment to GSK Biologicals. The storage temperature should be checked regularly and documented. Wherever possible, a backup facility for storage of serum samples should be available.
- A standard Biological Specimen Listing Form, specifying the samples being shipped for individual subjects at each timepoint, should be prepared for each shipment. A copy of this list should be retained at the study site, while the original should be sealed in a plastic envelope and shipped with the serum samples.
- Once shipment details are known, a standard Specimen Transfer Form must be completed and faxed to GSK Biologicals to the number provided below. A copy of the Specimen Transfer Form must be in the parcel.[1]

GLAXOSMITHKLINE BIOLOGICALS

PII - blinded

[1] The Biological Specimen Listing Form and the Specimen Transfer Form are standard documents used in GSK Biologicals' clinical trials. These documents are provided by GSK Biologicals' Clinical Trials' monitor at study initiation.

Appendix C Shipment of Biological Samples

Instructions for Shipment of Serum Samples

Serum samples should be sent to GSK Biologicals at regular intervals. The frequency of shipment of samples should be decided upon by the Site Monitor, Central Study Coordinator and the investigator prior to the study start.

Serum samples must be placed with dry ice (maximum -20°C) in a container complying with International Air Transport Association (IATA) requirements if shipment by air or complying with ADR or local regulations if transport by road. The completed standard Biological Specimen Listing Form should always accompany the shipment.

The container must be clearly identified with the labels provided by GSK Biologicals specifying the shipment address and the storage temperature (-20°C).

The airway bill should contain the instruction for storage of samples at maximum -20°C.

A "proforma" invoice, stating a value for customs purposes only, should be prepared and attached to the container. This document should contain the instruction for storage of samples at maximum -20°C.

Details of the shipment, including:

* number of samples
* airway bill
* flight number
* flight departure and arrival times

should be sent by fax or by e-mail, two days before shipment, to:

PII - blinded

Appendix D Vaccine supplies, packaging and accountability

1. Vaccine and/or other supplies

GSK Biologicals will supply the following study vaccines, sufficient number of doses to administer to all subjects as described in the present protocol.

Sufficient doses for:

- up to 11 000 recipients of RTS,S/AS01E (5500 to receive 3 doses plus boost, 5500 to receive 3 doses without boost)
- up to 3500 recipients of Rabies vaccine (all to receive 3 doses)
- up to 11 000 recipients of meningococcal C vaccine (3500 to receive a primary course, 11 000 to receive boost)
- up to 10 000 recipients of Tritanrix HepB/Hib (all to receive 3 doses)
- up to 10 000 recipients of Polio Sabin (all to receive 4 doses)

Amended 24 October 2008

At least an additional 3% of each vaccine will be supplied for replacement in case of breakage, bad storage conditions or any other reason that would make the vaccine unusable (i.e. given by mistake to another subject).

All containers must be accounted for on the form provided.

2. Labels for sample identification:

The investigator will receive labels from GSK Biologicals to identify samples taken from each subject at each timepoint. Each label will contain the following information: study number, subject number, sampling timepoint (e.g., post vaccination 3), timing (e.g., study Month 7).

Amended 26 November 2009

In addition to the vaccines, the study documentation and the sample labels, the investigator will receive the following supplies:

- tubes with screw caps for serum samples,
- racks for the tubes of serum.

The investigator or pharmacist must sign a statement that he/she has received the clinical supplies for the study.

It is NOT permitted to use any of the supplies provided by GSK Biologicals for purposes other than those specified in the protocol.

3. Vaccine packaging

The vaccines will be packed in labelled boxes. The box label will contain, as a minimum, the following information: study number, treatment number, lot number (or numbers,

when double-blind), instructions for vaccine administration and any other relevant regulatory requirements.

Vaccine shipment from GSK Biologicals Rixensart to local country medical department, dispatching centre or investigational site from local country medical department to investigational site

Upon reception of the shipment, its content, quality and maintenance of the cold-chain must be checked.

The supplies receipt documents must then be returned to the sender, as appropriate:

PII - blinded *PII - blinded*

Amended 26 November 2009

In case of any temperature deviation, the official written approval for the use of vaccine must be obtained from GSK.

4. Vaccine accountability

At all times the figures on supplied, used and remaining vaccine doses should match. At the end of the study, it must be possible to reconcile delivery records with those of used and unused stocks. An explanation must be given of any discrepancies.

After approval from GSK Biologicals and in accordance with GSK SOP WWD-1102, used and unused vaccine containers should be destroyed at the study site using locally approved biosafety procedures and documentation unless otherwise described in the protocol. If no adequate biosafety procedures are available at the study site, the used and unused vaccine containers are to be returned to an appropriate GSK Biologicals site for destruction, also in accordance with current GSK SOP WWD-1102.

5. Transfers of clinical vaccines or products from country medical department or dispatch centre to study sites or between sites

Storage temperatures must be maintained during transport and deviations must be reported to GSK for guidance. All transfers of clinical vaccines or products must be documented.

All packaging and shipment procedures for transfer of clinical vaccines or products must follow procedures approved by the sponsor.

Clinical vaccines or products should always be sent by contract courier designated by the sponsor, unless otherwise requested by the sponsor.

Appendix E Assessment of a potential case of severe malaria disease

Algorithm for the evaluation of a hospital admission as a potential case of severe malaria

For all acute hospital admissions (i.e. except planned admissions for medical investigation/care or elective surgery and trauma admissions), a CRF/eCRF module will be filled in and a blood sample taken for evaluation of

- malaria parasite density
- blood culture
- hemoglobin
- blood glucose, lactate and base excess determination

Amended 24 October 2008
Amended 26 November 2009

Lumbar Puncture (LP) is indicated by:

- Seizure except simple febrile seizure (simple febrile seizure is defined as associated with fever, lasts for 5 minutes or less, generalized as opposed to focal, not followed by transient or persistent neurological abnormalities, occurring in a child ≥ 6 months of age, with full recovery within 1 hour)
- Level of consciousness < 5 (children ≤ 9 months of age < 4 [in association with best motor response of 1])
- Prostration in child < 3 year of age
- Meningism stiff neck/bulging fontanelle
- Clinician’s judgment: note that this list is not an exhaustive list of indications for lumbar puncture in clinical practice

NB can be delayed until child is stable

Chest X-ray (CXR) is indicated by:

- Tachypnea (≥50 breaths per minute < 1 year and ≥ 40 breaths per minute ≥ 1year) [Berkley, 2003]
- Lower chest wall indrawing
- Abnormally deep breathing
- Clinician’s judgment: note that this list is not an exhaustive list of indications for CXR in clinical practice

Amended 24 October 2008

Methodologies for the assessment of clinical signs

Level of consciousness (Blantyre Score)

Conscious level will be scored using the Blantyre coma scale (Appendix E Table 1) [Molyneux, 1989].

Appendix E Table 1 Coma scale for young children

	Score
**Best motor response**	
Localizes painful stimulus*	2
Withdraws limb from painful stimulus†	1
No response or inappropriate response	0
**Best verbal response**	
Cries appropriately with painful stimulus* or, if verbal, speaks	2
Moan or abnormal cry with painful stimulus	1
No vocal response to painful stimulus	0
**Eye movement**	
Watches or follows (e.g. mother's face)	1
Fails to watch or follow	0

To obtain 'coma score', add the scores for each section; maximum = 5, minimum = 0
* Pressure with blunt end of pencil on sternum or supraorbital ridge
† Pressure with horizontal pencil on nailbed of finger or toe

Guidance on the application of the scale is given by WHO [WHO, 2000a]. 'Testing should begin with a minimal stimulus which should be increased only to a point where a clear response is obtained. A localizing response must be distinguished from a brisk flexion response which brings the hand into coincidental proximity to the stimulus. The interpretation of 'verbal cry' is difficult, some children are stoical and the appropriateness of verbal response needs to be considered in the light of other responses and the age of the child. It is best to test orientation by asking the mother to move her face across the child's field of vision as the child may be less interested in looking at a strangers face.'

The coma score will be assessed at presentation. If it is depressed, it will be re-assessed after initial resuscitation; approximately one hour after correction of hypoglycemia and control of fits. This value will be recorded in the CRF/eCRF (if anticonvulsants were given prior to the assessment this will be documented in the CRF/eCRF). The intention is to avoid diagnosing cerebral malaria in a child with post-ictal coma [WHO, 2000a]. A child with persistent seizure activity despite optimal therapy, should have the coma scale recorded 1 hour after treatment is optimized.

A normal coma score in a child of > 9 months of age is 5. In children ≤ 9 months of age, because localization of pain is difficult to assess, a normal coma score is 4 (in association with a best motor response of 1).

Skin Turgor

Dehydration will be assessed by skin pinch test. The standard procedures are:

- Locate the area on the child's abdomen halfway between the umbilicus and the side of the abdomen. The hand should be placed so that when the skin is pinched, the fold of skin will be in a line up and down the child's body and not across the child's body
- Pinch the skin using the thumb and first finger. It is important to firmly pick up all of the layers of skin and the tissue under them for one second and then release it.
- When released, the skin pinch goes back either very slowly (longer than 2 seconds), or slowly (skin stays up even for a brief instant), or immediately. In a child with marasmus (severe malnutrition), the skin may go back slowly even if the child is not dehydrated. In an overweight child, or a child with edema, the skin may go back immediately even if the child is dehydrated. [WHO, 2000b; WHO, 2001; WHO, 2005]

Appendix E Figure 1 Diagnosis of dehydration: Left: pinching the child's abdomen to test for decreased skin turgor Right: Slow return of skin pinch in severe dehydration [WHO, 2005]

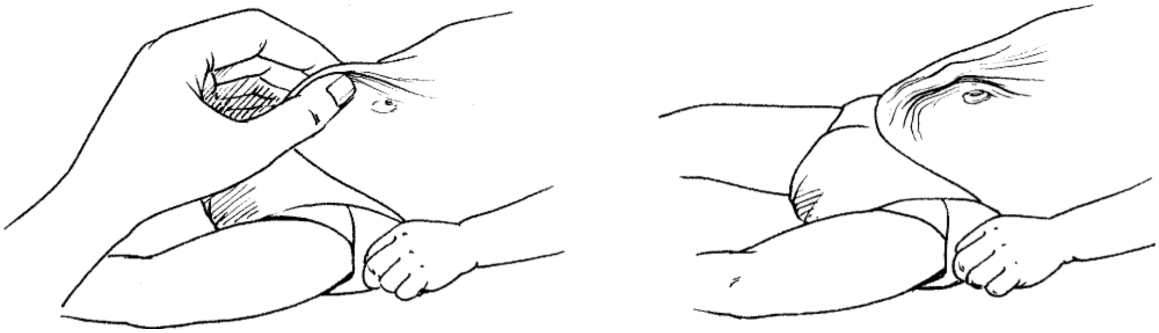

Prostration

Prostration will be defined as, in an acutely sick child, the inability to perform previously-acquired motor function: in a child previously able to stand, inability to stand; in a child previously able to sit, inability to sit and in a very young child, inability to suck.

Guidance on the assessment of prostration is given by the WHO [WHO, 2000a]. 'Prostration must always be recorded directly and not based on history. Many children who are pyrexial and feel unwell prefer to lie or be carried but are capable of sitting if gently encouraged to do so.'

Respiratory signs

Respiratory signs will be assessed in line with the guidance on the assessment of respiratory signs by WHO [WHO, 2005]. 'Respiratory signs should be looked for while the child is calm; (usually before the child is disturbed by hands on clinical examination). Ask the mother or caretaker to cautiously reveal part of the chest to look for lower chest wall indrawing or to count the respiratory rate. If a child is distressed or crying, it might

need to be left for a brief time with its mother in order to settle, or the mother could be ask to breastfeed, before key signs such as respiratory rate can be measured. Then proceed to signs which require touching the child but are little disturbing.'

Rate

The respiratory rate will be counted over one full minute while the child is calm [WHO, 2005].

Lower chest wall indrawing

Lower chest wall indrawing will be defined as the inward movement of the bony structure of the chest wall with inspiration [WHO, 2001].

Appendix E Figure 2 Lower chest wall indrawing: with inspiration, the lower chest wall moves in [WHO, 2005]

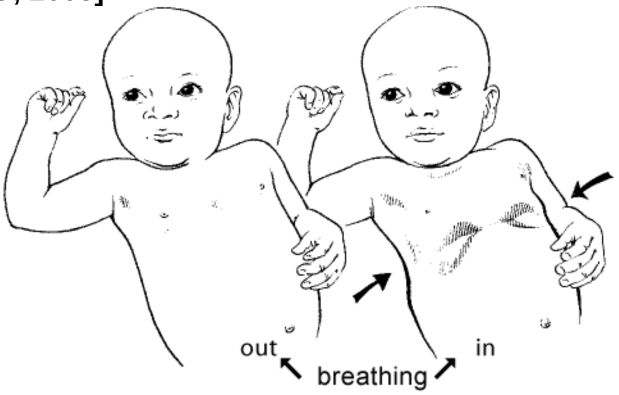

Note: chest indrawing should only be considered present if it is consistently present in a calm child. Agitation, a blocked nose or breastfeeding can all cause temporary chest indrawing [WHO, 2001].

Note: lower chest wall indrawing occurs when the lower chest wall goes in when the child breathes in; if only the soft tissue between the ribs or above the clavicle goes in when the child breathes, this is not lower chest wall indrawing [WHO, 2005].

Acidotic or Kussmaul respiration

Abnormally deep breathing will be defined as breathing that is deep and labored while the chest is clear [WHO, 2005]. The key component is increased inspiratory and expiratory excursion of the chest [WHO, 2000a].

References for Appendix E

Berkley JA, Ross A, Mwangi I et al. Prognostic indicators of early and late death in children admitted to district hospital in Kenya: cohort study. British Medical Journal 2003; 326: 361-366.

Molyneux ME, Taylor TE, Wirima JJ, Borgstein A. Clinical features and prognostic indicators in paediatric cerebral malaria: a study of 131 comatose Malawian children *Quarterly Journal of Medicine* 1989; 71 265 441-459

WHO Communicable Diseases Cluster. Severe *falciparum* malaria. *Trans R Soc Trop Med Hyg.* 2000 a; 94 Suppl 1: S1-90.

WHO Management of the Child with a Serious Infection or Severe Malnutrition: Guidelines for Care at the first-referral level in developing countries. 2000 b; http://www.who.int/child-adolescent-health/publications/referral_care/Referral_Care_en.pdf

WHO. Pocket book of hospital care for children; Guidelines for the management of common illnesses with limited resources. 2005; http://www.who.int/child-adolescent-health/New_Publications/CHILD_HEALTH/PB/00.PB_full_low.pdf (Accessed 11 April 2007).

WHO. Model Chapter for Textbooks: IMCI, Integrated Management of Childhood Illness. 2001. http://www.who.int/child-adolescent-health/New_Publications/IMCI/WHO_FCH_CAH_00.40/WHO_FCH_CAH_00.40.pdf (Accessed 11 April 2007).

Appendix F Determination of *Plasmodium falciparum* asexual parasite density

Slide reading methodology

Two blood slide reading methods will be used in the study. Comparability of results will be assured through an external Quality Assessment Program ongoing through the trial. All slide readers, by both methods will be required to demonstrate proficiency at "Competent" level or above. The external quality assurance program is based on current WHO recommendations [World Health Organization, 2006].

Method 1: Counting against known blood cell concentration

Refer to the method described in Greenwood and Armstrong 1991 [Greenwood, 1991].

A contemporaneous measured white and red blood cell count will be determined and used in parasite density calculation.

6 μL of blood for the thick smear and 2 μL of blood for the thin smear are put on the same slide with a standard template. All slides are prepared in duplicates.

Negative result: 100 fields on the thick blood smear free of parasites are to be read before a slide is declared negative

Positive result:

- ***High parasitemia:*** If 100 parasites or more are seen on the first field of the thick smear, the parasites are counted on the thin film. RBC and parasitized red blood cells are counted on the thin film until a minimum of 20 parasitized RBC are reached. If 20 parasitized RBCs are counted before all the parasitized RBCs in a field are counted, finish counting all parasitized RBCs and non-parasitized RBCs in that field.
- ***Medium parasitemia:*** If less than 100 parasites are seen on the first field of the thick smear, parasites are counted up to when 200 white blood cells (WBC) are counted, on the thick smear. If 200 WBC are counted before finishing a field, finish counting all parasites and WBC in that field.
- ***Low parasitemia:*** If less than 10 parasites are counted per 200 WBC on the thick smear, the parasite counting is extended up to when 500 WBC have been counted. If 500 WBC are counted before finishing a field, finish counting all parasites and WBC in that field.

Method 2. Counting against known blood volume

Refer to the method described in Planche et al, 2001 [Planche, 2001].

10 μL blood are spread with a pipette on an area of 1x1.8 cm, using a template on which the slide is set.

Negative result: 100 fields free of parasites are read before a slide is declared negative

Positive result: if there are 1-9 parasites per field, 100 fields are counted. If there are 10-99 parasites per field, 10 fields are counted. If there are 100-999 parasites per field, 1 field is counted.

The parasitemia count will be calculated on the basis of the following formula:

- Parasites/µL = parasites/field x Microscope Factor (MF, assumed blood volume/microscope high power field).

To calculate the MF:

- Make a thick blood film with 10 µL of blood
- Calculate the mean number of leucocytes per high power field, after counting leucocytes across 10 fields
- Count the number of leucocytes in 10 µL of blood using a computerized blood analyzer.

X = Mean leucocytes number per high power field (microscopy)

Y = Leucocytes in 10 µL of blood (automated analyzer)

MF = Y / X

For each microscope this predetermination of MF is done once.

Criteria for concordance for double reading of slides

All slides will be read twice, by two independent readers to quantify the *P. falciparum* parasite density. Slides are judged to be discordant and a third independent read will be organized in the following cases:

a. The result from one reader is negative and the one of the other is positive
b. For high and medium positive parasitemia results (blood parasitemia > 400/µL), the higher count divided by the lower count is > 2
c. For low parasitemia (blood parasitemia ≤ 400/µL), the highest reading density is more than one $\log_{10}$ higher than the lowest reading.

If parasitemia result is high or medium and one is low, i.e. one is > 400/µL and the other is ≤ 400/µL, parasitemia, criteria (C) will be applied.

Amended 24 October 2008

Determination of final result

If there are two concordant results the final result is the geometric mean of the two readings.

If the first two readings are discordant then the final result will follow the following principles:

- For cases of positive/negative discrepancy (A), the majority decision will be adopted. If the decision is positive, the final result will be the geometrical mean of the two positives.
- For cases of three positive reads (B and C), the final result will be the geometric mean of the two geometrically closest readings

Identification of *Plasmodium* species

Positive parasitemia identified on any thick blood film must always be identified to species. This will be done on thin blood film except in case of low parasitemia. Only *Plasmodium falciparum* results will be accounted for in the efficacy analyses.

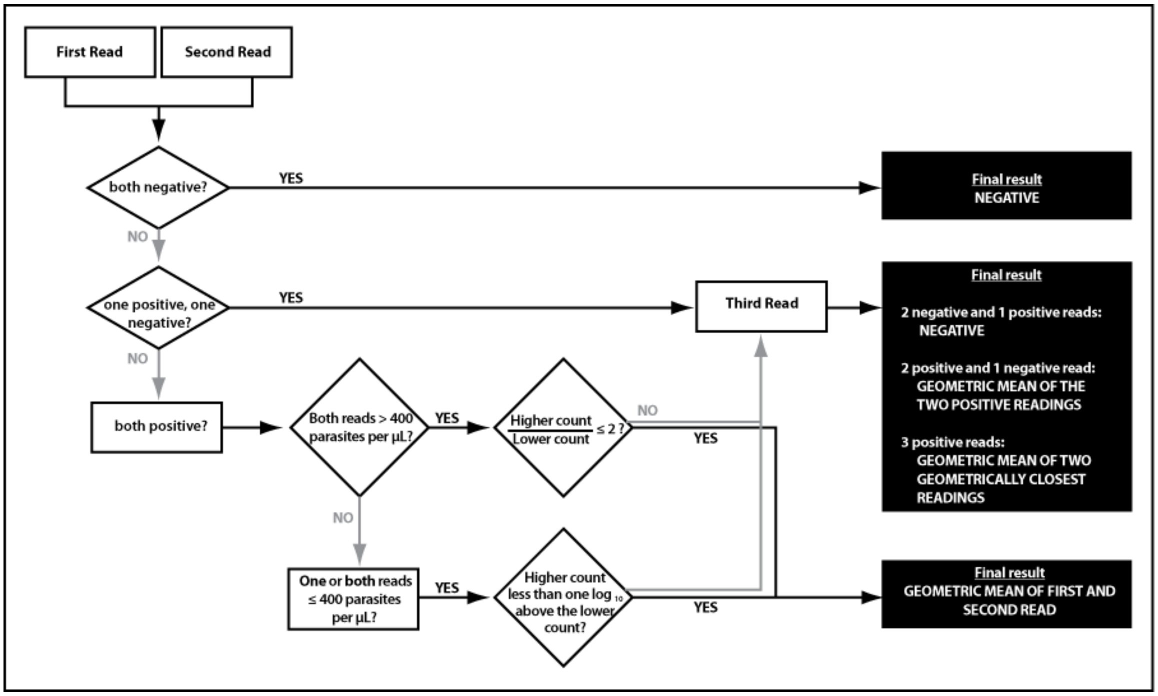

Amended 24 October 2008

Archiving of blood slides

Two blood slides are taken, one for reading and archiving at the sites; the second one will be archived centrally.

Amended 19 August 2008

References for Appendix F

Greenwood BM and Armstrong JRM. Comparison of two simple methods for determining malaria parasite density. *Transactions of the Royal Society of Tropical Medicine and Hygiene* 1991; 85: 186-88.

Planche T, Krishna S, Kombila M, Engel K, Faucher JF et al. Comparison of methods for the rapid laboratory assessment of children with malaria. *American Journal of Tropical Medicine and Hygiene* 2001; 65: 599-602.

World Health Organization. *Informal consultation on quality control of malaria microscopy*. 2006. WHO Geneva.

Appendix G Radiography

PERFORMANCE OF CHEST X-RAY

Chest anterior-posterior erect basic: technique

1. Position the patient. Support the child by holding him/her by the arms (If possible, his/her feet can rest on a stool or the floor or by another person holding his/her thighs) with its back resting against the front of the cassette holder.

Amended 19 August 2008

2. USE A PROTECTIVE LEAD STRIP OVER THE INFANT'S PELVIC AREA.
3. THE PERSON HOLDI NG THE CHILD, preferably one of the parents, MUST WEAR A LEAD APRON and whenever possible LEAD GLOVES.
4. Center between the nipples. COLLIMATE FURTHER, if possible.
5. Expose when the infant is not moving, preferably in INSPIRATION.

Chest anterior-posterior supine: technique

Required for infant weighing up to 10 kg and for older children as judged appropriate by the radiologist

Amended 24 October 2008

1. Lie the infant on its back on the cassette. THE INFANTS' HEAD AND LEGS MUST BE SUPPORTED.
2. USE A PROTECTIVE LEAD STRIP OVER THE INFANT'S PELVIC AREA.
3. THOSE SUPPORTING, preferably the infants' parents, MUST WEAR LEAD APRONS, and, whenever possible, LEAD GLOVES.
4. Center between the nipples. COLLIMATE FURTHER, if possible.
5. Expose when the infant is not moving, preferably in INSPIRATION.

Quality X-ray parameters

- ID on the right side
- Position: clavicles and ribs symmetric on each side of the spine
- Boundaries: Rib cage and costophrenics angles
- Inspiration: dome of the diaphragm is below the anterior tip of the 6th right rib

Amended 24 October 2008

- Movement: heart, diaphragm, central vessels and ribs sharply defined, without blurring
- Exposure: vascular shadows can be seen in lung periphery, thoracic vertebrae and large lower lobe vessels visible through cardiac silhouette
- Contrast: background outside patient's silhouette is black. Bones and airway easily distinguished from soft tissue.

Digital X-ray quality parameters

It is recommended to follow the guidelines listed in the manual 'Standardization of interpretation of chest radiographs for the diagnosis of pneumonia in children' (WHO Pneumonia Vaccine Trial Investigators' Group).

The objectives are:

- To establish standard definitions for the interpretation of chest radiographs in children with suspected pneumonia for use as an epidemiological tool.
- To establish a mechanism for ensuring the quality of radiographic images stored in digital format.

Optimal interpretation of a chest radiograph will depend on the quality of the image and the methods used to interpret the image.

Computed Radiography (CR) digital system

Most CR systems are easy to configure and order, easy to install and to operate and are virtually maintenance free. Good CR systems provide excellent image quality. Systems use an Imaging Plate ("electronic" film) which is placed in a reader that can provide the image using standard computers.

The inherent sensitivity of CR systems is comparable to Speed 200 in Film screen systems. Depending on image quality requirements sensitivity in the range of 200 to 400 can be selected. Dose levels should be kept within the limits recommended for pediatric chest x-rays.

INTERPRETATION OF CHEST X-RAYS

All x-rays will be classified for quality and findings according to the scales in Appendix G Table 1 and Appendix G Table 2. For more details about these definitions, please refer to [WHO, 2001]. Note that for 'uninterpretable' chest x-rays, no further reading will be made.

Appendix G Table 1: Classification of quality of chest x-rays

**Uninterpretable**	an image is classified as “uninterpretable” if the features of the image are not interpretable without additional images. No further reading should be made for such images.
**Suboptimal**	an image is classified as “suboptimal” if the features allow interpretation of primary end-point but not of other infiltrates or findings. No entries should be made for other infiltrates for such images
**Adequate**	an image is classified as “adequate” if the features allow confident interpretation of end-point as well as other infiltrates

Appendix G Table 2: Classification of findings of chest x-rays

**Consolidation or pleural effusion**	where consolidation is defined as a dense opacity that may be a fluffy consolidation of a portion or whole of a lobe or of the entire lung, often containing air bronchograms* and where pleural effusion is defined if it occurs in the lateral pleural space (and not just in the minor or oblique fissure) and is spatially associated with a pulmonary parenchymal infiltrate (including other infiltrate) or if the effusion obliterates enough of the hemithorax to obscure an opacity<br>* atelectasis of an entire lobe that produces a dense opacity and a positive silhouette sign with the mediastinal border will be considered to be an endpoint consolidation<br>Amended 24 October 2008
**Other infiltrate**	linear and patchy densities (interstitial infiltrate) in a lacy pattern involving both lungs, featuring peribronchial thickening and multiple areas of atelectasis. Lung inflation is normal to increased. It also includes minor patchy infiltrates that are not of sufficient magnitude to constitute primary end-point consolidation, and small areas of atelectasis which in children can be difficult to distinguish from consolidation.
**No consolidation, infiltrate or effusion**	

Definitions of terms: for the purposes of this study. (See Appendix G Figure 1, below)
1) **Infiltrate**: any pathologic density in the lung.
2) **Alveoli**: tiny air-filled spaces where oxygen and $C0_2$ are exchanged (see diagram B)
3) **Bronchi**: tubes leading from the trachea to the alveoli
4) **Interstitium**: lung tissue outside the air-containing spaces: includes support tissues, blood vessels, bronchial walls, lymphatics
5) **Alveolar infiltrate**: alveoli filled with fluid (pus, edema, etc.)
6) **Heart and diaphragm borders**: (see diagram A).
7) **Air bronchogram**: branching linear lucent structure representing air still present in bronchi after the alveoli around them have consolidated; not to be confused with peribronchial thickening (an interstitial infiltrate)
8) **Consolidation**: especially dense, often homogeneous, confluent alveolar infiltrate sometimes may encompass an entire lobe or large segment, fluffy, mass-like, cloud-like density, erases heart and diaphragm borders (silhouette sign); often contains air bronchograms
9) **Atelectasis**: volume loss as air is absorbed from lung tissue, usually distal to an airway obstruction (e.g. a mucous plug). The lung tissue collapses like a Japanese fan, leaving a dense streak on the film that radiates outward from the hilum (see diagram D).
10) **Interstitial infiltrate**: includes peribronchial thickening and tiny areas of atelectasis (thought to be typical of viral infection).
11) **Pleural effusion:** fluid collecting in the pleural space around the lung, seen as a dense rim (the same density as the chest-wall muscles) interposed between the lung and the ribs (see diagram C)
12) **Peribronchial thickening or cuffing**: increased density of the walls of the smaller bronchi (away from the immediate hilar area) so that they become visible as circles or parallel lines (see diagram E)

Appendix G Figure 1: Diagrams to support classification of findings of chest x-rays

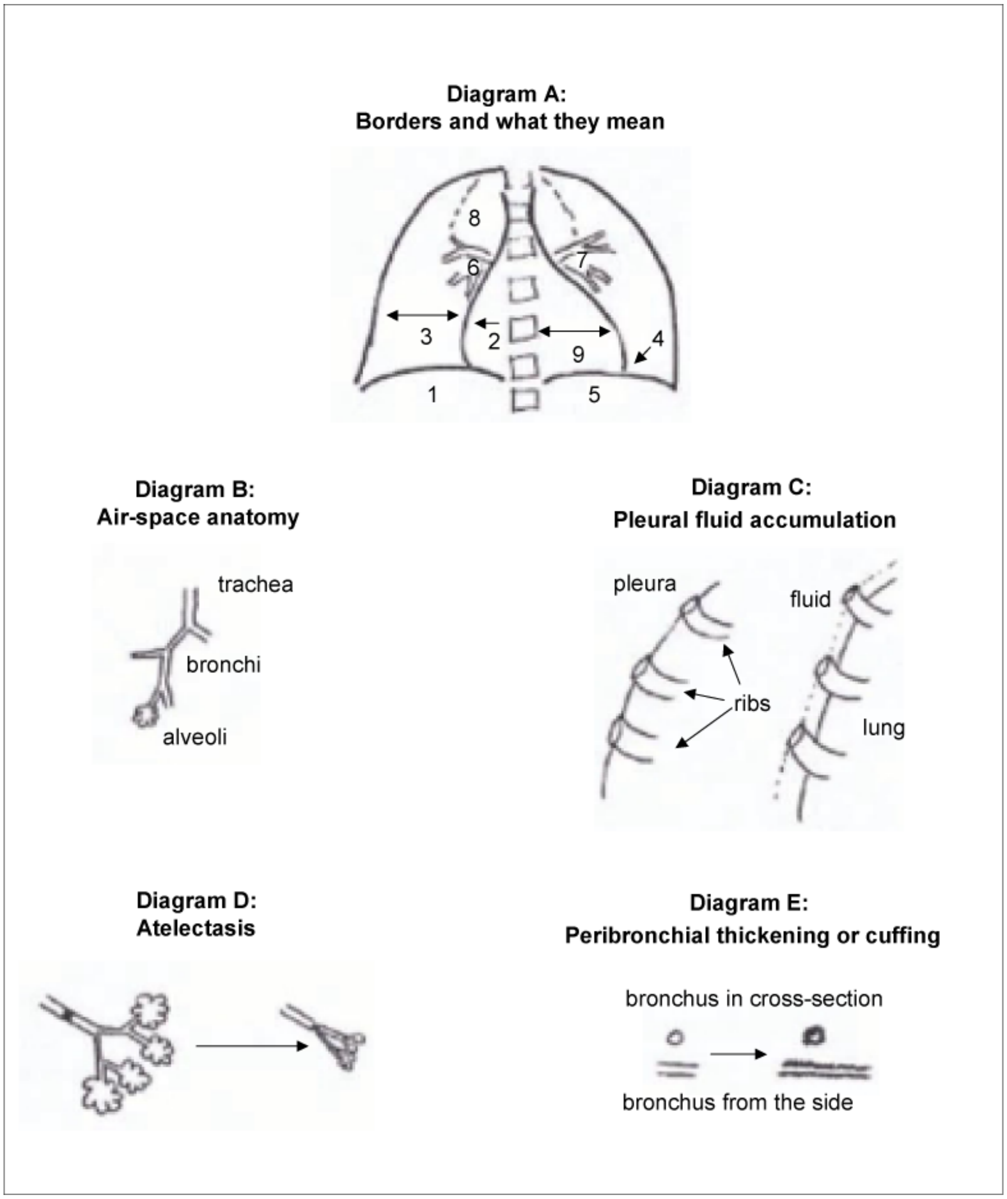

1) **Right diaphragm:** erased by a right lower lobe infiltrate.
2) **Right heart (right atrium):** erased by a right middle lobe infiltrate.
3) **Minor fissure:** divides the right upper lobe from the right middle lobe; seen as a line when there is fluid in it; seen as a border when there is infiltrate in the lobe adjacent to it.
4) **Left heart (left ventricle):** erased by an infiltrate in the lingula (homolog of the right middle lobe; actually a part of the left upper lobe).
5) **Left diaphragm:** erased by an infiltrate in the left lower lobe, often behind the heart shadow.
6) & 7) **Right hilum and left hilum:** contain large blood vessels, lymph nodes, and main bronchi; bronchial walls may be normally visible here, but should disappear quickly just outside the immediate hilar area.
8) **Thymus:** bi-lobed semi-lucent structure in the upper mid chest with defined borders, may resemble an upper lobe infiltrate but often shrinks when the child is sick.
9) **Heart:** from the spine to the left heart border should be the same density.

QUALITY ASSURANCE PROCESS FOR THE STANDARDIZED INTERPRETATION OF CHEST X-RAYS

Appendix G Figure 2: quality assurance process

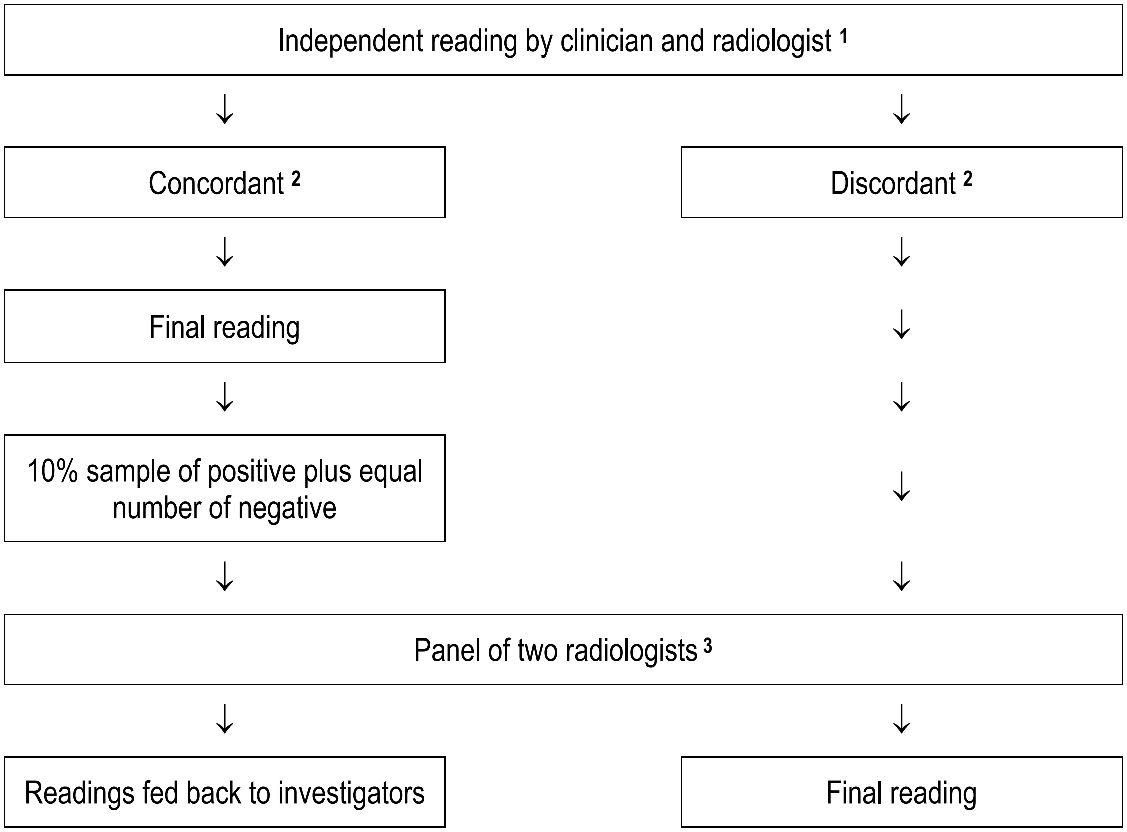

1. The first read of each CXR will be done by:
a clinician trained in per-protocol CXR interpretation attached to the site where the CXR is taken AND
a radiologist trained in per-protocol CXR interpretation
The CXRs will be classified according to quality (Appendix G Table 1) and findings (Appendix G Table 2). Note that for 'uninterpretable' chest x-rays, no further reading will be made.
2. Concordance will be defined as agreement on the presence or absence of consolidation or pleural effusion as defined in Appendix G Table 2.
3. A reference panel of two radiologists will be established. They will review all CXRs that are not concordant and, of those that are concordant. In addition, they will review a 10% sample of all concordant CXRs with presence of consolidation or pleural effusion and an equal number of absences of consolidation or pleural effusion as defined in Appendix G Table 2.

References for Appendix G

WHO. Standardization of interpretation of chest radiographs for the diagnosis of pneumonia in children. World Health Organization, Geneva, 2001.

Appendix H Microbiology methodology and interpretation of results

Blood culture methodology

Bactec bottles and automated Bactec incubators will be used.

The blood culture bottles will be inspected for damage or defect before use, and it will be ensured that the expiry date is not exceeded. It will be ensured that the blood culture bottles are stored according to the manufacturers specifications.

Blood will be withdrawn by peripheral venipuncture. To avoid contamination, the use of disposable plastic gloves will be encouraged. It is required to disinfect adequately the site of venipuncture. The skin will be cleaned with water and soap if necessary and disinfected with iodine or alcohol-based solution.

Samples will not be taken through an intravenous catheter or other access device unless no other access is available. Blood culture bottles will be inoculated immediately after blood collection. If blood samples for other tests are to be taken at the same venipuncture, the blood culture bottles should be inoculated first to avoid contamination.

Single pediatric bottles will be used. The recommended ideal volume for this purpose is 3 mL. The minimal volume inoculated will be 0.5 mL. The maximum recommended volume per bottle will not be exceeded.

Blood culturing should be started as soon as possible after sampling, using a licensed fully automated continuously monitoring blood culture system, detecting positive cultures based on CO_2 production. In the meanwhile, blood should not be stored in a refrigerator but at room temperature with air conditioning (15-25°C) out of direct sunlight.

Amended 26 November 2009

Once the incubator detects a positive sample, the sample will be processed as soon as possible and certainly less than 10 hours after detection of positivity. Positive cultures (or other types of cultures) will be sub-cultured by plating onto chocolate, sheep or horse blood agar and MacConkey agar plates and incubated aerobically with 5-10% CO_2 at 35°C for 48 hours. A sample of each positive blood culture bottle will be stored for possible further analysis in case the bacterial subculture was negative. Bacterial isolates will be kept in 20% skim milk medium or other cryo-conservation medium at -70 °C.

Amended 19 August 2008
Amended 26 November 2009

Blood culture result interpretation

The principles of interpretation will be according to previously described methodologies [WHO, 1999].

Blood culture will be considered to be positive if:

- a definite pathogen is isolated; examples are *Streptococcus pneumoniae*, *S. agalactiae*, *S. pyogenes*, *Haemophilus influenzae*, *Salmonella* spp., (this list is not exhaustive)
- a bacteria that could be either a pathogen or a contaminant is isolated within 48 hours of incubation; examples are *Escherichia coli*, *Klebsiella pneumoniae*, *Staphylococcus aureus*, *Enterococcus faecalis* (group D), (this list is not exhaustive)

Blood culture will be considered to be contaminated if:

- a known contaminant is isolated; examples are coagulase-negative staphylococci, *Micrococcus* spp., and *Bacillus* spp. (i.e., aerobic spore-bearing bacilli), (this list is not exhaustive)
- a bacteria that could be either a pathogen or a contaminant is isolated after more than 48 hours of incubation

Blood culture will be considered to be negative if:

- there is no bacteria isolated

Other body fluids

Cerebrospinal fluid will be collected by lumbar puncture following the same sterility precautions as described above. Cerebrospinal fluid will be collected in a sterile plastic collector tube and brought to the laboratory without delay after collection.

Cerebrospinal fluid and other normally sterile body fluids, such as pleural effusion, synovial fluid, peritoneal fluid or pericardial fluid, will be examined by direct smears and cultured in parallel on appropriate chocolate and blood agar plates. Samples may also be inoculated in automated culture bottles as are used for blood culture. Positive automated cultures will be sub-cultured by plating onto chocolate and blood agar plates and incubating aerobically with 5-10% CO_2 at 35°C for 48 hours.

Gram staining, white cell count and differential, latex antigen (*Streptococcus pneumoniae*, *Haemophilus influenzae*, *Neisseria meningitidis*) agglutination tests, glucose and protein concentration determination will be performed on cerebrospinal fluid by standard methods.

In case of macroscopic blood contamination of the cerebrospinal fluid, only culture results will be taken into account.

More details of microbiology procedures including bacterial isolates identification and the set up of laboratory internal and external Quality Assurance programs will be described in internal laboratory standard operating procedures.

Excess CSF may be frozen for future testing for infectious agents if applicable.

Amended 19 August 2008

References for Appendix H

WHO Young Infants Study Group. Bacterial etiology of serious infections in young infants in developing countries: results of a multicenter study. *Pediatr Infect Dis J*. 1999;18 (Suppl): S17-22)

Appendix I The WHO pediatric clinical staging system (for HIV disease)

The text below is an extract from the ‘Pocket book of hospital care for children: guidelines for the management of common illnesses with limited resources’ [WHO, 2005]. The document can currently be downloaded from http://whqlibdoc.who.int/publications/2005/9241546700.pdf (accessed 07 March 2008). Please refer to the current version of the guidelines for updated procedures.

For use in those <13 years with confirmed laboratory evidence of HIV infection (HIV AB where age >18 months, DNA or RNA virological testing for those age <18 months)

STAGE 3

Unexplained moderate malnutrition not responding to standard therapy

Unexplained persistent diarrhea (>14 days)

Unexplained persistent fever (intermittent or constant, for longer than 1 month)

Oral candidiasis (outside neonatal period)

Oral hairy leukoplakia

Pulmonary tuberculosis[1]

Severe recurrent presumed bacterial pneumonia (2 or more episodes in 6 months)

Acute necrotizing ulcerative gingivitis/periodontitis

LIP (lymphoid interstitial pneumonia)

Unexplained anemia (<8 g/dL), neutropenia (<500/mm^3) or thrombocytopenia (<30,000/mm^3) for >1 month

STAGE 4

Unexplained severe wasting or severe malnutrition not responding to standard therapy

Pneumocystis pneumonia

Recurrent severe presumed bacterial infections (2 or more episodes within one year, e.g. empyema, pyomyositis, bone or joint infection, meningitis, but excluding pneumonia)

Chronic orolabial or cutaneous herpes simplex infection (of >1 month duration)

Disseminated or extrapulmonary tuberculosis

Kaposi sarcoma

Oesophageal candida

Symptomatic HIV seropositive infant <18 months with 2 or more of the following; oral thrush, +/– severe pneumonia, +/– failure to thrive, +/– severe sepsis[2]

CMV retinitis

CNS toxoplasmosis

Any disseminated endemic mycosis including cryptococcal meningitis (e.g. extra-pulmonary cryptococcosis, histoplasmosis, coccidiomycosis, penicilliosis)

Cryptosporidiosis or isosporiasis (with diarrhea >1 month)

Cytomegalovirus infection (onset at age >1 month in an organ other than liver, spleen, or lymph nodes)

Disseminated mycobacterial disease other than tuberculous

Candida of trachea, bronchi or lungs

Acquired HIV-related recto-vesico fistula

Cerebral or B cell non-Hodgkin's lymphoma

Progressive multifocal leukoencephalopathy (PML)

HIV encephalopathy

HIV-related cardiomyopathy

HIV-related nephropathy

Footnotes:

1 TB may occur at any CD4 count and CD4 % should be considered where available

2 Presumptive diagnosis of stage 4 disease in seropositive children <18 month

Appendix J Documentation of bednet usage and indoor residual spraying

Bednet usage and indoor residual spraying will be assessed at two home visits at the timepoints 14 months from study start (Visit 16) (corresponding to the primary analysis) and at the last home visit, 11 months after the boost dose (Visit 31) prior to study conclusion.

During the extension, bednet usage and indoor residual spraying will be assessed at the field worker visit (Visit 37), within one month prior to the last extension visit (Visit 38).

Amended 01 December 2010

Bednets

The parent/guardian will be asked if the child sleeps under a bednet and whether or not the net is impregnated with insecticide. The child's bednet will be inspected and the integrity of the net (i.e. whether or not the net has holes) documented.

Net usage will be classified in the CRF/eCRF according to the following choices:

1. No bednet
2. Impregnated bed net with no hole large enough to admit three fingers
3. Impregnated bed net with at least one hole large enough to admit three fingers
4. Untreated bed net with no hole large enough to admit three fingers
5. Untreated bed net with at least one hole large enough to admit three fingers

Amended 26 November 2009

Indoor residual spraying

The parent/guardian will be asked if the house has been sprayed with a residual insecticide and if so when.

The question asked at Visit 16 will be

'Has indoor residual spraying been performed since vaccination 1?'.

The question at Visit 33 will be

'Has indoor residual spraying been performed since vaccination 4?'.

The response to these questions (yes/no) will be documented in the CRF/eCRF.

The investigator will endeavor to find out what chemical was used from the malaria control programs in the study area.

Appendix K Anthropometry methods

The methodologies used for anthropometry have been adapted from Cogill, 2003.

MEASURING WEIGHT IN INFANTS AND CHILDREN

Scales should be digital and for medical purposes (precision class III or IIII according to the European directive 90/384/CEE) . The scale should not be over-heated in the sun.

Children to the age of 2 years should be weighed nude using digital baby scales.

Children of 2 years and above should be weighed in light clothing without shoes using digital standing scales.

1. Measurer or assistant: Put the scale on an even surface enabling the reading to be clear. Ask the mother to undress the child as required above.
2. Measurer: Adjust the scale to zero.
3. Assistant: Stand behind and to one side of the measurer ready to record the measurement. Have the questionnaire ready.
4. Measurer and assistant: Check the child’s position. Make sure the child is lying or standing on the scale completely and not touching anything. Repeat any steps as necessary.
5. Measurer: Read the weight. Call out the measurement when the child is still and the scale is stationary.
6. Assistant: Immediately record the measurement and show it to the measurer.
7. Measurer: Check the recorded measurement for accuracy and legibility.

MEASURING LENGTH/HEIGHT FOR INFANTS AND CHILDREN

Measuring height for children 2 years and above

1. Measurer or assistant: Place the measuring board on a hard flat surface against a wall, table, tree, staircase, etc. Make sure the board is not moving.
2. Measurer or assistant: Ask the mother to remove the child’s shoes and unbraid any hair that would interfere with the height measurement. Ask her to walk the child to the board and to kneel in front of the child. If a Microtoise measure is used, stand the child vertically in the middle of the platform.
3. Assistant: Place the questionnaire and pencil on the ground (Arrow 1). Kneel with both knees on the right side of the child (Arrow 2).
4. Measurer: Kneel on your right knee on the child’s left side (Arrow 3). This will give you maximum mobility.
5. Assistant: Place the child’s feet flat and together in the center of and against the back and base of the board/wall. Place your right hand just above the child’s ankles on the

shins (Arrow 4), your left hand on the child's knees (Arrow 5) and push against the board/wall. Make sure the child's legs are straight and the heels and calves are against the board/wall (Arrows 6 and 7). Tell the measurer when you have completed positioning the feet and legs.

6. Measurer: Tell the child to look straight ahead at the mother who should stand in front of the child. Make sure the child's line of sight is level with the ground (Arrow 8). Place your open left hand under the child's chin. Gradually close your hand (Arrow 9). Do not cover the child's mouth or ears. Make sure the shoulders are level (Arrow 10), the hands are at the child's side (Arrow 11), and the head, shoulder blades and buttocks are against the board/wall (Arrows 12, 13, and 14). With your right hand, lower the headpiece on top of the child's head. Make sure you push through the child's hair (Arrow 15).
7. Measurer and assistant: Check the child's position (Arrows 1-15). Repeat any steps as necessary.
8. Measurer: When the child's position is correct, read and call out the measurement to the nearest 0.1 cm. Remove the headpiece from the child's head and your left hand from the child's chin.
9. Assistant: Immediately record the measurement and show it to the measurer.
10. Measurer: Check the recorded measurement for accuracy and legibility

Appendix K Figure 1 Child height measurement - height for children 2 years and older [United Nations, 1986]

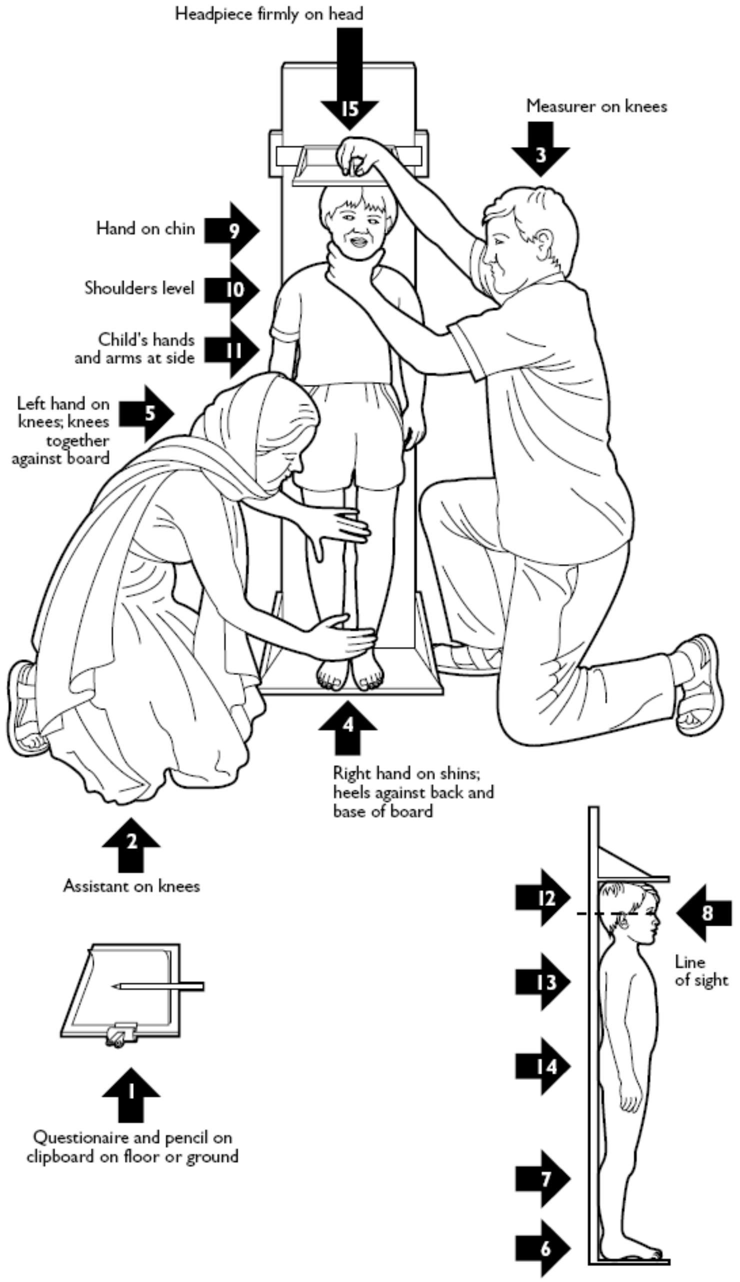

Measuring length for infants and children to the age of 2 years

1. Measurer or assistant: Place the measuring board on a hard flat surface, i.e., ground, floor, or steady table.
2. Assistant: Place the questionnaire and pencil on the ground, floor, or table (Arrow 1). Kneel with both knees behind the base of the board if it is on the ground or floor (Arrow 2).

3. Measurer: Kneel on the right side of the child so that you can hold the foot piece with your right hand (Arrow 3).
4. Measurer and assistant: With the mother's help, lay the child on the board by supporting the back of the child's head with one hand and the trunk of the body with the other hand. Gradually lower the child onto the board.
5. Measurer or assistant: Ask the mother to kneel close on the opposite side of the board facing the measurer as this will help to keep the child calm.
6. Assistant: Cup your hands over the child's ears (Arrow 4). With your arms comfortably straight (Arrow 5), place the child's head against the base of the board so that the child is looking straight up. The child's line of sight should be perpendicular to the ground (Arrow 6). Your head should be straight over the child's head. Look directly into the child's eyes.
7. Measurer: Make sure the child is lying flat and in the center of the board (Arrows 7). Place your left hand on the child's shins (above the ankles) or on the knees (Arrow 8). Press them firmly against the board. With your right hand, place the foot piece firmly against the child's heels (Arrow 9).
8. Measurer and assistant: Check the child's position (Arrows 1-9). Repeat any steps as necessary.
9. Measurer: When the child's position is correct, read and call out the measurement to the nearest 0.1 cm. Remove the foot piece and release your left hand from the child's shins or knees.
10. Assistant: Immediately release the child's head, record the measurement, and show it to the measurer.
11. Measurer: Check the recorded measurement for accuracy and legibility

Appendix K Figure 2 Child length measurement - length for infants and children to the age of 2 years [United Nations, 1986]

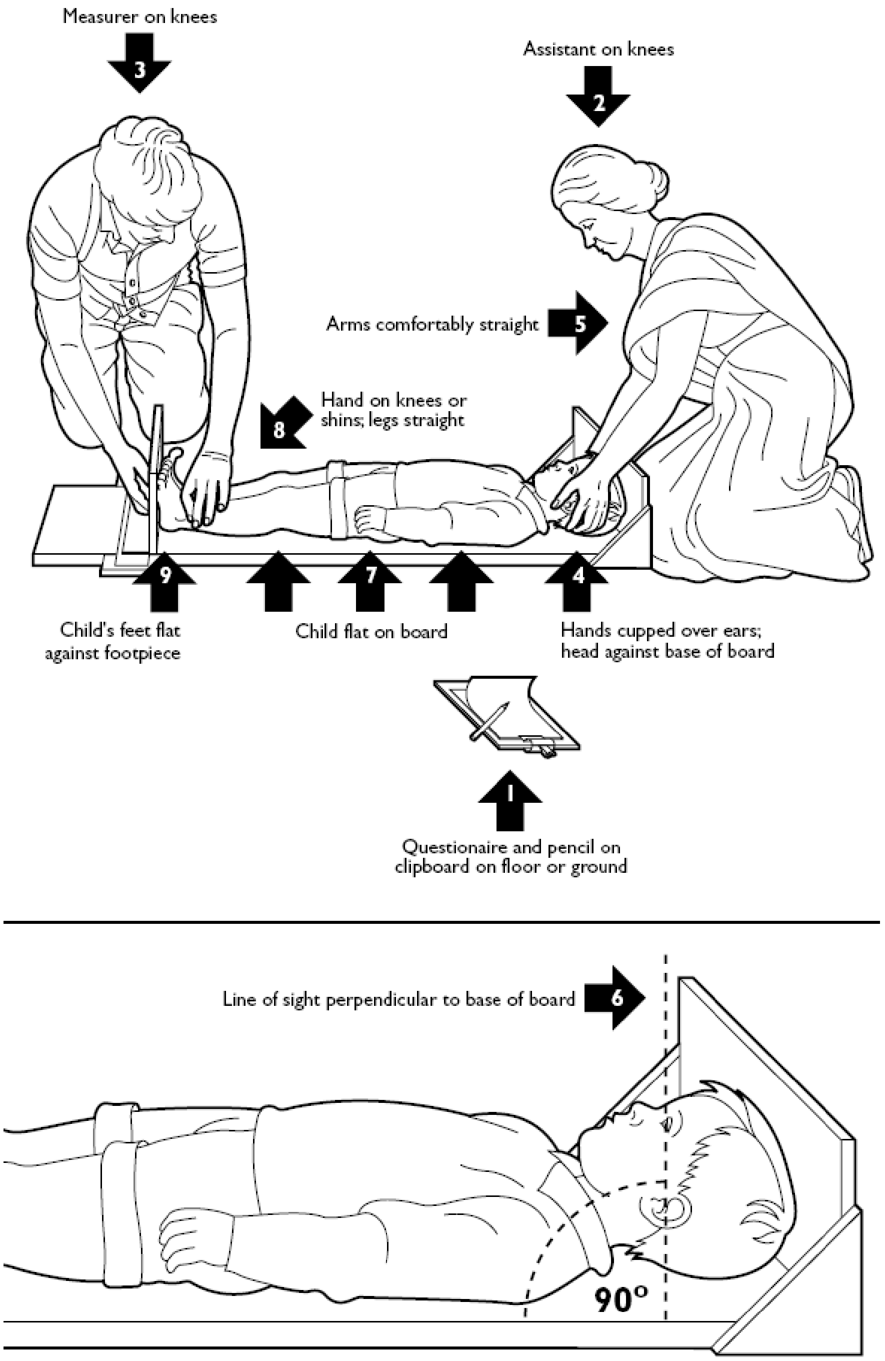

MEASURING CHILD MID-UPPER ARM CIRCUMFERENCE (MUAC)

1. Measurer: Keep your work at eye level. Sit down when possible. Very young children can be held by their mother during this procedure. Ask the mother to remove clothing that may cover the child's left arm.
2. Measurer: Calculate the midpoint of the child's left upper arm by first locating the tip of the child's shoulder (Arrows 1 and 2) with your finger tips. Bend the child's elbow to make a right angle (Arrow 3). Place the tape at zero, which is indicated by two arrows, on the tip of the shoulder (Arrow 4) and pull the tape straight down past the tip of the elbow (Arrow 5). Read the number at the tip of the elbow to the nearest centimeter. Divide this number by two to estimate the midpoint. As an alternative, bend the tape up to the middle length to estimate the midpoint. A piece of string can also be used for this purpose. Either you or an assistant can mark the midpoint with a pen on the arm (Arrow 6).
3. Measurer: Straighten the child's arm and wrap the tape around the arm at midpoint. Make sure the numbers are right side up. Make sure the tape is flat around the skin (Arrow 7).
4. Measurer and assistant: Inspect the tension of the tape on the child's arm. Make sure the tape has the proper tension (Arrow 7) and is not too tight or too loose (Arrows 8-9). Repeat any steps as necessary.
5. Measurer: When the tape is in the correct position on the arm with the correct tension, read and call out the measurement to the nearest 0.1cm (Arrow 10).
6. Assistant: Immediately record the measurement on the questionnaire and show it to the measurer.
7. Measurer: While the assistant records the measurement, loosen the tape on the child's arm.
8. Measurer: Check the recorded measurement for accuracy and legibility
9. Measurer: Remove the tape from the child's arm.

Appendix K Figure 3 Child mid-upper arm circumference measurement [United Nations, 1986]

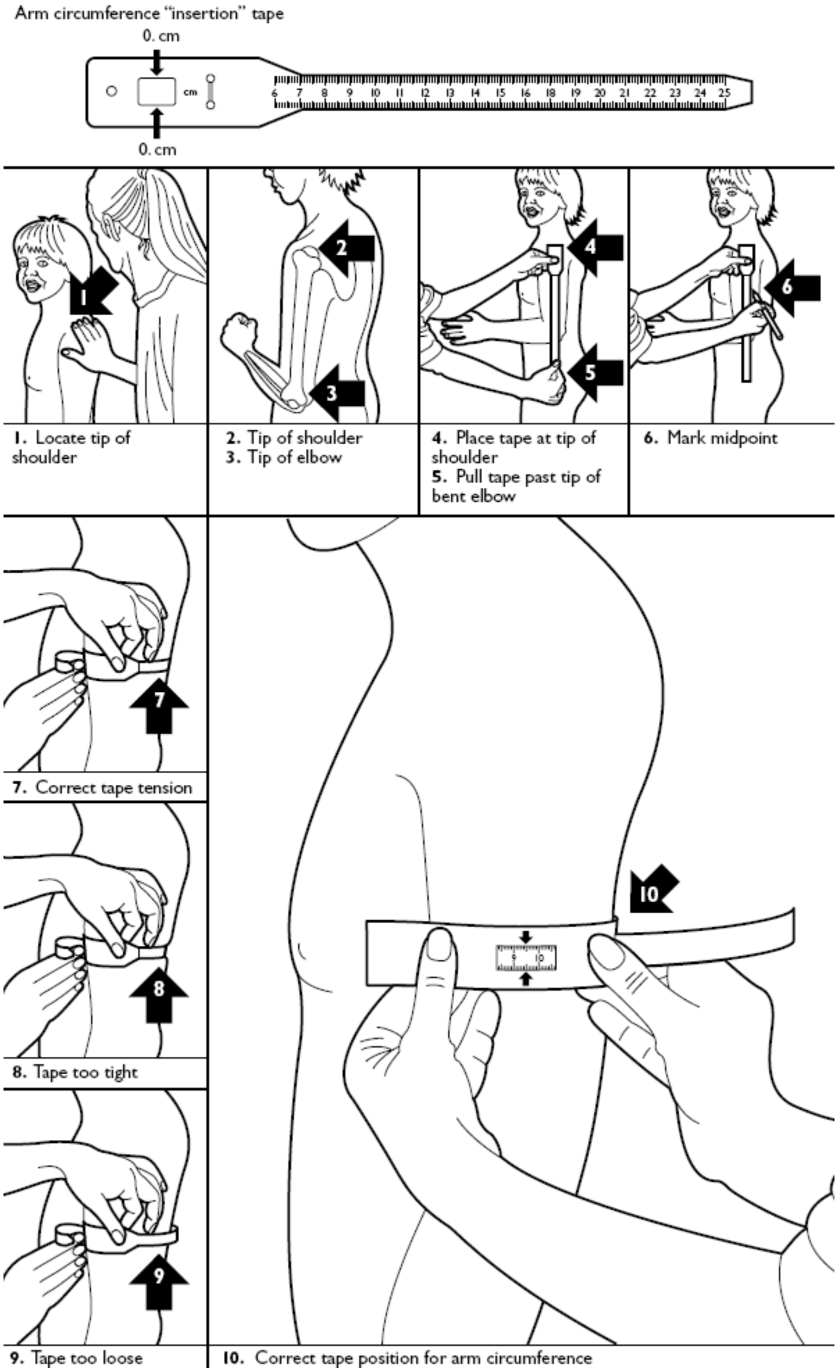

References for Appendix K

Cogill, B. Anthropometric Indicators Measurement Guide. Food and Nutrition Technical Assistance Project, Academy for Educational Development - Chapter 5: Taking Measurements. Washington, DC, 2003.

United Nations. How to Weigh and Measure Children: Assessing the Nutritional Status of Young Children. 1986.
