# Supplementary material for: Efficacy and Safety of the RTS,S/AS01 Malaria Vaccine during 18 Months after Vaccination: A Phase 3 Randomized, Controlled Trial in Children and Young Infants at 11 African Sites
Source: PLoS Med. 2014 Jul 29;11(7):e1001685. doi: 10.1371/journal.pmed.1001685 (PMC4114488; doi:10.1371/journal.pmed.1001685)
Supplement: Text S1 — Supplementary methods. (DOCX) [file pmed.1001685.s029.docx]

**Supplementary Methods**

TABLE OF CONTENTS

Page

1. Authors and Affiliations 2

2. Supplementary Methods 2

2.1. Ethical considerations 2

2.2. Roles of investigators and sponsor 3

2.3. Study sites and affiliated partners 3

2.4. Screening and informed consent 3

2.5. Randomization and blinding 6

2.6. Study vaccines 7

2.7. Bednets and indoor residual spraying (IRS) 9

2.8. Safety assessments 10

2.9. Surveillance for clinical and severe malaria episodes 12

2.10. Chest radiographs 13

2.11. Anthropometry 15

2.12. Laboratory analysis 15

2.13. Immunological assessment 18

2.14. Data collection, data management and statistical analysis 19

2.15. Contribution to the per-protocol analyses 19

2.16. Statistical methods for the analyses of efficacy at Month 20 20

2.17. Additional analyses 26

2.18. Major protocol deviations 27

2.19. Trademarks 27

3. Groups that have contributed to the delivery of this trial 28

4. References 28

# Authors and Affiliations

Submitted by the RTS,S Clinical Trials Partnership.

# Supplementary Methods

This supplement describes general information on the study population and study conduct with a particular emphasis on the statistical methods used for this analysis.

## Ethical considerations

This phase III, double-blind (observer-blind), randomized, controlled multicenter study is currently being undertaken in 11 sites across sub-Saharan Africa. The study design and rationale for selection of endpoints have been described previously.[1] The study is being conducted in accordance with the current Declaration of Helsinki, International Committee on Harmonization Good Clinical Practice guidelines [2] and with the local rules and regulations of each country. The study is monitored by the sponsor, GlaxoSmithKline (GSK) Biologicals SA (GSK monitors or outsourced monitors from Quintiles [Quintiles, Centurion, South Africa] contracted by GSK Biologicals SA), and overseen by a formally constituted Independent Data Monitoring Committee (IDMC), that reviewed, among other information, unblinded comprehensive safety data every three months to authorize trial continuation. The IDMC conferred before the initiation of the study and has had three-monthly teleconferences and one annual meeting thereafter. A Local Safety Monitor, who is an experienced clinician not taking part in the study, was available at each study site to support the clinical investigators and to act as a link between the investigators and the IDMC. The study protocol and amendments, consent forms, and other information that required pre-approval were reviewed and approved by a national, regional, or research center ethics committee (EC) or institutional review board (IRB) as per local requirements. A list of all EC/IRBs is provided in supplementary table 1a.

## Roles of investigators and sponsor

The study is sponsored by GSK Biologicals SA, the vaccine developer and manufacturer, and funded by both GSK Biologicals SA and the PATH Malaria Vaccine Initiative (MVI).The study was designed by the Clinical Trials Partnership Committee (CTPC), consisting of representatives of all research sites, study sponsor and study funders (as detailed in [1]). All authors were involved in data collection. All data were analyzed following a pre-defined analysis plan. The CTPC had full access to the study data, made the decision to publish the manuscript in its current form, and prepared the manuscript.

## Study sites and affiliated partners

The study is being conducted in 11 sites located in seven countries in sub-Saharan Africa and involves collaboration with partner institutions. The study sites represent the range of malaria transmission seen across sub-Saharan Africa (Figure 1 in the paper). The list of study sites and affiliated partners is provided in supplementary table 1b.

## Screening and informed consent

Two age-categories of children were eligible for inclusion in the trial. One age-category comprised infants who were 6-12 weeks of age (inclusive) at the time of first vaccination and who had not previously received a dose of vaccine against diphtheria, tetanus, pertussis or *Haemophilus influenzae* type b and the other age-category comprised children 5- 17 months of age (inclusive) at the time of first vaccination. Participants should not have received any vaccine within the seven days preceding the first dose of study vaccine. Screening procedures included a review of the child’s medical history, a physical examination and a blood test for assessment of hemoglobin concentration. The main exclusion criteria were: moderate or severe illness at the time of enrolment, a major congenital defect, malnutrition requiring hospitalization, severe anemia - defined as a hemoglobin concentration < 5.0 g/dL or a hemoglobin concentration < 8.0 g/dL associated with clinical signs of heart failure or severe respiratory distress, or a past history of a neurological disorder or of an atypical febrile seizure. A past history of a simple febrile seizure was not an exclusion criterion. Children with active HIV disease of Stage III or Stage IV severity, as defined by the World Health Organization, at the time of screening were excluded.[3] A previous history of active Stage III or Stage IV HIV disease was not an exclusion criterion.

There was no routine testing for HIV in this trial. HIV positive cases were reported on the general medical history taken at screening or identified by morbidity surveillance during the trial. The decision to report a new HIV infection depended on the investigators judgment as to whether it met the criteria for a serious adverse event. Likewise, it was at the investigators discretion whether to perform antibody or PCR confirmatory testing. Voluntary counseling and testing, highly active anti-retroviral therapy (HAART) and prevention of mother to child transmission (PMCT) were available at all study sites according to national policies.

Prior to enrolment, study teams conducted a series of information activities. Study teams held discussion meetings with the administrative leaders and/or community leaders. They described the outline of the proposed study, paying particular attention to study procedures including screening of children, immunization, blood collection, follow-up and their associated risks.

Following the community meetings, and a positive recommendation from community leaders, the parent(s)/guardian(s) of children in the eligible age-categories were approached. The need for a vaccine against malaria was discussed and the objectives of the study were explained. The study procedures were described carefully including the blinding of study treatment, the immunization and blood collection. Parent(s)/guardian(s) interested in enrolling their child into the study were invited to the screening visit.

The site investigator or his/her designate described the protocol to the parent(s)/guardian(s) of potential participating children face to face or the informed consent information was presented to groups at an initial information session. Information was provided in both oral and written form in a language fully comprehensible to the child’s family. Each child’s family had the opportunity to inquire about details of the study and ask any questions individually in a private place. Formal informed consent was obtained from each child’s parent(s) or guardian(s) prior to the performance of any study-specific procedures. Literate parent(s)/guardians willing to let their child enter into the study were asked to sign and date the informed consent form (ICF). If the parents or guardians were illiterate, the study and the ICF were explained point by point in the presence of an impartial witness. The impartial witness could be a friend or family member accompanying the parents or any other literate person independent from the study team. Parent(s)/guardian(s) confirmed their consent for their child to take part in the study by marking the ICF with their thumbprint and the impartial witness personally signed and dated the ICF.

## Randomization and blinding

After verification of eligibility criteria, and prior to the first vaccination, a unique treatment number was assigned to each participating child. Participating children from each age category were randomized into one of three study groups according to a 1:1:1 ratio (R3R, R3C or C3C) using a randomization algorithm with SAS version 9.1 (Supplementary figure 1). Randomization was stratified for age category using study site as a minimization factor, ensuring balanced treatment allocation within each study site. All children’s parent(s)/guardian(s) were provided with a study identification card with a photo of their child, the child’s name and a unique subject number. All data were collected using remote data entry (RDE) and electronic case report forms (eCRF).

Data were collected in a double-blinded (observer-blind) manner; the vaccinated children and their parent(s)/guardian(s) as well as those responsible for the evaluation of study endpoints were unaware of whether RTS,S/AS01 or control vaccine had been administered to a particular child. The vaccines used in this study were of different appearance. The content of the syringe was, therefore, masked with an opaque tape to ensure that parent(s)/guardian(s) were blinded. The only study staff who knew of the vaccine assignment were those responsible for preparation and administration of vaccines; these staff played no other role in the study except screening or collection of biologic specimens.

## Study vaccines

Each child received three doses of either the candidate malaria vaccine RTS,S/AS01 or the control vaccine. In the 5-17 months age-category, the control vaccine was a rabies vaccine VeroRab™ (Sanofi-Pasteur) and in the 6-12 weeks age-category the control vaccine was a Meningococcal C conjugate vaccine Menjugate™ (Novartis). Vaccines were administered intramuscularly into the left deltoid for the 5-17 months age category and into the left anterolateral thigh for the 6-12 weeks age-category. The choice of control vaccines was guided by the principles of benefit to the control group without compromising the evaluation of clinical study endpoints. Infants enrolled in the 6-12 weeks age-category received the RTS,S/AS01 or control vaccine in co-administration with the DTPwHepB/Hib pentavalent vaccine (Tritanrix™ HepB/Hib, GSK Vaccines) administered into the right anterolateral thigh and an oral polio vaccine containing serotypes 1, 2 and 3 (Polio Sabin™, GSK Vaccines).

The RTS,S/AS01 candidate vaccine has been developed and manufactured by GSK Vaccines and is designed to protect against *P. falciparum* malaria. Manufacturing and quality control are performed in line with current Good Manufacturing Practices. No quality issues in the vaccines used in this trial were recorded. “RTS,S” comprises the carboxyl terminal portion (amino acids 207 to 395) of the circumsporozoite protein from the NF54 strain of *P. falciparum* fused to the hepatitis B surface antigen, co-expressed in yeast with non-fused hepatitis B surface antigen."AS01" describes the Adjuvant System comprising liposomes, MPL (3-O-desacyl-4’-monophosphoryl lipid A) and QS-21* (a triterpene glycoside purified from the bark of *Quillaja saponaria*).
* QS-21 is licensed from Antigenics Inc, a wholly owned subsidiary of Agenus Inc.

Each dose of reconstituted RTS,S/AS01 (0.5 mL) contains approximately 25 μg of antigen, 25 μg of MPL and 25 μg of QS-21 with liposomes.[4]

Sanofi-Pasteur’s chromatographically purified Vero cell culture rabies vaccine VeroRab™ is based on inactivated Wistar Rabies PM/W138 1503-3M strain and it is given in a ≥2.5 IU/0.5 mL dose.

One dose (0.5 mL) of Novartis’s Meningococcal C conjugate vaccine contains 10 μg *Neisseria meningitidis* (strain C11) group C oligosaccharide conjugated to 12.5‑25 μg *Corynebacterium diptheriae* CRM_197_ protein adsorbed on aluminum hydroxide (1.0 mg). The excipients of the reconstituted vaccine include mannitol, sodium phosphate monobasic monohydrate, sodium phosphate dibasic heptahydrate, sodium chloride and water for injections.

GSK Vaccines’ DTPwHepB/Hib vaccine is prepared by reconstitution of the Hiberix™ pellet with the Tritanrix™ HepB suspension. Each 0.5 mL dose contains not less than 30 IU of adsorbed diphtheria toxoid, not less than 60 IU of adsorbed tetanus toxoid, not less than 4 IU of whole cell pertussis, 10 μg of recombinant hepatitis B antigen (HBsAg) protein and 10 μg of purified capsular polyribosyl ribitol phosphate covalently bound to approximately 30 μg tetanus toxoid. Tritanrix™ HepB also contains 2-phenoxyethanol, polysorbate 20, sodium chloride, thiomersal and water for injection. Hiberix™ also contains lactose.

The oral polio vaccine obtained from GSK Vaccines is a stabilized suspension of types 1, 2 and 3 live attenuated polioviruses (Sabin strains): Type 1 (strain LSc, 2ab), Type 2 (strain P 712 ch, 2ab), Type 3 (strain Leon 12a, 1b). The excipients comprise magnesium chloride, L-arginine, polysorbate 80, neomycin sulphate (residual), polymyxin B sulphate (residual) and purified water.

Children were observed closely for at least 30 minutes after vaccination, with appropriate medical treatment and equipment readily available in case of an anaphylactic reaction. A study clinician accredited in pediatric resuscitation was available at all vaccination sessions.

## Bednets and indoor residual spraying (IRS)

The research team ensured that insecticide treated bednet use was optimized in each study population: in two study sites (Kilifi, Kenya and Bagamoyo, Tanzania) this was achieved through close collaboration with the respective National Malaria Control Programs. In the other sites, impregnated bednets were distributed by the study teams to all children who underwent screening, regardless of whether they were eligible for the trial.

Data were collected on malaria control measures used by the participants’ families during the period of surveillance. Bednet usage and indoor residual spraying (IRS) were documented 12 months after the third vaccine dose had been given. Children’s parents were asked if their house had been sprayed with a residual insecticide and, if so, when this was done. Then they were asked if their child sleeps under a bednet. During a home visit, a field worker inspected the child’s bednet and the integrity of the net was recorded as follows: 1: no bednet; 2: impregnated bednet with no hole large enough to admit three fingers; 3: impregnated bednet with at least one hole large enough to admit three fingers; 4: untreated bednet with no hole large enough to admit three fingers; 5: untreated bednet with at least one hole large enough to admit three fingers.

## Safety assessments

During the study, investigators or their designates were responsible for documenting and reporting events meeting the criteria and definition of an adverse event (AE) or serious adverse event (SAE). Parents/guardians of children participating in the study were requested to contact study personnel immediately if their child showed any signs or symptoms they perceived as serious.

An adverse event was defined as any untoward medical occurrence in a child participating in the clinical trial, temporally associated with vaccination whether or not considered related to the vaccine. An AE could, therefore, be any unfavorable and unintended sign (including an abnormal laboratory finding), symptom, or disease (new or exacerbated) temporally associated with vaccination.

For the purpose of this study, a serious adverse event was defined as any untoward medical occurrence that resulted in death, was life-threatening, required hospitalization or prolongation of existing hospitalization, resulted in disability/incapacity, or a seizure that occurred within 30 days of vaccination. Abnormal laboratory findings that were judged by the assessing clinician to be clinically significant were recorded as an SAE if they met the criteria for an SAE as defined above.

Seizures occurring within 30 days of vaccination and immune-mediated disorders occurring at any time during the study were reported as SAEs in order to ensure availability of full case narrative descriptions.[1] Data on seizures occurring within seven days following vaccination were collected and analyzed according to the Brighton Collaboration guidelines[5] and have been published previously.[6,7]

Because pediatric auto-immune diseases are rare and may be underestimated in sub-Saharan Africa, training material on pediatric auto-immune disease presentation and diagnosis was provided by the study sponsor. A specific, standardized clinical data collection questionnaire was generated. Collaborations with reference laboratories in South Africa were initiated so that serum samples or histopathologic specimens could be sent to South Africa for analyses not available locally.

In this trial, diagnosis for all adverse events, including the diagnosis of meningitis, is based on all available clinical evidence and is not bound by stringent laboratory or diagnostic criteria. Efforts have been made both prospectively and retrospectively to confirm diagnoses of meningitis on cerebrospinal fluid (CSF) examination when available using biochemical, microbiologic and molecular testing as described in section 2.12. The IDMC also reviewed unblinded safety reports containing specific sections on seizures and meningitis. The IDMC recommended study continuation and will continue to review results from further investigations.

SAEs were collected for all participating children throughout the study period, from the time of parental consent. At every visit/contact, information was sought on the occurrence of AEs/SAEs. SAEs were identified by surveillance at health facilities in the study area and through monthly home visits with the participating children. All AEs that were observed directly or that were observed by a clinical collaborator, those that were identified through surveillance at health facilities in the study area or those reported by the child’s parent/guardian spontaneously or in response to a direct question were evaluated.

Assessments were made of the maximum intensity of all unsolicited AEs and SAEs during the period of the event. The assessment was based on the attending clinician’s medical judgment. A grade was assigned to all adverse events as follows; grade 1 (mild): an AE which is easily tolerated by the child, causing minimal discomfort and not interfering with everyday activities; grade 2 (moderate): an AE which is sufficiently discomforting to interfere with normal everyday activities and grade 3 (severe): an AE which prevents normal, everyday activities.

SAEs were coded according to the MedDRA (Medical Dictionary for Drug Regulatory Activities). Non-malaria SAEs were defined as those which excluded the MedDRA terms “*Plasmodium falciparum* infection”, “Malaria” and “Cerebral malaria”.

Verbal autopsies were carried out on all children who died outside a health facility to ascribe the cause of death using a questionnaire based on the International Network for the Demographic Evaluation of Populations and Their Health in Developing Countries (INDEPTH) standard questionnaire, adapted to be locally appropriate.[8] To support the timely reporting of SAEs, diagnoses were made according to the usual processes of each study site.

## Surveillance for clinical and severe malaria episodes

During the informed consent process, parents were asked to bring their child to a study health facility as soon as possible if their child fell sick during the trial. All participating children who presented to a health facility in the study area were evaluated as potential cases of malaria using a standardized algorithm. All parents were asked whether the child had had a fever within the previous 24 hours and all children had their temperature measured*.* A blood sample was taken for testing for malaria parasites in all children who had a history of fever during the previous 24 hours or who had a measured axillary temperature ≥ 37.5°C at the time of presentation.

Children who needed inpatient care were provided transport to a hospital participating in the trial. All participating children who presented for admission were evaluated as a potential case of severe malaria following a predefined algorithm (Supplementary table 3). Detection and management of severe malaria have been described in detail by Vekemans et al.[9] During any hospitalization, the child’s course was monitored to capture the signs and blood parameters indicative of progression to severe malaria. If a child’s condition deteriorated following admission then additional investigations were performed.

Treatment of malaria was conducted in accordance with national guidelines. In nine of the 11 study sites, the first line treatment for uncomplicated malaria was a 6-dose regimen of artemether-lumefantrine whilst in two, both in Ghana, it was artesunate-amodiaquine. Children who required inpatient care were admitted to the hospital and received treatment with intravenous quinine, according to national guidelines.

## Chest radiographs

Chest radiographs were performed as part of the standardized evaluation of study participants brought to a healthcare facility with tachypnea, lower chest wall indrawing, abnormally deep breathing or if a study clinician considered this to be an appropriate investigation.[9]

A digital radiography system was provided to each study site to facilitate radiological assessment of study participants. The radiographers and the physicians who read the images for the trial endpoints received standardized technical training by the manufacturer of the radiography equipment and training on interpretation of chest radiograph images by expert radiologists and physicists. To ensure a robust and verifiable radiograph data base, quality control systems that included local on-site training, development of quality manuals, quality control checks, on-site radiology committees and external audits were implemented. Digital images were anonymized and sent to a central repository at GSK Vaccines via a satellite internet connection.

For the purpose of endpoints assessment, to ensure accurate diagnosis of pneumonia, a process developed by WHO [10] was followed. Each radiograph was read independently by a clinician attached to the center where the radiograph was taken, and by an external radiologist. GSK Vaccines reviewed all readings made by the sites and by the external radiologists and any images with discordant readings were sent to another panel of radiologists for a final reading. The reporting of pneumonia as a SAE was made based on clinicians’ judgment and independent of this protocol-specific assessment.

Clinicians and external radiologists were trained in chest radiograph interpretation according to WHO guidelines.[10]

## Anthropometry

Length/height, weight and mid-upper arm circumference were measured at screening and one month after the third dose of vaccine. The methodologies used for anthropometry were adapted from Cogill.[11]

## Laboratory analysis

Development of standardized laboratory methods and quality control processes for this trial have been described fully in a separate publication [12] and are summarized briefly here.

- ***P. falciparum* counts by blood smear**

All slides were read independently by two trained microscopists. A third independent microscopist read the slide if there was any of the following discrepancies between the first two readings: (1) a positive reading by one microscopist and a negative reading by the other; (2) both microscopists recorded a parasitemia >400 parasites/μL but the higher count divided by the lower count was >2; (3) at least one microscopist recorded a parasitemia ≤400 parasites/μL but the higher reading was more than 10 times the lower reading.

If the initial two readings gave concordant results, the final parasite density was considered to be the geometric mean of the two readings. If the readings were discordant, then the following principles were applied: (1) where one reading was positive and the other negative, the majority decision obtained following the reading by the third microscopist was adopted and, when the slide was considered positive, the parasite density was recorded as the geometric mean of the two positive results; (2) when all three readings were positive, the final result was the geometric mean of the two closest readings (in log scale). As a quality measure, agreement between the two microscopists was calculated by means of the Kappa statistic.

Internal QC was performed on one negative and one positive slide for each batch of stain. The External QA process for slide reading comprised species identification and parasite quantification. Three assessments per year were carried out, including 20 samples per microscopist. Microscopists who were below the level defined as competent were considered to be 'in training' and were not allowed to read study slides until they were retrained and re-assessed.

- ***Hematology and biochemistry***

Automated biochemical and hematologic methods were used. All biochemistry automated analyzers were enrolled initially with International External Quality Assessment (EQA) but later switched to the program run by the Royal College of Pathologists of Australia, because the latter was more appropriate for the study requirements at the time. All hematology automated analyzers were enrolled in EQA. Each laboratory had to demonstrate method qualification for biochemistry and hematology, including analysis of repeatability, reproducibility, linearity, QC stability and accuracy between main and back-up analyzers. Data were sent to GSK Vaccines for analysis and feedback was provided to laboratories.

Daily internal QC was performed at each laboratory, and external quality control was performed monthly for biochemistry and hematology samples.

- ***Microbiology***

Standard microbiology methods for blood and CSF culture were followed using automated Bactec^TM^ incubators and pediatric bottles (Bactec BD Diagnostic Systems, USA). Positive cultures were sub-cultured using standard methods.[13, 14] For the purpose of trial analysis, as opposed to clinical care, results were classified by standardized case definitions based on an established methodology.[15] A blood culture was considered positive if a definite pathogen was isolated (e.g. *Streptococcus pneumoniae*, *S. agalactiae*, *S. pyogenes*, *Haemophilus influenzae*, Salmonella species) or if a bacterium that could be either a pathogen or a contaminant was isolated within 48 hours of incubation (e.g. *Escherichia coli*, *Klebsiella pneumoniae*, *Staphylococcus aureus*, *Enterococcus faecalis*). A blood culture was considered to be contaminated if a known contaminant was isolated or if a bacterium that could be either a pathogen or a contaminant was isolated after 48 hours of incubation.[15]

CSF was examined by Gram stain and a white cell count was performed using a hemocytometer. Direct agglutination methods using commercial kits (Remel Wellcogen Bacterial Meningitis Antigen Latex Kit or BIO-RAD Pastorex Meningitis Kit) were used for early detection of specific organisms like *S. pneumoniae,* group B streptococci*, H. influenzae* type b*, E. coli* and *Neisseria meningitidis.* In parallel, CSF was inoculated directly onto recommended culture media and in the same bottles used for blood culture in automated incubators to allow for bacterial growth, identification and antimicrobial sensitivity testing using the disk diffusion method.

For the assessment of protocol endpoints, bacterial meningitis was defined as the presence of a CSF white cell count of ≥50 x 10^6^/L, a positive CSF culture of compatible organisms or a positive CSF latex agglutination test for either *H. influenzae* type b (Hib), *N. meningitidis* or *S. pneumoniae*.[9, 16] The reporting of a meningitis case as an SAE was independent of this definition. SAE diagnoses were made by the study clinicians, using clinical judgment, based on the clinical and laboratory evidence available.

Microbiology quality assessment included evaluation of microscopy, culture, identification and antimicrobial susceptibility testing. Each laboratory received six samples (with at least two meningeal and two enteric organisms) three times per year, and the criteria of acceptability were defined by the National Institute of Communicable Disease (NICD, South Africa). Internal quality control was performed using American Type Culture Collection (ATCC) control strains for species identification every week or when a new batch of reagent was received or when discordant results were obtained. The contamination rate of the clinical specimens was evaluated monthly by internal assessment. Continuous assessment allowed re-training programs for both clinical and laboratory staff and more intense quality evaluation when there was a high contamination rate.

## Immunological assessment

Antibodies specific for the circumsporozoite protein tandem repeat epitope were assessed by a standard, validated ELISA with plates adsorbed with the recombinant antigen R32LR that contains the sequence [NVDP(NANP)15] 2LR as described previously.[17] Briefly, R32LR protein was coated onto a 96-well polystyrene plate. Serial dilutions of serum were added to the 96-well plate and, after incubation, the plates were washed and Horseradish Peroxidase conjugated polyclonal rabbit anti-human IgG was added. After a final washing step, a color reaction was developed with 3, 3',5,5' tetramethylbenzidine and the plates were read in an ELISA reader. Antibody concentrations were calculated from a standard curve with the software SoftMax^®^ Pro (using a four parameters equation) and expressed as EU/mL. Anti-CS antibodies were measured at the CEVAC Laboratory, University of Ghent, Belgium. The cut-off for the anti-CS ELISA was 0.5 EU/mL. Serum samples with a titer below the cut-off value were given a value of 0.25 EU/mL.

## Data collection, data management and statistical analysis

At each study site, data were remotely entered on electronic case report forms and transferred to GSK Vaccines for data management. External monitors reviewed medical records, sample storage, and laboratory procedures to ensure data integrity.

In order to preserve the blinding of the ongoing trial, all data cleaning processes were blinded to study group and analyses were conducted by an external statistician (4Clinics) who performed the analyses using SAS Drug Development (SDD) version 3.5 on SunOS/5.10. (SAS Institute Inc., Cary, NC, USA) based on a cleaned dataset and quality controlled programs provided by GSK Vaccines.

## Contribution to the per-protocol analyses

To be included in the per-protocol analysis of efficacy at Month 20, participants enrolled in each age-category must have received three doses of RTS,S/AS01 or control vaccine according to protocol procedures within specified intervals and contributed to the time at risk in the follow-up period starting 14 days post dose-3. Participants unblinded by the safety department were also excluded from the per-protocol population for efficacy. In addition participants in the 6-12 weeks age-category must have received three doses of co-administered vaccine (DTPwHepB/Hib and OPV).

To be included in the per-protocol analysis of immunogenicity, participants must have received all vaccinations according to protocol procedures. Subject should also have followed protocol defined intervals for vaccinations and blood sampling schedules. Participants with protocol deviations in terms of administration of concomitant vaccinations (in the 6-12 weeks age-category), screening procedures or participants unblinded by the safety department or investigators were excluded from the per-protocol analysis of immunogenicity.

## Statistical methods for the analyses of efficacy at Month 20

We now report the VE during an 18-month follow-up after the third dose of vaccine overall and by site. The study protocol was amended to include this analysis at 18 months post dose-3 in response to a request from the WHO and site specific data are included for the first time to help explain the findings of the previous analysis. Details on the analyses performed and reported in this manuscript are presented in Supplementary table 18.

- ***Presentation of results by transmission intensity***

The incidence of clinical malaria meeting the secondary case definition (a measured or reported fever within the previous 24h and a parasite density >0 parasites per cubic millimeter) in infants in the control group measured over 12 months of follow-up was used to categorize malaria transmission across study sites. This measure was used because it most closely reflects force of infection and is less influenced by acquired immunity that might reduce clinical malaria incidence in older children. For all tables and figures, study sites are presented from the lowest to the highest incidence of clinical malaria.

- ***Vaccine efficacy against clinical malaria***

Vaccine efficacy against all episodes of clinical malaria was estimated as 1-IR where IR is the incidence ratio (total number of events/follow-up time in the RTS,S/AS01 group over the total number of events/follow-up time in the control group) calculated by negative binomial regression allowing for interdependence between episodes within the same subject (mixed model with overdispersion parameter estimated from the random effect) and presented together with 95% confidence interval (CI) and p-values calculated from this model. VE estimates were unadjusted for covariates. Fourteen days following an episode meeting the case definition under evaluation were subtracted from the follow-up time. Results are presented per site and overall (adjusted for study site). The p-value for the interaction term between site and group allocation is presented with p < 0.10 indicating a significant inter-site difference.

Vaccine efficacy against first or only episodes of clinical malaria was estimated as 1-HR where HR is the hazard ratio (log survival in the RTS,S/AS01 group over log survival in the control group) calculated by Cox regression models and presented together with 95% CI and p-values (likelihood ratio test). Vaccine efficacy estimates were unadjusted for covariates. Results are presented per site and overall (stratified for study site).

- ***Vaccine efficacy against severe malaria, incident anemia, malaria hospitalization, fatal malaria and against other serious illnesses***

Severe malaria, incident severe anemia, malaria hospitalization, fatal malaria, sepsis, hospitalized pneumonia, all-cause hospitalization and all-cause mortality were analyzed by the proportion of children affected. VE was estimated as 1-RR where RR is the risk ratio (proportion of participants reporting the event in the RTS,S/AS01 group over the proportion in controls) over the entire follow-up period, and presented together with 95% CIs and p-values. Vaccine efficacy estimates were unadjusted for covariates. Results are presented per site and overall.

- ***Vaccine efficacy against prevalent parasitemia and prevalent anemia***

VE against prevalent endpoints (parasitemia, moderate and severe anemia) assessed at study Month 20 (i.e. 18 months post dose-3) was estimated as 1-RR where RR is the risk ratio (proportion of participants reporting events in the RTS,S/AS01 group over the proportion in controls) and was presented together with 95%CIs and p-values. VE estimates were unadjusted for covariates. Results are presented per site and overall. The geometric mean parasite density and arithmetic mean hemoglobin level were calculated per site and overall. The effect of the group was evaluated using the t-test.

- ***Vaccine efficacy over time***

To evaluate potential waning, Schoenfeld residuals and models with time-varying covariates (failure time^-2^, failure time^-1^, failure time^-0.5^, log(failure time), failure time, failure time^0.5^, failure time^2^, piecewise) were evaluated on Cox models on first or only episodes and including multiple episodes of clinical malaria (Andersen and Gill) for each site and overall. Graphical presentations of VE over time using the best model fit are presented. Note that analyses to detect differences from constant efficacy are dependent on the number of malaria episodes. As a (mathematical) result, low transmission sites are less likely to show waning efficacy (Supplementary figure 4).

The incidence of clinical and severe malaria (total number of episodes/person year) was calculated by 6-monthly periods (1-6 months, 7-12 months and 13-18 months post dose-3) and presented by group (RTS,S/AS01 versus controls) for each site and overall. Incidence comparisons were performed for each of the three time periods and presented with 95% CIs and p-values for clinical malaria endpoints. The reduction in incidence should be interpreted with caution: these results are expressed as a reduction of malaria incidence in the RTS,S/AS01 group compared to the control group and not a vaccine efficacy because, when dividing the analysis by 6-month breakdown, the randomization is lost for the second and third periods (7-12 and 13-18 months) and important differences may exist between groups at the beginning of each time period.

- ***Vaccine impact***

For each site and overall, the number of cases of clinical malaria, severe malaria, and malaria hospitalizations averted was calculated (difference between incidences, expressed per 1000 population vaccinated) for each 6-monthly time period and totaled over the entire follow-up period. The more sensitive secondary case definitions of clinical malaria (a measured or reported fever within the previous 24h and a parasite density >0 parasites per cubic millimeter) and severe malaria were used for evaluation of the impact of RTS,S/AS01 on the burden of malaria because, in clinical practice, these children would receive treatment for malaria. Graphical presentations were also generated, showing the cumulative number of averted cases across the three 6-monthly periods of follow-up.

To evaluate effect on growth, height for age, weight for age and mid arm circumference, z-scores for each age-category as well as the absolute height (for the 6-12 weeks age-category only) at Month 20 were tabulated and the mean values were compared between study groups using a t-test.

- ***Exploratory model of protection***

To investigate the hypothesis that the transmission intensity (measured by “incidence of clinical malaria in control infants”) could be a driver of difference of efficacy between sites, a statistical model was tested that includes “incidence in controls” in the respective age category as a main effect and as an interaction term on the number of episodes of clinical malaria experienced by participants (negative binomial regression model). The main effect refers to the impact of transmission intensity on the number of episodes of clinical malaria and was included because it allowed interpretation of the interaction effect. The interaction effect reflects whether the influence of the main effect differed between study groups, and allowed the question whether VE differed by the levels of transmission intensity to be answered. A limitation of this model is that transmission intensity is more accurately estimated by measuring the incidence of malaria infection using active surveillance. Clinical malaria incidence is influenced by acquired immunity, geographical factors and care-seeking behavior. The high level of clinical care provided at all sites is likely to have minimized differences between sites in the latter. None-the-less, variations that exist between sites may impact our analysis of VE by transmission level.

Because sites may also differ with respect to a number of other covariates influencing the incidence of clinical malaria, these covariates were included in the model as main and interaction effects. The interaction effects could be interpreted in an analogous fashion to that described above for transmission intensity. The full model included the following variables: group (RTS,S/AS01 versus control); incidence in controls in the respective age category; anti-CS antibody GMT at one month post dose-3 in RTS,S/AS01 recipients; indoor residual spraying; bednet use; distance to outpatient facility; age at first dose; Intermittent Preventive Treatment in Infants (IPTi) use; height for age z score (HAZ at baseline); weight for age Z score (WAZ) at baseline; anemia and all these variables interactions with group. Height for age and weight for age z-scores have been derived from height and weight measurements, using standard WHO growth charts.

This model was tested for the per-protocol population for efficacy using the primary case definition of clinical malaria. Participants with missing data on covariates were not included. A first model included all covariates and their interaction with group. A final model excluded the interaction effects from Model 1 that did not reach statistical significance at the 0.05 level.

- ***Exploratory model: determinants of anti-CS antibody titer***

Within the RTS,S/AS01 recipients, the risk of clinical malaria (primary case definition) was modeled as a function of post vaccination anti-CS responses one month post dose-3 for the first 200 participants enrolled in each age-category at each site by negative binomial regression. The following covariates were taken into account: anti-CS antibody at baseline; age at first dose; incidence in controls; vitamin A usage; HAZ at baseline; WAZ at baseline and hepatitis B vaccine priming prior to RTS,S/AS01 vaccination (in the 5-17 months age-category; at least one dose/no priming). This model quantified the effect of post dose-3 level of anti-CS on the risk of clinical malaria.

A second model assessed the effect of the above mentioned covariates on the post dose-3 level of anti-CS response with linear regression analysis, applied on the same set of study participants. To assess potential problems of collinearity among the predictor variables, the variance inflation factor was estimated from the model. Univariate analysis of post vaccination anti-CS responses by different levels of covariates (age by month/weeks, number of prior hepatitis B vaccine doses) was also performed (Information on prior hepatitis B vaccination among children aged 5-17 months was obtained from children’s health cards, or when unavailable, from a mother’s report).

## Additional analyses

Previous analyses have evaluated vaccine efficacy against clinical malaria over 12 months post third vaccine dose in the first 6000 participants enrolled in the 5-17 months age-category and these results have been reported previously.[6] Overall, the trial enrolled 8923 children in the 5-17 months age-category, therefore, it was decided during the development of the statistical analysis plan, and prior to the performance of any statistical analysis, to add an analysis of vaccine efficacy over 12 months of follow-up for all participants enrolled in the 5-17 months age-category and the results of this analysis are reported here (Supplementary figure 8 and Supplementary table 16).

In order to evaluate the vaccine efficacy over time, an analysis of the incidence of clinical malaria and severe malaria calculated by 6-monthly periods was added to the statistical analysis plan, prior to the performance of any statistical analysis.

Per-protocol, an analysis of cases averted was planned to be performed at trial end. It was decided during the development of the statistical analysis plan, prior to the performance of any statistical analysis, to add an analysis of cases averted for clinical malaria, severe malaria and malaria hospitalization at Month 20 to evaluate the impact of the vaccine up to 18 months post third vaccine dose.

In order to investigate key elements linked to protection and key elements affecting the anti-CS response, exploratory models evaluating malaria incidence over time and interactions (determinants of protection) as well as exploratory models on immune responses (determinants of anti-CS response, efficacy and anti-CS response) were added in the statistical analysis plans, prior to the performance of any statistical analysis.

## Major protocol deviations

Deviations related to defaults in bednet distribution at screening and exposure of study vaccines to temperatures outside the recommended ranges were described in detail when the first results of this trial were reported. [6] These deviations did not pertain to participants enrolled in the 6-12 weeks age-category. No other protocol deviations were detected which had the potential to influence the integrity of the results of the analyses at Month 20 presented here.

## Trademarks

*“Tritanrix HepB/Hib, Polio Sabin, Hiberix and Tritanrix HepB are registered trademarks of the GlaxoSmithKline group of companies. Menjugate is a trademark of Novartis. VeroRab is a trademark of Sanofi-Pasteur”*

# Groups that have contributed to the delivery of this trial

Writing group (September 2013): Salim Abdulla (Chair), John Aponte, Brian Greenwood, Mary J Hamel, Didier Leboulleux, Amanda Leach, Marc Lievens, Patricia Njuguna, Aurélie Olivier, David Schellenberg, Marcel Tanner, Johan Vekemans, John Lusingu, Lucas Otieno.

Statistical working group: John Aponte, Marc Lievens (Chair), Bruno Mmbando, Ali Mohamed Ali, John Williamson, Wasima Rida.

Clinical Trials Partnership Committee (September 2013): Salim Abdulla, Tsiri Agbenyega, Selidji Agnandji Todagbe, Daniel Ansong, Kwaku Poku Asante (Co-Chair), Umberto D'Alessandro, Samwel Gesase, Brian Greenwood, Mary J. Hamel, Tinto Halidou (Chair), Irving Hoffman, Simon Kariuki, David Kaslow, Peter Gottfried Kremsner, Didier Lapierre, Amanda Leach, Didier Leboulleux, John Lusingu, Eusebio Macete, Kevin Marsh, Francis Martinson, Patricia Njuguna, Lucas Otieno, Walter Otieno, Aoife Pauley, David Poland, Barbara Savarese, Jahit Sacarlal, Marcel Tanner, Johan Vekemans.

# References

1. Leach A, Vekemans J, Lievens M, et al. Design of a phase III multicenter trial to evaluate the efficacy of the RTS,S/AS01 malaria vaccine in children across diverse transmission settings in Africa. Malar J 2011;10:224.
2. ICH Harmonised Tripartite Guideline. Good Clinical Practice: Consolidated Guideline. International Conference on Harmonization of Technical Requirements for Registration of Pharmaceuticals for Human Use (ICH). 1996. Section 6 “Clinical Trial Protocol and Protocol Amendment(s)” and Section 8 “Essential Documents for the Conduct of a Clinical Trial.”
3. WHO. Pocket book of Hospital care for children; Guidelines for the management of common illnesses with limited resources. World Health Organization, Geneva, 2005. (Accessed June 12, 2013 at http://whqlibdoc.who.int/publications/2005/9241546700.pdf)
4. Cohen J, Nussenzweig V, Nussenzweig R, Vekemans J, Leach A. From the circumsporozoite protein to the RTS, S/AS candidate vaccine. Hum Vacc 2010;6:90-96.
5. Bonhoeffer J, Menkes J, Gold MS, et al. Generalized convulsive seizure as an adverse event following immunization: case definition and guidelines for data collection, analysis and presentation. Vaccine 2004;22:557-562.
6. The RTS,S Clinical Trials Partnership. First Results of a Phase 3 Trial of RTS,S/AS01 Malaria Vaccine in African Children. N Engl J Med 2011;365:1863-1875.
7. The RTS,S Clinical Trials Partnership. A Phase 3 Trial of RTS,S/AS01 Malaria Vaccine in African Infants. N Engl J Med 2012;367:2284-2295
8. WHO. Verbal Autopsy Standards: ascertaining and attributing cause of death. World Health Organization, Geneva, 2007. (Accessed June 12, 2013 at http://apps.who.int/iris/handle/10665/43764)
9. Vekemans J, Marsh K, Greenwood B et al. Assessment of severe malaria in a multicentre, phase III, RTS,S/AS01 malaria candidate vaccine trial: case definition, standardization of data collection and patient care. Malar J 2011;10:221.
10. WHO. Standardization of interpretation of chest radiographs for the diagnosis of pneumonia in children. World Health Organization, Geneva, 2001. (Accessed June 12, 2013 at http://www.who.int/vaccine_research/diseases/ari/www616.pdf)
11. Cogill, B. Anthropometric Indicators Measurement Guide. Food and Nutrition Technical Assistance Project, Academy for Educational Development. Washington, DC, 2003.
12. Swysen C, Vekemans J, Bruls M, et al. Development of standardized laboratory methods and quality processes for a phase III study of the RTS,S/AS01 candidate malaria vaccine. Malar J 2011;10:223.
13. Mahon CR, Manuselis G. Textbook of Diagnostic Microbiology. 2nd Edition. Philadelphia, USA: Saunders, 2000.
14. Winn W, Allen S, Janda W, et al. Koneman’s Color Atlas and Textbook of Diagnostic Microbiology. 6th ed. Philadelphia, USA: Lippincott Williams and Wilkins, 2006.
15. WHO. The WHO Young Infants Study Group. Bacterial etiology of serious infections in young infants in developing countries: results of a multicentre study. Pediatr Infect Dis J 1999;18:S17-S22.
16. Berkley JA, Mwangi I, Ngetsa CJ, et al. Diagnosis of acute bacterial meningitis in children at a district hospital in sub-Saharan Africa. Lancet 2001;357:1753-57.
17. Clement F, Dewar V, Van Braeckel E et al. Validation of an enzyme-linked immunosorbent assay for the quantification of human IgG directed against the repeat region of the circumsporozoite protein of the parasite Plasmodium falciparum. Malar J 2012;11:384.
