# Supplementary material for: Efficacy and Safety of the RTS,S/AS01 Malaria Vaccine during 18 Months after Vaccination: A Phase 3 Randomized, Controlled Trial in Children and Young Infants at 11 African Sites
Source: PLoS Med. 2014 Jul 29;11(7):e1001685. doi: 10.1371/journal.pmed.1001685 (PMC4114488; doi:10.1371/journal.pmed.1001685)
Supplement: Text S2 — List of institutional review boards. (DOC) [file pmed.1001685.s030.doc]

List of Independent Ethics Committees/Institutional Review Boards

| **Center Number(s)** | **Ethics Review Body** | **Location** |
| --- | --- | --- |
| 041087 (Lambaréné, Gabon), 041089 (Kintampo, Ghana), 041092 (Agogo, Ghana), 041104 (Kilifi, Kenya), 041105 (Manhiça, Mozambique), 041106 (Bagamoyo, Tanzania), 041107 (Korogwe, Tanzania), 041108 (Kombewa, Kenya), 053530 (Nanoro, Burkina Faso), 053597 (Lilongwe, Malawi), 053598 (Siaya, Kenya) | Western Institutional Review Board (WIRB) | 3535 Seventh Avenue, SW-Olympia, WA 98502 – 5010, USA |
| 053530 (Nanoro, Burkina Faso) | Comité d’Ethique Institutionnel du Centre Muraz | 01 BP 390  Bobo-Dioulasso 01  Burkina Faso |
|  | Comite d’Ethique pour la Recherche en Santé | BP 7009  Ouagadougou 03  Burkina Faso |
| 041104 (Kilifi, Kenya), 041108 (Kombewa, Kenya), 053598 (Siaya, Kenya) | Kenya Medical Research Institute (KEMRI) National Ethics Review Committee | P.O. Box 54840  00200 Nairobi, Kenya |
| 053598 (Siaya, Kenya) | Centres for Disease Control (CDC) – IRB | Office of the Chief Science Officer, Human  Research Protection Office  1600 Clifton Rd.NE, M/S D-73 Atlanta, GA 30033, USA |
| 041108 (Kombewa, Kenya) | Walter Reed Army Institute of Research (WRAIR) IRB | 503 Robert Grant Avenue  Silver Spring, MD 20910-7500, USA |
| 041089 (Kintampo, Ghana) | Kintampo HealthResearch Centre (KHRC) Institutional Ethics Committee (IEC) | P.O. Box 200, Kintampo, Brong Ahofo Region, Ghana |
| 041089 (Kintampo, Ghana), 041107 (Korogwe, Tanzania) | London School of Hygiene and Tropical Medicine Ethics Committee | Keppel Street, London WC1E7HT, UK |
| 041106 (Bagamoyo, Tanzania), 041107 (Korogwe, Tanzania) | National Institute for Medical Research (NIMR) | P.O. Box 9653  Dar-es-Salaam, Tanzania |
| 041106 (Bagamoyo, Tanzania) | Ethikkommission beider Basel (EKBB)  (Ethics Committee of the Swiss Tropical Institute) | Schweizerisches Tropeninstitut (STI)  Socinstrasse 57, 4002 Basel, Switzerland |
| 041089 (Kintampo, Ghana), 041092 (Agogo, Ghana) | Ghana Health Service (GHS) Ethical Review Committee (ERC)  Research and Development Division | Ghana Health Service  P.O. Box MB 190  Accra, Ghana |
| 041092 (Agogo, Ghana) | Committee on Human Research Publication and Ethics (CHRPE) | Private Mail Bag  University Post Office  Kumasi, Ghana |
| 041106 (Bagamoyo, Tanzania) | Ifakara Health Institute research training services - IRB | P.O. Box 78373  Dar es Salaam, Tanzania |
| 041087 (Lambaréné, Gabon) | Comité d’Ethique Régional Indépendant de Lambaréné (CERIL)  (Independent Regional Ethics Committee of Lambaréné) | P.O. Box 250  Lambaréné, Gabon |
|  | Comité National d’Ethique pour la Recherche  (National Ethics Committee for Research  The Board) | P.O. Box 2217  Libreville, Gabon |
| 041107 (Korogwe, Tanzania) | University of Copenhagen  Centre of Medical Parasitology | CSS Building 22 + 23  Oster Farimagsgade 5  1014 Kobenhavn K |
| 053597 (Lilongwe, Malawi) | National Health Sciences Research Committee | Ministry of Health and Population  P.O. Box 30377  Lilongwe 3, Malawi |
|  | Office of Human Research Ethics | University of North Carolina  Medical School Building 52  Mason Farm Road  CB# 7097  Chapel Hill, NC27599-7097, USA |
| 041105 (Manhiça, Mozambique) | Comitè Ético de Investigación Clinica (Barcelona Ethical Committee) | Hospital Clinic I Provincial De Barcelona  Villarroel  170-08036 Barcelona, Spain |
|  | Comité Nacional de Bioética para a Saúde  (National Bioethical Health Committee) | Ministério Da Saúde  C. Postal 264 Av. Eduardo Mondlane/ Salvador Allende  Maputo, Mozambique |
